# Standard laboratory housing for mice restricts their ability to segregate space into clean and dirty areas

**DOI:** 10.1038/s41598-019-42512-3

**Published:** 2019-04-16

**Authors:** I. Joanna Makowska, Becca Franks, Cathy El-Hinn, Tina Jorgensen, Daniel M. Weary

**Affiliations:** 1 Animal Welfare Program, University of British Columbia, 2357 Main Mall, Vancouver, BC V6T 1Z4 Canada; 2 Department of Environmental Studies, New York University, 285 Mercer St, New York City, NY 10003 USA; 3 Western College of Veterinary Medicine, University of Saskatchewan, 52 Campus Drive, Saskatoon, SK S7N 5B4 Canada; 4 Centre for Comparative Medicine, University of British Columbia, 4145 Wesbrook Mall, Vancouver, BC V6T 1W5 Canada

## Abstract

Laboratory mice *(Mus musculus)* are typically housed in simple cages consisting of one open space. These standard cages may thwart mouse ability to segregate resting areas from areas where they eliminate, a behaviour that is prevalent across the animal kingdom. No scientific work has directly tested whether mice engage in such segregation behaviour, or whether the ability to do so may have welfare consequences. Here we show that mice, whether housed in standard cages or a complex housing system consisting of three interconnected standard cages, kept nesting and elimination sites highly segregated, with nest and urine co-occurring in the same location only 2% of the time. However, mice in the complex system established these clean and dirty sites in separate cages instead of separate locations within one cage, and carried bedding materials (cellulose pellets) from their nesting cages to their latrine cage. Moreover, mice in the complex system displayed more behaviours associated with positive welfare and were less disturbed by weekly husbandry procedures. We conclude that mice find waste products aversive, and that housing mice in a way that facilitates spatial segregation provides a simple way of allowing the expression of natural behaviours and improving welfare.

## Introduction

Segregation of resting areas from areas where animals defaecate and urinate is widespread in the animal kingdom. Among the insects, various species of ants, aphids, bees, crickets and termites designate specific locations within their dwellings for the deposition of waste^1^. The leaf-cutting ant *Atta colombica* excretes outside the nest, and specialised ‘waste worker’ ants carry the excreta away from nest entrances^2^. Caterpillar larvae use hydrostatic pressure to expel faecal pellets as far as 40 body lengths away from their shelters^3^. Among the birds, most nesting species carry their chicks’ faecal sacs away from the nest^4^, while a burrowing species – the puffin – creates a designated toilet area near the burrow’s entrance^5^. At least two species of reptiles, yakka skinks^6^ and thorny devils^7^, defaecate in designated areas away from their burrows and basking sites. The use of latrines has also been documented in a wide range of mammalian species, from antelopes and elephants to lemurs, river otters, and several rodent species^8–20^. Two commonly proposed functions of this segregation are predator avoidance^20,21^ and reduced risk of disease^2,11^. In humans, it is believed that disgust – one of Darwin’s six basic emotions^22^ – evolved as a mechanism to protect against the risk of infection^23^. Some of the most reliable elicitors of disgust in people are faeces and urine^24^.

Mice (*Mus musculus*) are commonly used in biomedical research. When used in laboratories, mice are typically housed in cages that consist of a single, small, open space. This cage may thwart the animals’ ability to move away from their own excrement. There is indirect evidence that mice prefer to separate elimination behaviour from nesting activity. For example, one study of bedding preferences found that when mice were housed in a multi-cage system containing different types of bedding, they spent the most time and nested in cages containing shredded filter paper, but tended to defaecate and urinate in cages containing sawdust^25^. Another study with a similar multiple-choice set-up found that mice spent the most time in a cage containing fresh bedding rather than in cages that contained bedding that had been soiled for 1, 7, or 14 days^26^. While this previous research suggests that the segregation of nesting and soiling sites may be important to mice, no scientific work has directly tested whether standard cages allow mice to segregate space into clean and dirty areas, or whether the ability to segregate these activities has welfare consequences.

Proximity to faeces and urine can result in exposure to high ammonia levels^27,28^. Moreover, the inability to perform highly motivated behaviours (e.g., moving away from an area one is motivated to avoid) can also cause frustration or anxiety and may lead to disturbed social behaviour, abnormal behaviours, and behavioural suppression^29–31^. In mice, low levels of activity are associated with suppressed normal behaviour^30^ and can result in abnormal physiological profiles and morbidity^32^. For social animals, maintaining voluntary proximity with conspecifics and allogrooming are associated with positive welfare^33^. Affiliative behaviours are believed to strengthen social bonds, resulting in reduced aggression and better group cohesion.

**Figure 1:**
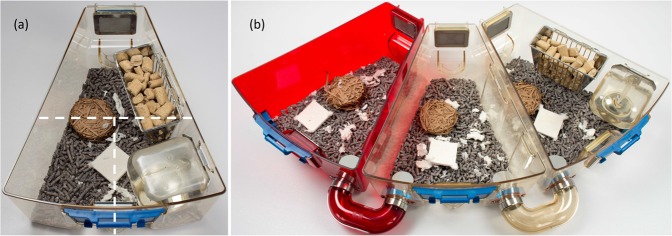
The standard and the complex housing systems. (**a**) Each standard cage was provided with food, water and two types of nesting material. The three locations used to score activities were: front-right (near the water bottle and part of the feeder); front-left (the most open space in the cage); and back (semi-open space behind and under part of the feeder at the back of the cage). (**b**) The complex system consisted of three cages connected by external tunnels. In each triad, one cage was red polycarbonate and placed on one end. Food and water were only available in one of the amber cages, and the position of this food and water cage was alternated between the end and the middle position every two weeks. Two types of nesting material were provided in each cage of the triad. The three locations used to score activities were: red cage; neutral cage; and food and water cage. The photographs were taken by Dr. Bev Chua, Staff Veterinarian and Communications Director at Animal Care Systems. *Note*: The bedding in the photographs is different from that used in this study (pictured here, Estes’ Cell Sorb Plus). The correct bedding can be seen in Supplementary Information.

The general aims of this study were to investigate how mice use space in a standard laboratory cage (Fig. 1a) versus a complex housing system that facilitates spatial segregation (Fig. 1b), and how the two systems affect behavioural indicators of welfare. The first specific aim was to investigate the location of nesting and elimination sites relative to each other (distance between the two sites) and relative to other environmental features (e.g., proximity to food and water, light intensity, accessibility of the specific area). We were also interested in the relationships between these factors and nest quality. We hypothesised that mice would establish separate sites for nesting and elimination, and that they would place a larger distance between these sites in the complex housing system. The second specific aim was to investigate behavioural patterns in the two systems and whether these were differentially affected by weekly husbandry procedures. We hypothesised that mice in the complex system would display more behaviours associated with positive welfare (e.g., affiliative behaviours, locomotion) and fewer behaviours associated with poor welfare (e.g., agonistic and abnormal behaviours). We also hypothesised that mice in the complex system would be less disturbed by weekly husbandry procedures.

## Results

### Nesting and soiling

**Figure 2:**
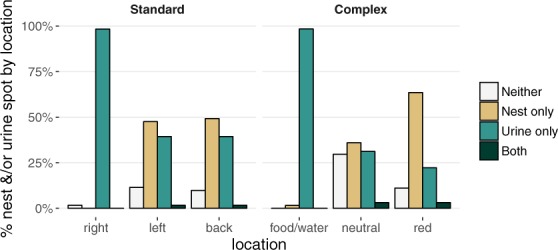
Nest and/or urine spot sites by location within each housing system. In both systems, the location with food and water almost always contained a urine spot, while the other two locations contained either a urine spot or a nest, but almost never both. In the standard system (*n* = 61 observations of nesting and urination sites by standard system), right denotes front-right (this location contained food and water), left denotes front-left, and back denotes back of the cage; in the complex system (*n* = 63 observations of nesting and urination sites by complex system), food/water denotes the food and water cage, neutral denotes the neutral cage, and red denotes the red cage.

Mice in both systems usually built only one nest (nest score of 3 or higher). The mean ± standard error (SE) number of nests was 1.00 ± 0.05 in the standard system vs. 1.08 ± 0.05 in the complex system (GLMM: t(8.23) = 1.13; p > 0.2). The mice also averaged just under two locations for urination spots. The mean ± SE number of urine spots was 1.80 ± 0.10 in the standard system vs. 1.79 ± 0.09 in the complex system (GLMM: t(7.68) < 1; p > 0.9). Mice kept these sites highly segregated in both housing systems: nests and urine spots occurred in the same location only 2% of the time. In contrast, locations contained only a nest 33% of the time or only a urine spot 55% of the time (Fig. 2). As such, regardless of system, the presence of a nest in a location was predictive of the absence of a urine spot (GLMM: z = 9.38, p < 0.0001). The mice in the complex system separated their nest and urine spot by a mean ± SE distance of 1.42 ± 0.07 cages, whereas the mice in the standard system were constrained to build their nest and urinate in the same cage. Moreover, the mean ± SE nest quality was higher in the complex compared to the standard system (4.31 ± 0.13 vs. 3.86 ± 0.13; GLMM: t(7.81) = 2.61; p < 0.04).

In the standard system, the mean ± SE soiling on cage-change day was 2.07 ± 0.06. In the complex system, the mean ± SE soiling of the most-soiled cage on cage-change day was 2.78 ± 0.10, but only 0.82 ± 0.07 for the other two cages. In the complex system, soiling levels were higher at the end of the week after a latrine-only change vs. a full change (on average, 0.28 ± 0.09 units higher across all cages; GLMM: t(162.23) = 3.11; p < 0.003). In the complex system, which allowed us to measure soiling level by location (i.e., cage-level), we found that as soiling increased in the cage, the probability of finding a urine spot in that cage also increased (GLMM: z = 6.63, p < 0.0001), and that the probability of finding a nest in that cage decreased (GLMM: z = 6.89, p < 0.0001).

Within each housing system, mice established preferred locations for elimination and nesting (Fig. 2). In the standard system, mice usually urinated in the front-right location near the water bottle and part of the feeder: the probability of finding a urine spot in the front-right location was 98% vs. 42% and 40% for the other two locations (GLMM LRT: χ2(2) = 71.85, p < 0.0001; Fig. 2). The front-right location was never used for nesting; instead, mice built their nest in the other two locations equally: probability of finding a nest in the front-right location was 0% vs. 50% for each of the other two locations (GLMM LRT: χ2(2) = 54.65, p < 0.0001; this model is slightly biased towards a conservative estimate of the effect: logistic regression models cannot run when there is perfect predictive value, so we had to change one of the observations from “nest absent” to “nest present” for the front-right location; Fig. 2). The mean ± SE quality of a nest was higher for nests built in the front-left compared to the back of the cage (4.03 ± 0.14 vs. 3.68 ± 0.14; GLMM: t(54.94) = 2.20; p < 0.04).

In the complex system, the food and water cage was most likely to contain a urine spot: the probability of finding a urine spot in the food and water cage was 98% vs. 34% and 27% for the neutral and red cages, respectively (GLMM LRT: χ2(2) = 97.42, p < 0.0001; food/water cage vs. neutral and red cages: z = 4.58, p < 0.0001 and z = 4.91, p < 0.0001, respectively; Fig. 2). The food and water cage was also the most heavily soiled: mean ± SE soiling score in the food and water cage was 2.66 ± 0.10 vs. 1.33 ± 0.10 and 0.51 ± 0.10 for the neutral and red cages, respectively (GLMM LRT: χ2(2) = 179.12, p < 0.0001; food/water cage vs. neutral and red cages: z = 10.62, p < 0.0001 and z = 17.11, p < 0.0001, respectively). Mice preferred to nest in the red cage: probability of finding a nest in the red cage was 67% vs. 39% and 2% in the neutral and food and water cages, respectively (GLMM LRT: χ2(2) = 72.58, p < 0.0001; red cage vs. neutral and food/water cages: z = 3.07, p < 0.003 and z = 4.64, p < 0.0001, respectively; Fig. 2).

**Figure 3:**
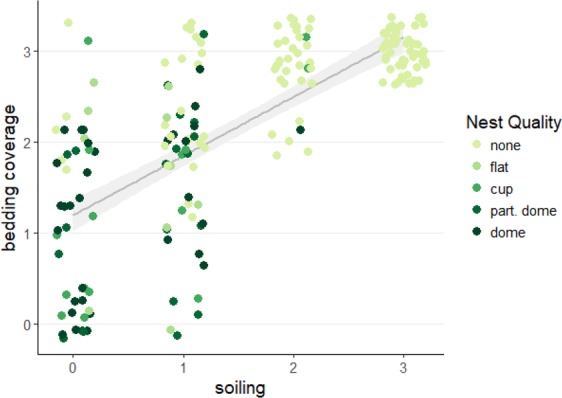
Nest quality by soiling and bedding coverage scores in the complex system. Higher quality nests were associated with cages that had lower soiling and lower bedding coverage scores, while lower quality nests or no nests were associated with cages that had higher soiling and higher bedding coverage scores. These results indicate that mice moved bedding out of the nesting cages and into the latrine cages. Each point represents one cage assessment. Points (*n* = 192 observed nests) are jittered slightly to avoid overlap, and the line and shaded area represent model prediction and 95% confidence interval.

Bedding coverage in the standard system was nearly complete (average score was 2.95 ± 0.03; on three occasions some bedding was built into the nest), as would be expected for a closed system. In the complex system, mice moved bedding out of the nesting cages and into the soiled cages: bedding coverage scores increased with increasing soiling level and decreased with increasing nest quality (GLMM soil and nest effects respectively: b = 0.46, t(174.02) = 7.90, p < 0.0001; b = −0.22, t(170.28) = 5.40, p < 0.0001; Fig. 3). Interestingly, including cage-type in the model did not affect the nature of these results (GLMM with cage-type as a covariate: nest and soil effects respectively: b = −0.22, t(165.83) = 5.40, p < 0.0001; b = 0.47, t(181.35) = 6.04, p < 0.0001) suggesting that how the mice used the space (i.e., nesting vs. soiling) was more predictive of bedding coverage than cage-type (i.e., red vs. food and water vs. neutral), which was a non-significant predictor of bedding coverage in the full model (GLMM of cage-type effects: LRT: χ2(2) = 1.31, p > 0.5).

### Behaviours

Instances where the focal mouse appeared to be influenced by the observer were rare, and we did not find evidence that the mice were differentially influenced in the two housing systems (3.4% and 6.7% of observations in complex and standard systems, respectively; z = 1.40; p > 0.16).

The percent of time behaviours were observed in each system is presented in Table 1. We tested the effect of housing system on those behaviours occurring at a high enough frequency to model statistically (i.e., occurring at a raw base-rate of at least 5% of time in at least one of the systems): affiliative behaviours (allogrooming, rest with partner, not visible in nest with partner), nest building, maintenance behaviours (self-groom, eat, drink), locomotion, rest alone, and not visible in nest alone.

**Table 1:** Ethogram with descriptions, and percent time the behaviours were observed in the standard and the complex housing systems.

Behaviour	Sub-behaviour	Description	Percent of time	
			Standard	Complex
Abnormal		Behaviour that is disturbing, destructive or detrimental to the physiological, psychological and social well-being of a mouse and cage mates	2.8%	1.3%
	Bar-mouthing	Sham biting on a fixed area of the feeder bars	0.0%	0.0%
	Circle	Repetitive tracing of a loosely circular path	0.0%	0.0%
	Vacuum digging	Stationary digging of cage floor after bedding was dug out of the way	2.8%	1.3%
Affiliative**		***Behaviour that functions to develop or strengthen social bonds***	***38.7%***	***49.5%***
	Allogrooming	Being licked or licking the fur of another mouse	1.3%	1.1%
	Not visible with partner(s)	Focal mouse and at least one other mouse are not visible in the nest	26.3%	29.4%
	Rest with partner(s)	Lying down with another mouse (less than approximately 0.5 cm apart)	11.1%	19.0%
Agonism		Social interaction that allows mouse to gain control of actual or future resources at the expense of another mouse	0.2%	0.6%
	Attack	Aggressive act where the focal mouse is the actor or receiver of an attack or a bite	0.2%	0.0%
	Chase	Following or being followed immediately before or after an attack	0.0%	0.4%
	Tunnel war	Two or more mice trying to go in opposite directions in the tunnel	n/a	0.2%
Climb		Suspended from the feeder	2.6%	1.9%
Frisky		Sudden skipping, hopping, jerky bouncy movements	1.3%	1.7%
Locomotion**		***General locomotion that is not otherwise defined***	***5.9%***	***12.1%***
**Maintenance**		**Behaviour that maintains the physiological stasis, comfort and appearance of the mouse**	**16.3%**	**13.0%**
	Drink	Licking the nozzle of the drinker	0.7%	0.8%
	Eat	Gnawing food pellets from the feeder	5.4%	4.1%
	Self-groom	Stroking, licking or scratching any part of own body	10.2%	8.1%
Mount		Mounting or being mounted from behind	0.0%	0.0%
Move bedding		Moving bedding material from one cage to another	n/a	0.6%
**Nest build**		**Manipulating bedding, nesting material or nestlet to form a nest**	**9.6%**	**5.8%**
	Carrying	Carrying pieces of bedding or nesting material in mouth	1.1%	0.9%
	Digging	A series of fast alternating movements of the forepaws, scraping back material.		
	Fluffing	Walls of nest appear to jump as the whole nest enlarges		
	Pulling-in	Reaching out of nest and pulling nesting material towards the nest		
	Push-digging	Forward pushing and kicking of bedding material with fast alternating movements of the forepaws		
	Shoveling	Plunging of nose into bedding material and burrowing under		
	Sorting	Placing specific nesting or bedding material into a particular location, while sitting in the nest		
	Fraying	Sideways movements of the forepaws to draw substrate through the mouth	8.5%	4.9%
Not visible alone*		***Only the focal mouse is not visible in the nest***	***5.2%***	***2.3%***
Rear		Raised on her hind legs and extending head upwards	3.3%	3.2%
Rest alone**		***Lying down alone; not in the nest***	***10.7%***	***4.5%***
Sniff (undirected)		Holding nose in the air and away from any identifiable stimulus	2.8%	2.8%
Sniff (social)		Focal mouse is the actor or receiver of an olfactory investigation of a conspecific	0.6%	0.6%

Non-bolded text is used for behaviours that occurred too rarely to meet model assumptions and were therefore not subjected to statistical analysis. Bolded text indicates behaviours that were analysed statistically; bolded and italicised text indicates behaviours that were significantly different between housing systems, where *denotes p < 0.05 and **denotes p < 0.01.

Maintenance and nest building behaviours occurred at similar frequencies in both systems (GLMMs for standard vs. complex: maintenance p > 0.17; nest building p > 0.12; Table 1), but all other behaviours were affected by housing. Compared to the complex system, affiliative behaviours and locomotion occurred less frequently in the standard system (GLMMs for standard vs. complex: affiliative z = 2.90, p < 0.01; locomotion z = 2.78, p < 0.01; Table 1), whereas rest alone and not visible in nest alone occurred more frequently in the standard system (GLMMs for standard vs. complex: rest alone z = 3.07; p < 0.01; not visible alone z = 2.18; p < 0.03; Table 1).

**Figure 4:**
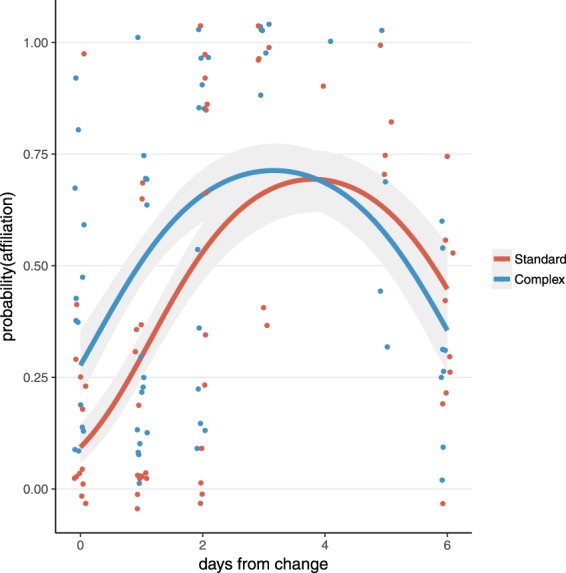
Affiliative behaviours by housing system across the week. The likelihood of observing affiliative behaviour was lower in the standard system vs. the complex system directly after cage-change and followed a quadratic pattern in both systems across the week. Each dot represents the probability of affiliative behaviour in each cage during an observation session (*n* = 119 for 12 days × 5 groups × 2 treatments – 1 day of missing data; dots are jittered slightly to avoid overlap); lines and grey shading reflect quadratic fit and 95% confidence interval, respectively.

Exploring these patterns in more detail revealed that affiliative behaviours were affected by days since cage changing, but more strongly in the standard system than in the complex system. Specifically, an interaction effect indicated the presence of differential effects of days-since-cage-change by housing system (GLMM of housing system by days-since-change interaction: z = 4.35, p < 0.0001). Specific parameter analyses revealed that directly after cage-change, affiliative behaviours were lower in the standard system than in the complex system (GLMM for standard vs. complex system directly after cage-change: z = 4.82, p < 0.0001; Fig. 4). After cage changing, affiliative behaviours in both systems followed a quadratic function: increasing for several days before decreasing again until the next cage-change (GLMM of quadratic effect: z = 9.84, p < 0.0001; Fig. 4).

## Discussion

One relatively unconsidered feature of standard laboratory housing is how it may restrict animals’ ability to segregate daily activities such as nesting and elimination. Here we provide the first direct evidence that mice are motivated to nest away from their urine and faeces. Mice housed in a complex housing system consisting of three interconnected standard cages were better able to segregate nesting and elimination sites and were less disturbed by weekly husbandry procedures. Mice in both systems segregated nesting and urination sites (these occurred in the same location in only 2% of all observations), but mice in the complex system established these sites in separate cages rather than separate locations within a single cage. Unlike in the complex system, where faeces and urine spots occurred in the same location, faeces in the standard system were dispersed throughout all locations, likely because of mixing and moving of bedding during activity^34^. Moreover, mice in the complex system moved the bulk of the bedding from the nesting and neutral cages to the latrine cage, perhaps as a way to further dilute the scent of their waste products: prolonged exposure to high ammonia levels leads to epithelial degeneration, necrosis of the olfactory epithelium and inflammatory lesions in the nasal passages^27^ and deeper bedding has been shown to reduce ammonia levels within a cage^35^. Thus, segregation of nesting and elimination sites appears to be highly motivated in mice. This behaviour is thwarted in standard laboratory cages, but facilitated in the complex housing system.

In addition to restricting segregation, we found that weekly husbandry routines were less disruptive to mice housed in the complex system than mice in the standard system. It is well documented that cage changing is disruptive and leads to increased aggression in male mice; this disruption is likely caused by the removal of odour cues^36,37^. Our results suggest that cage changing is more disruptive to standard-housed compared to complex-housed mice. The expression of affiliative behaviours is associated with positive welfare^33^, and standard-housed mice engaged in fewer affiliative behaviours after cage changing. Some of this effect may be due to the less intensive cage-changing routine in the complex system, which underwent a latrine-only change every second week. The ability to change only the latrine cage – which is less disruptive to the mice and also less labour-intensive – is a unique added benefit of the complex housing system.

Complex-housed mice also exhibited more locomotion. More frequent locomotion may also be associated with better welfare, as the sedentary lifestyle of standard-housed laboratory mice is associated with obesity, hypertension, insulin resistance and premature death^32^, and low activity may be associated with a more general suppression in normal behaviour^30^.

In the complex system, mice defaecated in the same location where they urinated. In the standard system, we did not score defaecation by location (i.e., within the cage), because data appeared to be confounded by the movement of bedding and faeces during activity^34^. In contrast, bedding soaked in urine was heavier and stickier and did not appear to have been displaced. Although we were not able to score defaecation per location in the standard system, it is likely that these mice also defaecated in a localised area; several authors have observed this elimination behaviour in cages where mice were given a demarcated area that prevented the mixing of bedding (e.g., glass dish, bottle or shelter^38–40^).

In the standard cage, mice set up their nest in the front-left and back locations with equal probability. However, nests built in the front-left location were of higher quality, perhaps to better shelter from the light and other disturbances that were more prominent at the front of the cage^41^. In the complex system, mice preferentially built their nest in the red cage, indicating a preference for lower light levels in the nesting area.

Mice built higher quality nests in the complex system compared to the standard system. In the complex system, mice had access to more nesting material in total – but not per capita – and more material was needed to build wider nests (usually taking up the entire cage) to accommodate all nine mice in the system. The resulting difference in nest quality between the two systems was therefore not likely due to the availability of more nesting material to build higher nest walls. Some differences in nest quality may be due to the higher likelihood of mice disturbing the nest by running through it in the smaller standard system compared to the larger complex system.

Several factors are known to affect nest quality, including light level, temperature (mice housed at lower temperatures build better quality nests^42^), and welfare (mice with compromised welfare build lower quality nests^42,43^). With regards to light level, we would have expected the standard-housed mice to build the better nests if sheltering from the light was the main driver for the better nest quality (as mice in the complex system typically nested in the red cage). With regards to temperature, nine mice sharing a nest generate more heat than three mice, so we would have again expected standard-housed mice to build the better nests if thermoregulation was the main driver for the difference in nest quality. Better quality nests in the complex system may therefore be the result of mice faring better in that system: experts have identified ‘use of nesting material’ to be one of the most important measures in the assessment of mouse welfare^43^. Other research has specifically linked aggression, illness, and pain to poor nest building^42^. In the present study, mice in the complex system displayed more behaviours associated with positive welfare (affiliative behaviour and locomotion) and fewer behaviours associated with negative welfare (resting alone and in nest alone). Follow-up studies could test the association between nest quality and other indicators of welfare.

In both housing systems, mice eliminated most in the location that contained food and water. One researcher who designed a cage with a focus on satisfying mouse needs also observed that mice eliminated near their food and water^44^. This researcher explained that mice tended to defaecate and urinate when they were active, especially when eating and drinking^45^. However, elimination during eating and drinking does not seem to be involuntary: in one study, mice had access to four cages each containing food and water, and despite consuming similar amounts of food and water in all cages, mice eliminated the least in the cage where they built their nest and spent the most time^25^. The choice to eliminate near food and water may be the result of an innate preference for eliminating *while* eating and drinking, or it could be driven by a preference for conducting all non-nest related activities in the same location (and away from the nest site^25,26^). As in previous research, the location of food and water was confounded in our complex system. In the standard system, however, food and water were only partially confounded. Of the three locations in the standard system, one contained neither food nor water, one contained both, and one contained only food. The two locations without water (the food-only location and the neither-food-nor-water location) were as likely to have a urine spot as not, but there was almost always (98% of the time) a urine spot in the location that contained food and water. These results suggest that mice may be motivated to urinate near water, a finding that should be considered in the creation of more effective housing for mice.

Affiliative behaviours are considered to be associated with positive welfare because of the physiological and psychological benefits of warmth, security, and strengthening of social bonds^33,46^. Mice in the complex system engaged in more affiliative behaviours and displayed fewer instances of resting alone and being alone in the nest. These findings may be the result of there being more mice in the complex system: for example, the odds of there being another mouse who wanted to rest or spend time in the nest at the same time as the focal mouse were higher when there were more possible partners. However, this cannot explain why the frequency of affiliative behaviours fluctuated more dramatically throughout the week in the standard system. Specifically, the frequency of affiliative behaviours was similar in the two systems mid-week, but was lower in the standard system immediately after cage-change. These results suggest that cage changing may have had a greater effect on mice in the standard system.

In both systems, the frequency of affiliative behaviours followed a quadratic function: affiliative behaviours decreased immediately after cage-change, then increased for three days before decreasing again in the days leading up to the next cage-change. One explanation for this pattern might be that cage changing is disruptive, causing an immediate drop in normal patterns of behaviour; with time mice resume normal affiliative behaviour, but as cage soiling increases, this again disrupts the behaviour. Despite better ability to segregate space in the complex system, mice must still come into contact with the dirty latrine when they feed and drink. Indeed, we found that by the end of the week, the latrine cage was more soiled in the complex system than the standard system. A follow-up study could test if this quadratic pattern is due to soiling by cleaning the latrine cage more frequently.

## Conclusions

Mice value the segregation of nesting sites from elimination sites, but standard laboratory cages thwart this natural behaviour. A complex housing system consisting of three standard cages connected via tunnels allowed mice to set-up their nest and latrine in separate cages, and to carry bedding from clean cages into the latrine. We conclude that mice find waste products aversive, an observation that opens avenues for new research including, for example, determining whether disgust, like pleasure and pain^47^, is highly conserved across species. Housing mice in a way that facilitates spatial segregation provides a simple way of allowing the expression of natural behaviours and improving welfare.

## Methods

This study was approved by the University of British Columbia’s Animal Care Committee (Protocol Number: A15-0091). All procedures were performed in accordance with the Canadian Council on Animal Care guidelines on care and use of mice in research.

### Animals and housing

Sixty female Swiss Webster mice aged 4–6 months were obtained as surplus breeding stock from the University of British Columbia. Before arriving at our facility, and for one month after arrival, these animals were housed in groups of four mice in standard rectangular cages. Three weeks before this study began, mice were randomly regrouped and randomly allocated to standard housing (*n* = 5 groups; 3 mice/group, 15 mice total) or complex housing (*n* = 5 groups; 9 mice/group, 45 mice total). Stocking density was equivalent in the two systems. Power analysis was not performed: the magnitude of treatment differences was not known because this was the first study of its kind. We viewed this project as an investigatory study into the idea of segregation of space. We used what we considered to be the absolute minimum number of cages that could generate usable data, which was *n* = 5 per treatment.

Mice weighed (mean ± standard deviation [SD]) 32.2 ± 3.0 g in the standard system and 32.2 ± 3.6 g in the complex system on the day they were placed into their respective housing systems, and 36.6 ± 3.4 g in the standard system and 36.6 ± 4.8 g in the complex system two weeks before the end of data collection (this was 16 weeks later).

Cages were distributed on five levels of a cage rack, with one housing of each type per level. The location of each housing type was also alternated within each level (left vs. right). Animals were marked with a permanent non-toxic animal marker (Stoelting Co., Wood Dale, IL, USA) for individual identification. Mice were housed on a reversed light cycle with lights on from 1.00 to 13.00 h. Mean ± SD temperature and humidity were 24.2 ± 1.1 °C and 55.2 ± 2.9%, respectively.

Standard housing consisted of an amber polysulfone filtered EVC cage (Optimice®, Animal Care Systems, Centennial, CO, USA) in the shape of a narrow trapezoid with 484 cm^2^ floor area (Fig. 1a). The cage contained bedding (Biofresh™ 1/8″ cellulose pellet + Enrichment, Ferndale, WA, USA) and two types of nesting material: Bed-r′Nest® (8 g, The Andersons, Inc, Maumee, OH, USA) and one Nestlets™ (Ancare, Bellmore, NY, USA). Mice had unrestricted access to food (Laboratory Rodent Diet 5001, Lab Diet, St. Louis, MO, USA) and tap water provided from a feeder and a bottle placed in a permanent location inside the cage. Cages were changed once per week. During cage changing, mice were scooped with a cupped hand (the tail was held loosely for security only) and transferred to a clean cage with a fixed amount of new bedding and nesting materials. About one third of the old nest was scattered across the clean cage to provide a familiar scent in the new cage. A few treats were also provided in the clean cage (sunflower seeds, shredded coconut or breakfast cereal).

Complex housing consisted of three standard cages (a “triad”) connected by two external tunnels (Blockparty®, Animal Care Systems, Centennial, CO, USA; Fig. 1b). One mouse developed a neoplasm and was euthanised during the second week of the study, so one triad housed eight mice instead of nine from this point on.

Complex housing triads underwent a ‘full change’ or ‘latrine-only change’ on alternating weeks. We included latrine-only changes to explore two potential added benefits provided by the complex housing system: reduced labour and reduced disturbance to the mice. We did not measure labour, but “latrine-only” changes necessarily involve less labour-per-mouse than the standard changes, such that the complex housing labour should be lower than that for standard housing. Second, we wanted to take advantage of an additional added benefit of the complex systems: that by facilitating latrine-only changes, they produce a lower impact on the mice.

During full change, mice were handled and given treats before being transferred into a clean triad with new bedding and new nesting materials (Bed-r′Nest® and Nestlets™) provided in each of the three cages making up the triad. About one third of the old nest was scattered across the three cages. The position of the food and water cage (middle vs. end) was changed at this time. The position of these cages was changed to test the relative importance of cage function (e.g., ‘food and water cage’ or ‘neutral cage’) versus position (e.g., at the end with one entry or in the middle with two entries) when establishing nesting and elimination sites relative to environmental features. The decision was made not to move the red cage, as it was anticipated that mice would use this cage for nesting and we did not want to introduce too much disruption. During latrine-only change, all mice were scooped up and given treats, but only the dirtiest cage was replaced with a clean cage with new bedding. No new nesting materials were provided; instead, the existing nest was left intact. The position of the food and water cage was not changed.

### Data collection

#### Nesting and soiling

To investigate the use of space for nesting and soiling in each housing system, the location of urine spots (identified as wet, clumped or discoloured bedding), the location of the nest, and nest quality (scored on a scale from 2: flat to 5: full dome^41^; ‘no nest’ was scored as 1) were recorded once per week for 15 weeks by direct observation during cage changing, starting three weeks after mice were placed in their respective housing systems. We obtained 12–14 observations/cage, for a total of 63 observations of standard and 64 observations of complex for analyses. Some data were lost due to human error (e.g., record missing from the record sheet), but losses were evenly distributed across weeks and cages. Observations were made once the cage was removed from the rack and mice removed from the cage. Each system was subdivided into three locations. In the standard system, locations were: front-right (near the water bottle and part of the feeder), front-left (this was the most open space in the cage), or back (semi-open space behind and under part of the feeder at the back of the cage; Fig. 1a). In the complex system, locations were: food and water cage, neutral cage, or red cage.

During cage changing, an investigator took one overhead-view and one bottom-view photograph (Samsung Galaxy Tab Pro SM-T520, Samsung Electronics Co., Ltd., Vietnam) of each cage after the mice and any nesting material, feeder and water bottle were removed. These photographs were later arranged into pairs (i.e., overhead- and bottom-view of each cage were placed next to each other and saved as one image), all identifying labels were removed, and their order was randomised (see Supplementary Information for these photos). Two independent observers, blind to date and cage position (except in the case of red cages, which were only in the complex system, never in the middle of the triad and never containing the food and water) scored each pair of photographs for soiling level and bedding coverage. Therefore, each standard housing unit (each cage) had one soiling and one bedding coverage score, while each complex housing unit (each triad) had three soiling and three bedding coverage scores (one for each cage in the triad). Observers could not be blinded to treatment because cages from the standard housing system did not have tunnel connector ports. However, no statistical comparisons were made between the two treatments on soiling level or bedding coverage (these were within system analyses).

Soiling level, which was used to estimate the amount of faecal pellets in the cage, was scored on a scale from 0 to 3, where 0 = clean or virtually clean; 1 = a little dirty; 2 = fairly dirty; and 3 = very dirty. Bedding coverage, which estimated the amount of bedding left in the cage, was also scored on a scale from 0 to 3, where 0 = less than a third of the floor covered with bedding; 1 = between one and two thirds of the floor covered; 2 = between two thirds and nearly all the floor covered; and 3 = the entire floor covered (bedding has depth). Each pair of photographs was scored by two independent observers.

### Behaviours

To investigate the use of space for various behaviours in each system, mice were observed over a three-week period via instantaneous scan sampling starting three weeks after they were placed in their respective housing systems. Approximately 10 to 15 min after entering the animal room (giving mice enough time to settle down), the observer approached each cage to within approximately 15 cm and recorded the behaviour of each mouse selected for behavioural observation. This included all mice in the standard system (*n* = 15) and three mice from each triad in the complex system (*n* = 15; randomly-selected at the beginning of the study and observed for the remainder of data collection). On 12 different days, the behaviour of each of these mice was instantaneously scan-sampled three times (always at least 2.5 hours before or after light change), producing an overall total of 1071 behavioural scans for analysis (we dropped 9 scans from one session for one of the complex systems because the water bottle had flooded the cage). If the mouse appeared to be influenced by the observer (e.g., sniffing in the direction of the observer or running away from the observer), the observer waited 15 s before resuming behaviour recording. To score behaviours, an ethogram based on^46,48,49^ was adapted to include behaviours particular to this study (e.g., move bedding; Table 1).

### Statistical analysis

We used generalised linear multilevel models (GLMMs), which allow for normal and non-normally distributed outcomes and crossed-random effects^50,51^, to account for both repeated observations by cage-system, session, and mouse. In general, we allowed all parameters (intercepts and slopes) to vary by cage-system, session, and mouse when we had sufficient observations for the model to run (i.e., multiple observations at that level). All models contained at least a random intercept by cage-system. Within the complex system, neither the location of the food/water cage (middle vs. end) nor the cage-change type (full vs. latrine-only) modulated any of the effects or associations between the variables considered and were thus not included in any of the reported models. All models were univariate (i.e., evaluated a single outcome), contained only one fixed-effect/independent variable (as indicated in the Results, unless otherwise noted, i.e., a covariate), and were checked to make sure they met model assumptions of a linear relationship between the outcome and the independent variable(s). The only time we found indication of a non-linear relationship was the effect of days since cage-change on the probability of affiliative behaviour, which appeared to follow a curvilinear path. For this behaviour only, therefore, we tested the quadratic (curvilinear) effect of days since cage-change on probability of affiliative behaviour.

Presence/absence outcomes (urine spots by location, nests by location, behaviours by scan) were modelled with logistic GLMMs (logit link, binomial error distribution) and evaluated with z-statistics. In accordance with model assumptions, we did not evaluate any presence/absence outcomes that had incidence rates below 5%. To quantify the presence/absence of nests and count the number of nests mice built within each system, we categorised each location within a cage-system as having a nest (score of 3: cup or higher) or not (having a score of 2: flat or lower). For example, if a complex system contained no nesting material (score of 1) in the food and water cage, flat nesting material (score of 2) in the middle cage, and high domed nest (score of 4) in the red cage, the system would receive a tally of 1 nest.

Bedding coverage was modelled with a normal GLMM (identity link, Gaussian error distribution) and evaluated with t-statistics using Satterthwaite-corrected degrees of freedom. The effect of location type (three types of locations in each system; see Methods) on the probability of finding a urine spot or a nest was assessed with a Likelihood Ratio Test (LRT) model comparison: the null model without the location factor vs. a model including the 3-level location factor. Interobserver reliability scores for soiling and bedding coverage were calculated using Spearman’s rank-order correlation. Interobserver reliability scores were ρ > 0.84 for soiling level and ρ > 0.94 for bedding coverage.

All data manipulation, plotting, and statistical analyses were conducted in R^52^, using Rstudio^53^ and the following packages: tidyverse^54^, psych^55^, lme4^56^, lmerTest^57^.

## Supplementary information

Supplementary Dataset 1

Supplementary Dataset 2

Supplementary Dataset 3

Supplementary Dataset 4

## Data Availability

All data generated or analysed during this study are included in this published article (and its Supplementary Information files).
