# Supplementary material for: Standard laboratory housing for mice restricts their ability to segregate space into clean and dirty areas
Source: Sci Rep. 2019 Apr 16;9:6179. doi: 10.1038/s41598-019-42512-3 (PMC6467917; doi:10.1038/s41598-019-42512-3)

Supplementary Information for

Standard laboratory housing for mice restricts their ability to segregate space into clean and dirty areas

I. Joanna Makowska, Becca Franks, Cathy El-Hinn, Tina Jorgensen, Daniel M. Weary

I.J. Makowska: joanna.makowska@.ubc.ca
B. Franks: baccafranks@gmail.com

This PDF file includes:
Photographs used to score soiling level and bedding coverage, legend included

June 2 COMP 1 left

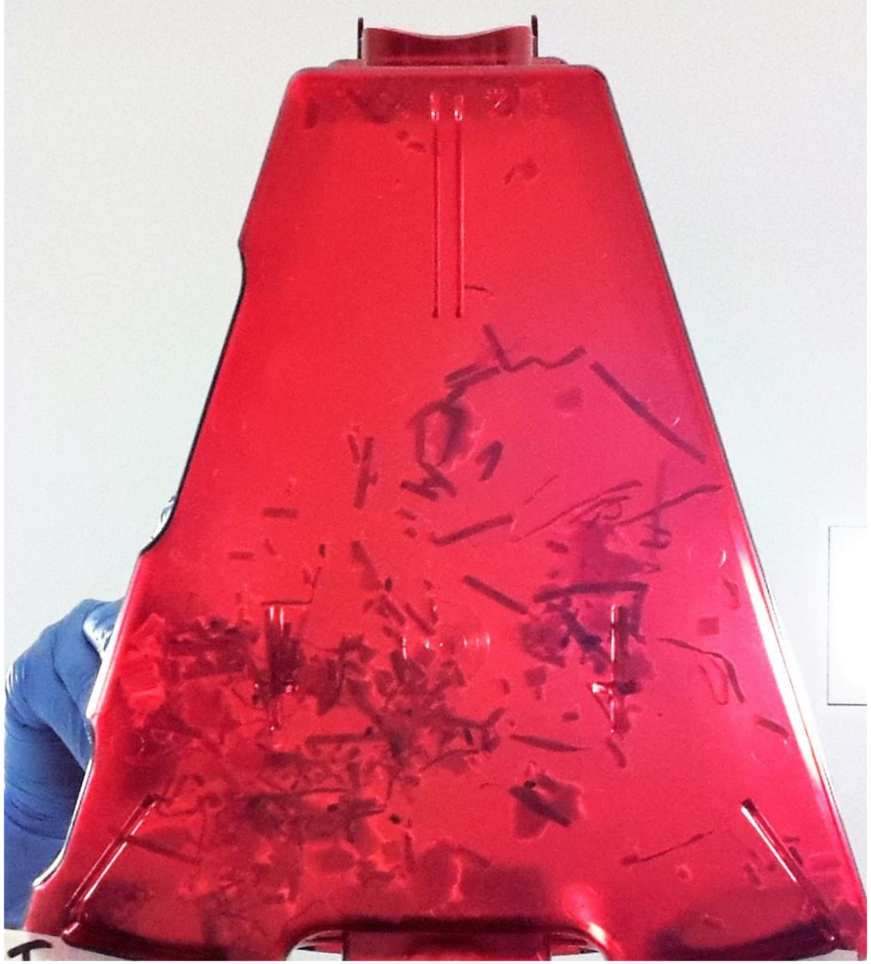

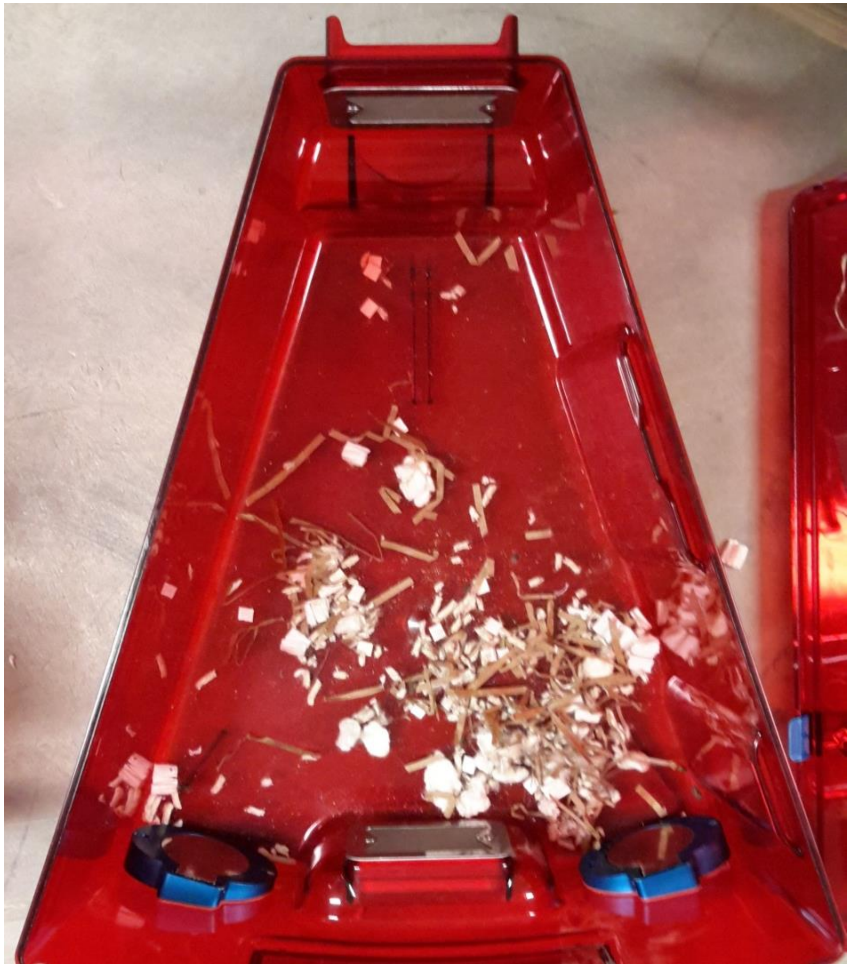

June 2 COMP 1 right

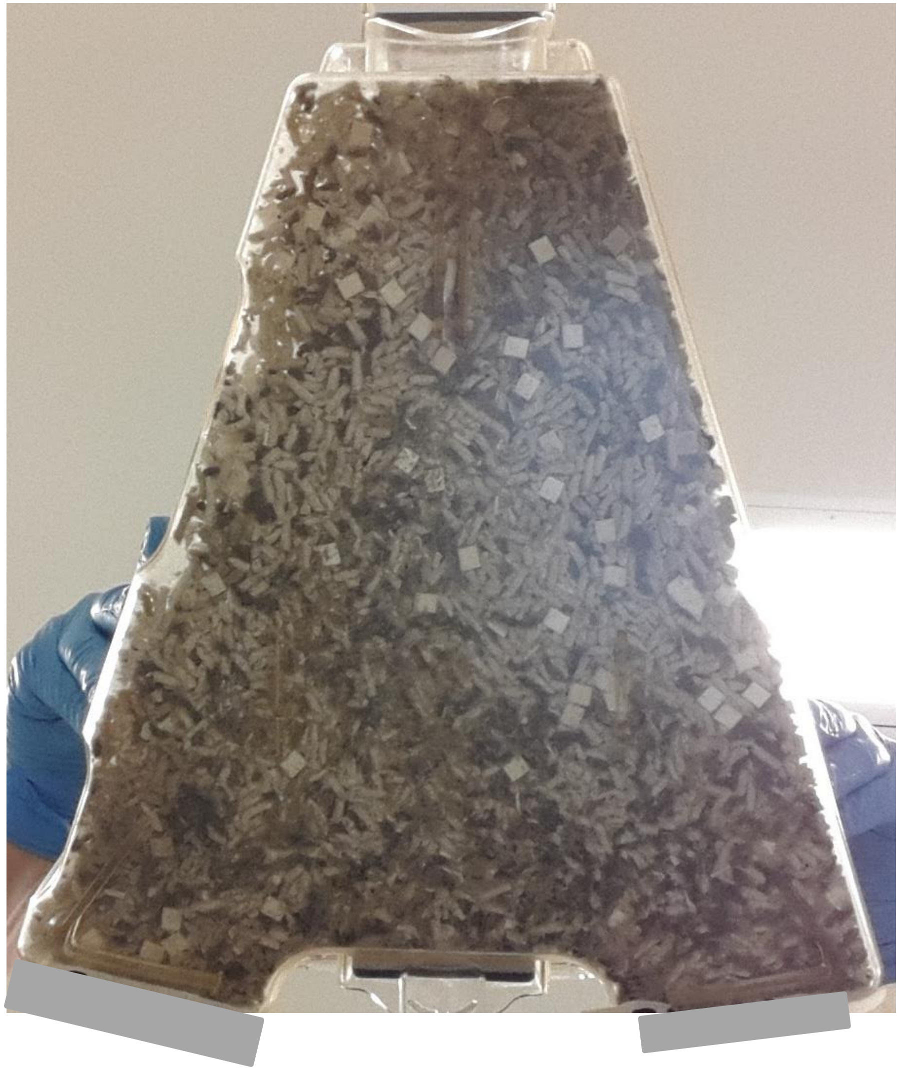

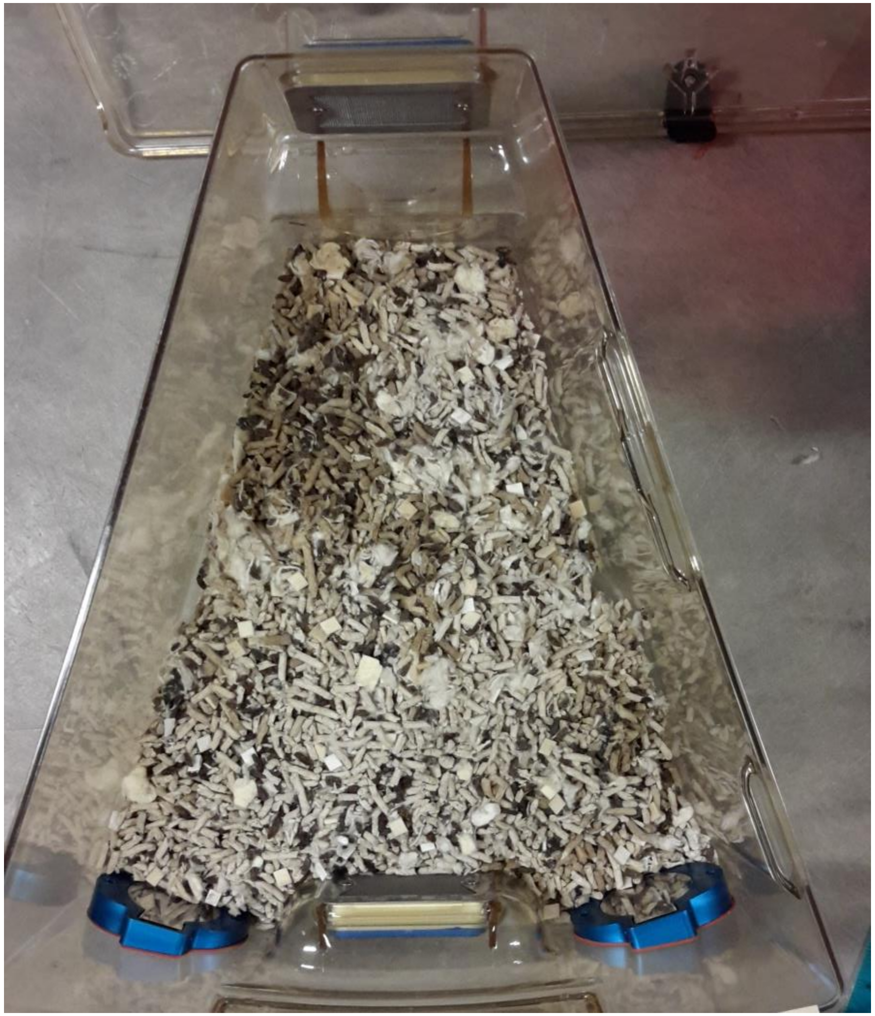

June 2 COMP 1 mid

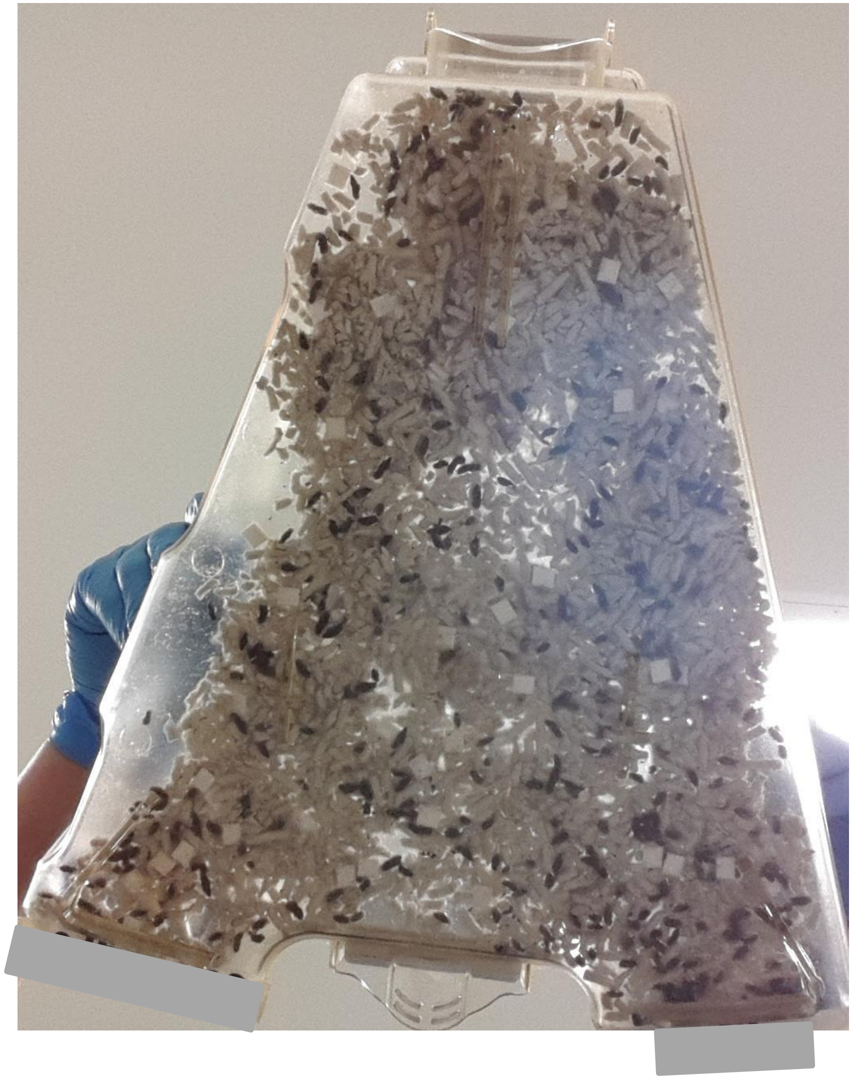

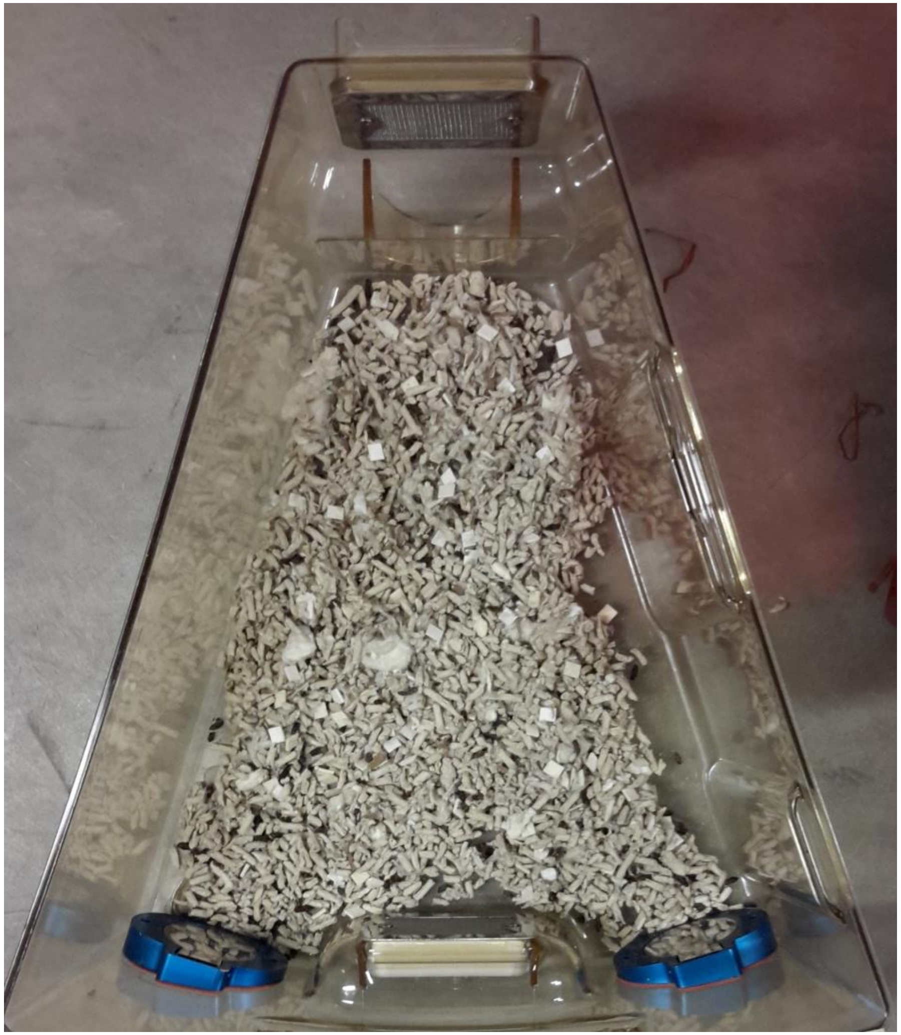

June 2 STD 1

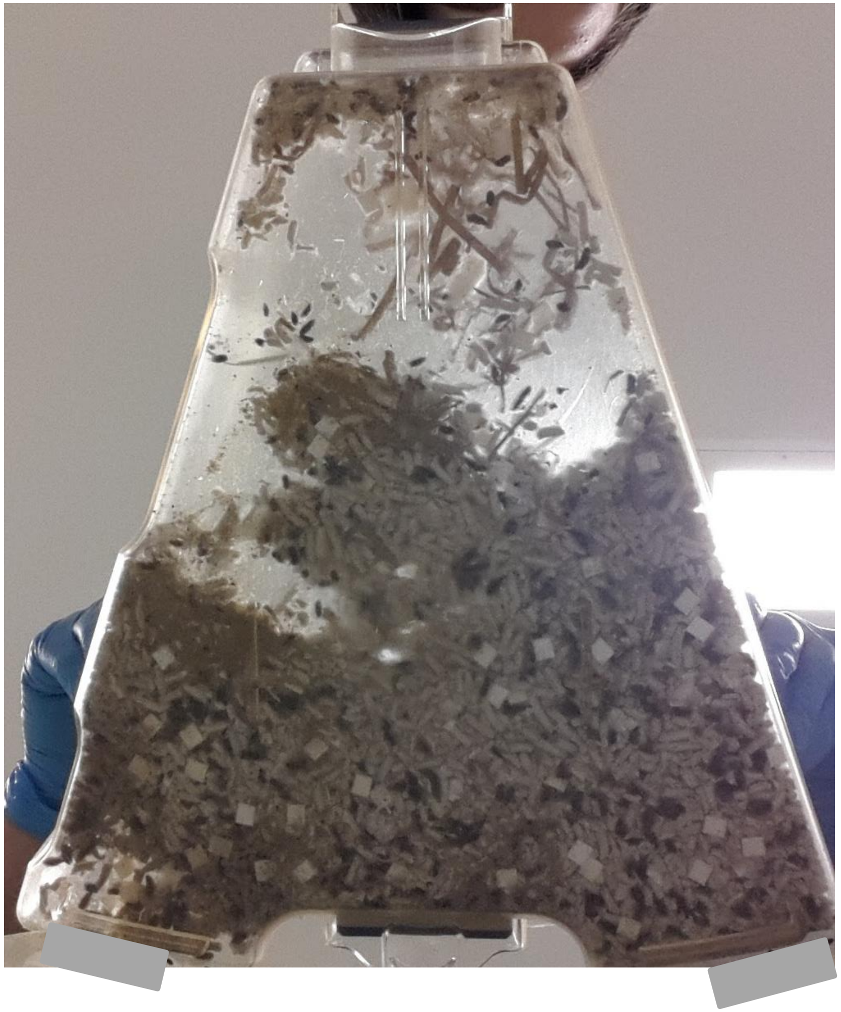

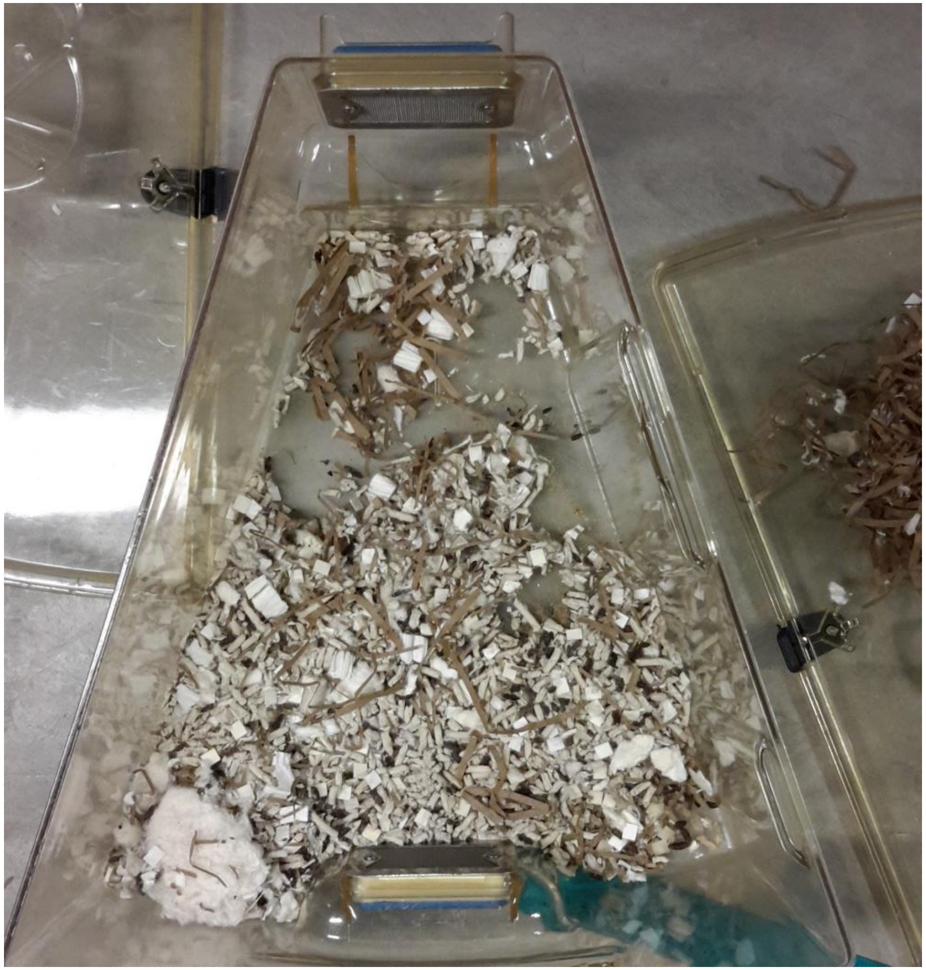

June 2 COMP 2 right

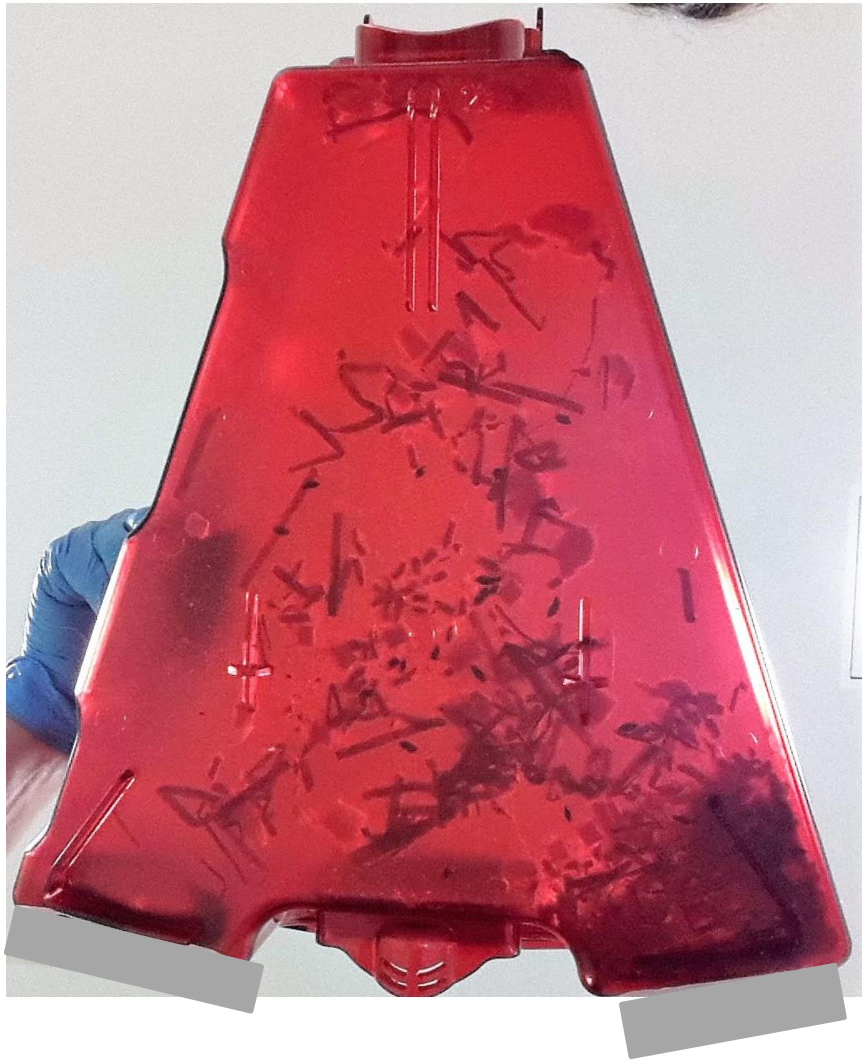

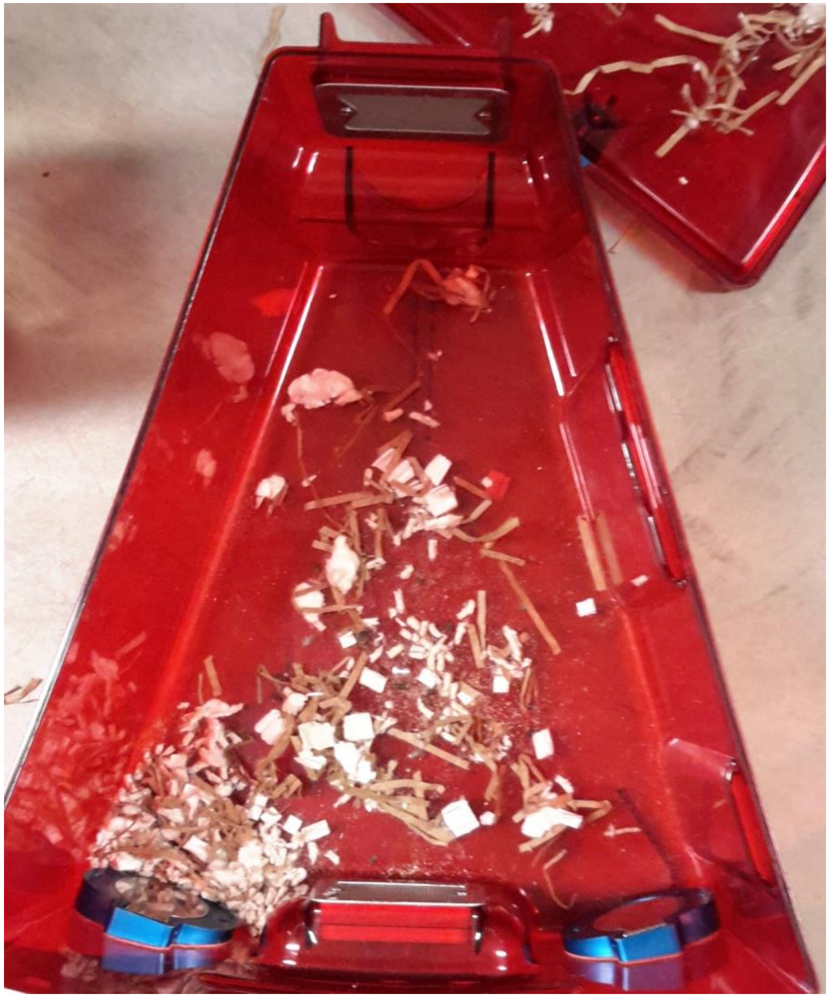

June 2 COMP 2 mid

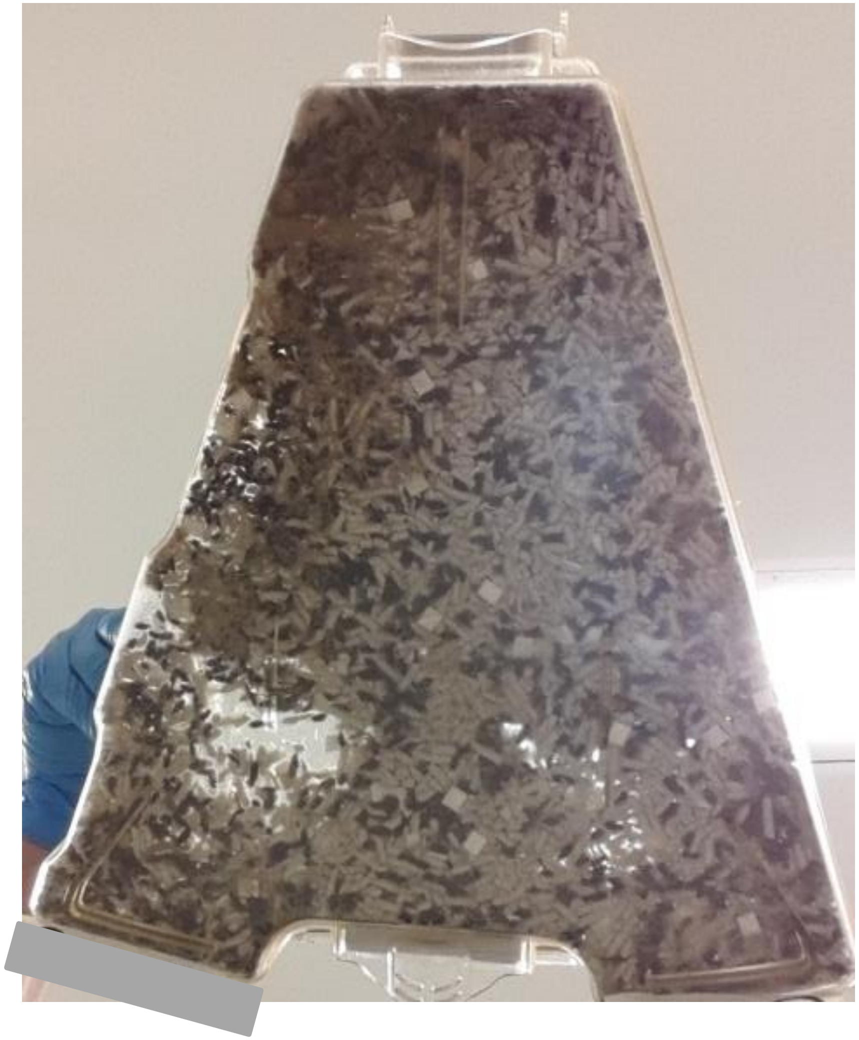

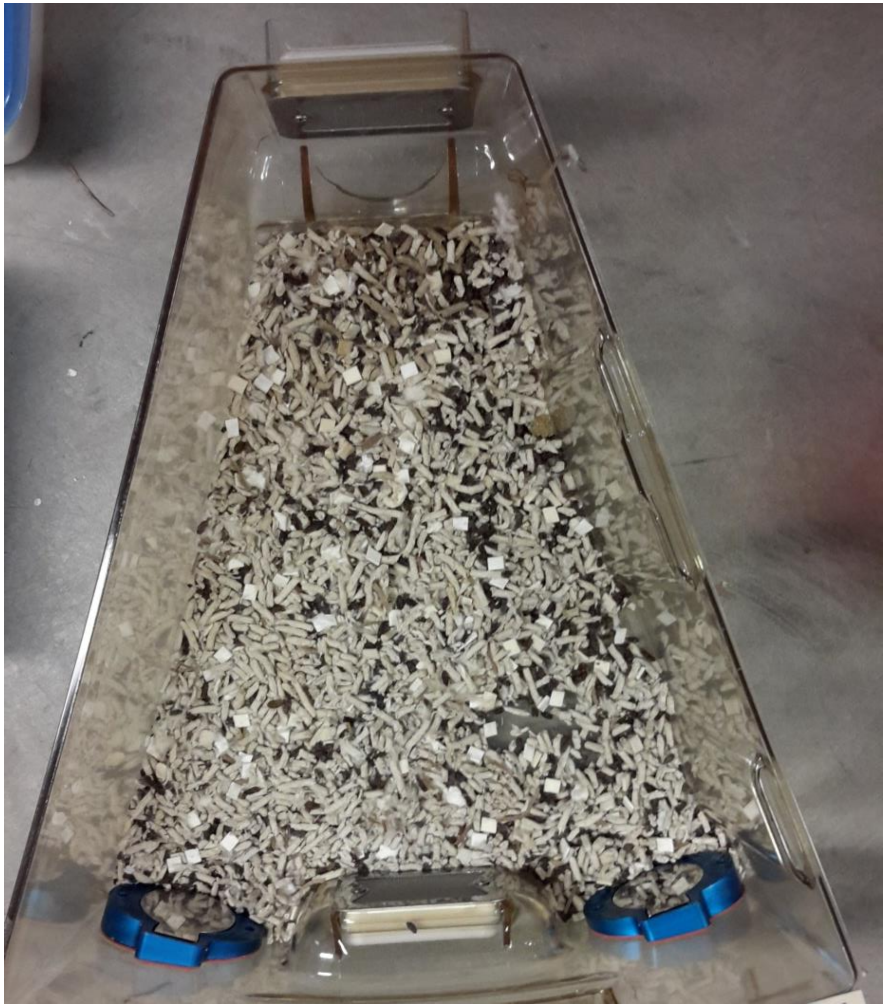

June 2 COMP 2 left

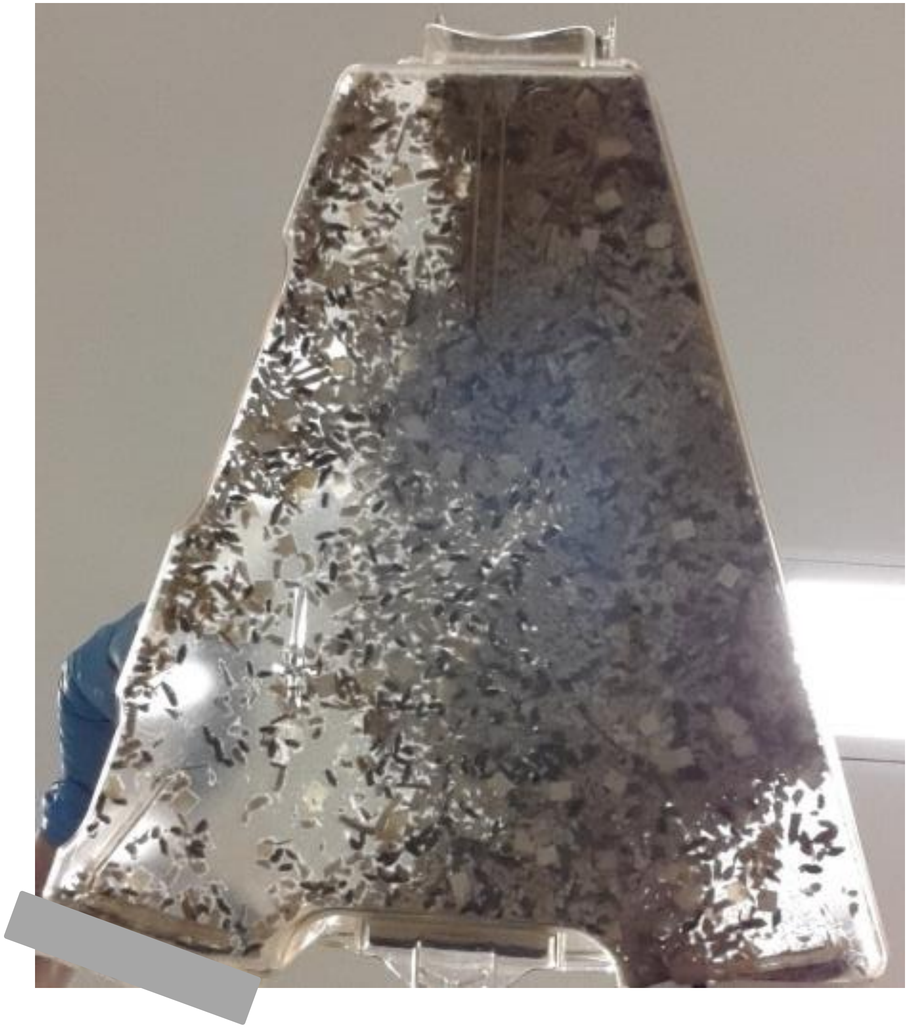

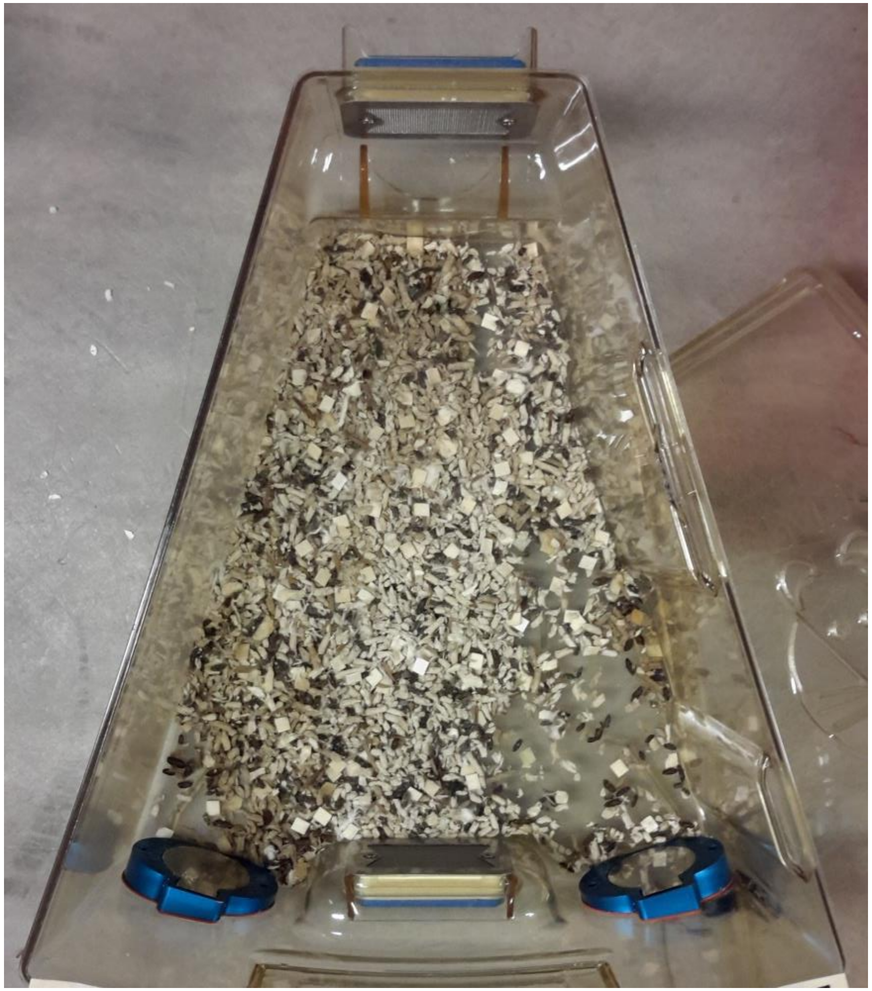

June 2 STD 2

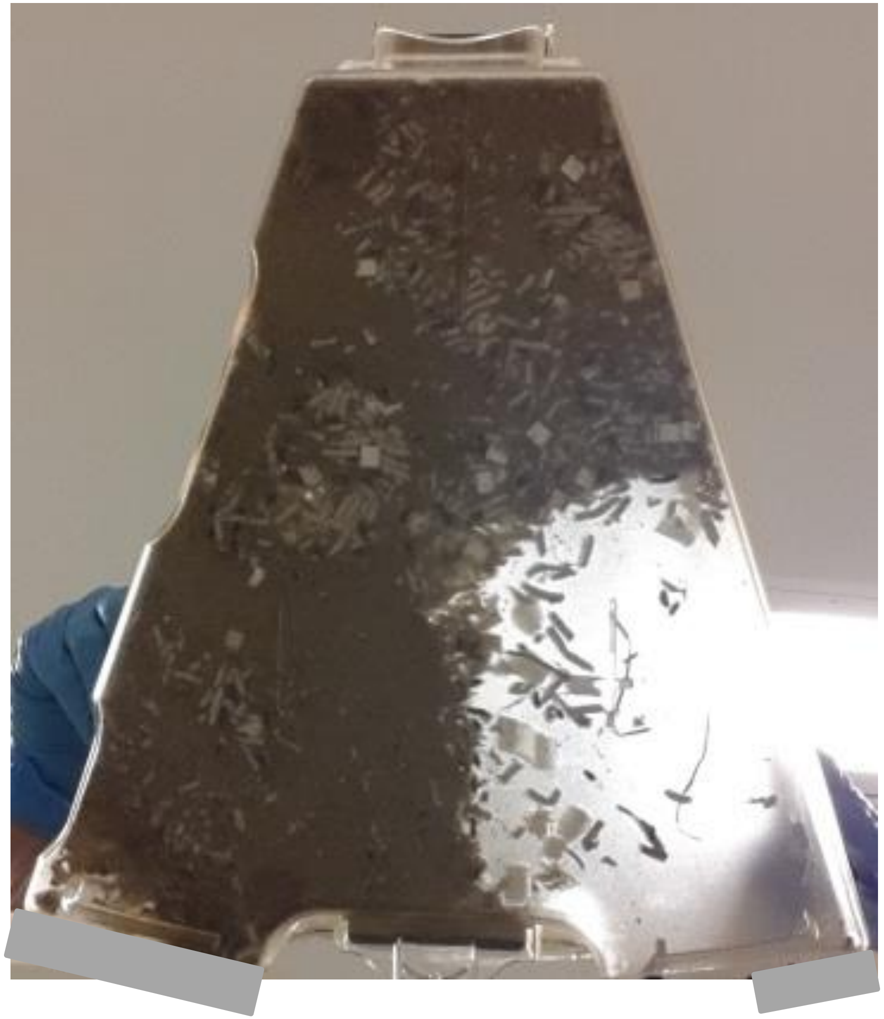

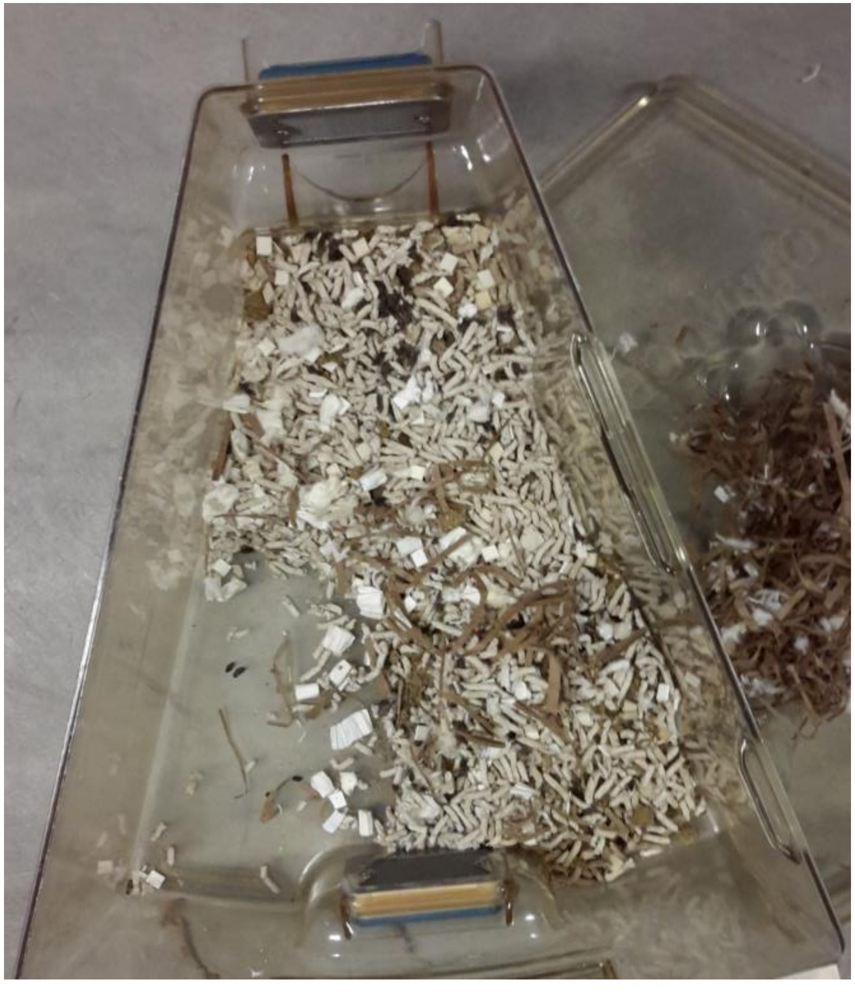

June 3 COMP 3 right

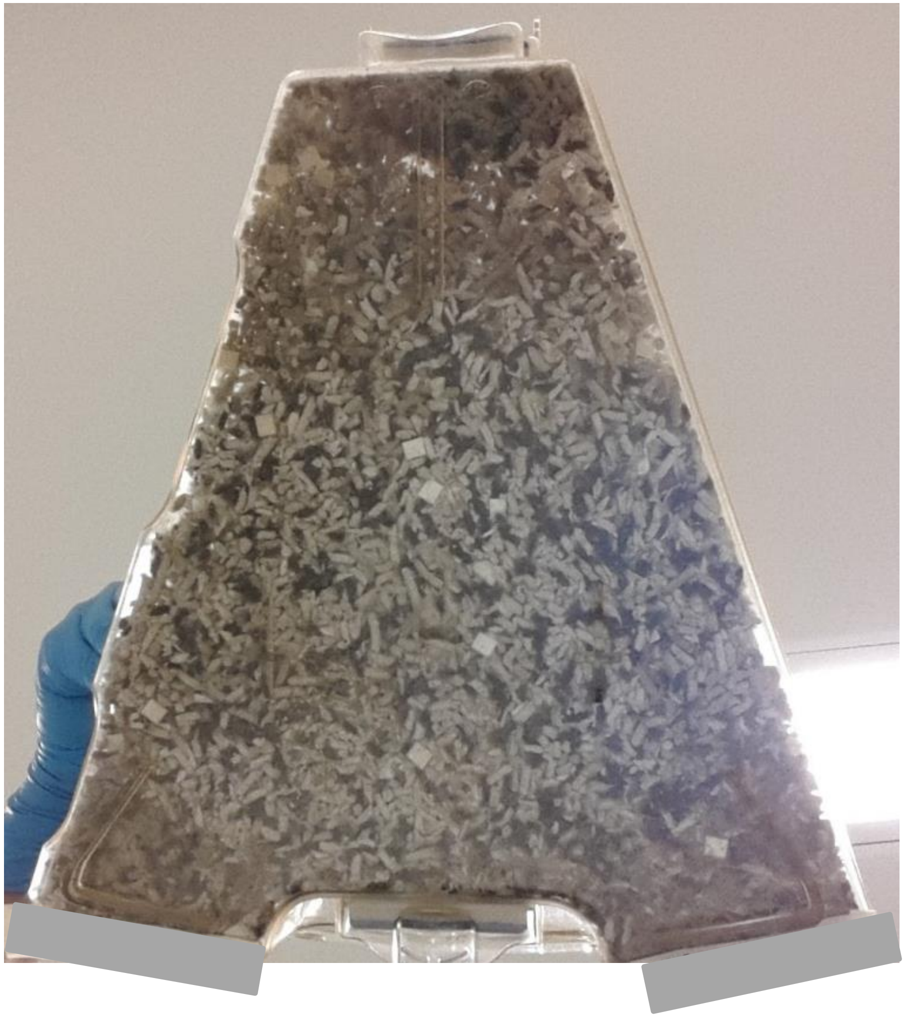

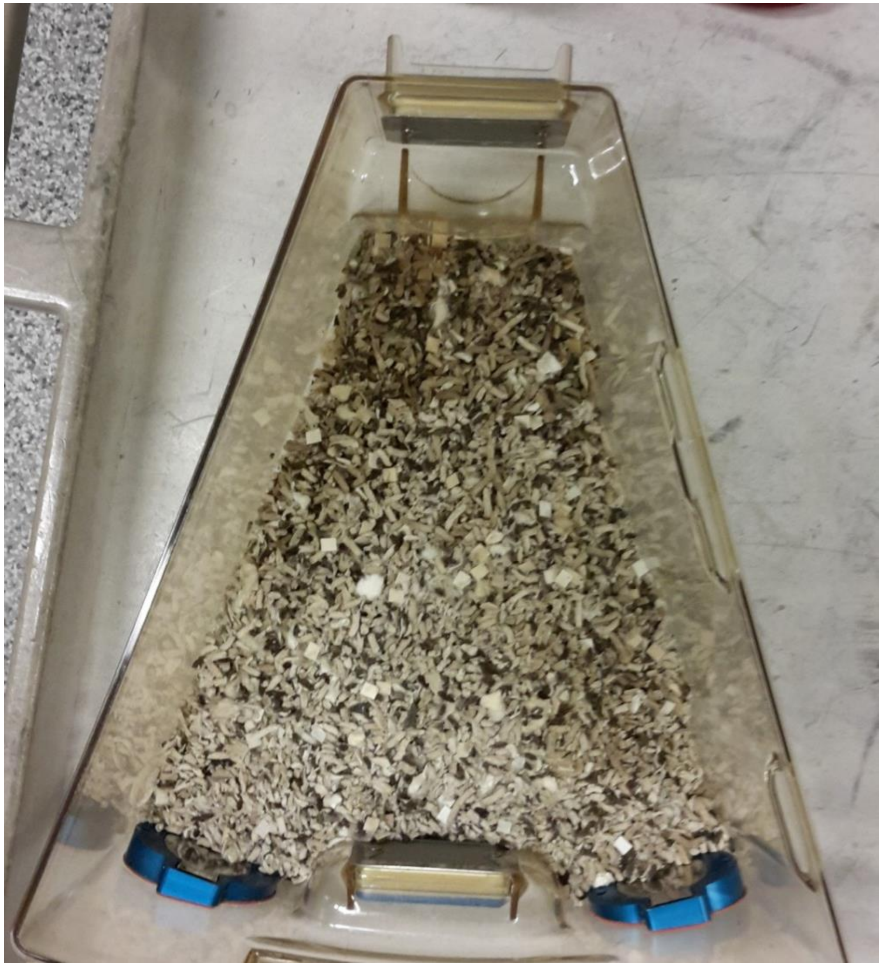

June 3 COMP 3 mid

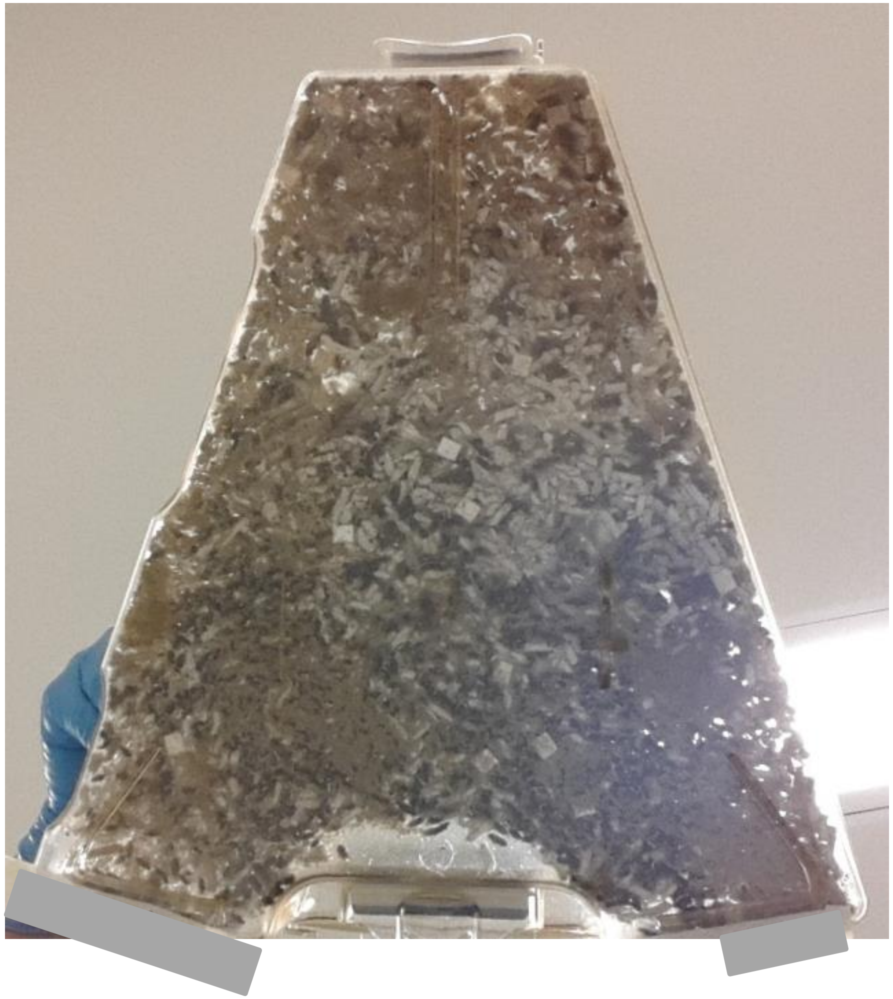

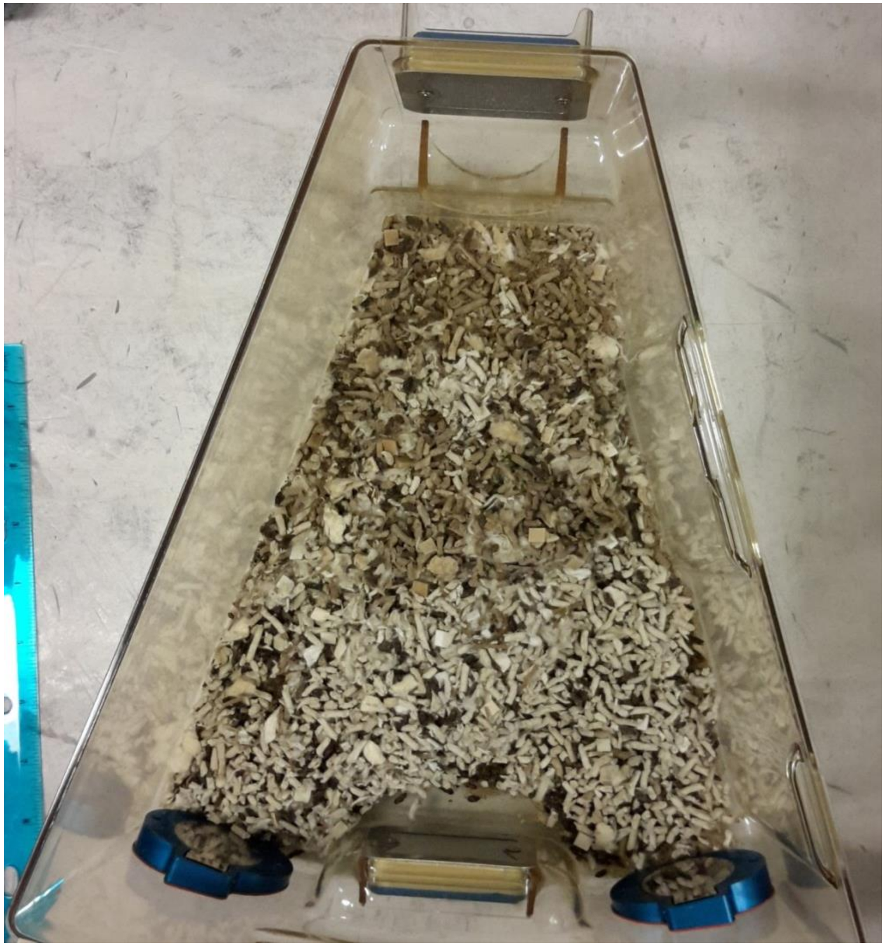

June 3 COMP 3 left

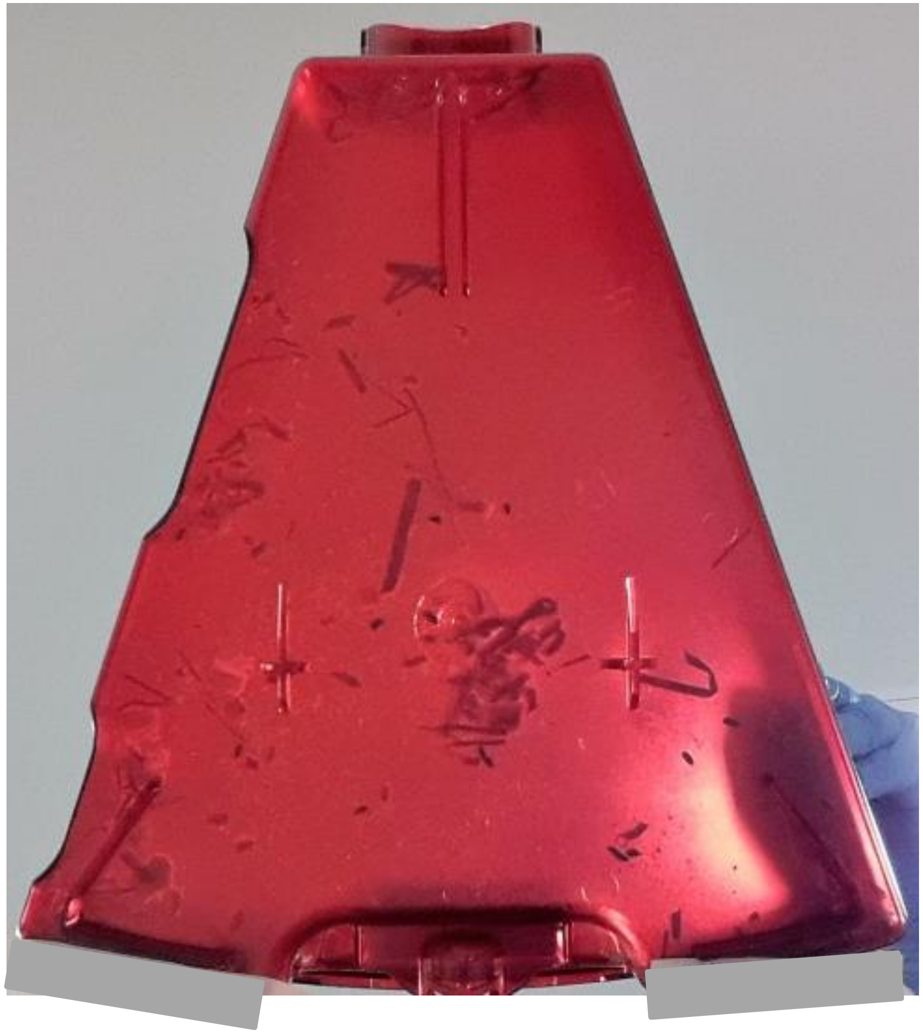

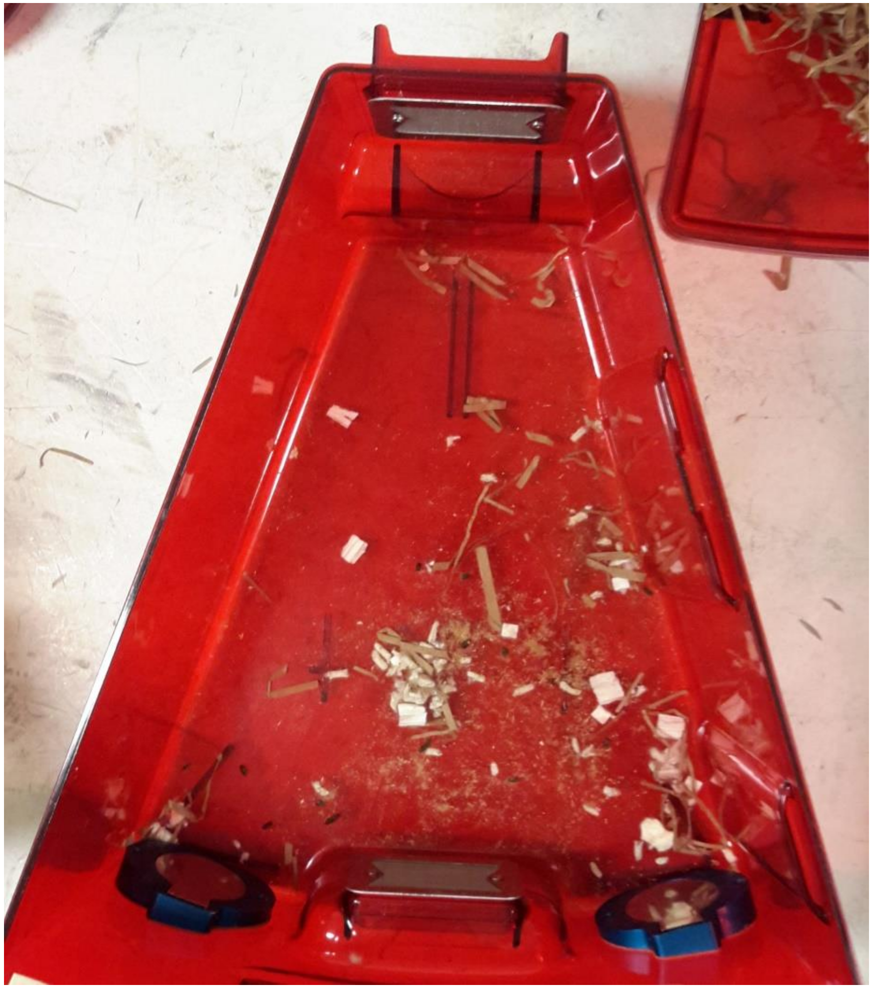

June 3 COMP 3 STD

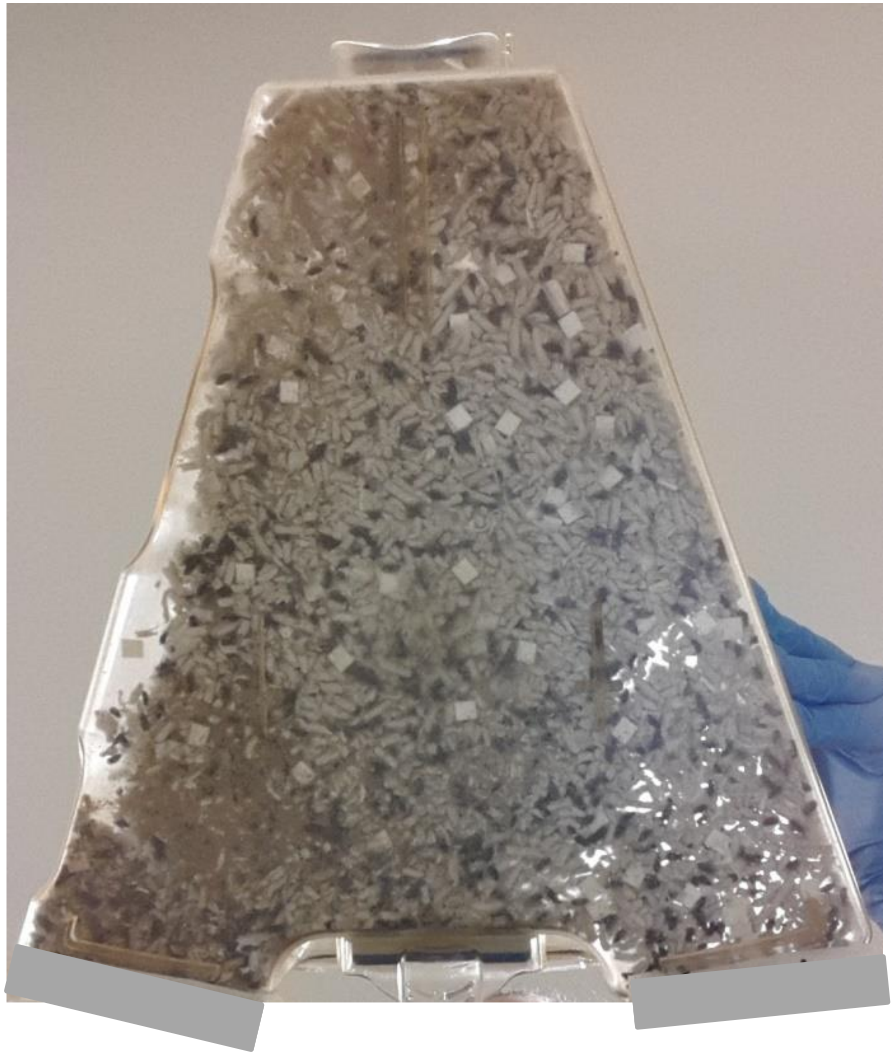

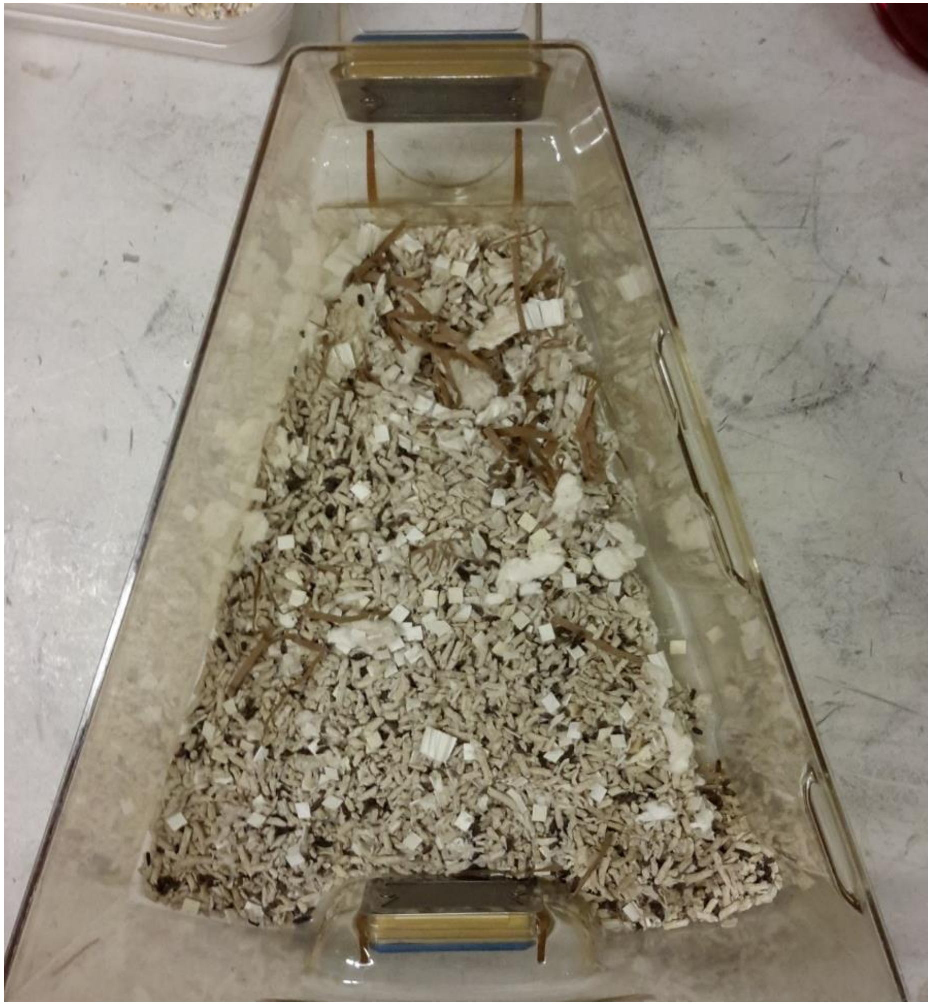

June 3 COMP 4 right

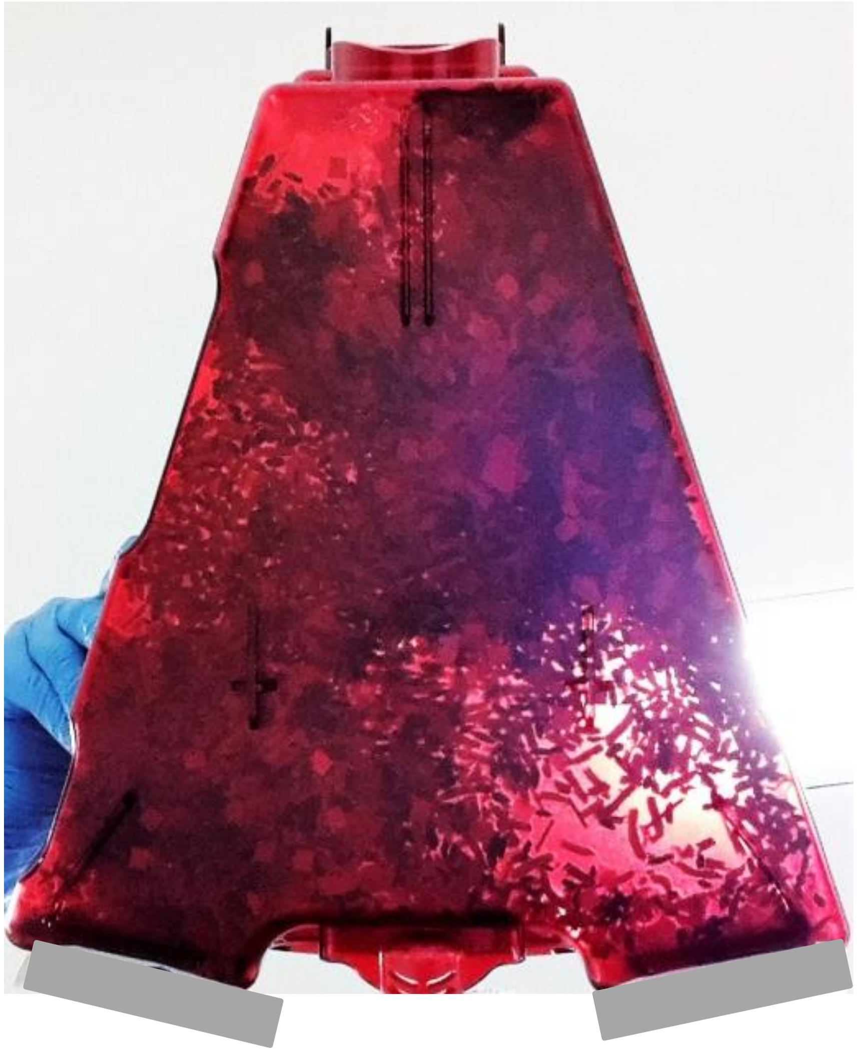

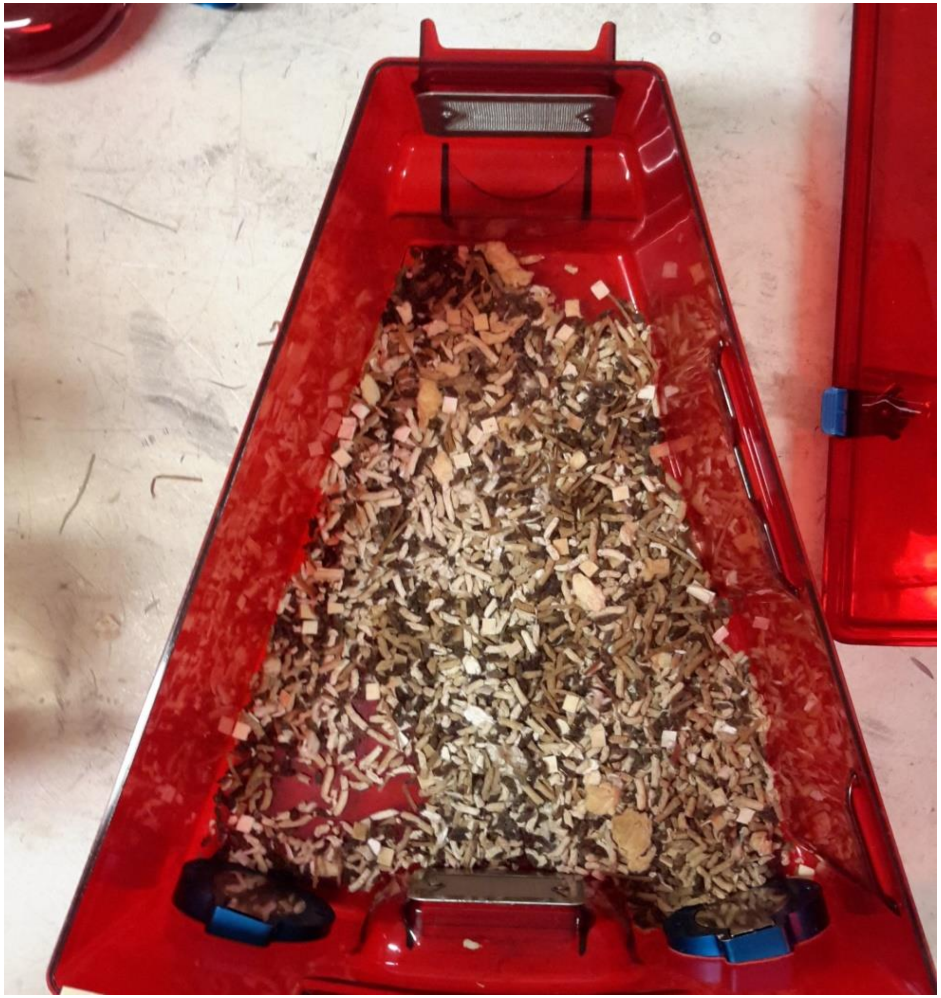

June 3 COMP 4 mid

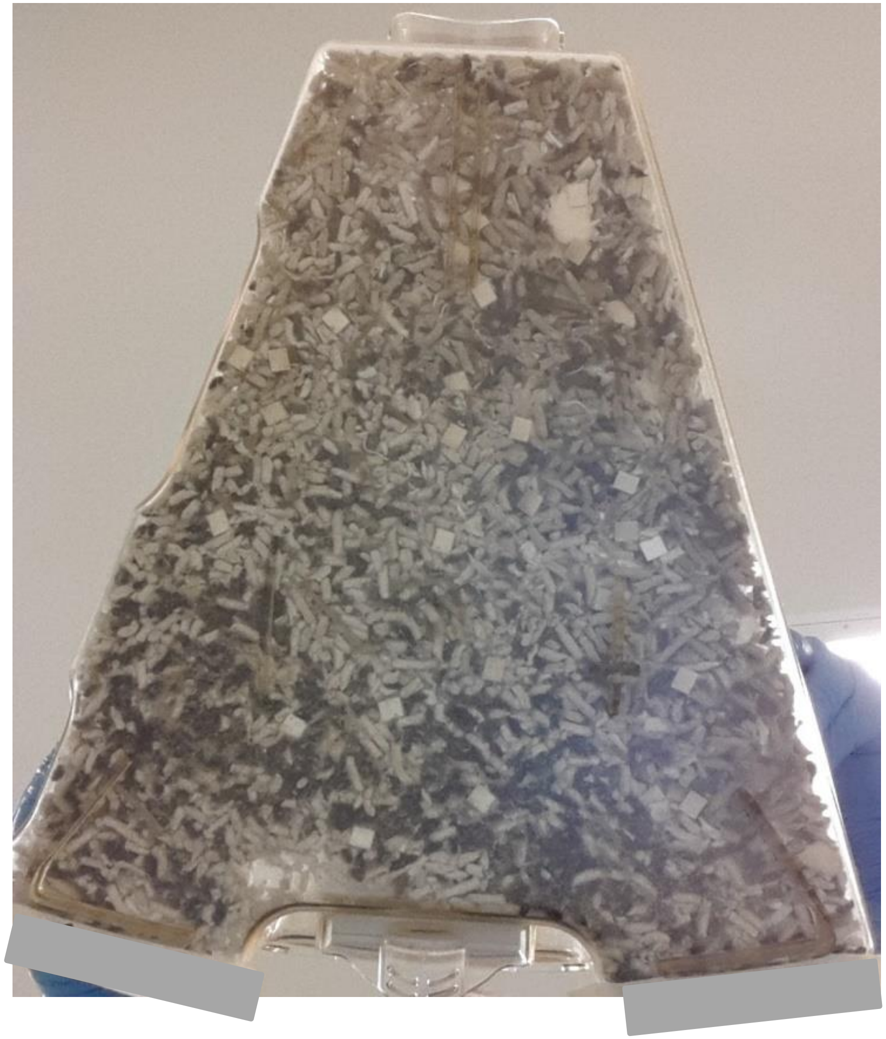

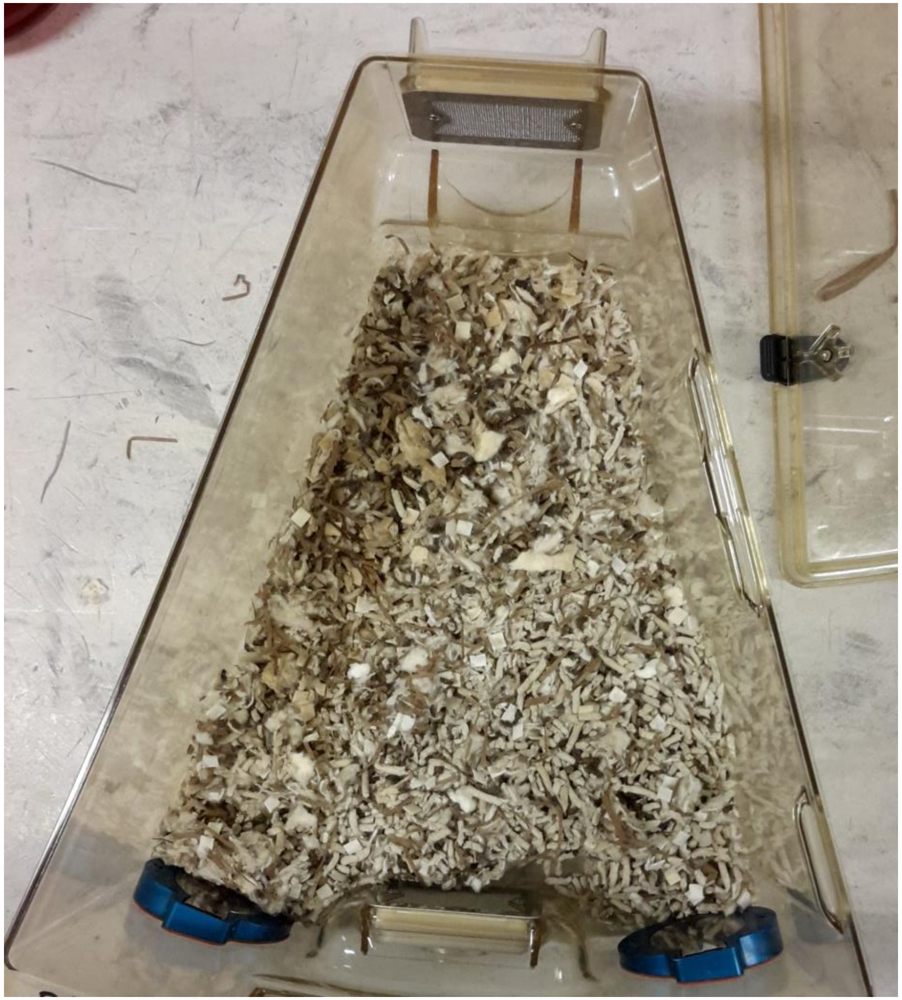

June 3 COMP 4 left

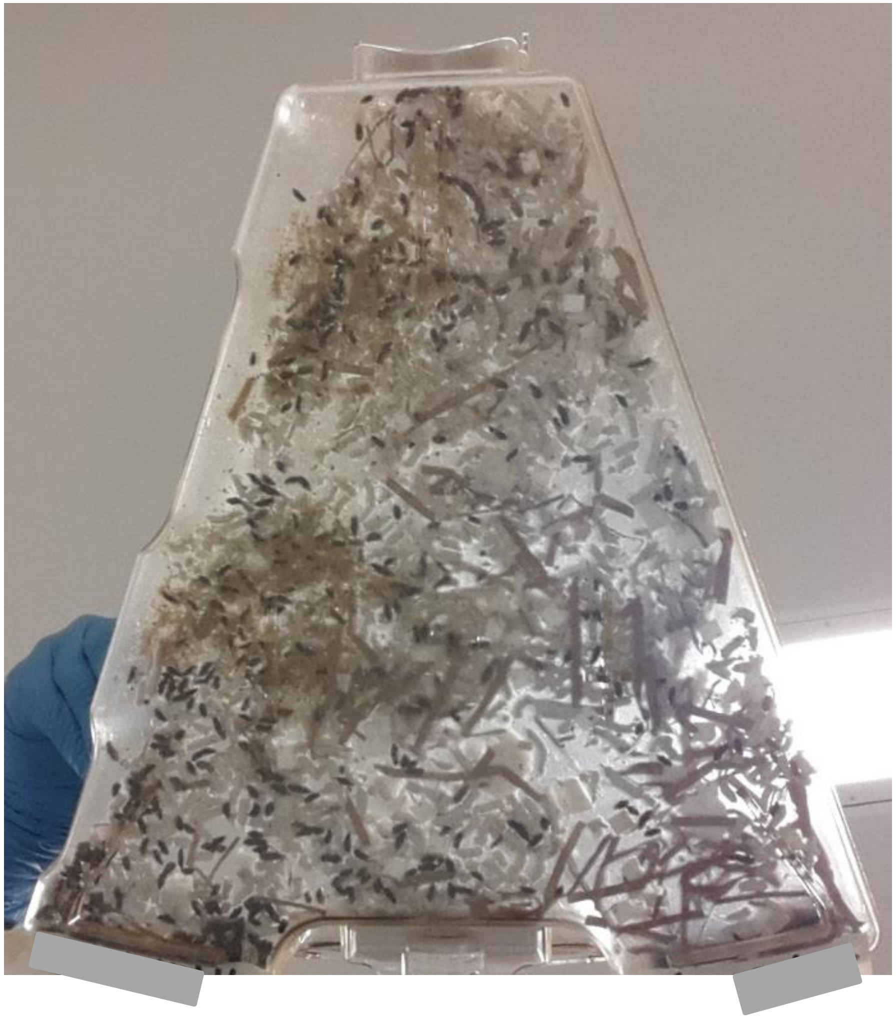

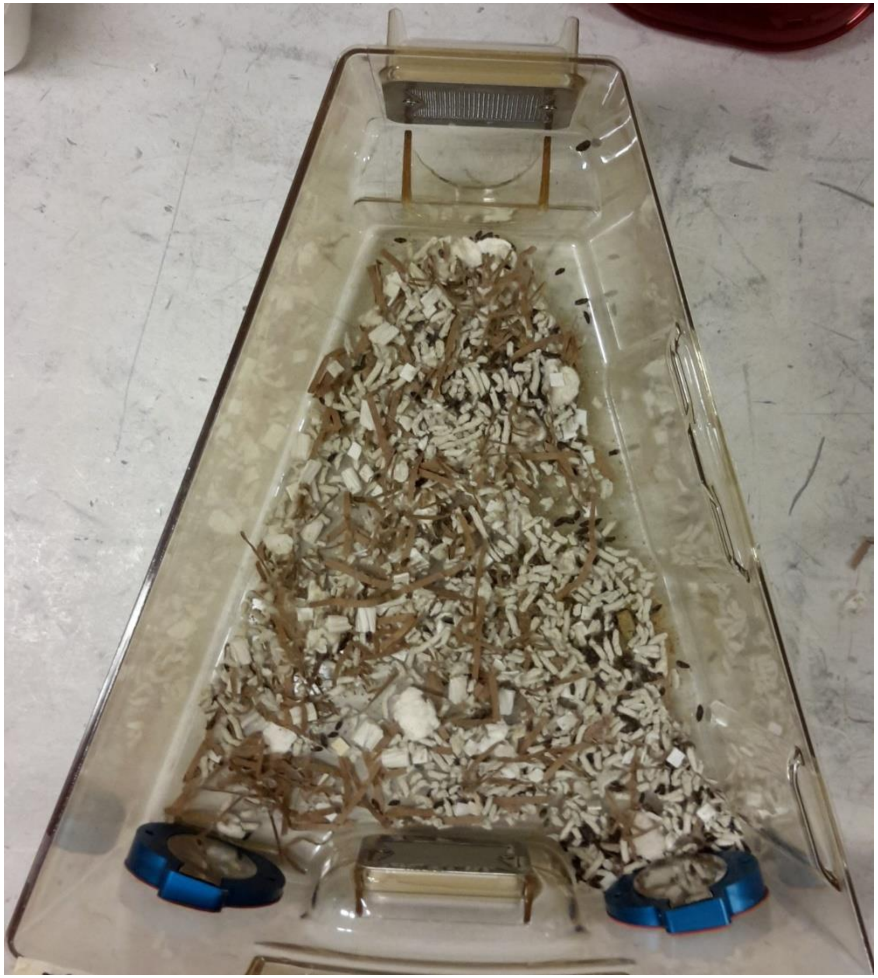

June 3 STD 4

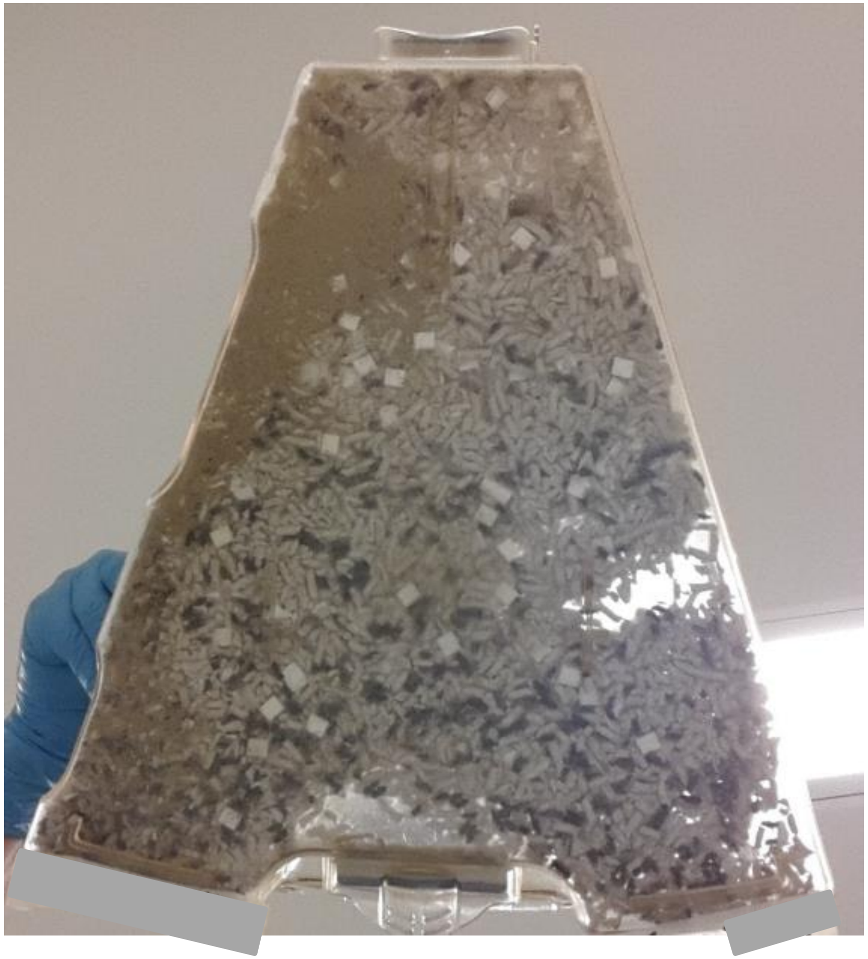

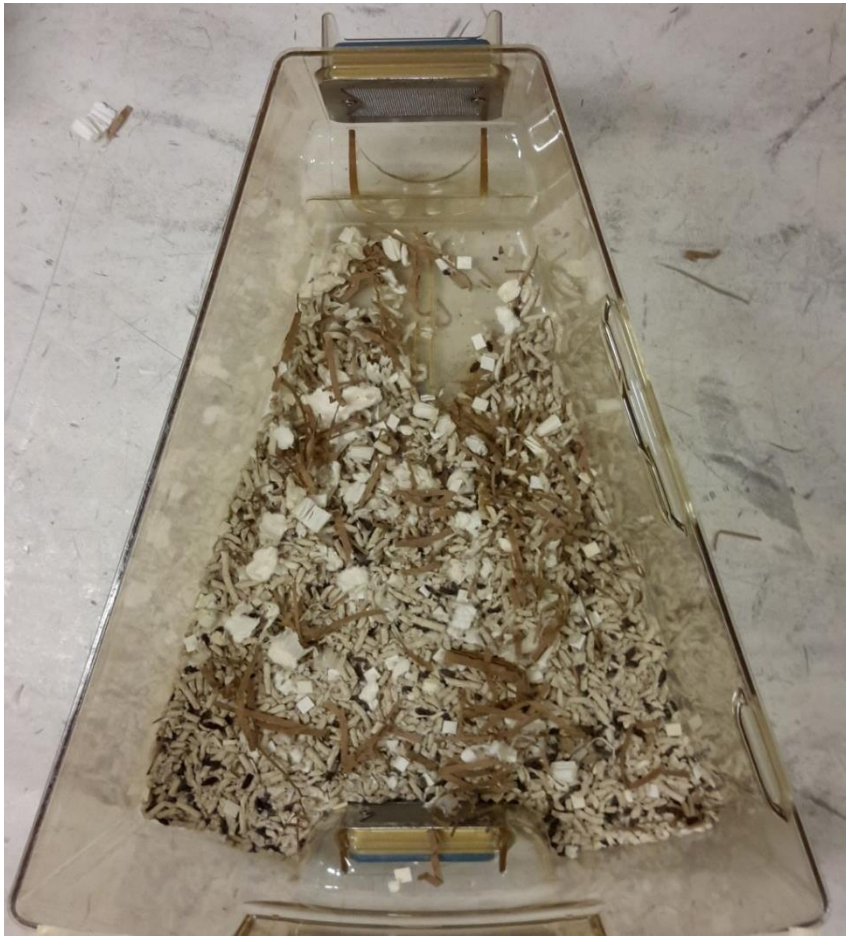

June 4 COMP 5 right

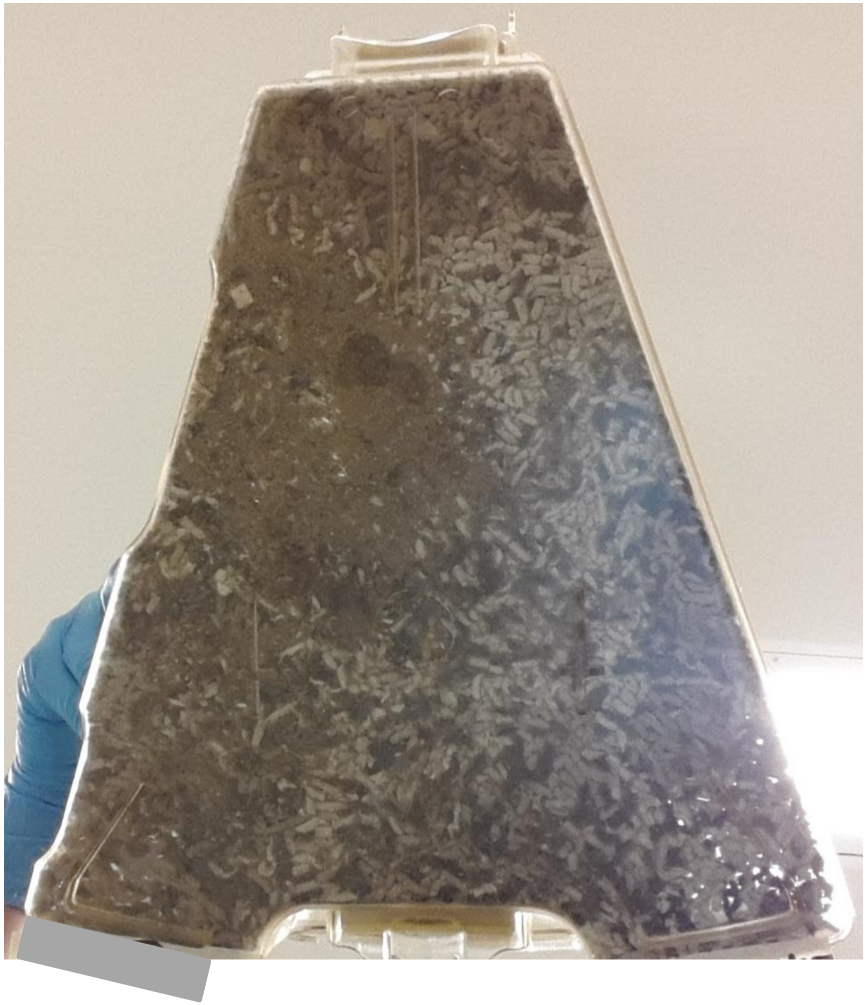

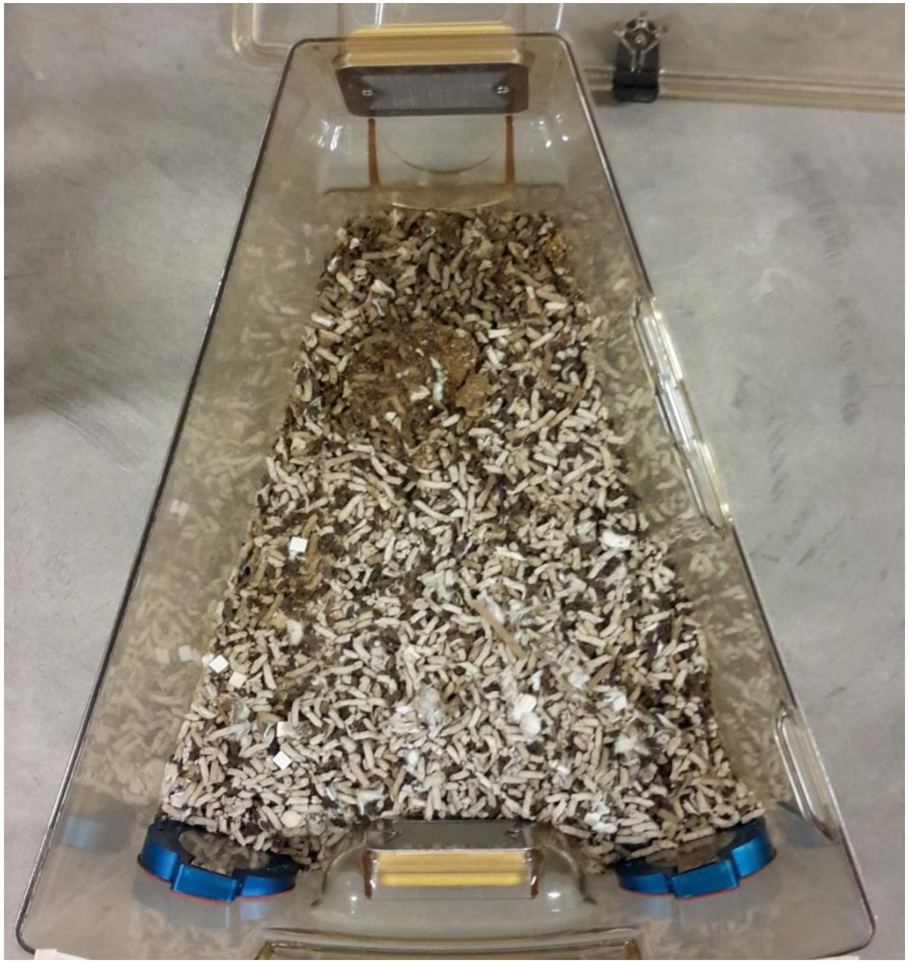

June 4 COMP 5 mid

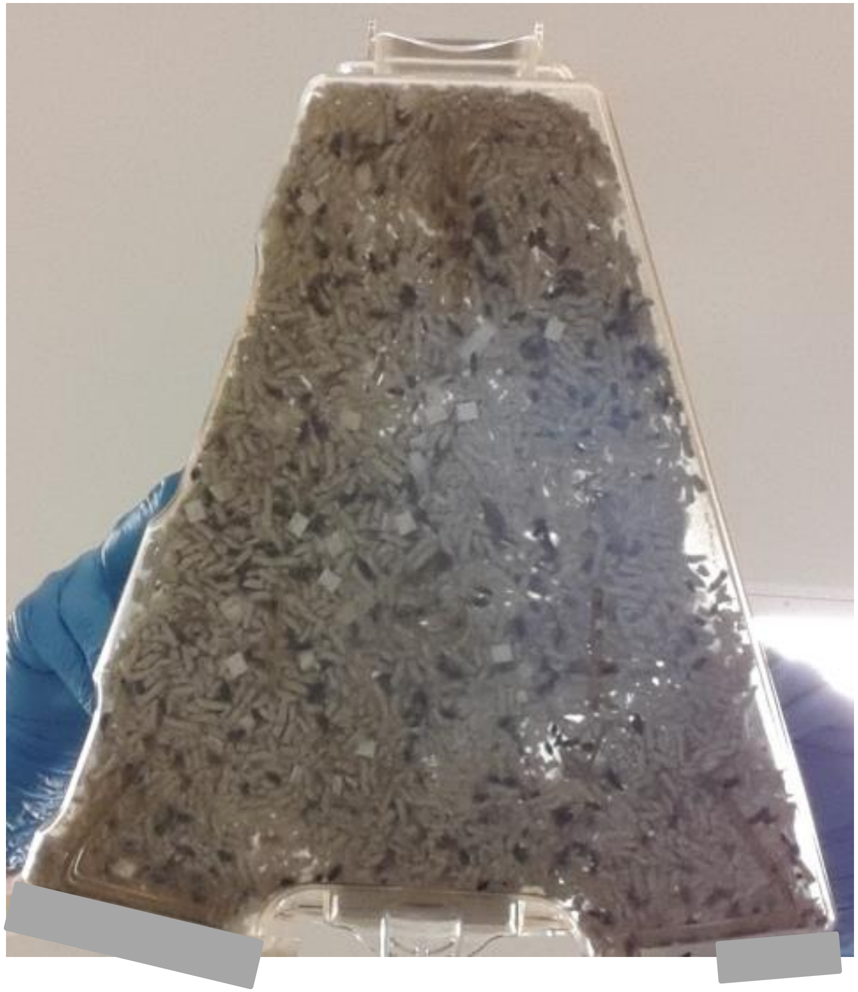

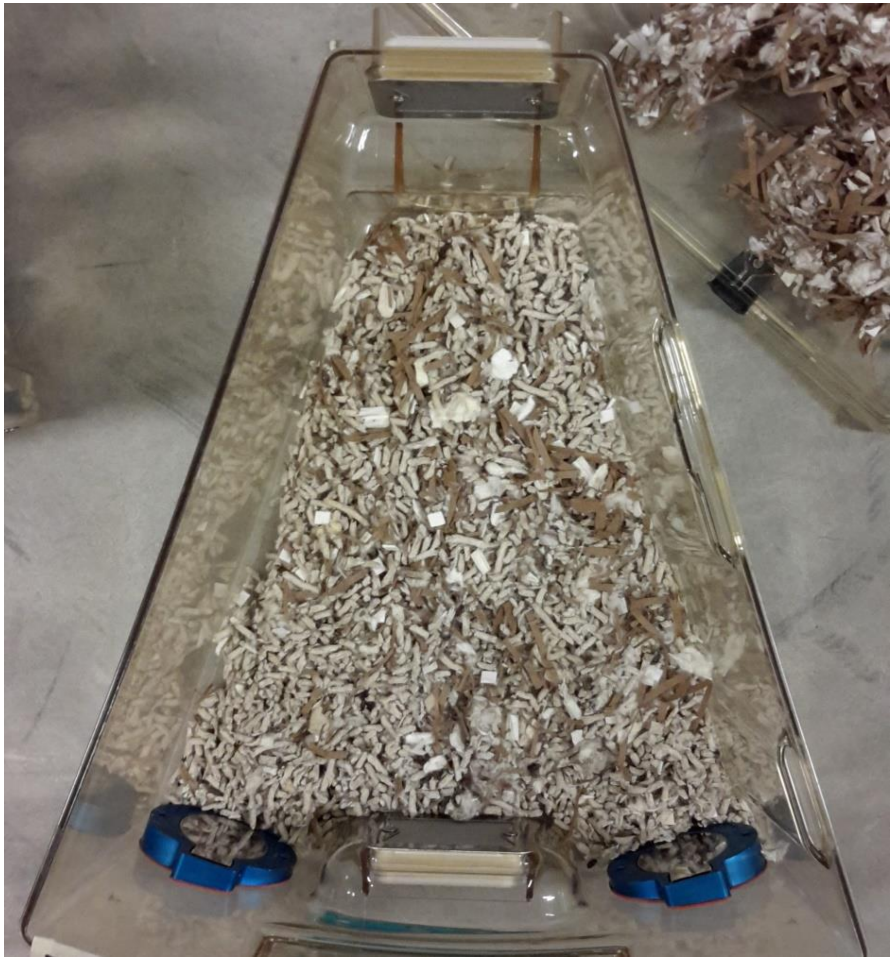

June 4 COMP 5 left

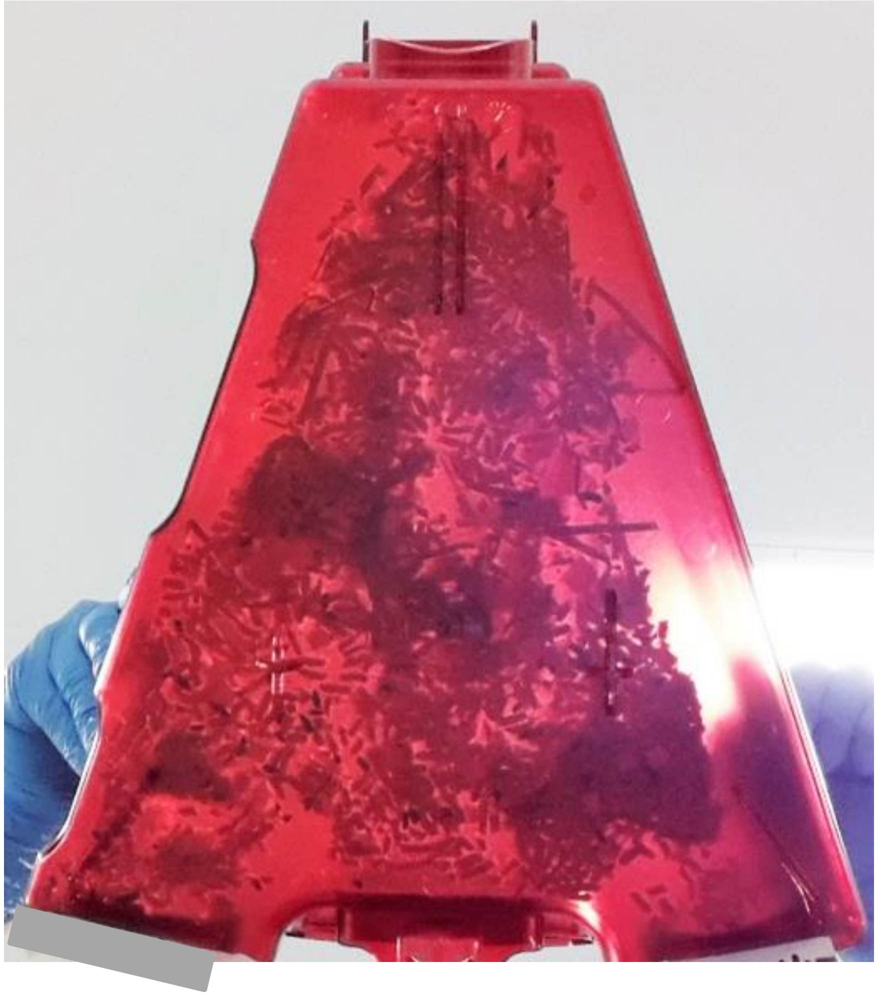

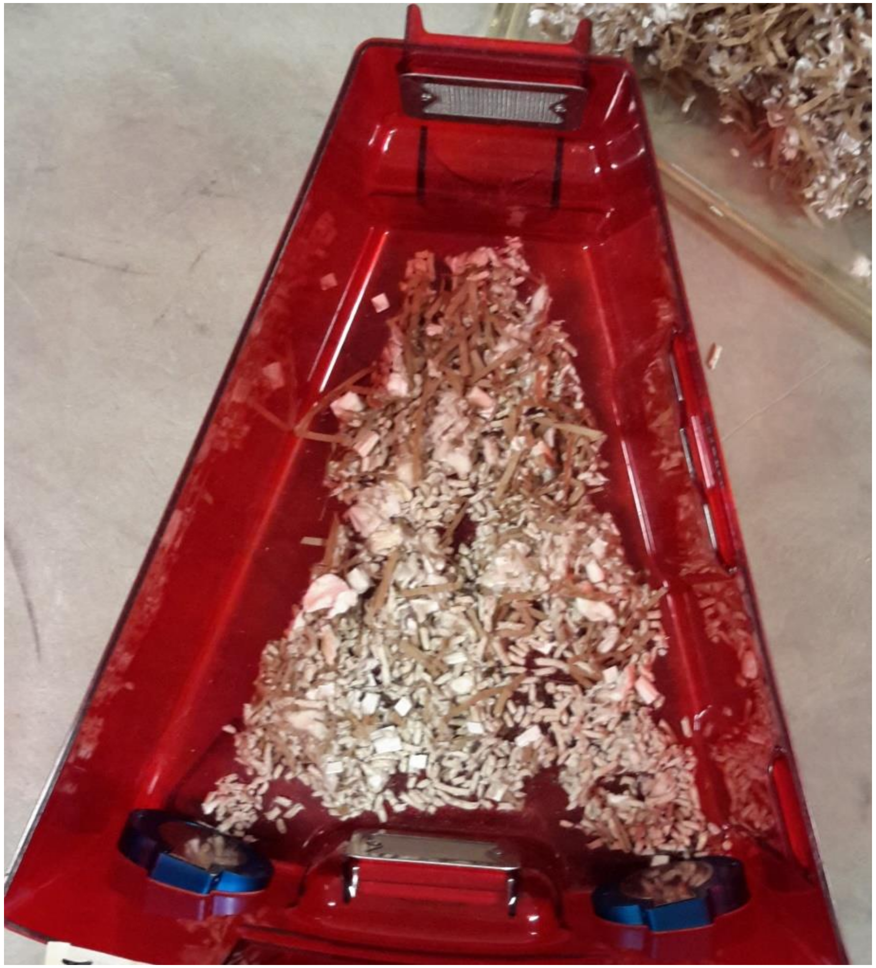

June 4 STD 5

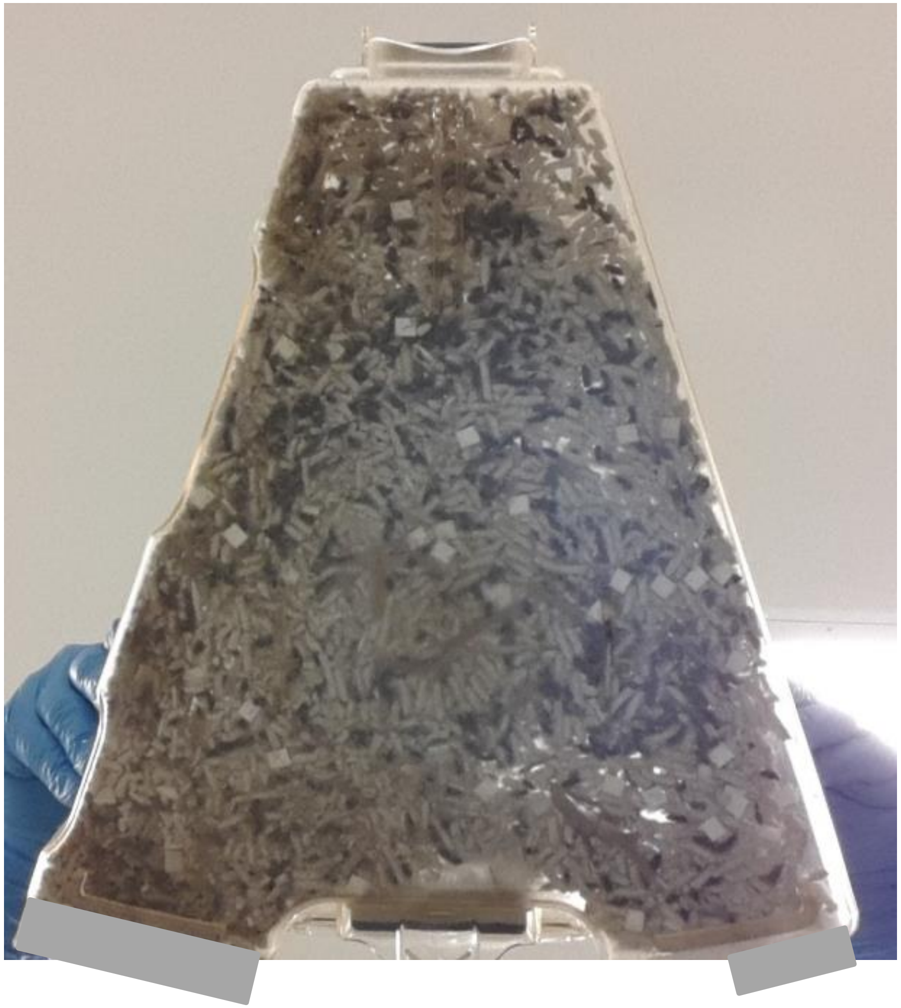

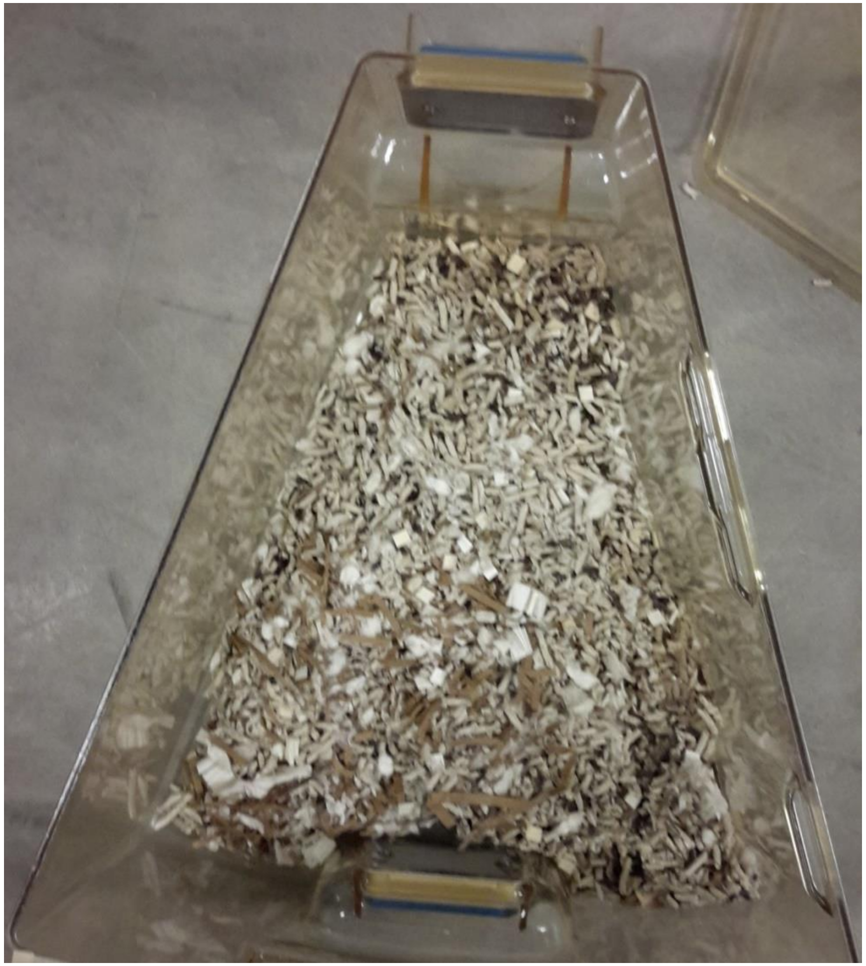

June 9 COMP 1 left

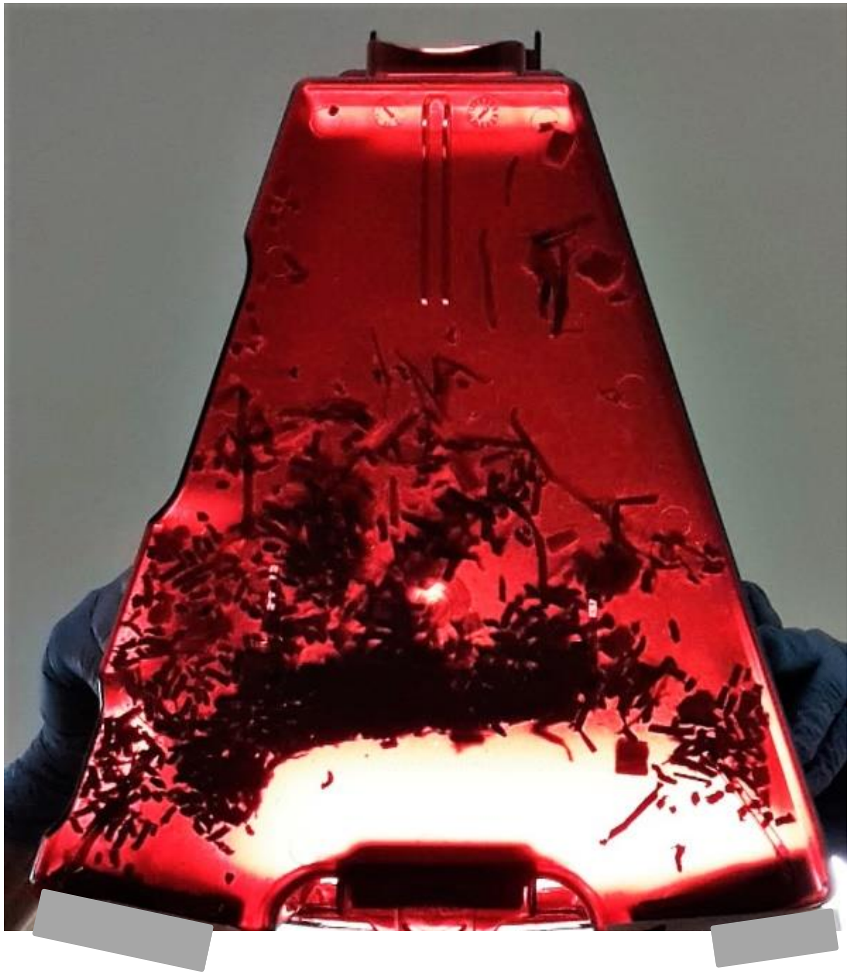

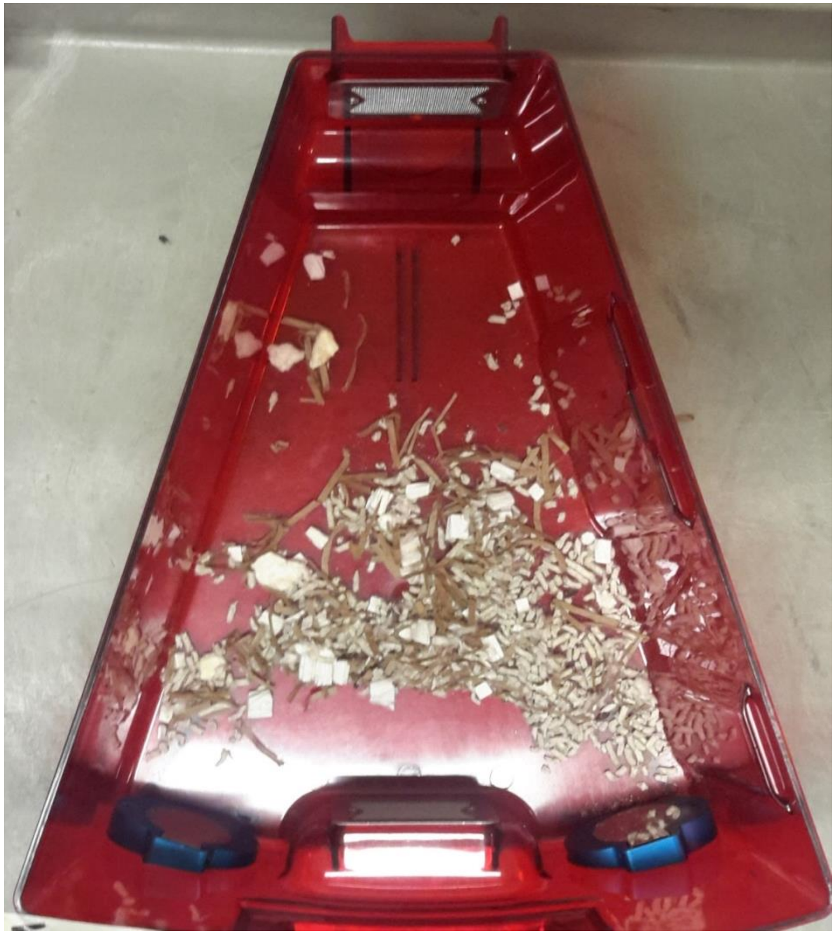

June 9 COMP 1 right

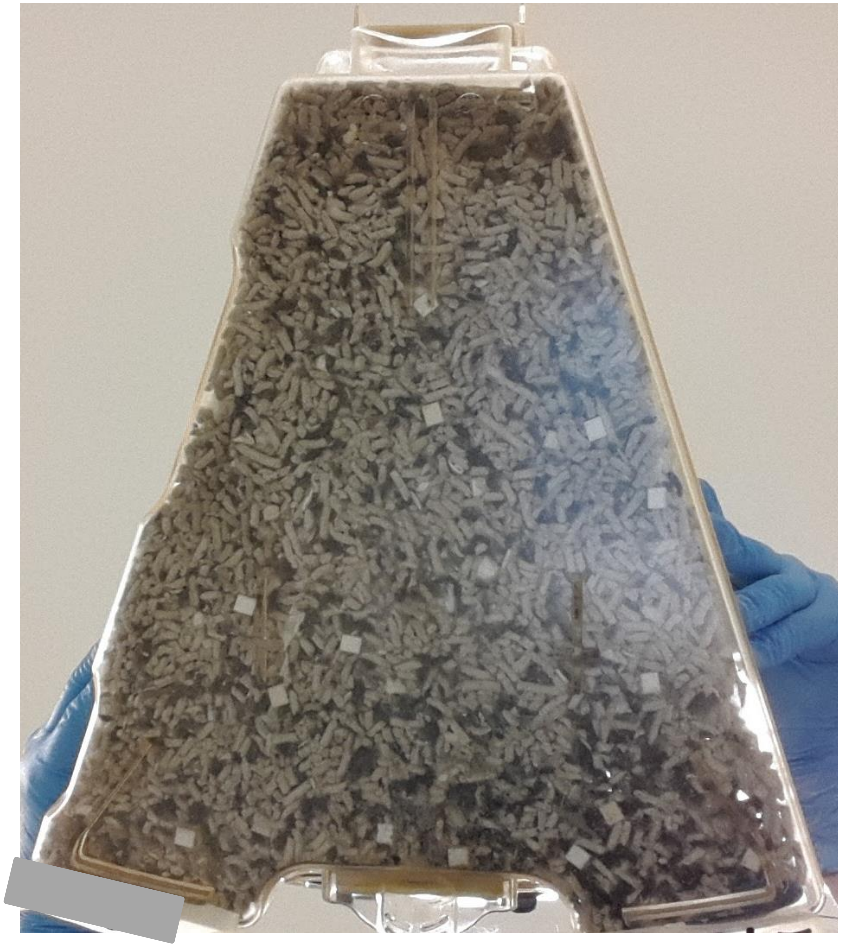

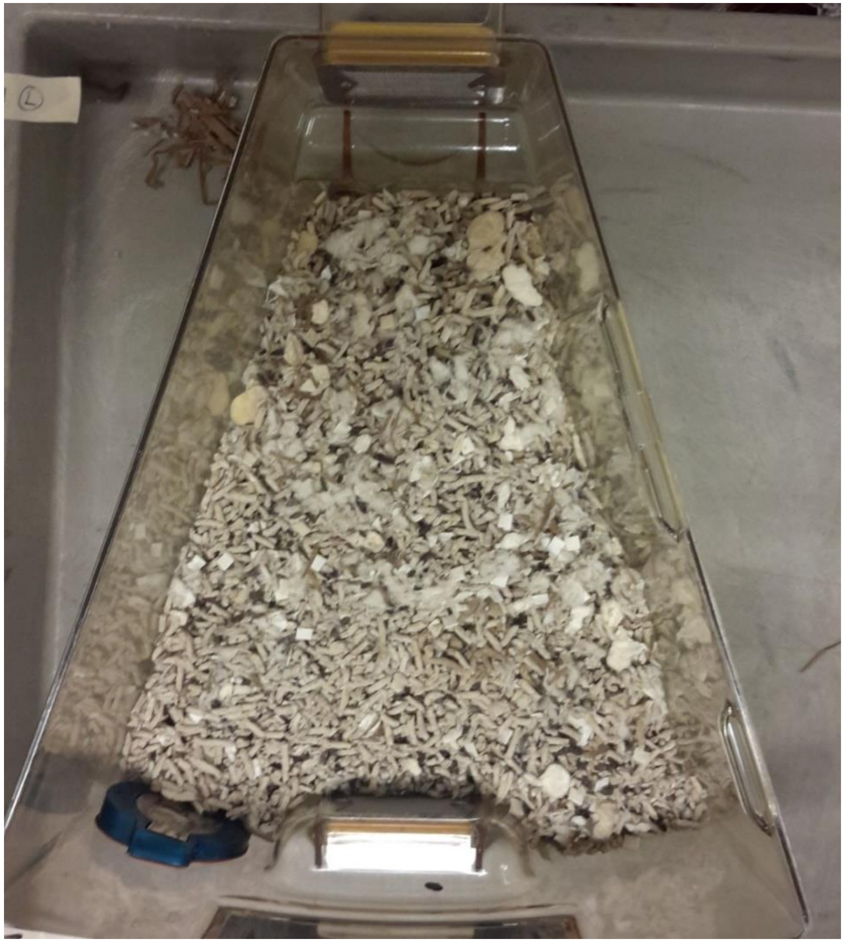

June 9 COMP 1 mid

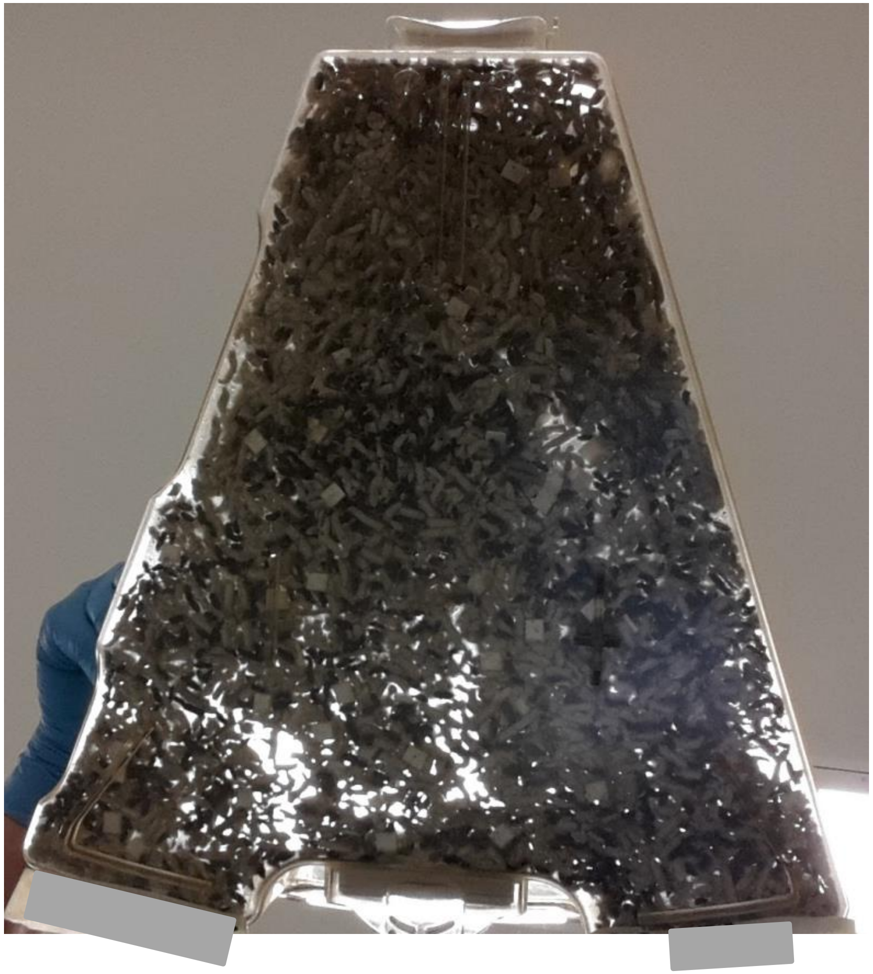

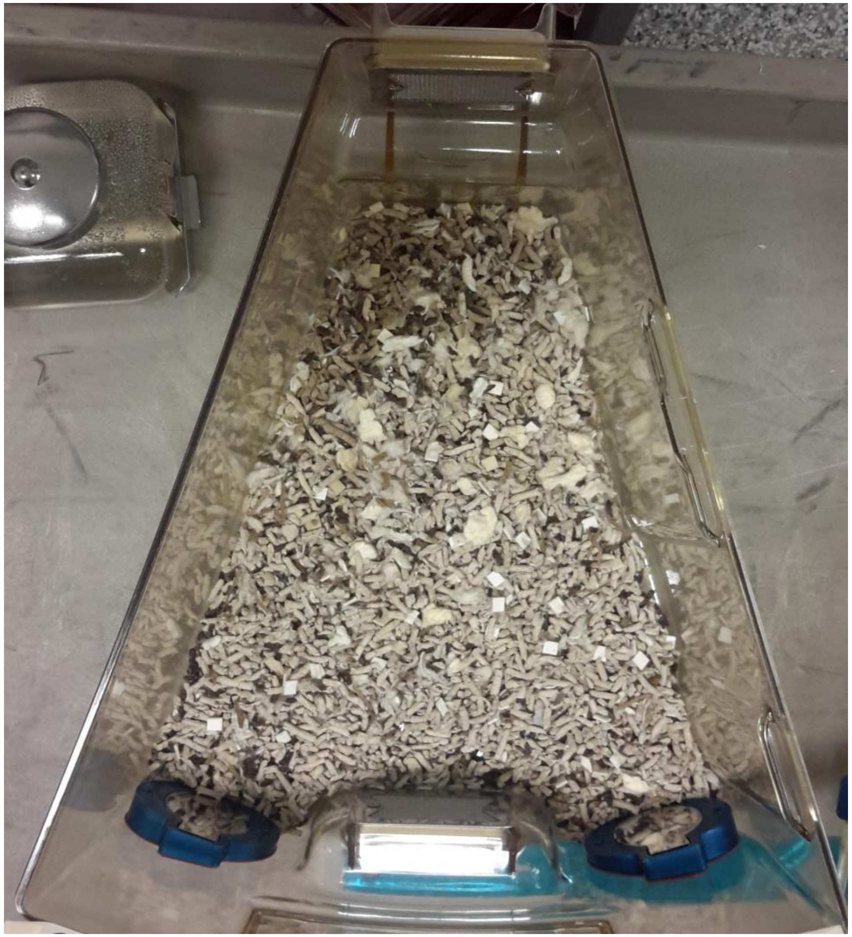

June 9 STD 1

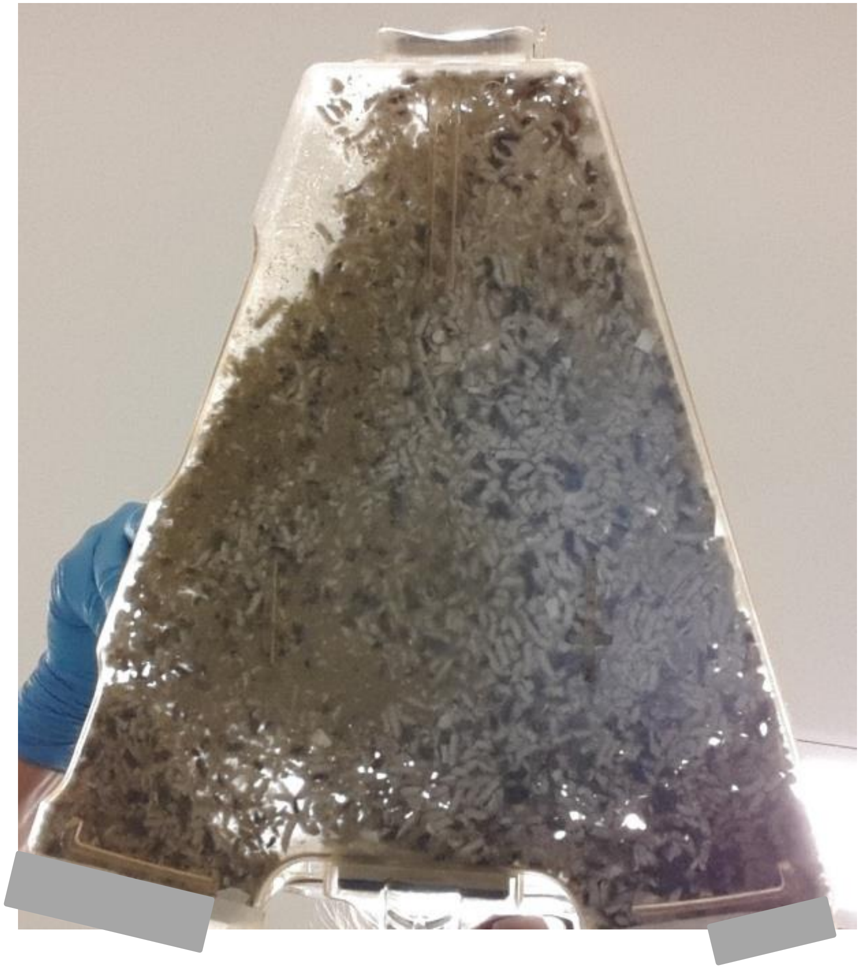

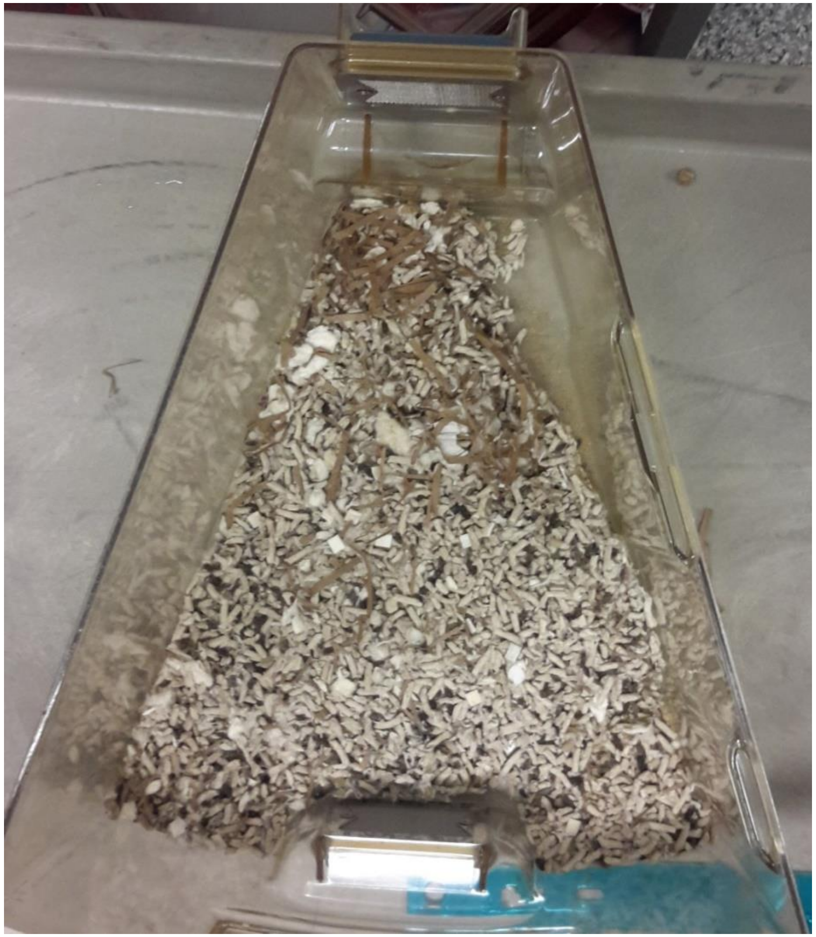

June 9 COMP 2 left

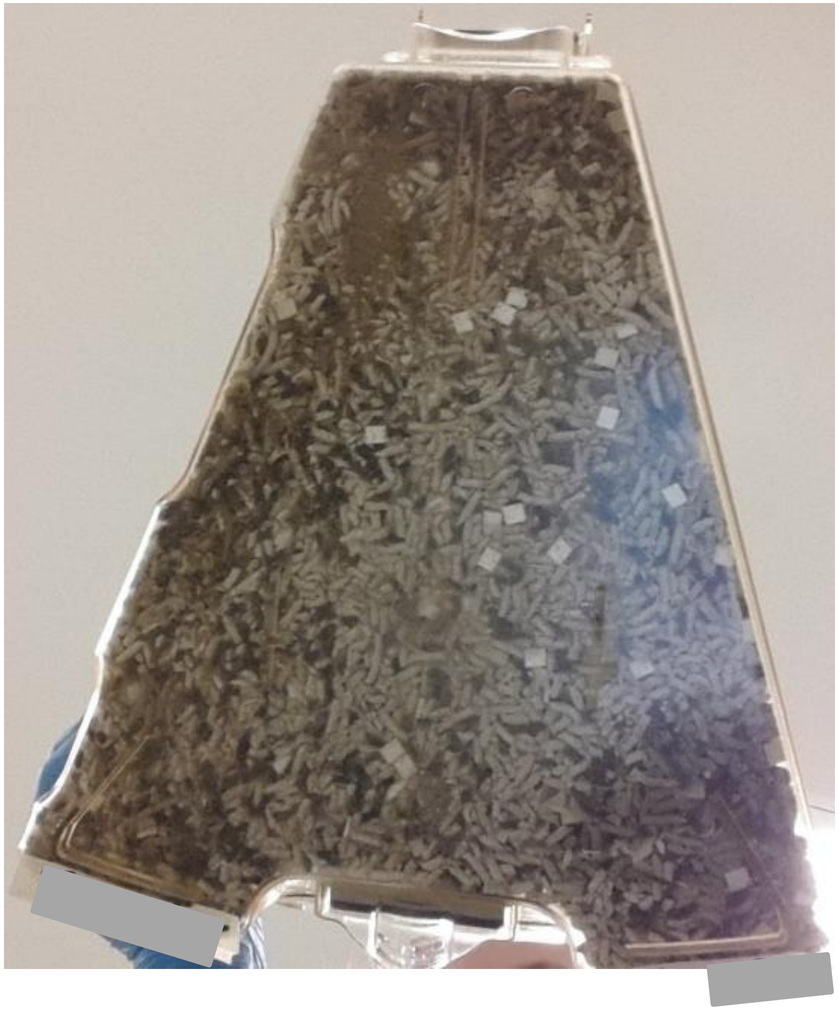

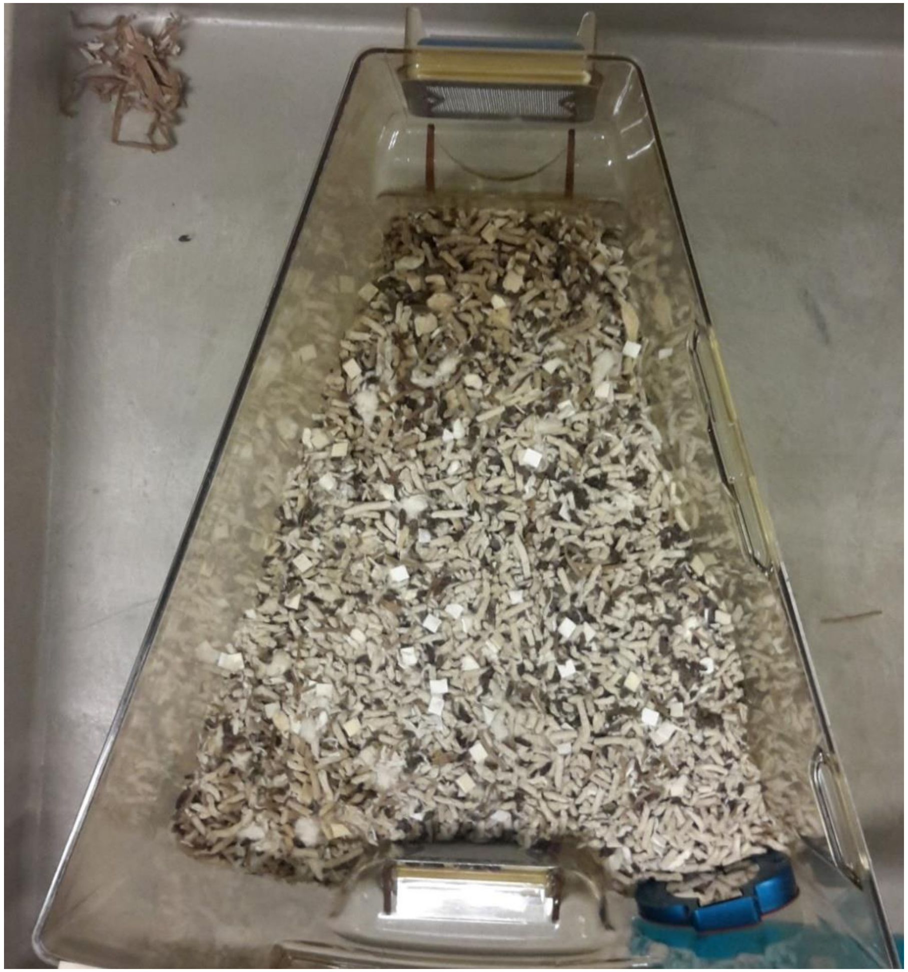

June 9 COMP 2 mid

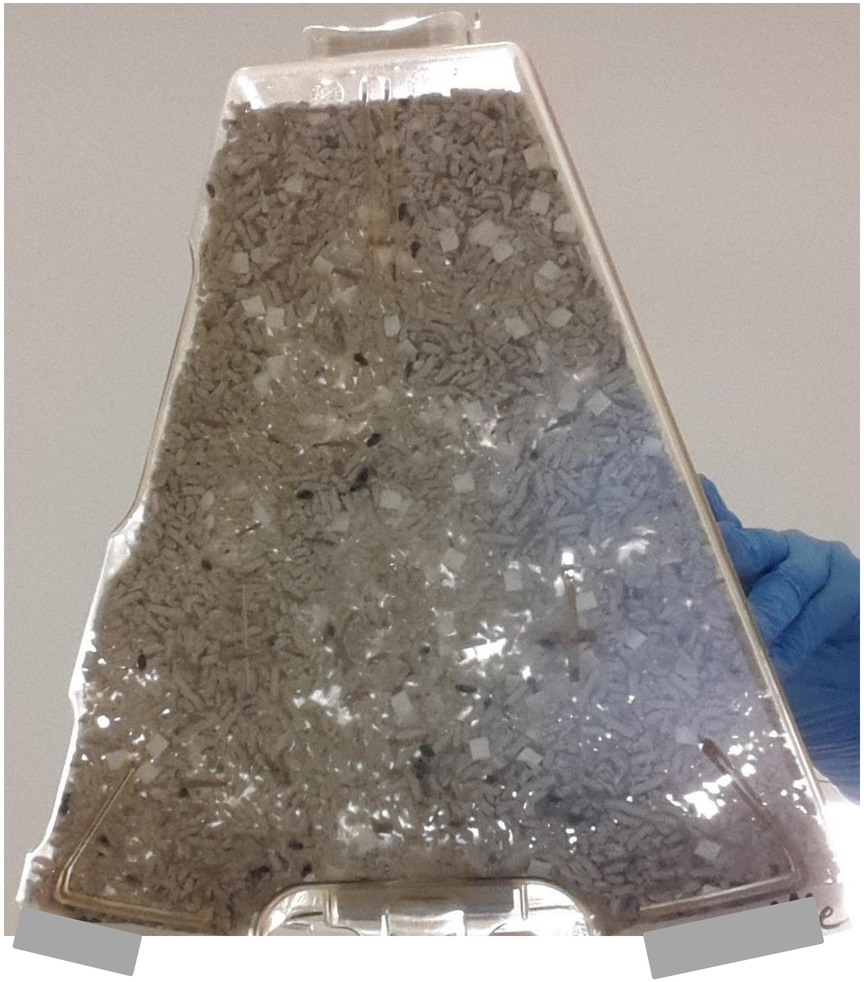

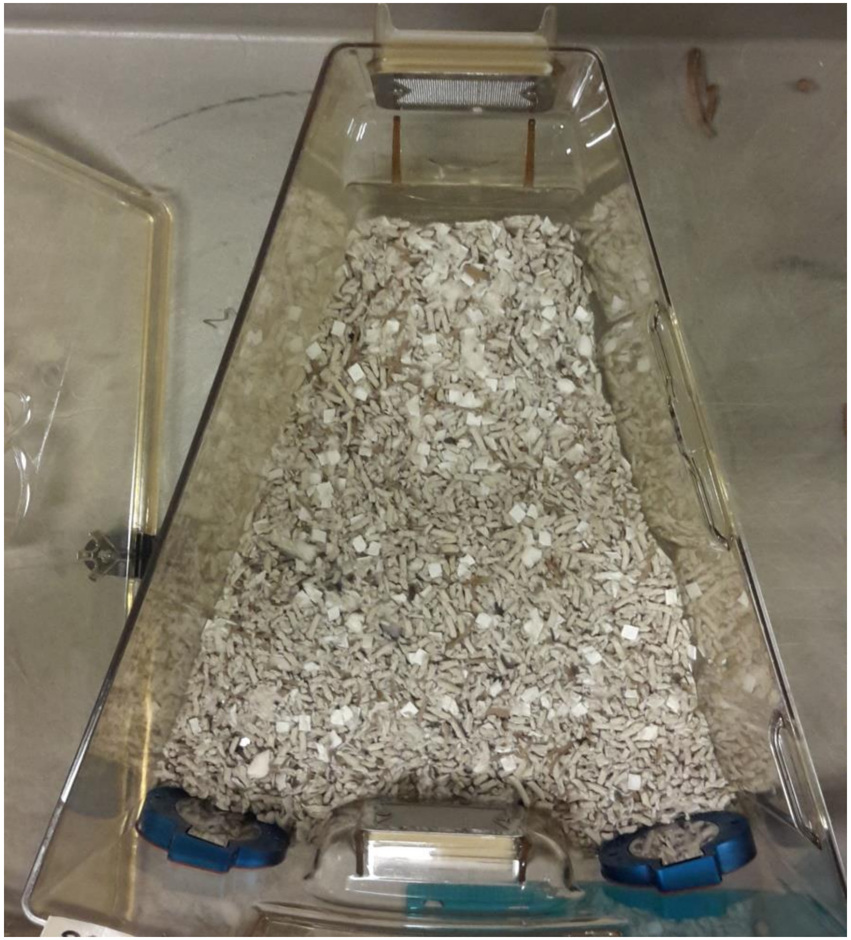

June 9 COMP 2 right

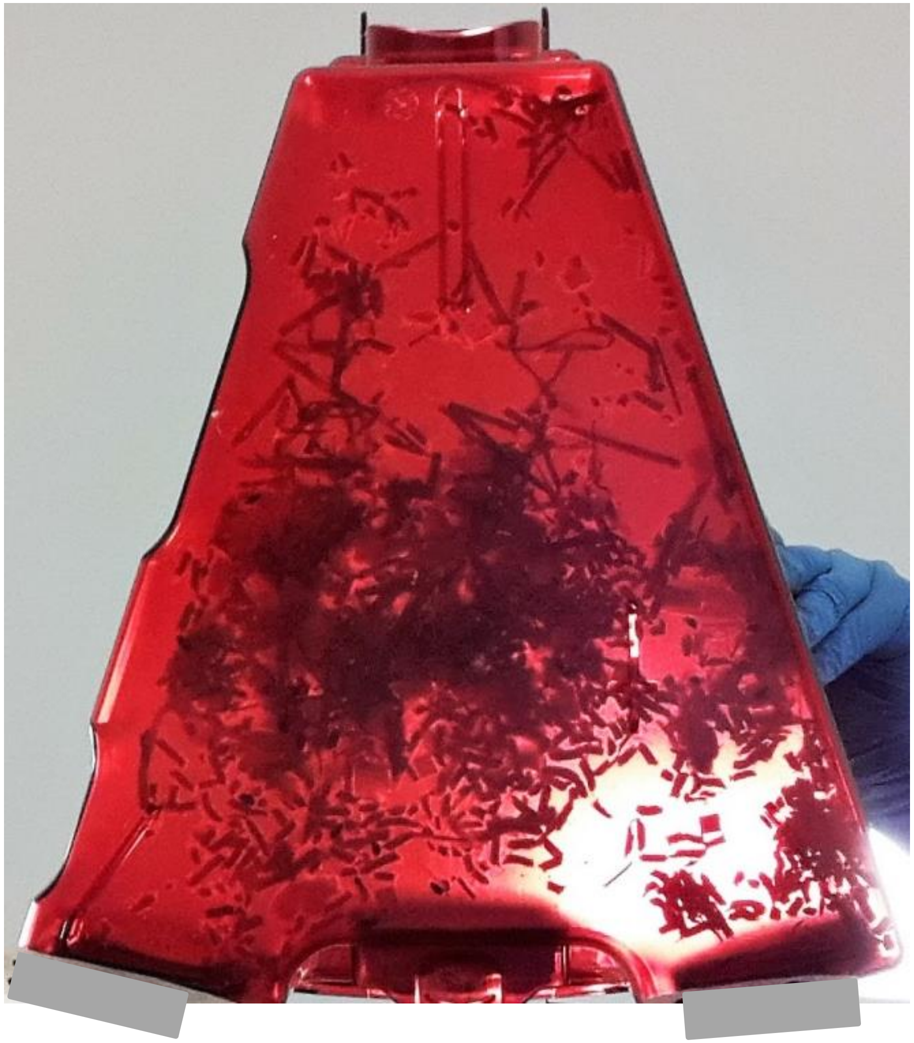

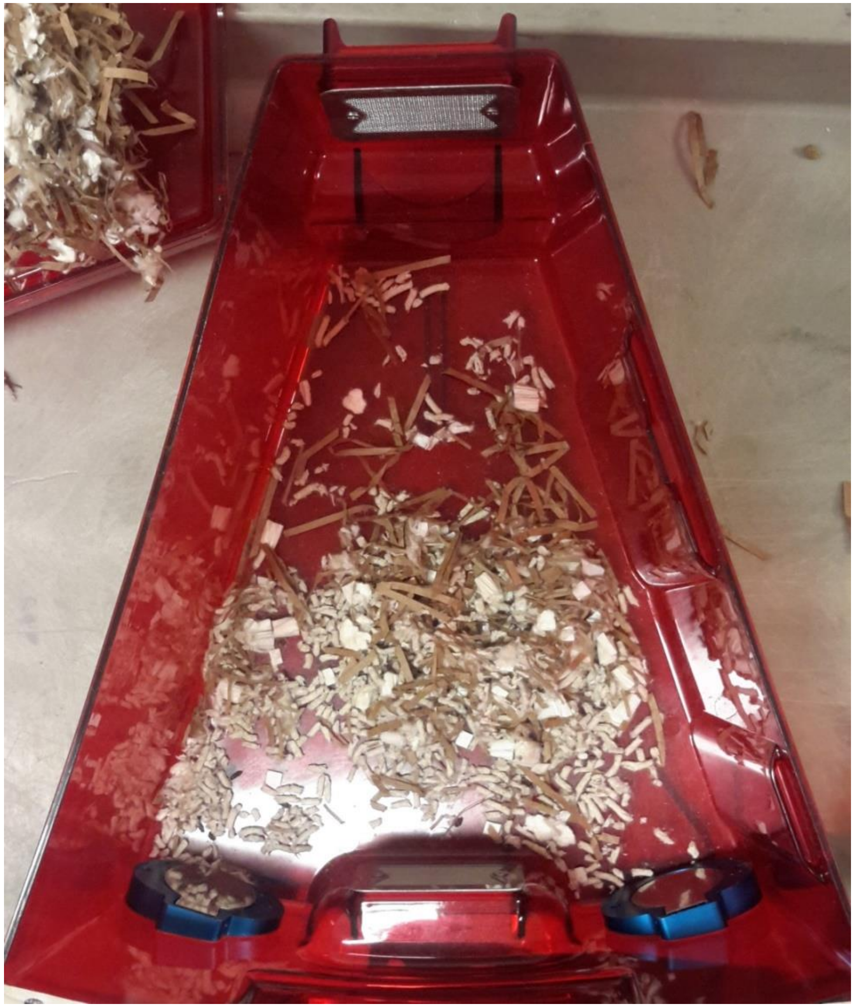

June 9 STD 2

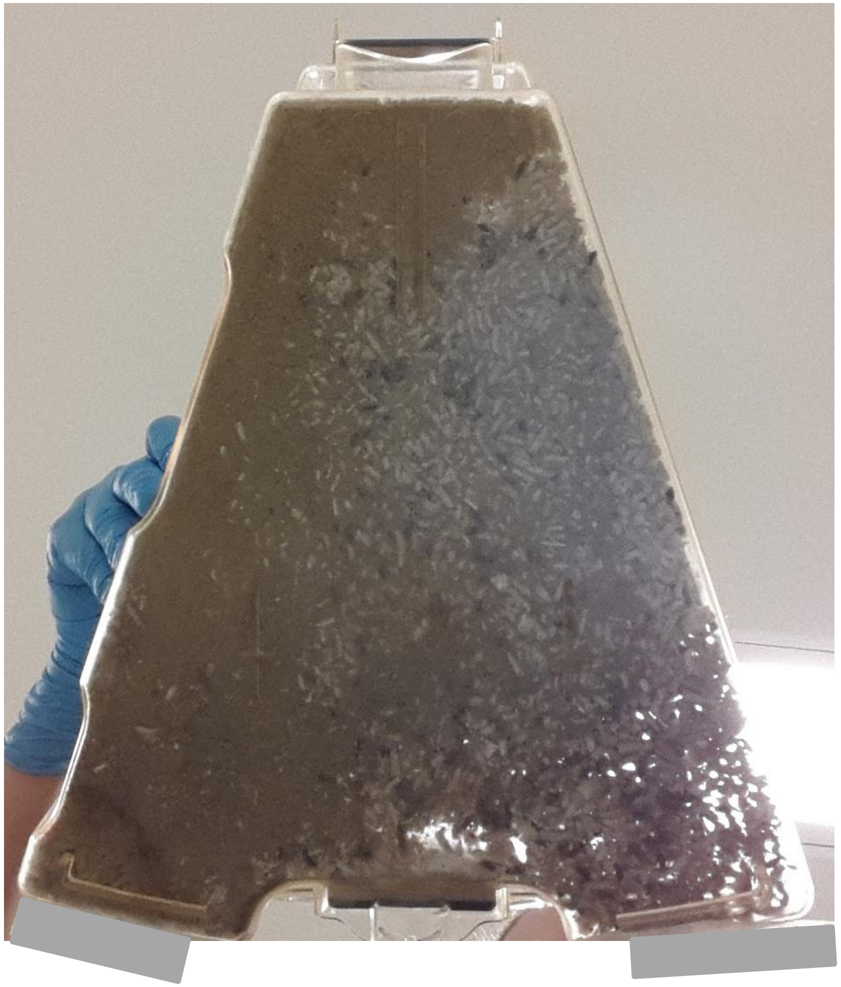

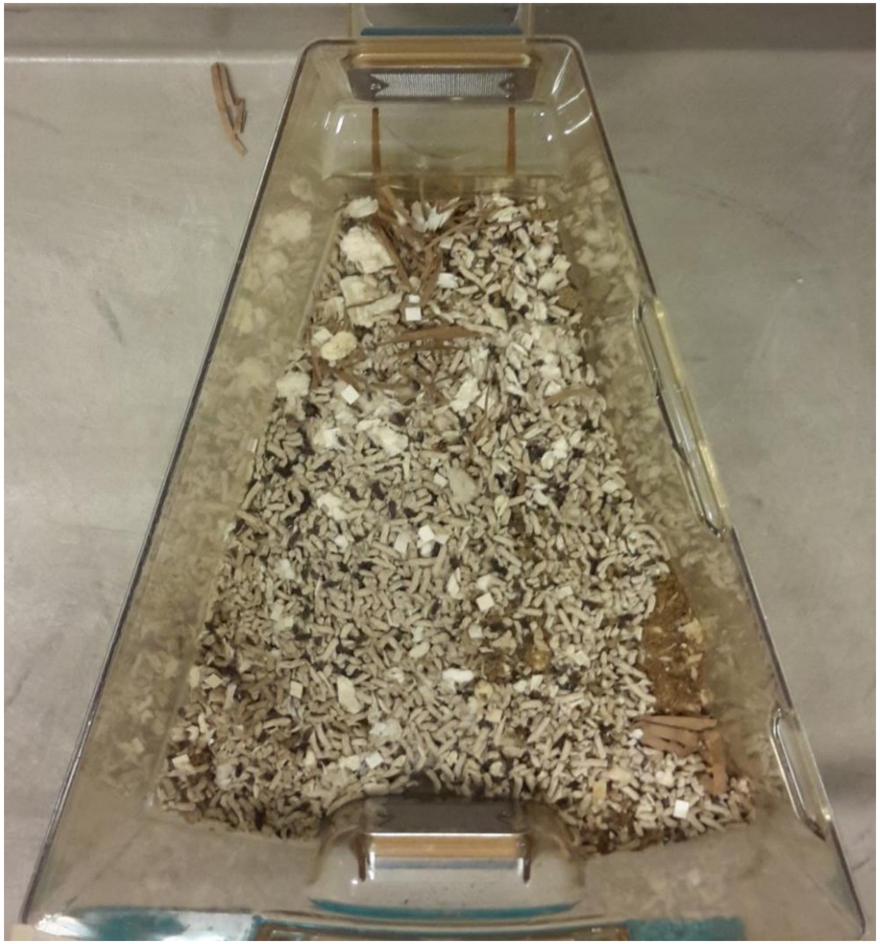

June 10 COMP 3 right

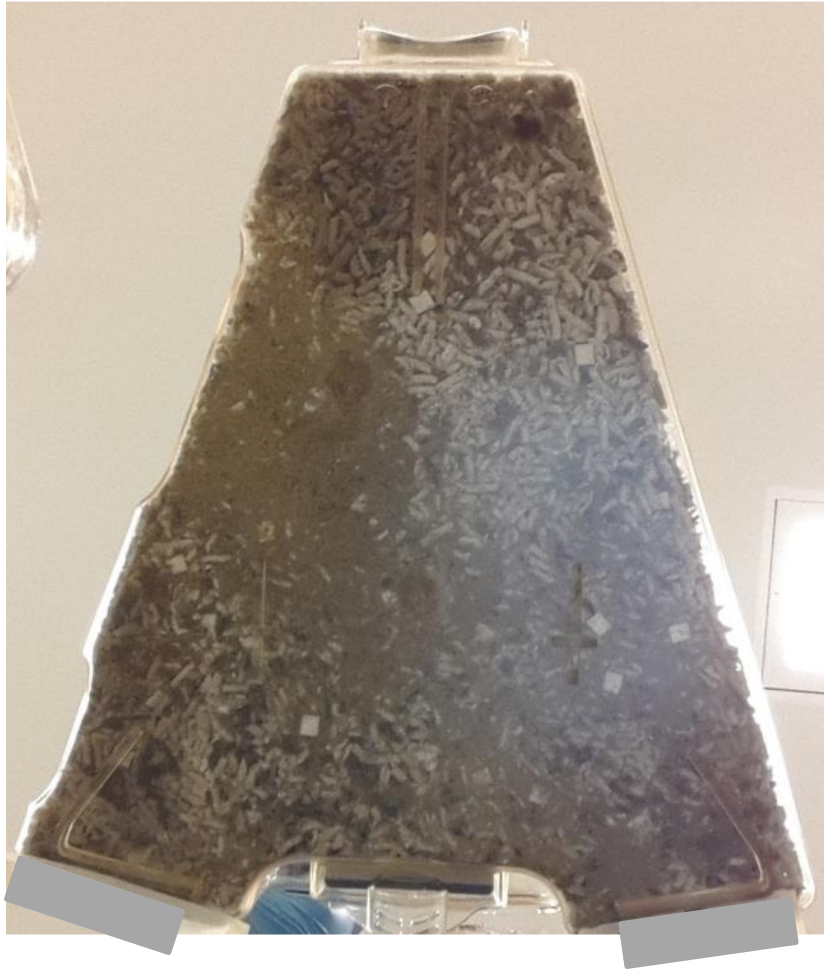

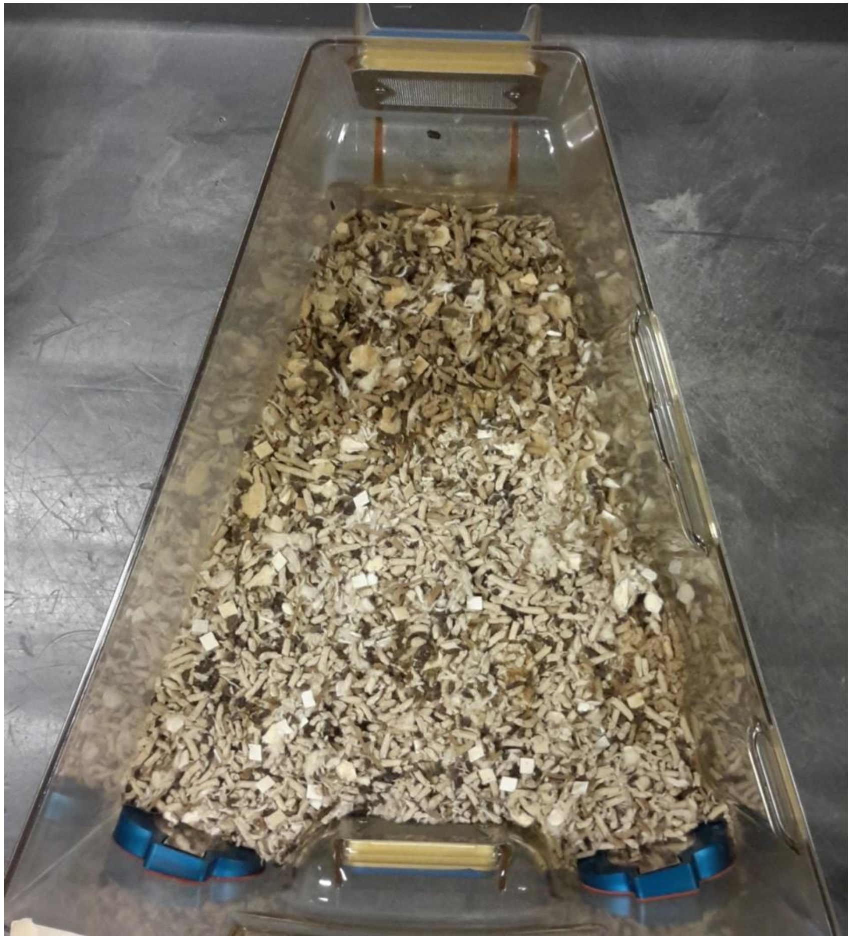

June 10 COMP 3 mid

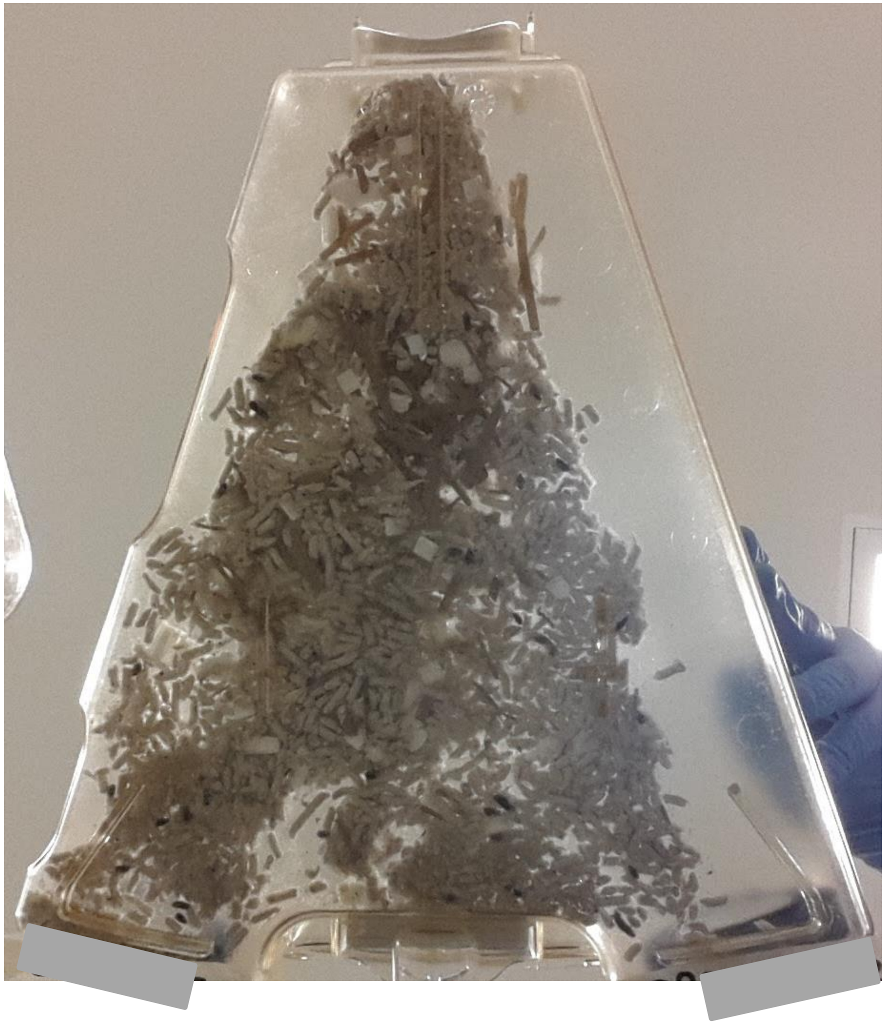

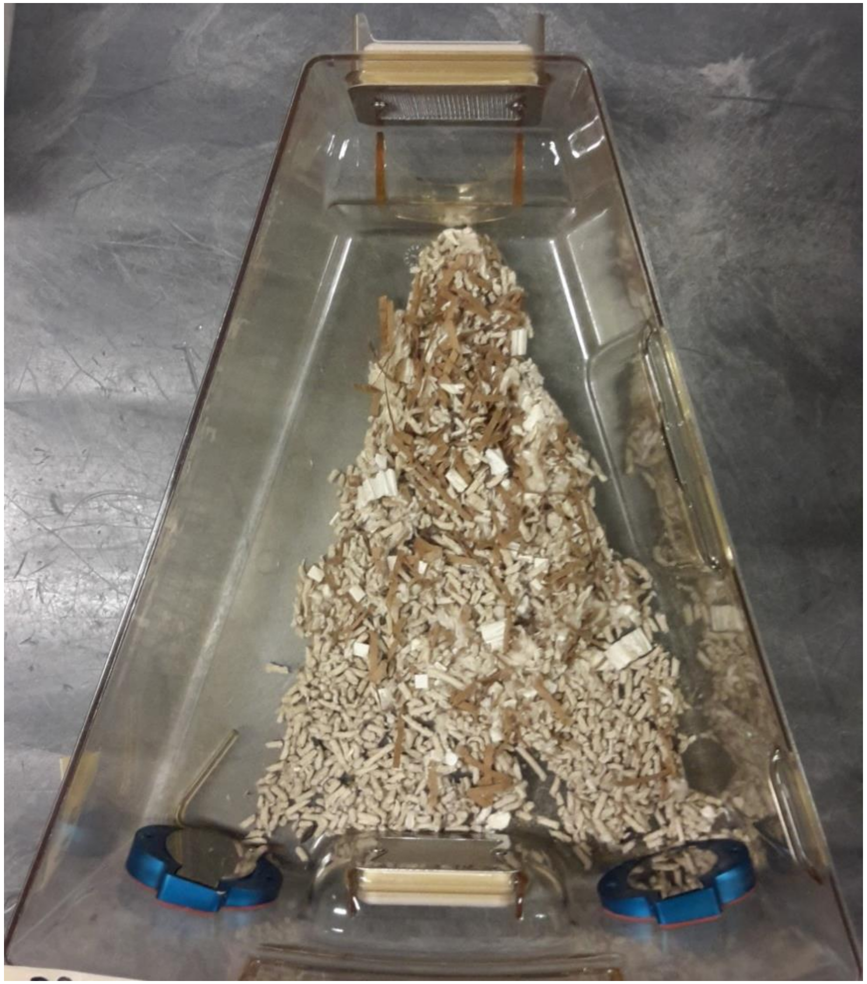

June 10 COMP 3 left

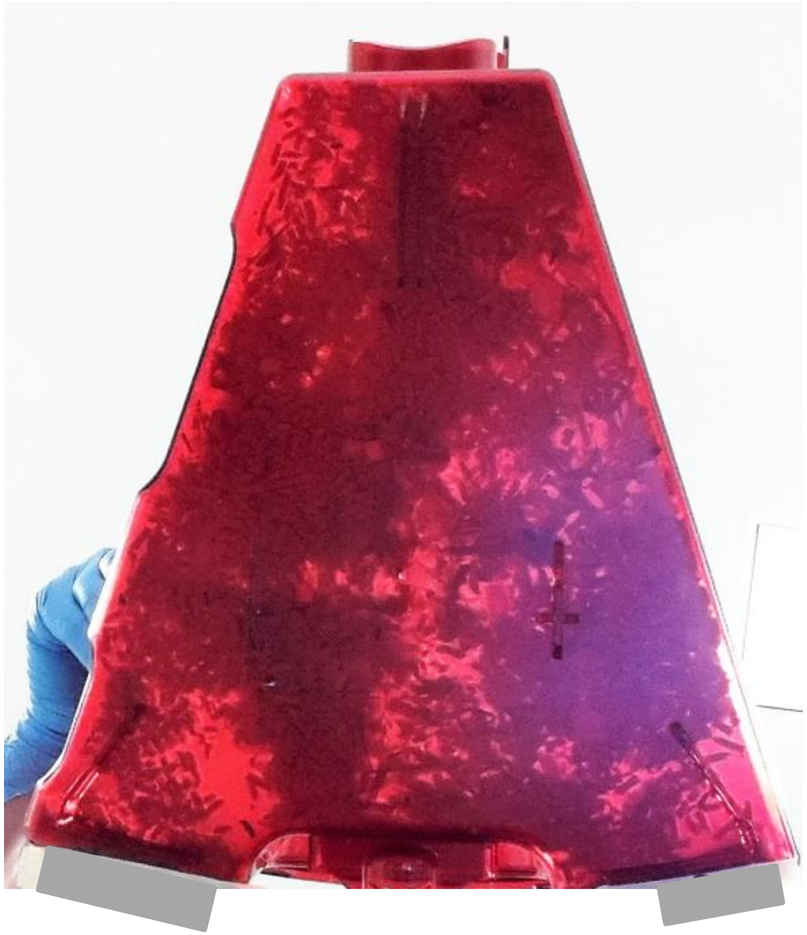

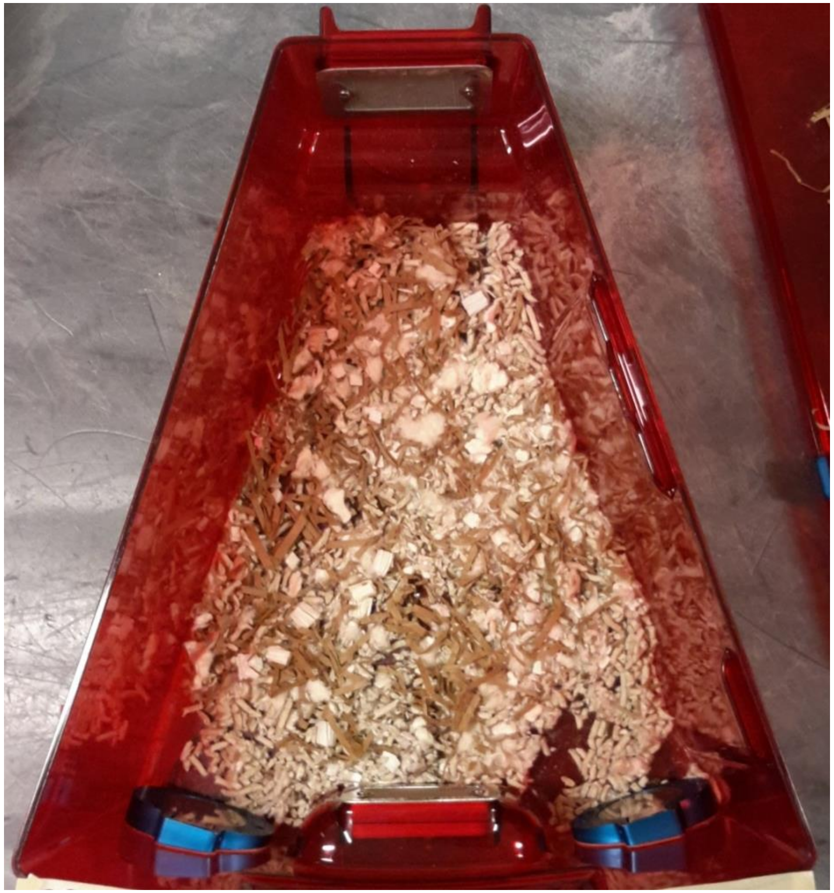

June 10 STD 3

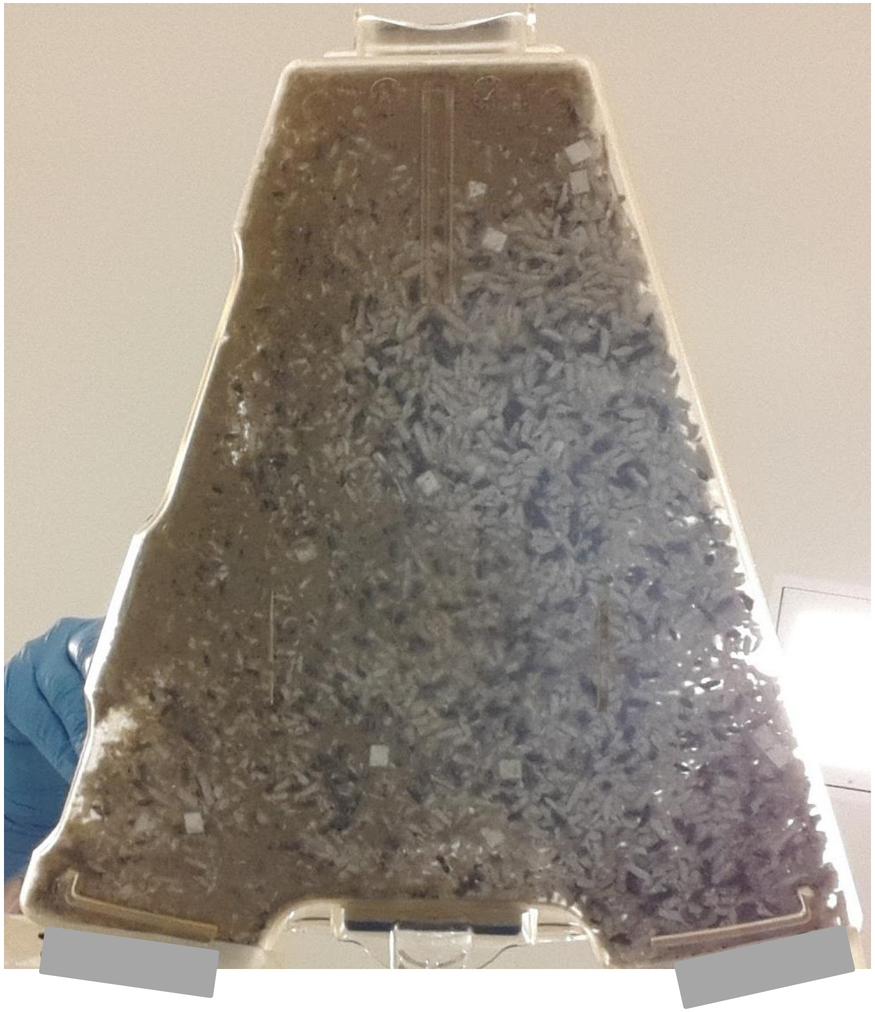

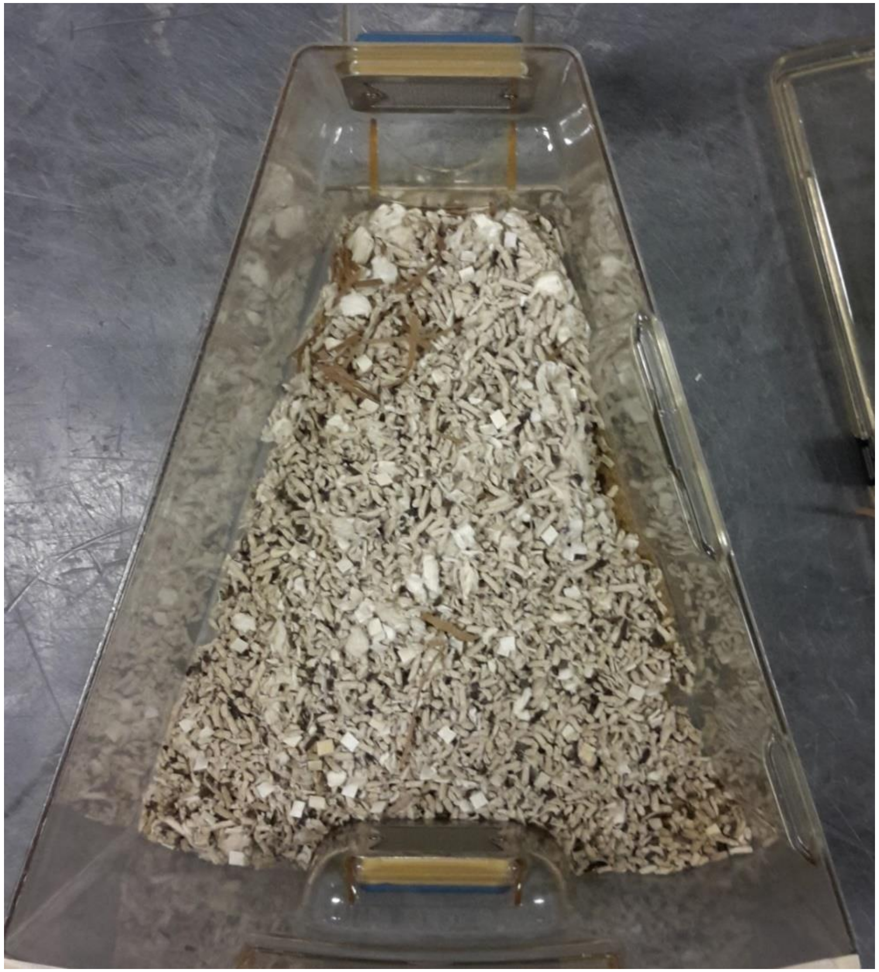

June 10 COMP 4 right

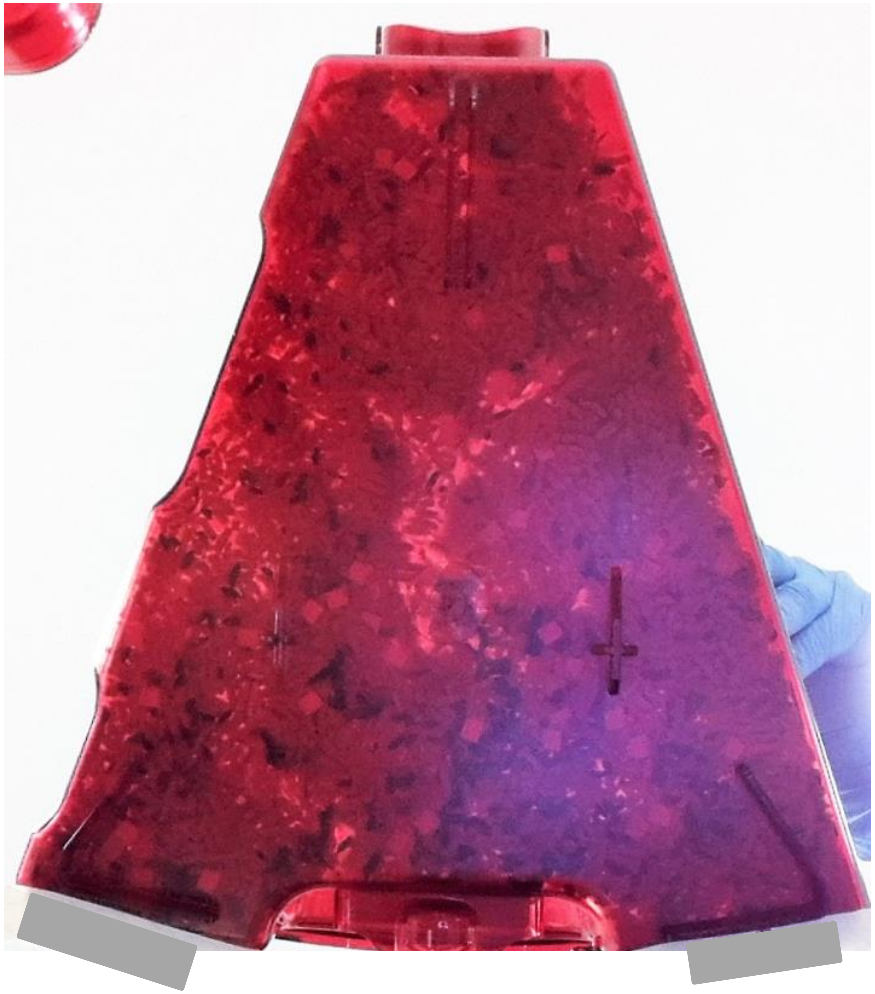

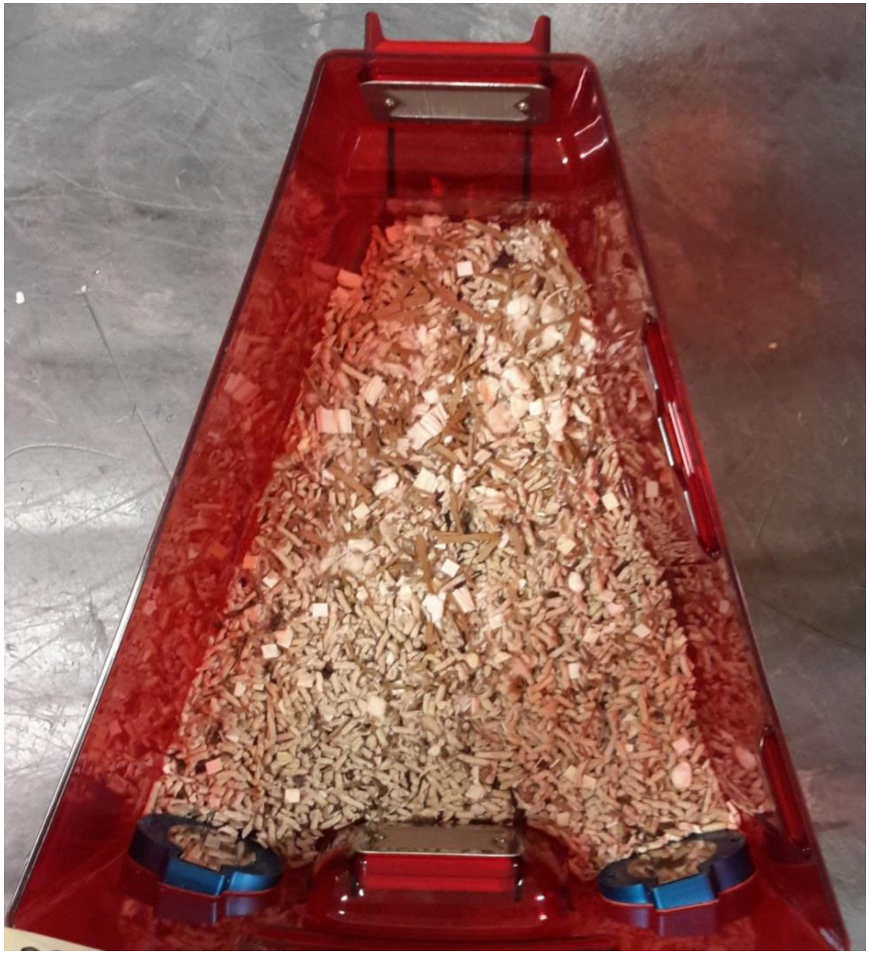

June 10 COMP 4 mid

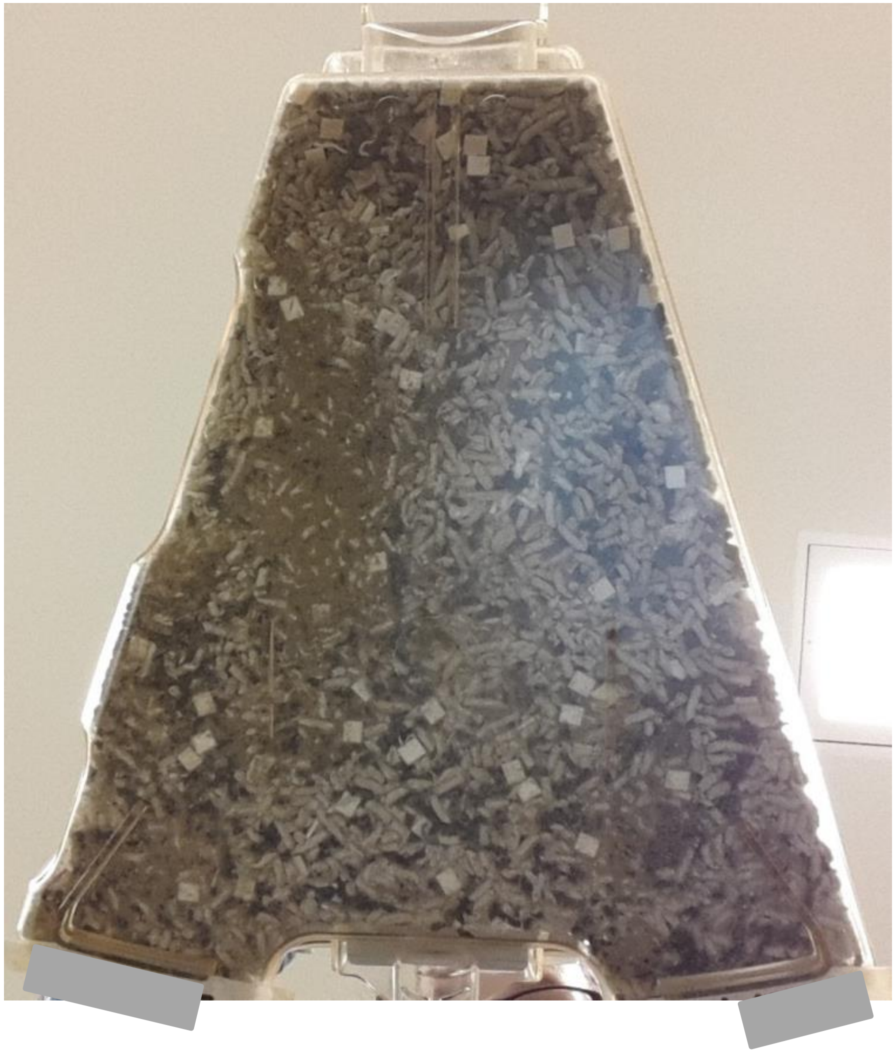

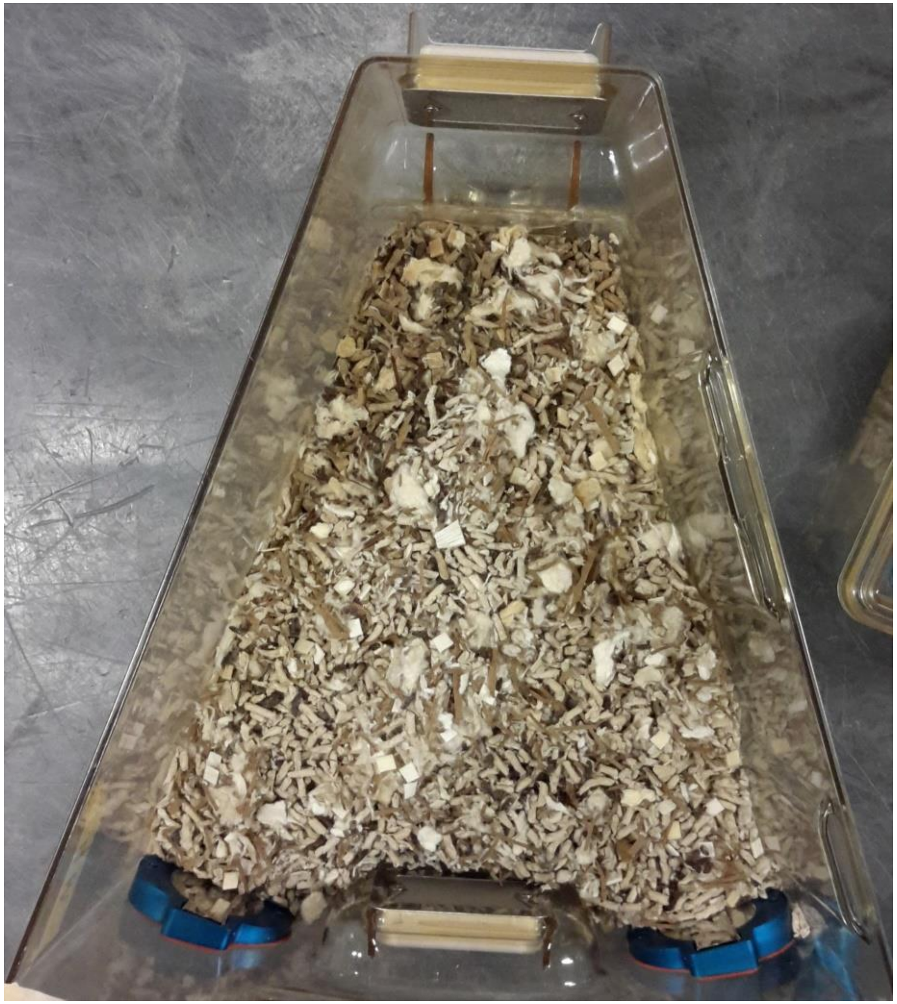

June 10 COMP 4 left

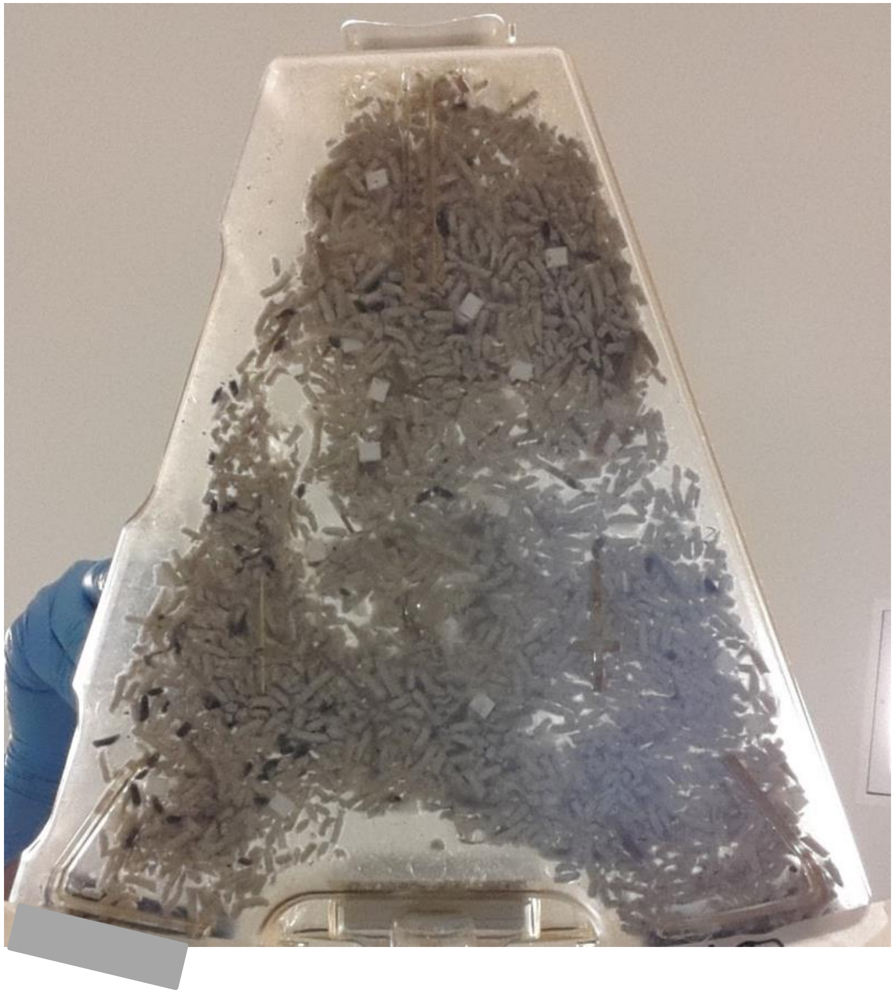

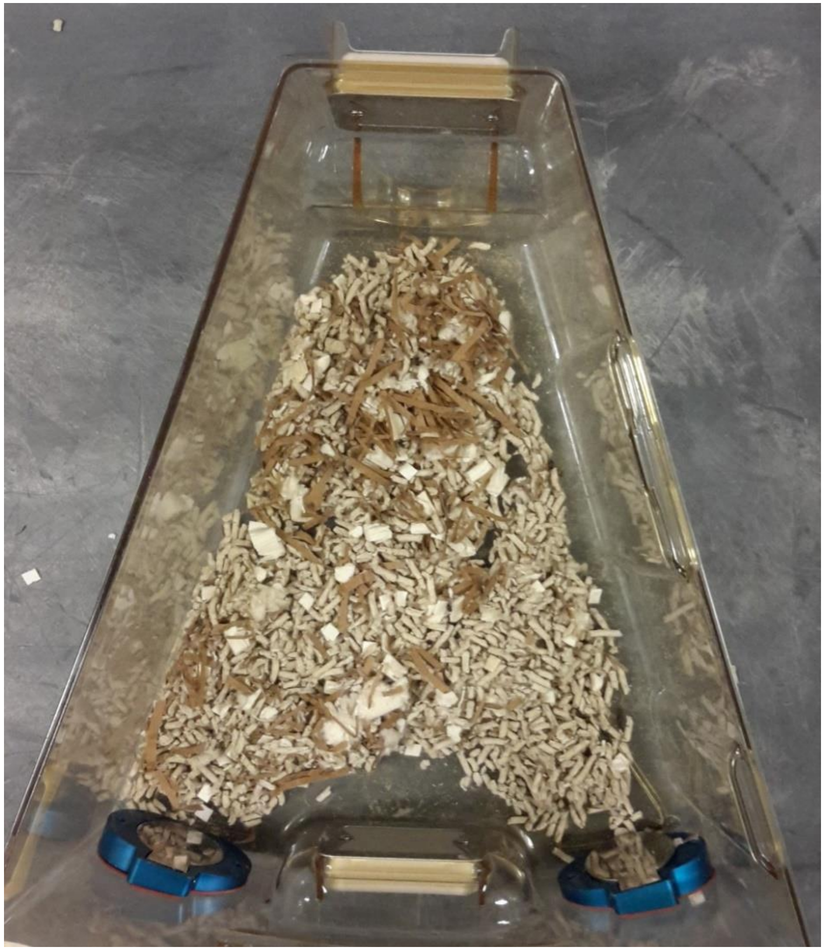

June 10 STD 4

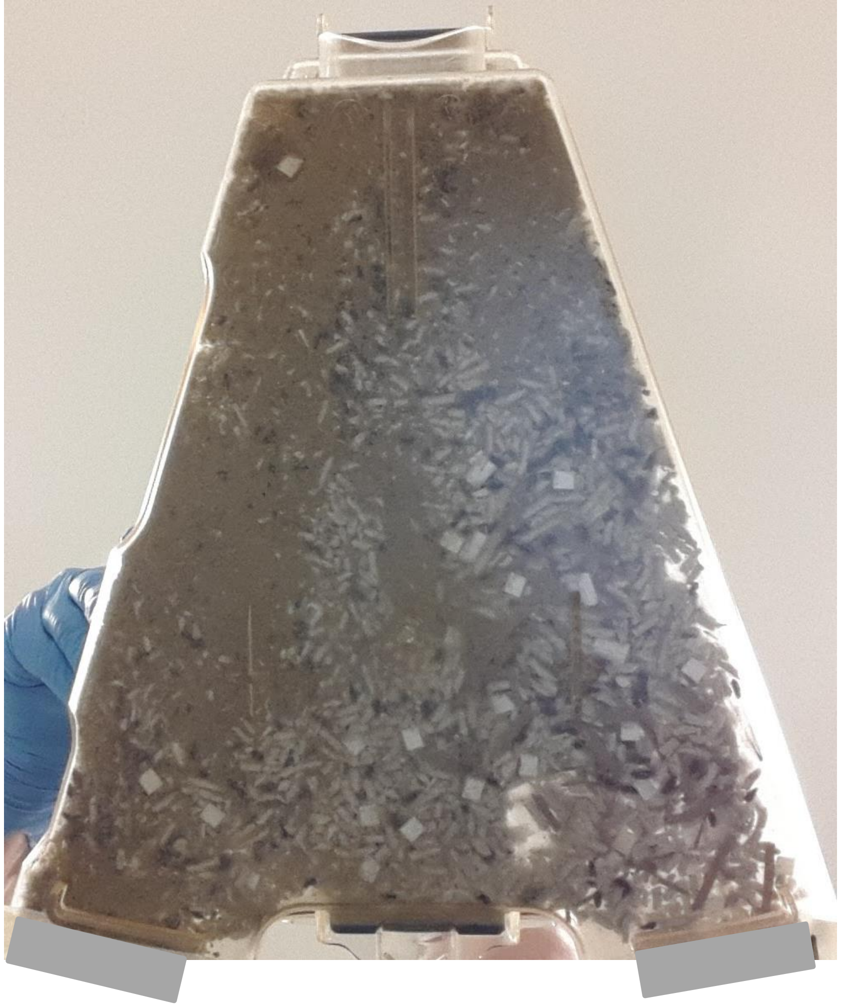

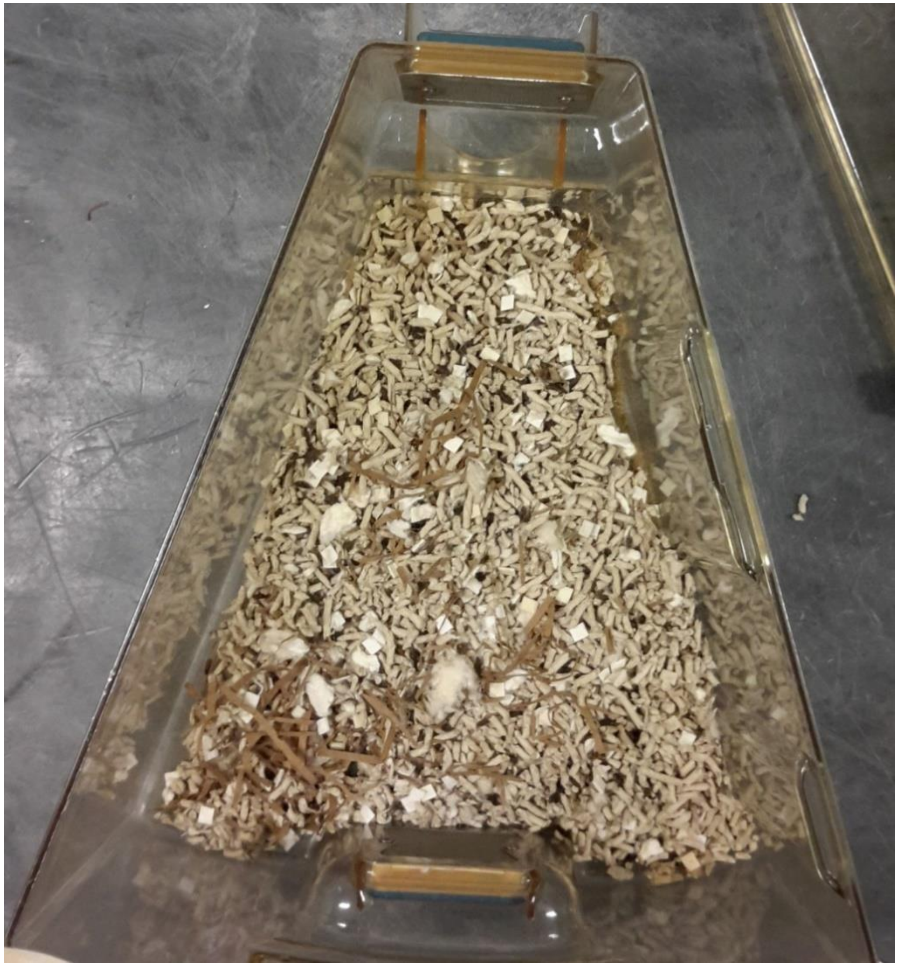

June 11 COMP 5 mid

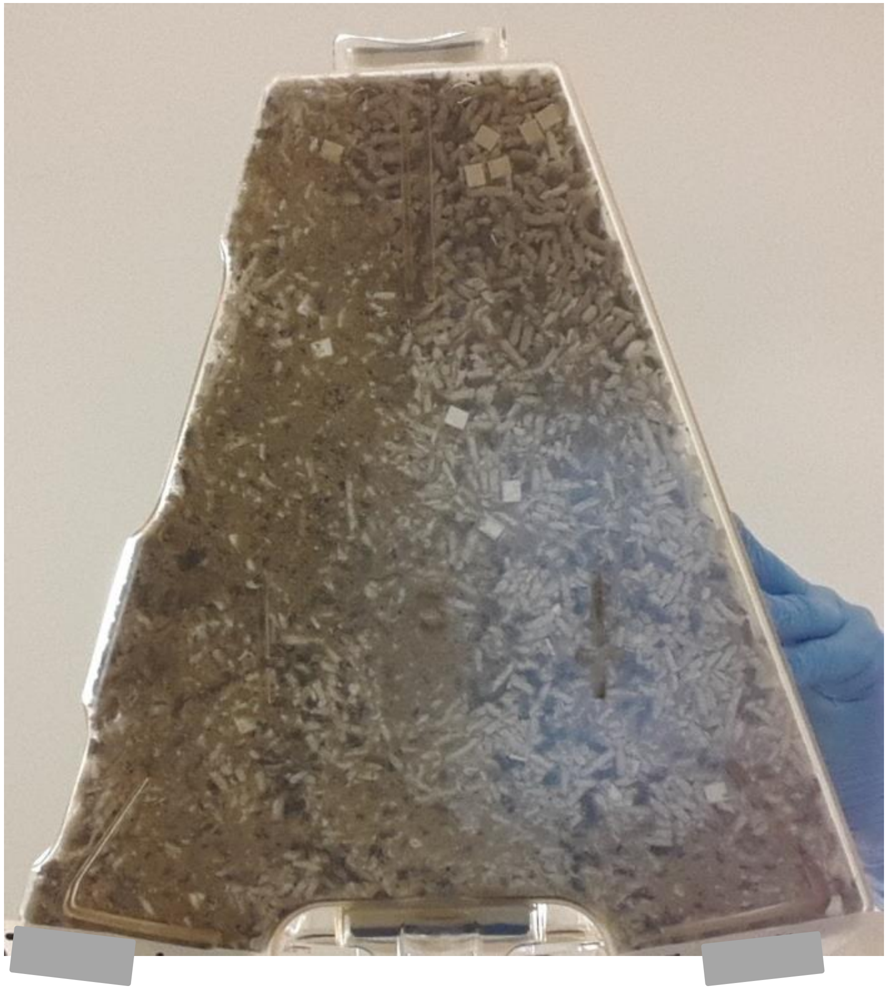

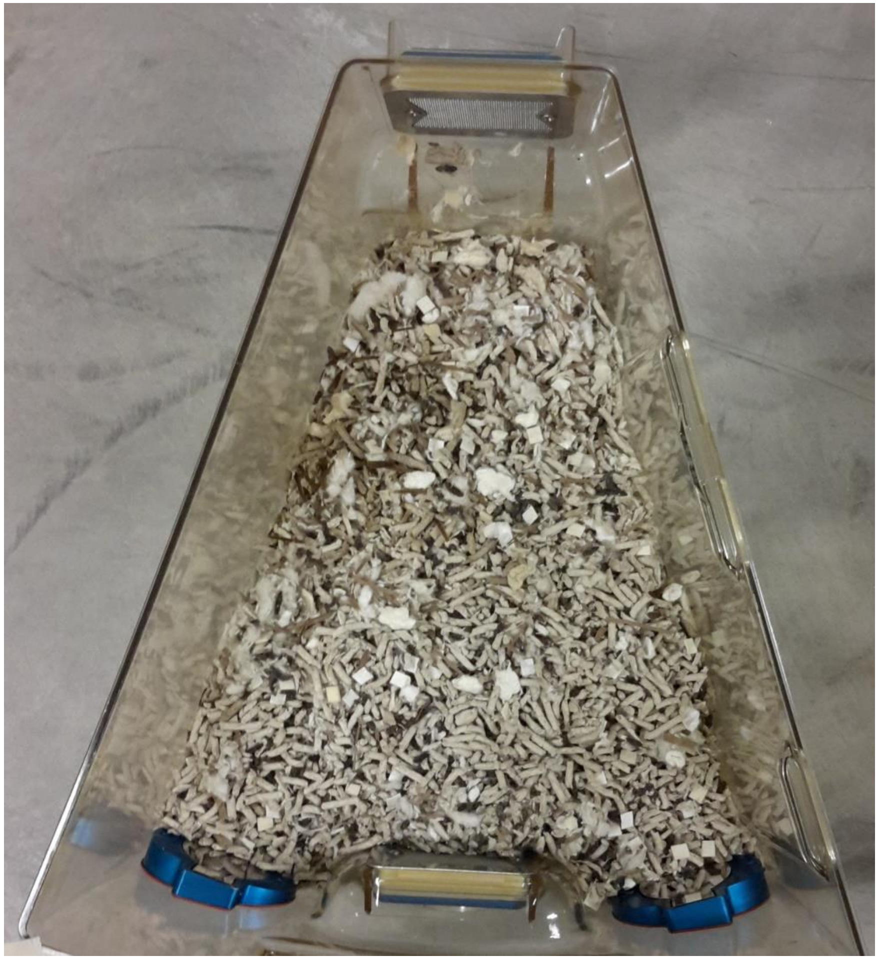

June 11 COMP 5 right

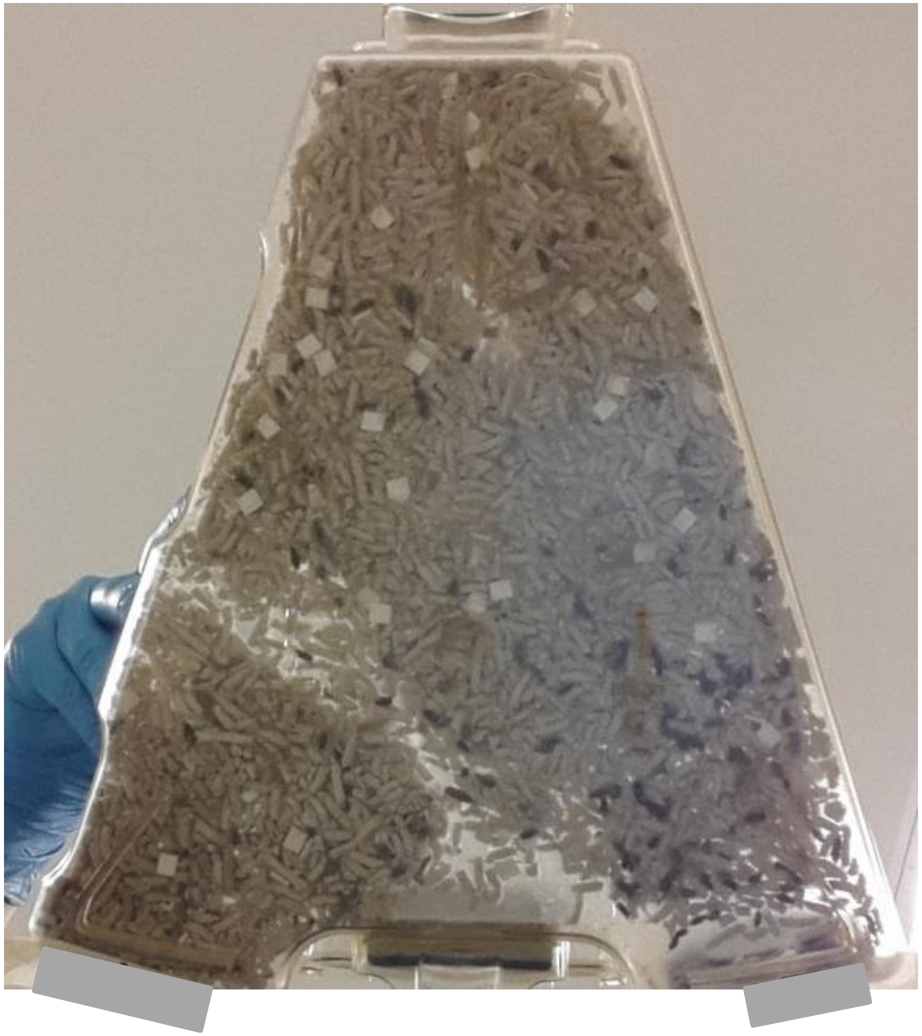

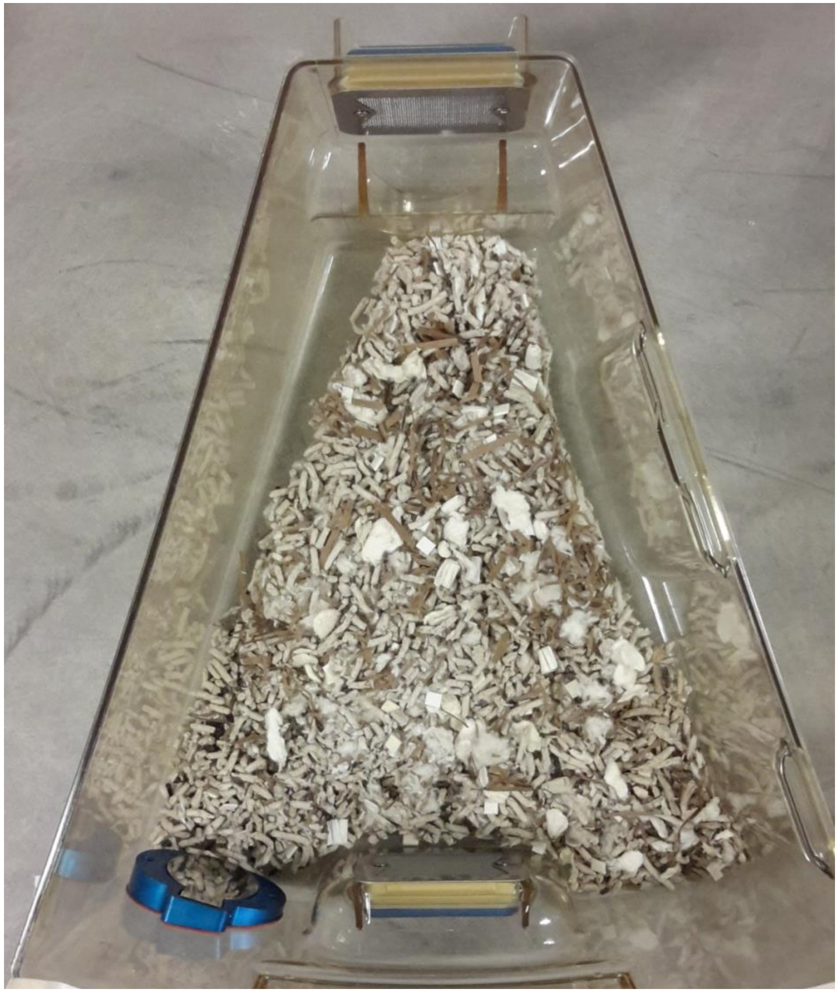

June 11 COMP 5 left

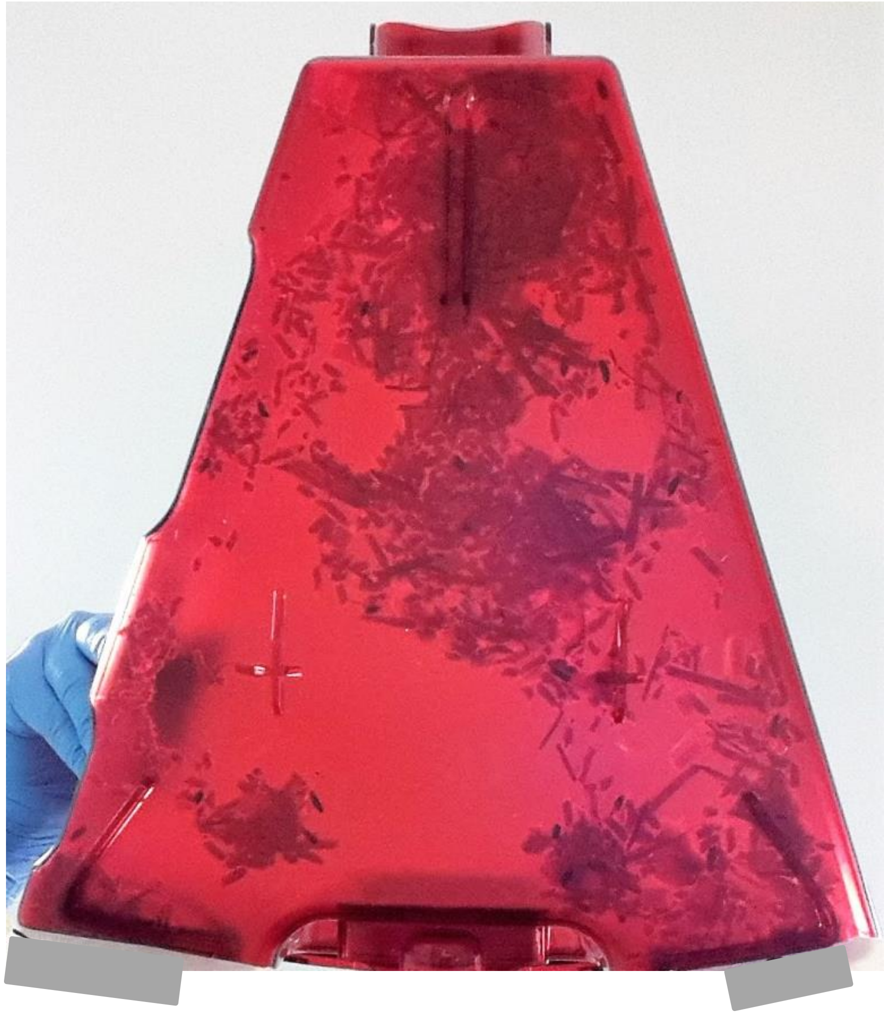

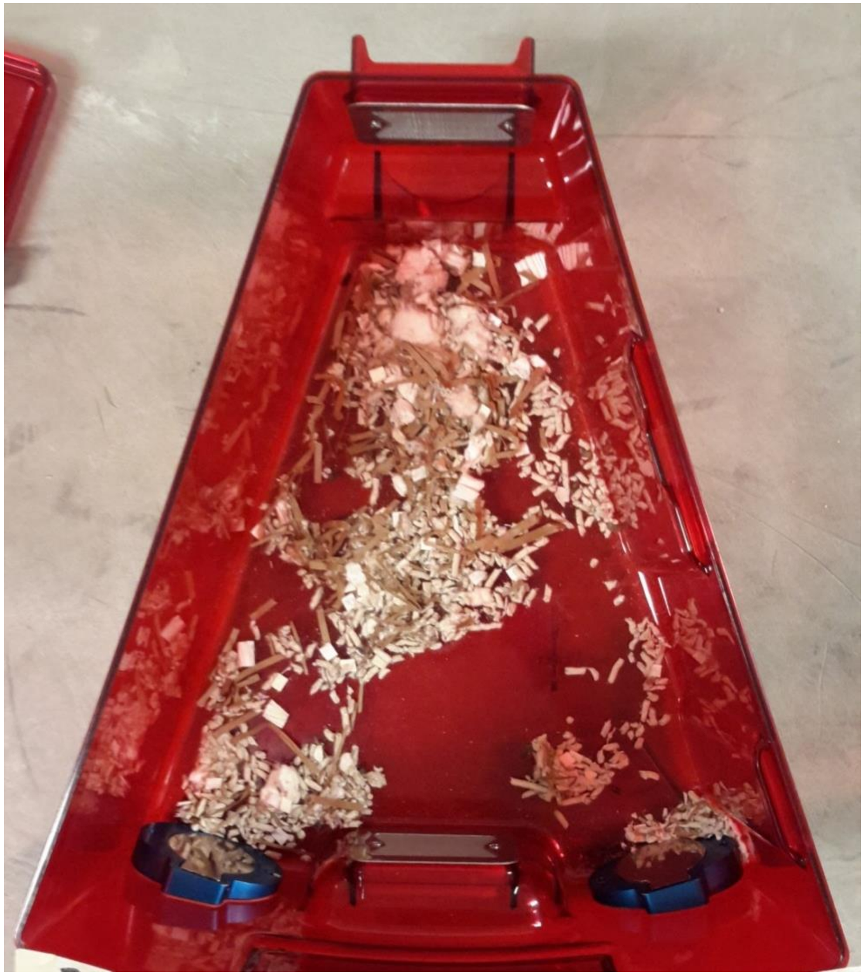

June 11 STD 5

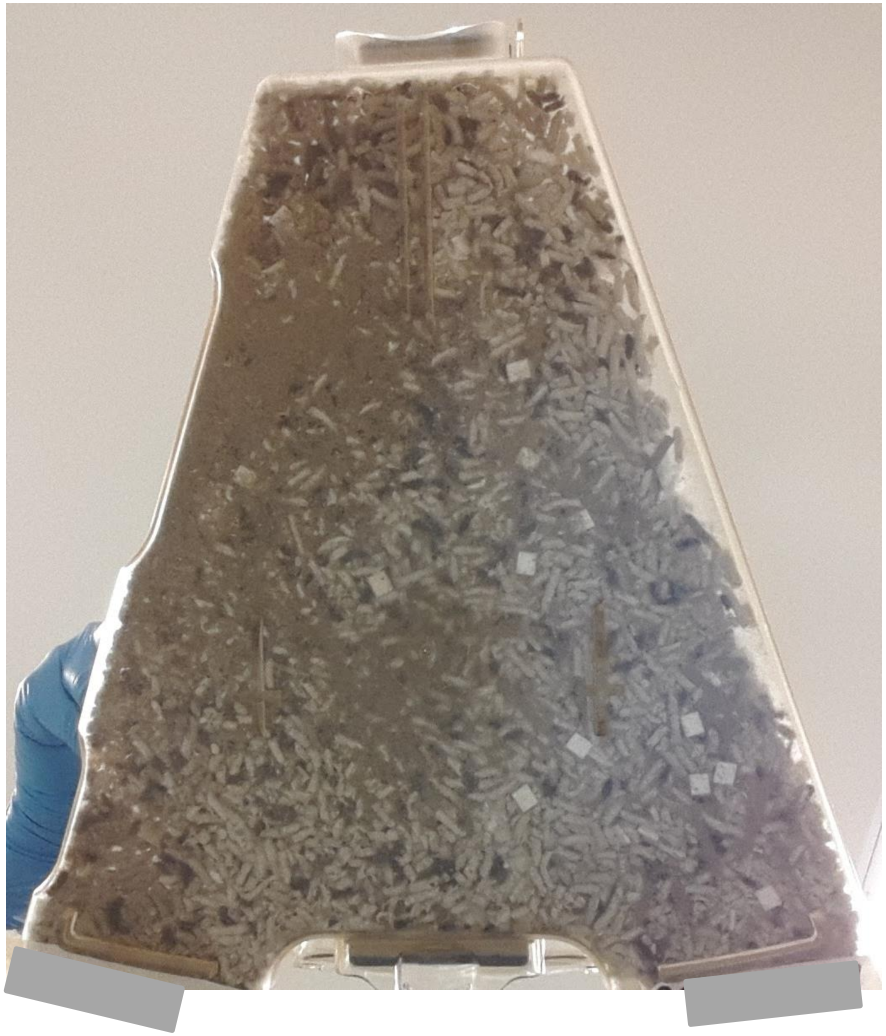

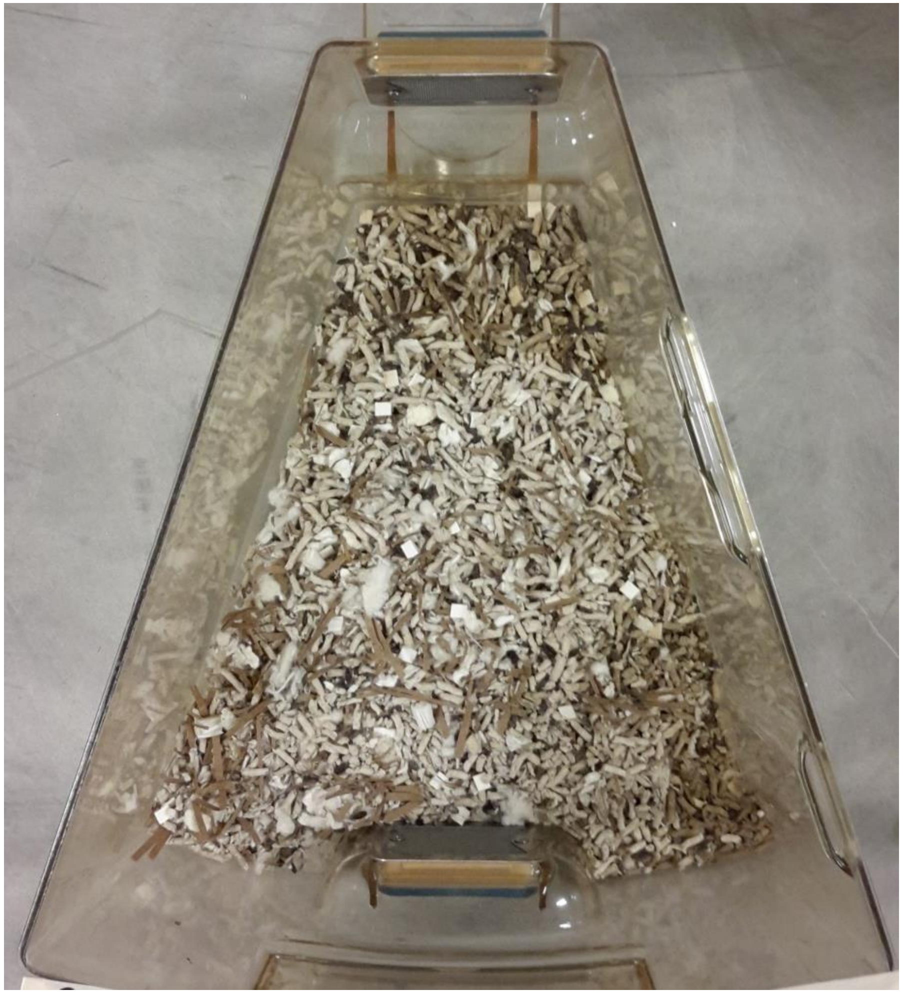

June 16 COMP 1 right

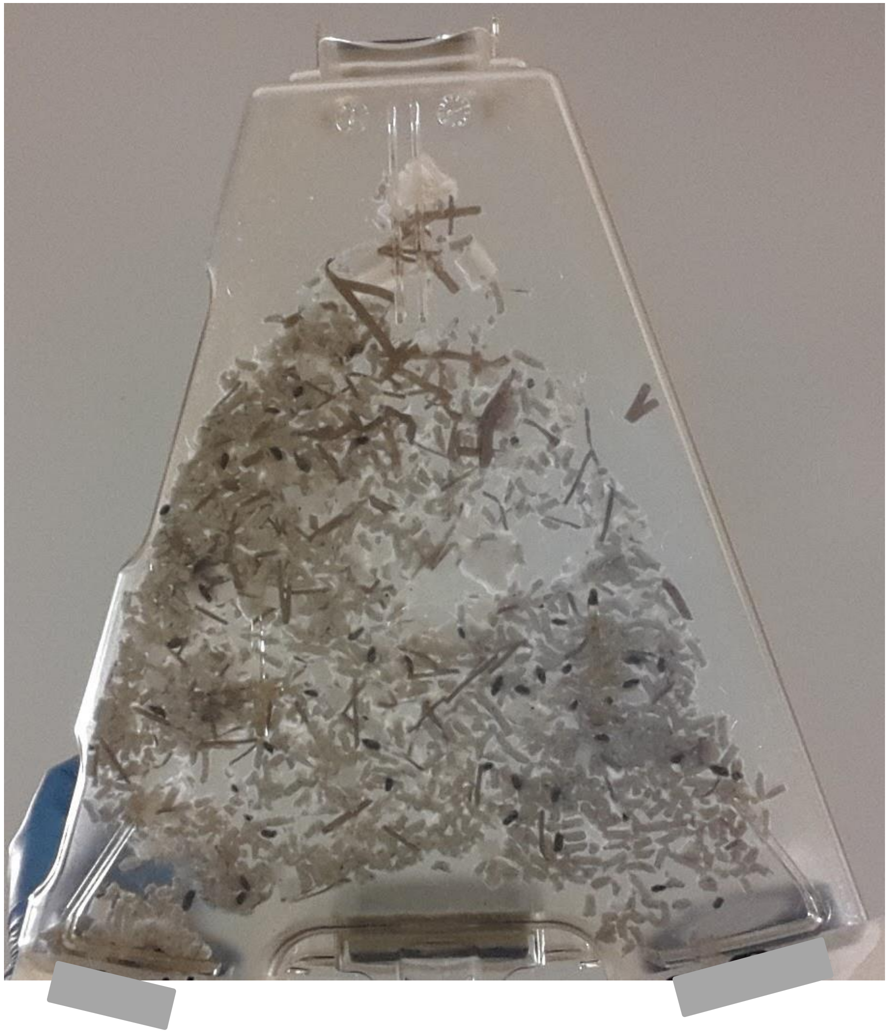

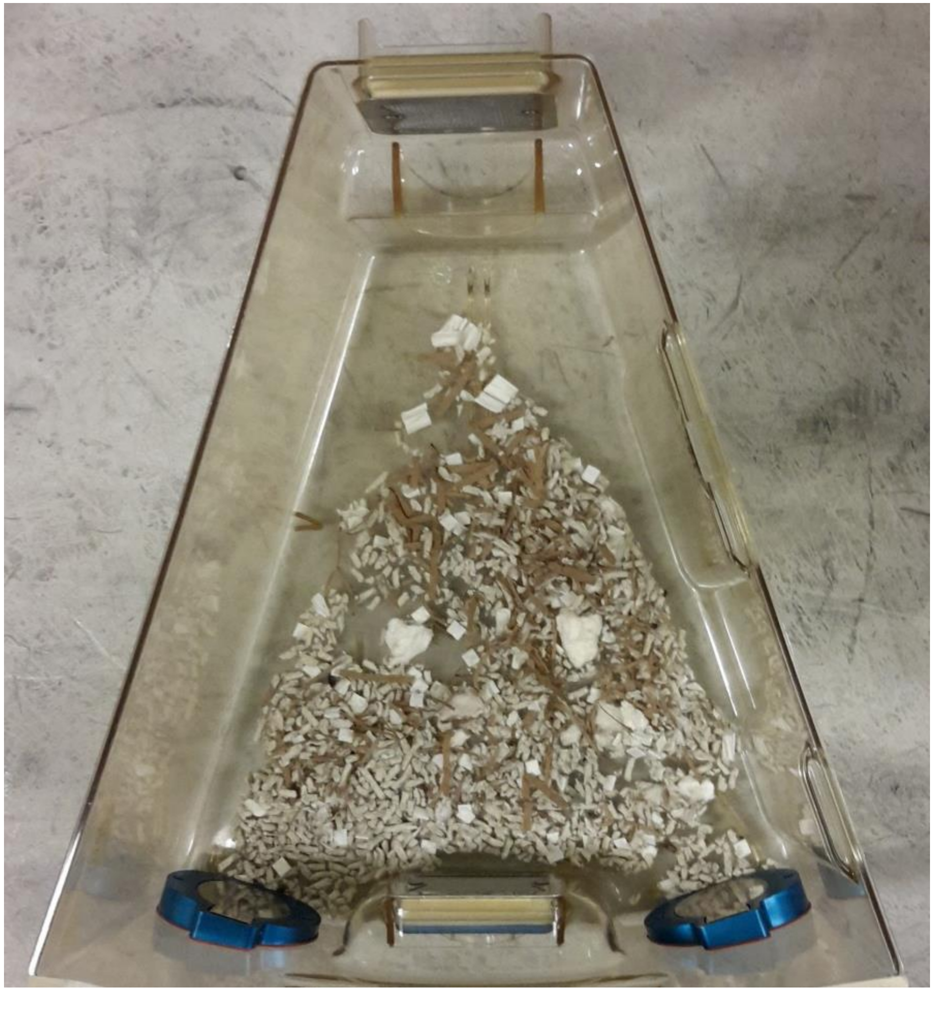

June 16 COMP 1 left

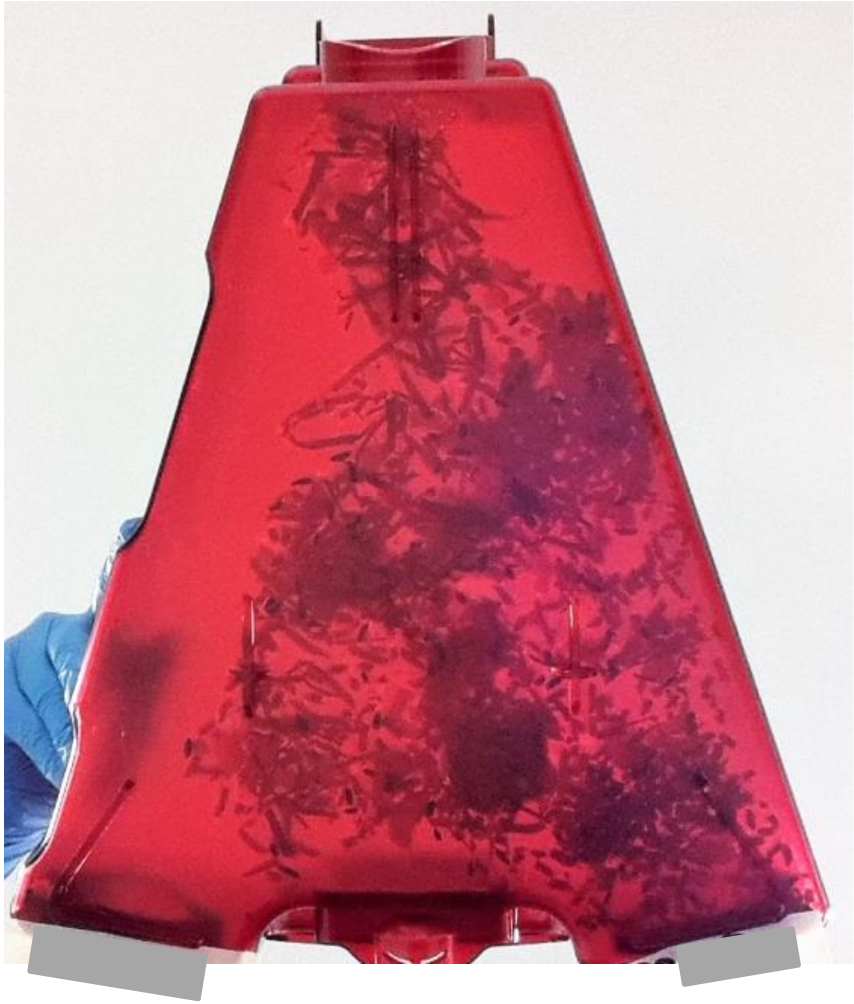

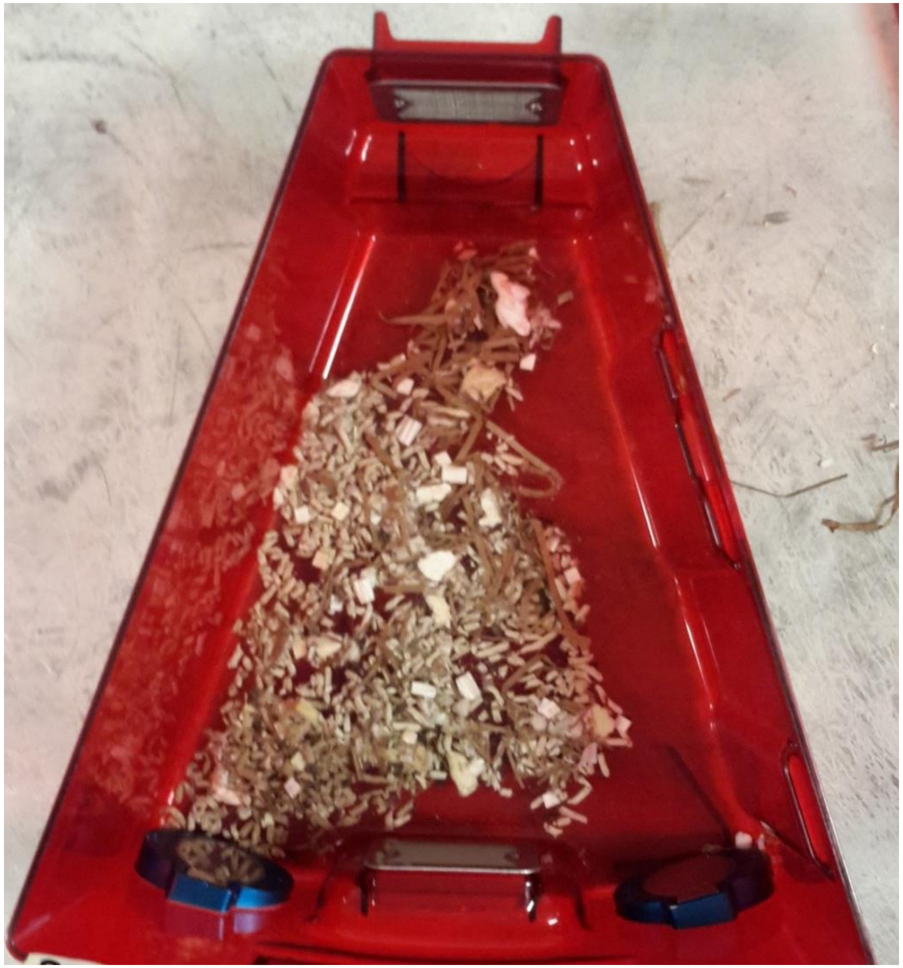

June 16 COMP 1 mid

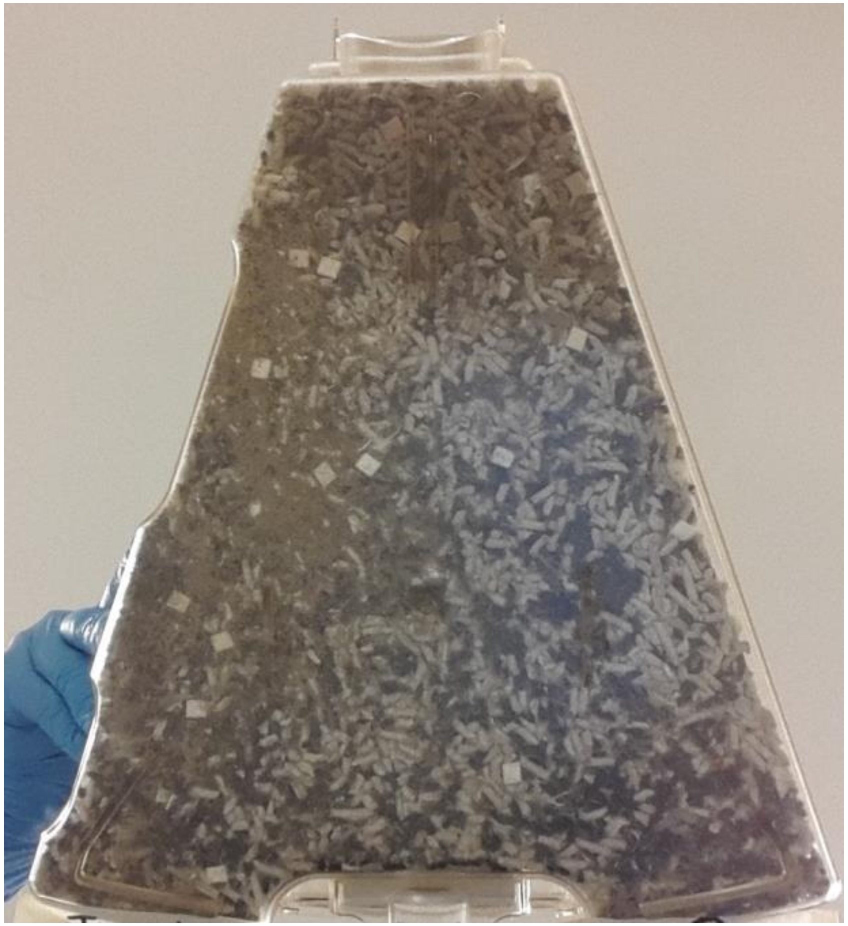

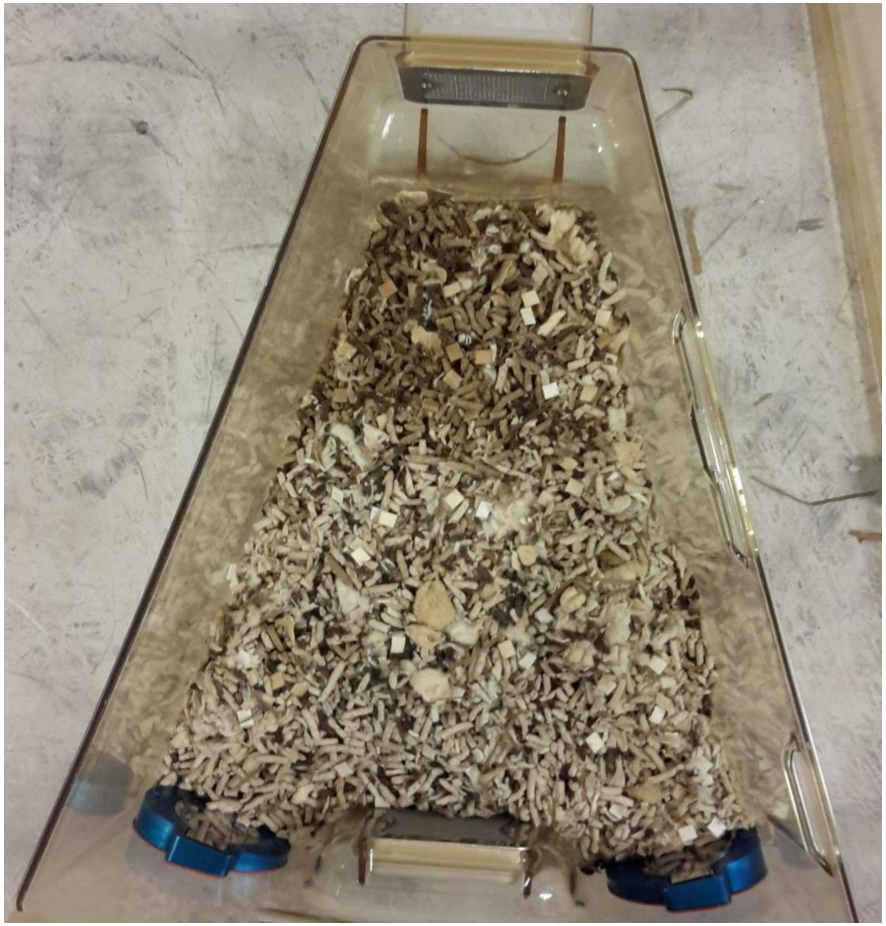

June 16 STD 1

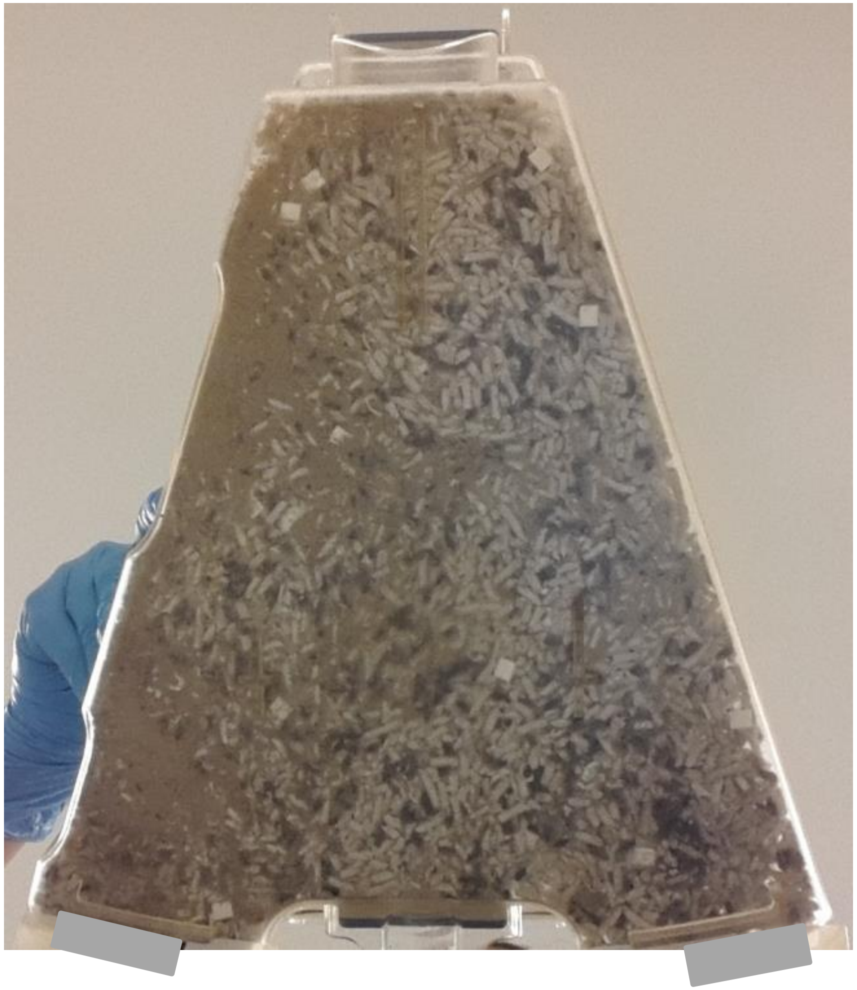

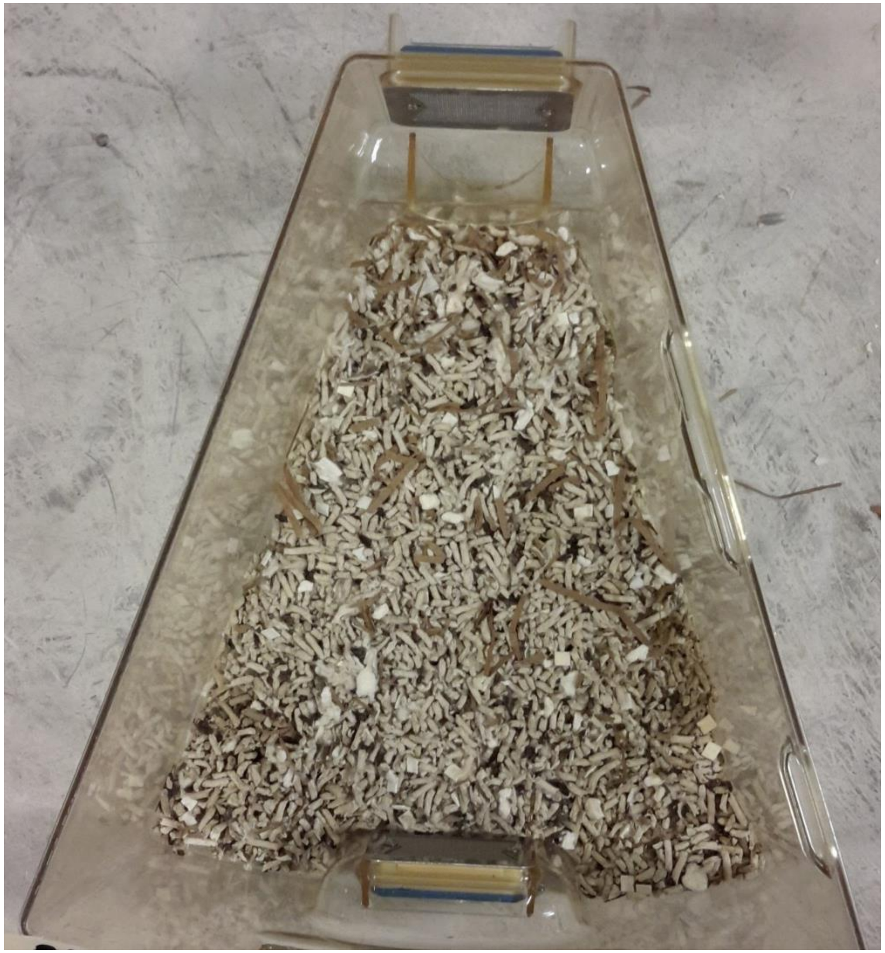

June 16 COMP 2 right

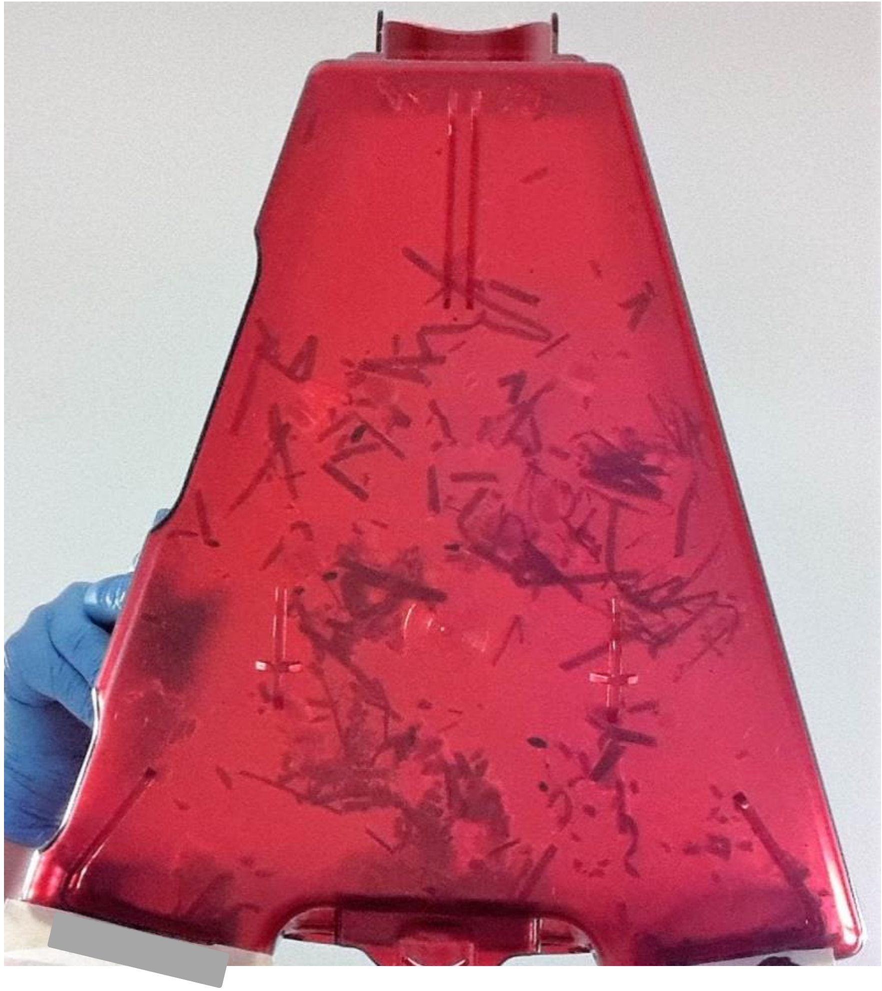

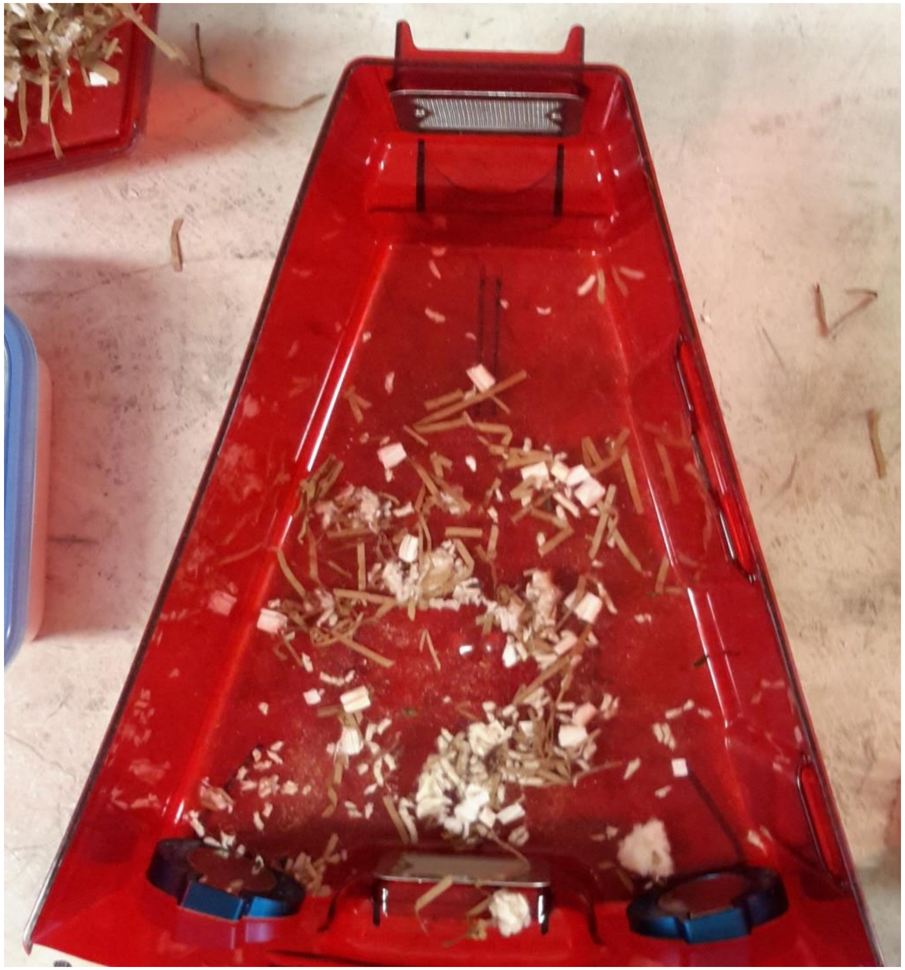

June 16 COMP 2 left

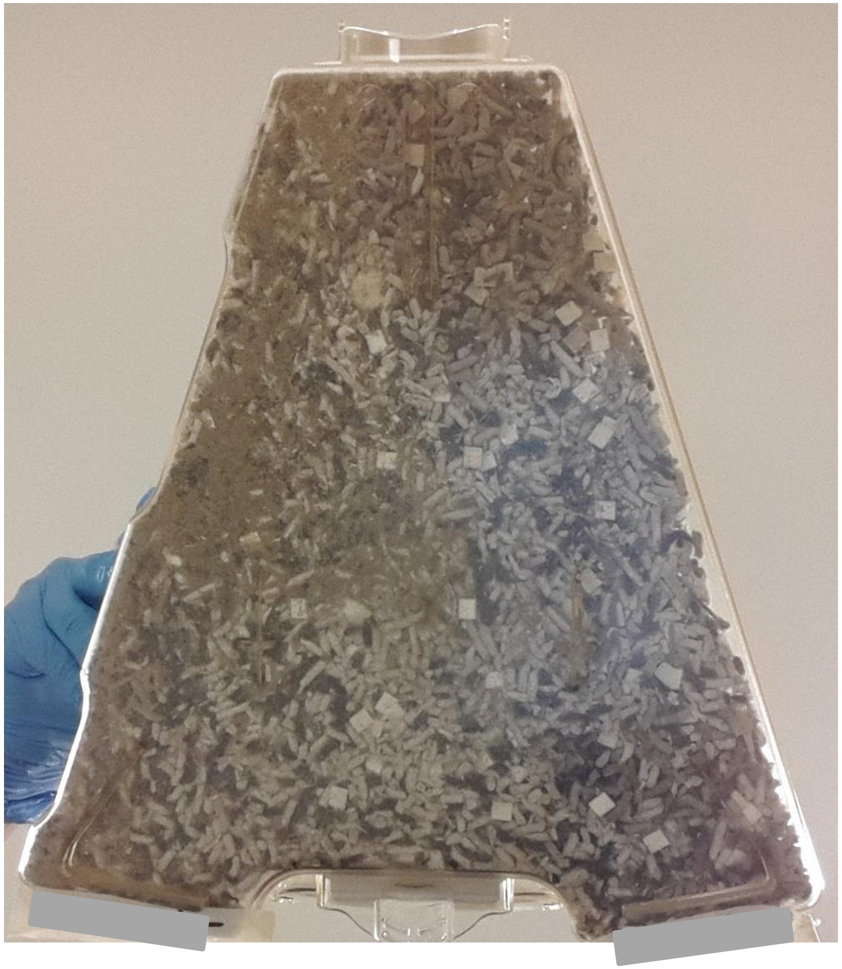

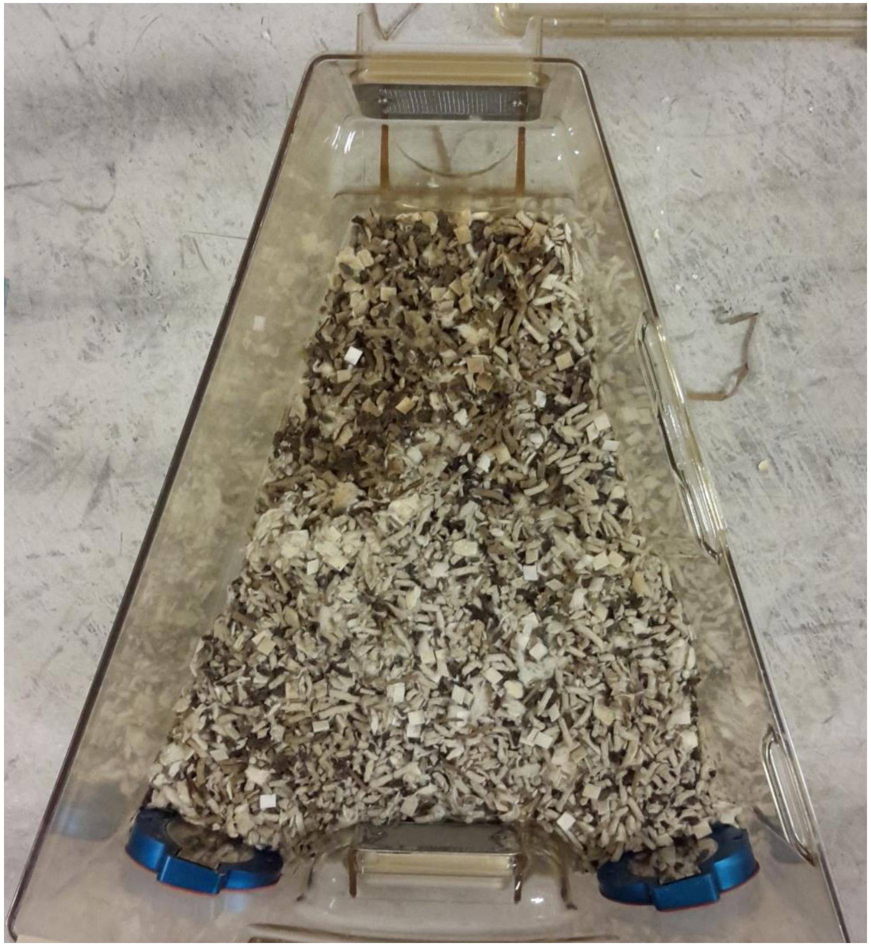

June 16 COMP 2 mid

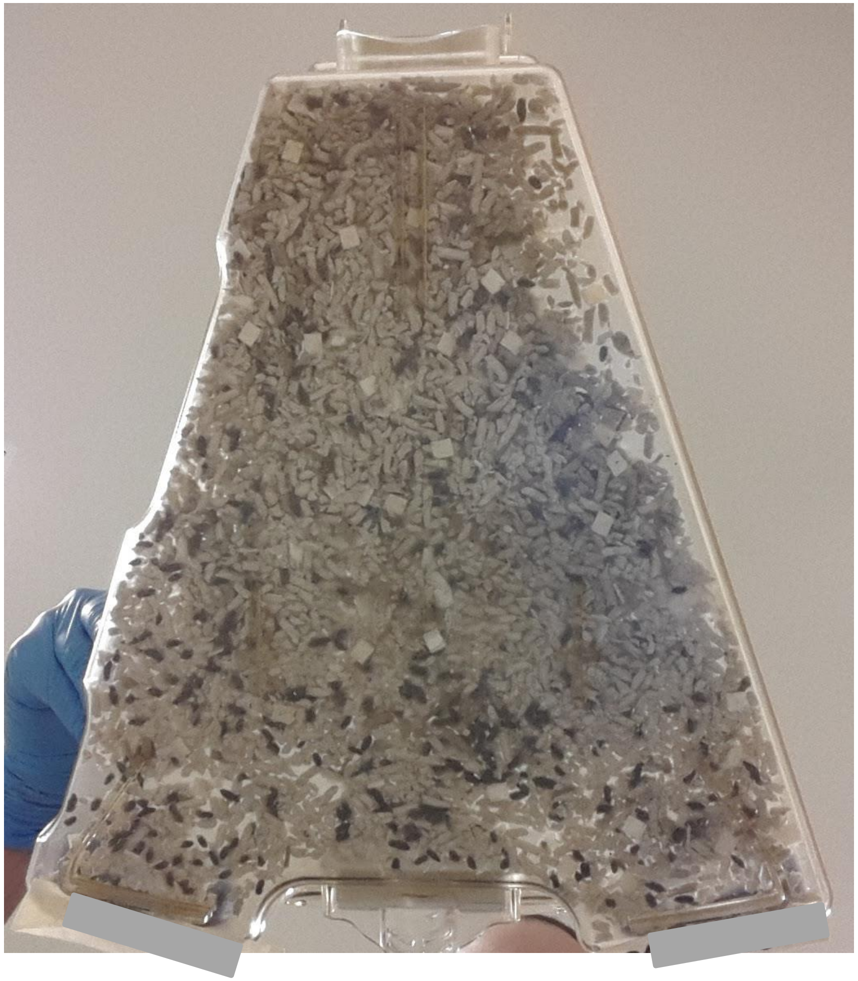

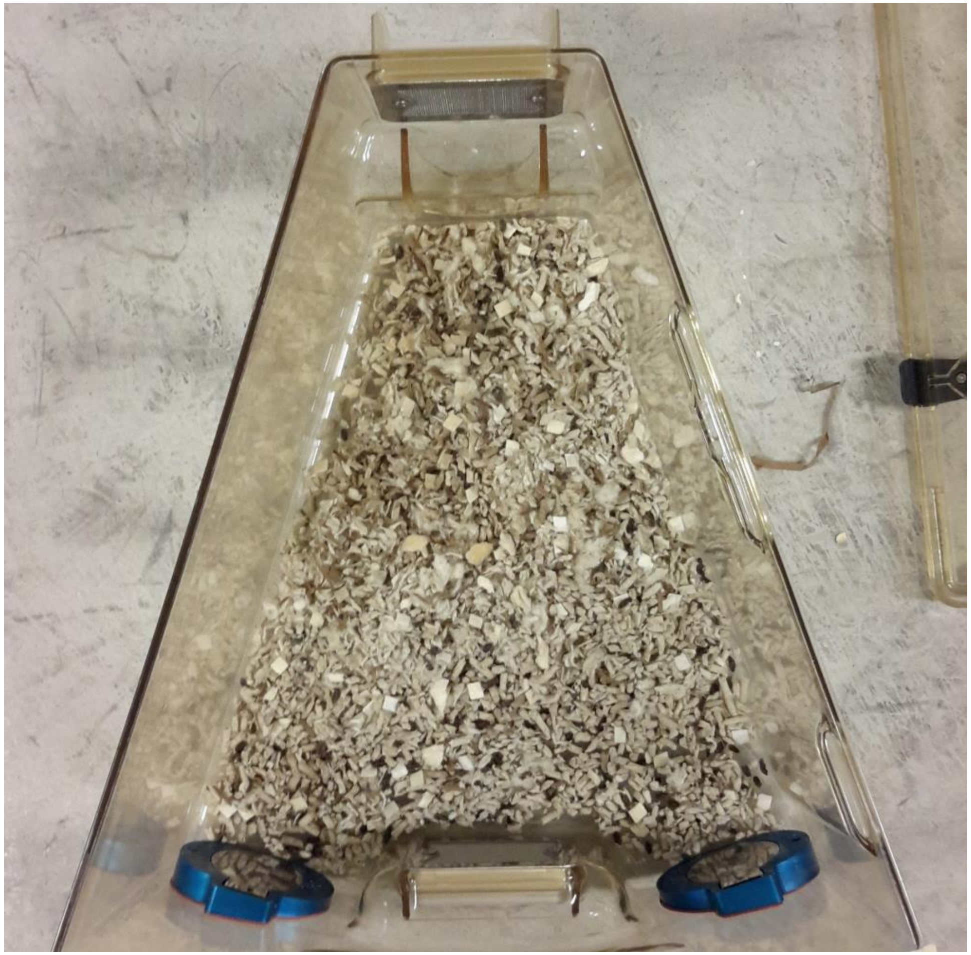

June 16 STD 2

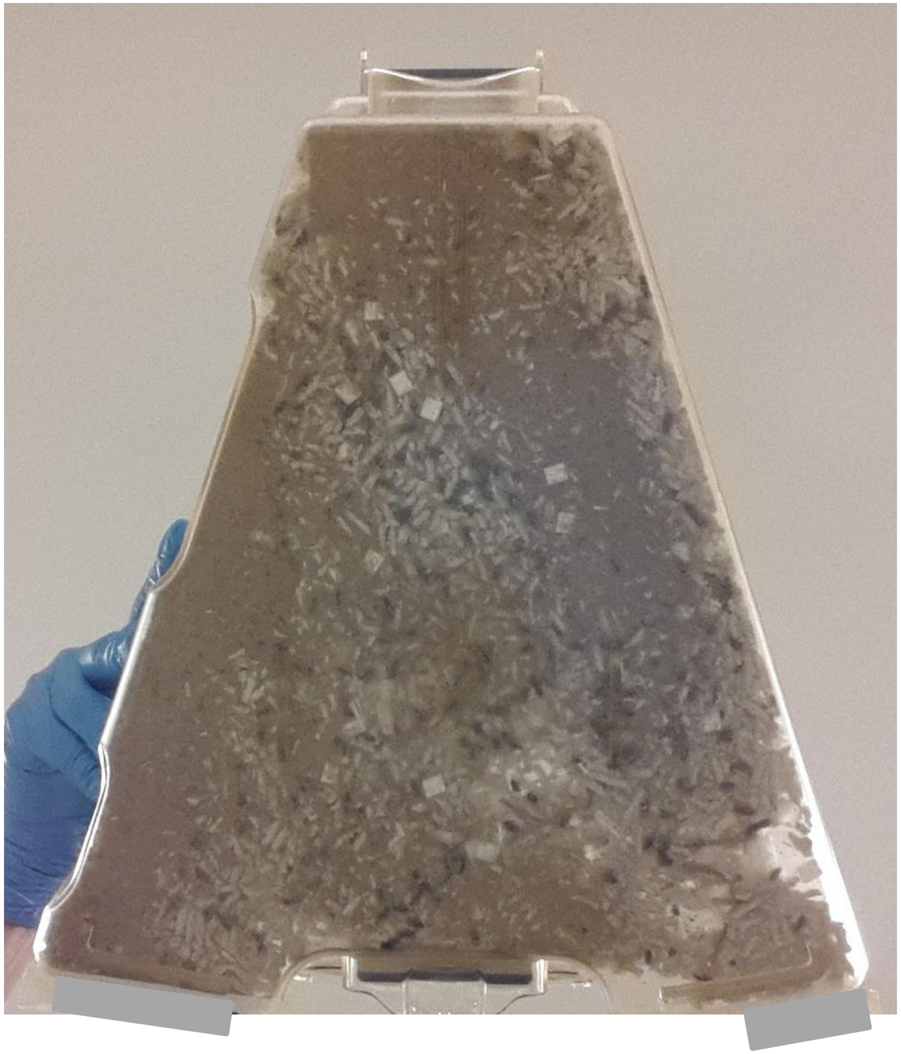

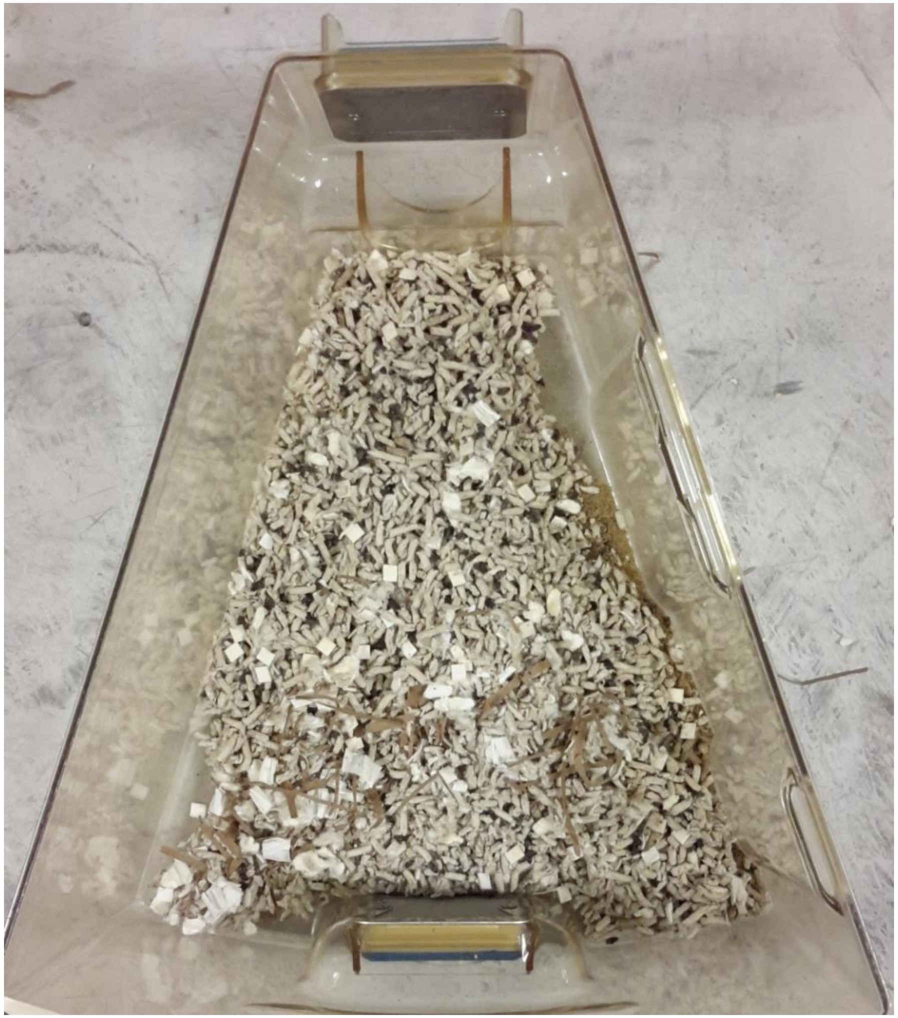

June 17 COMP 3 left

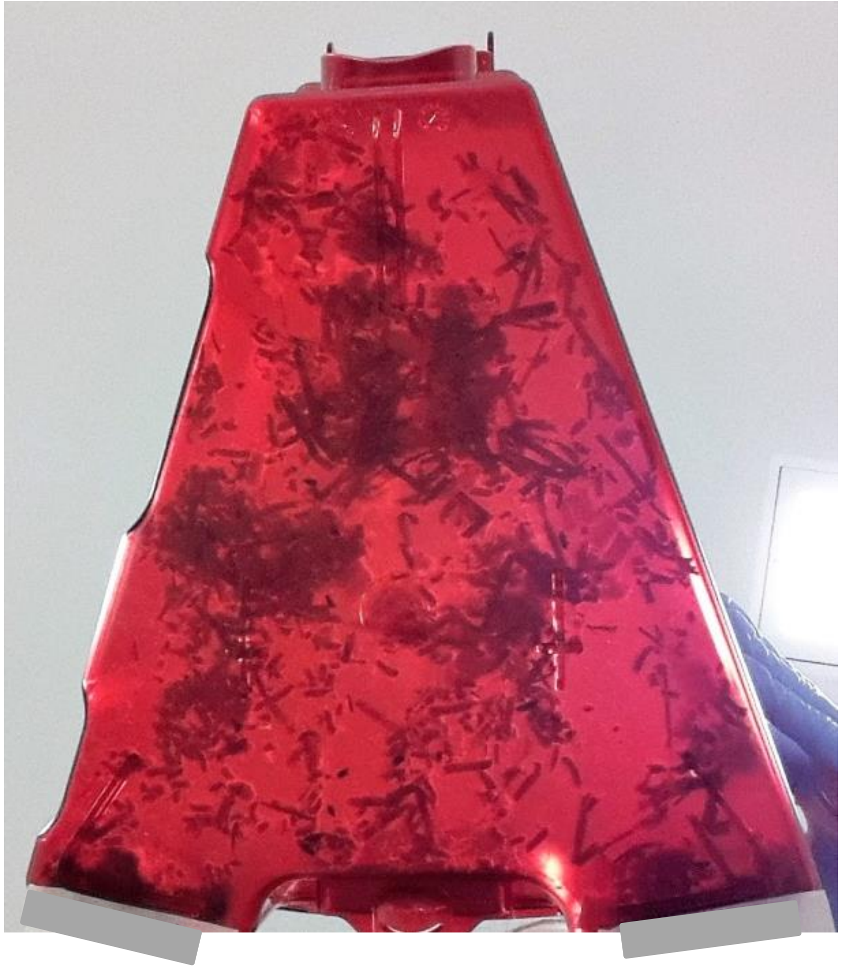

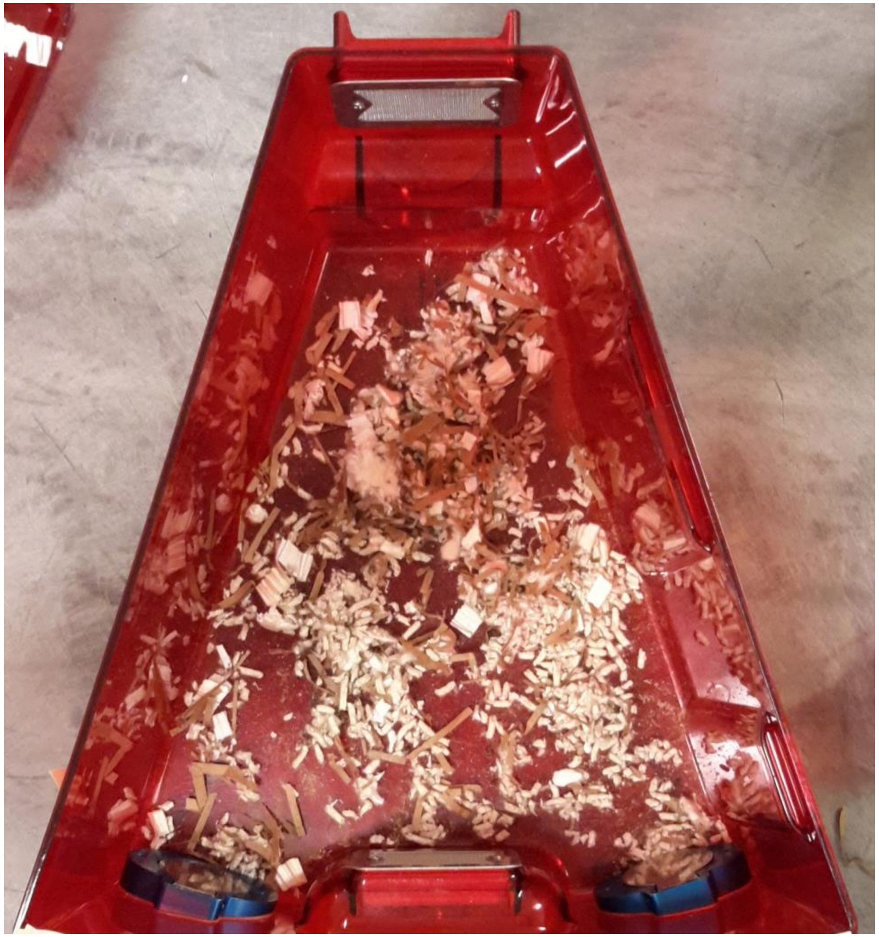

June 17 COMP 3 right

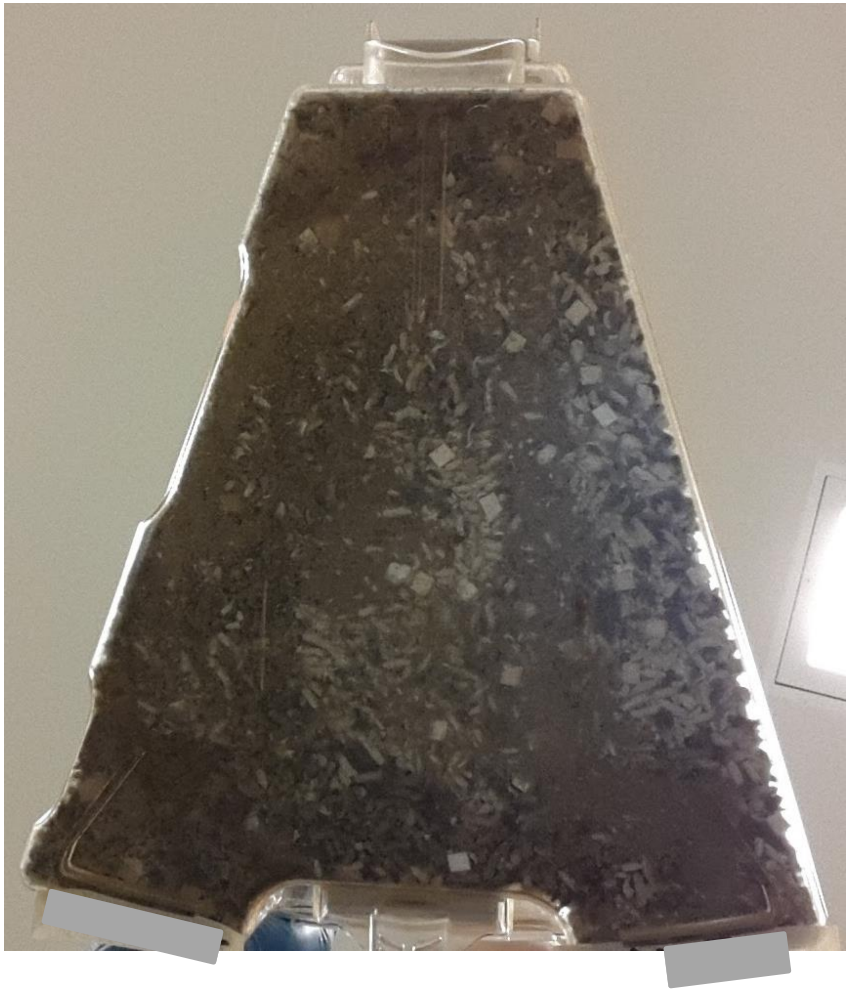

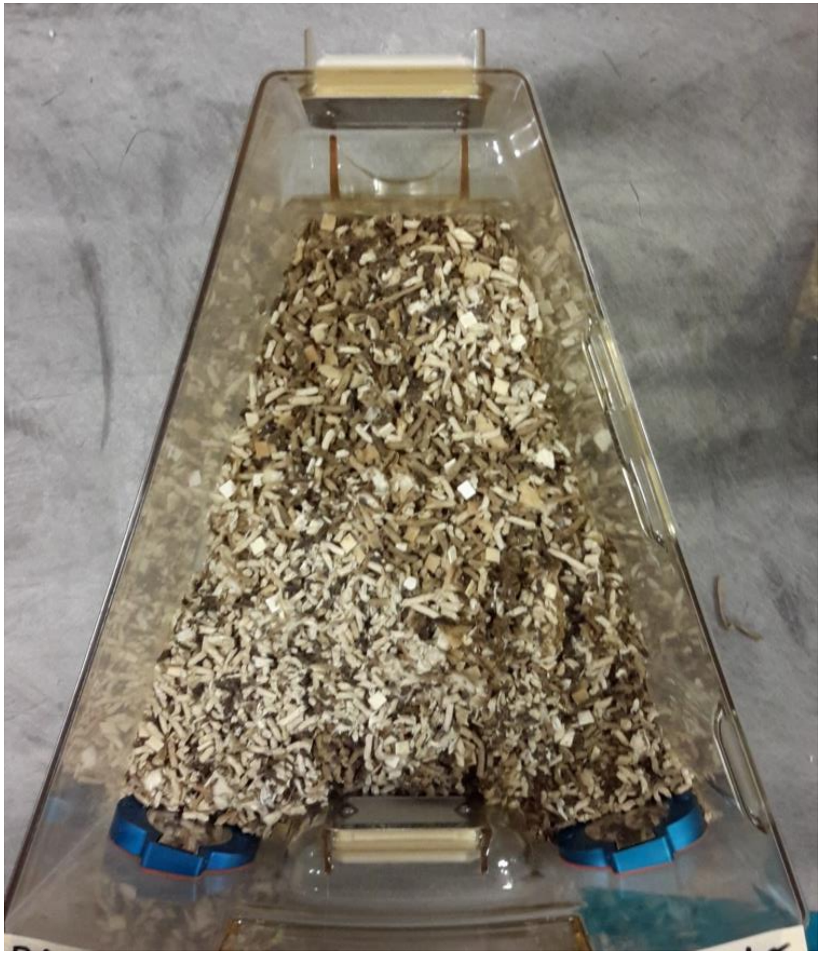

June 17 COMP 3 mid

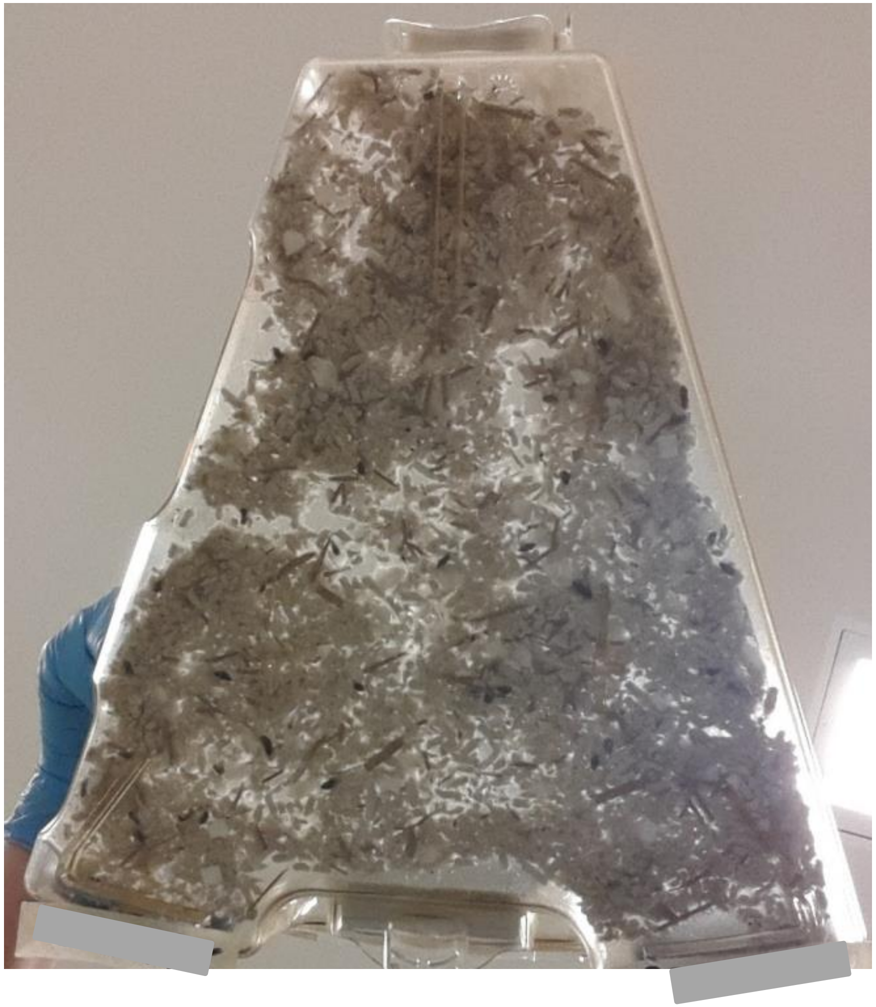

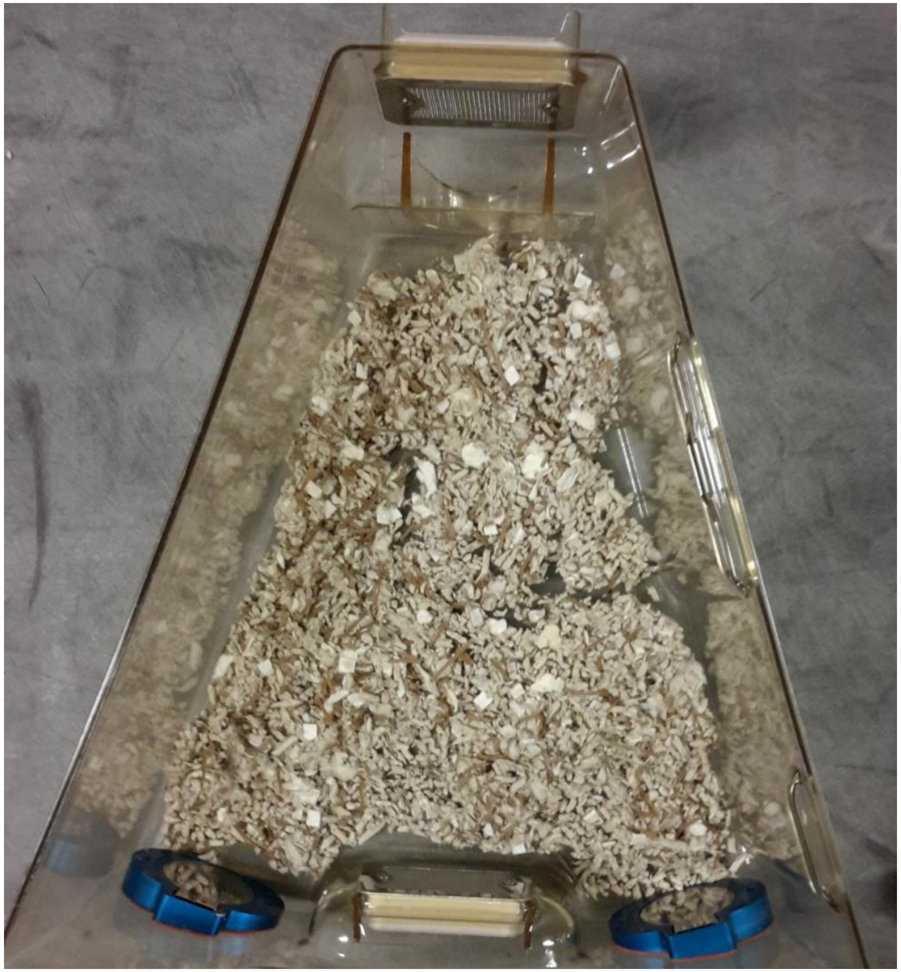

June 17 STD 3

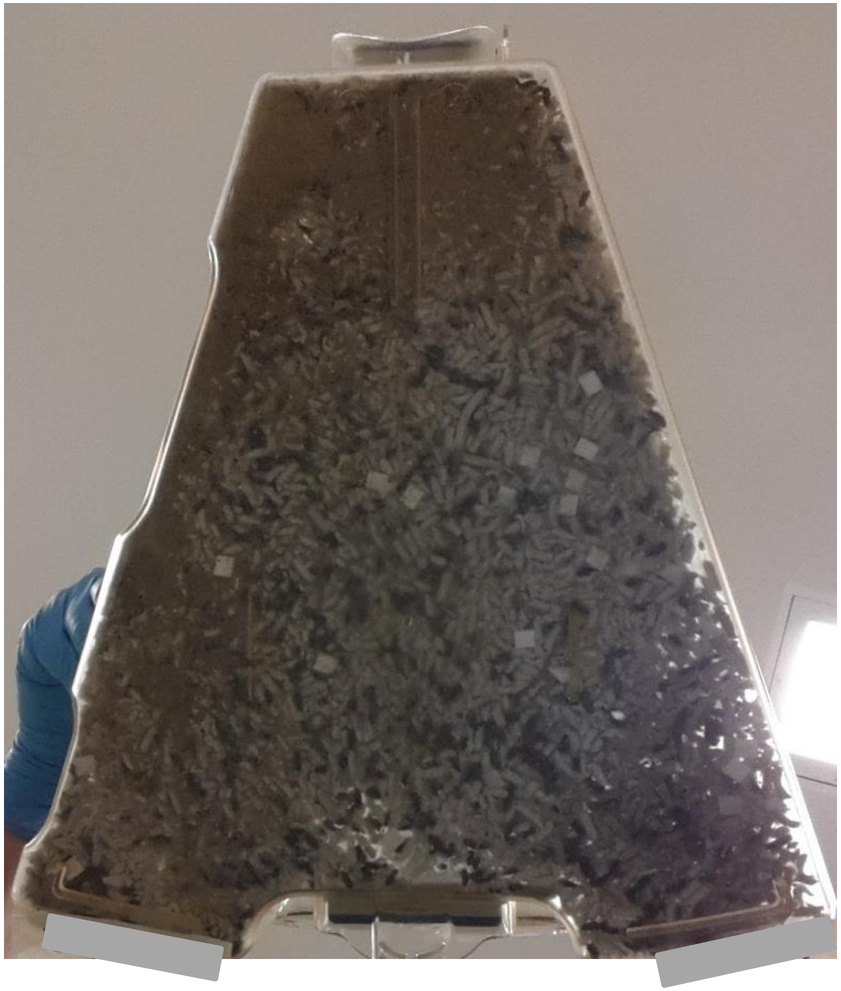

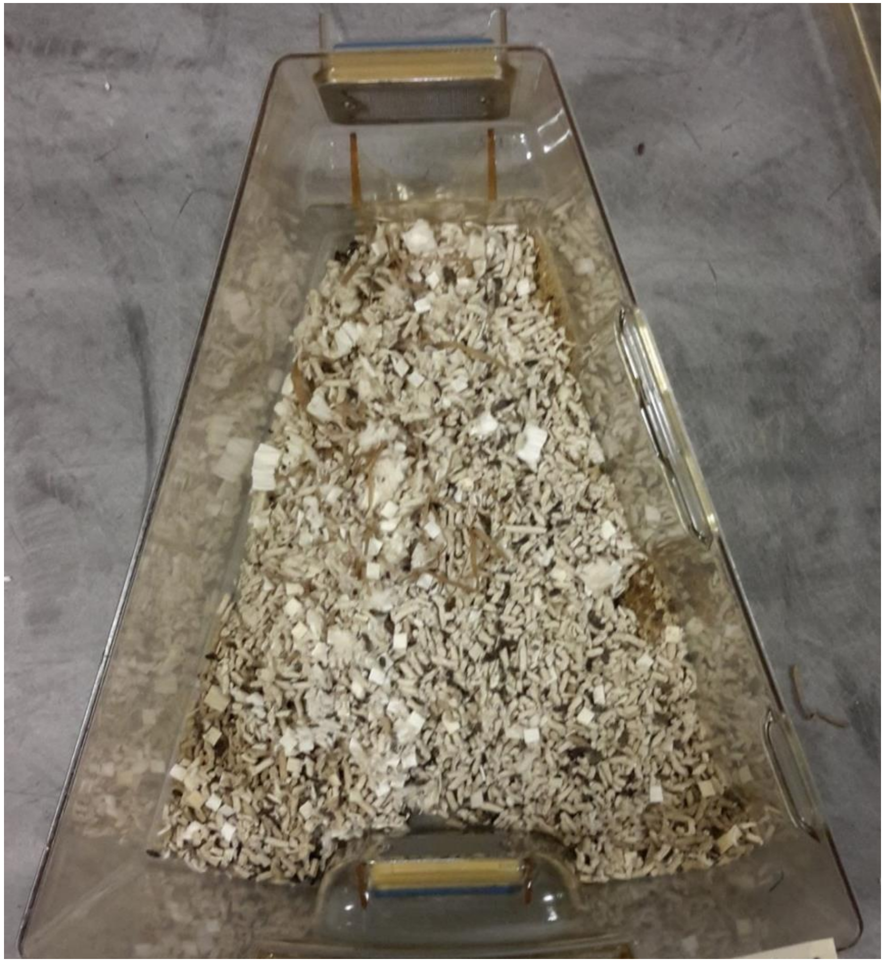

June 17 COMP 4 mid

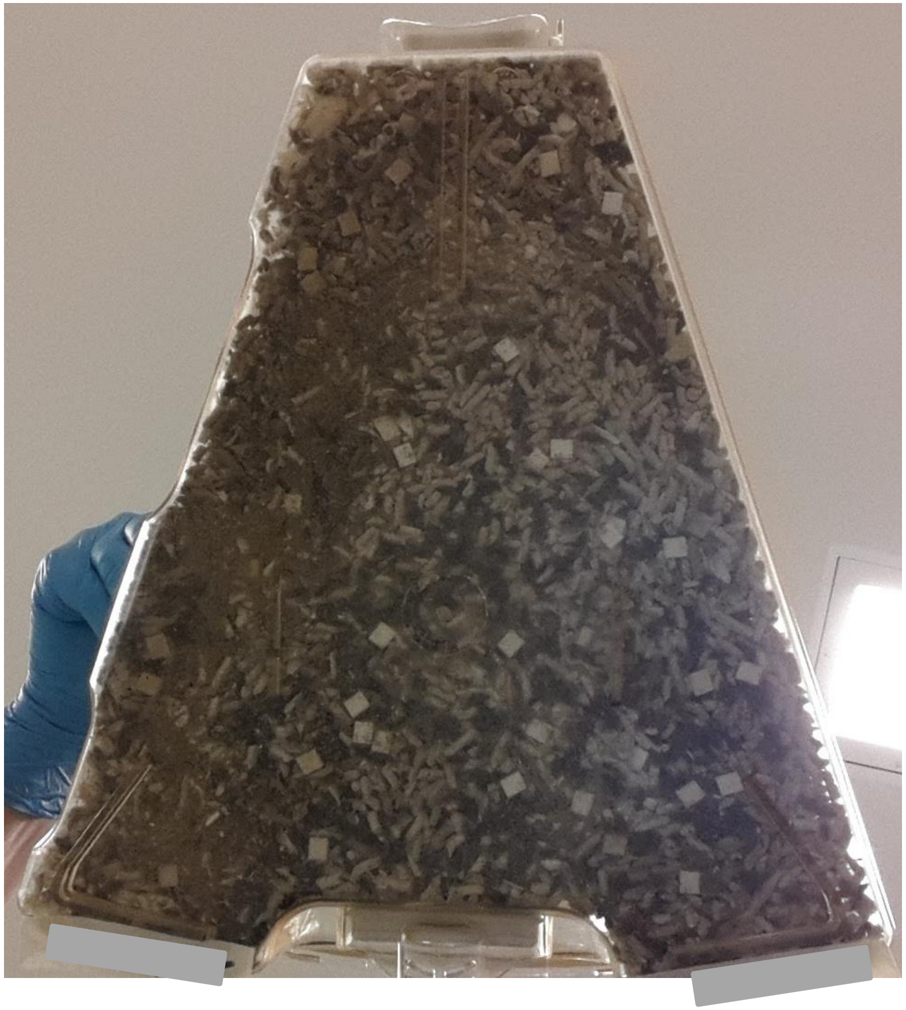

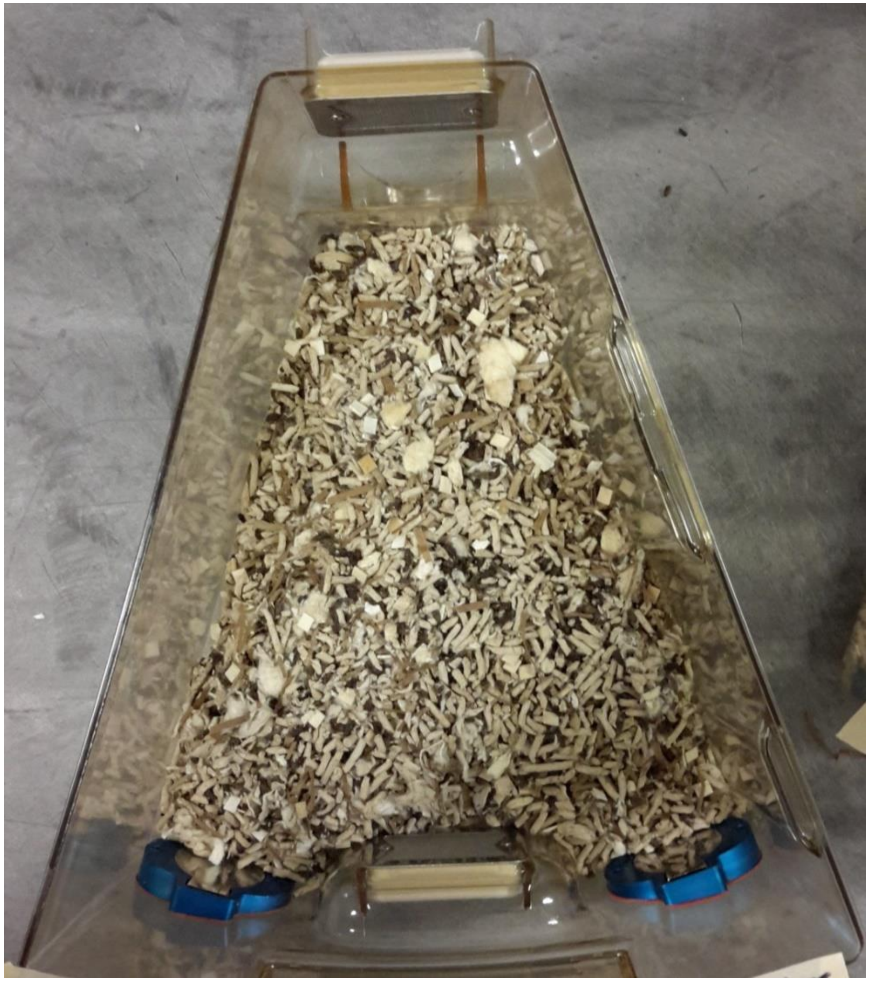

June 17 COMP 4 left

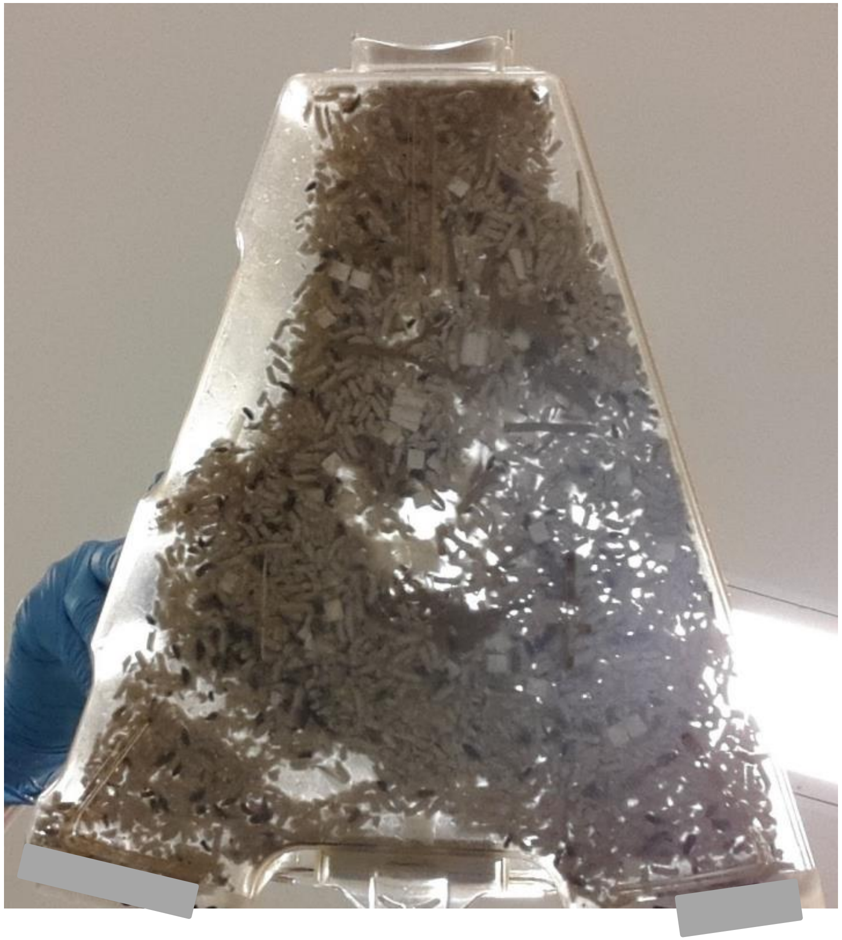

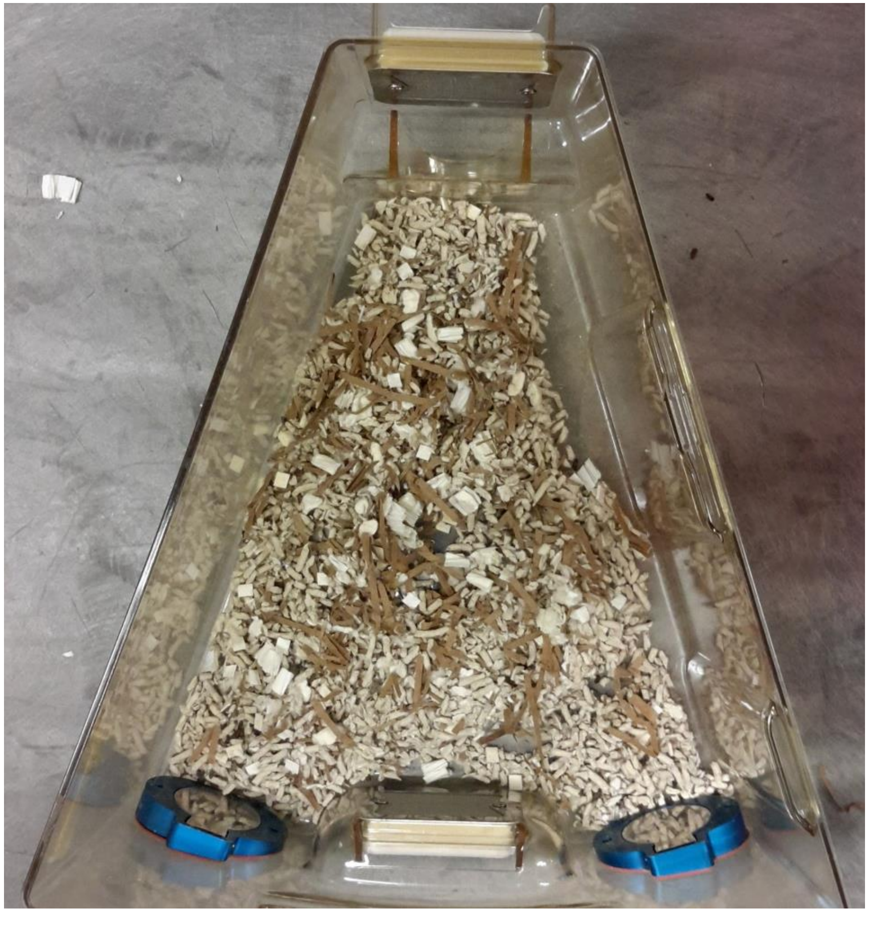

June 17 COMP 4 right

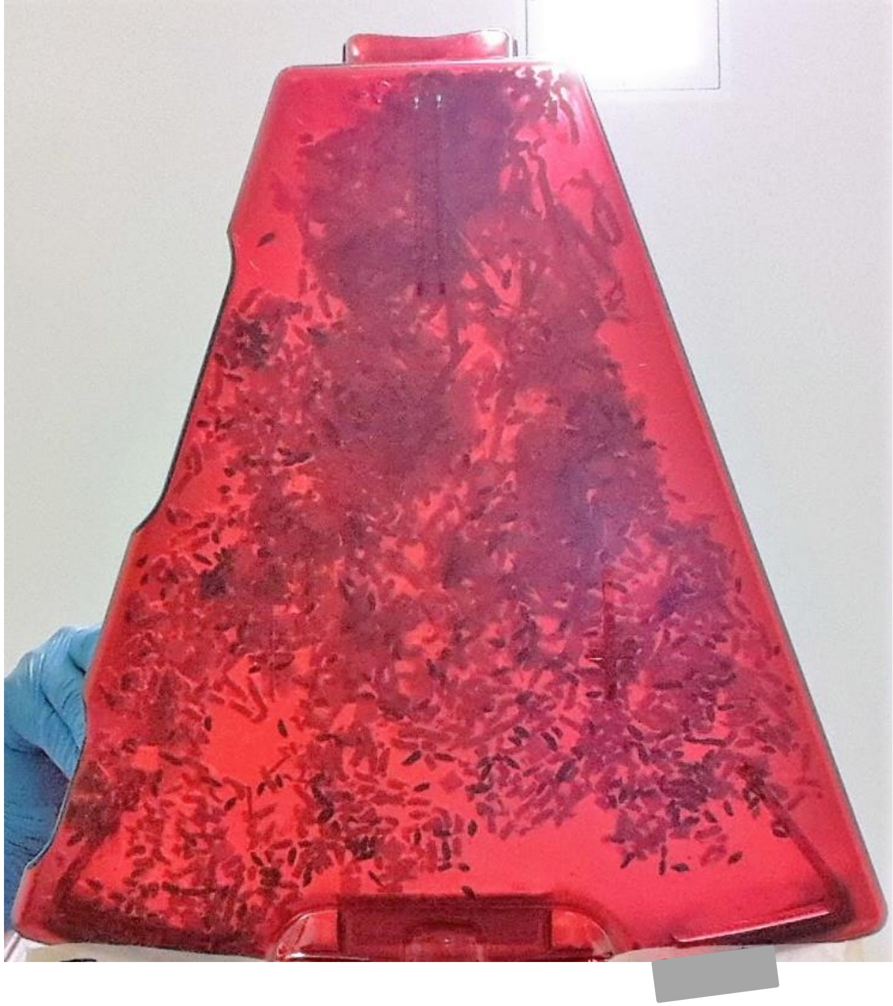

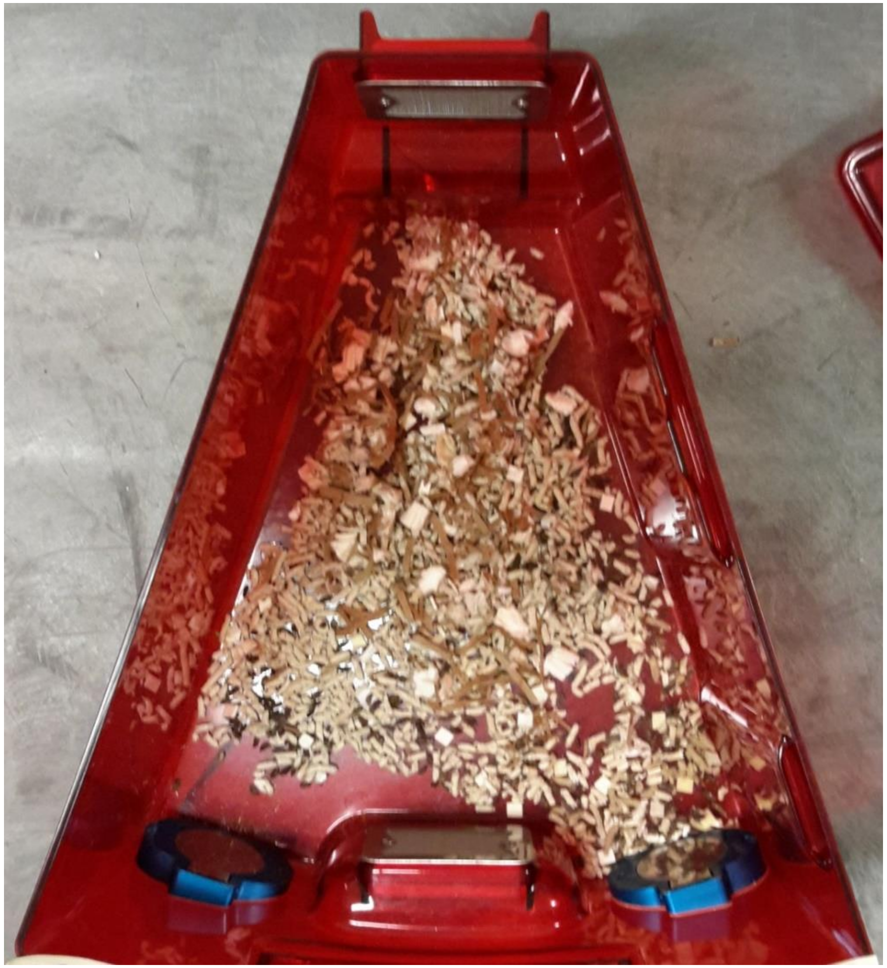

June 17 STD 4

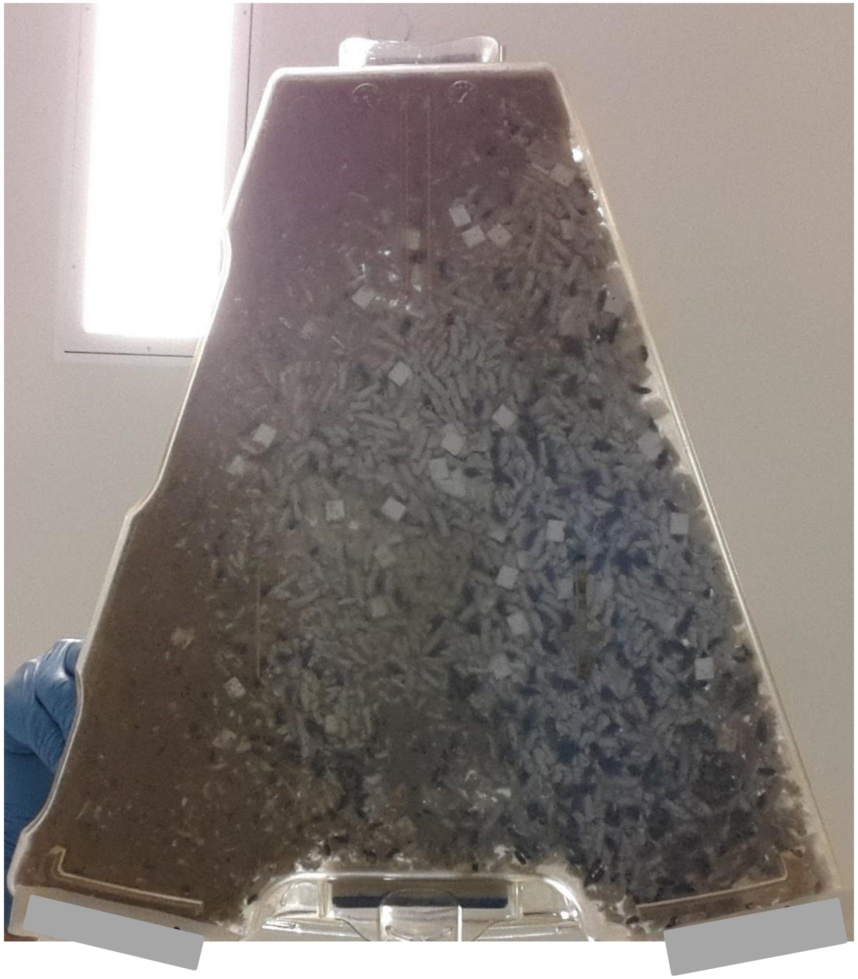
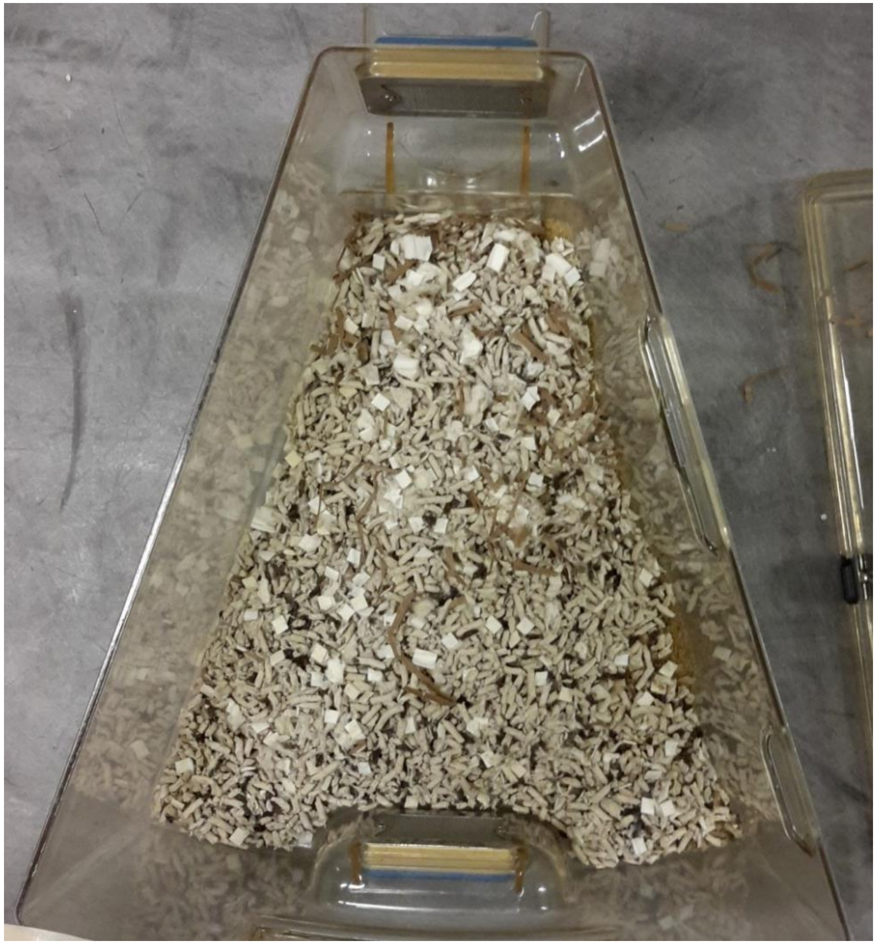

June 18 COMP 5 right

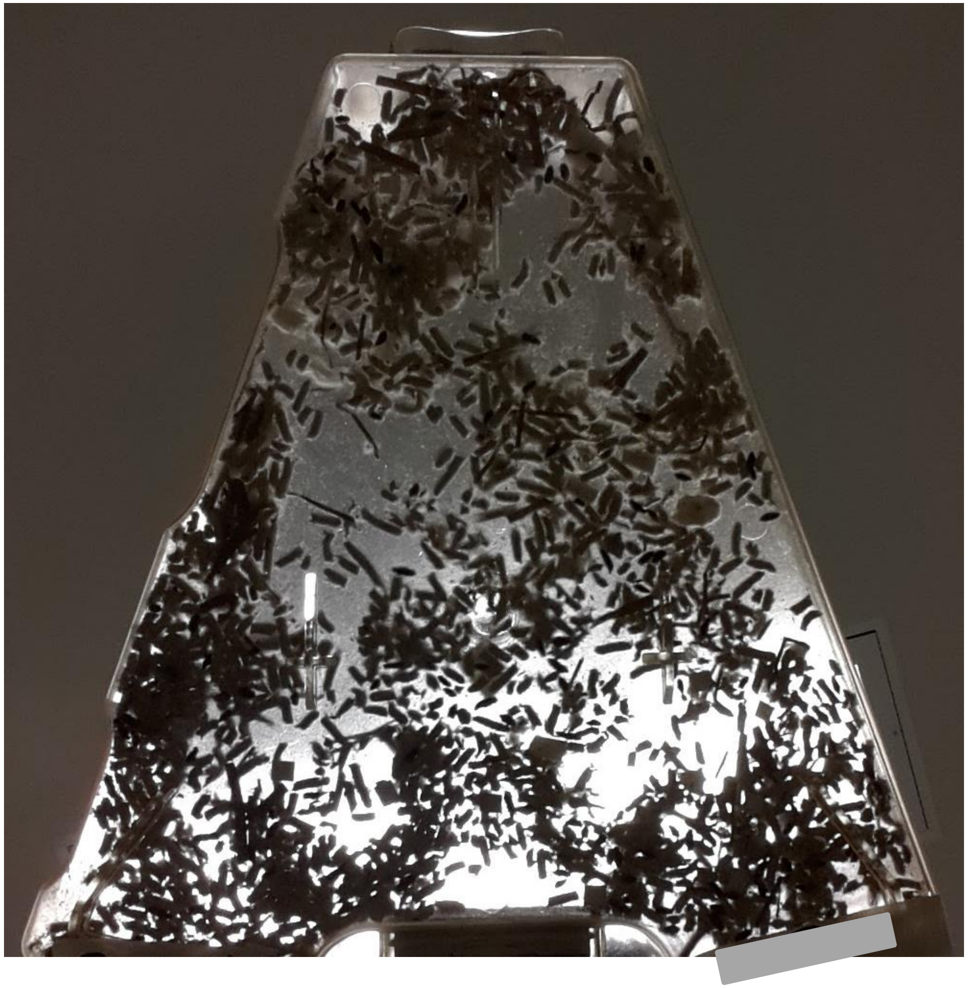

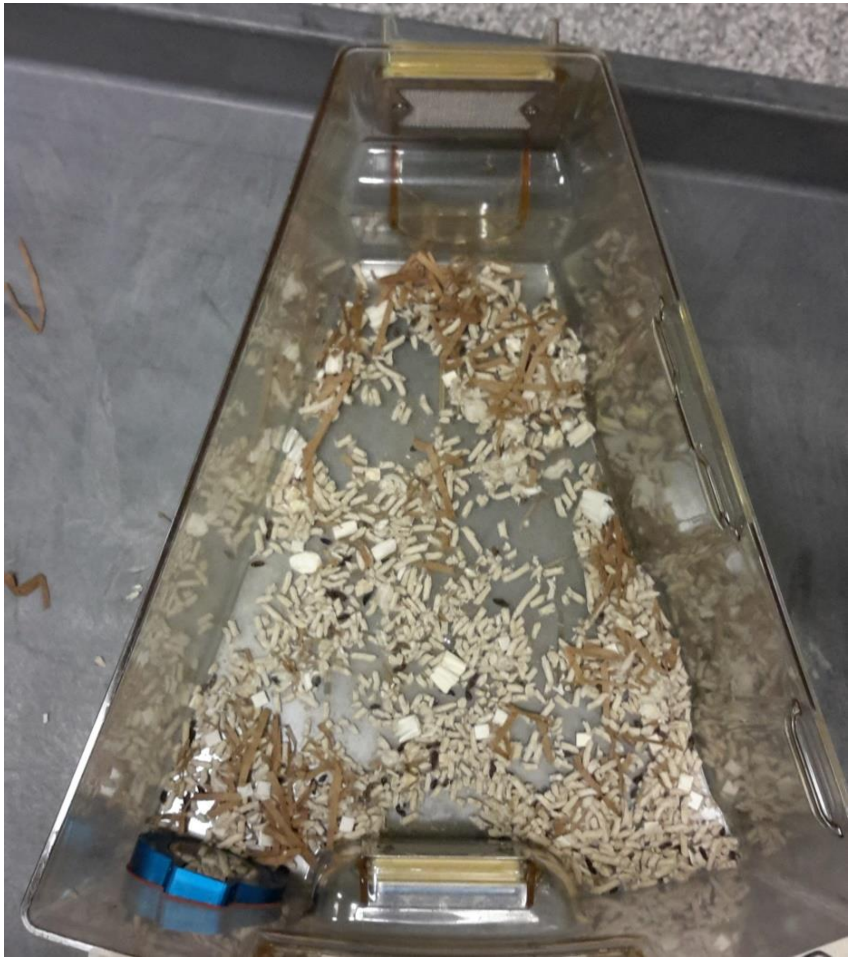

June 18 COMP 5 mid

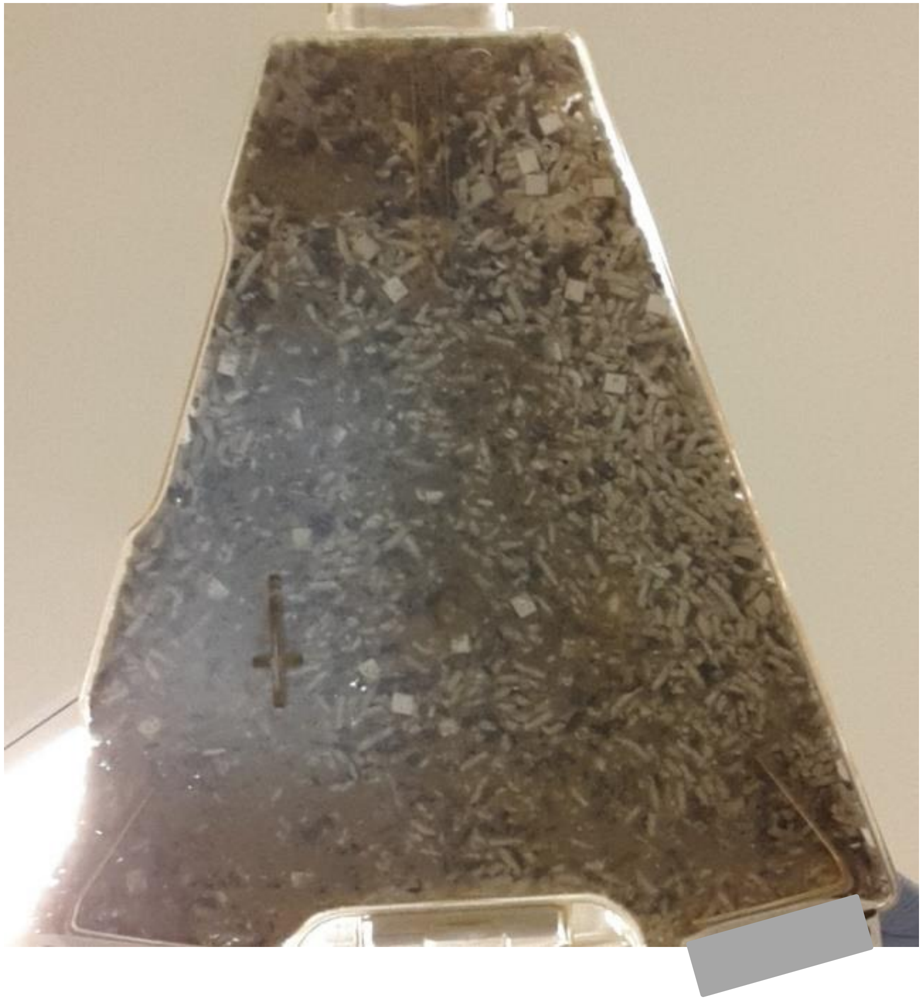

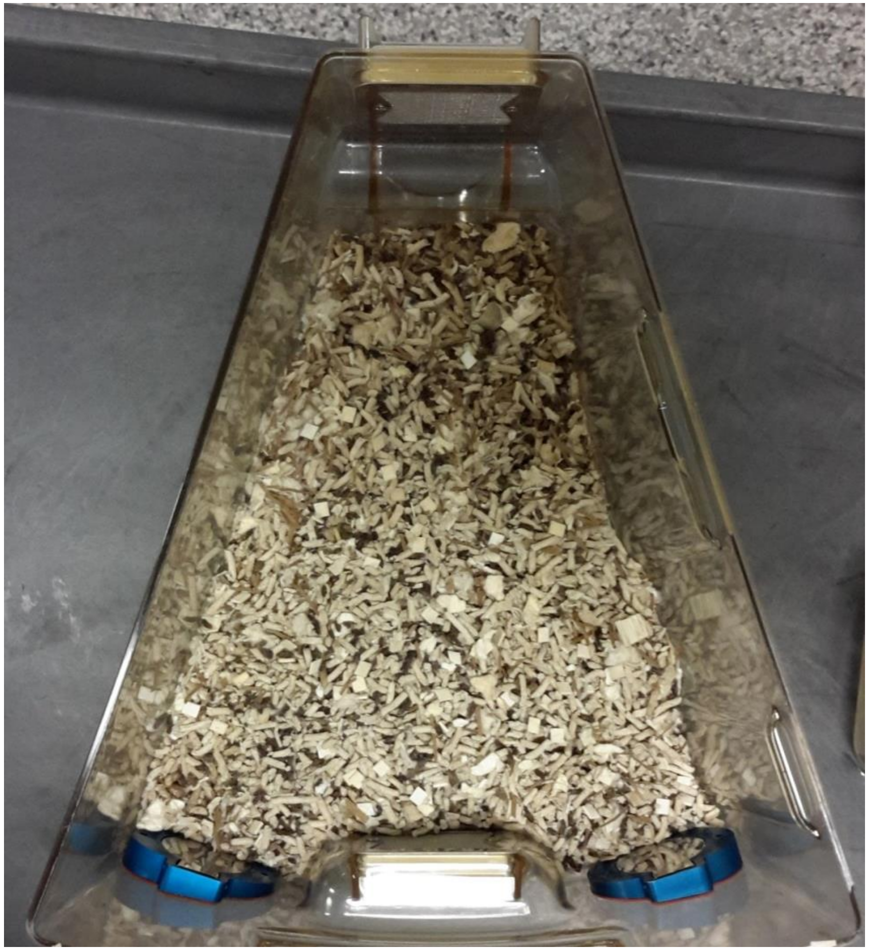

June 18 COMP 5 left

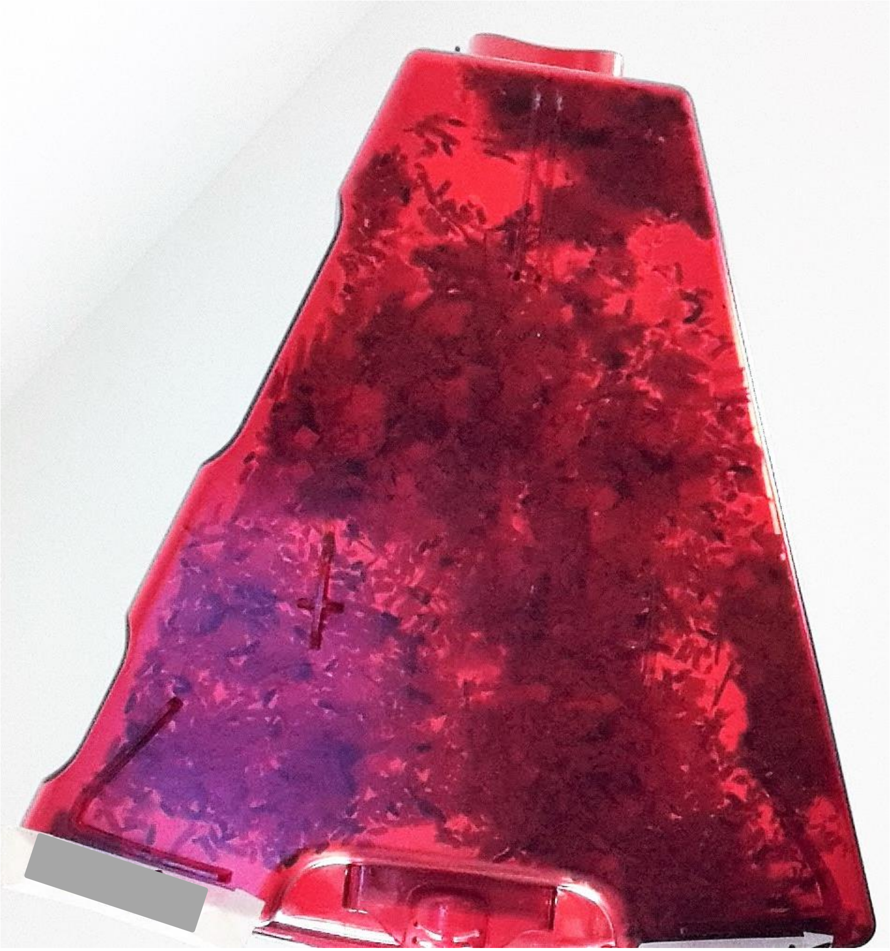

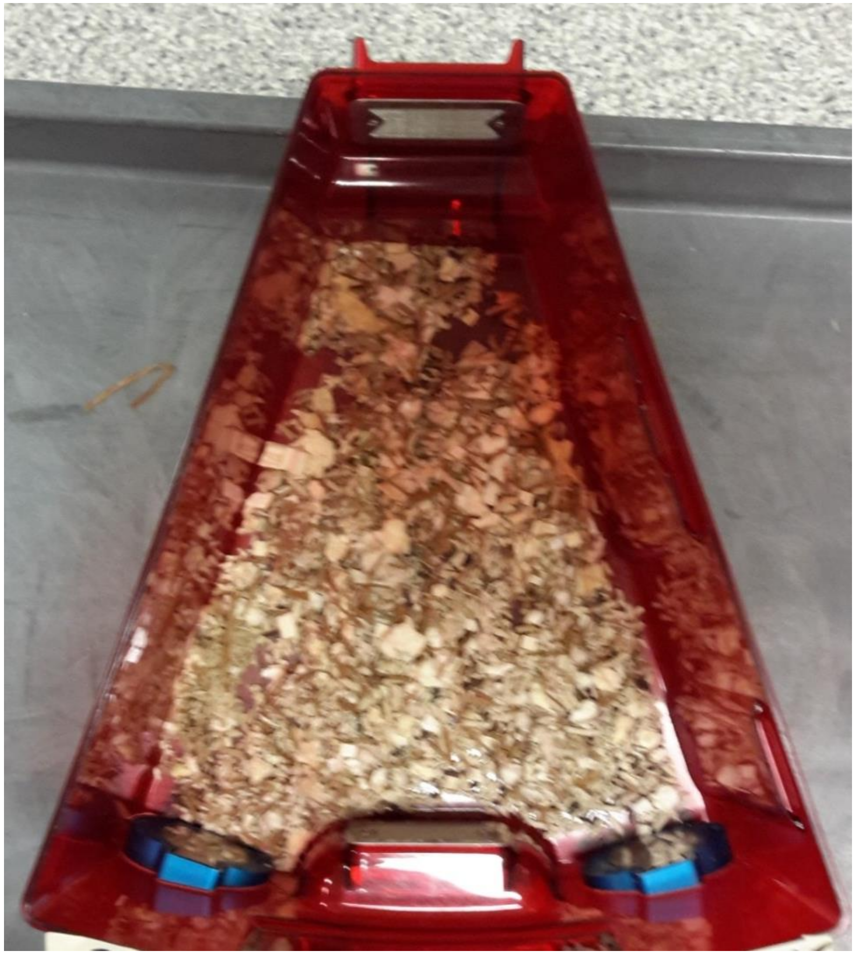

June 18 STD 5

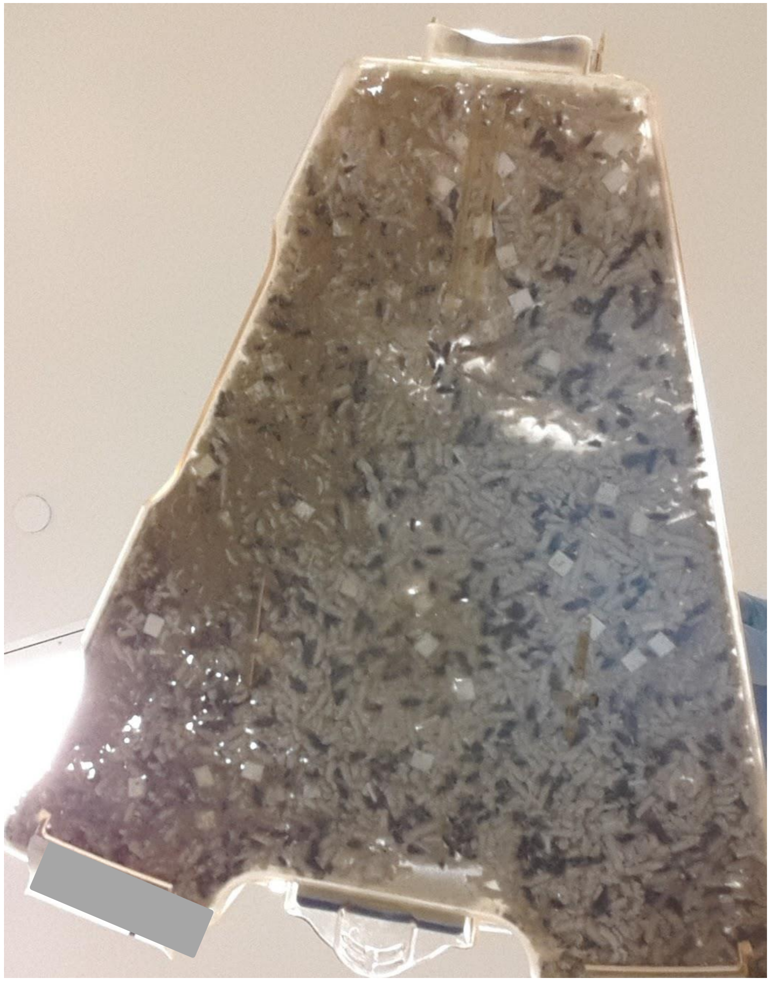

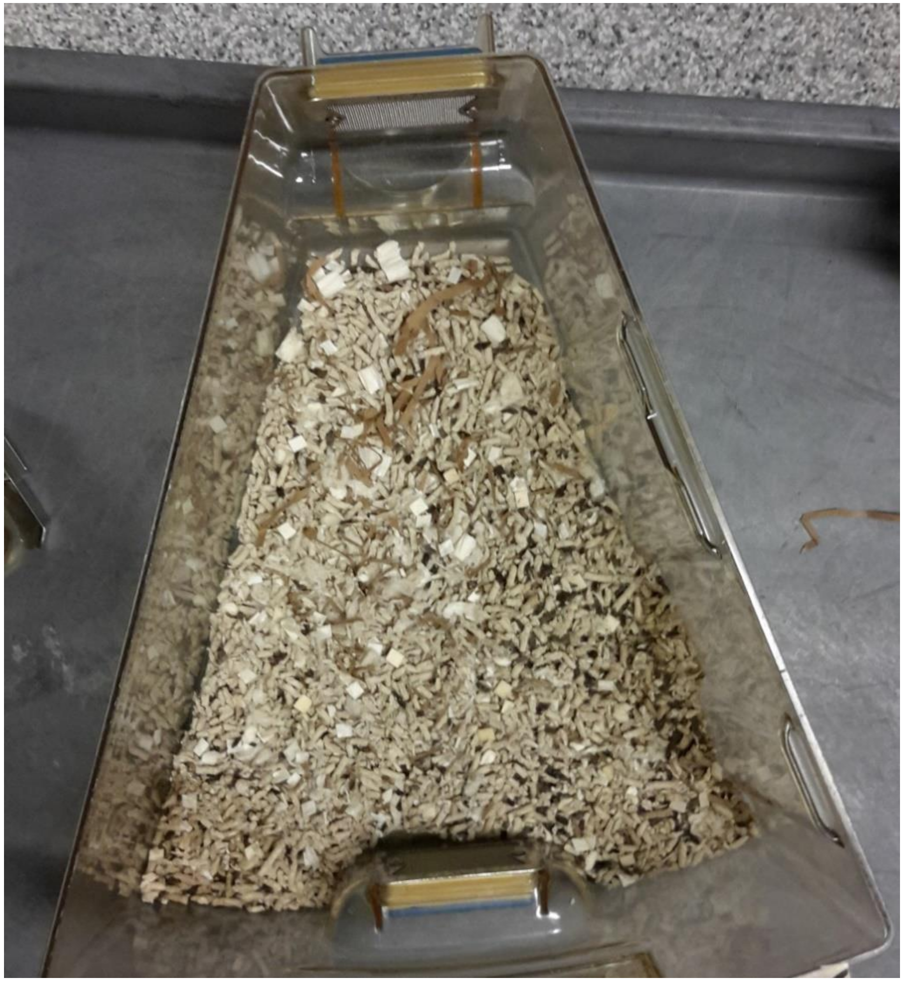

June 23 COMP 1 right

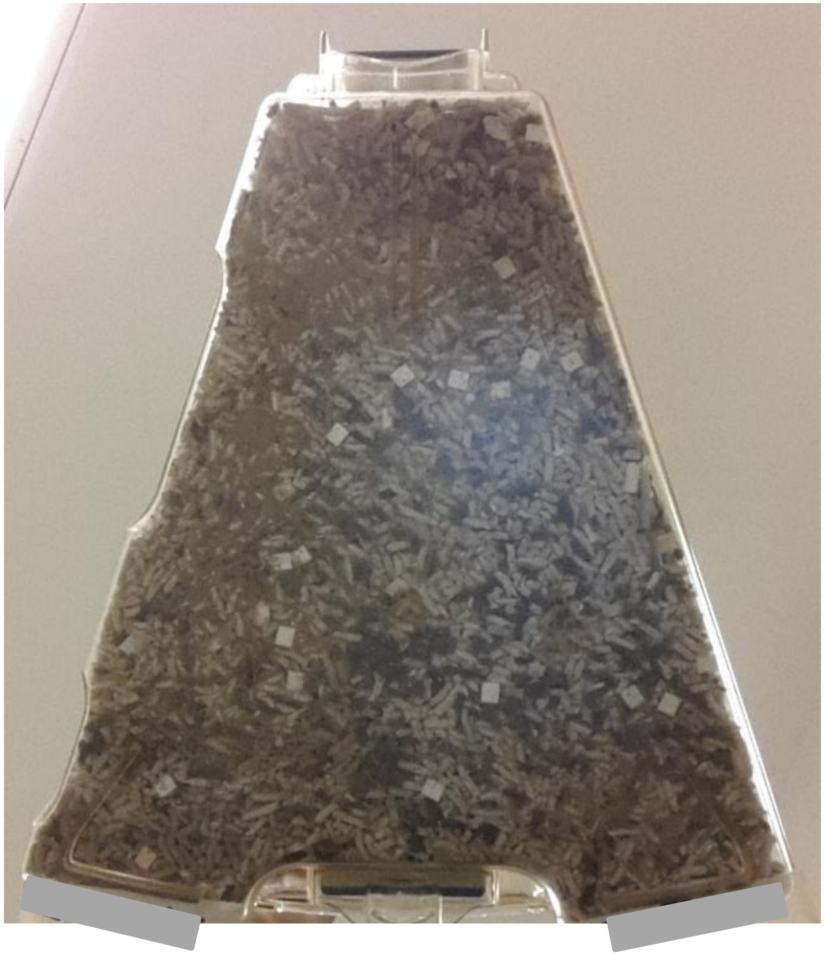

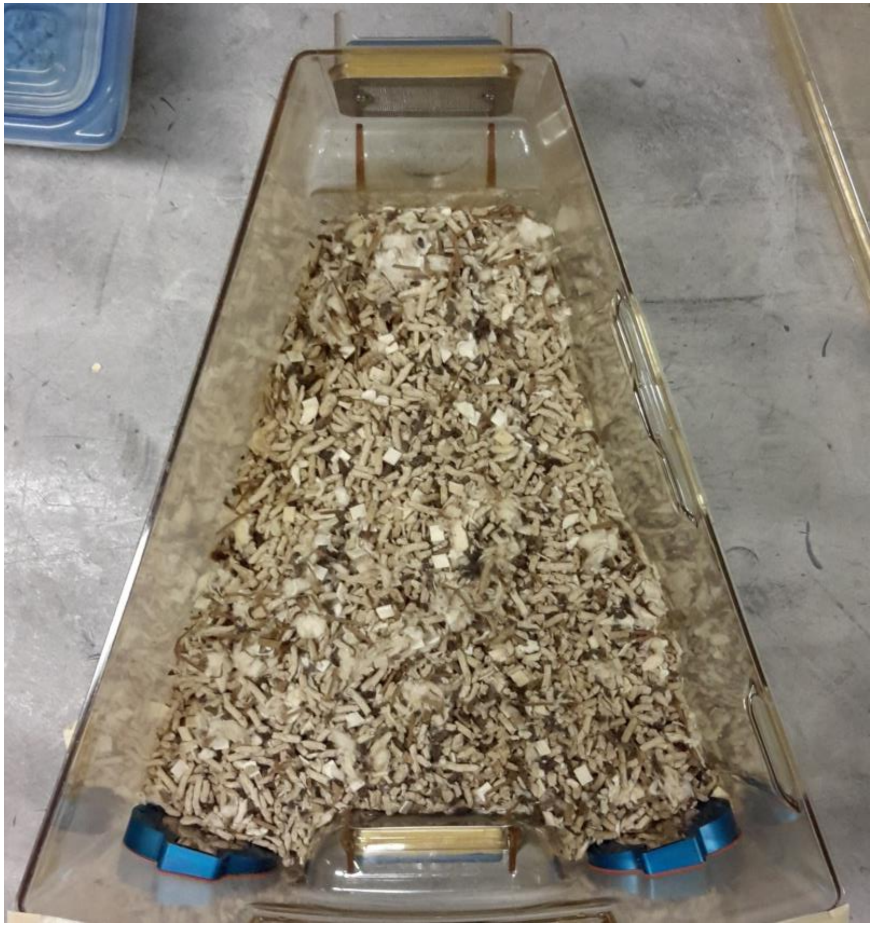

June 23 COMP 1 mid

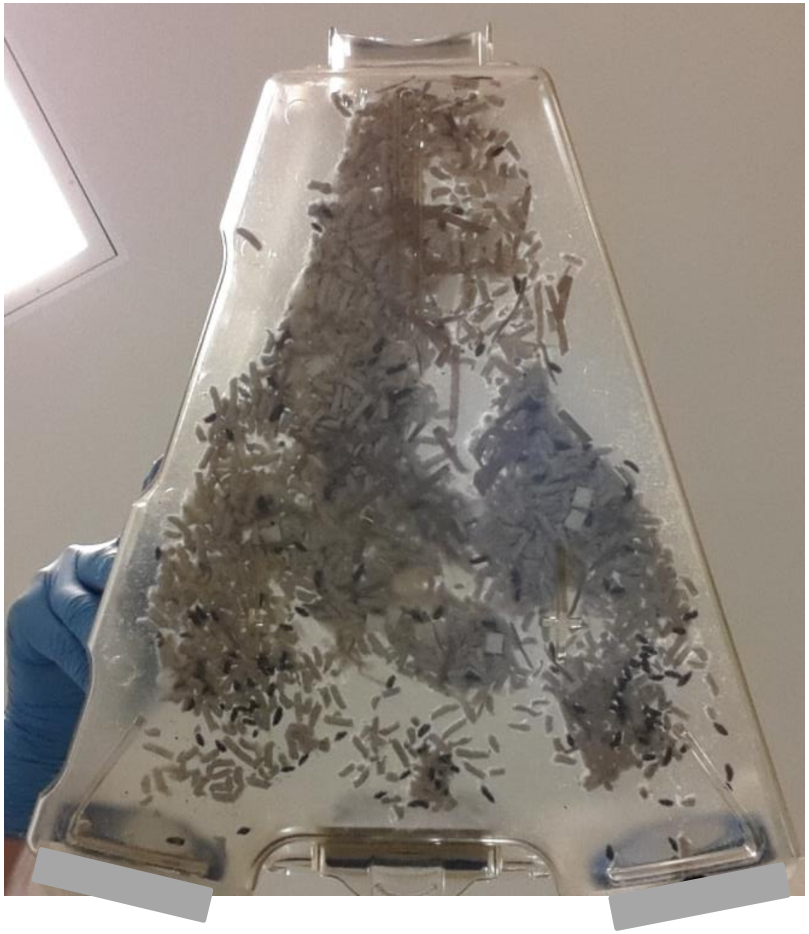

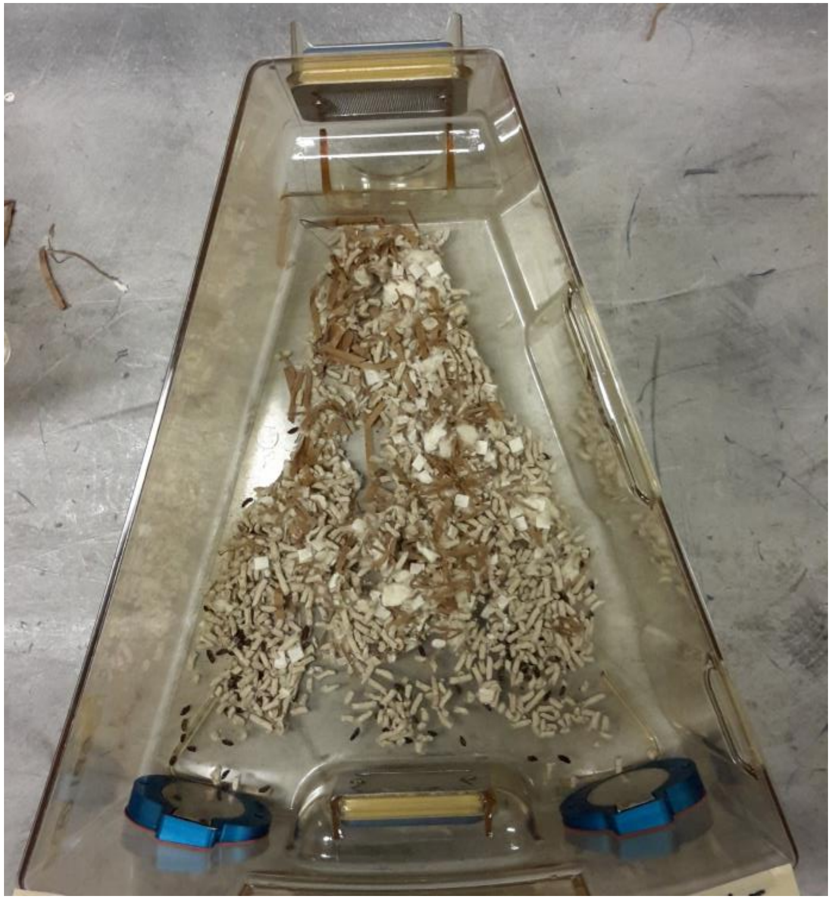

June 23 COMP 1 left

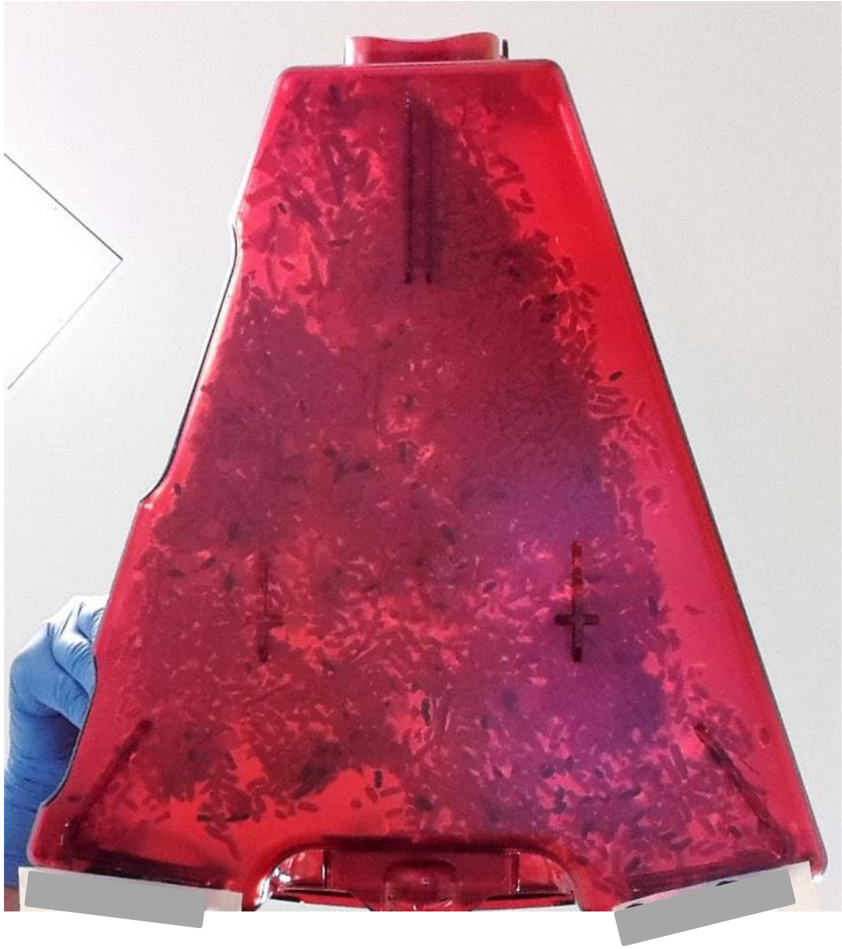

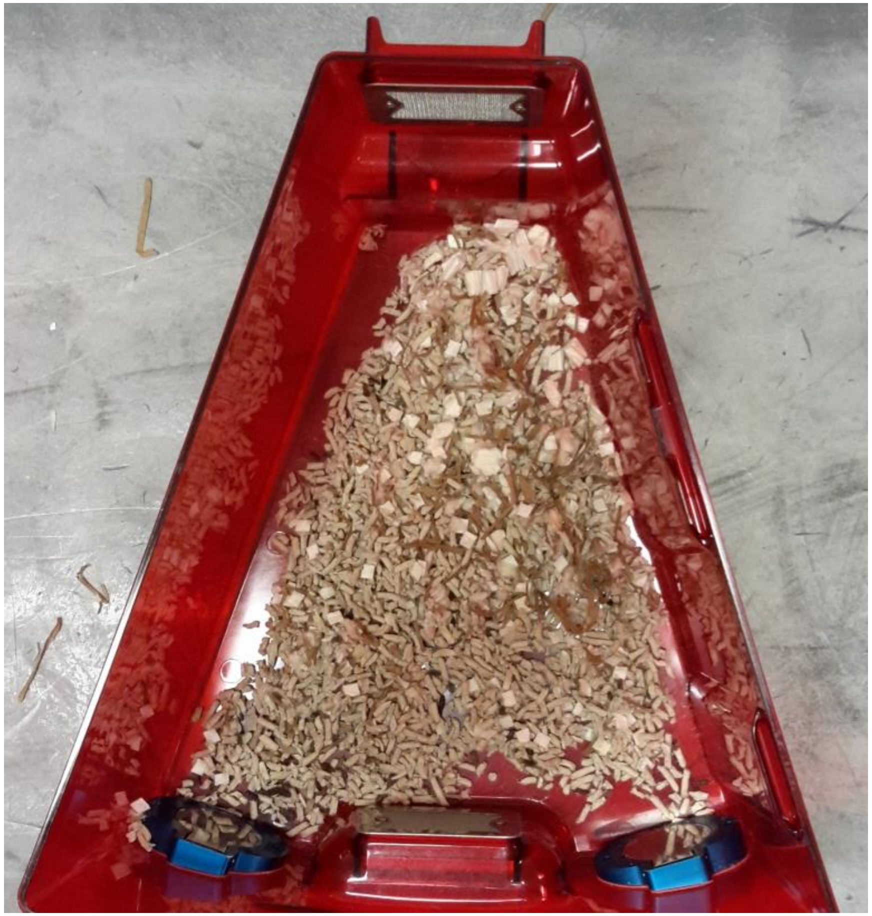

June 23 STD 1

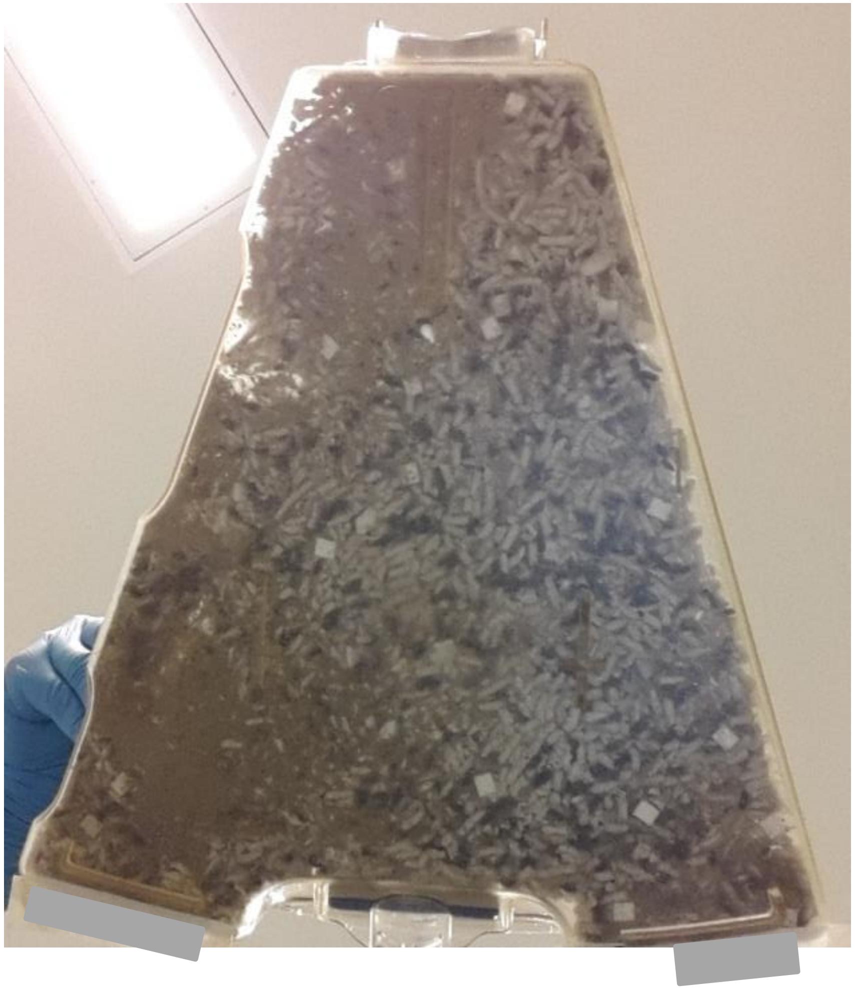

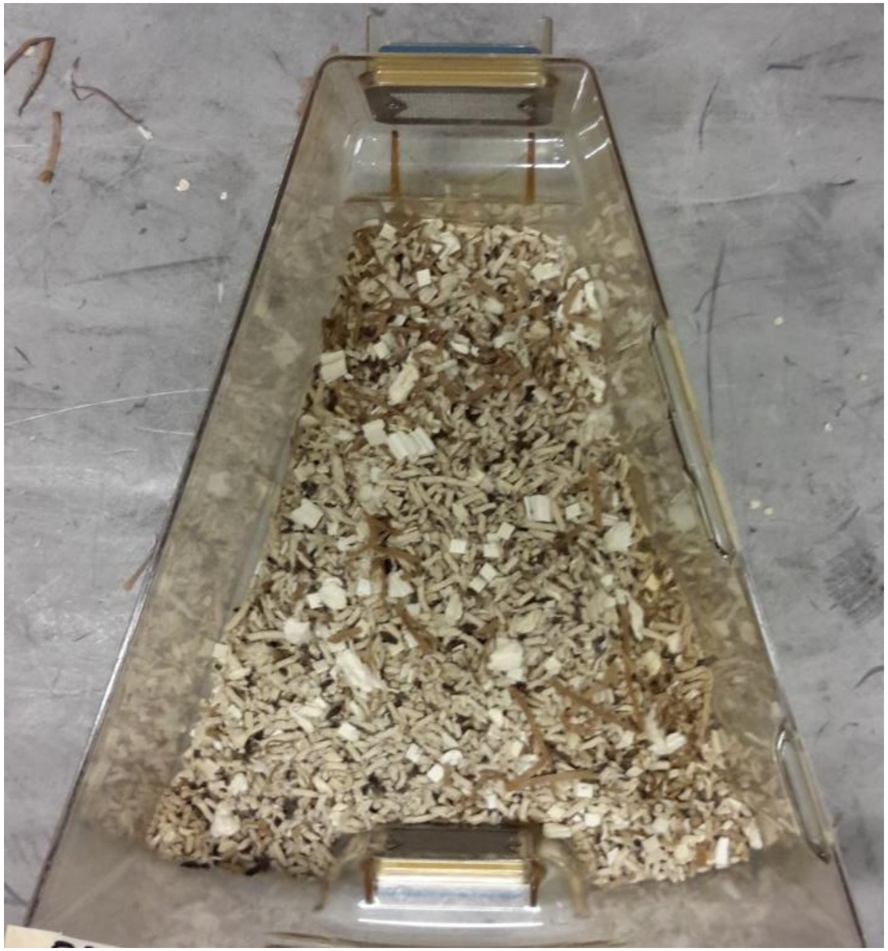

June 23 COMP 2 left

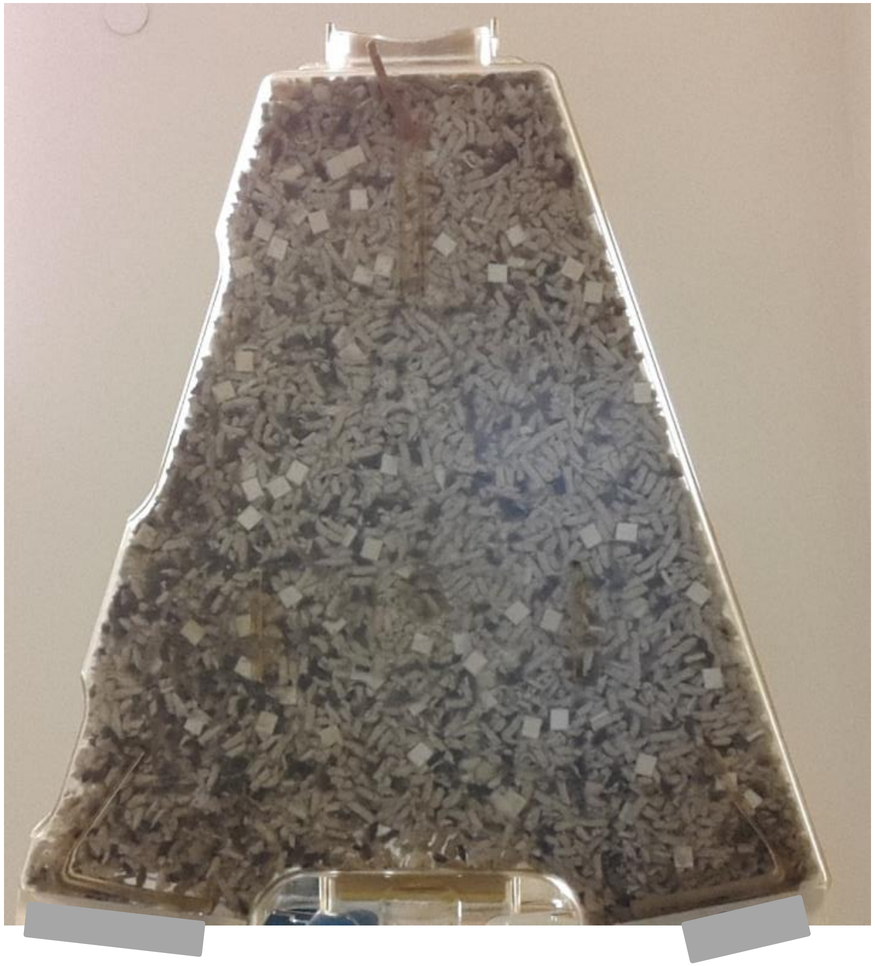

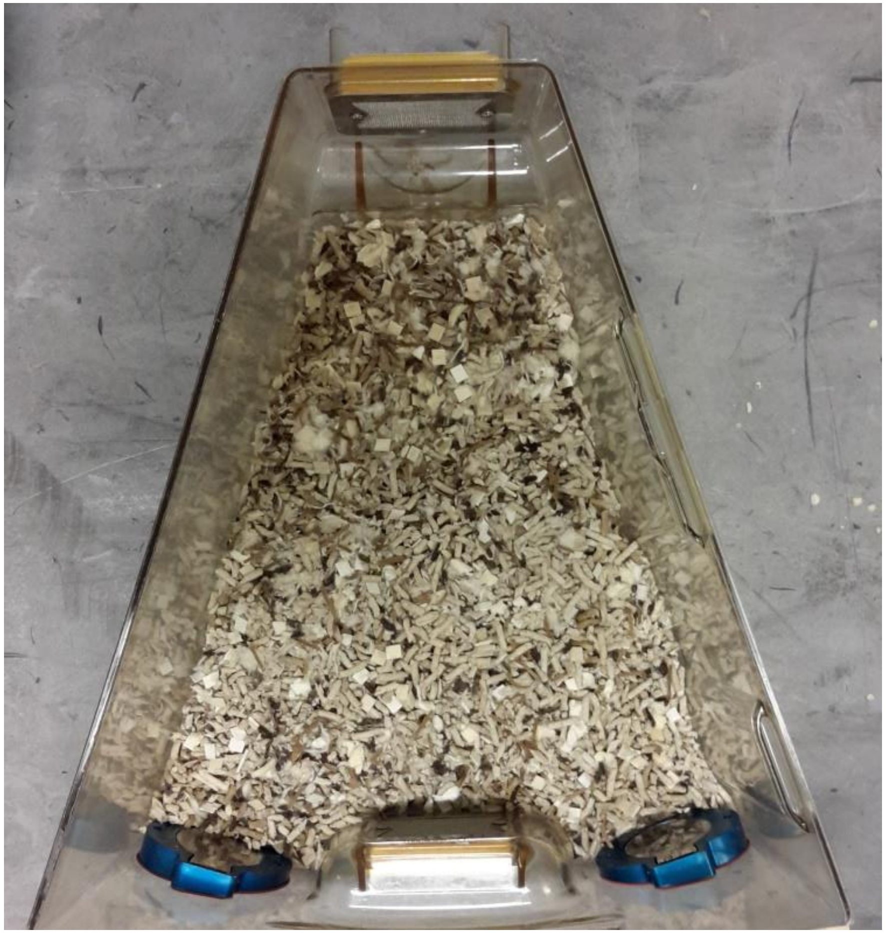

June 23 COMP 2 mid

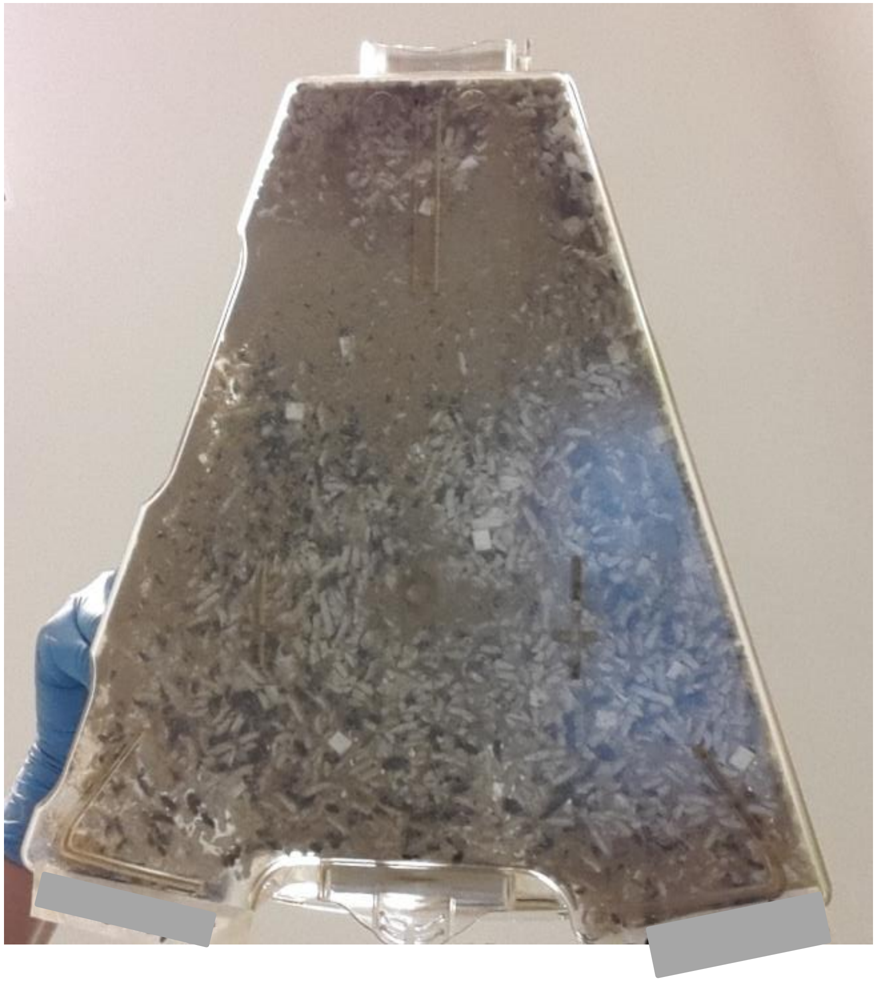

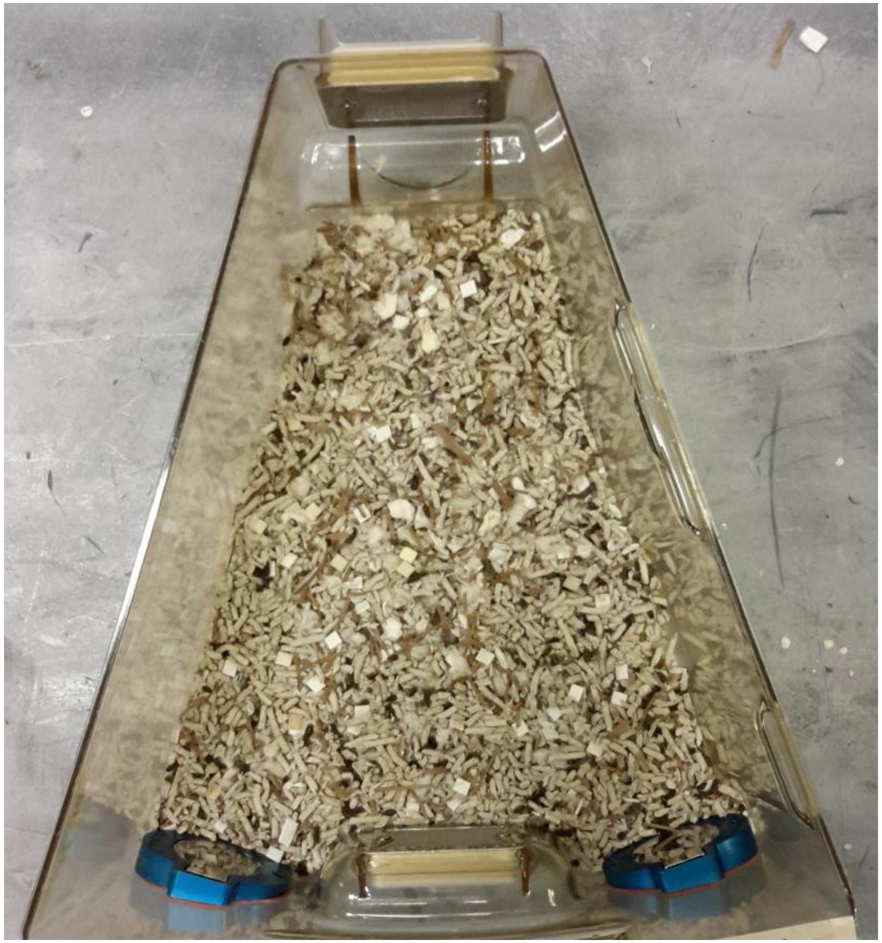

June 23 COMP 2 right

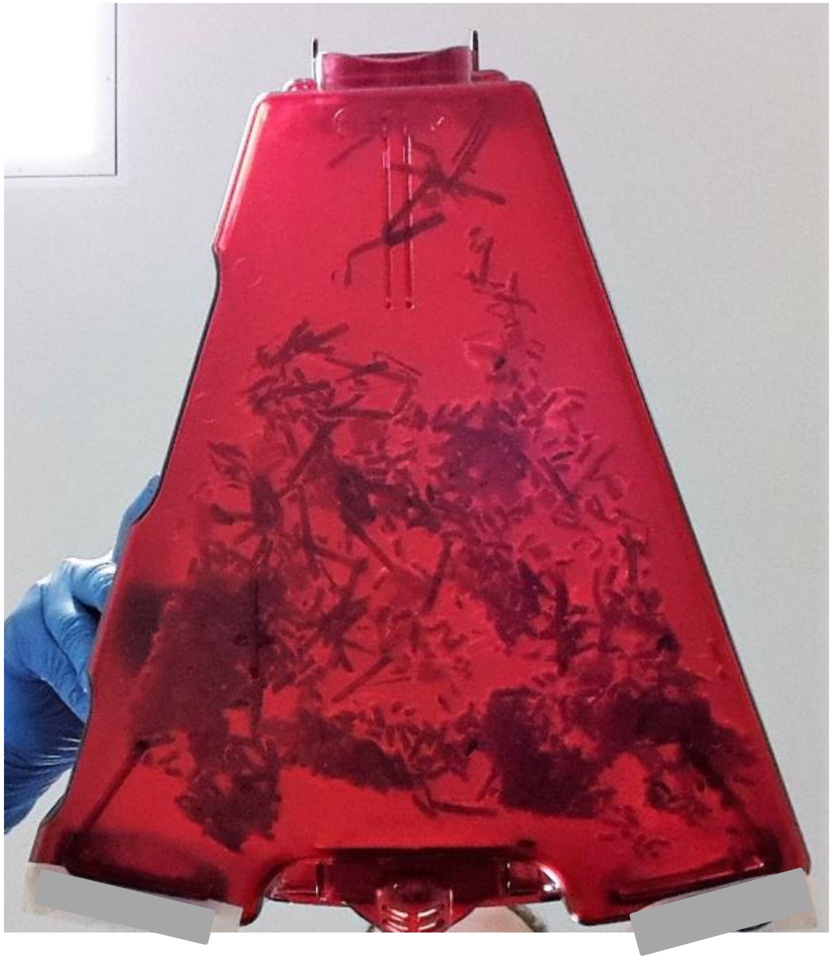

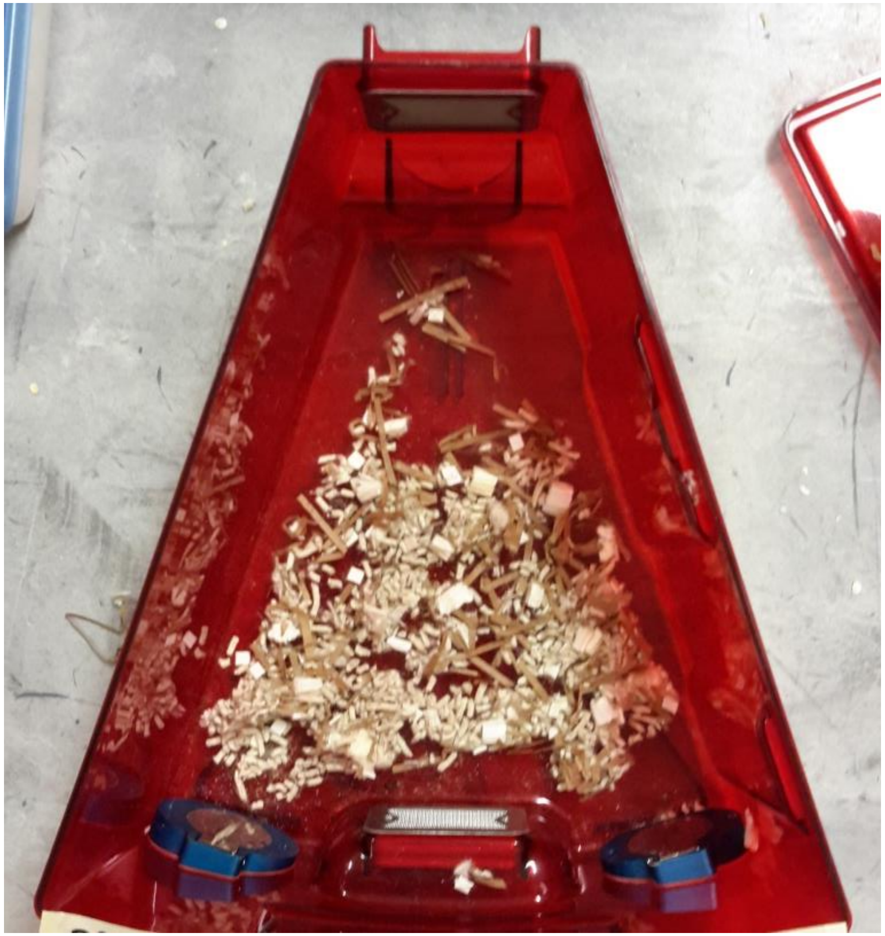

June 23 STD 2

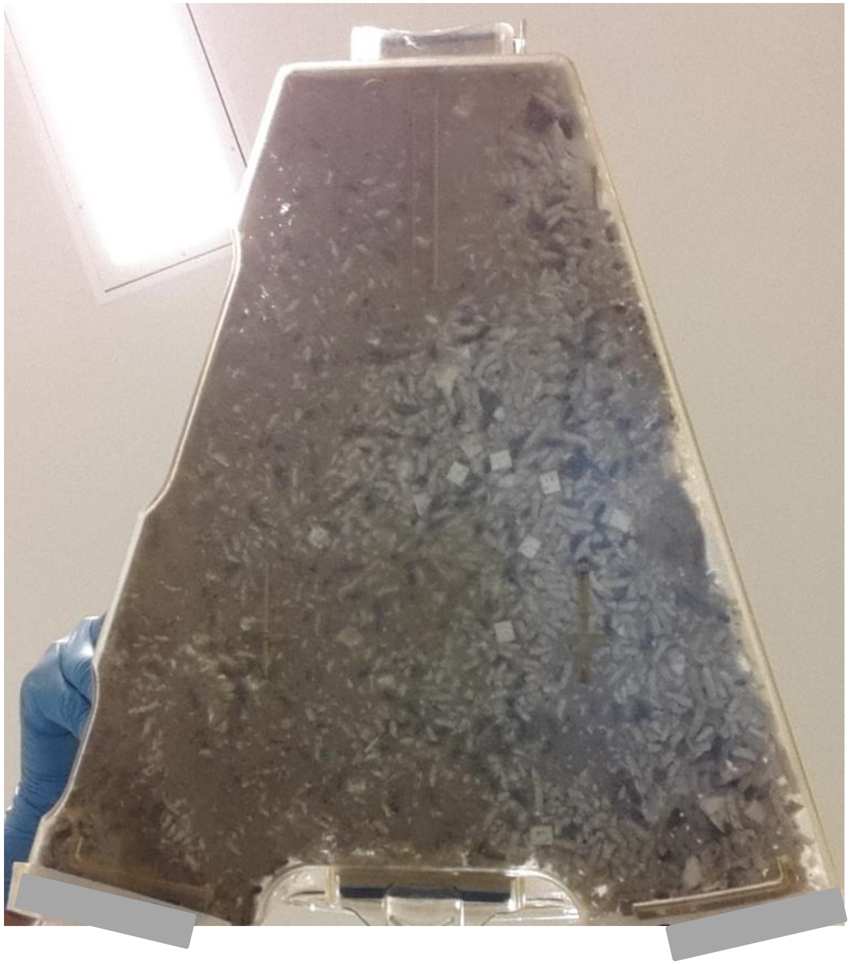

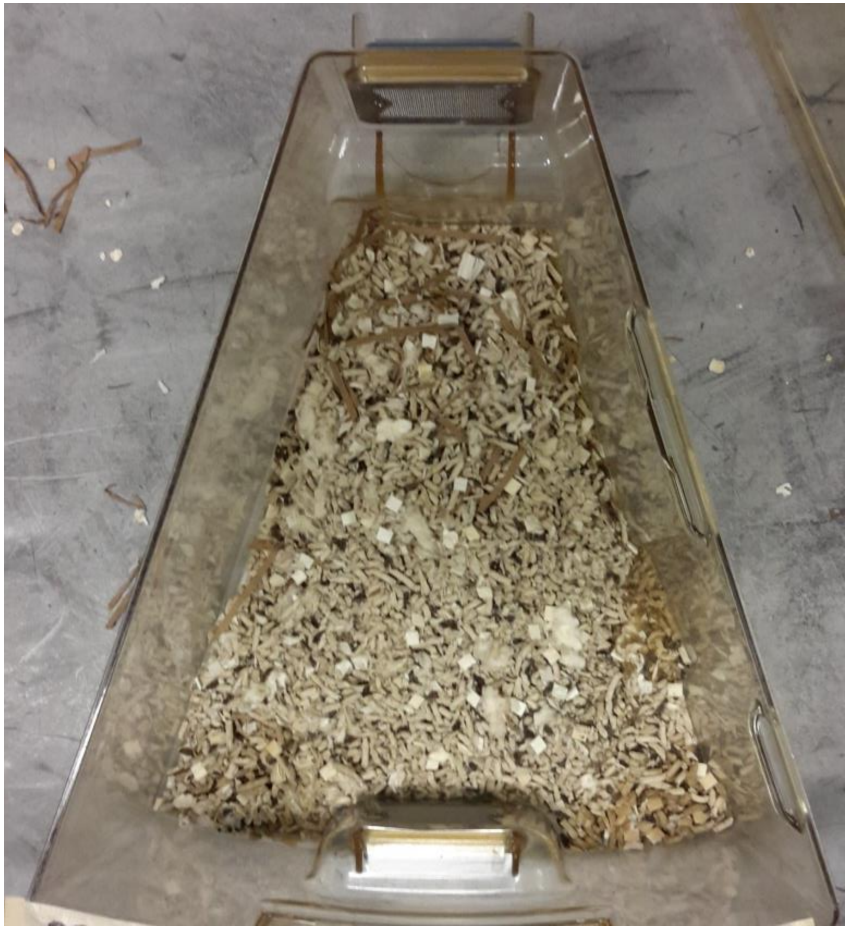

June 24 COMP 3 left

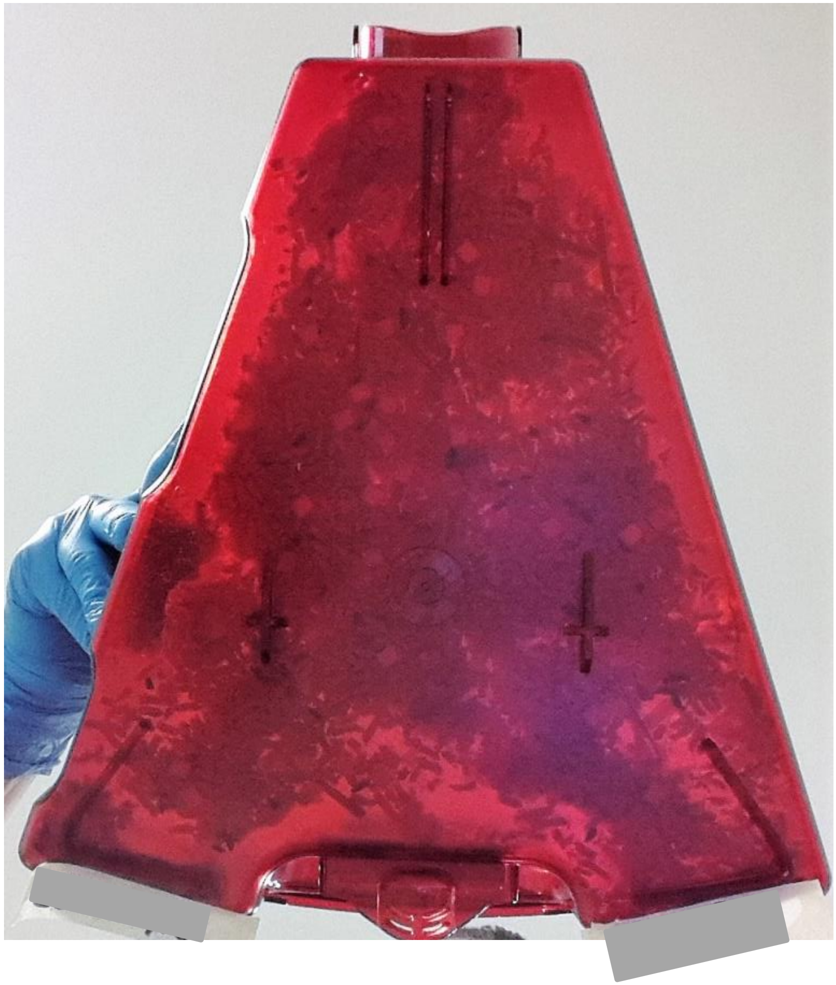

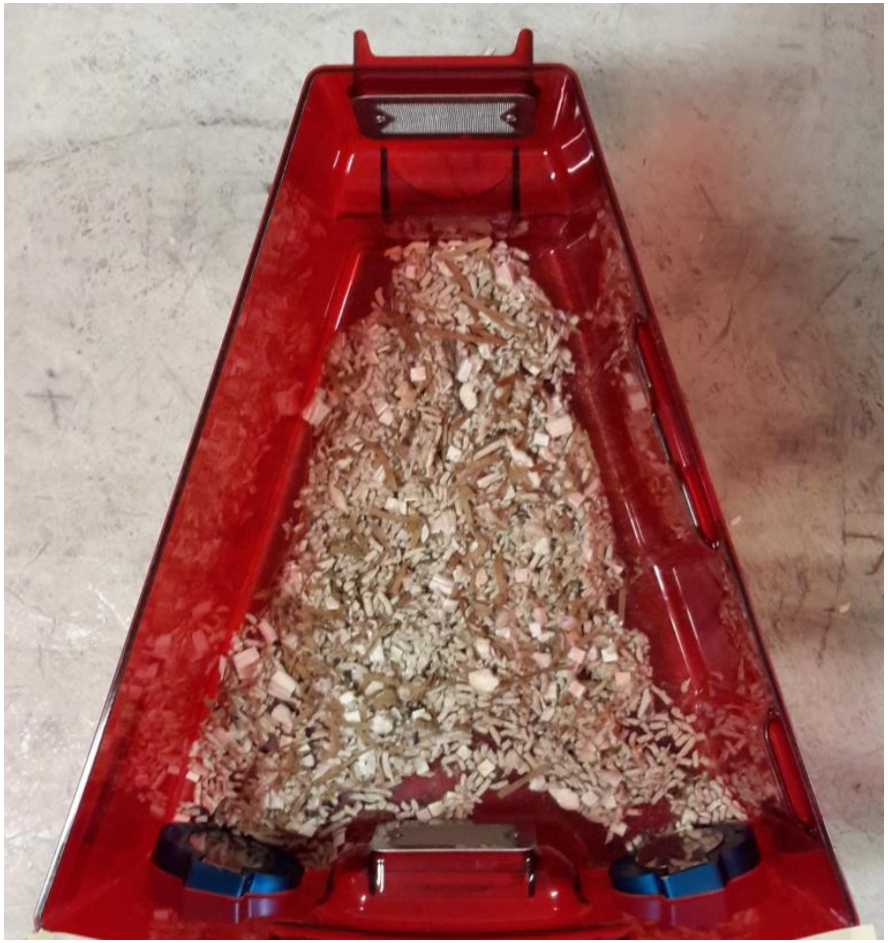

June 24 COMP 3 mid

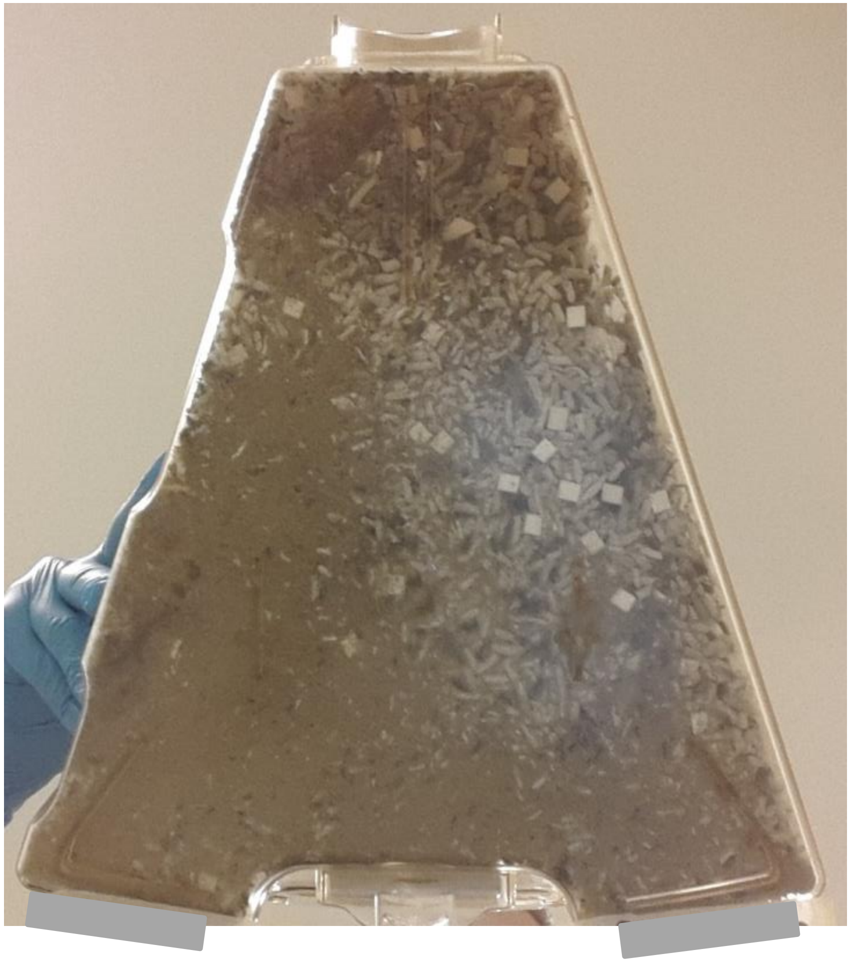

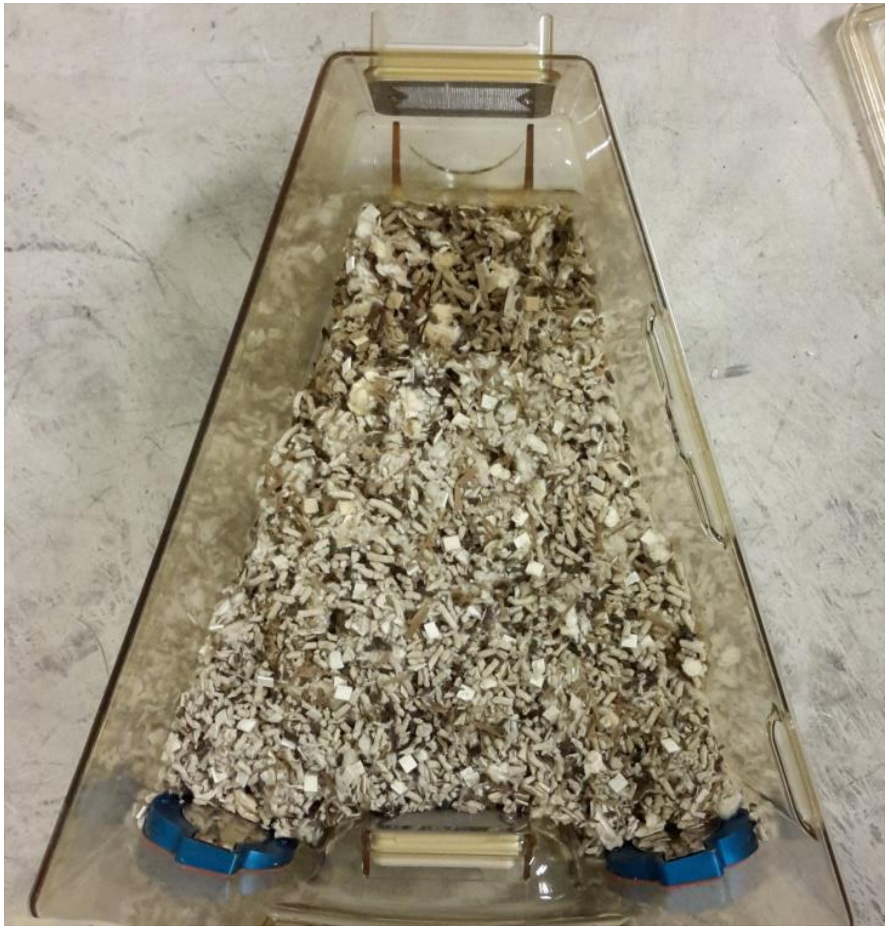

June 24 COMP 3 right

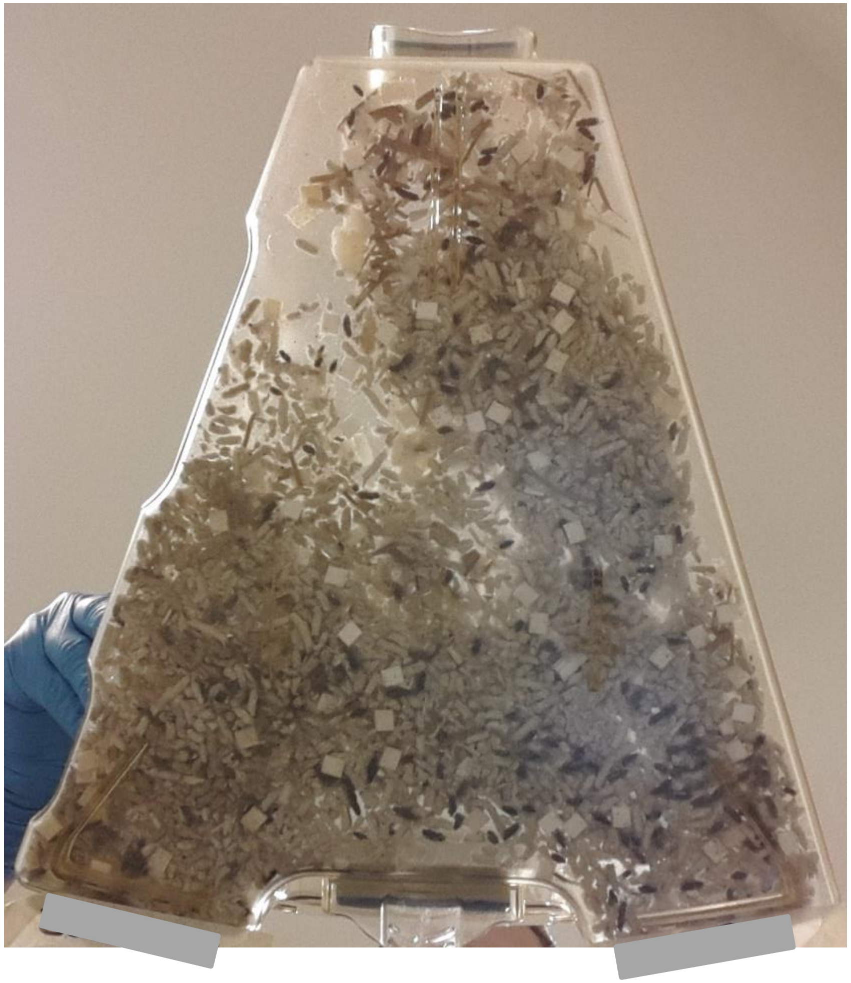

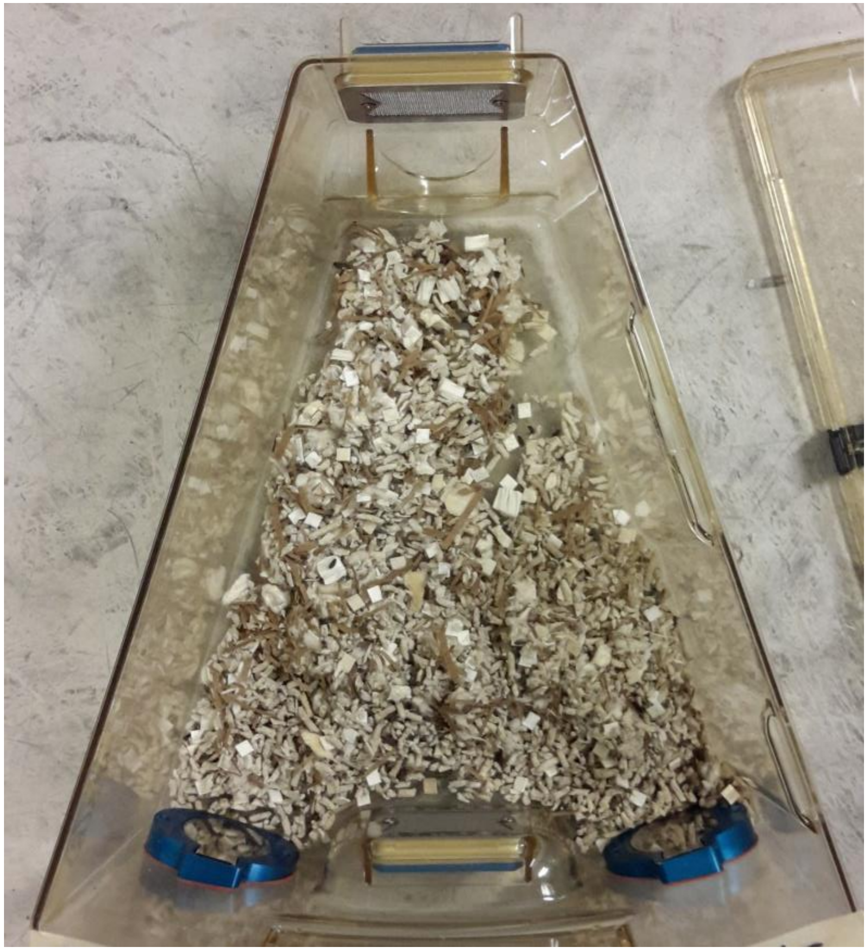

June 24 STD 3

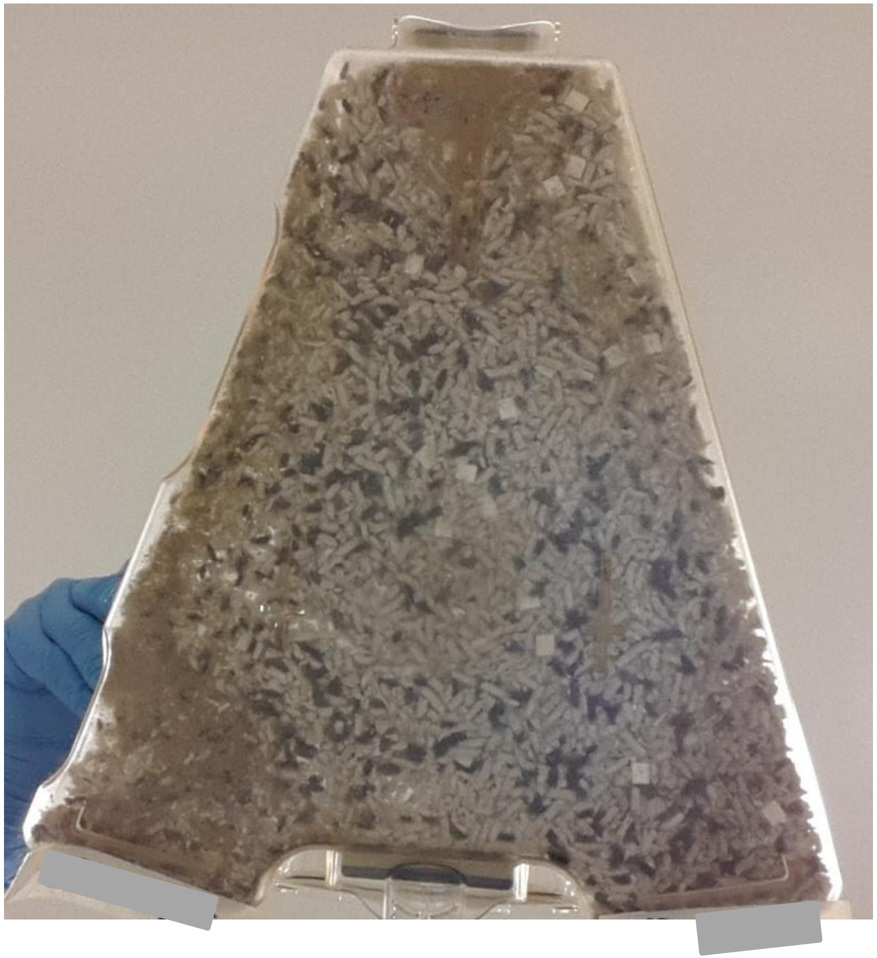

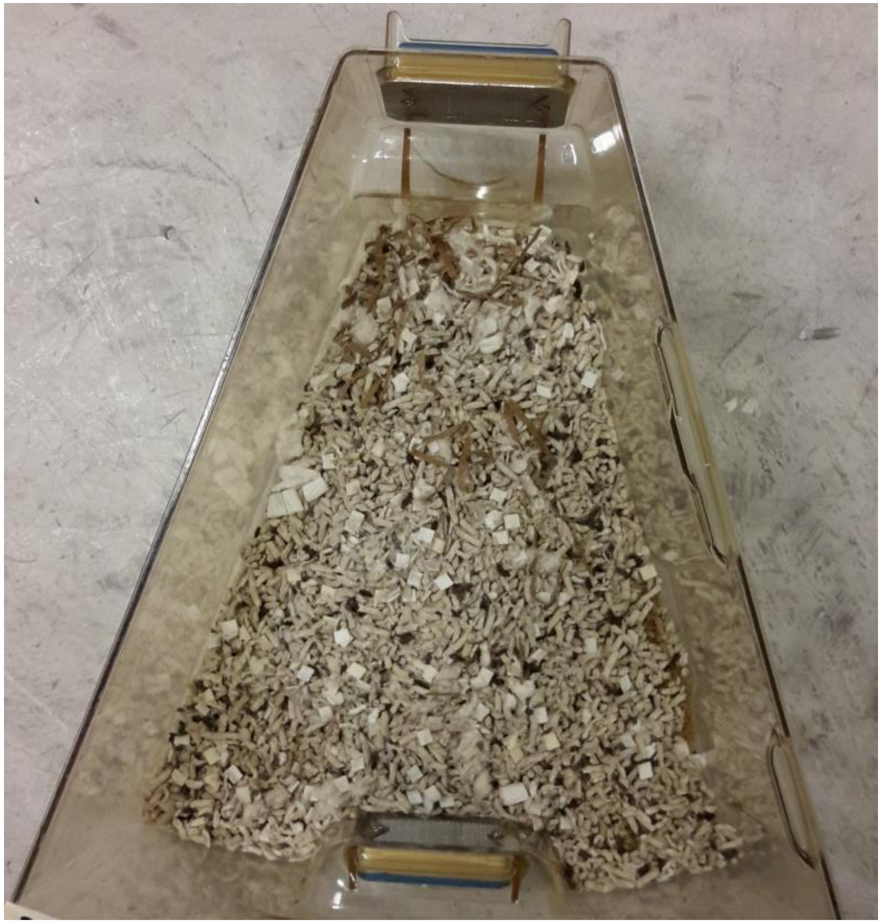

June 24 COMP 4 right

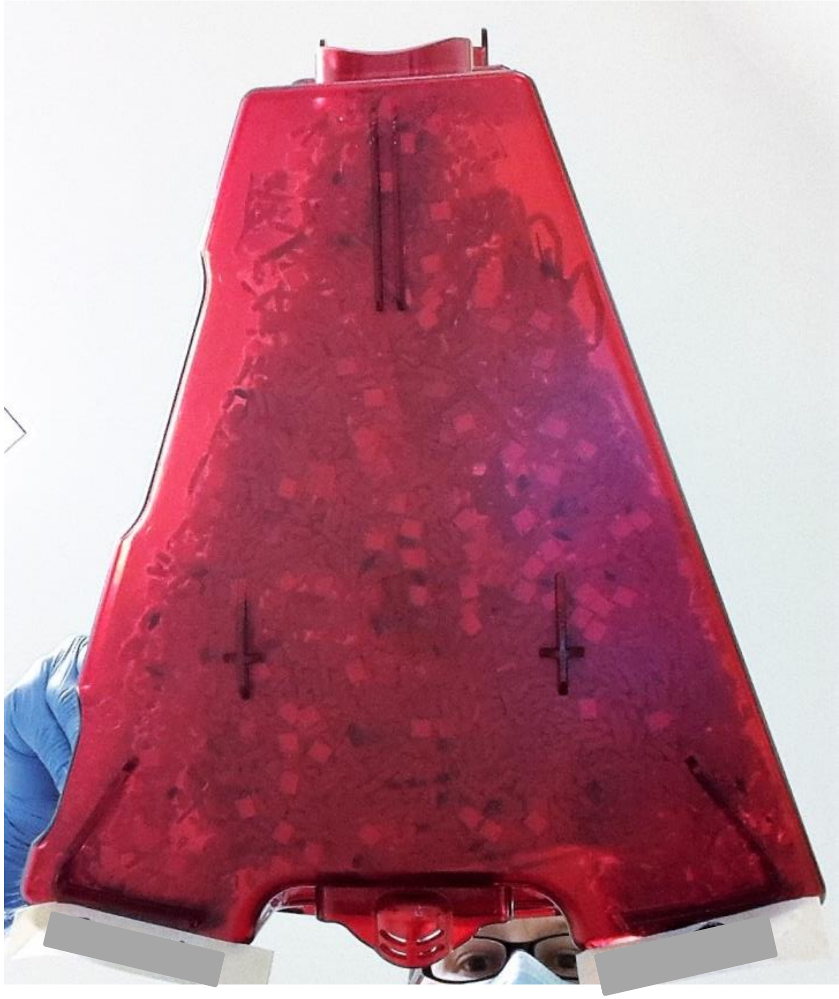

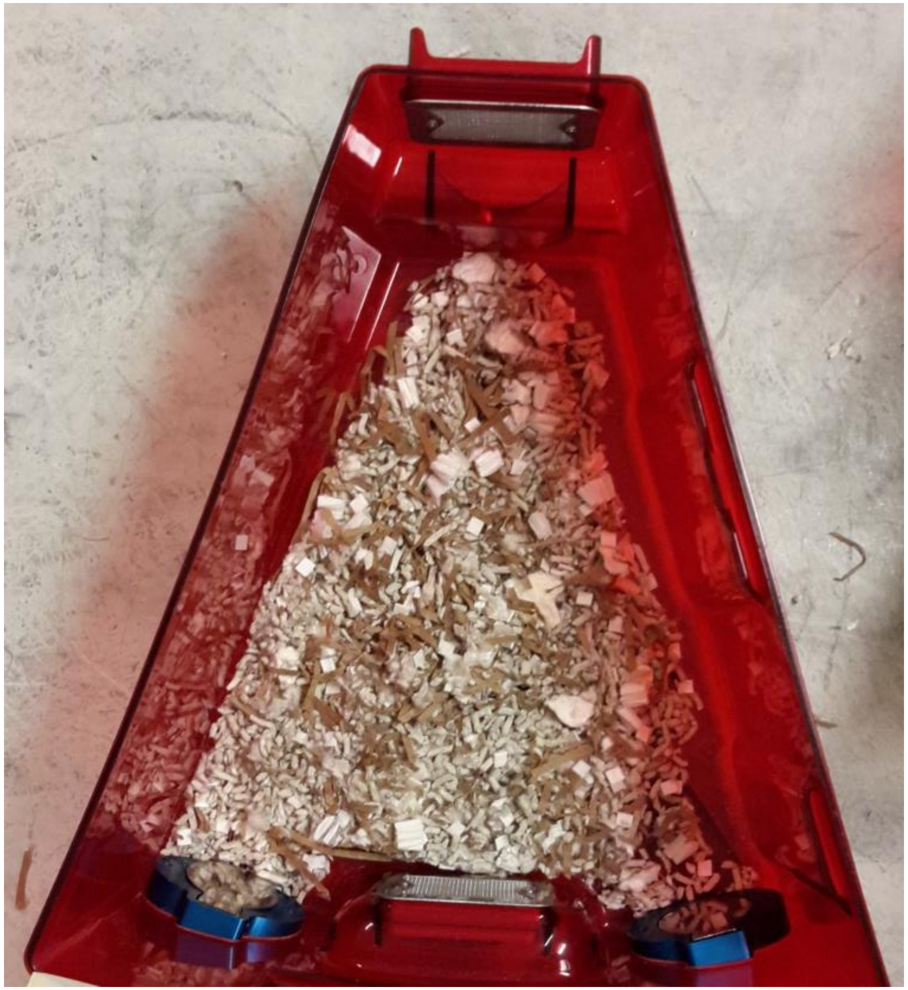

June 24 COMP 4 left

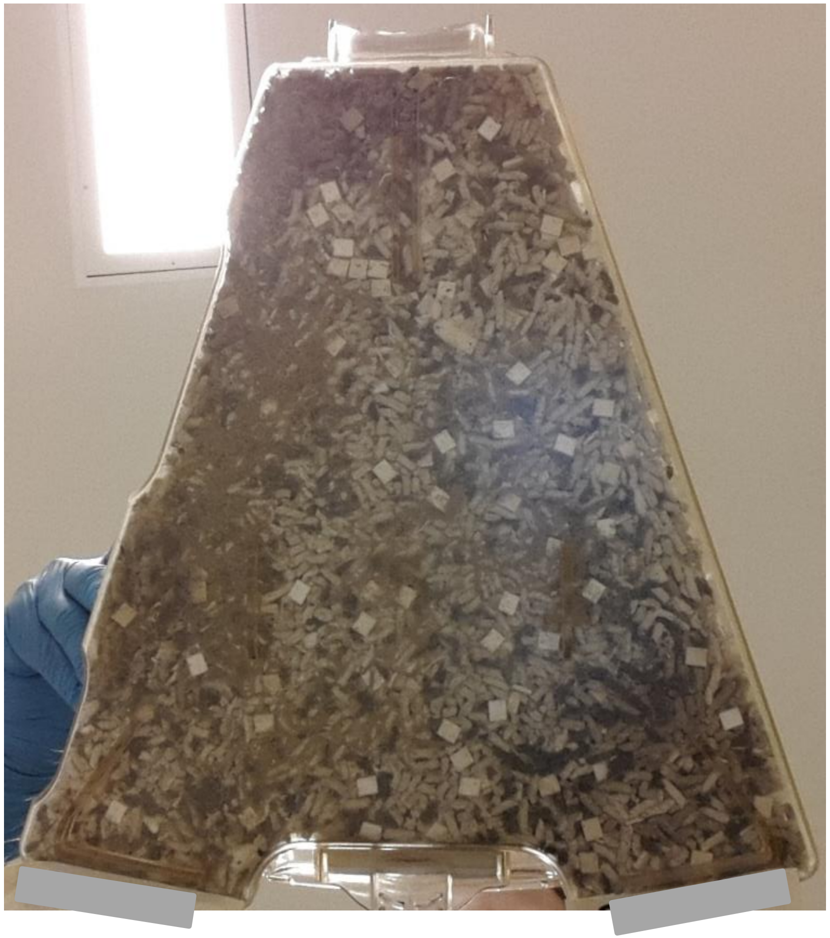

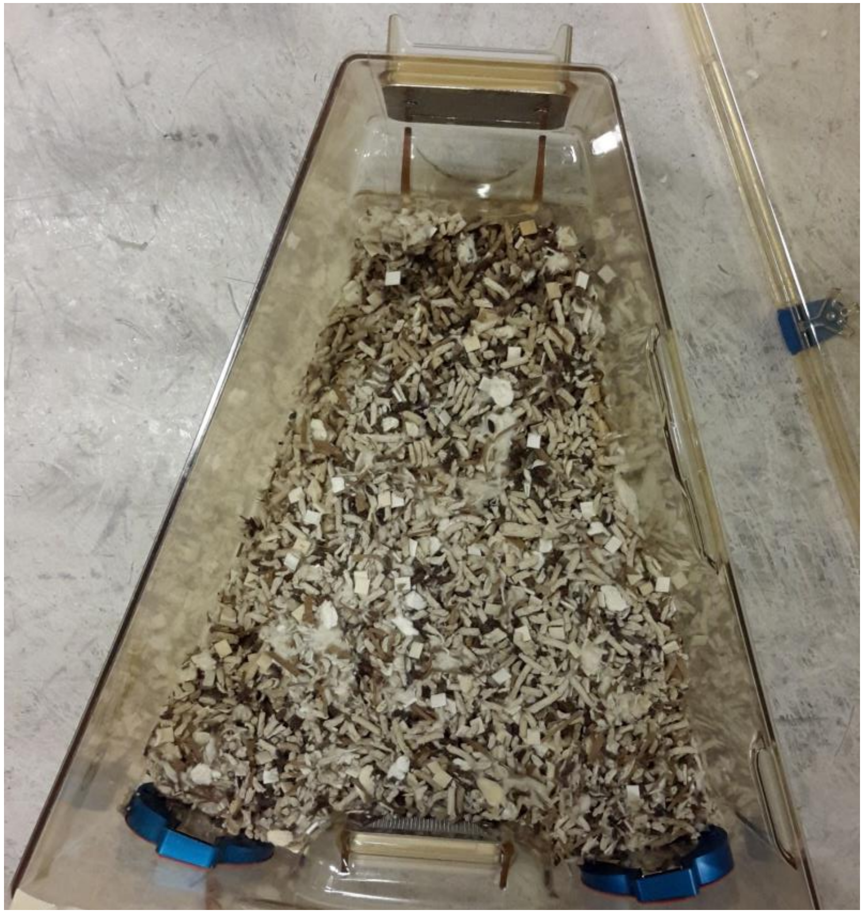

June 24 COMP 4 mid

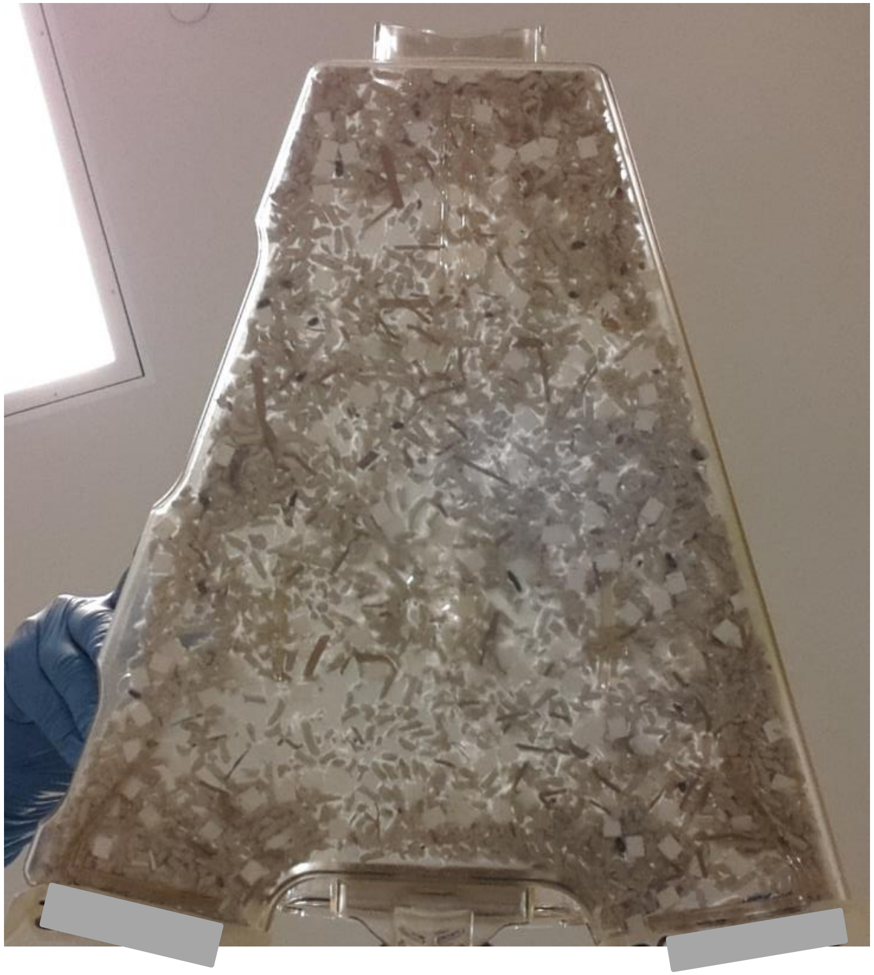

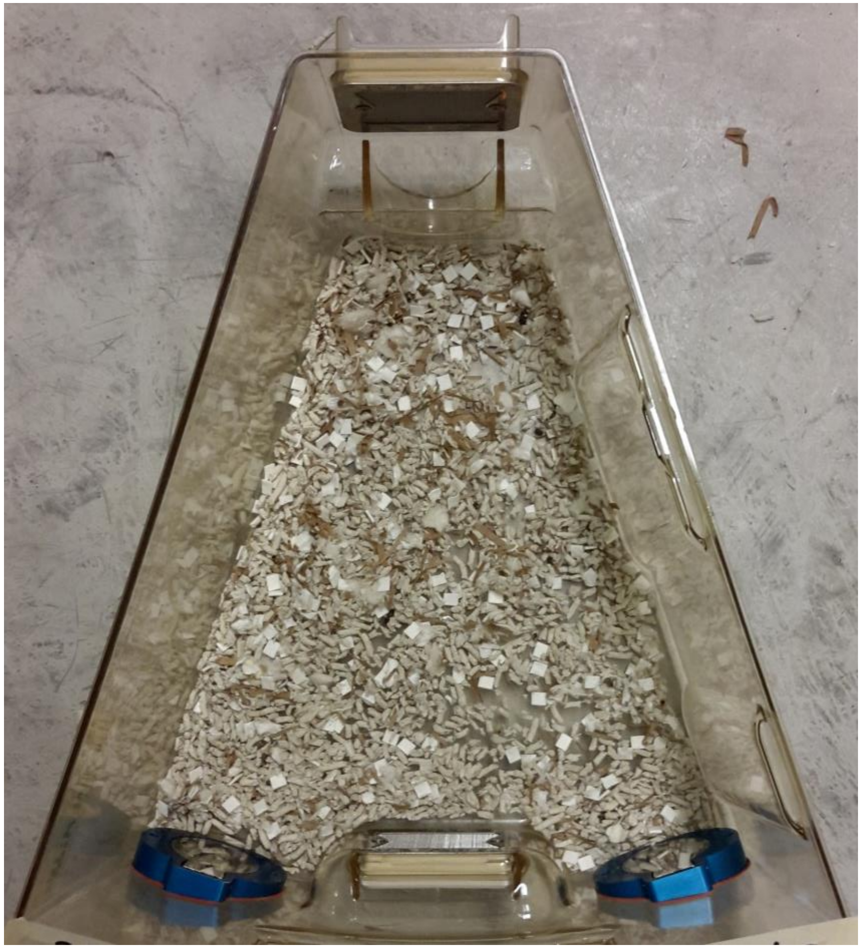

June 24 STD 4

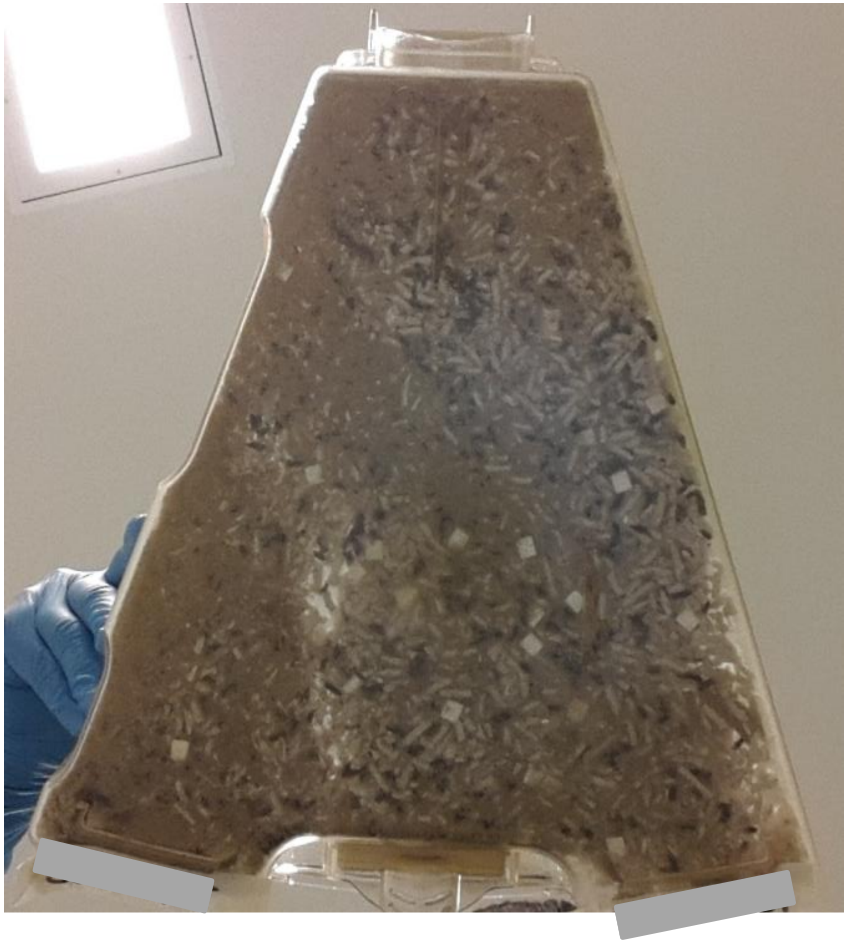

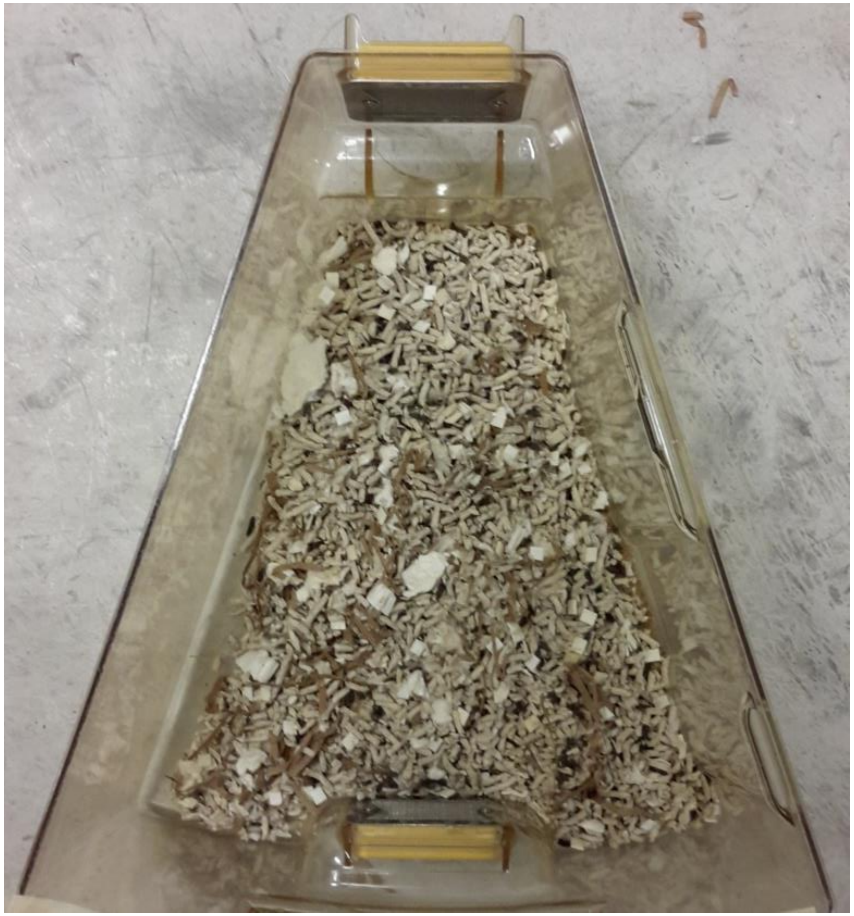

June 25 COMP 5 right

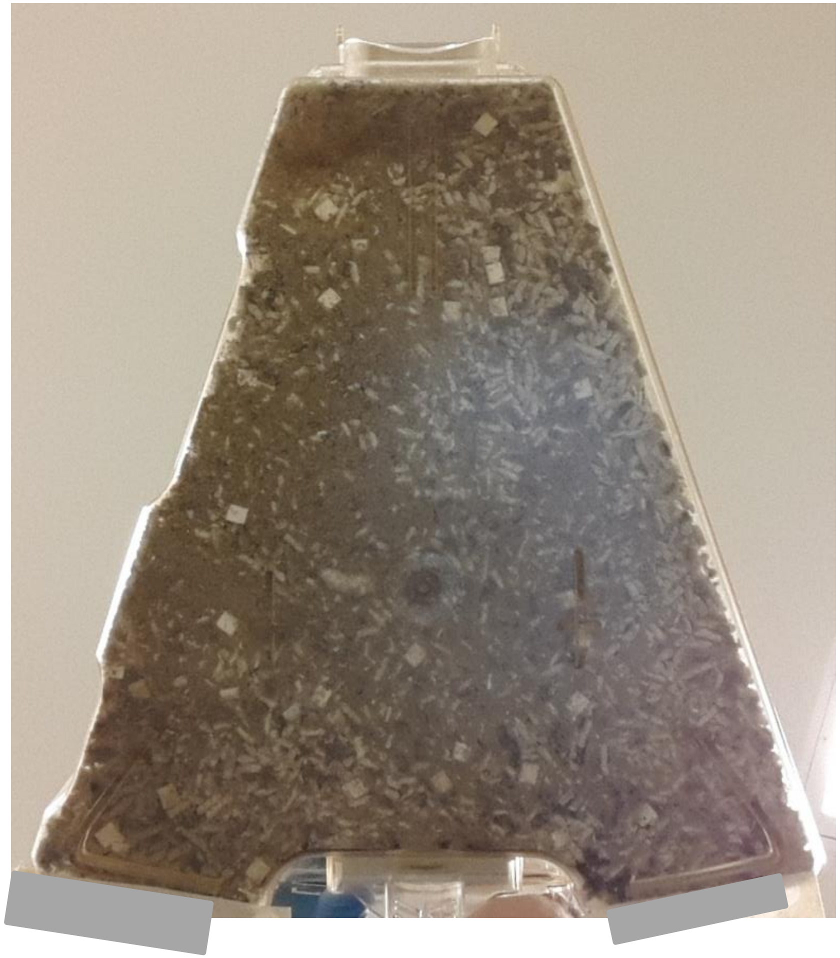

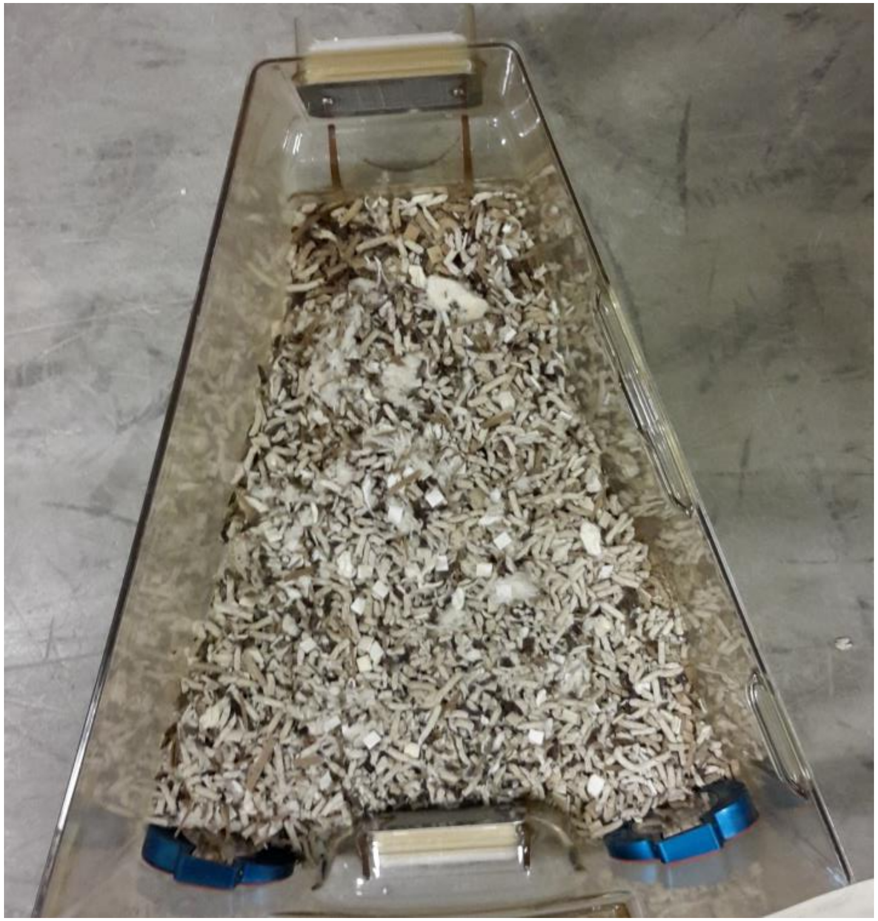

June 25 COMP 5 mid

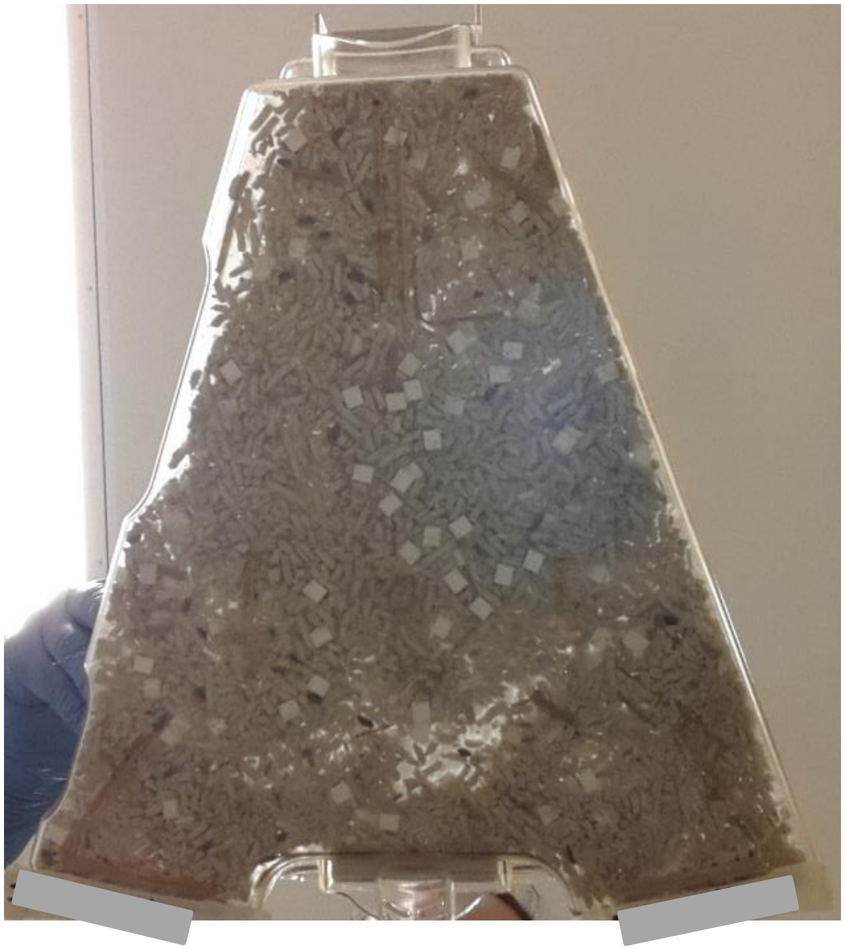

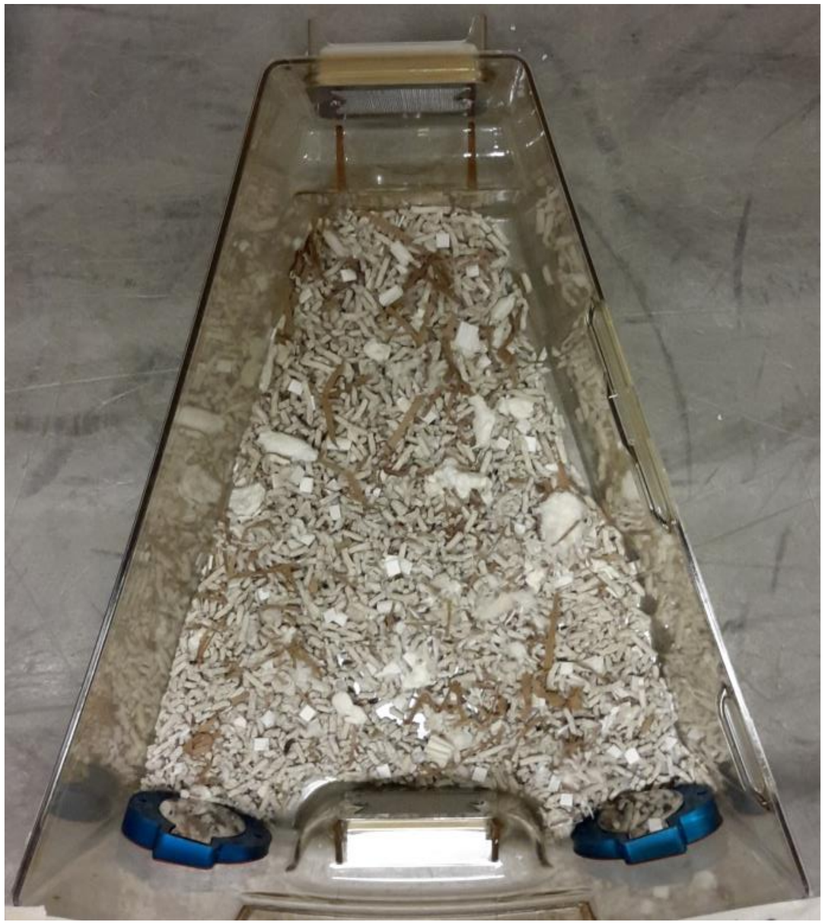

June 25 COMP 5 left

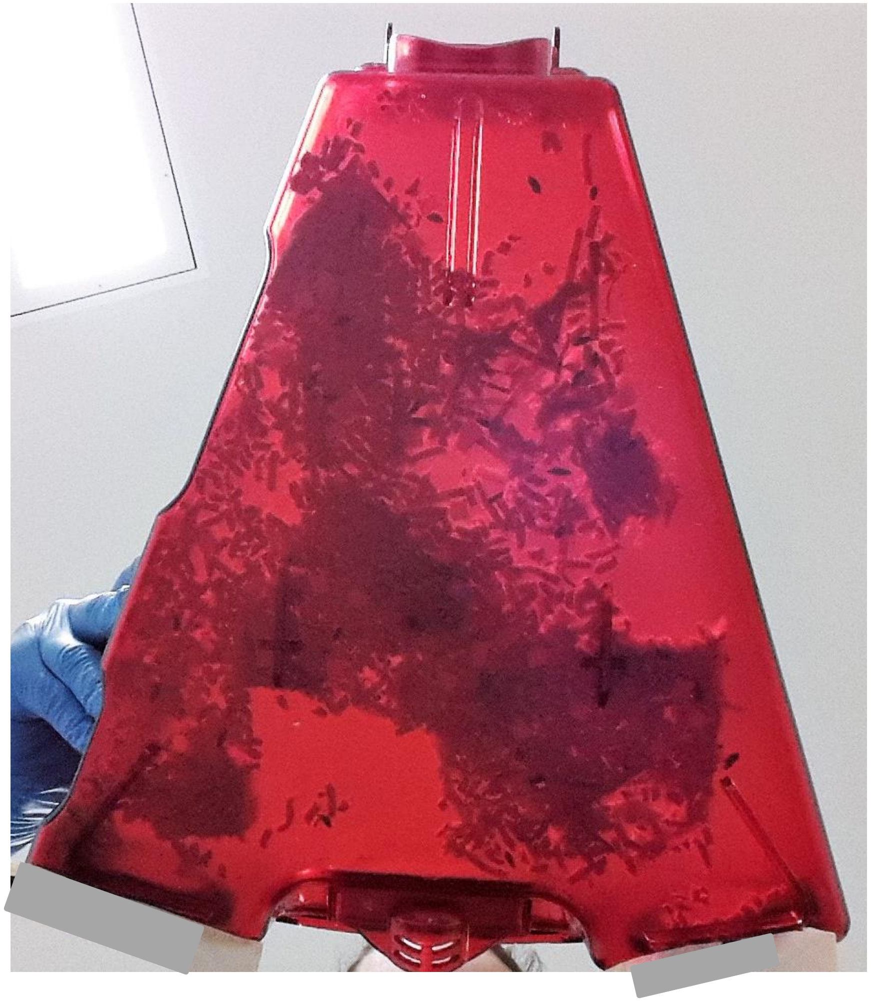

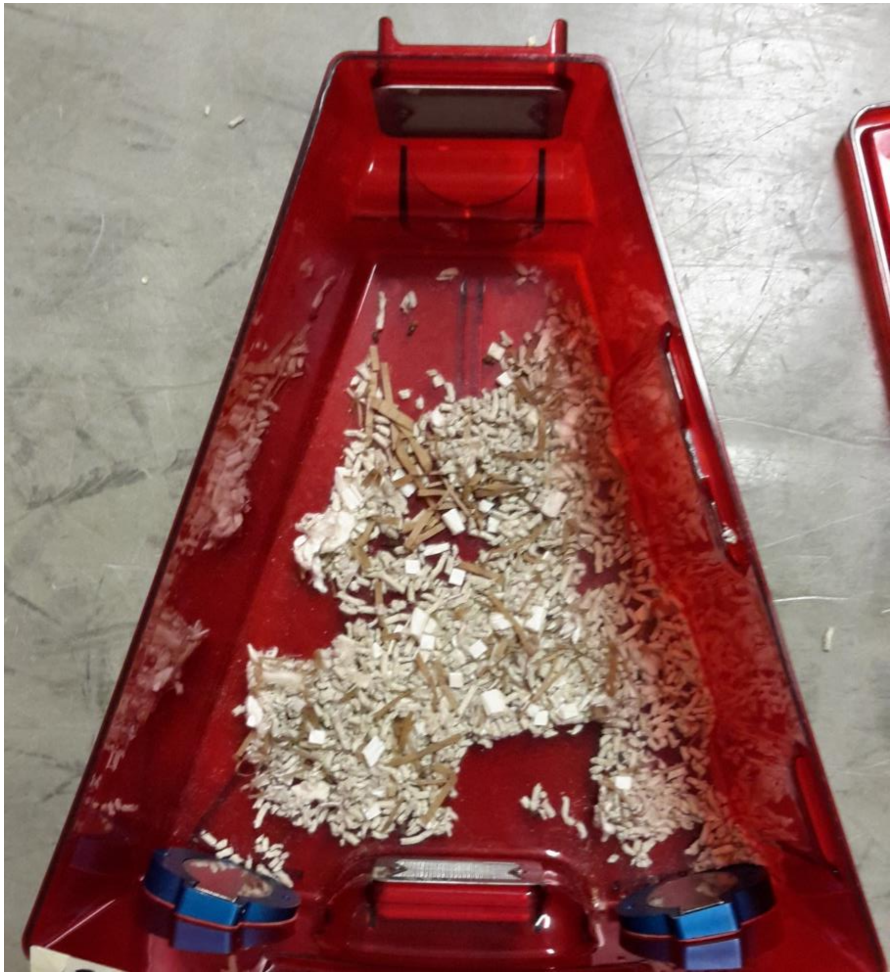

June 25 STD 5

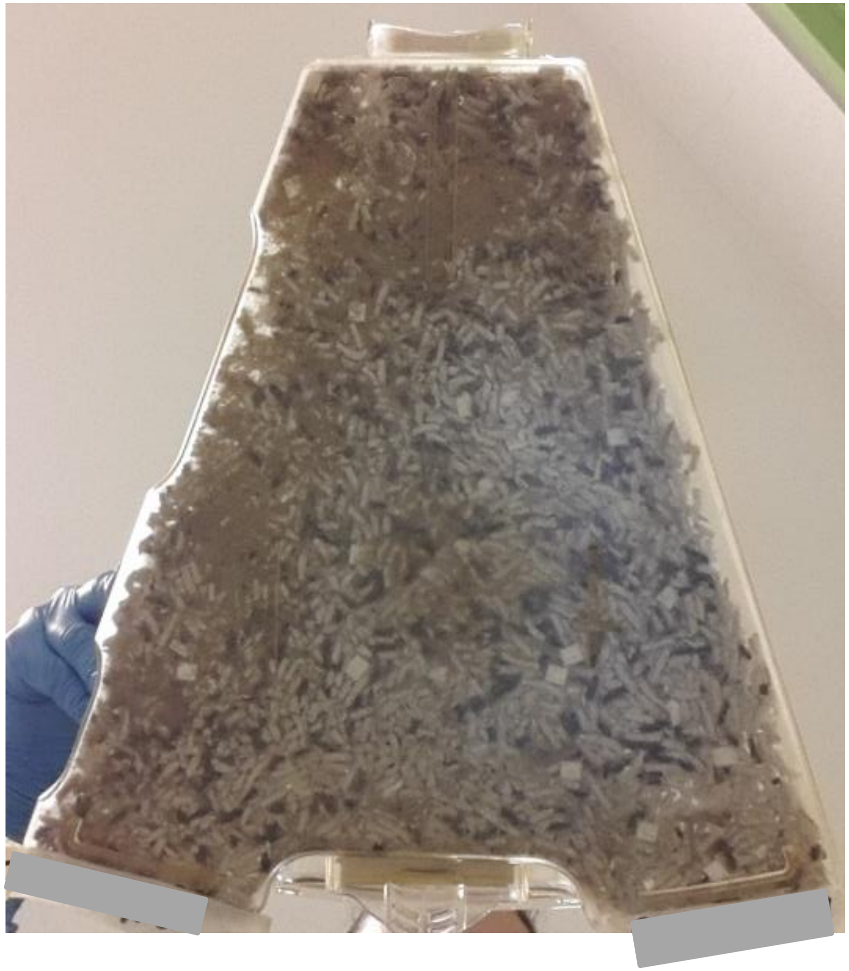

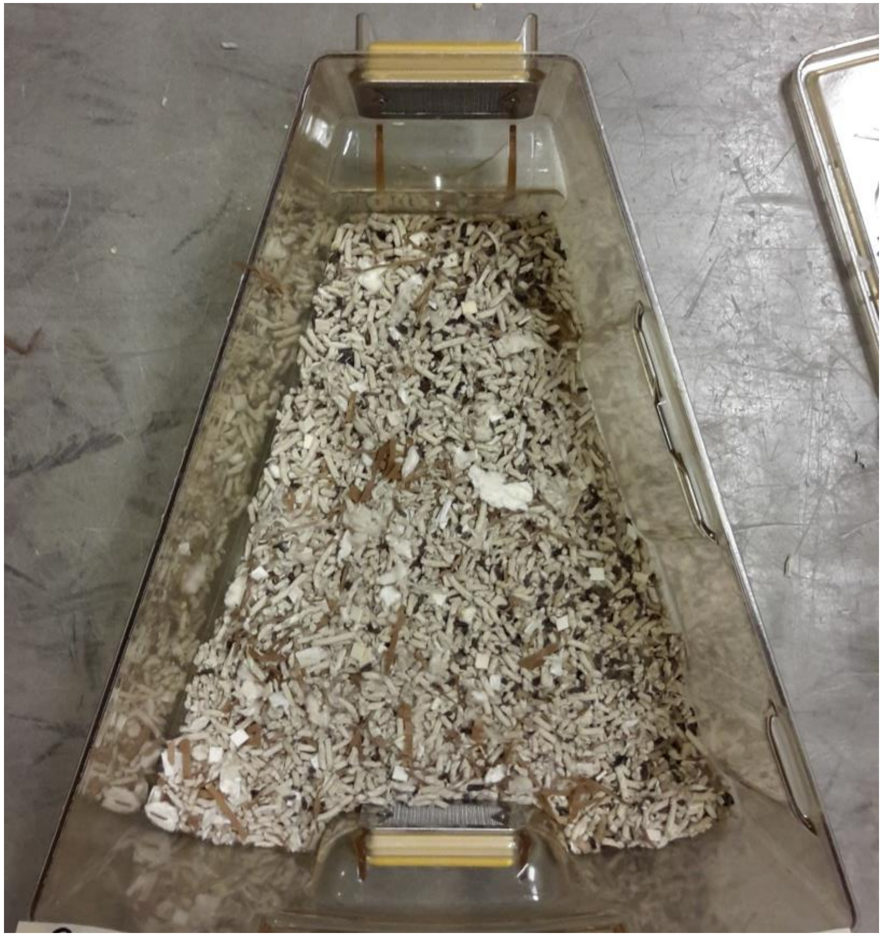

June 30 COMP 1 mid

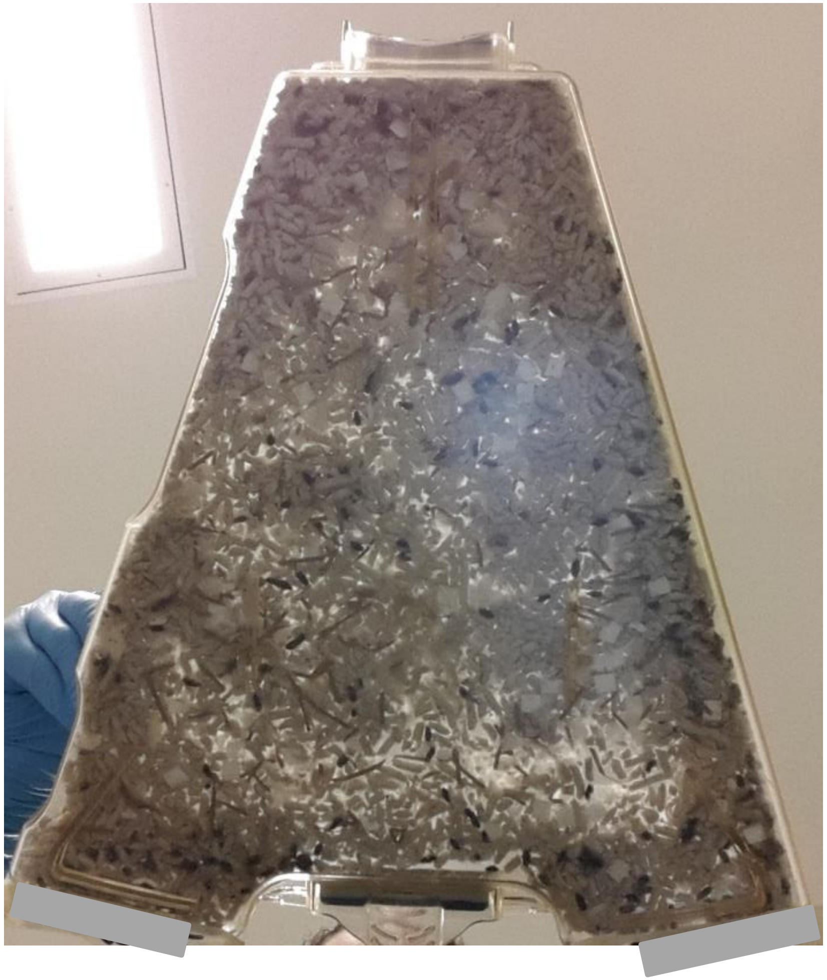

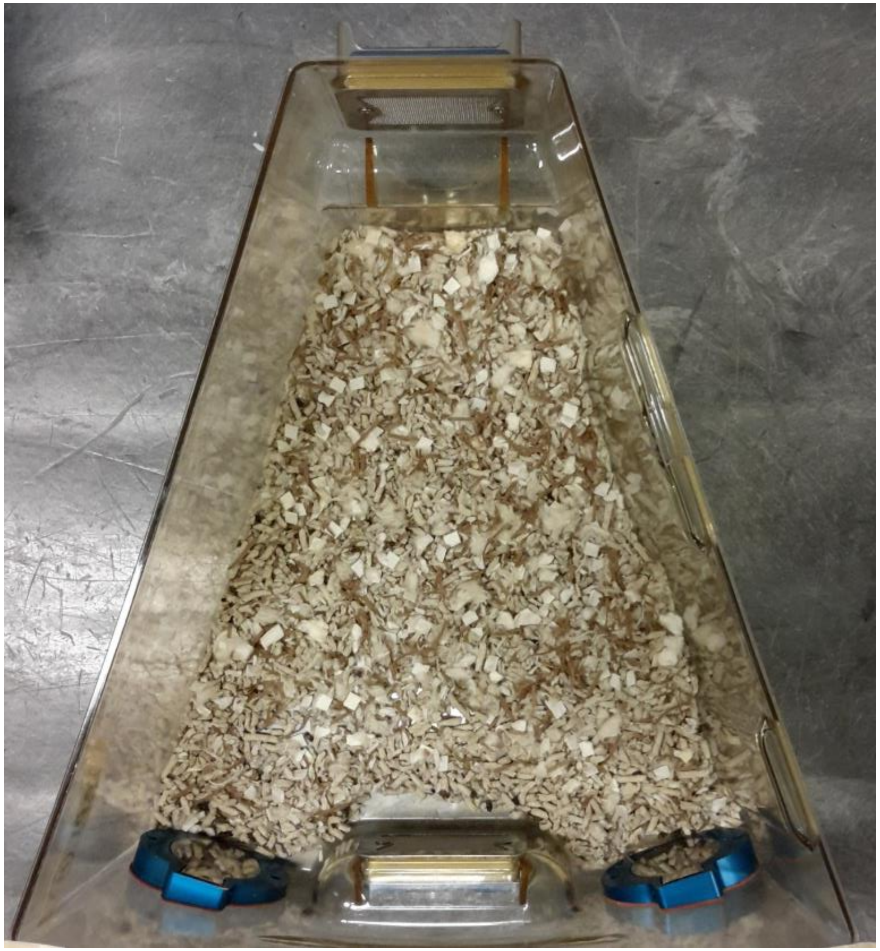

June 30 COMP 1 left

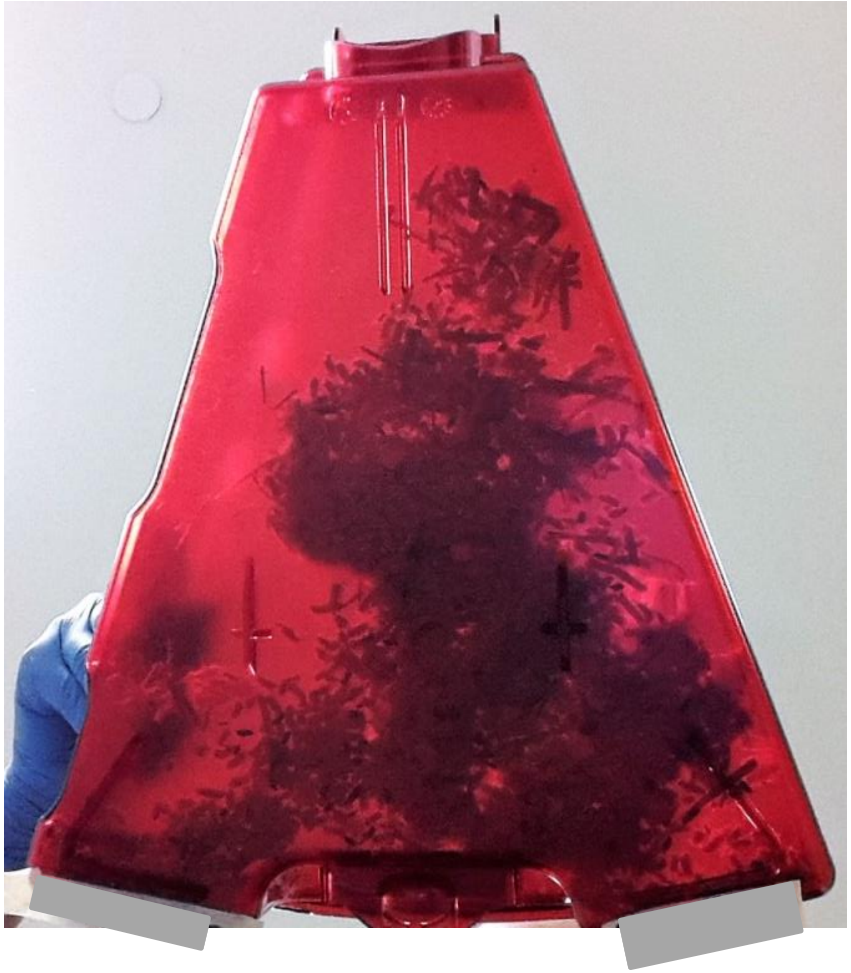

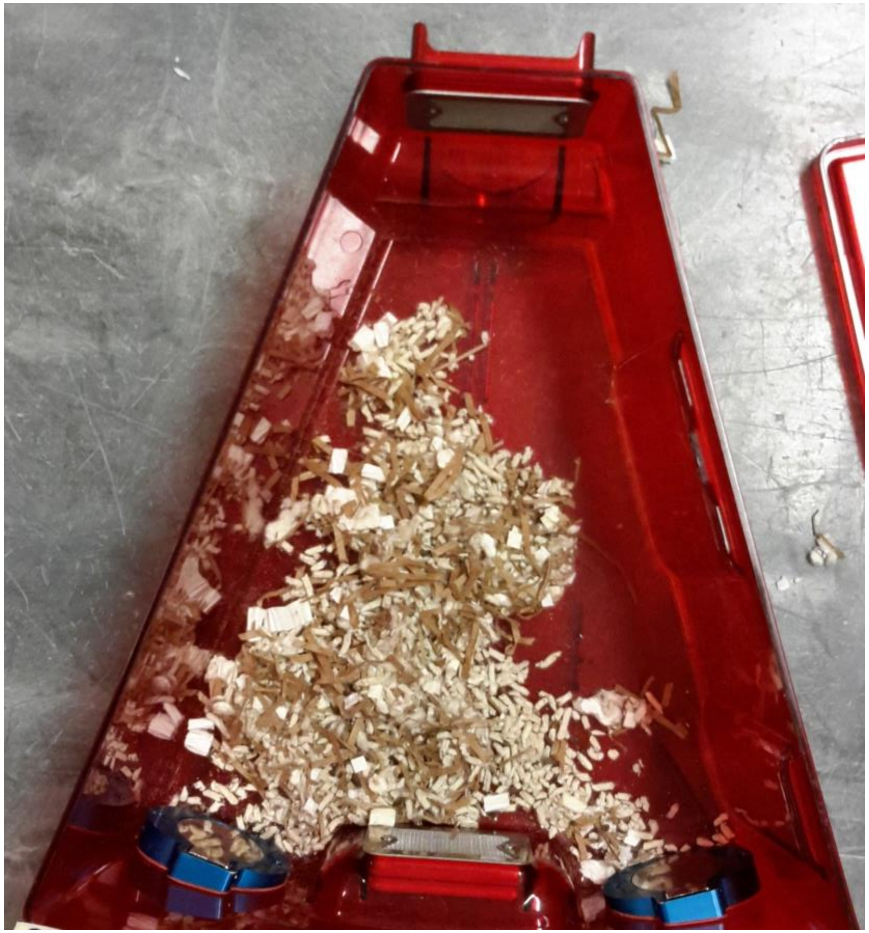

June 30 COMP 1 right

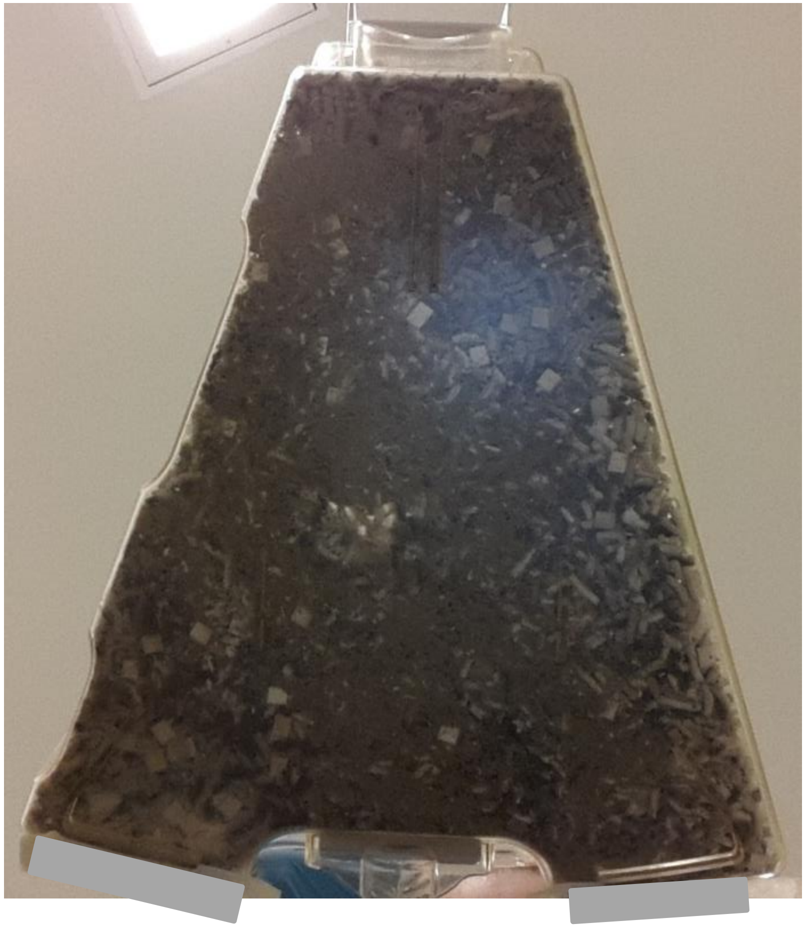

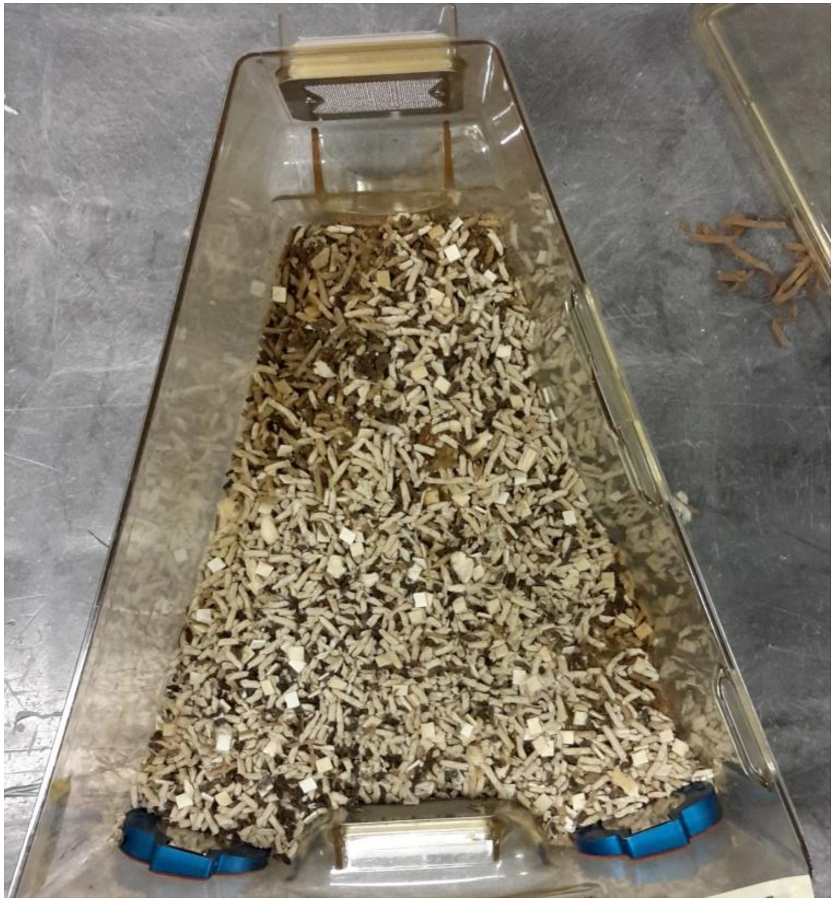

June 30 STD 1

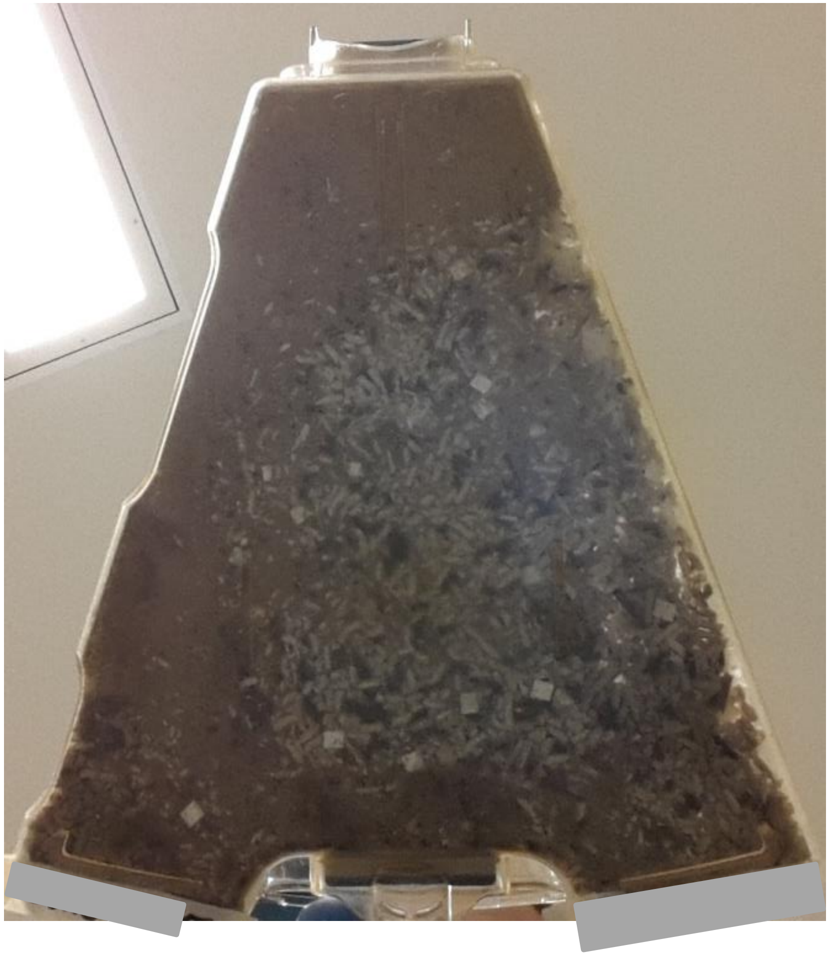

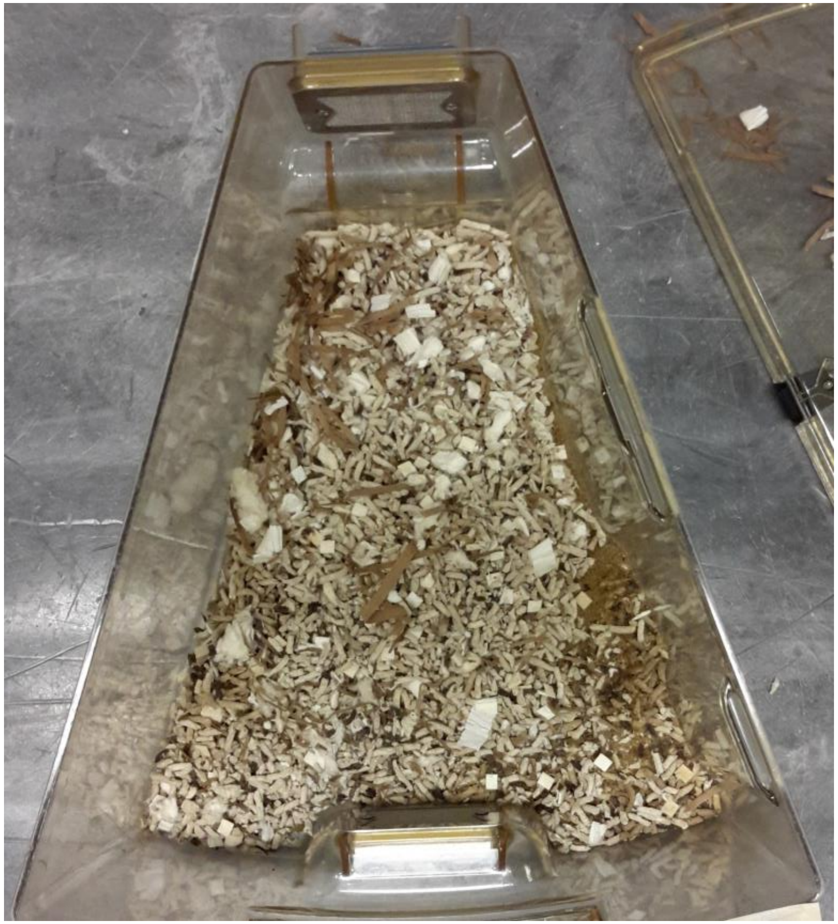

June 30 COMP 2 right

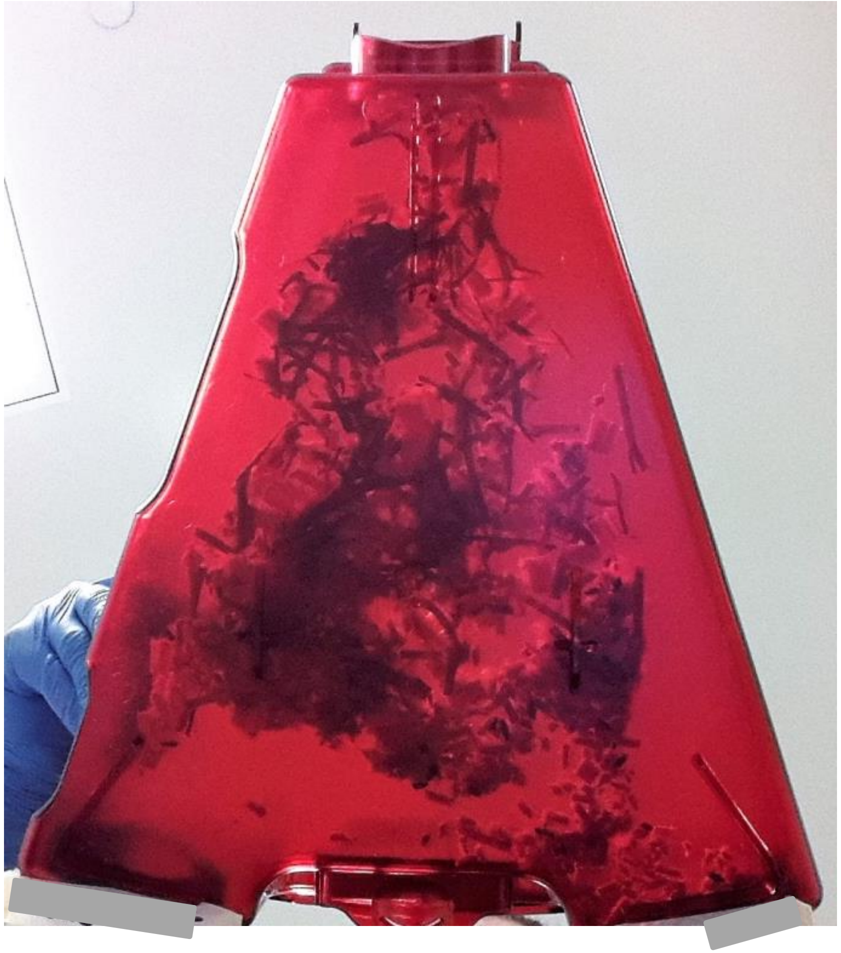

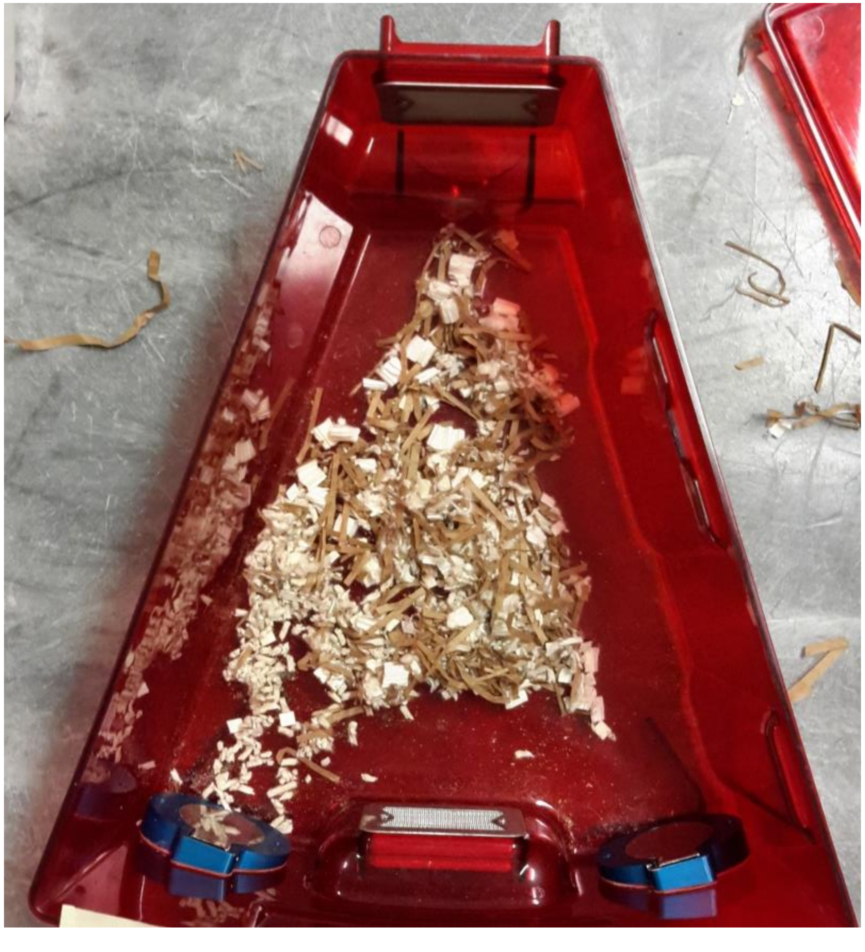

June 30 COMP 2 left

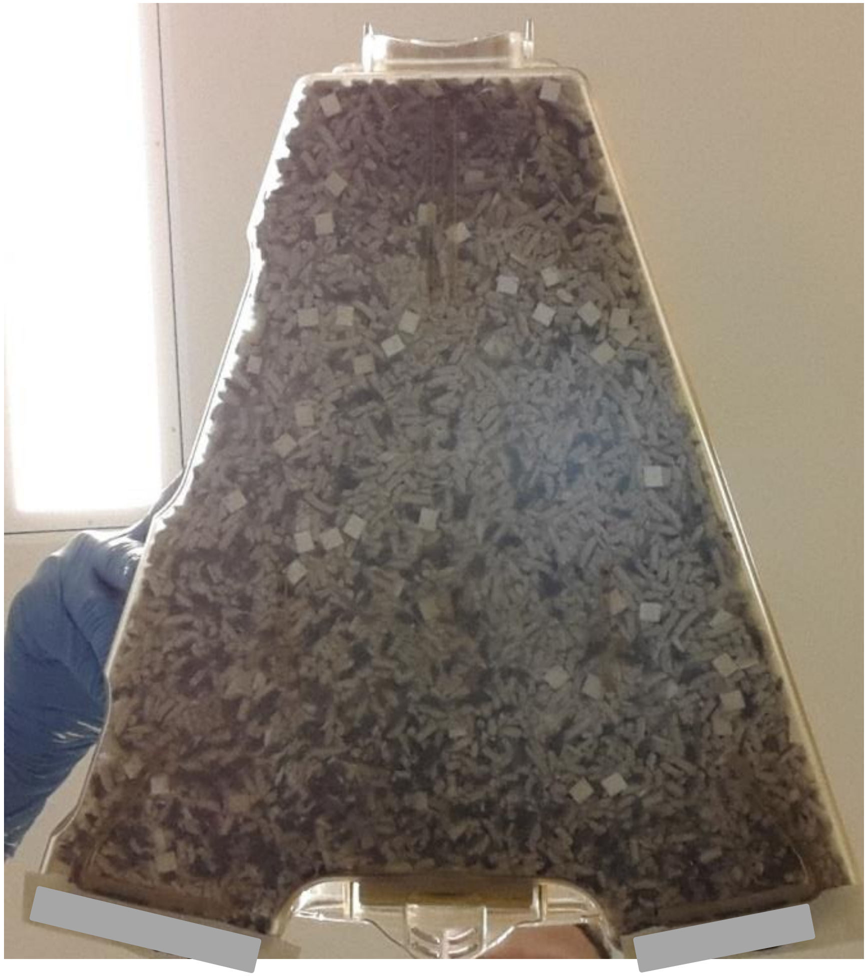

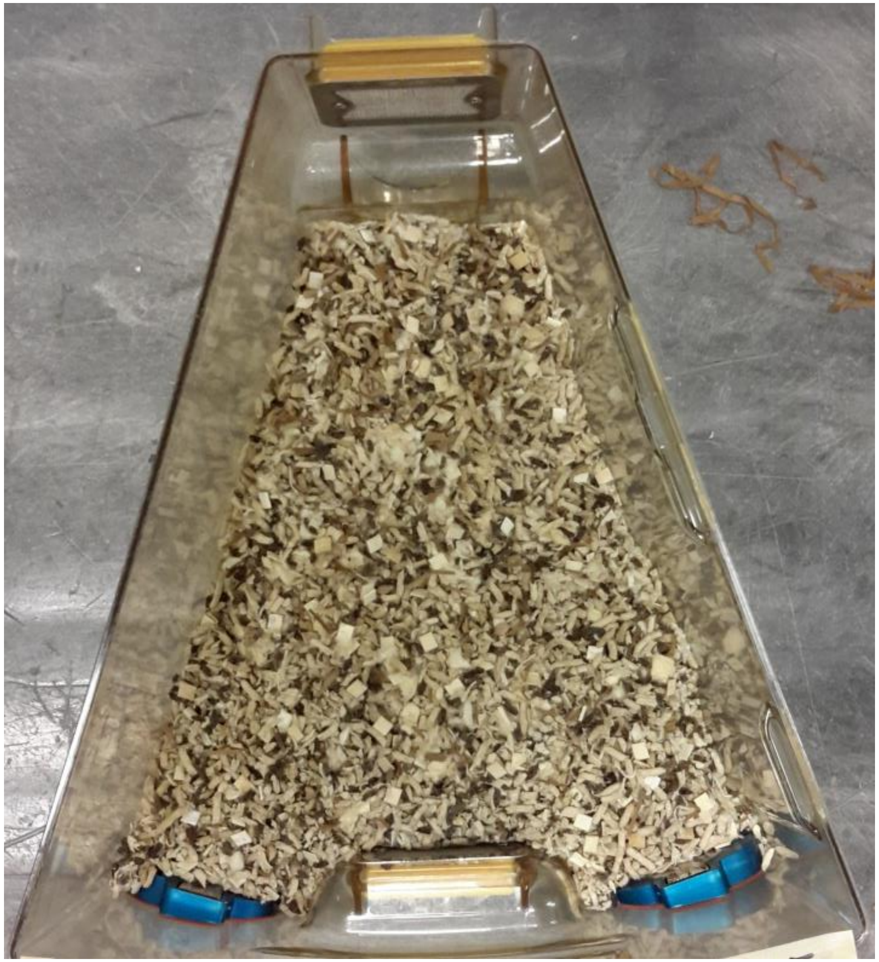

June 30 COMP 2 mid

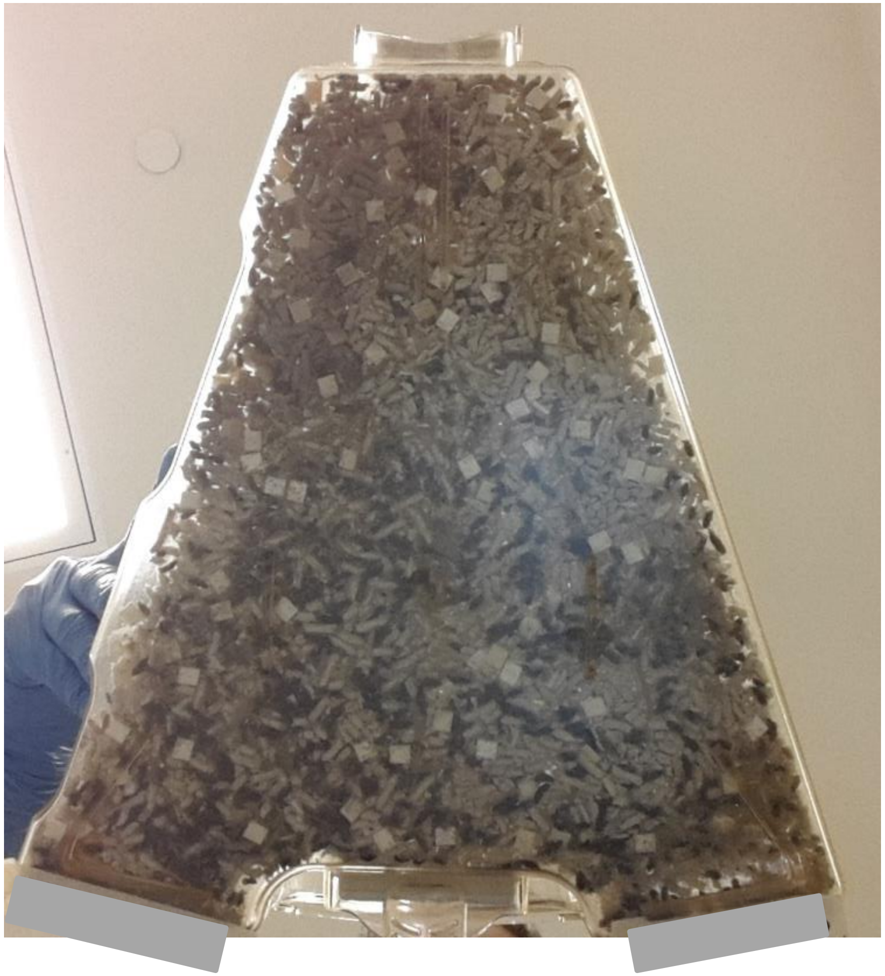

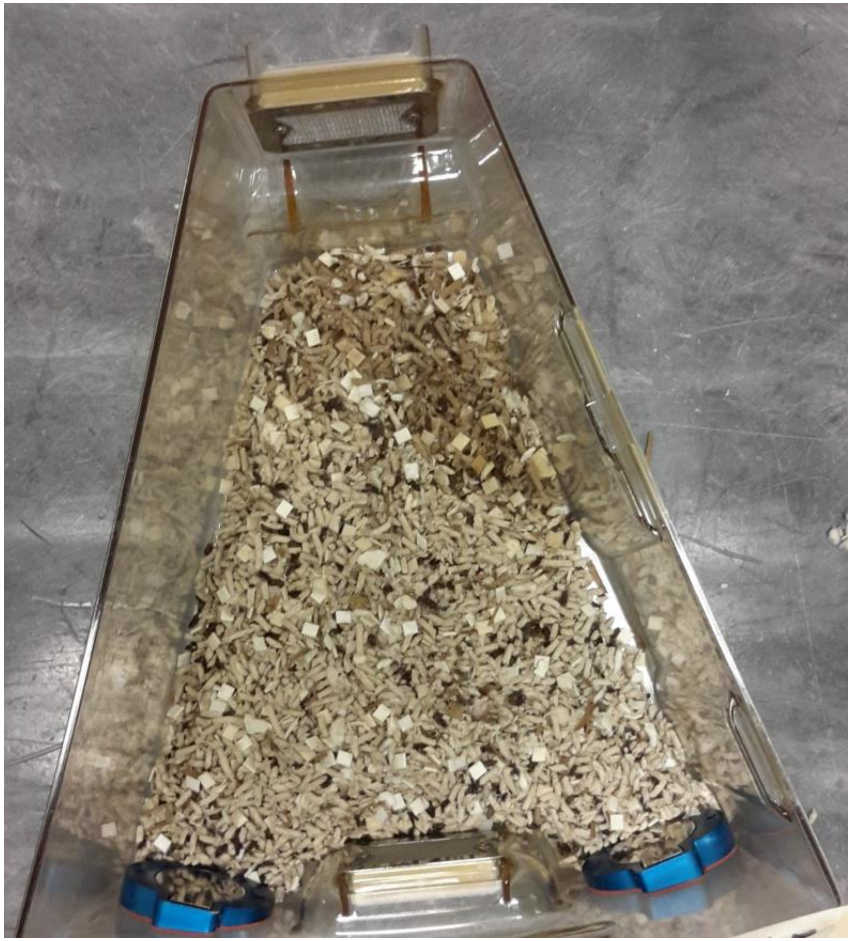

June 30 STD 2

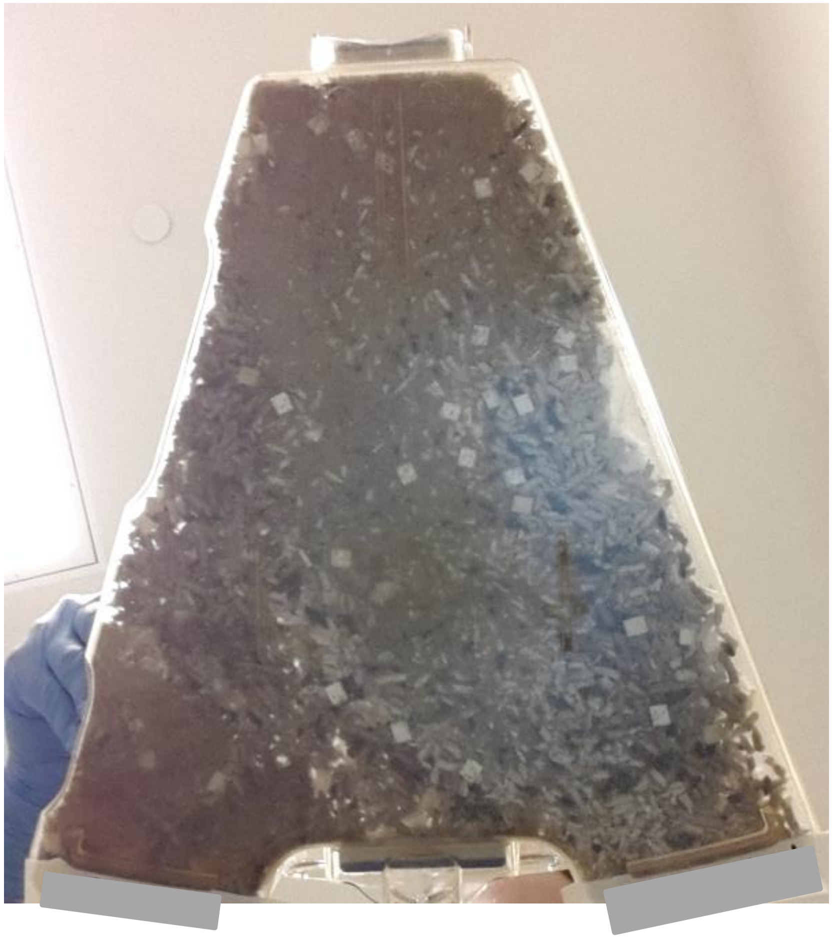

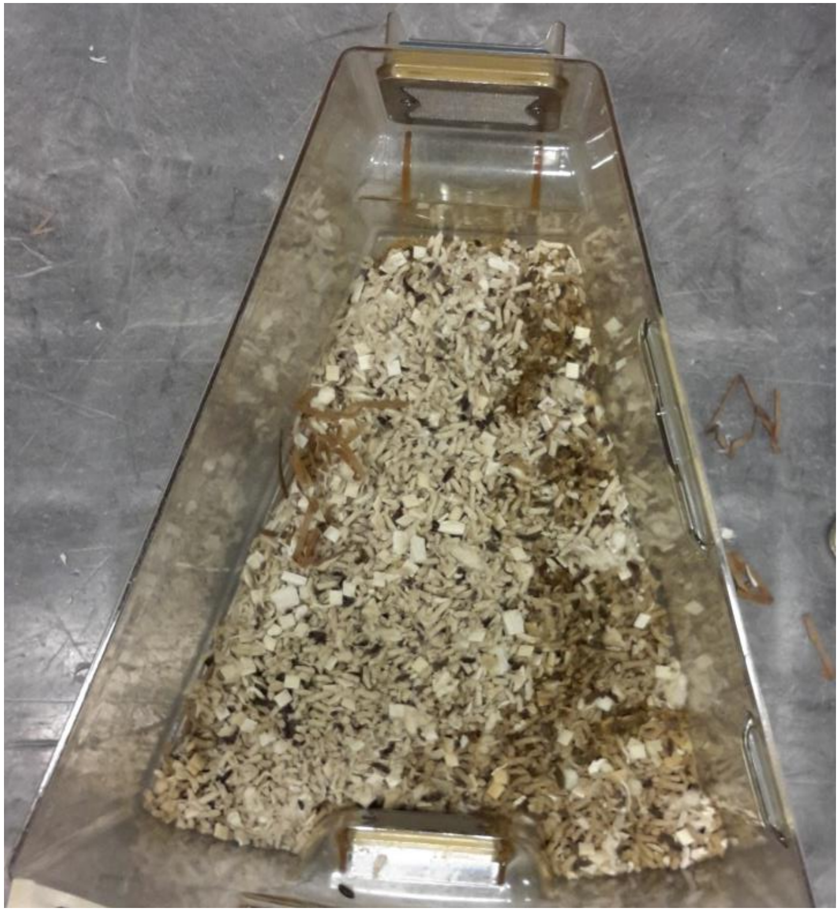

July 1 COMP 3 left

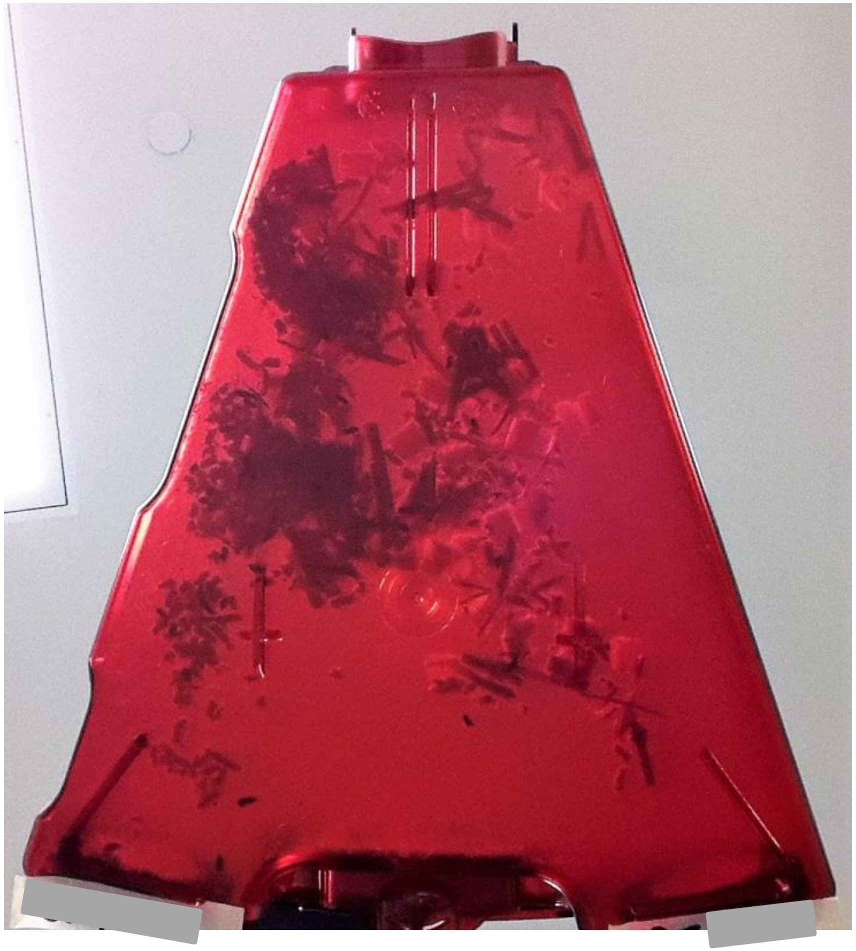

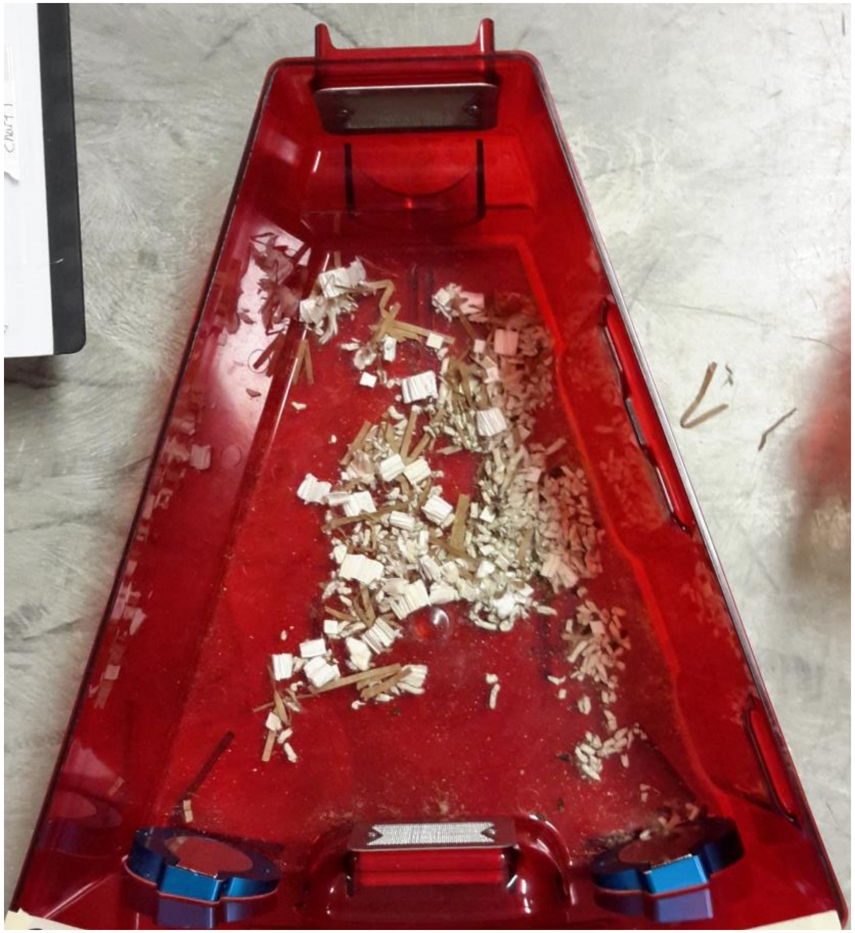

July 1 COMP 3 mid

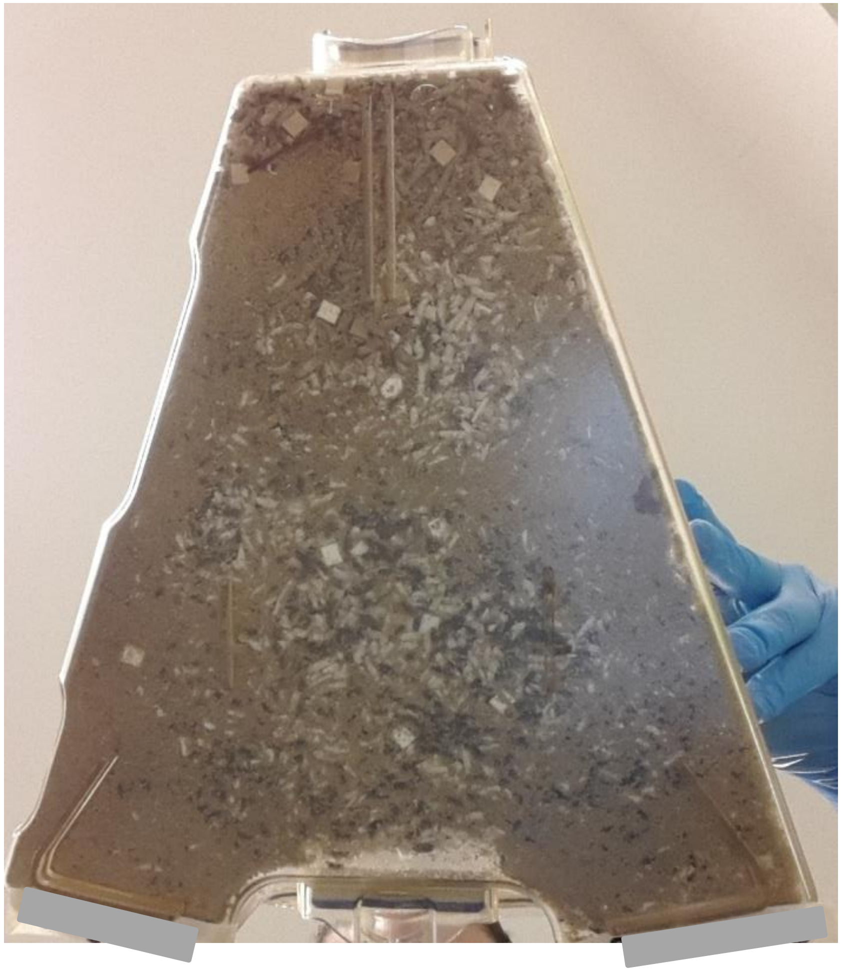

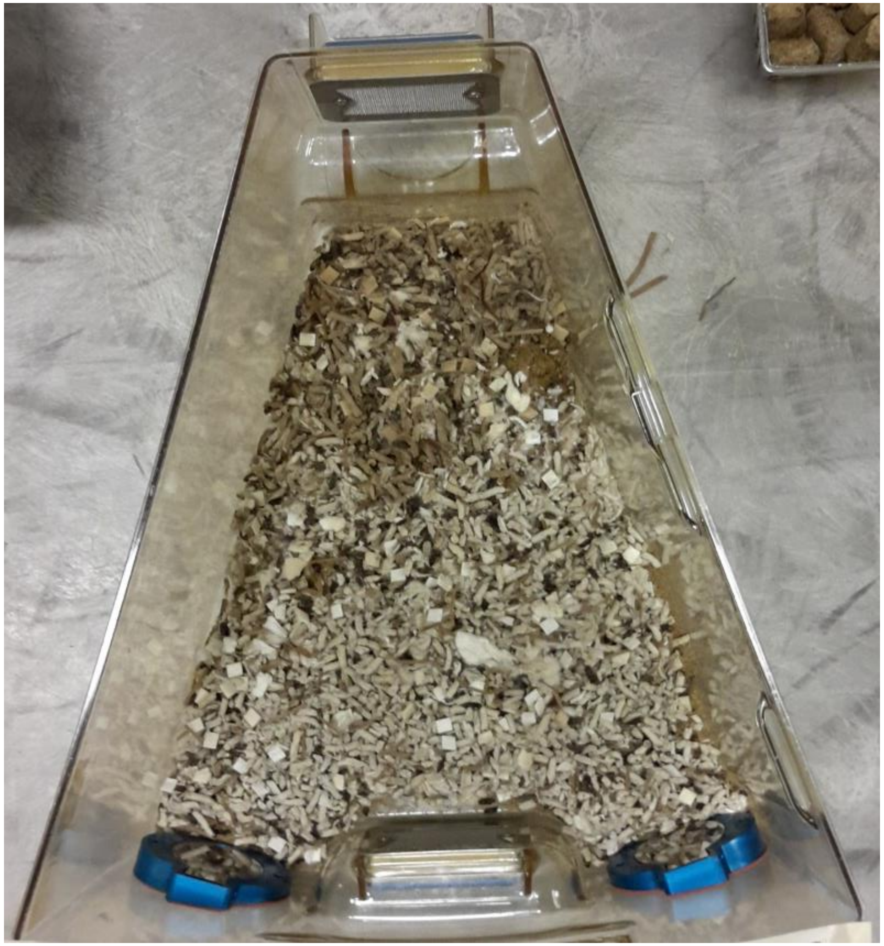

July 1 COMP 3 right

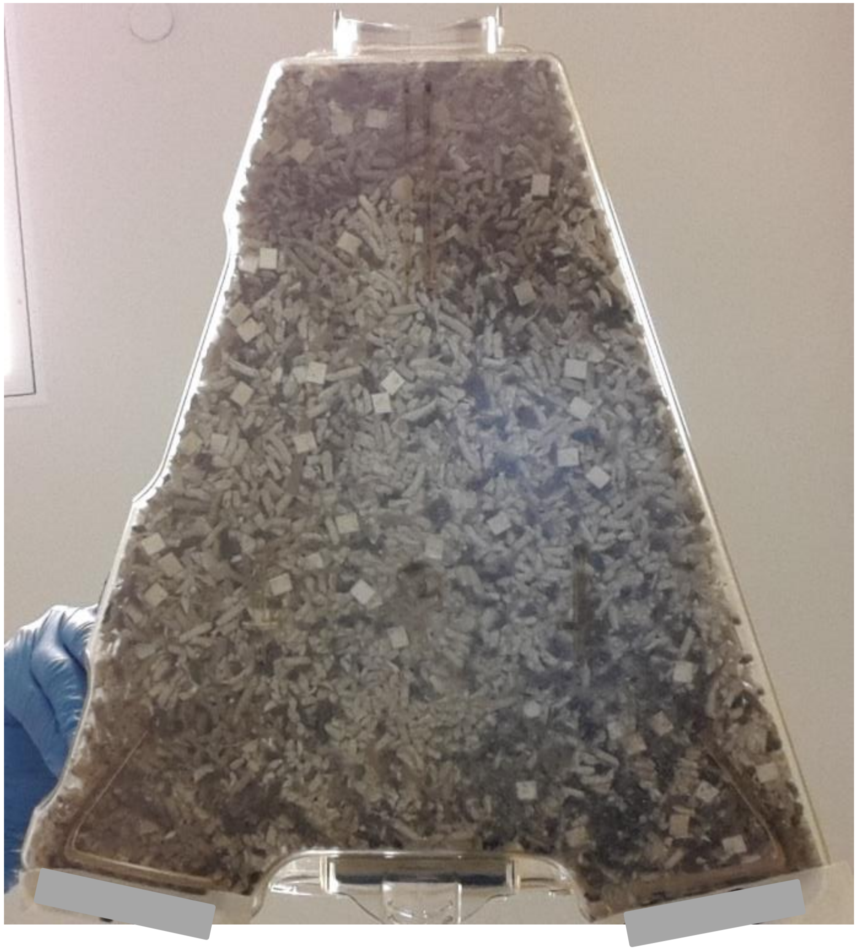

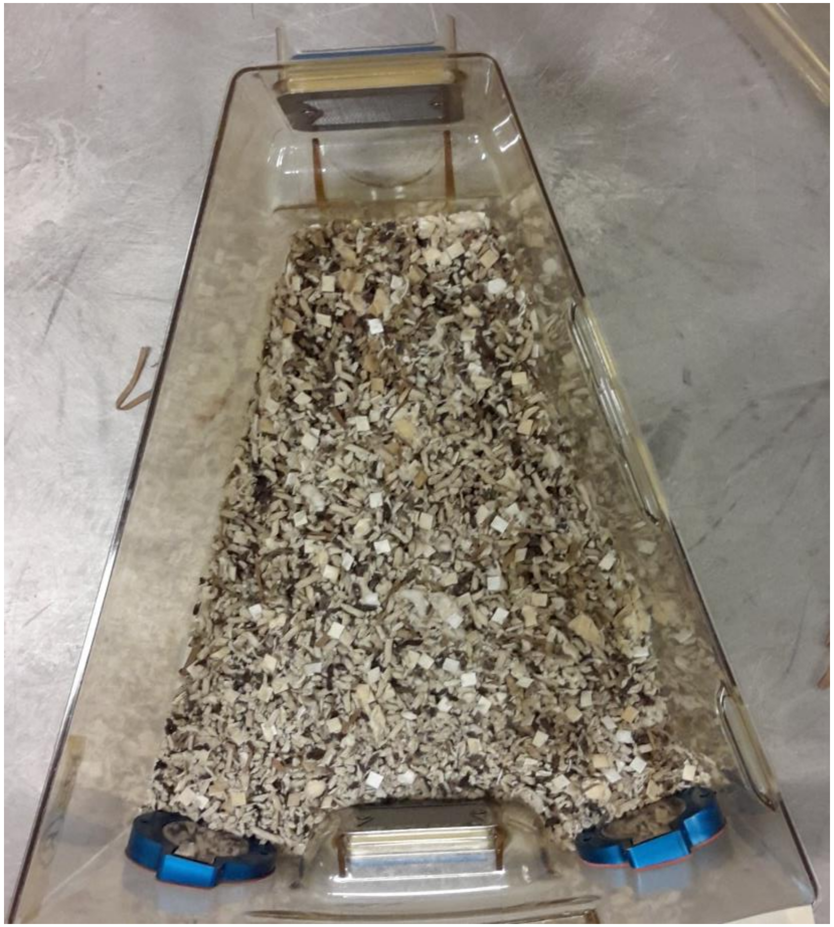

July 1 STD 3

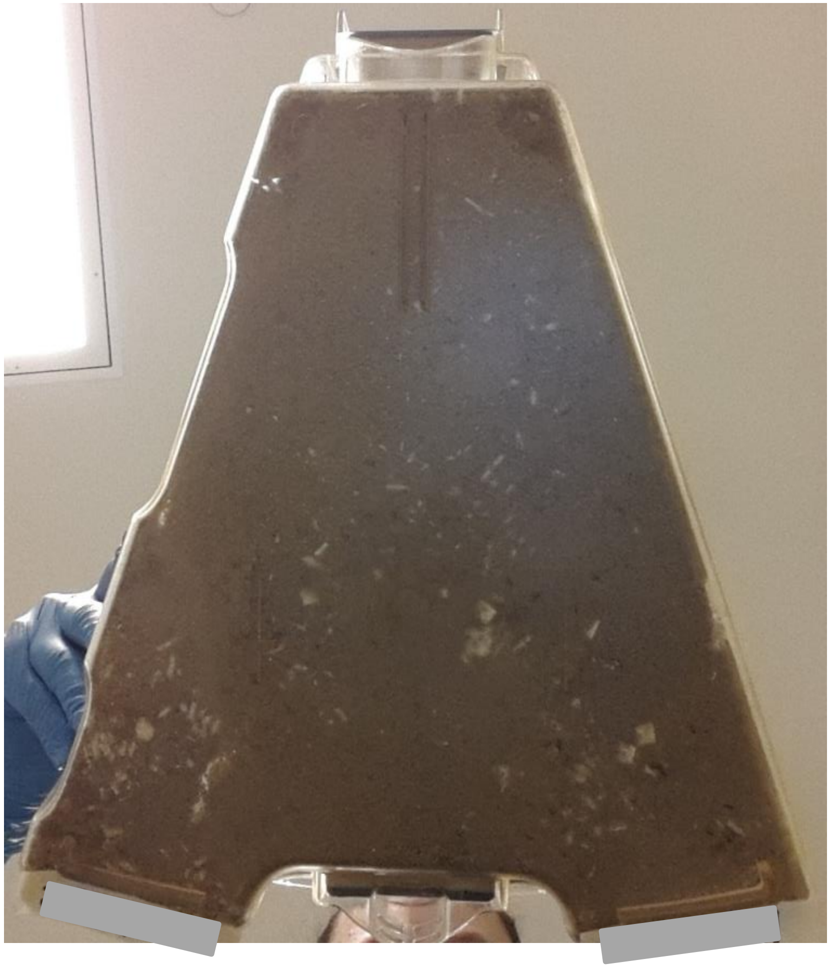

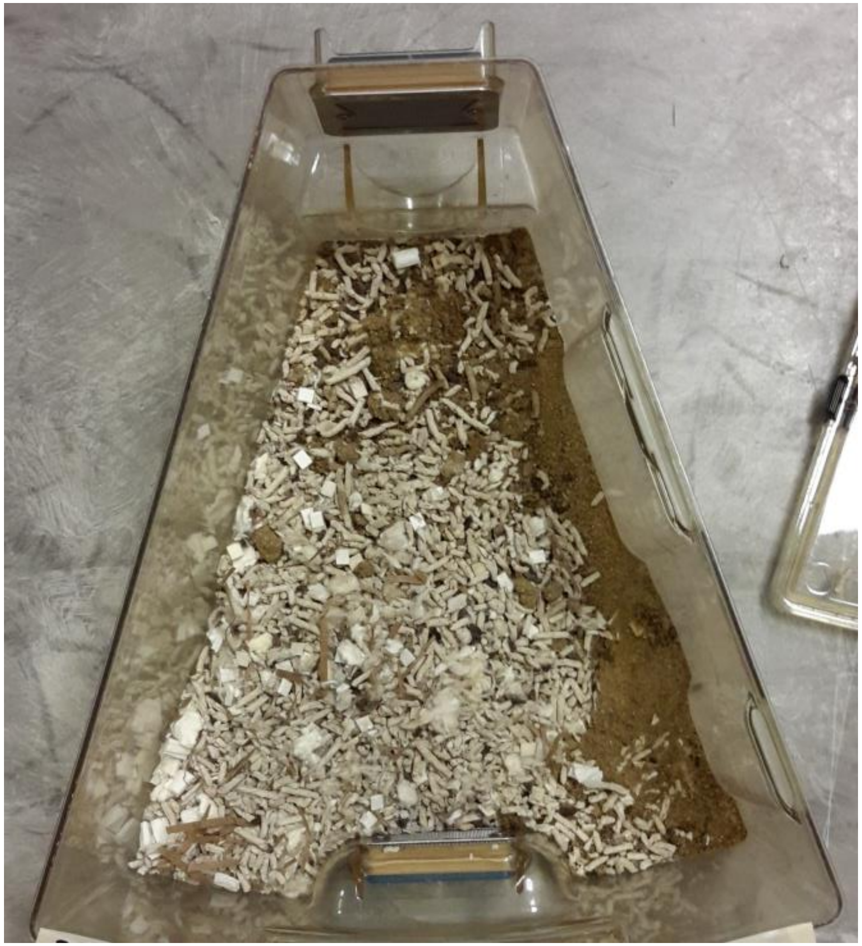

July 1 COMP 4 right

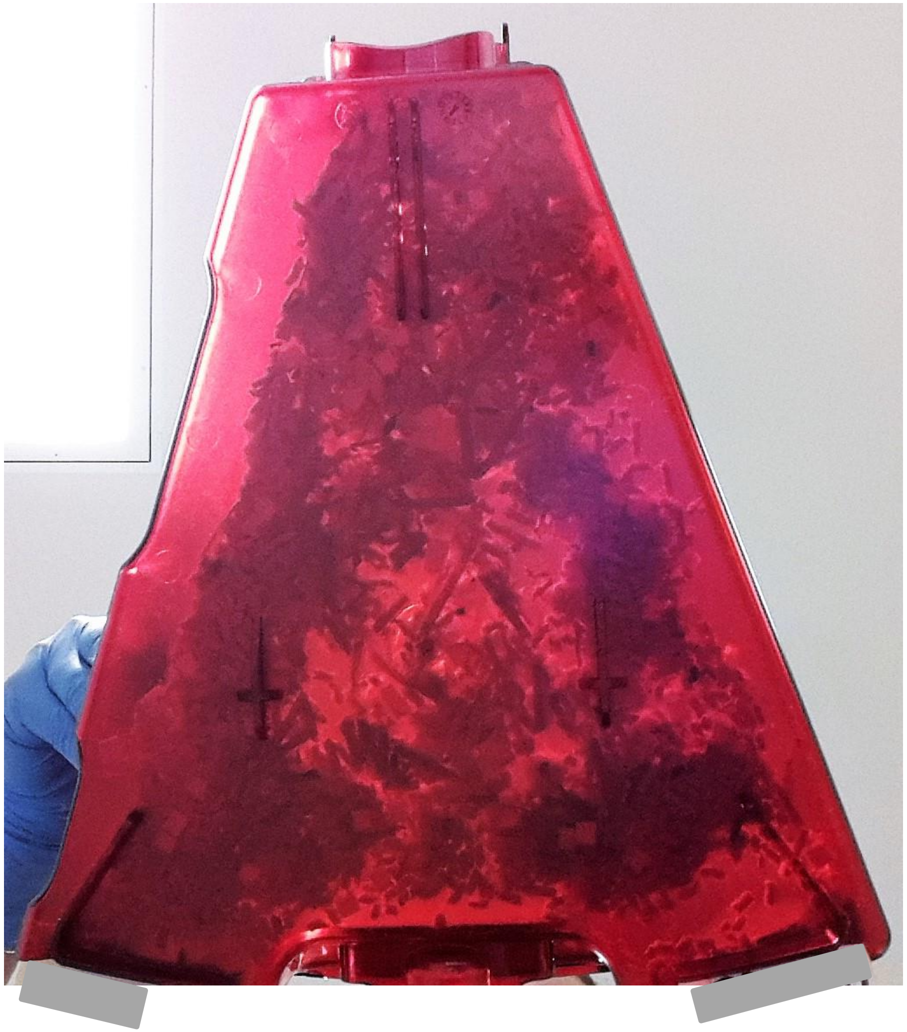

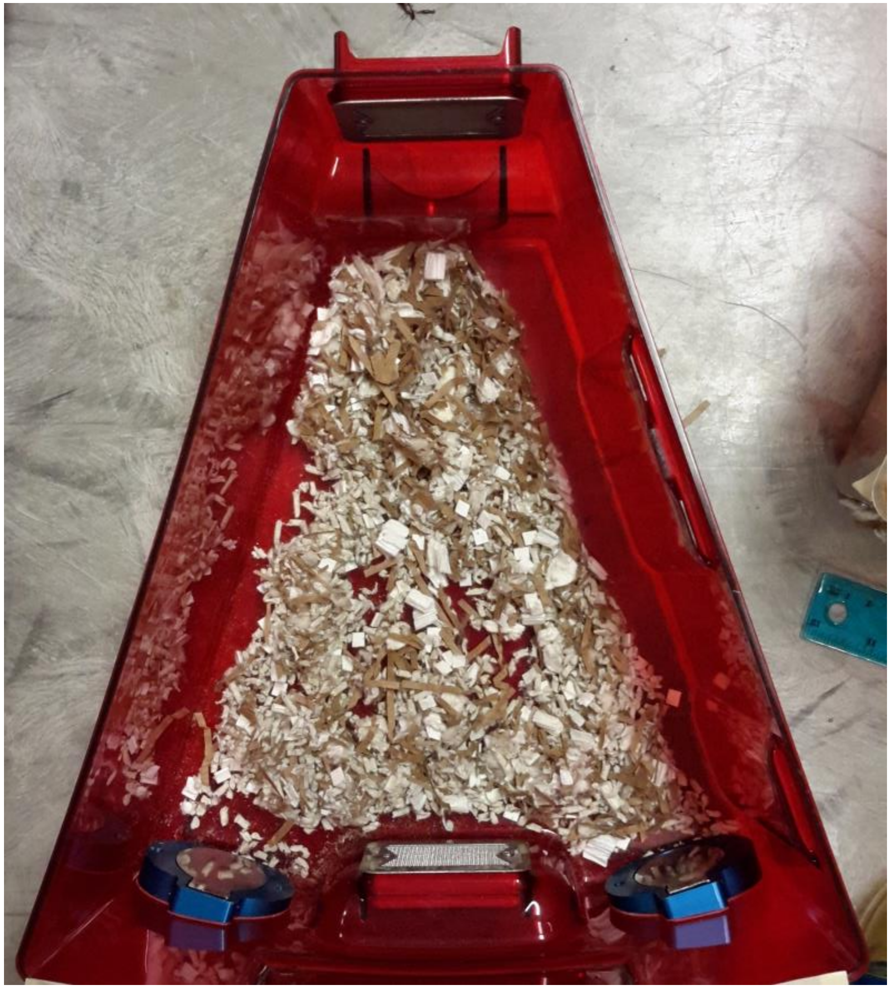

July 1 COMP 4 mid

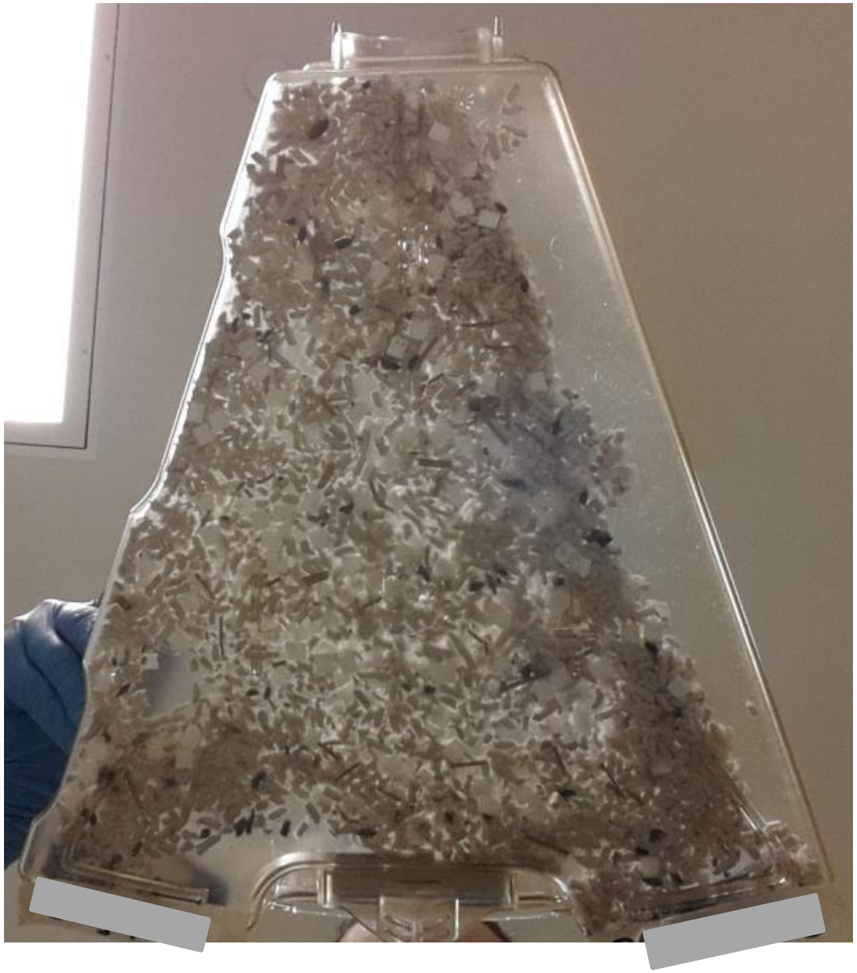

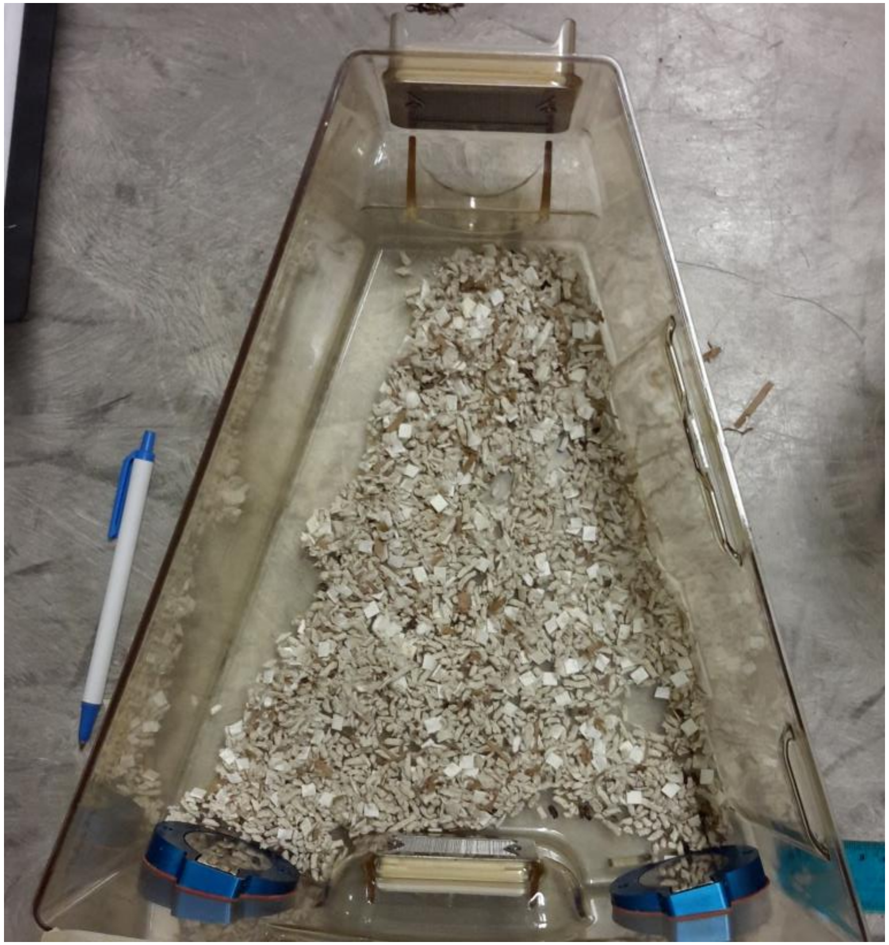

July 1 COMP 4 left

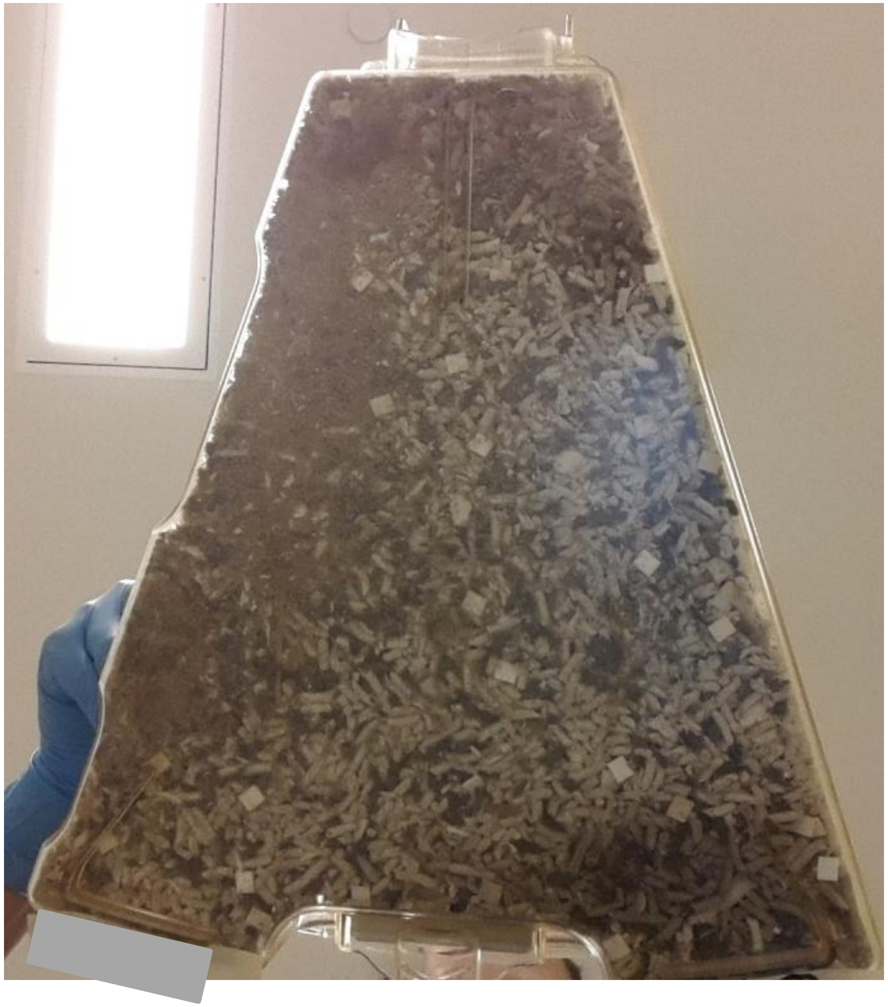

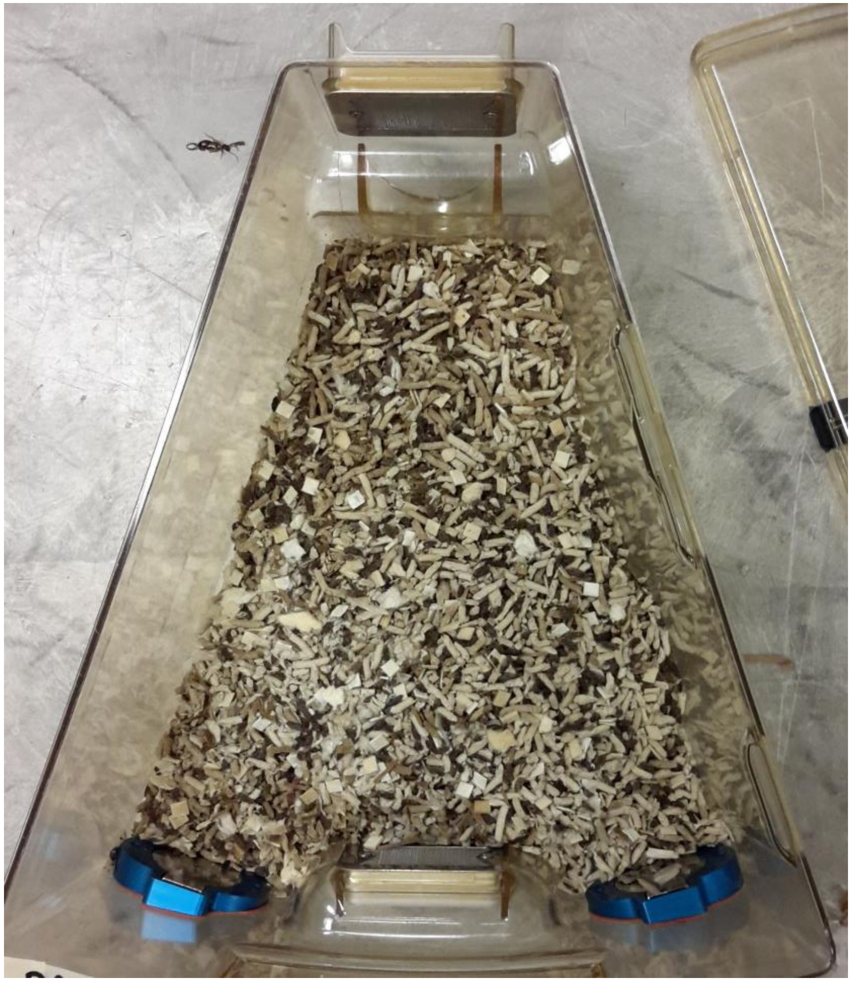

July 1 STD 4

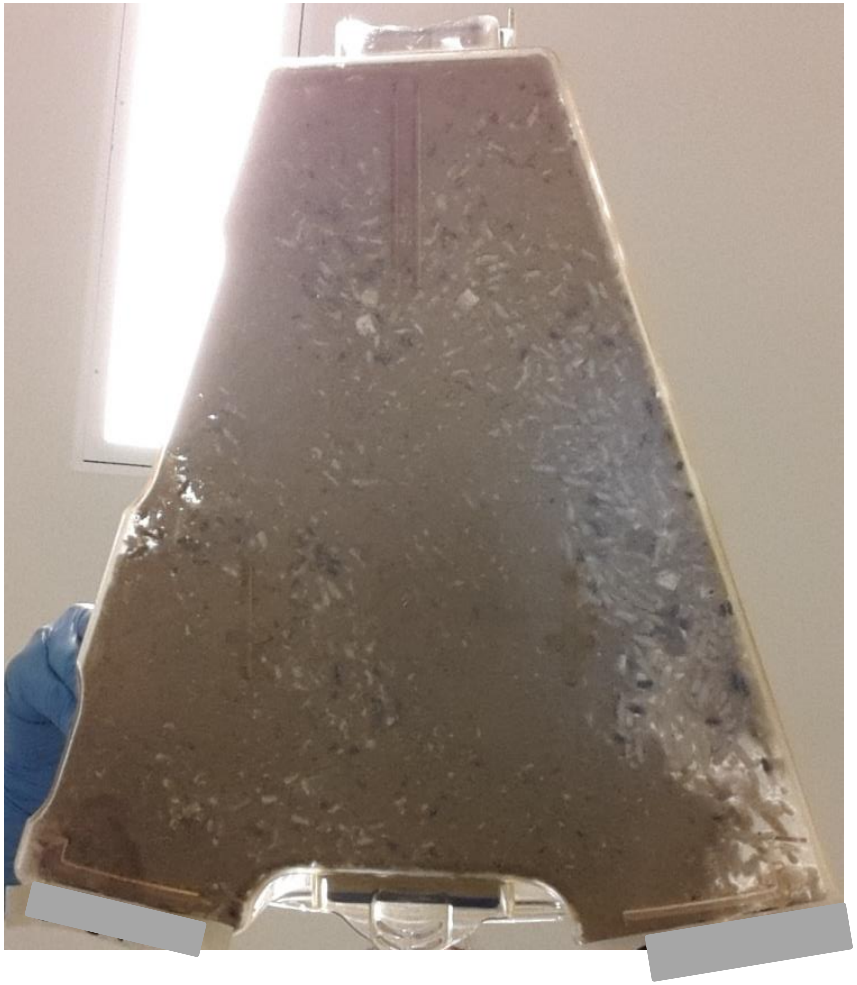

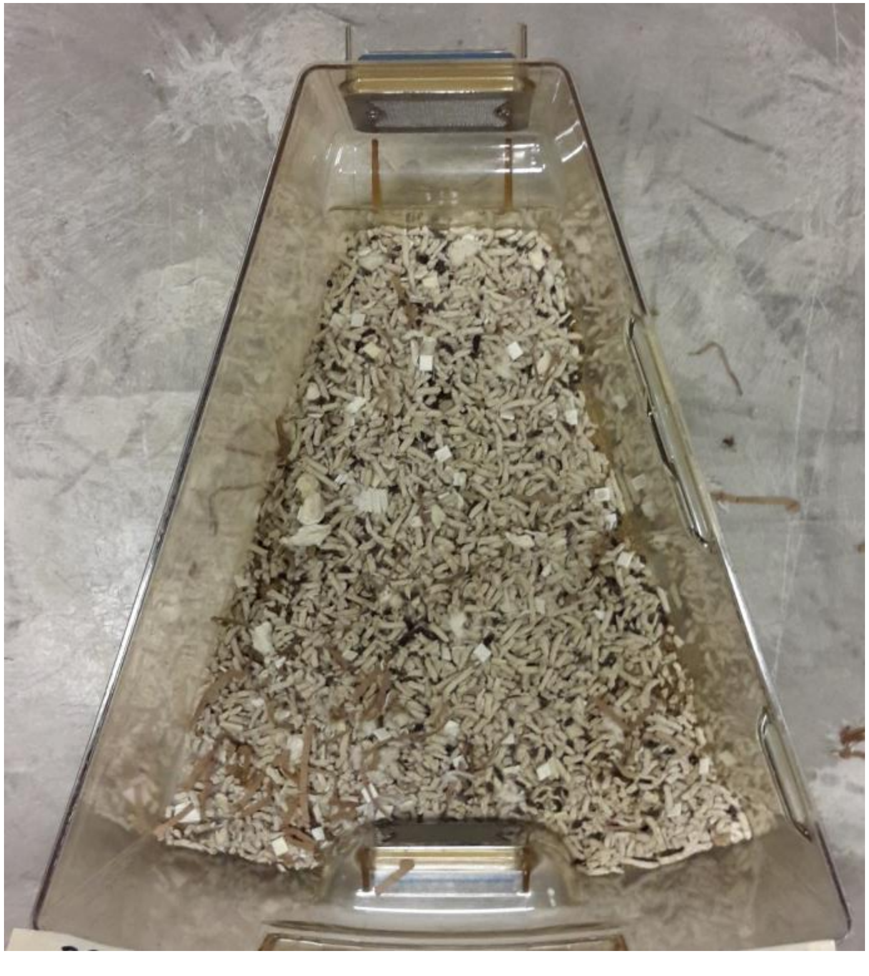

July 2 COMP 5 left

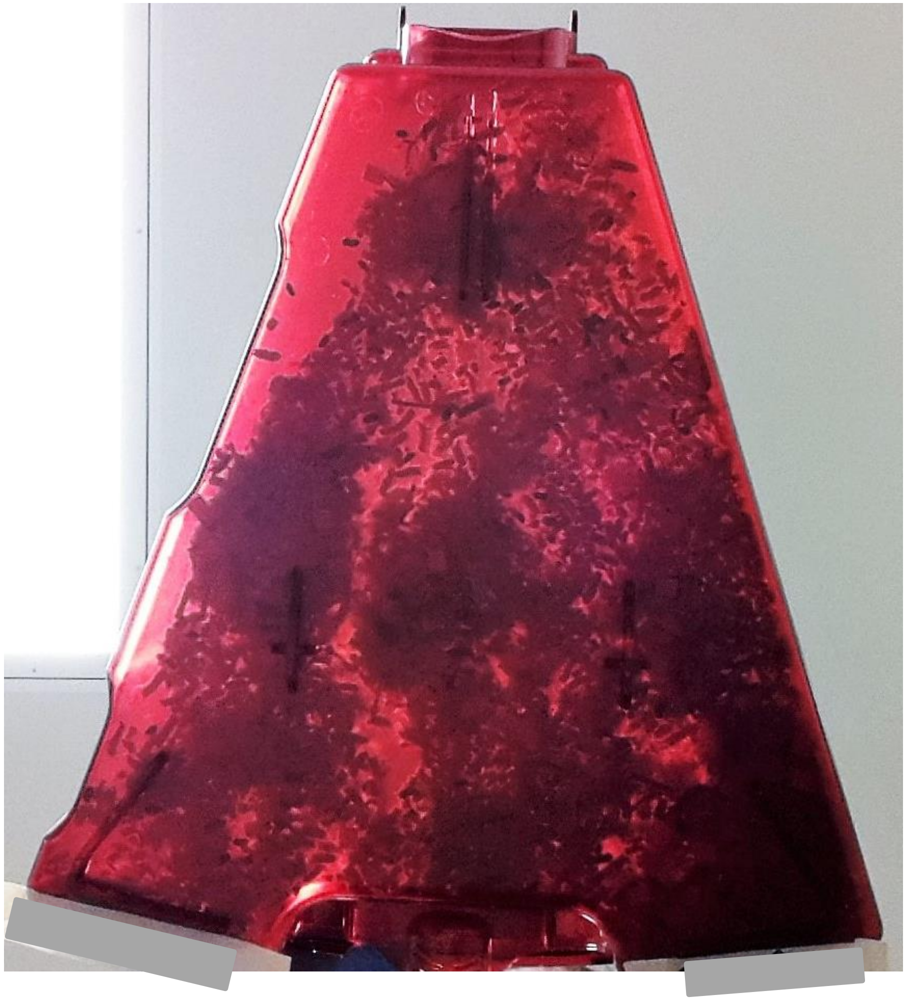

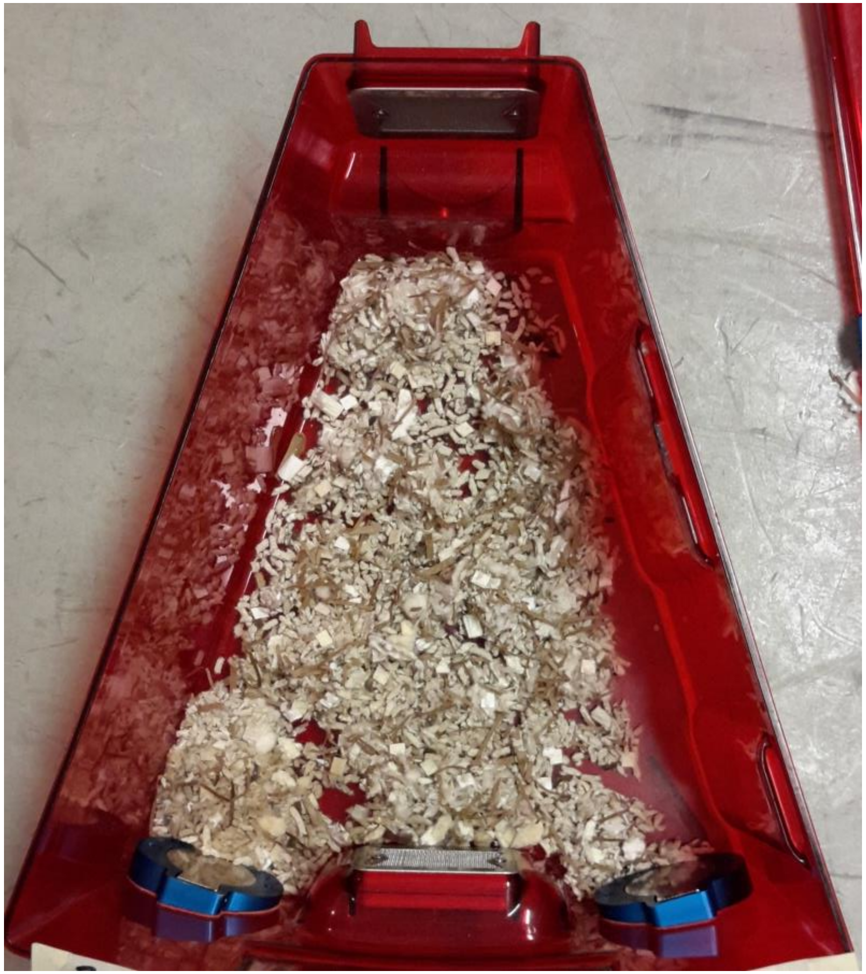

July 2 COMP 5 mid

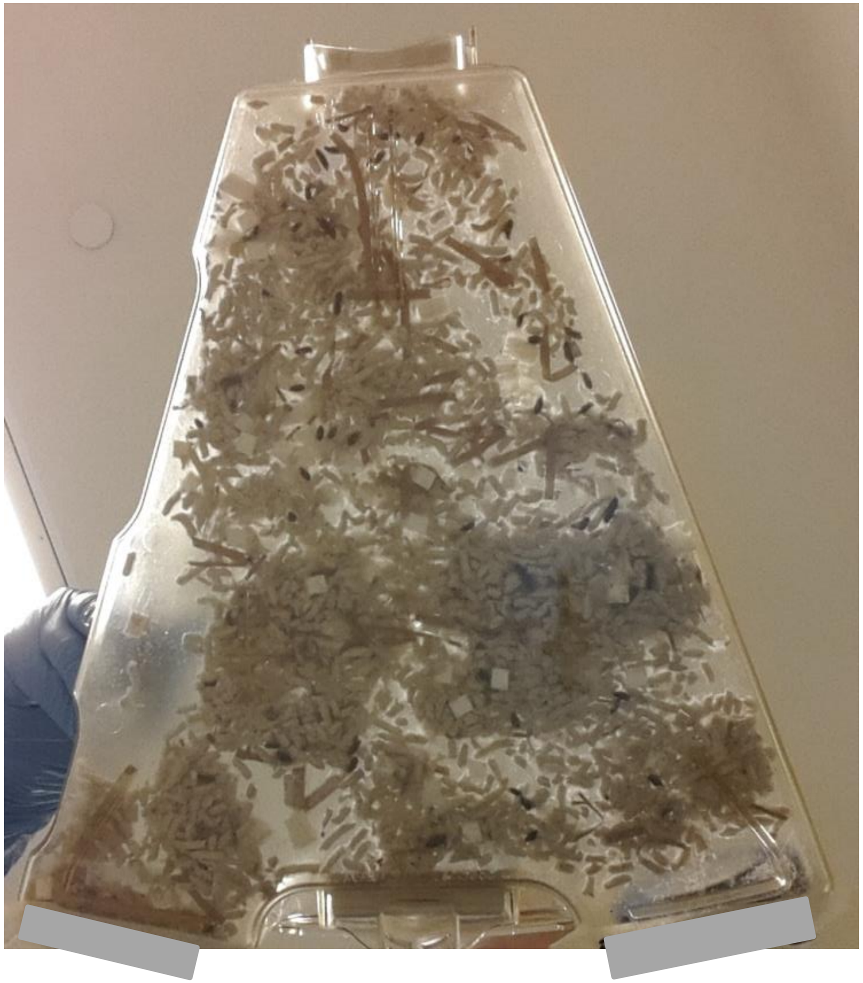

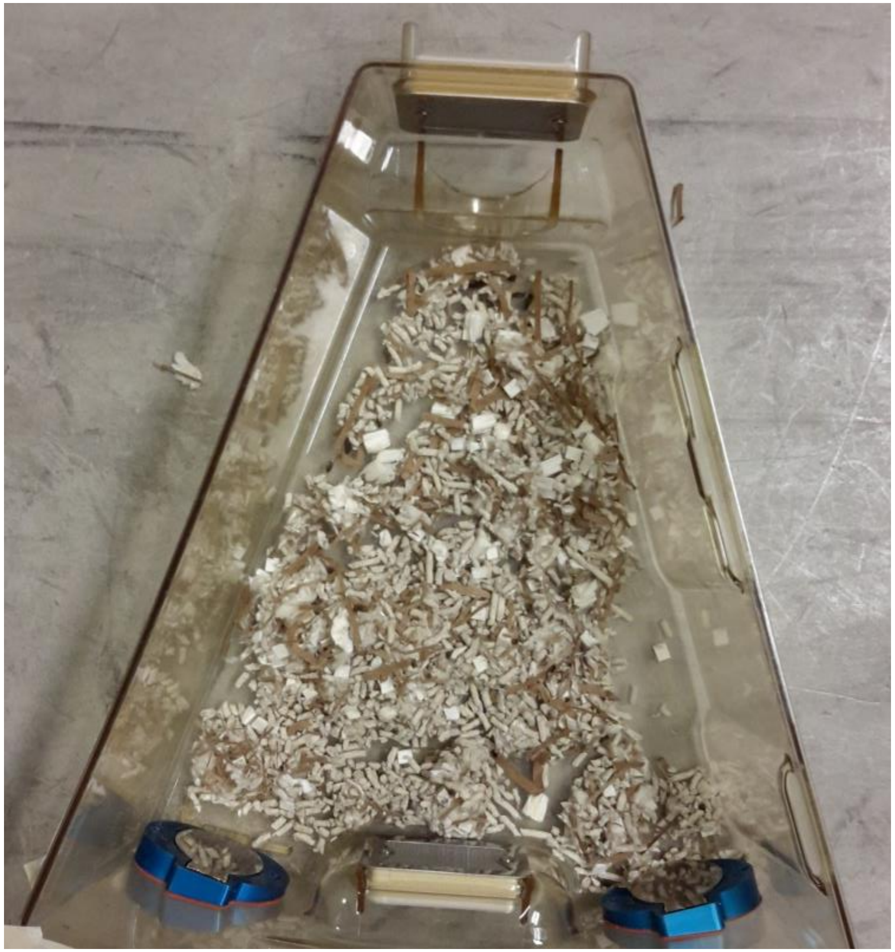

July 2 COMP 5 right

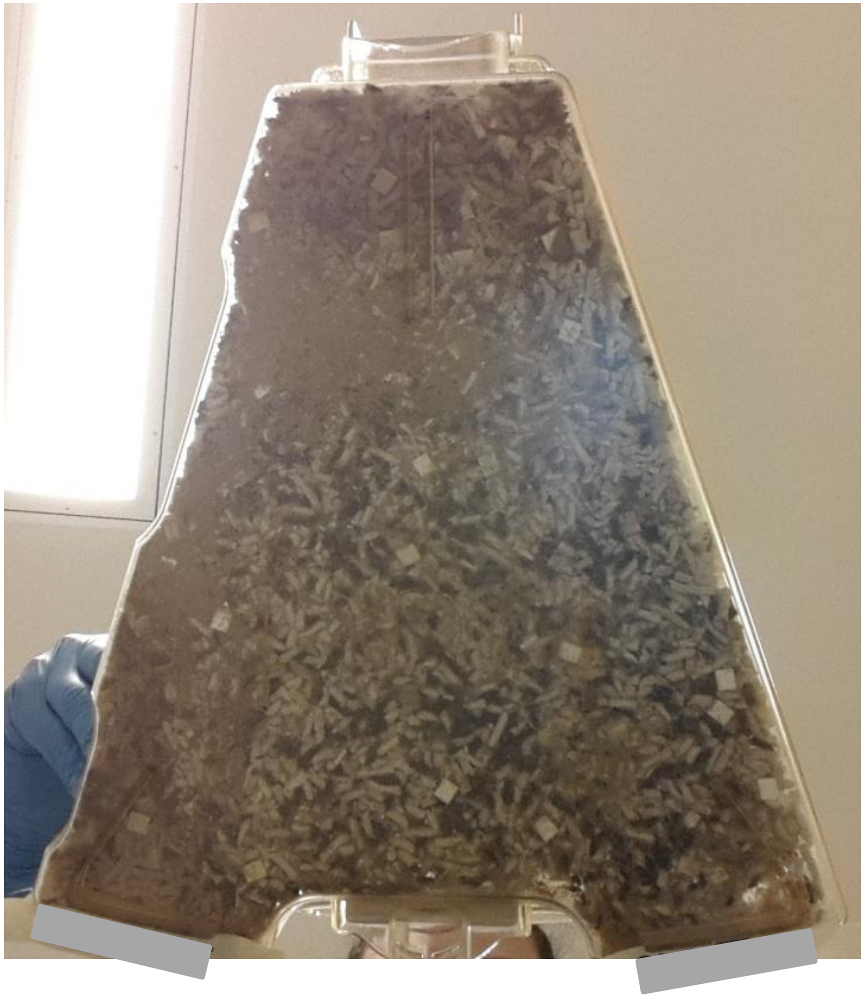

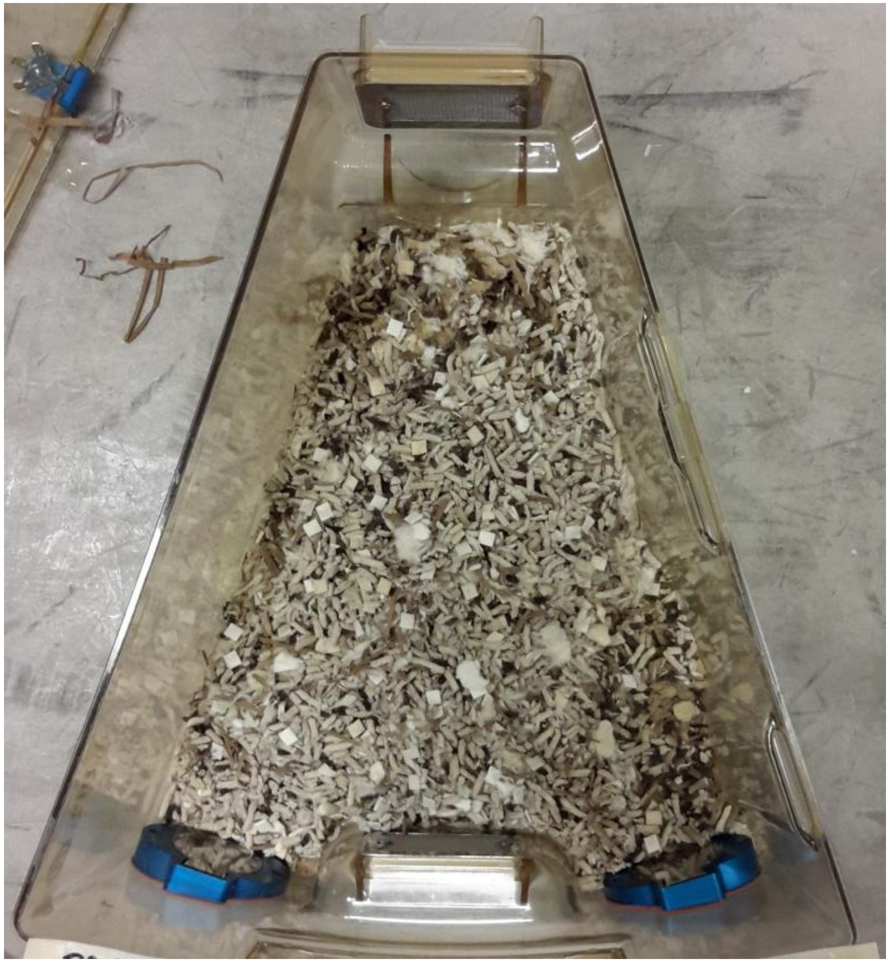

July 2 STD 5

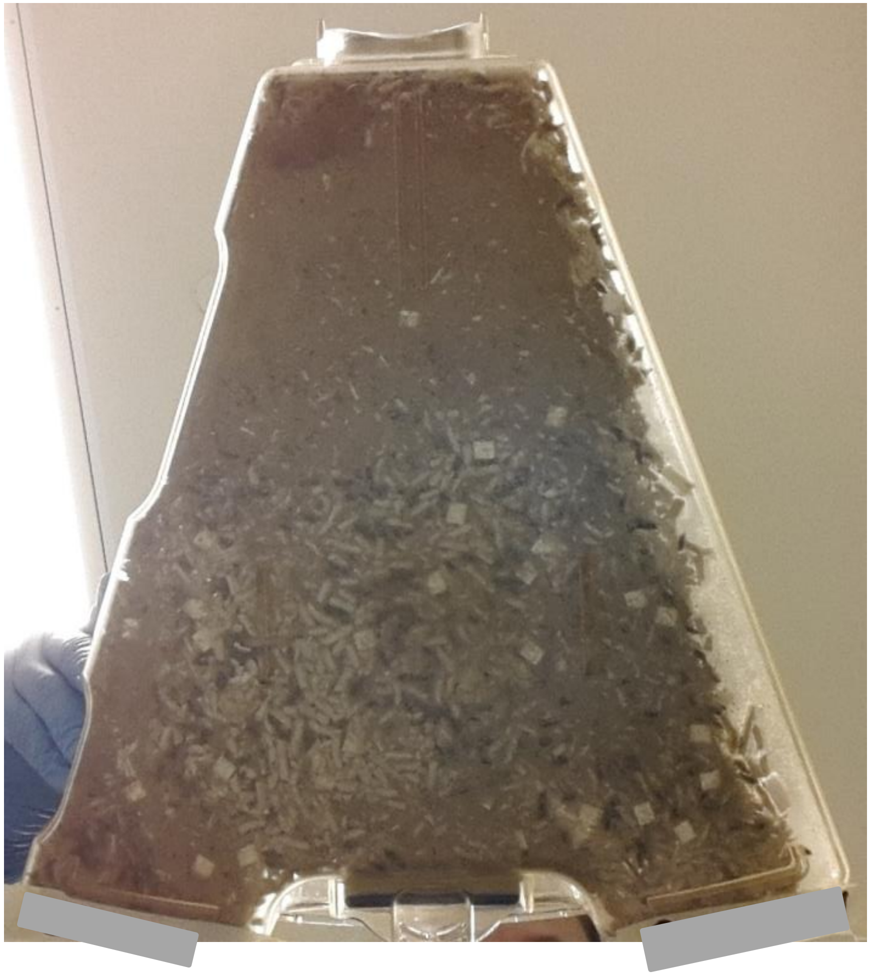

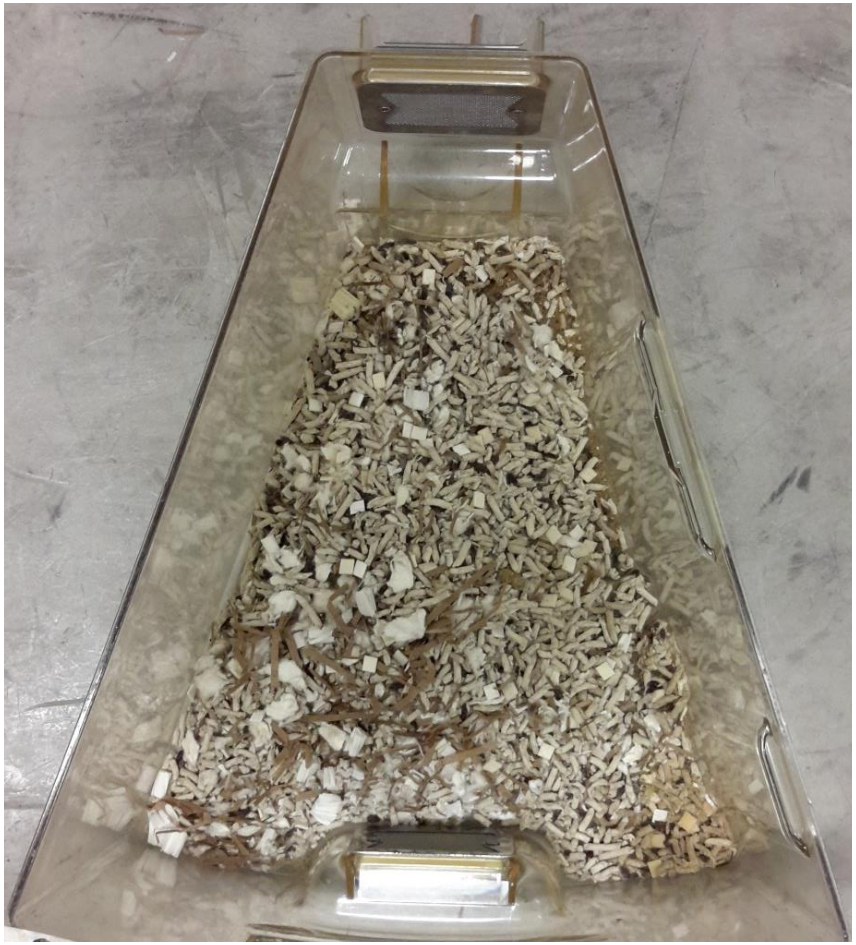

July 7 COMP 1 mid

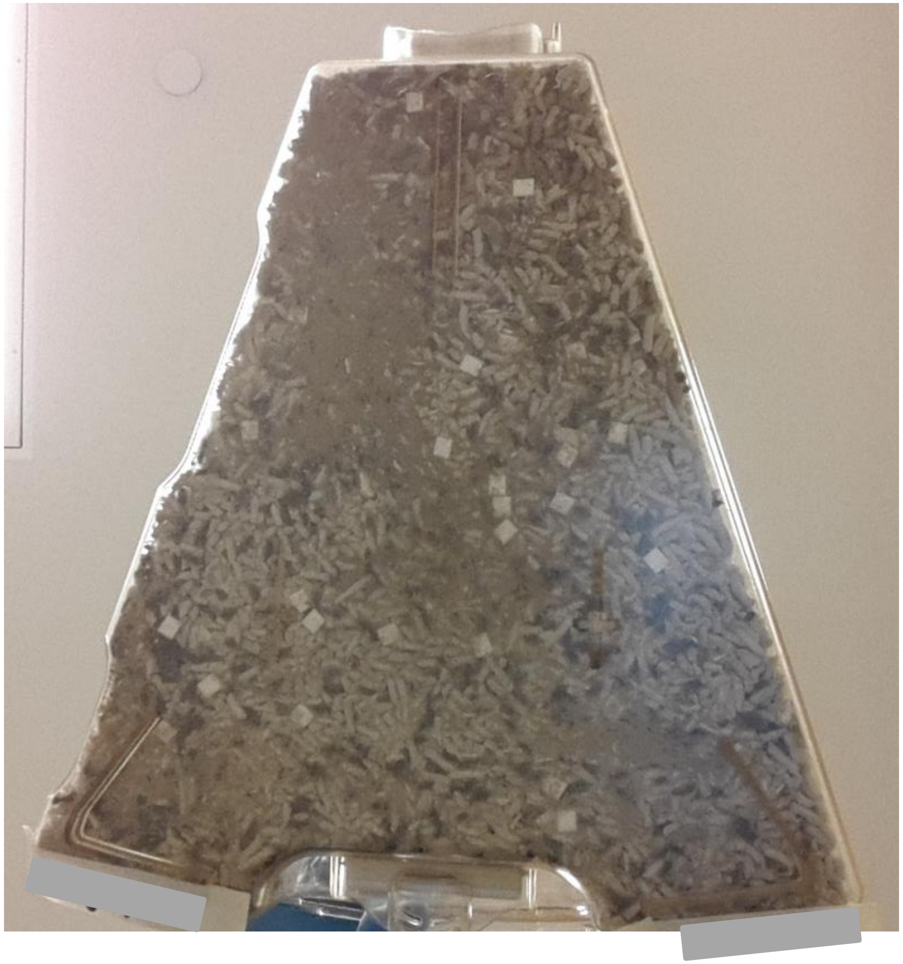

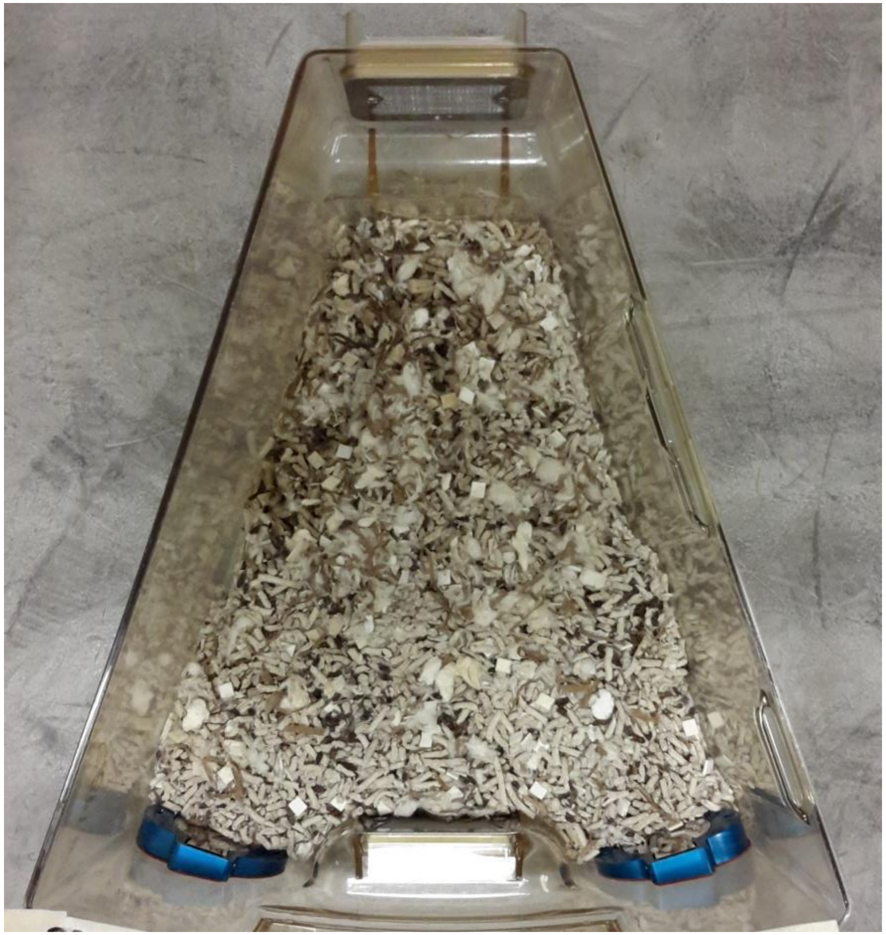

July 7 COMP 1 left

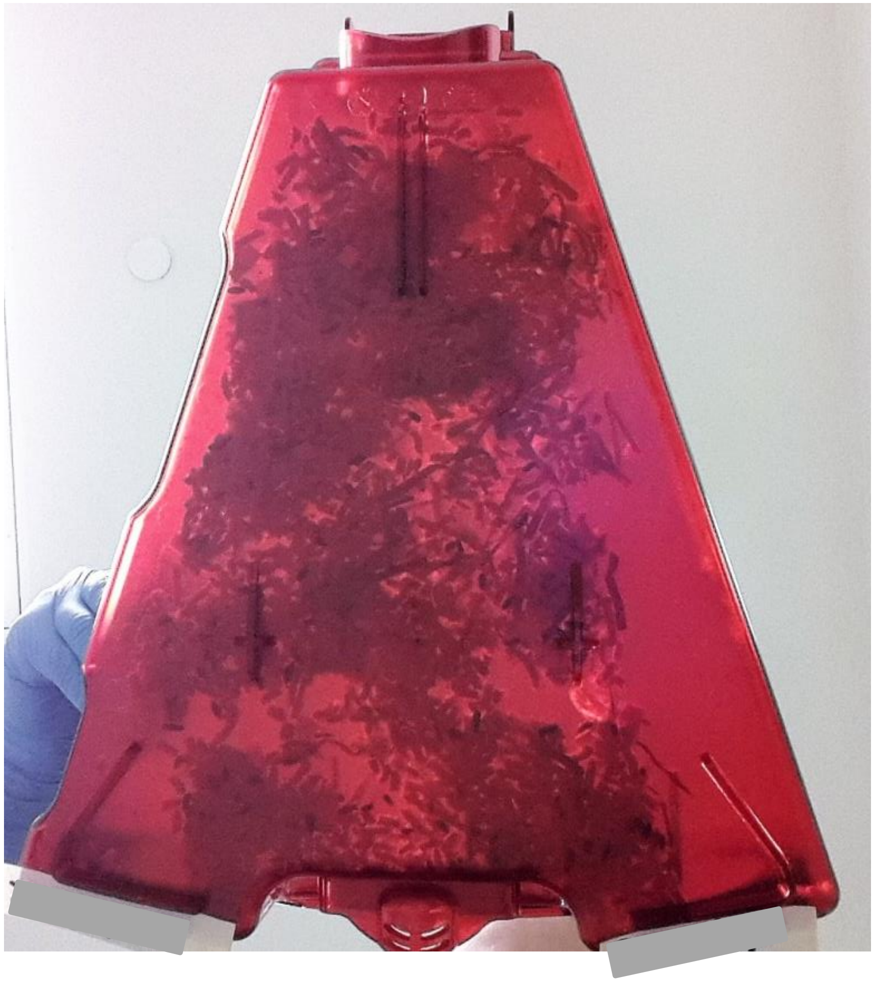

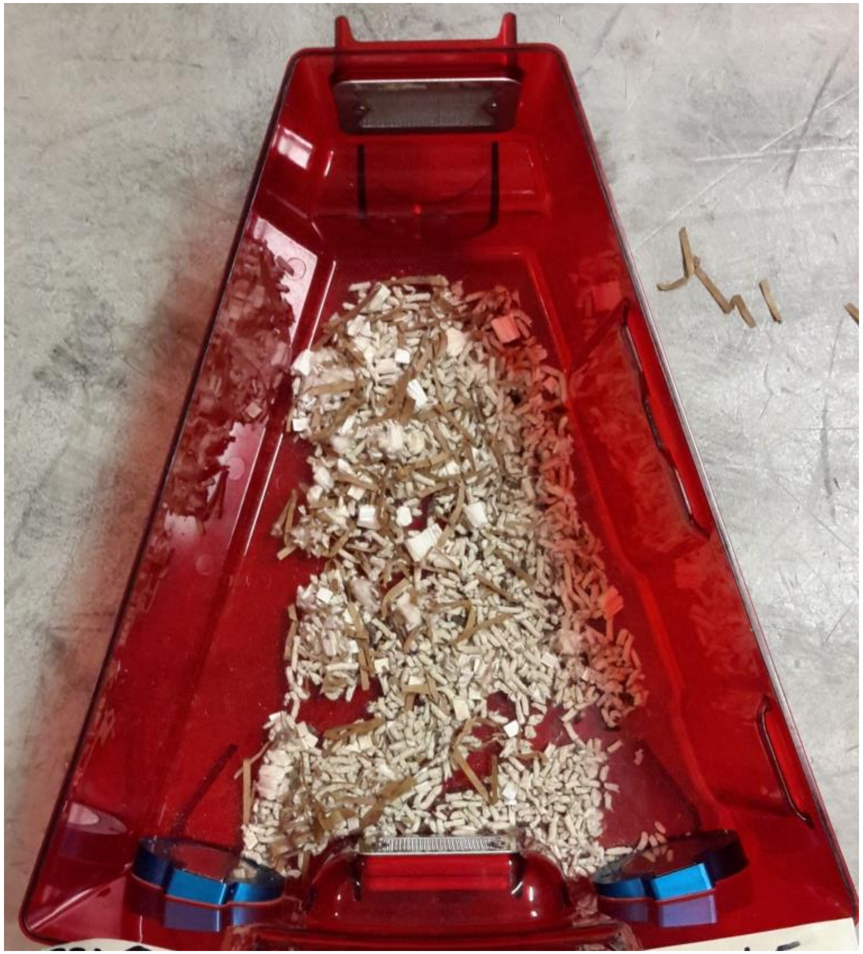

July 7 COMP 1 right

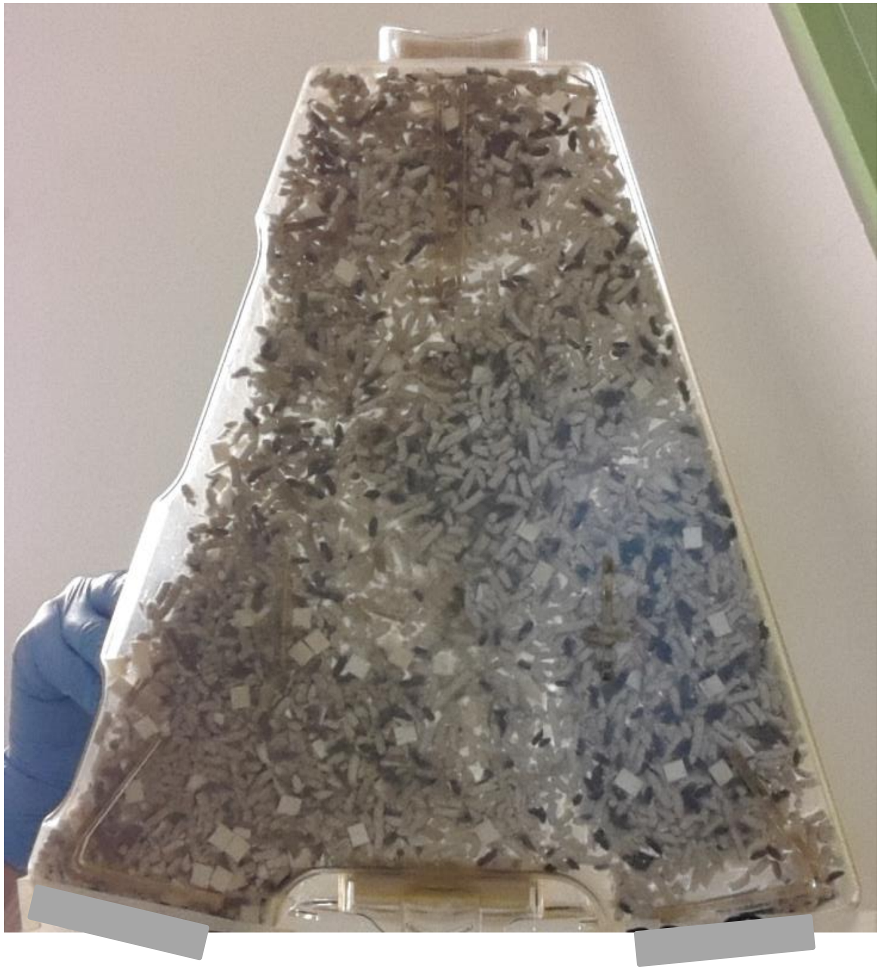

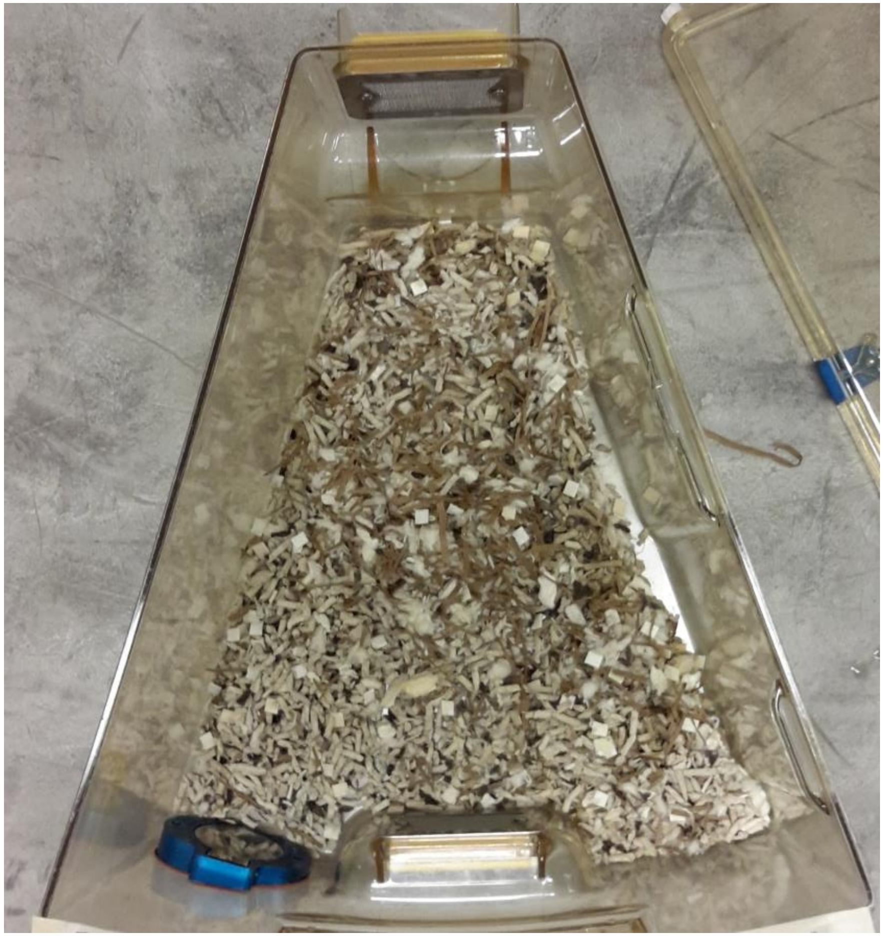

July 7 STD 1

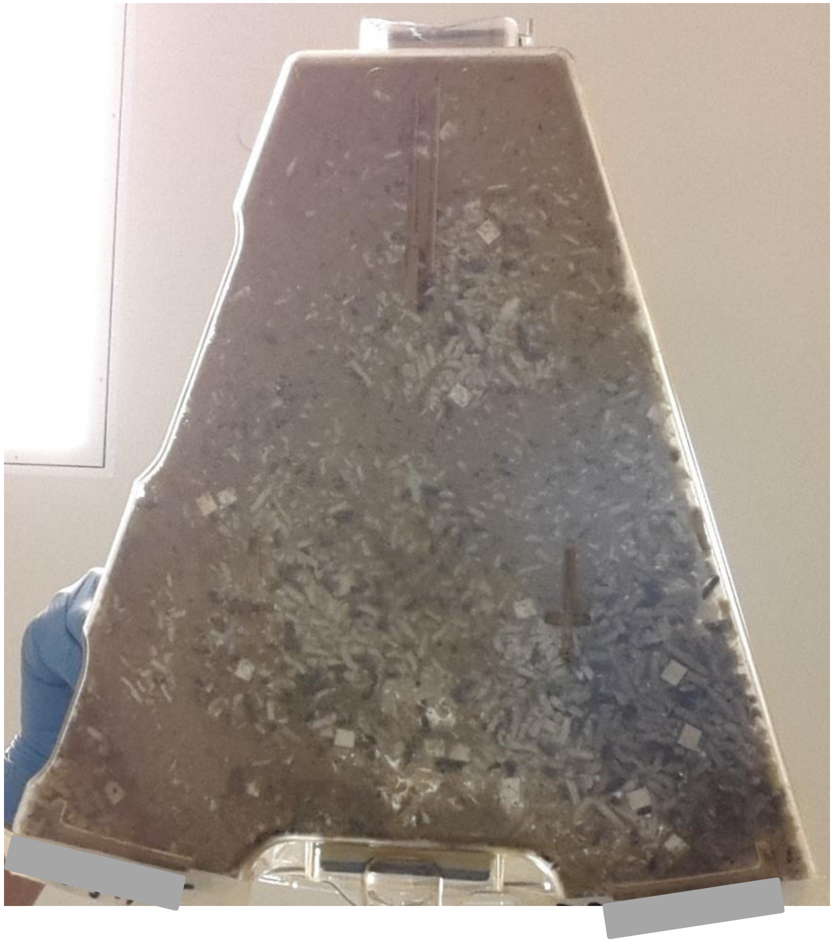

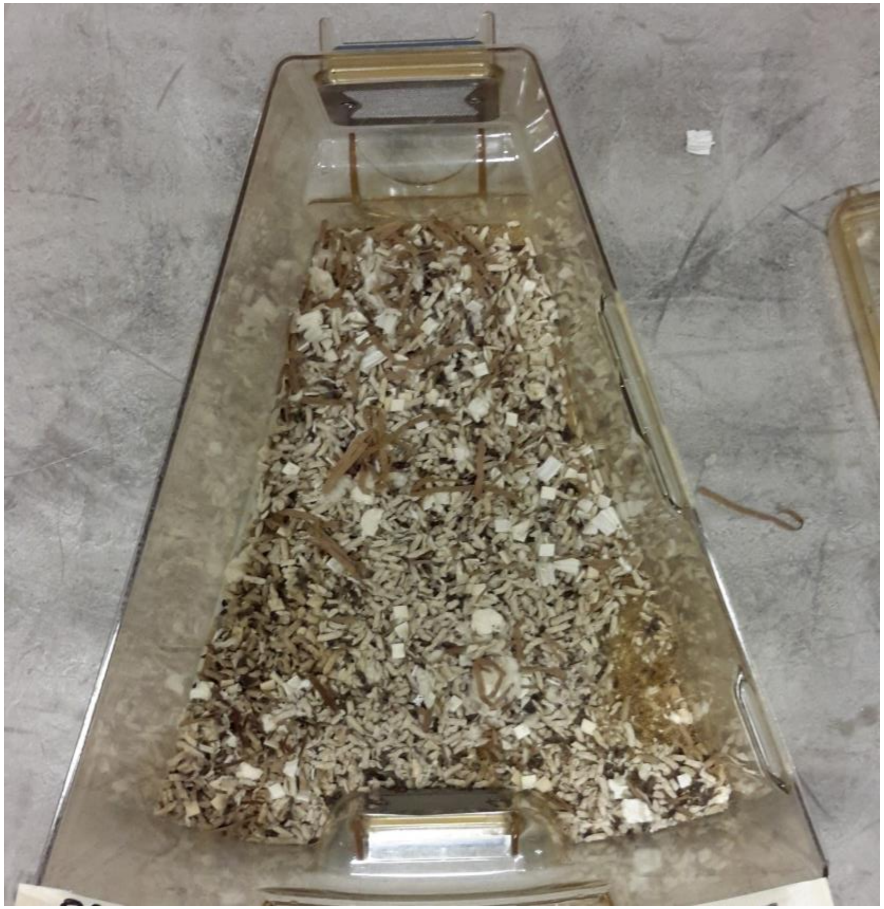

July 7 COMP 2 mid

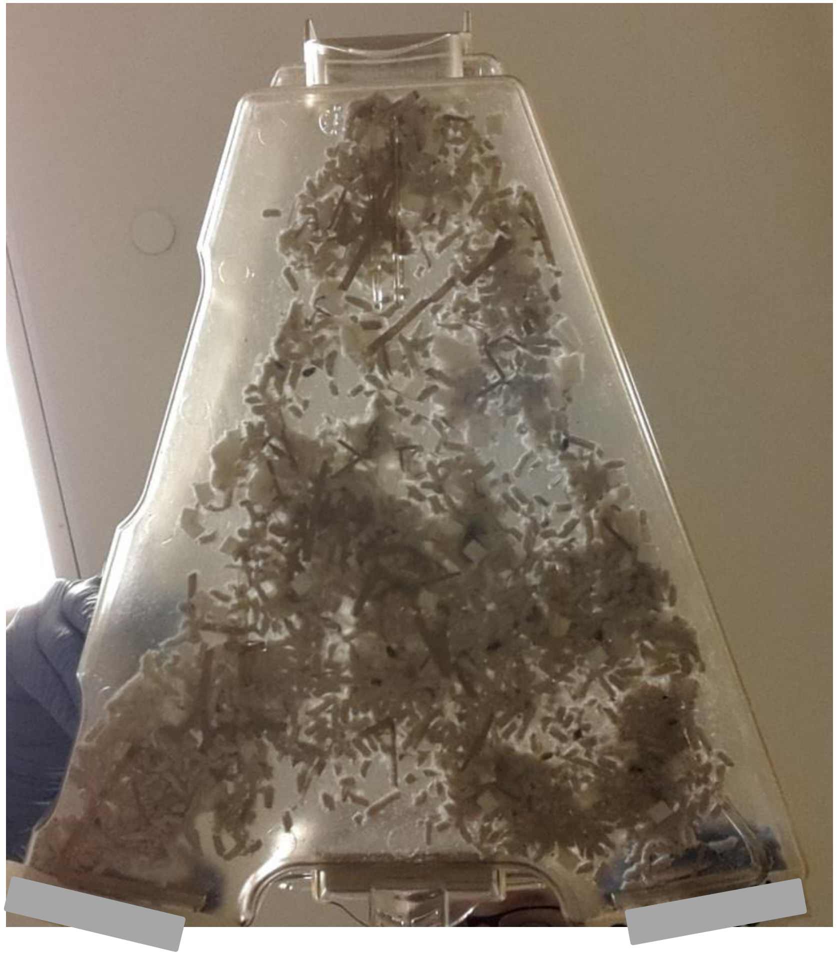

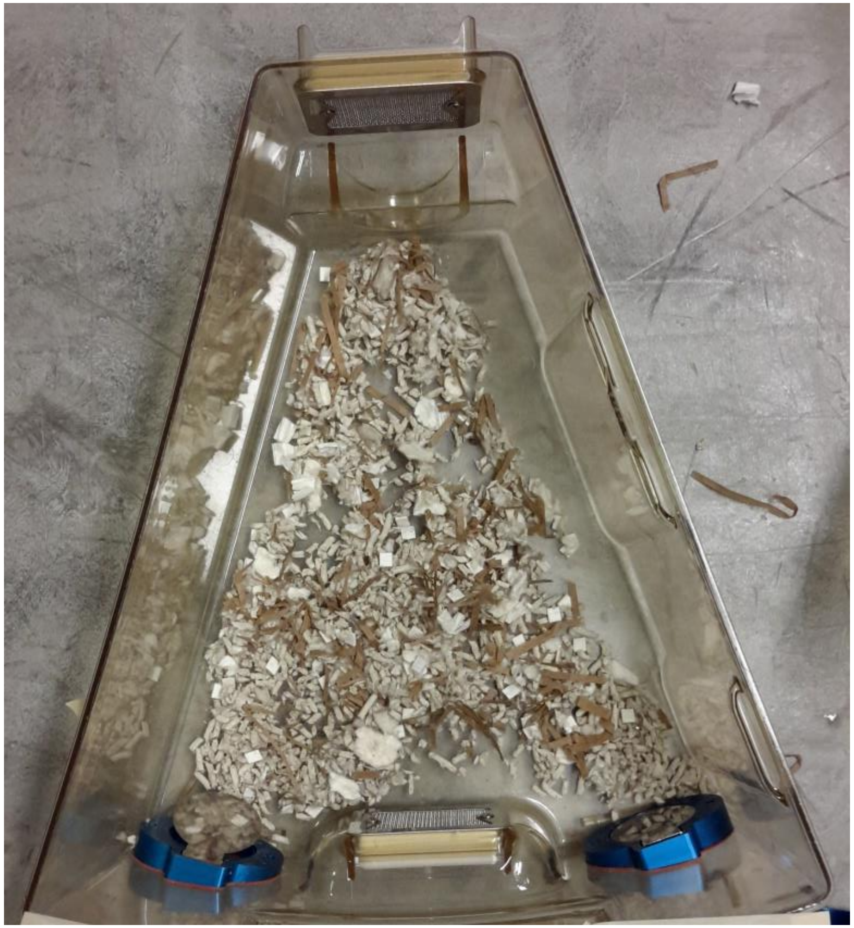

July 7 COMP 2 left

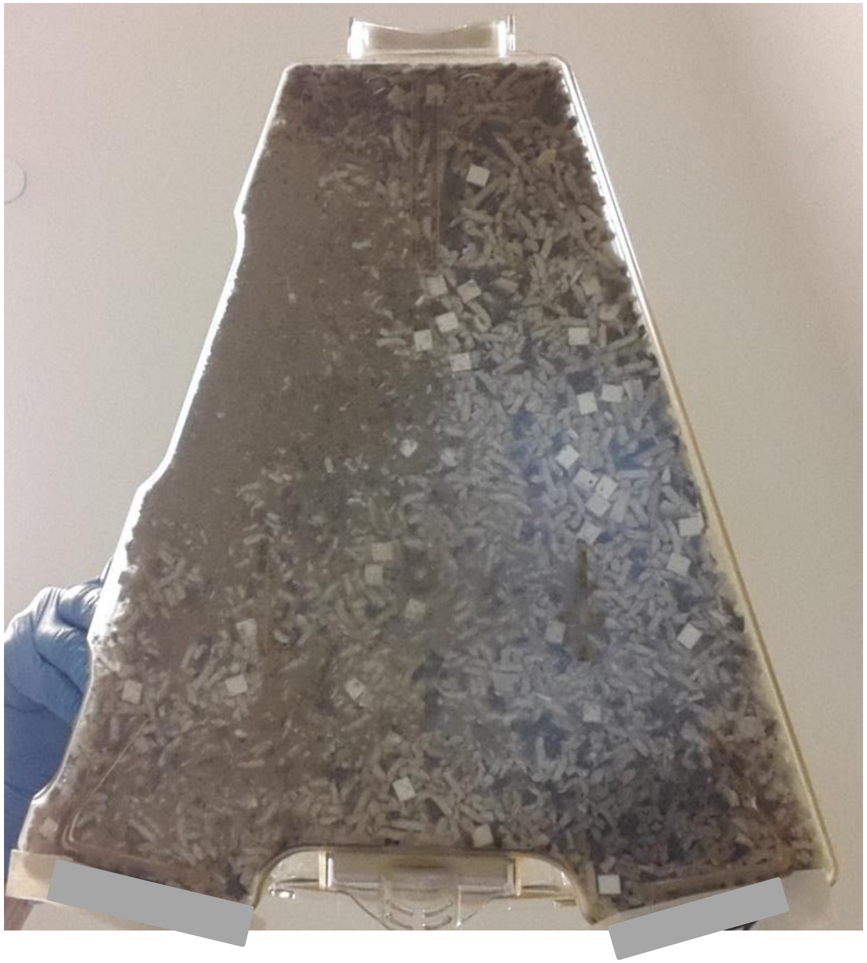

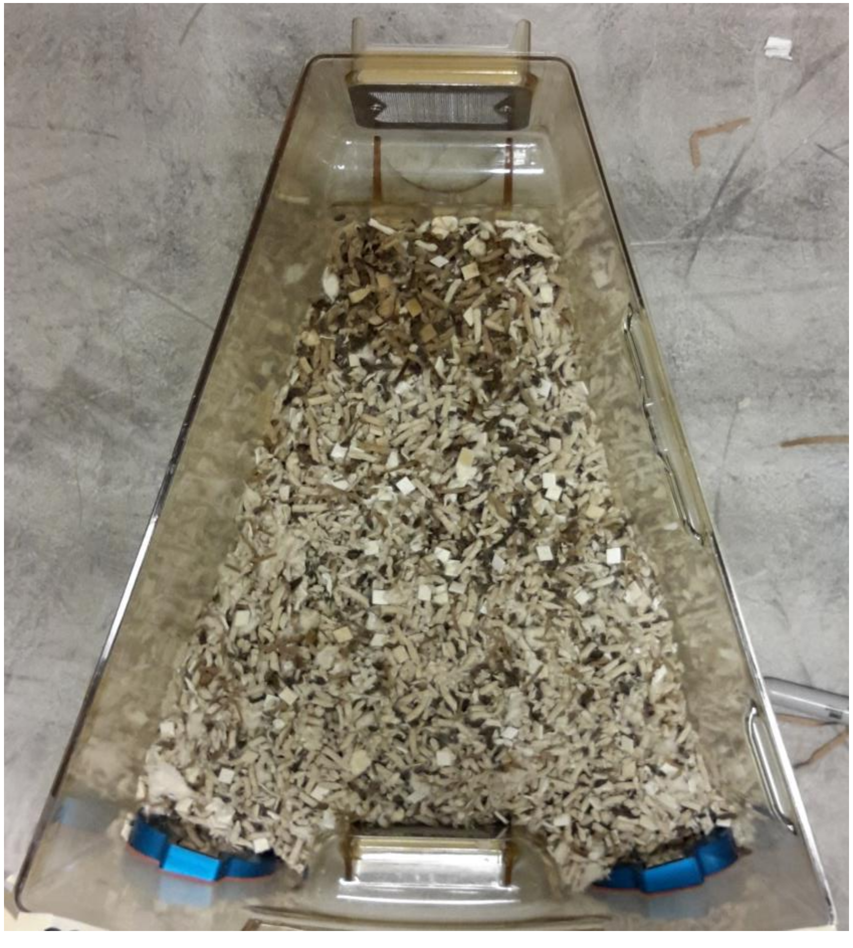

July 7 COMP 2 right

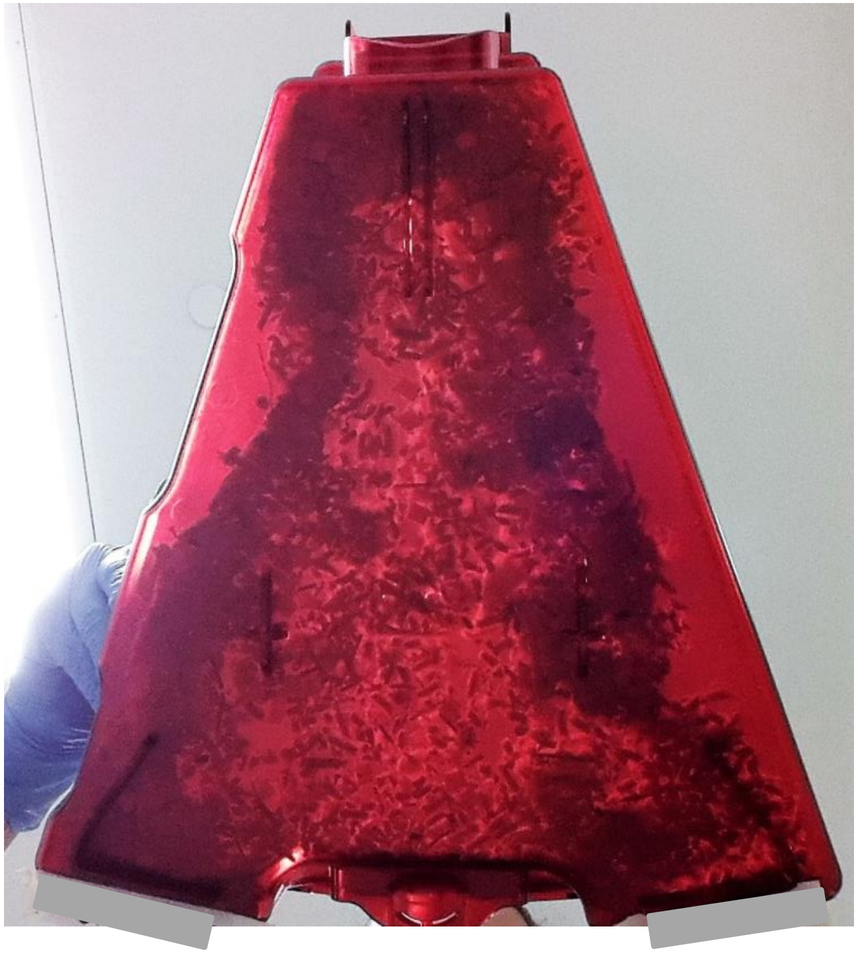

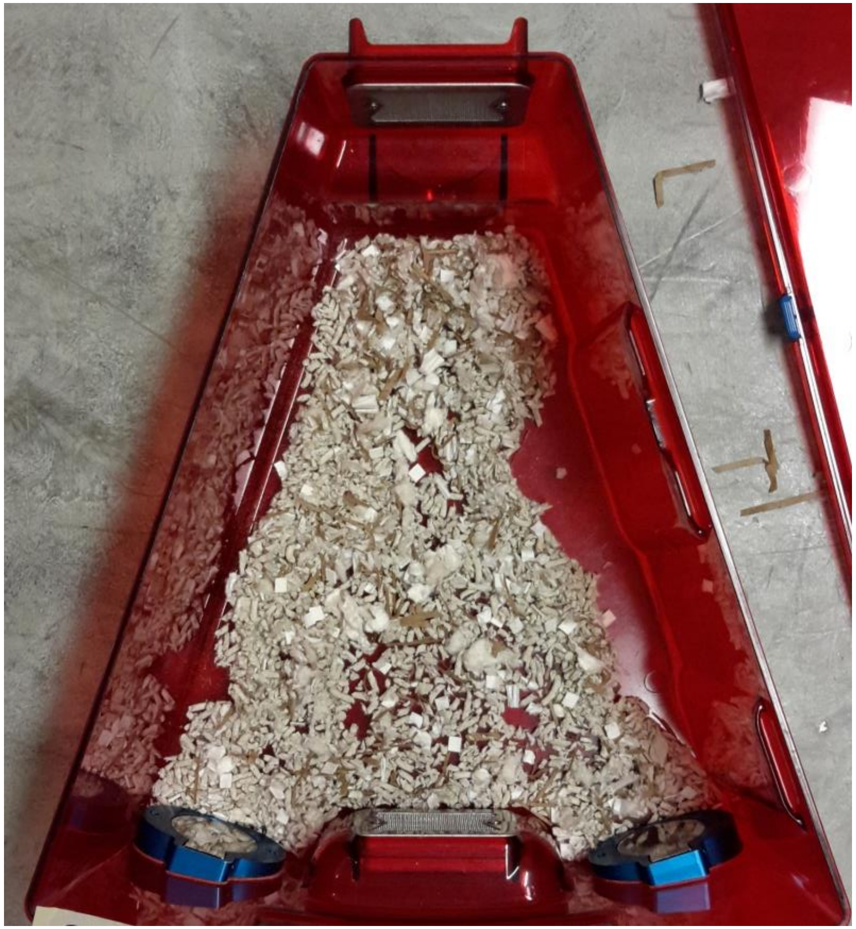

July 7 STD 2

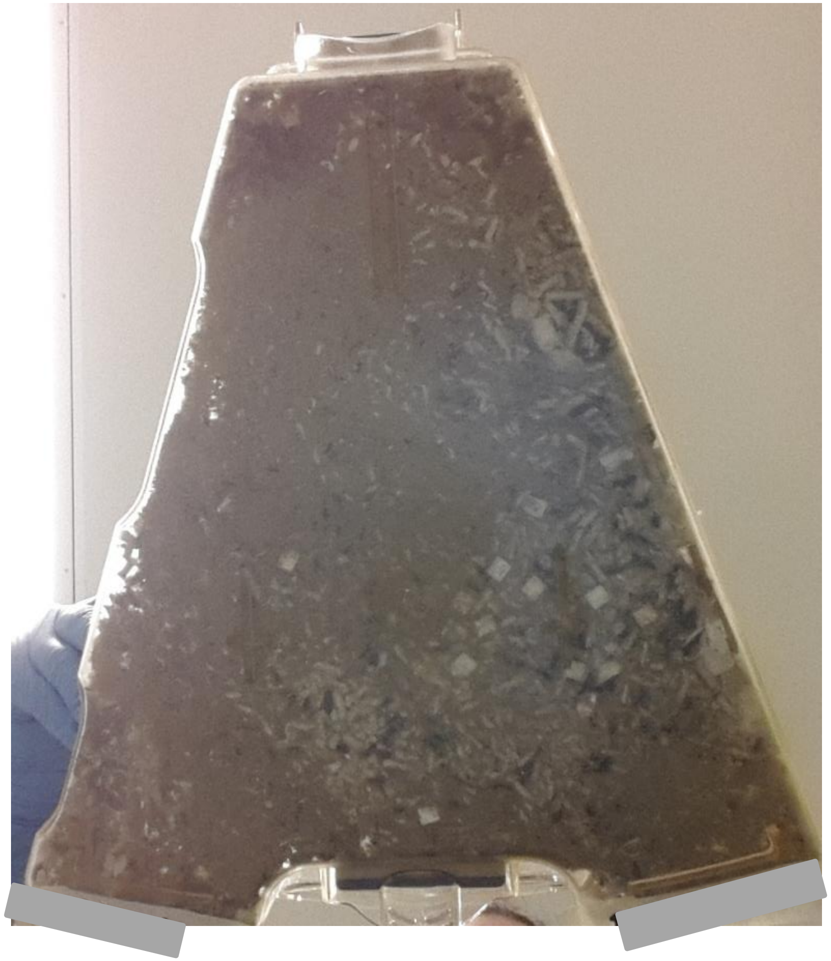

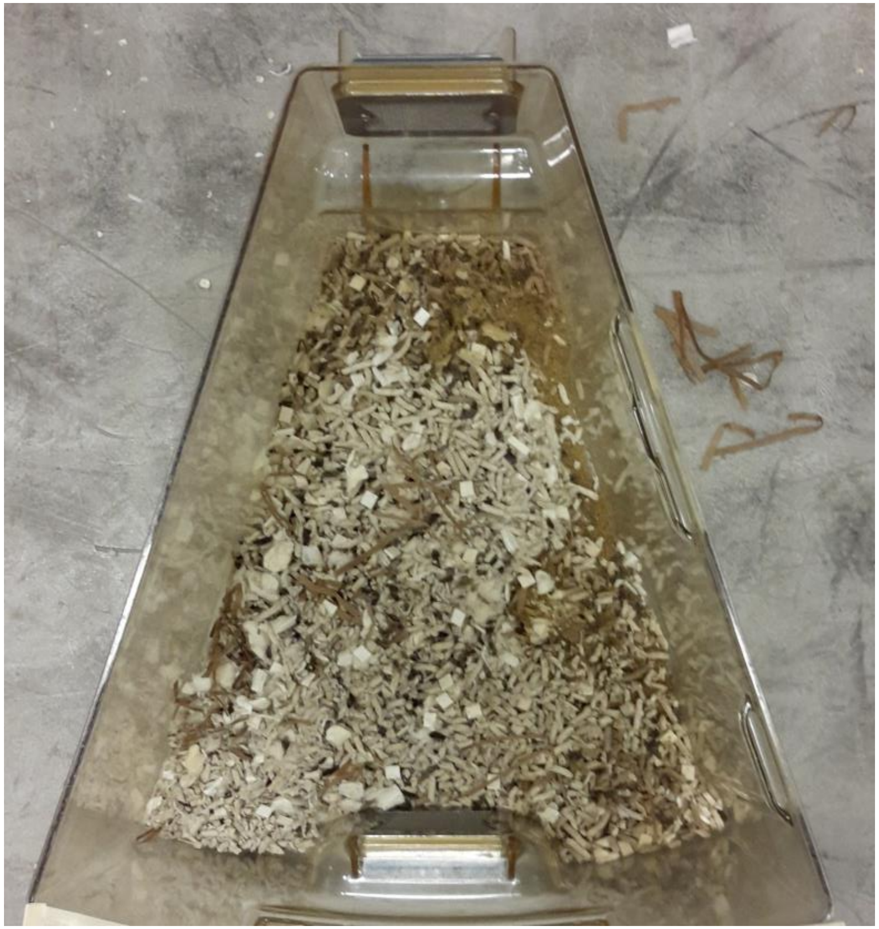

July 8 COMP 3 right

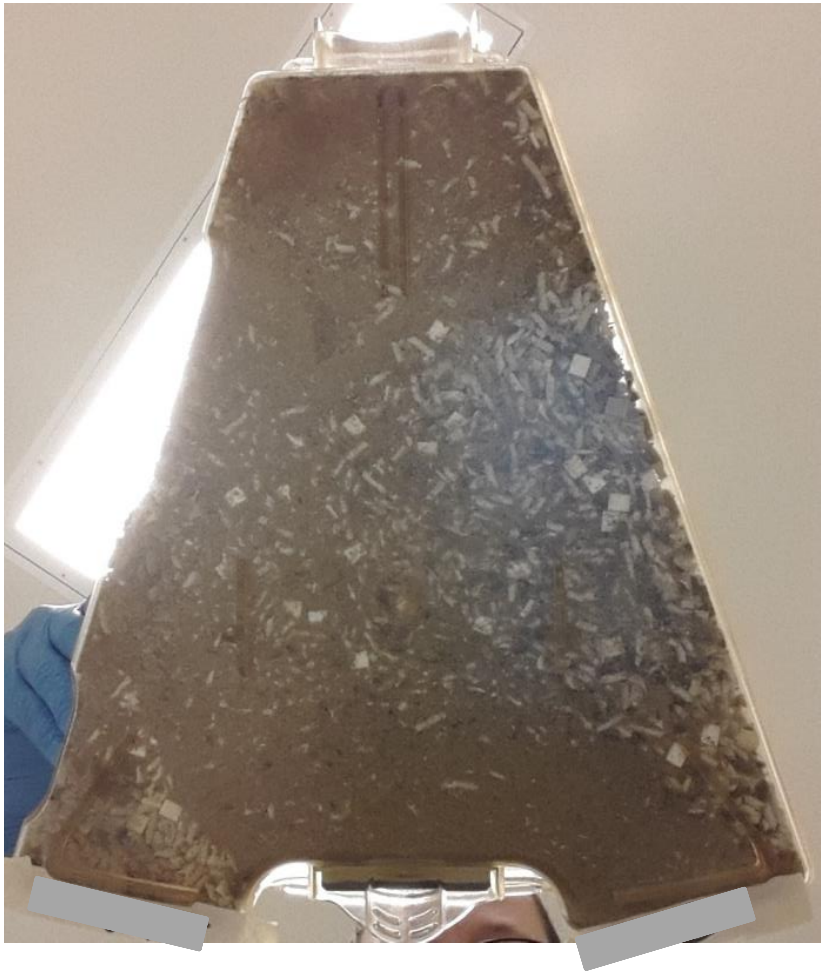

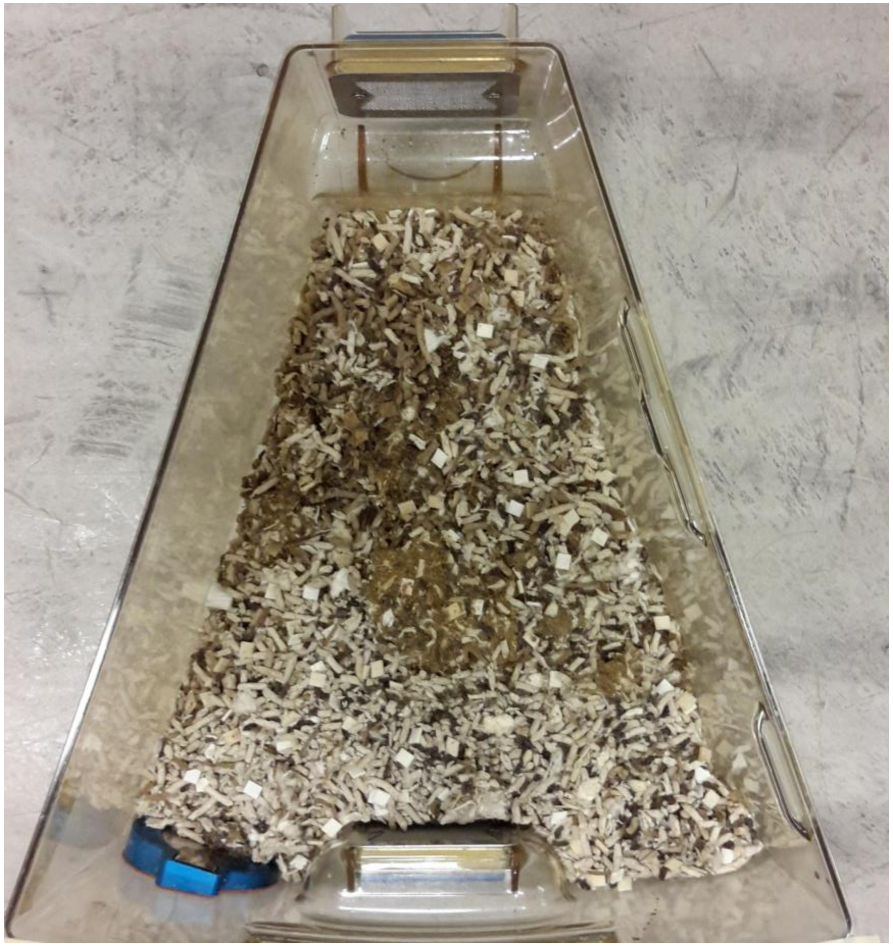

July 8 COMP 3 left

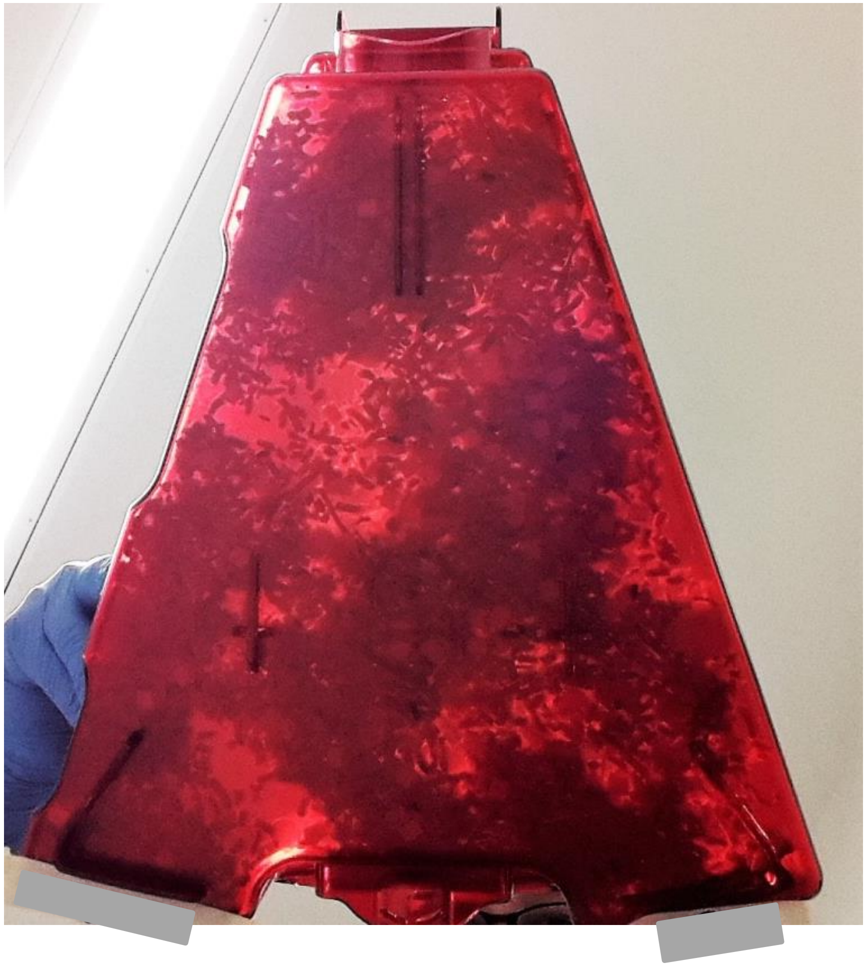

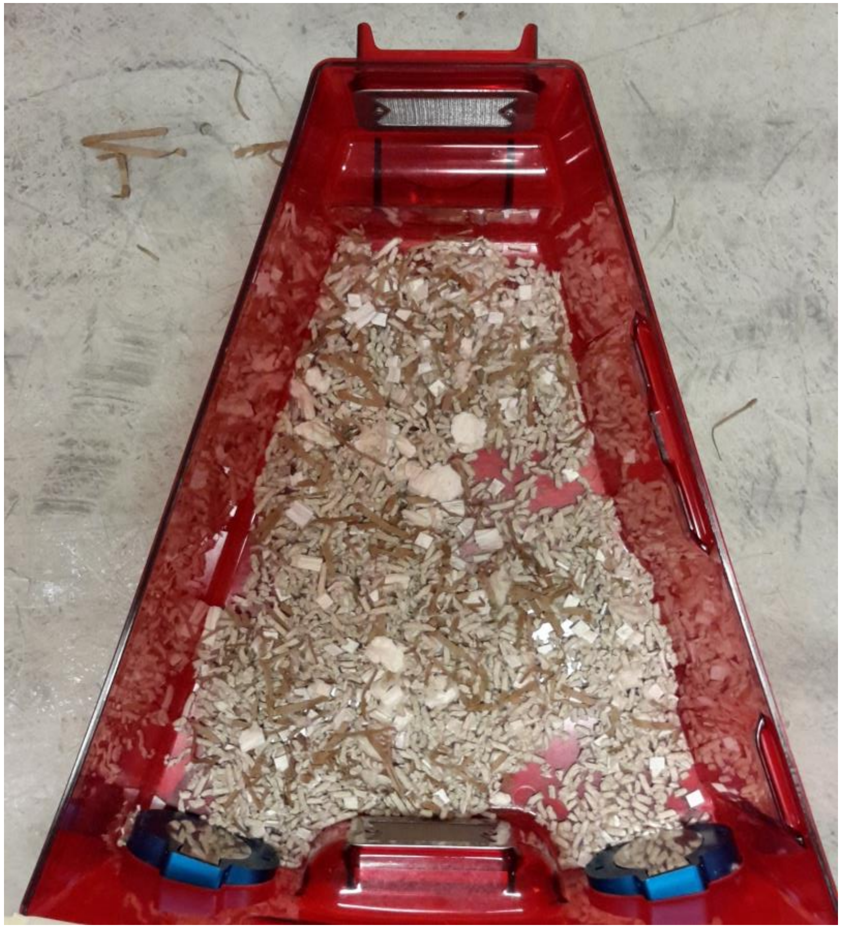

July 8 COMP 3 mid

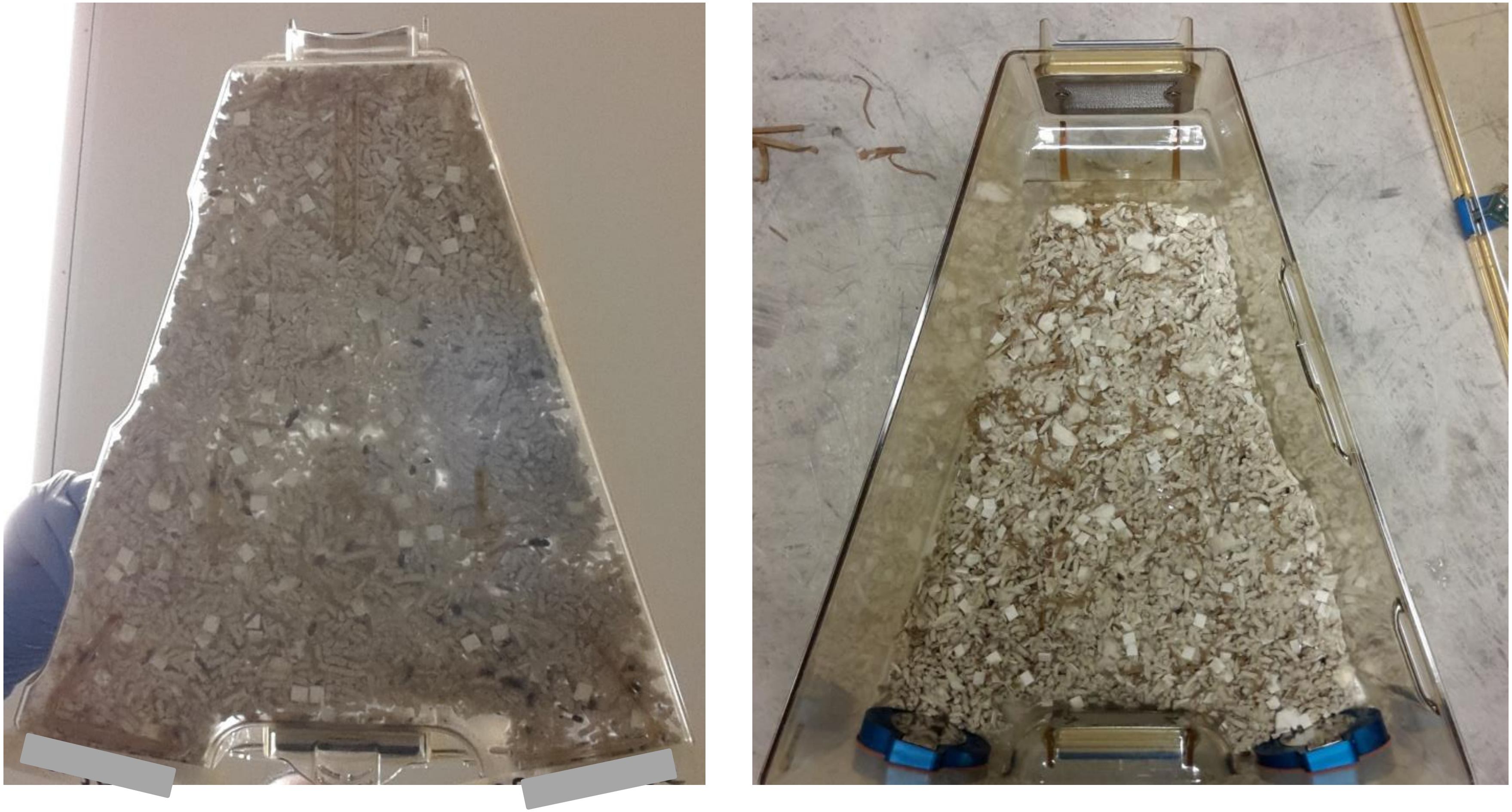

July 8 STD 3

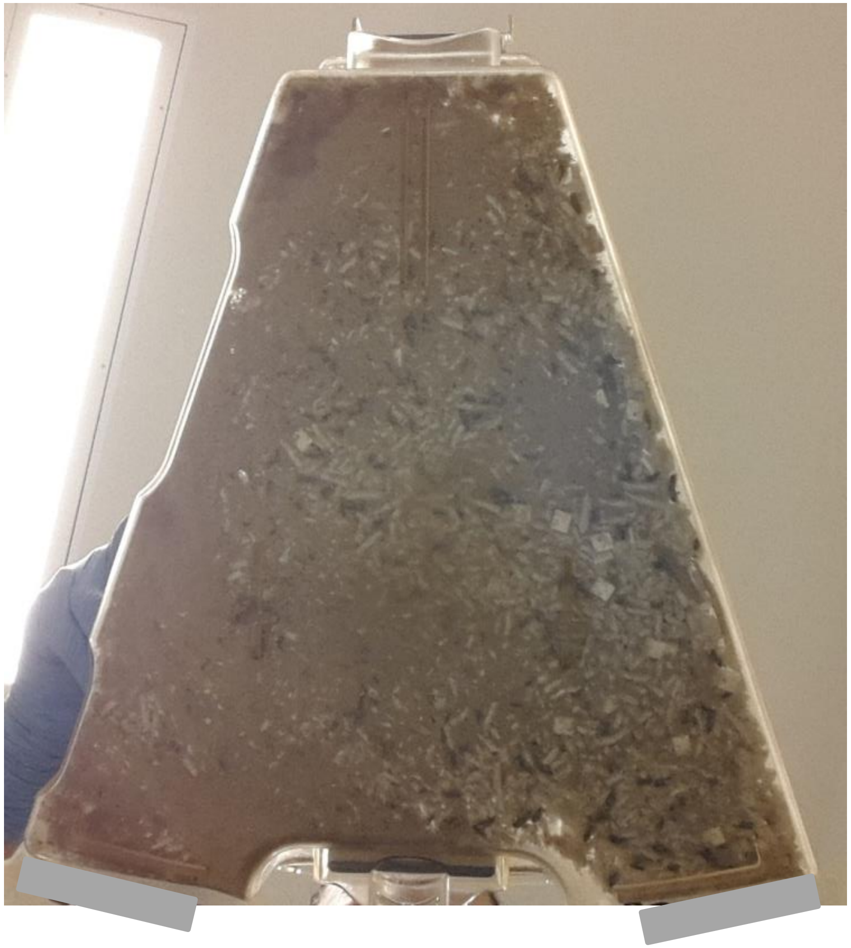

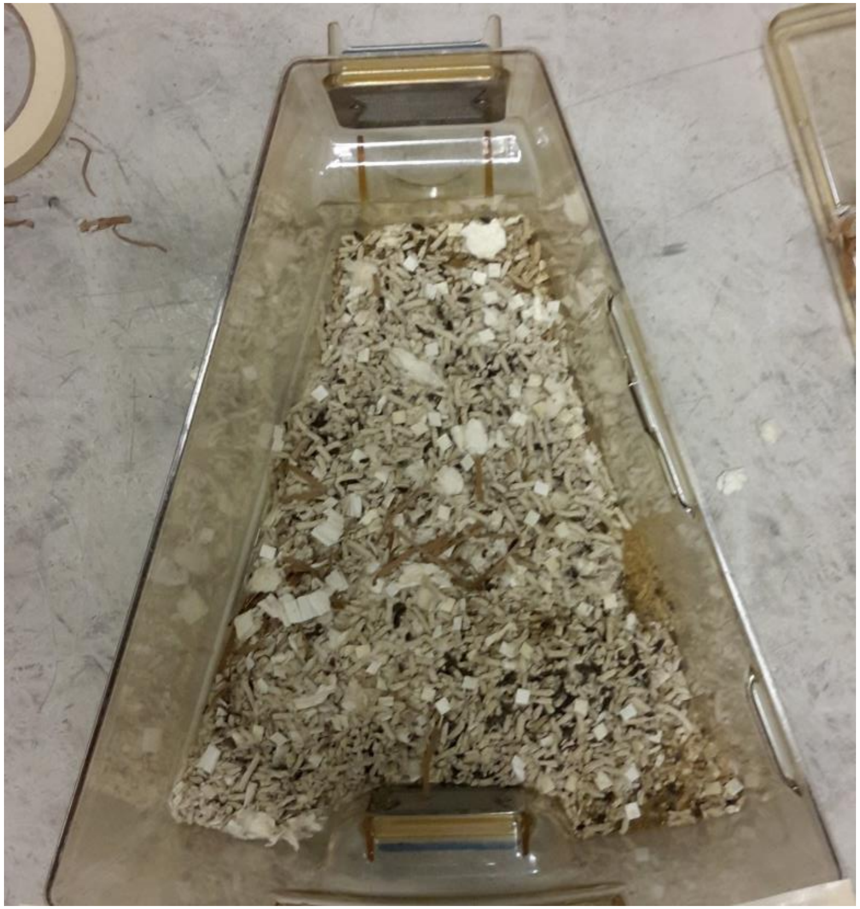

July 8 COMP 4 left

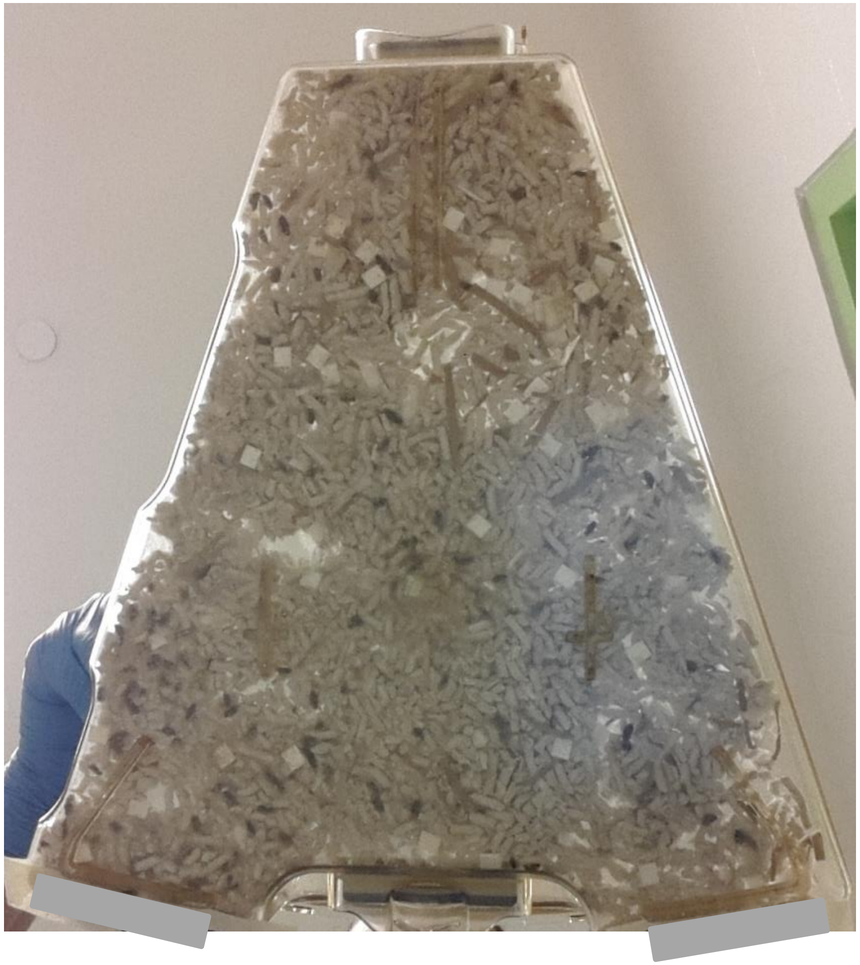

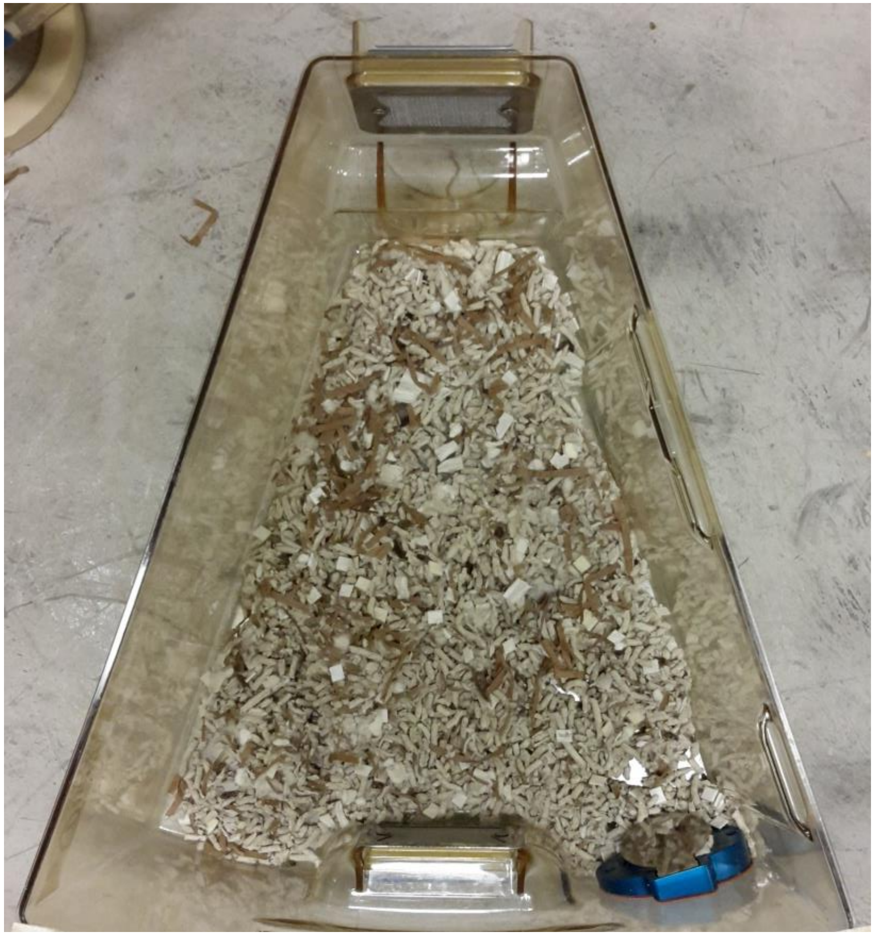

July 8 COMP 4 mid

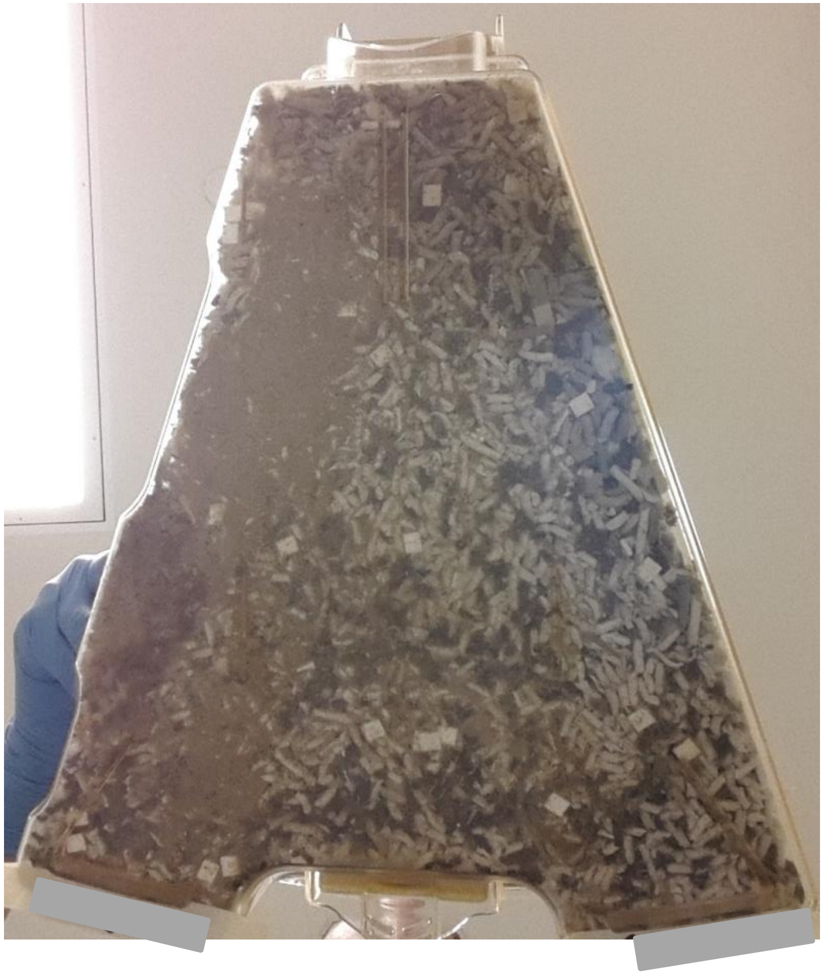

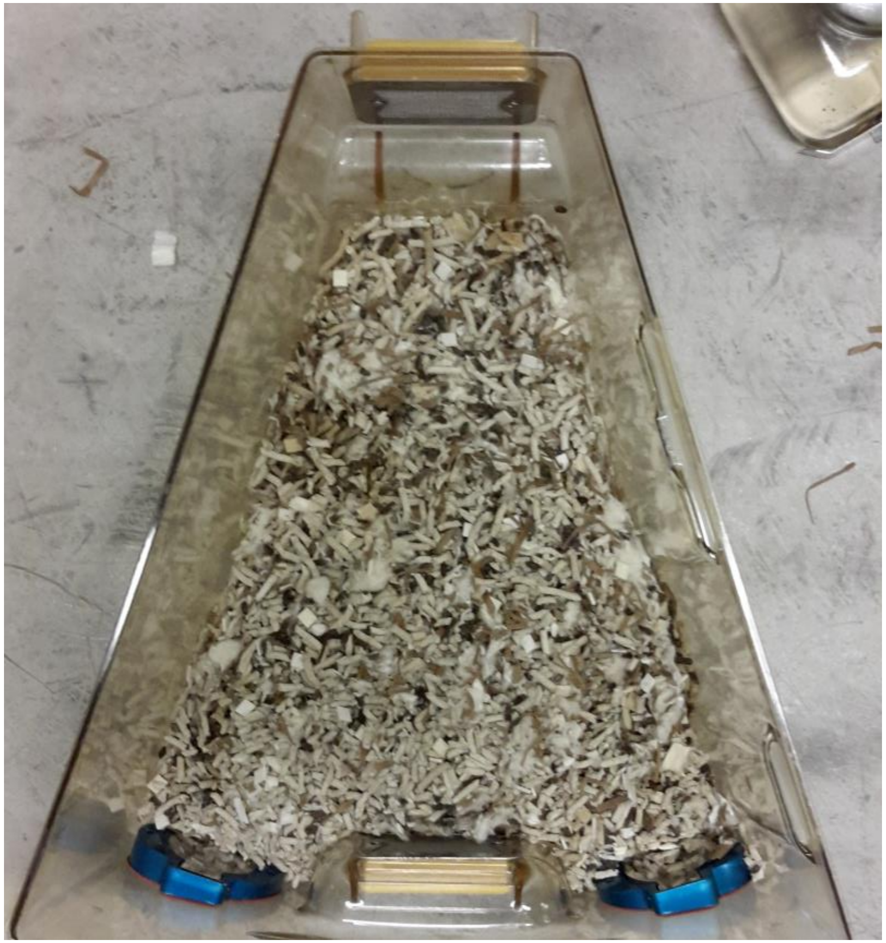

July 8 COMP 4 right

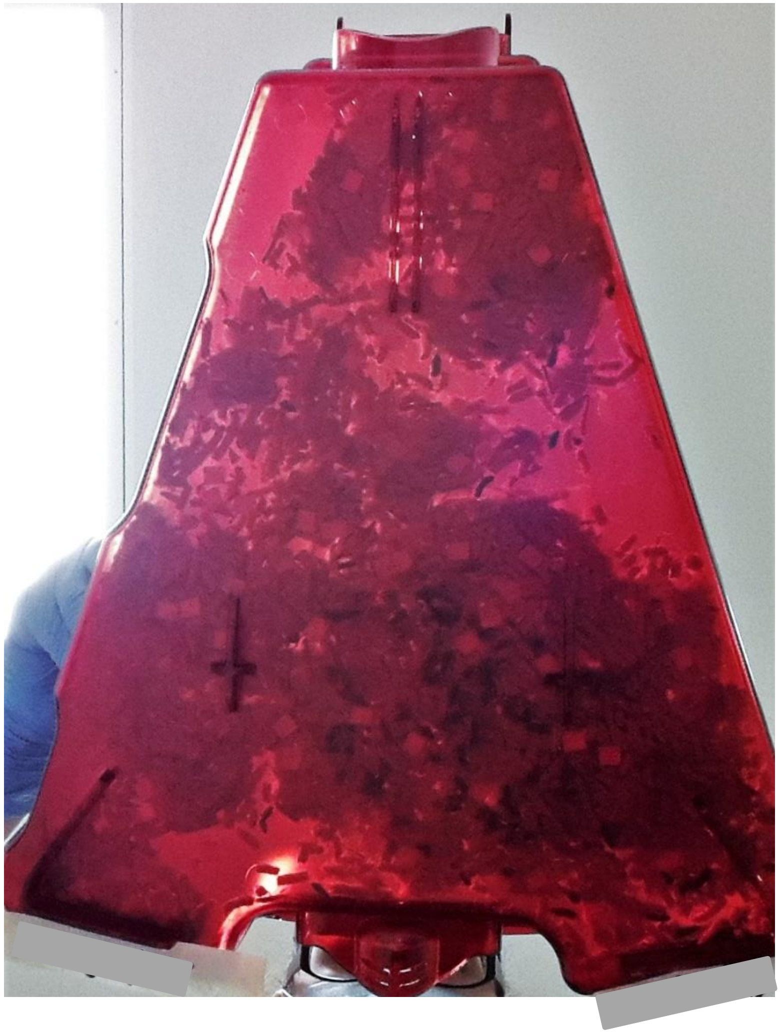

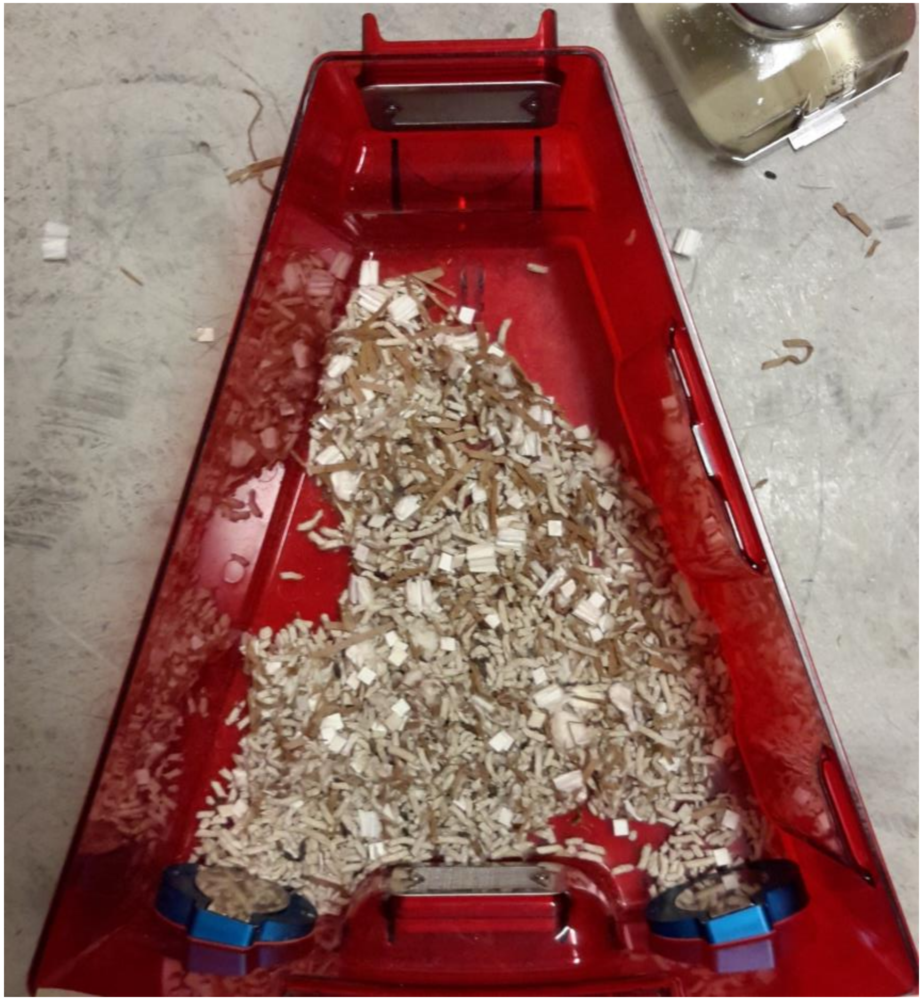

July 8 STD 4

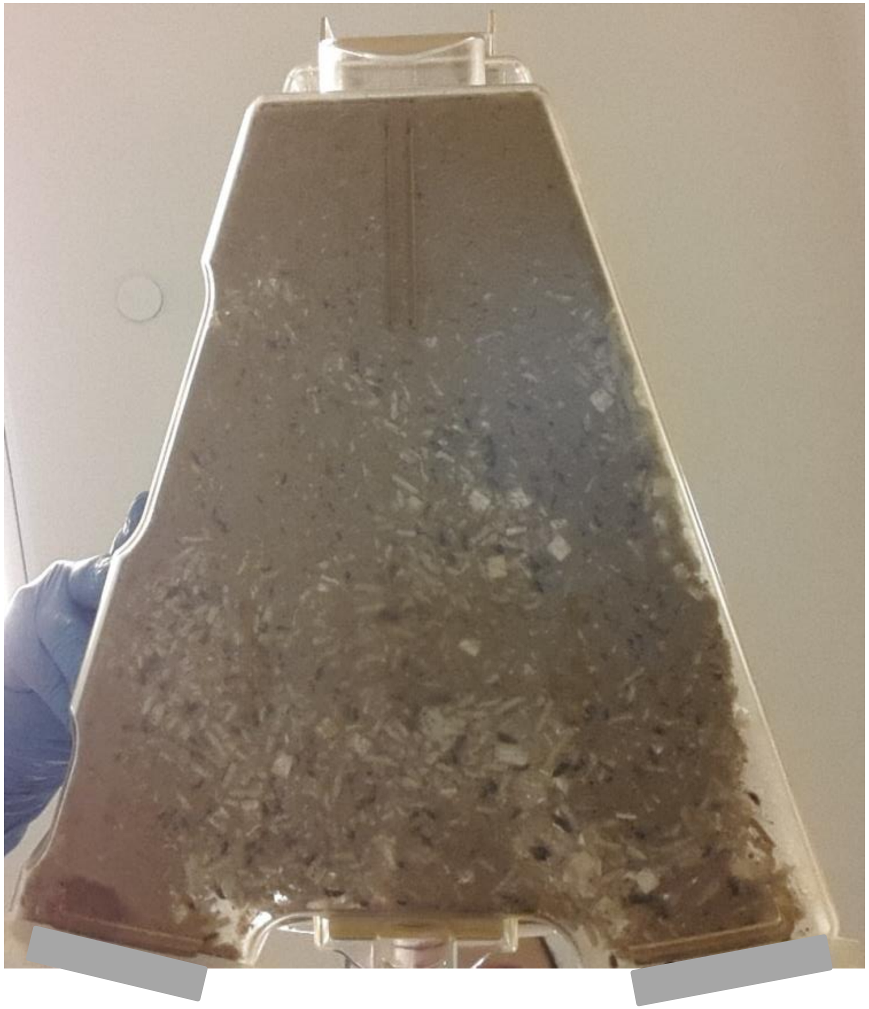

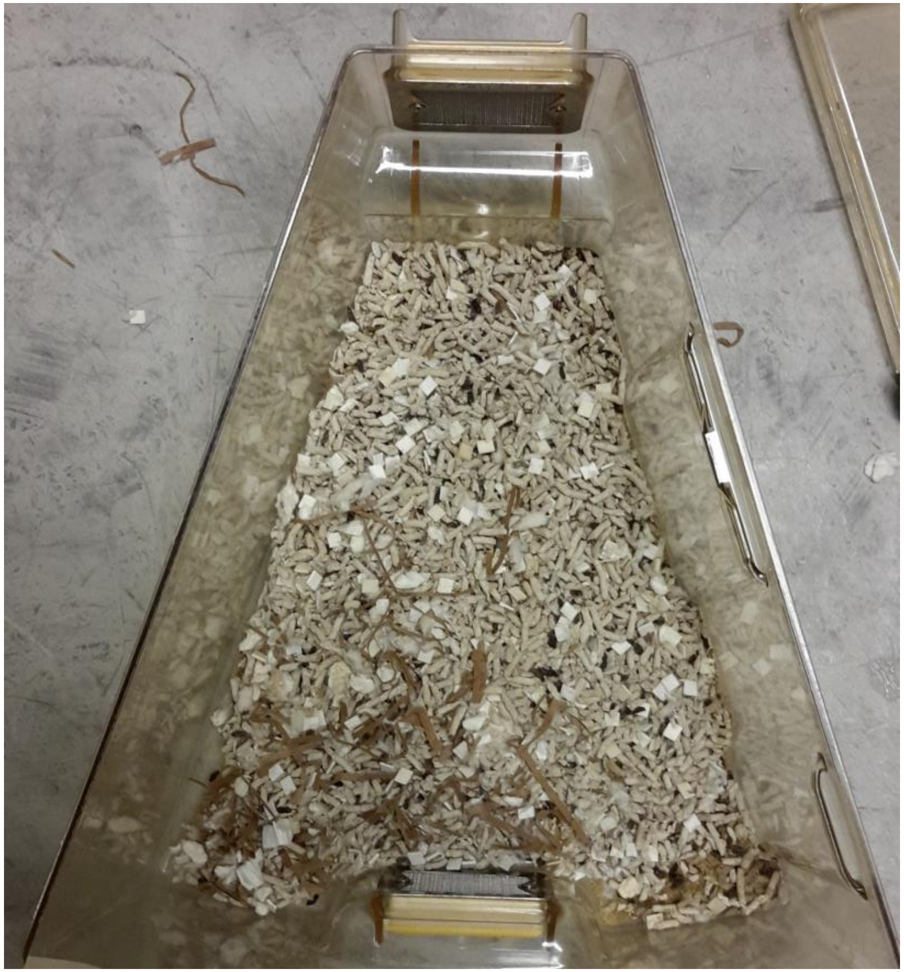

July 9 COMP 5 mid

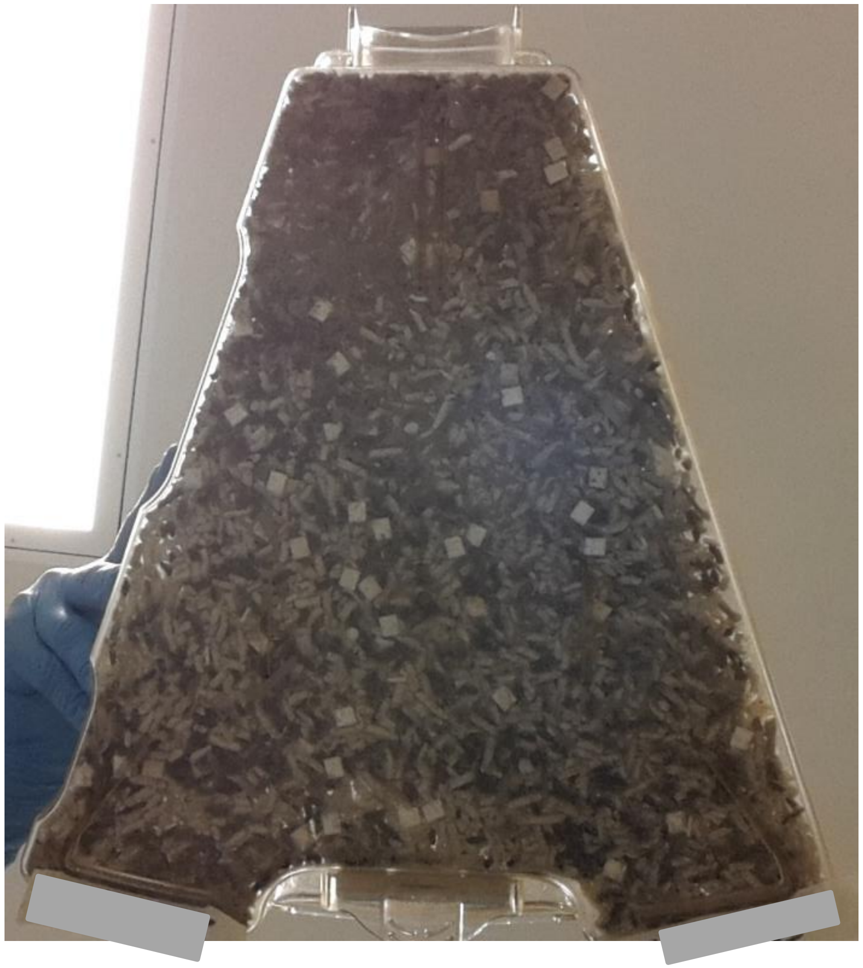 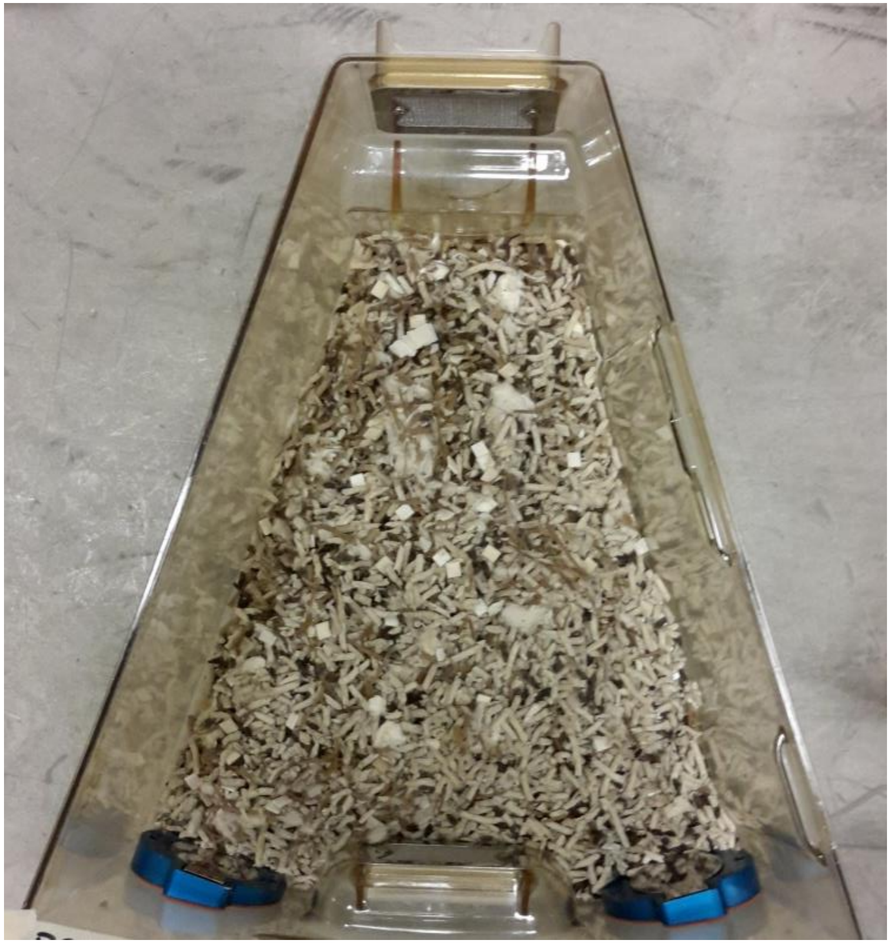

July 9 COMP 5 left

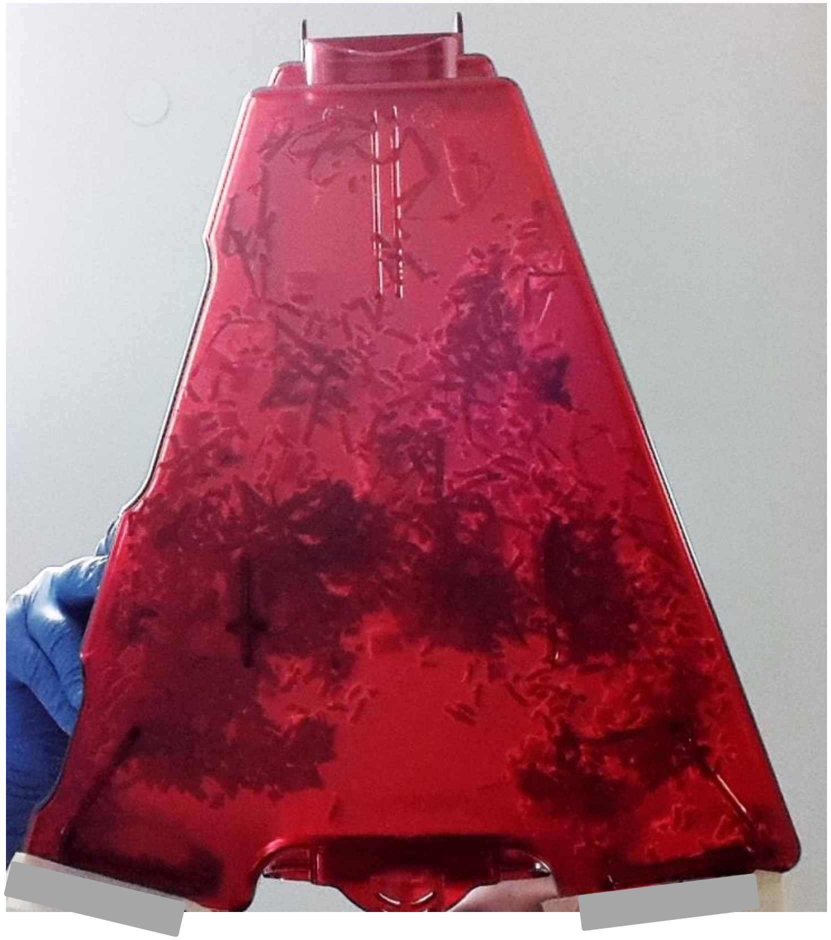

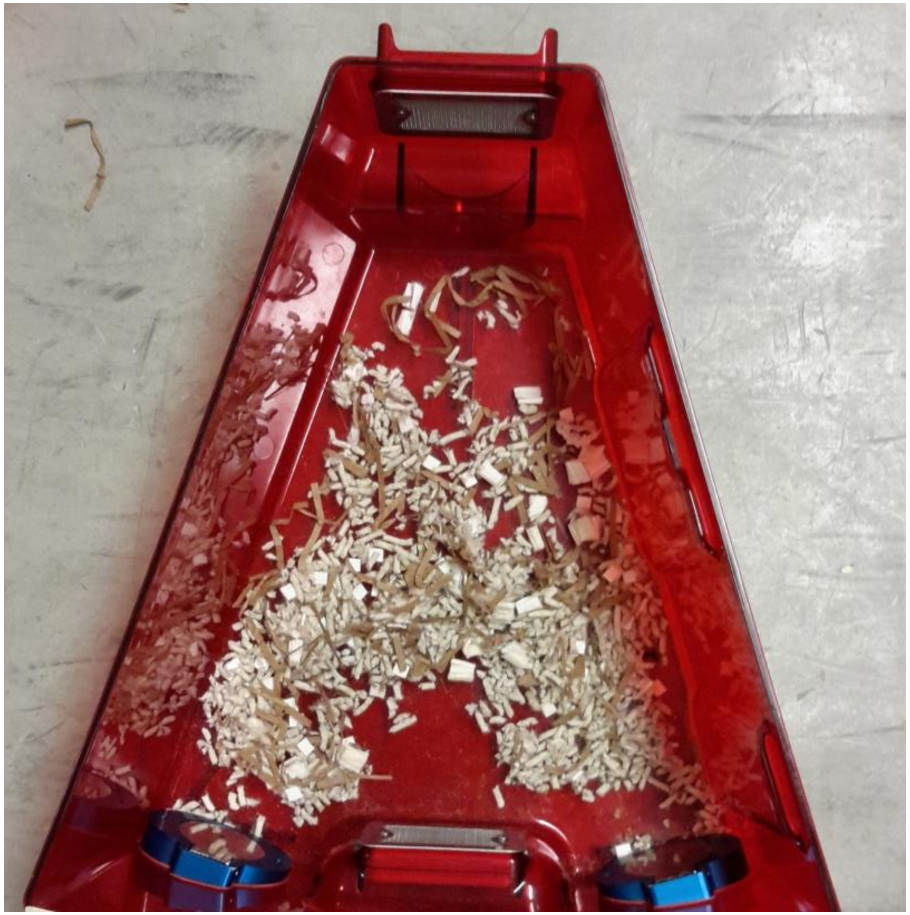

July 9 COMP 5 right

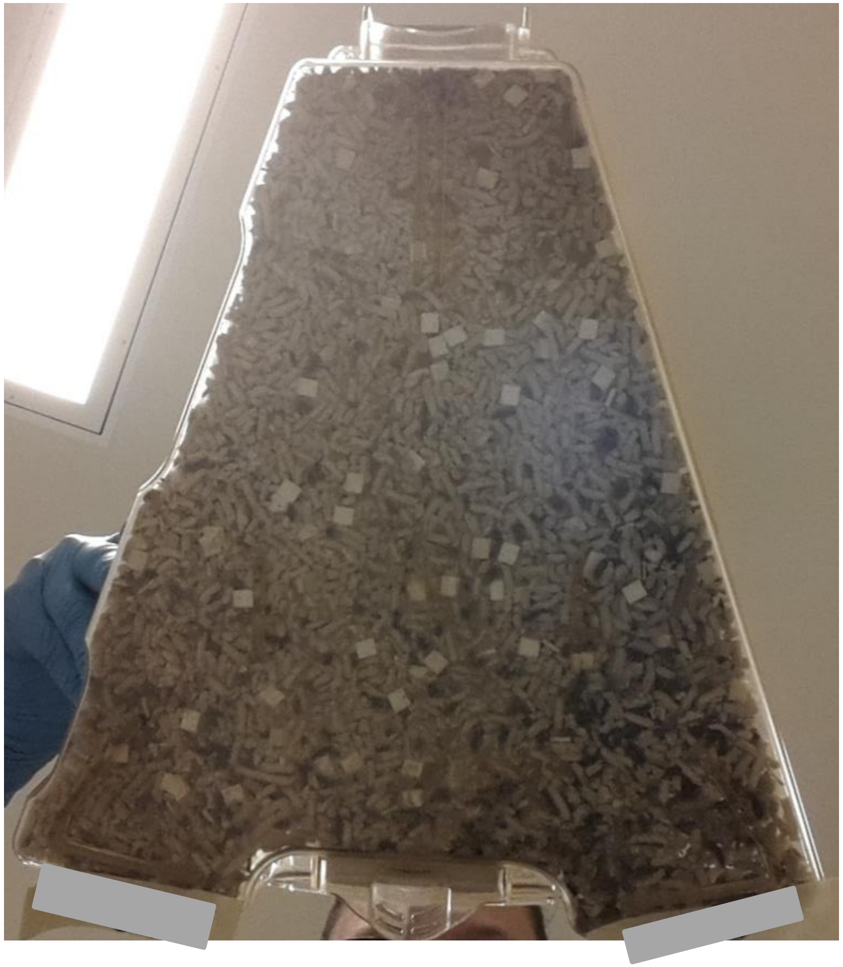

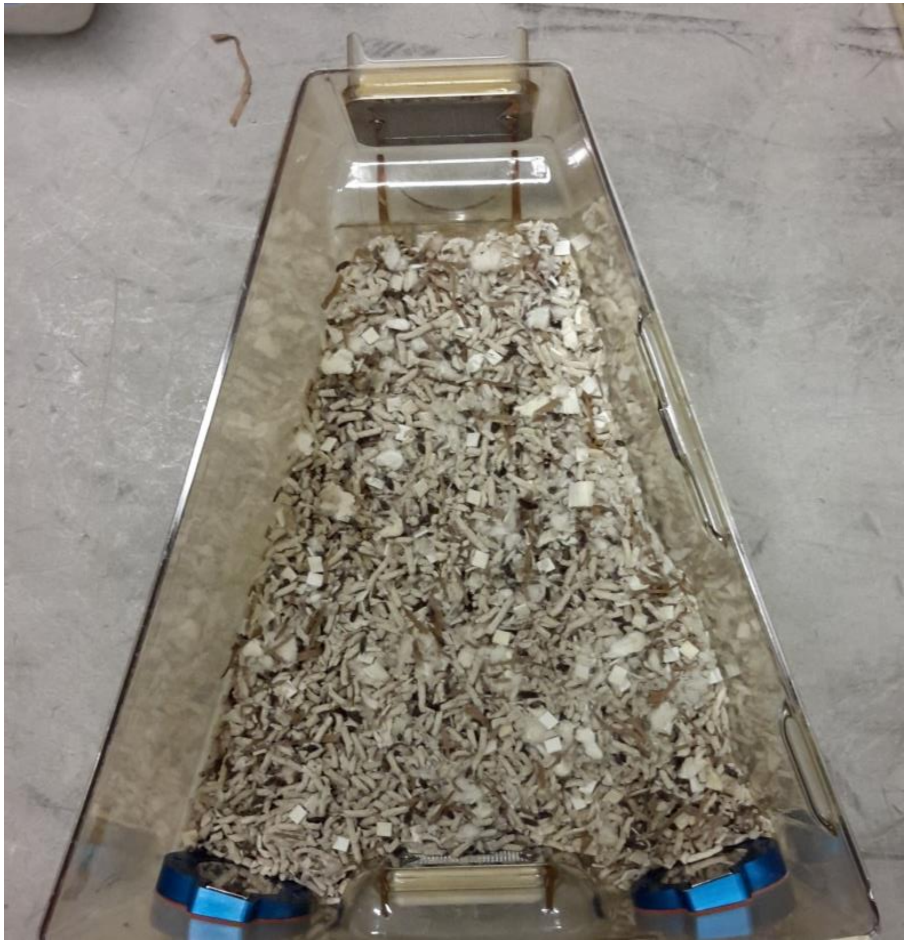

July 9 STD 5

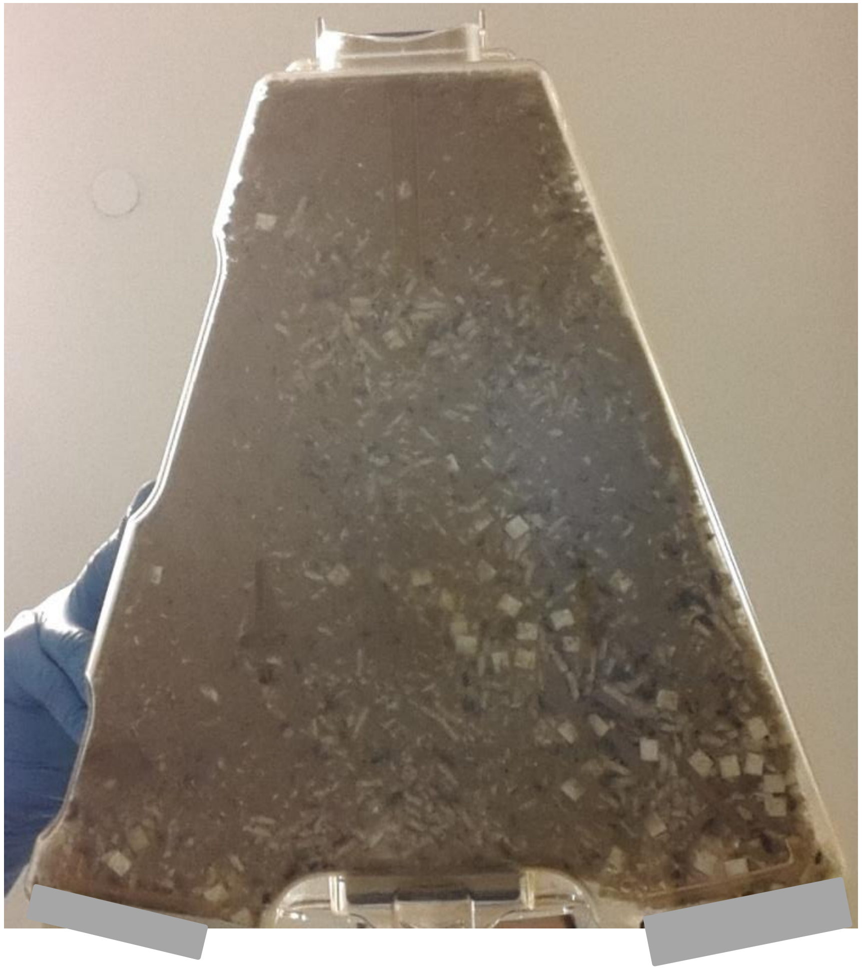

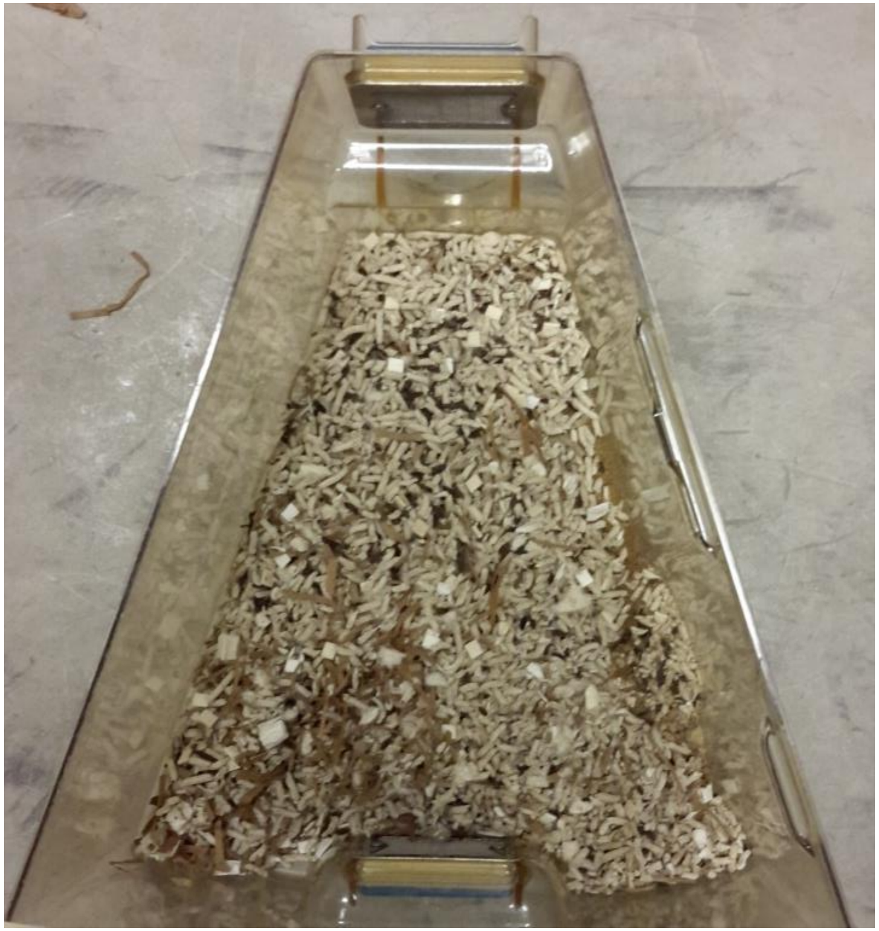

July 14 COMP 1 left

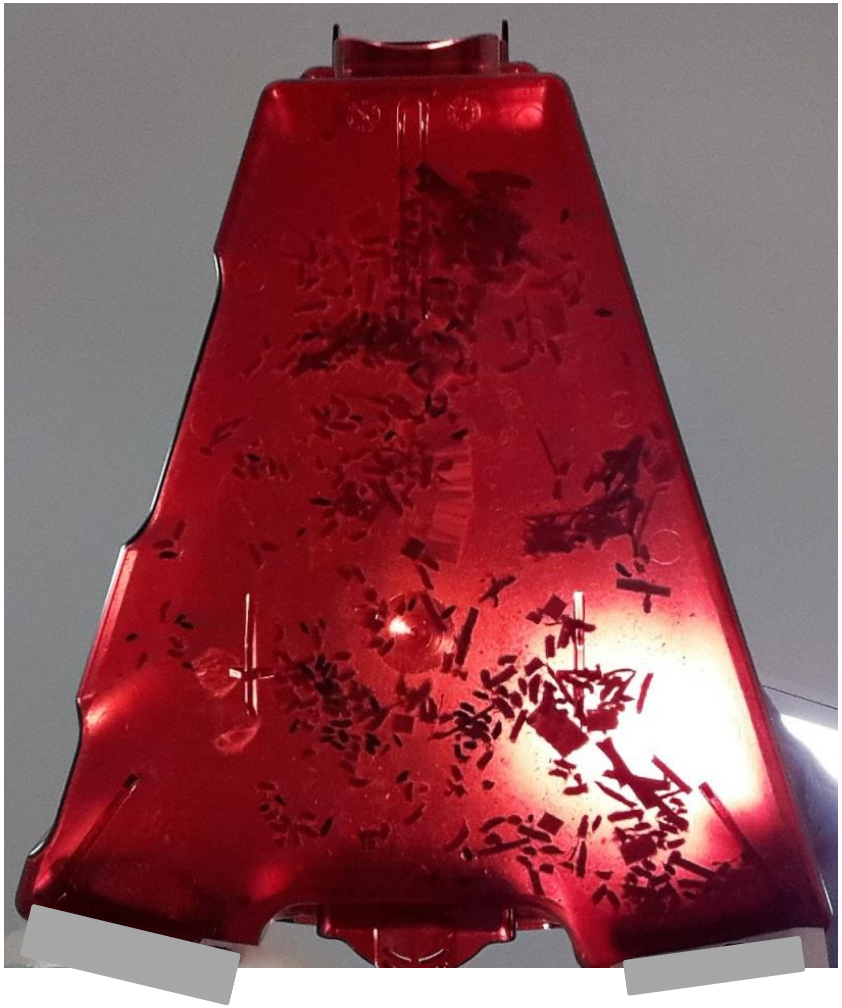

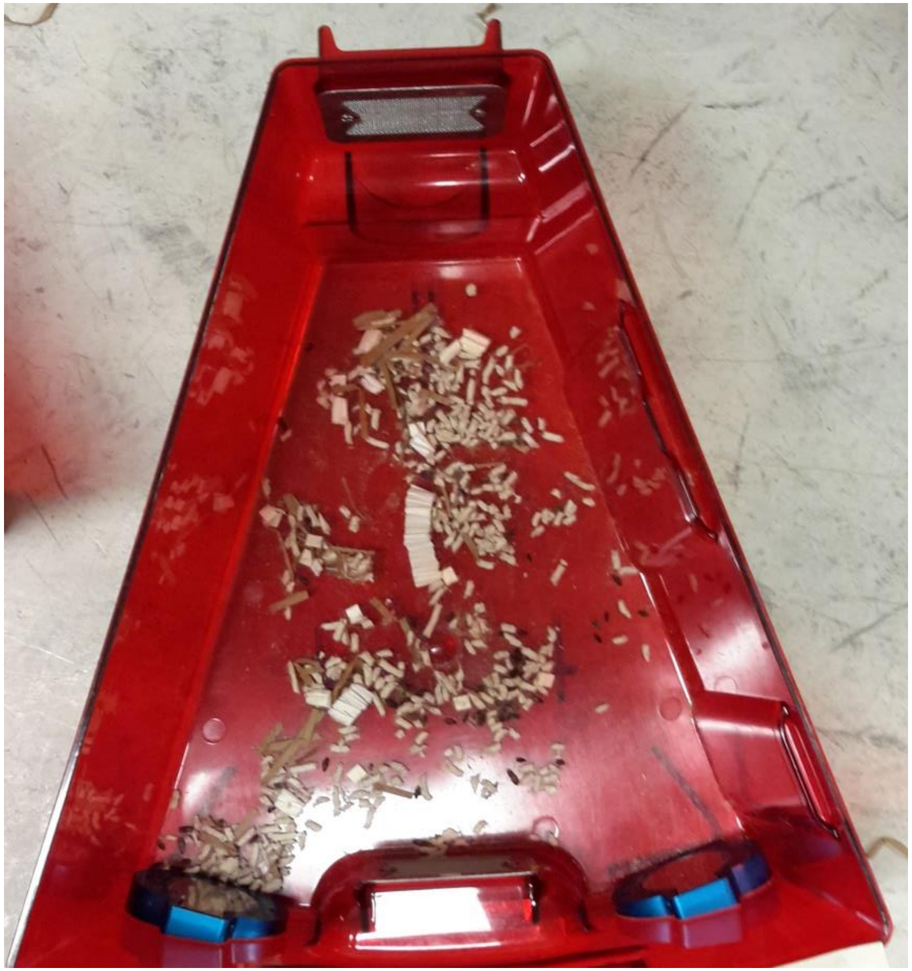

July 14 COMP 1 mid

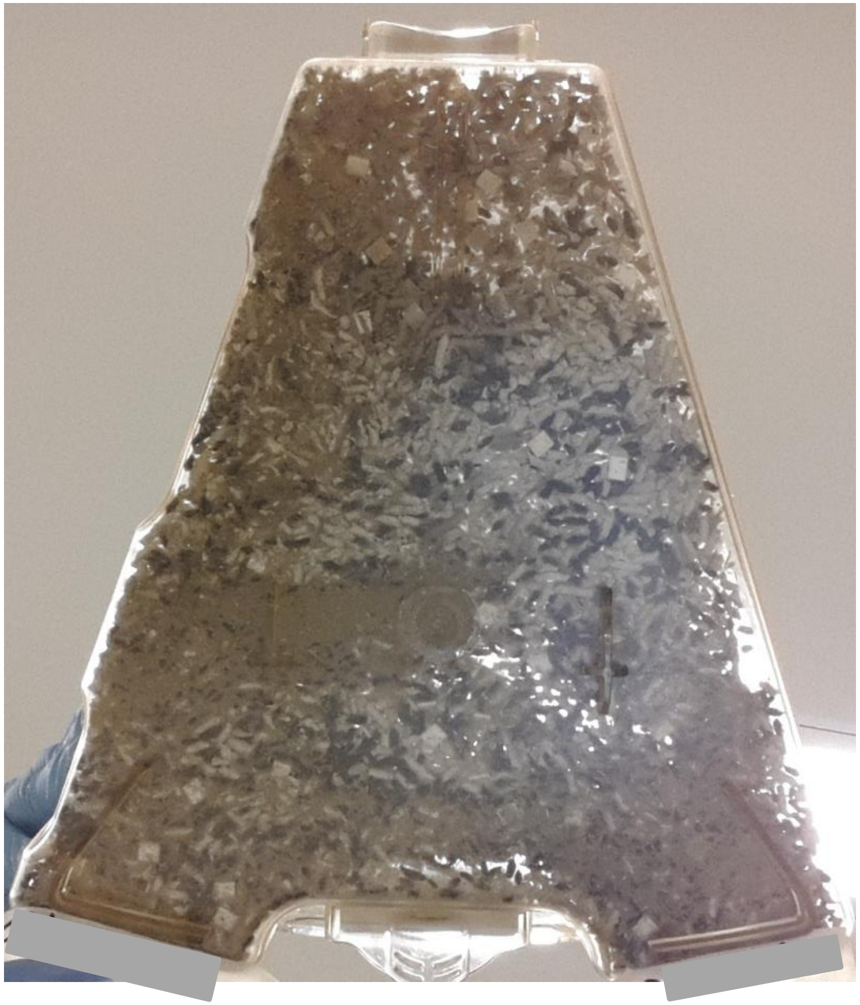

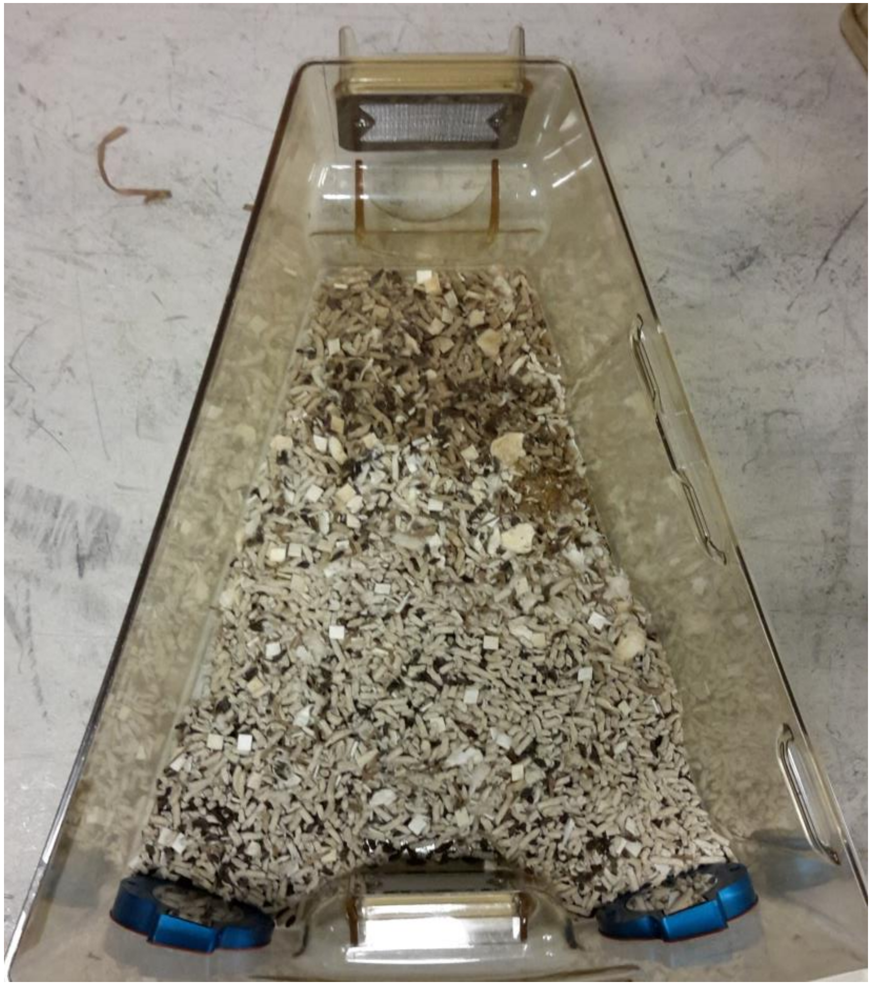

July 14 COMP 1 right

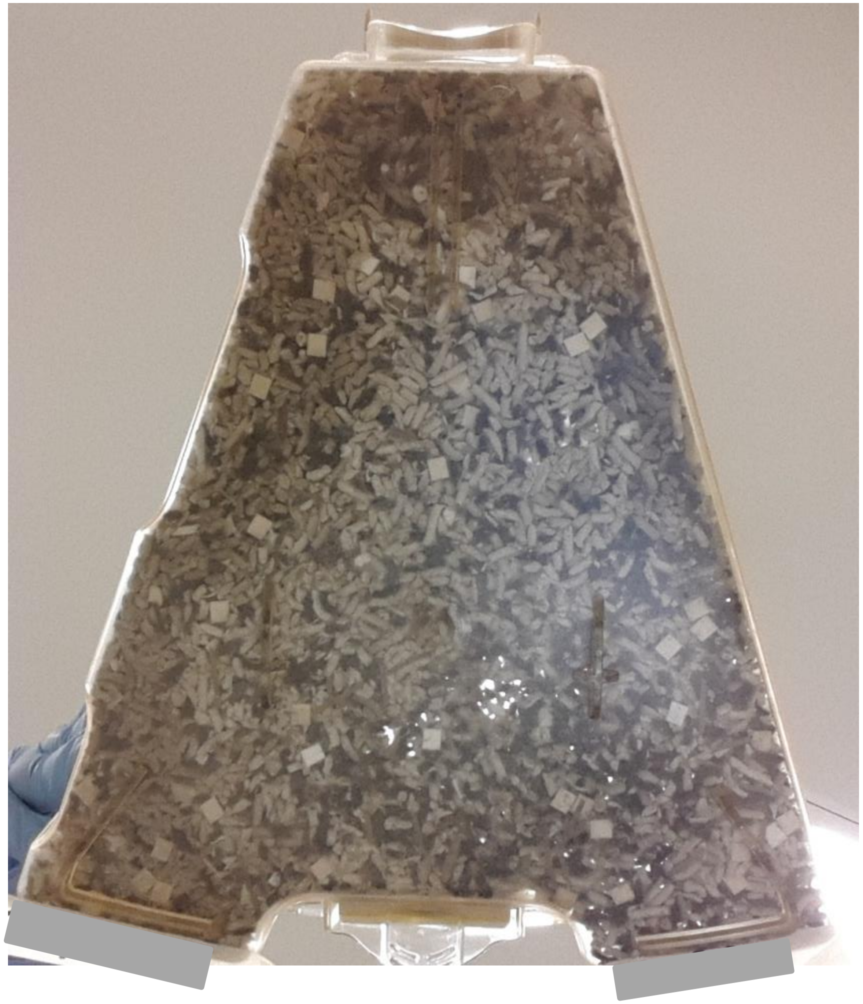

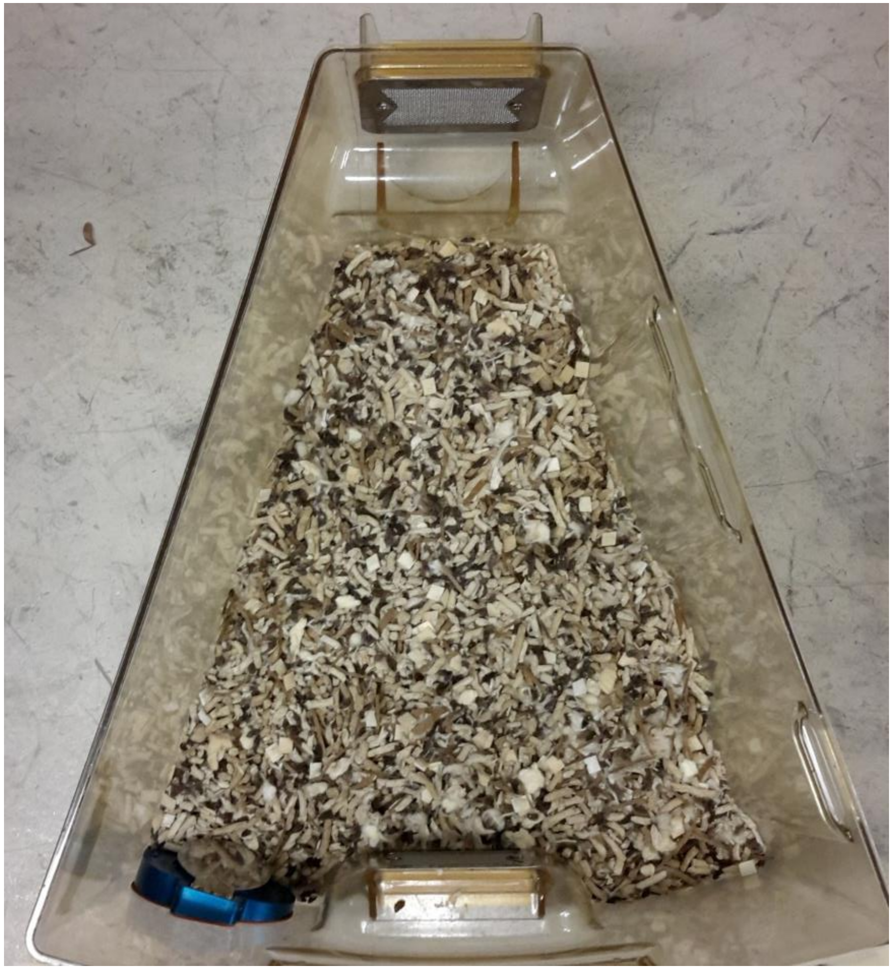

July 14 STD 1

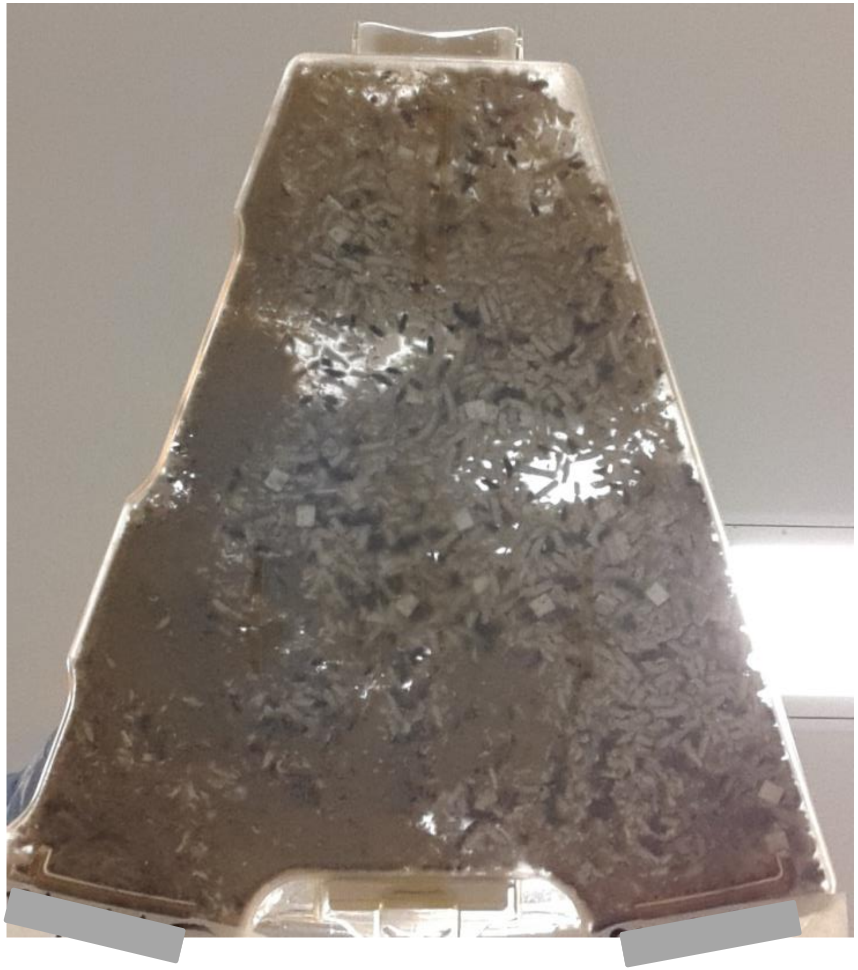

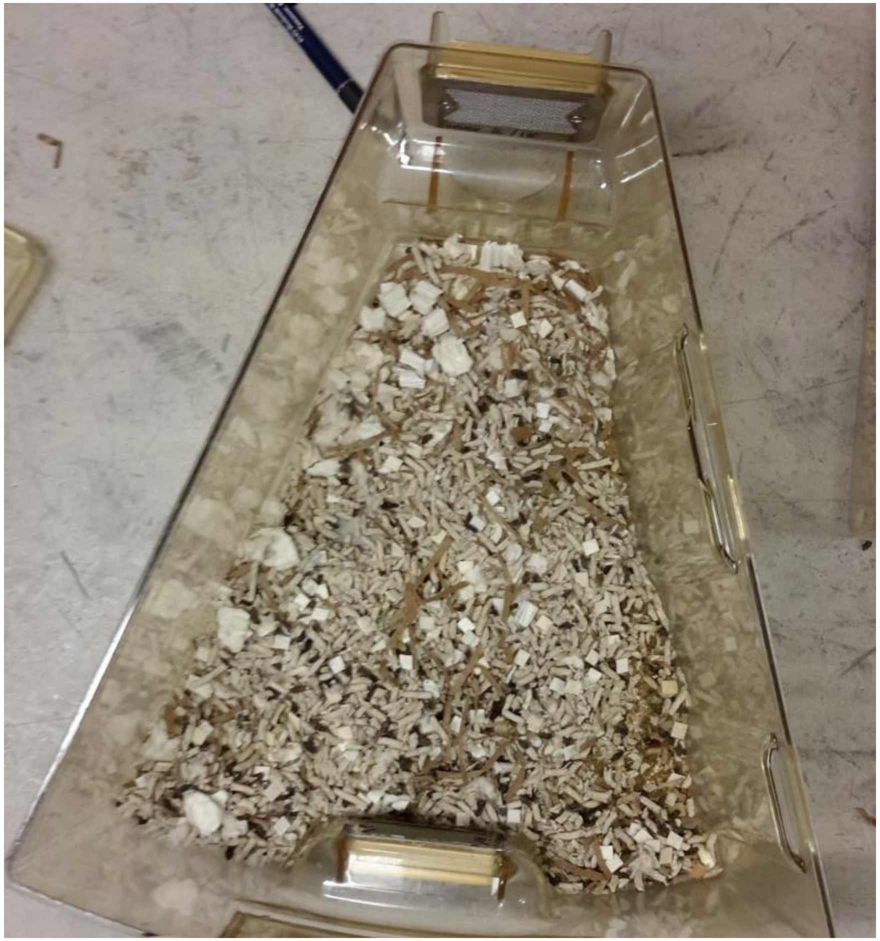

July 14 COMP 2 right

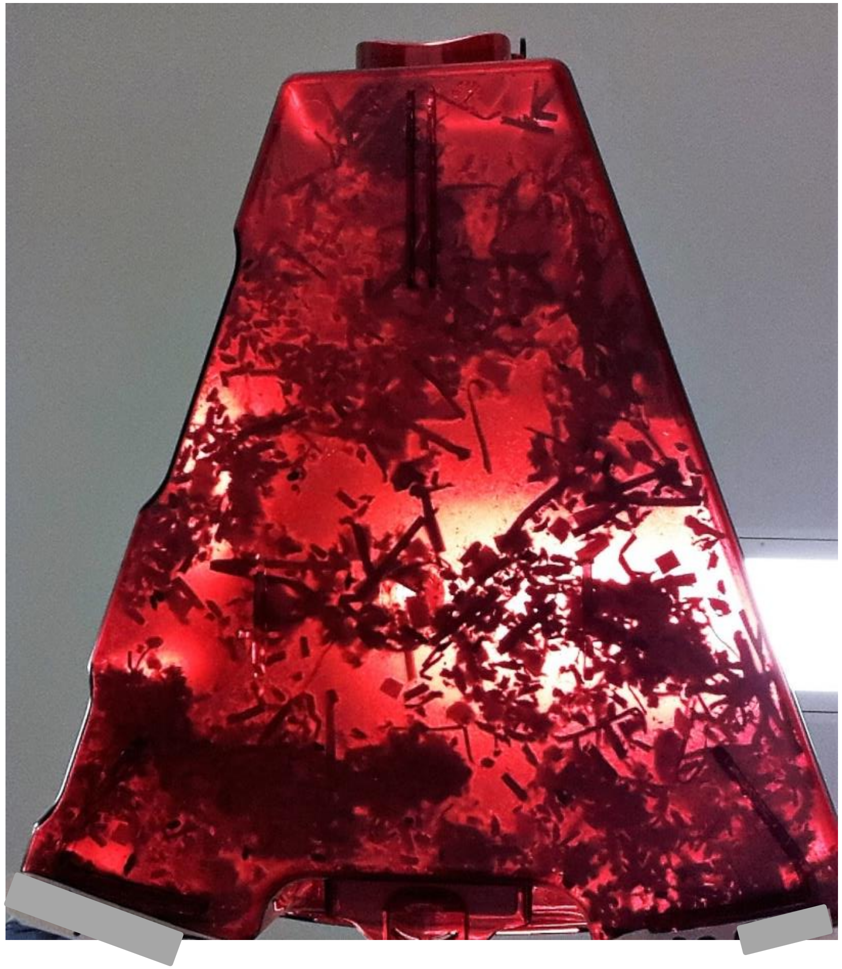

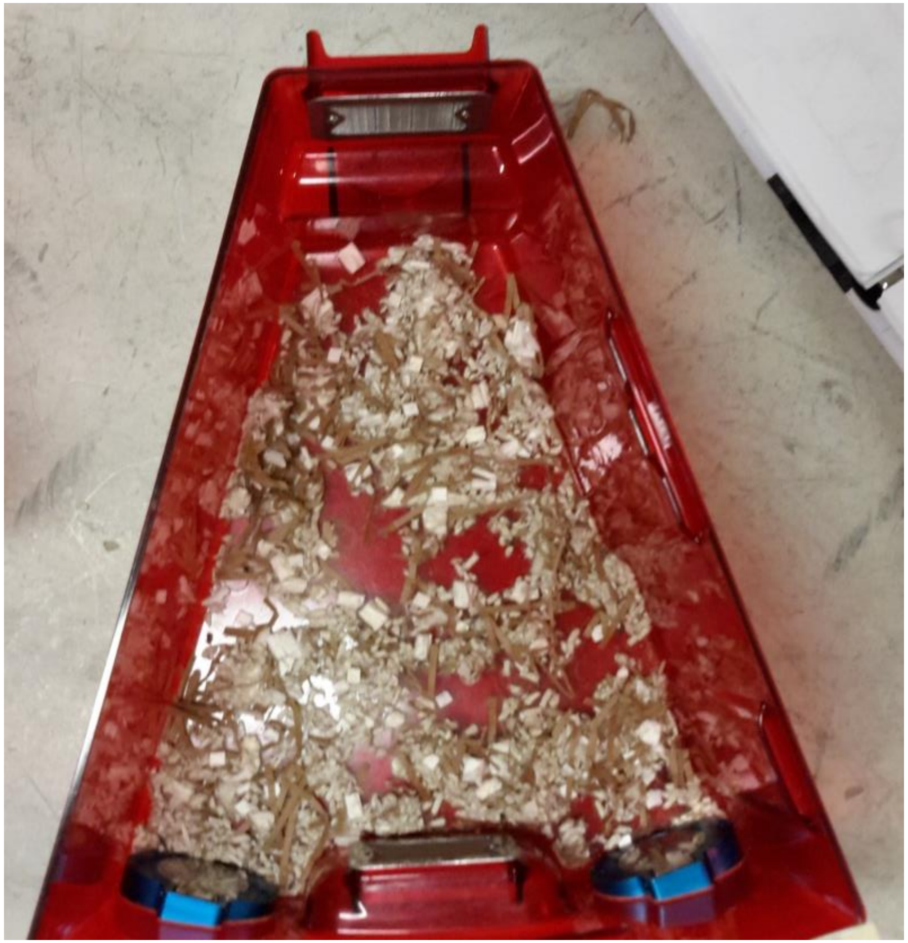

July 14 COMP 2 mid

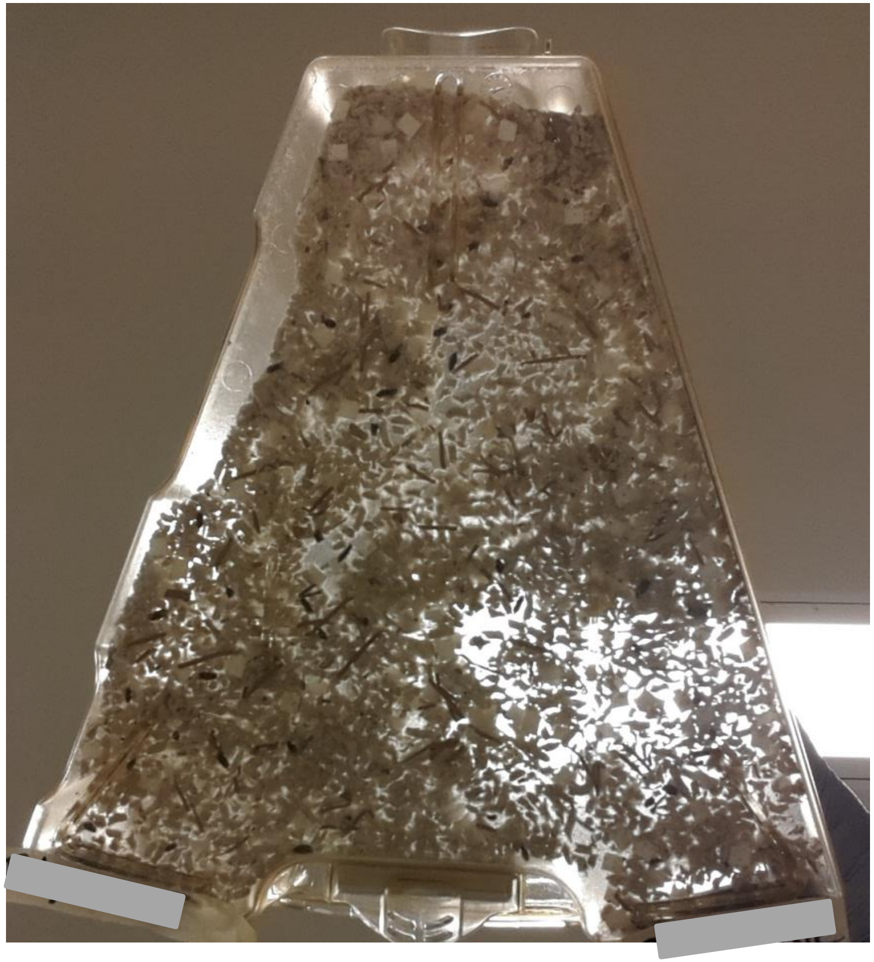

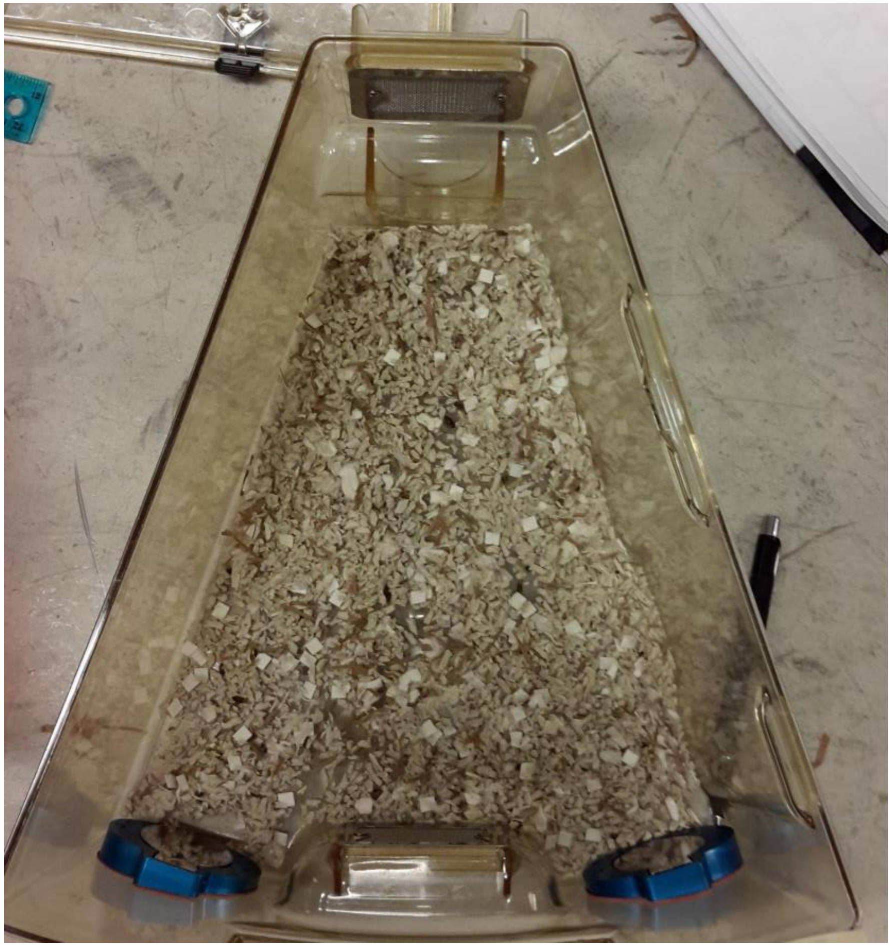

July 14 COMP 2 left

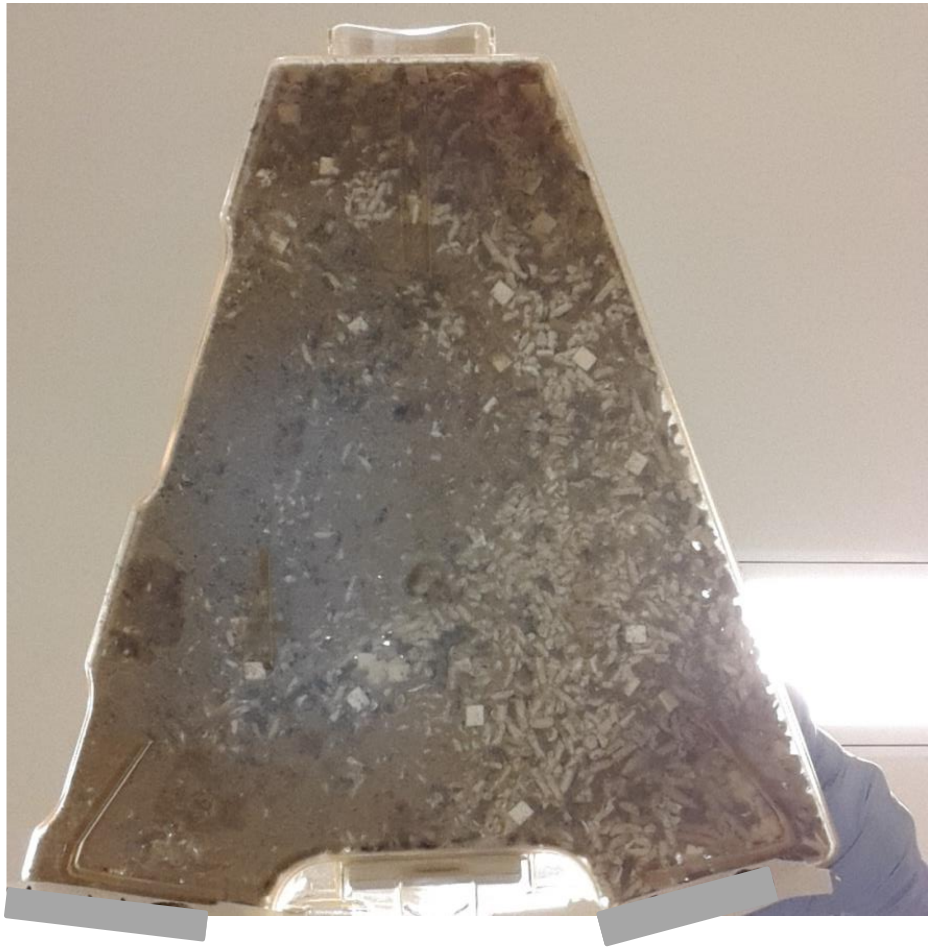

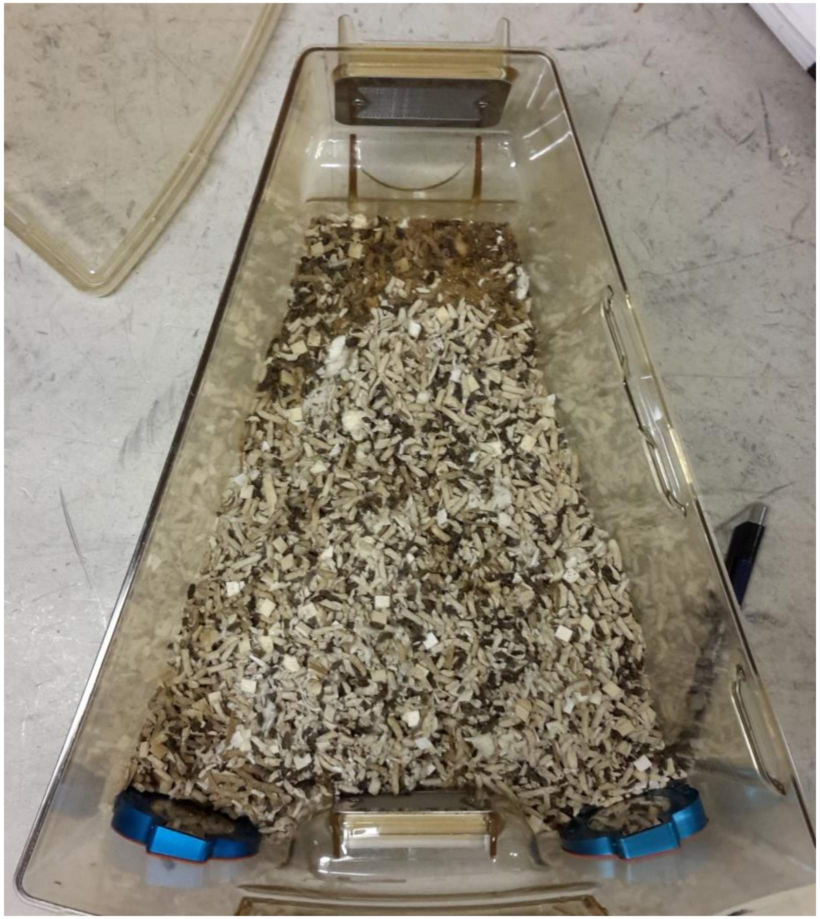

July 14 STD 2

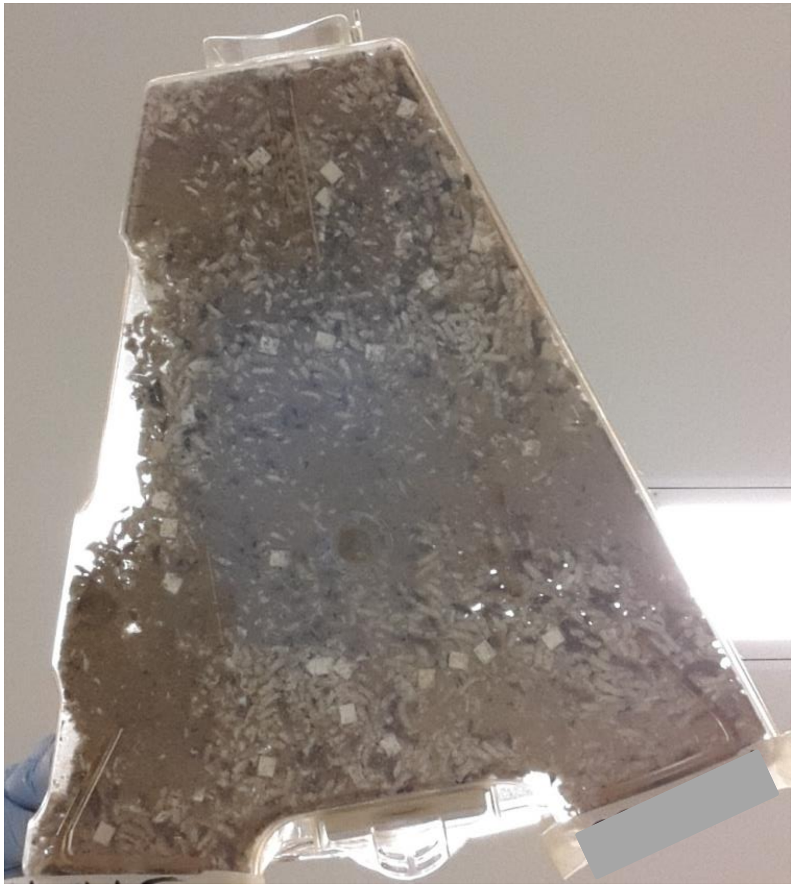

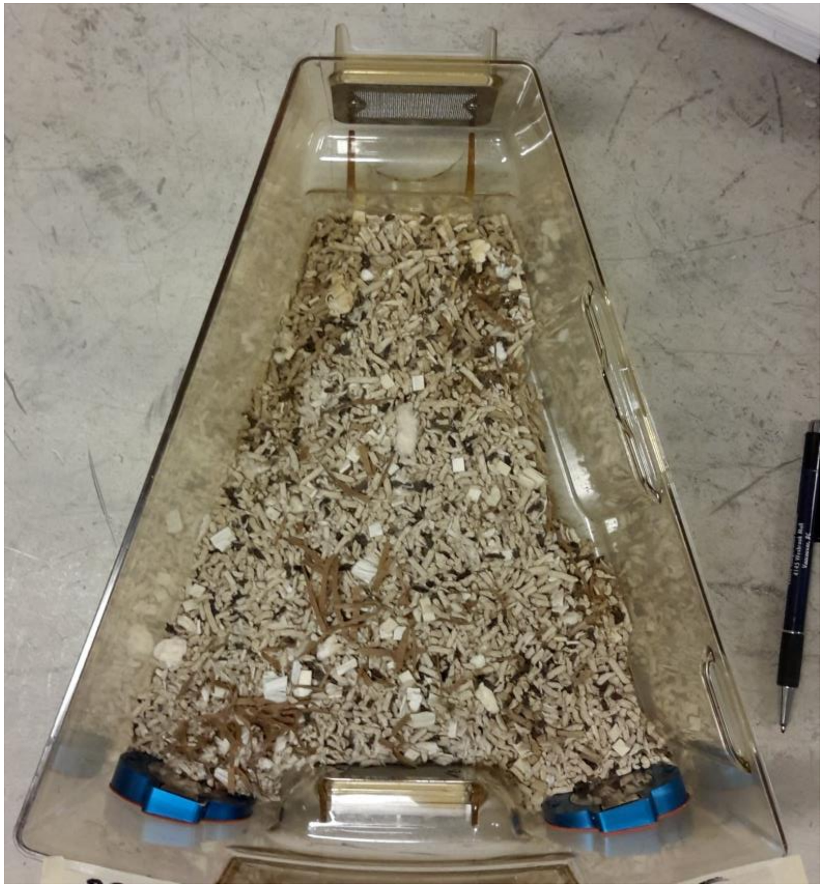

July 16 COMP 3 right

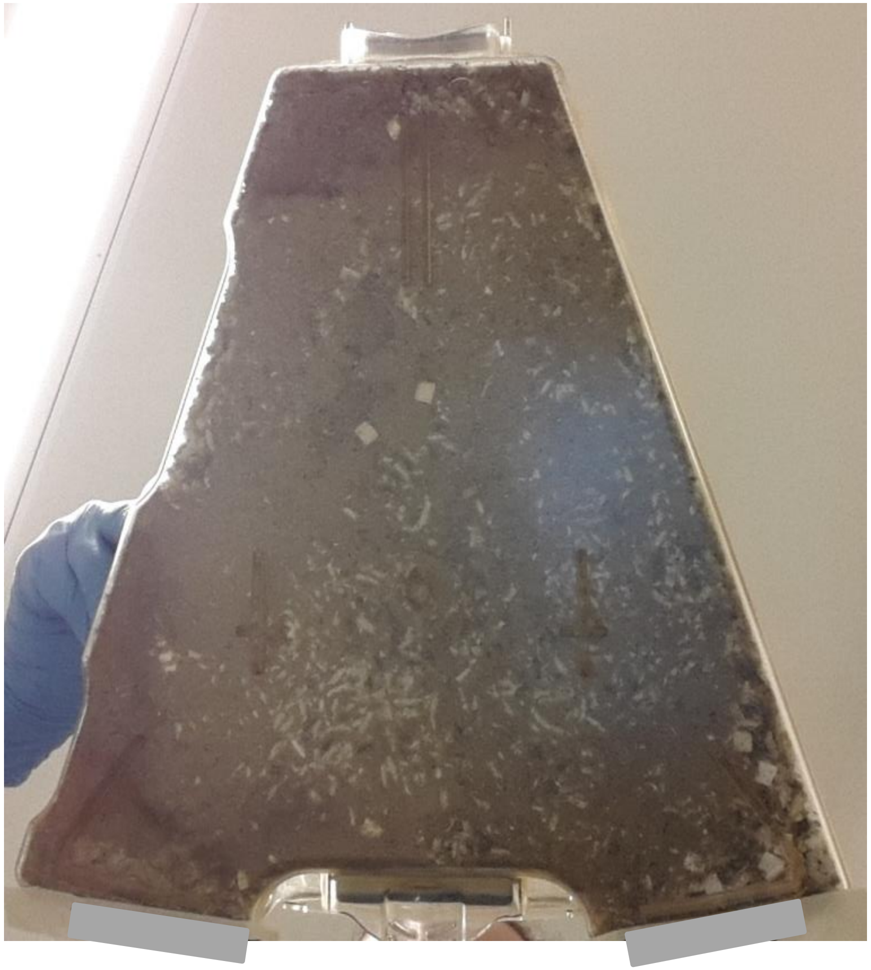

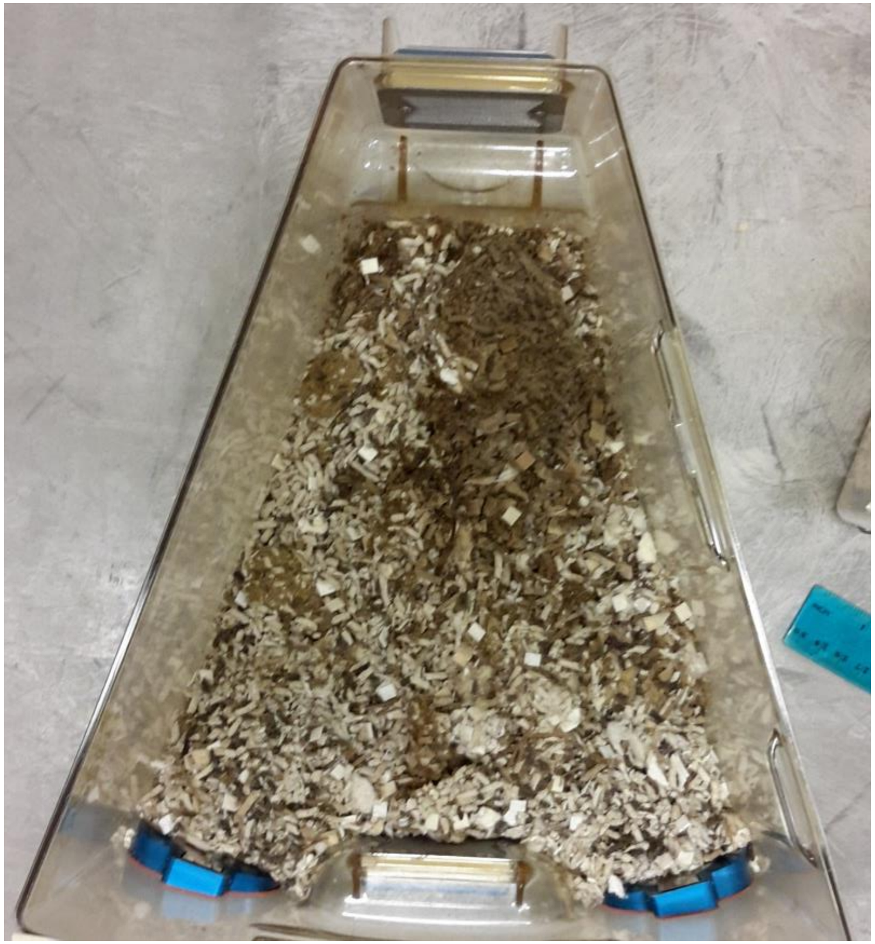

July 16 COMP 3 mid

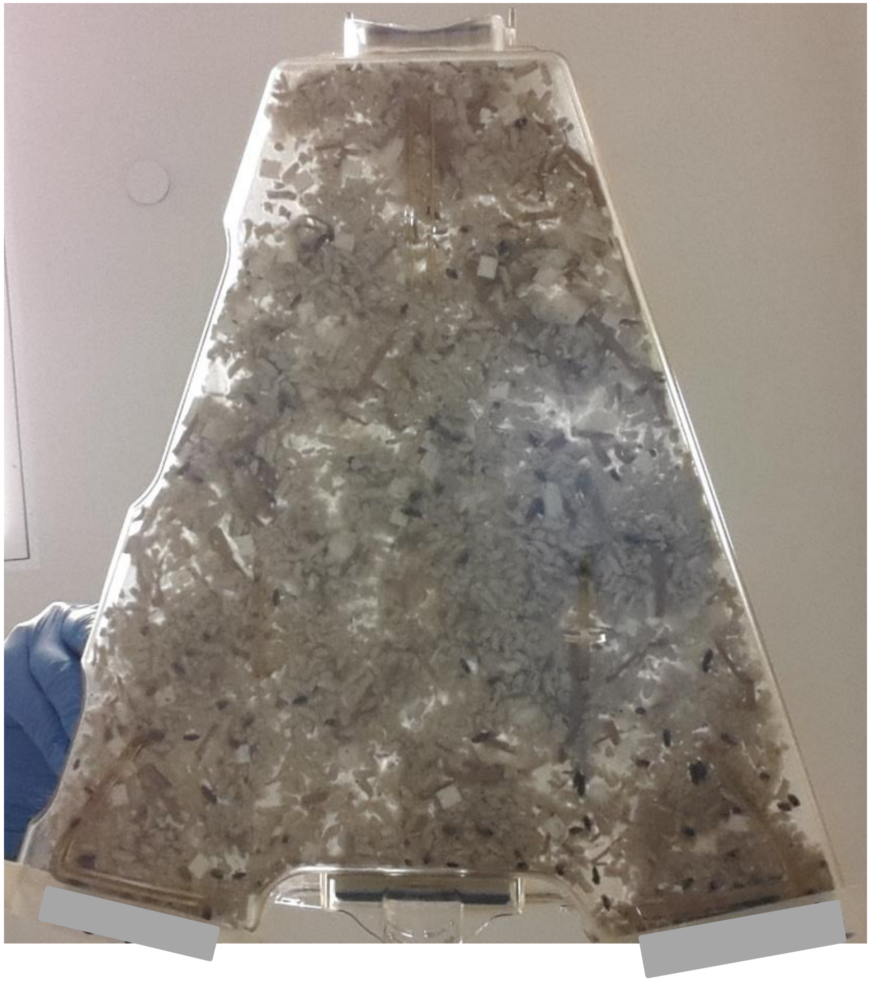

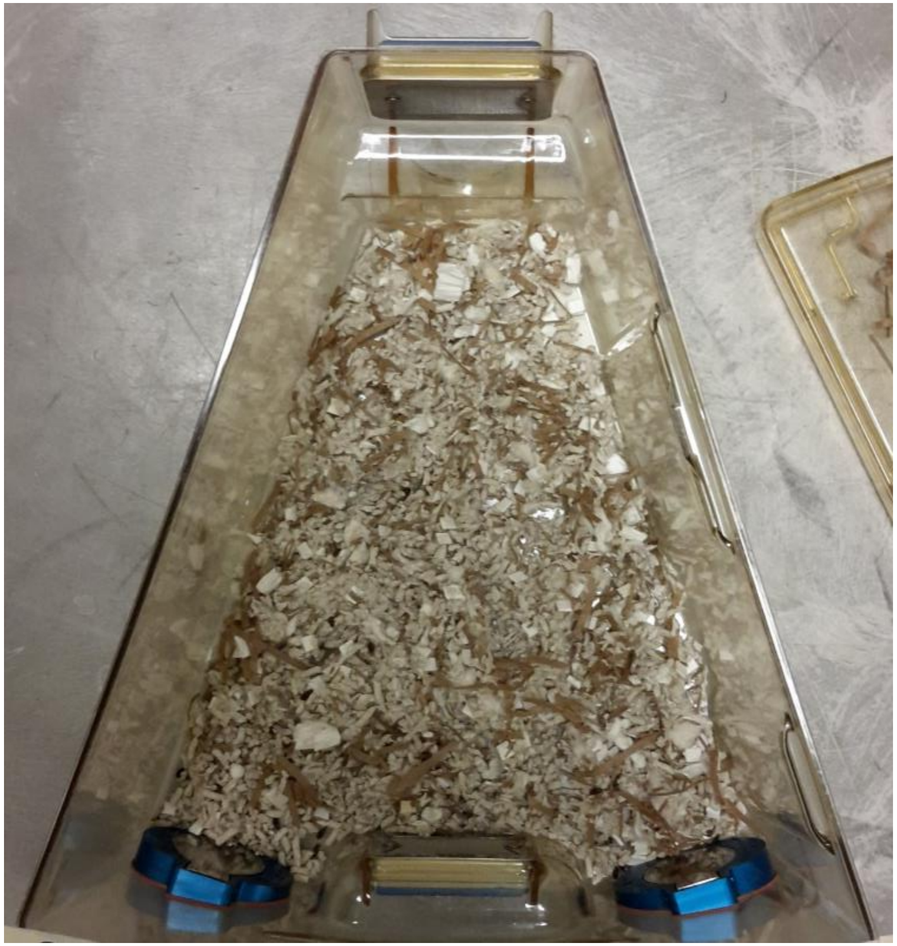

July 16 COMP 3 left

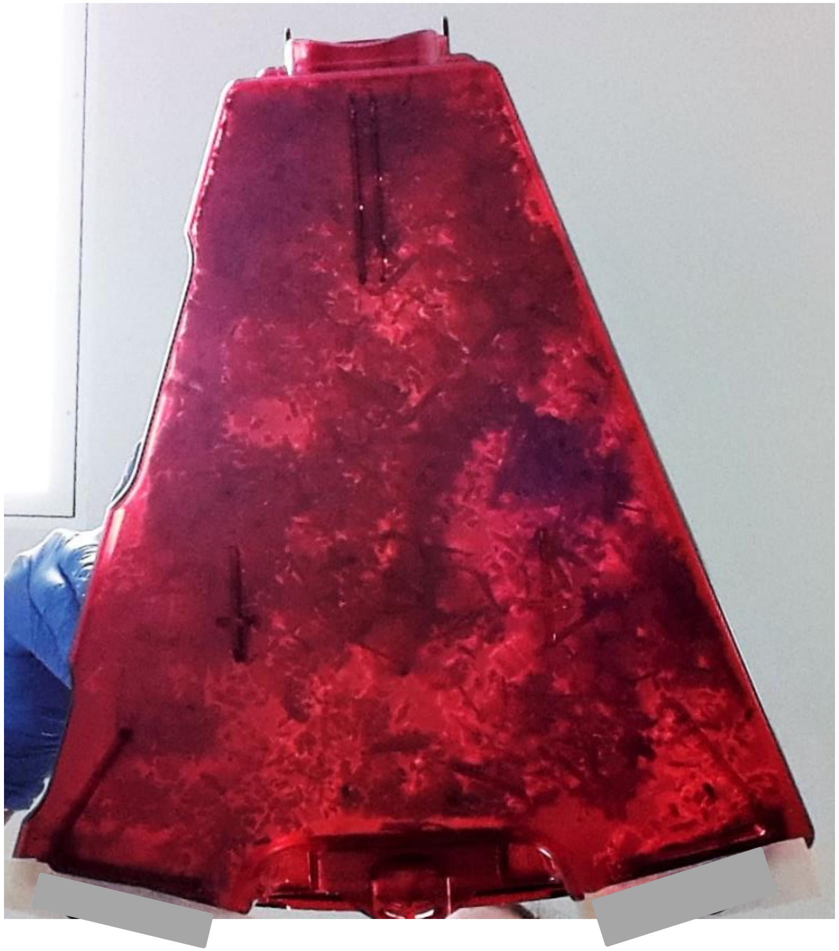

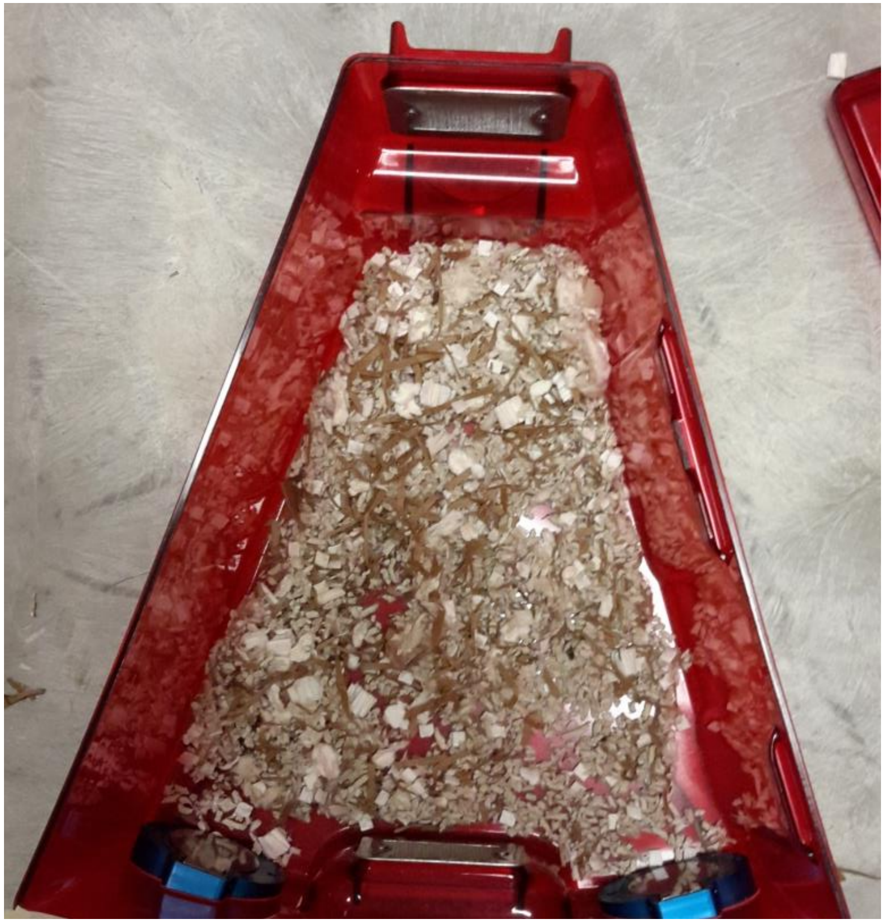

July 16 STD 3

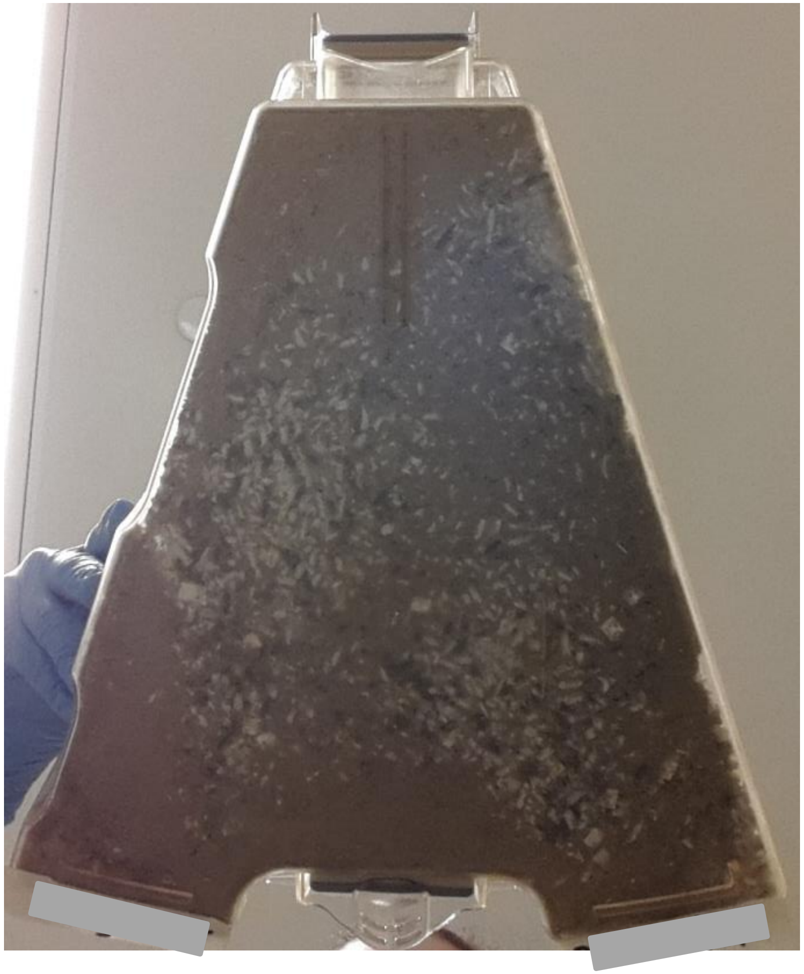

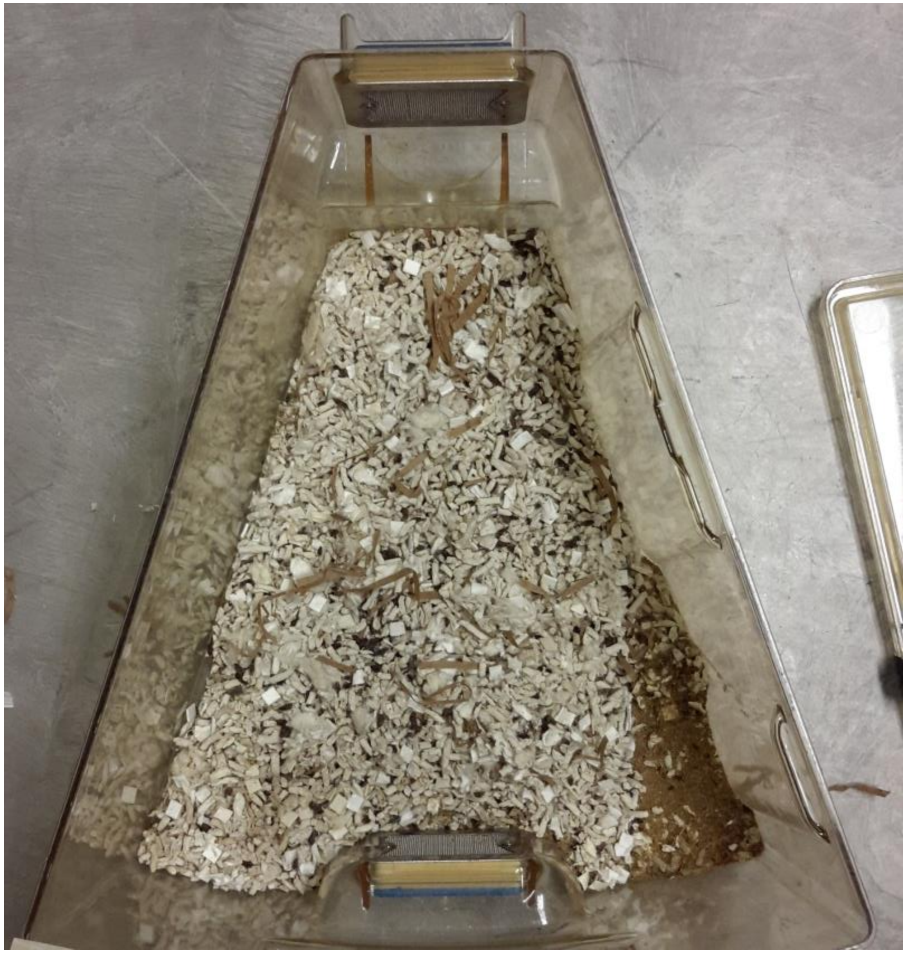

July 16 COMP 4 left

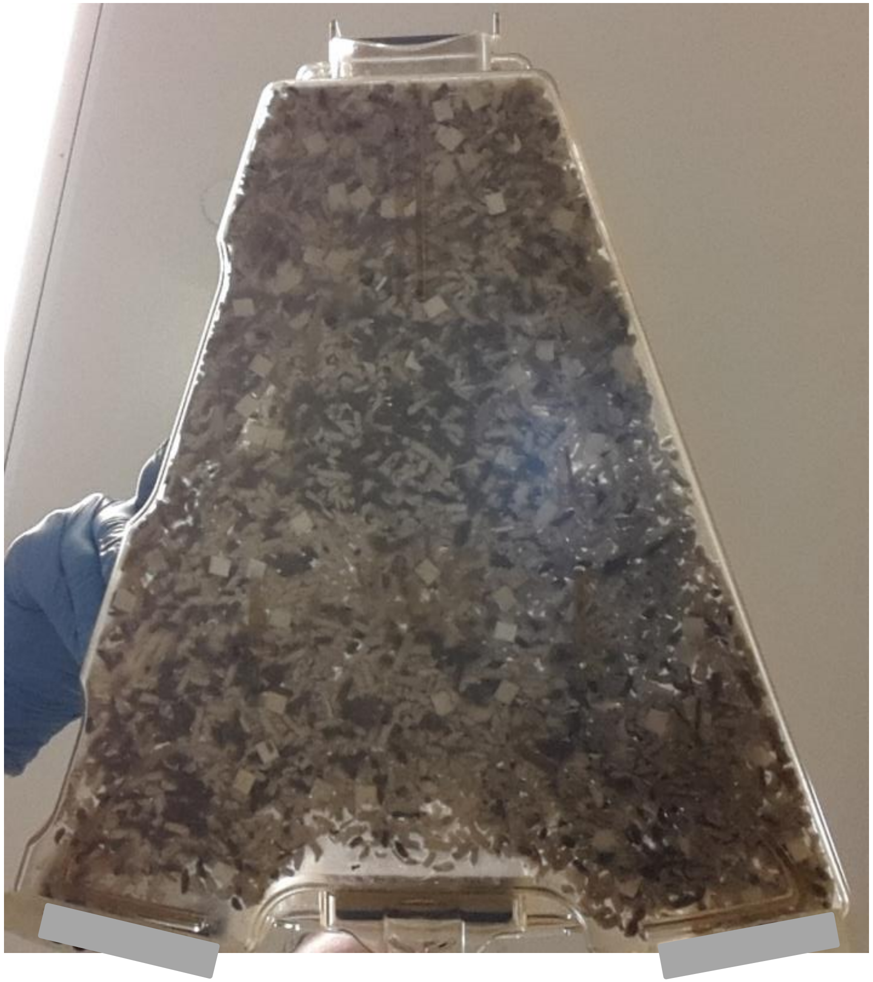

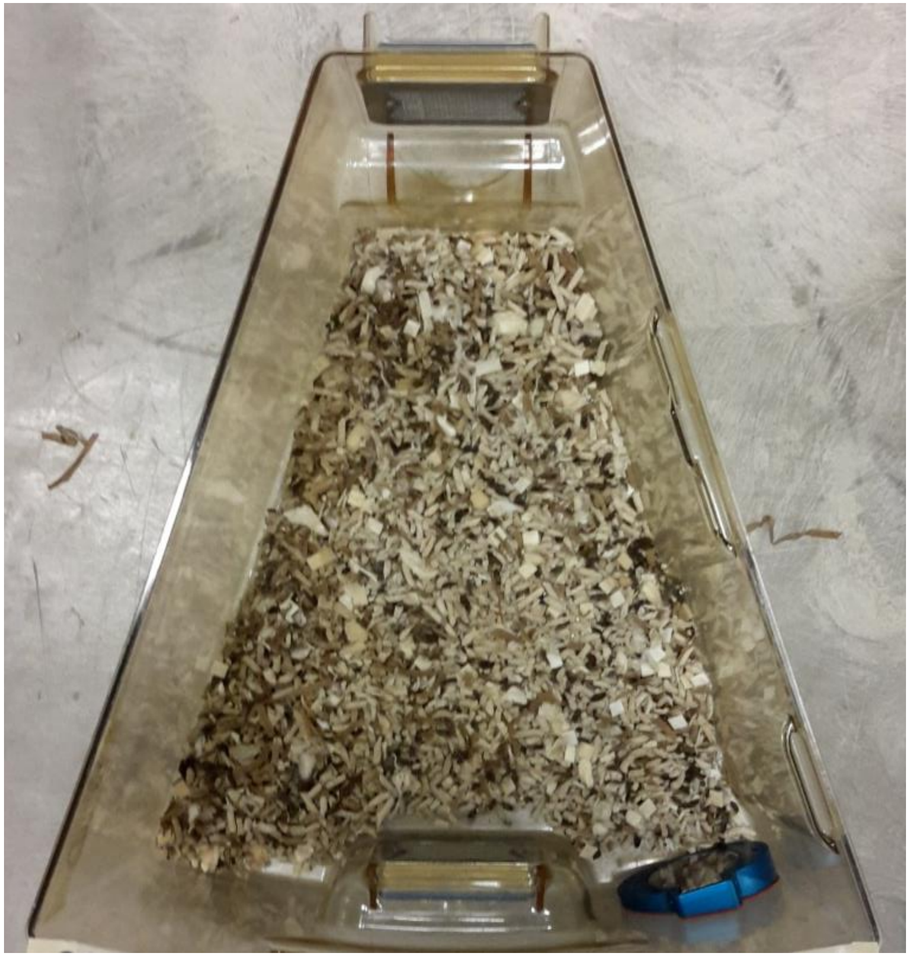

July 16 COMP 4 mid

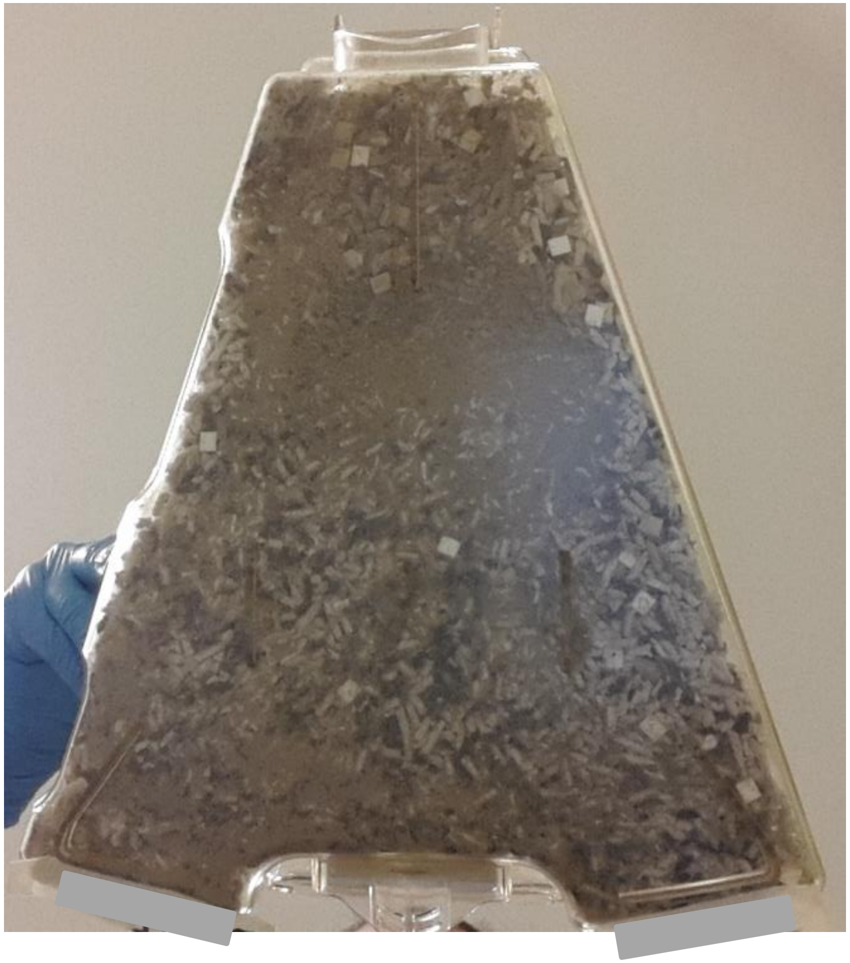

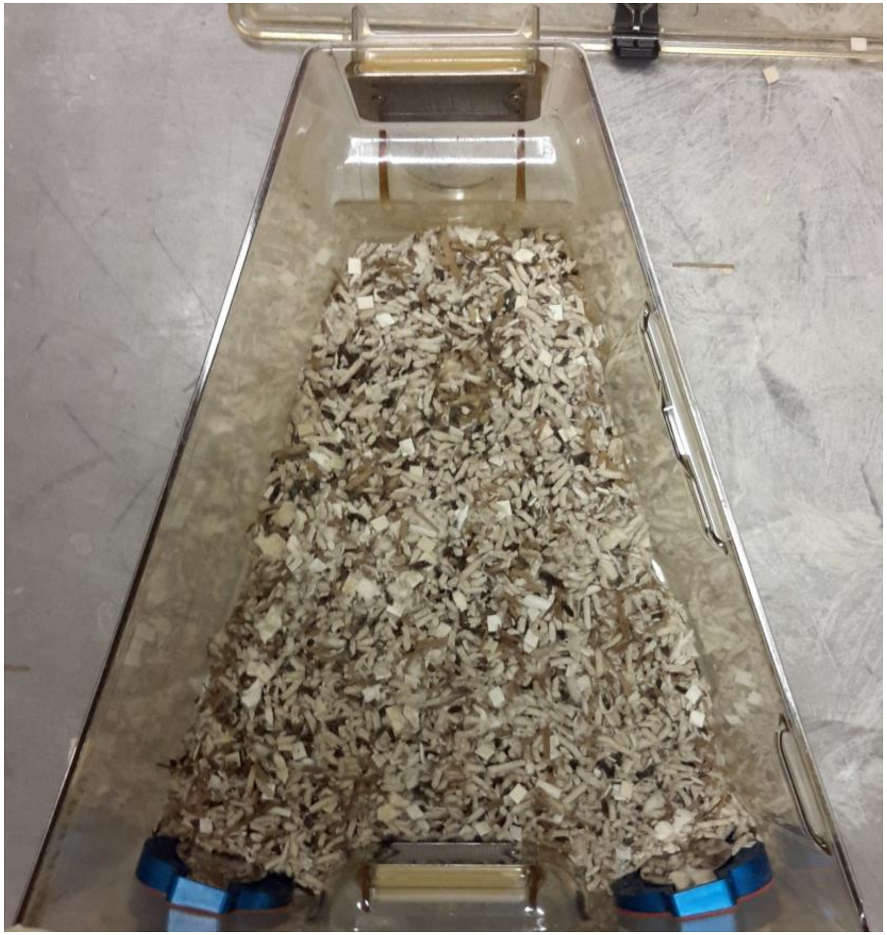

July 16 COMP 4 right

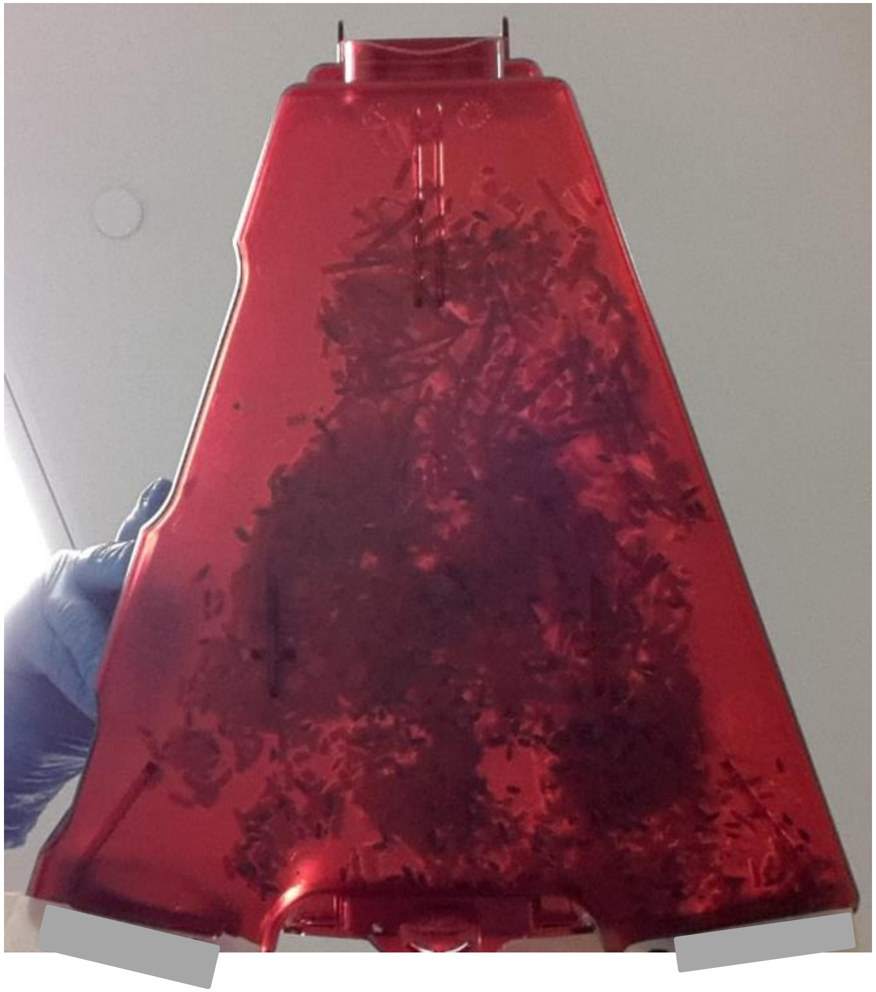

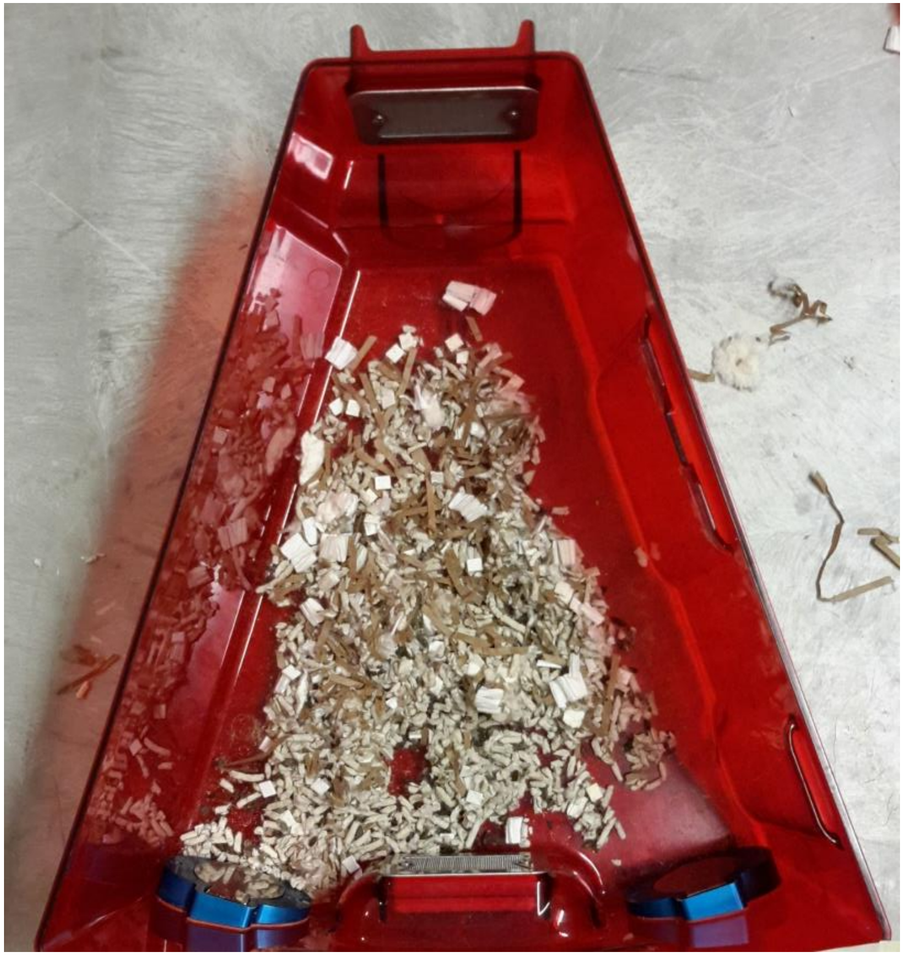

July 16 STD 4

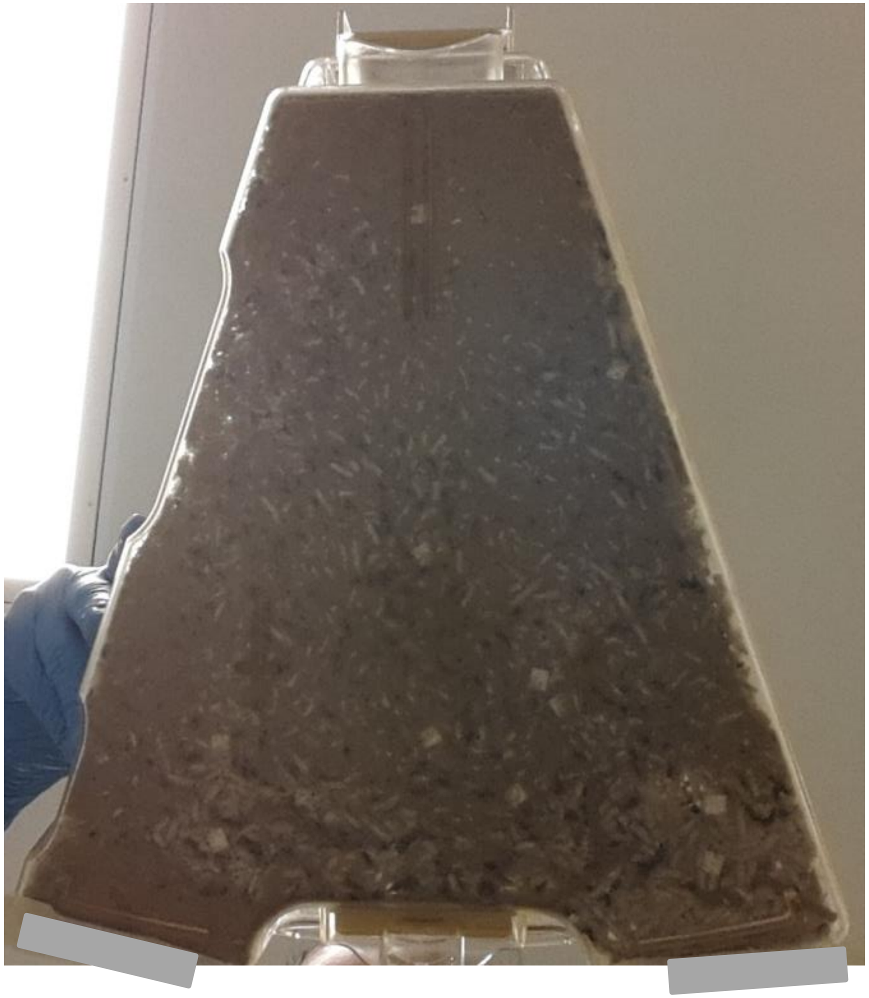

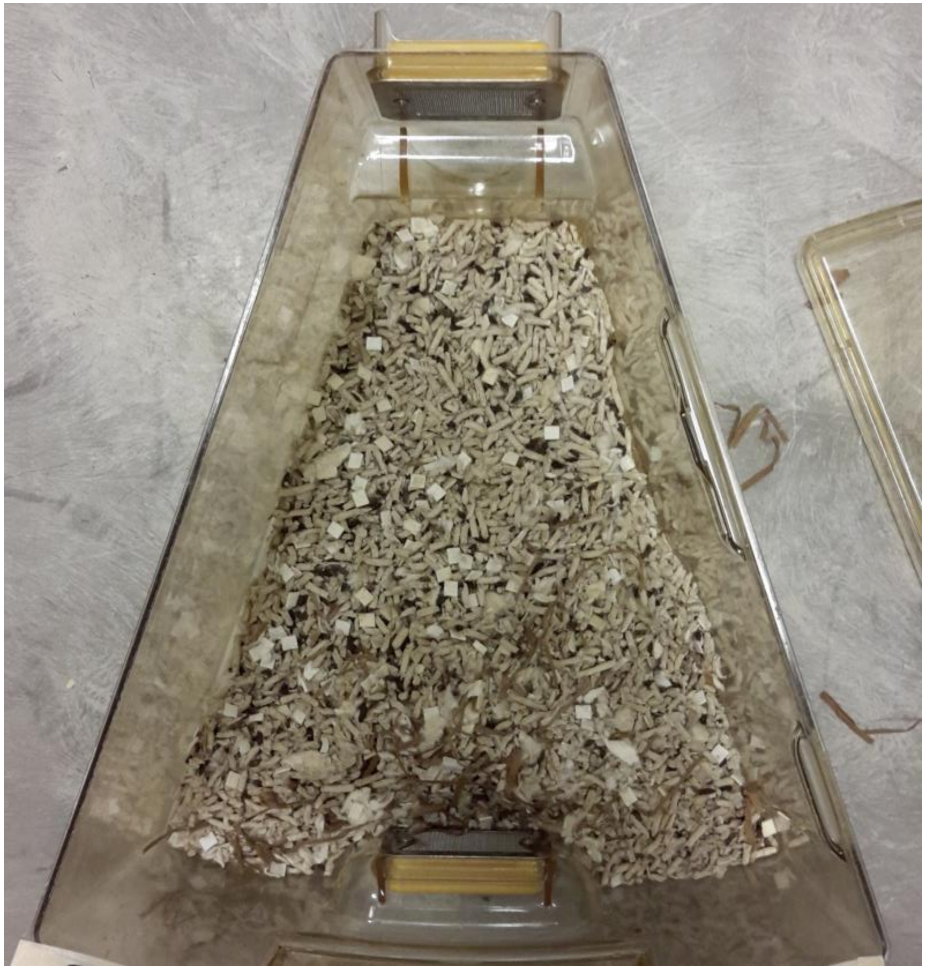

July 16 COMP 5 right

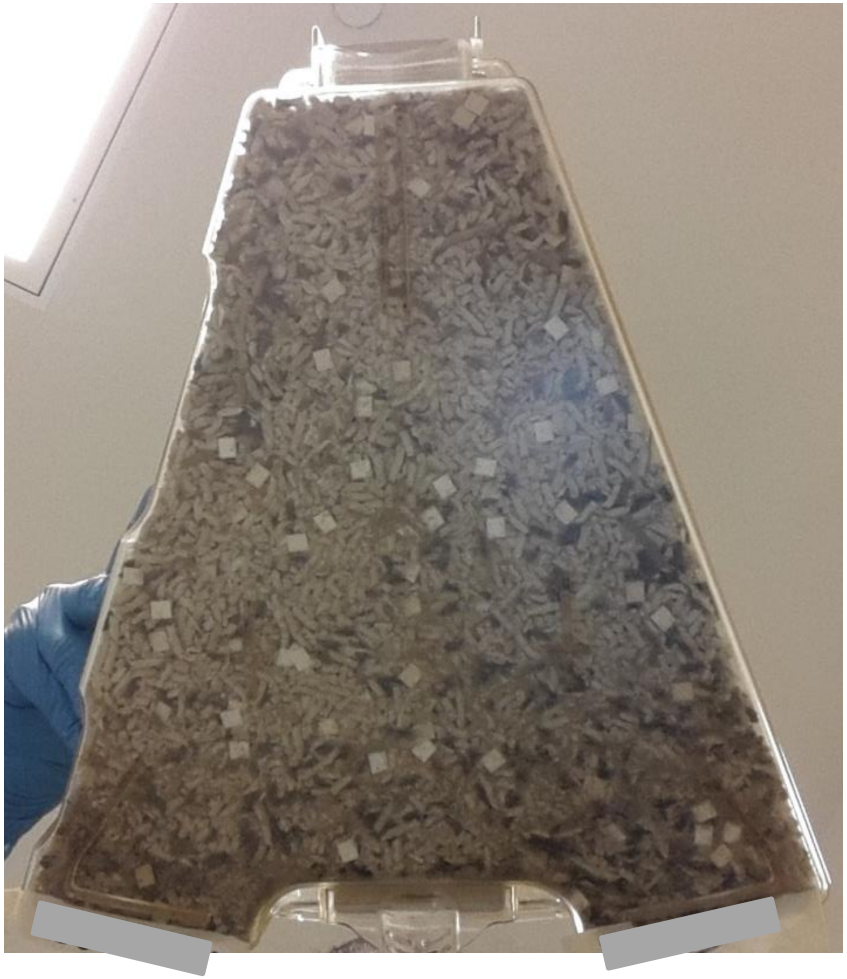

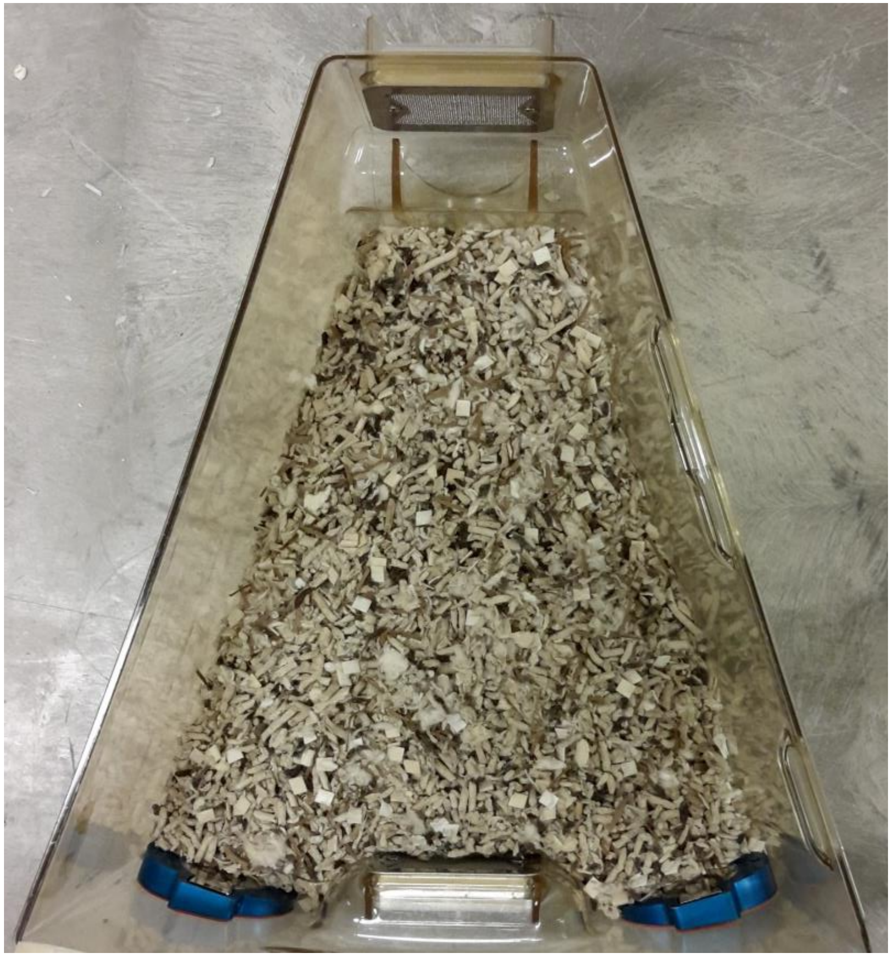

July 16 COMP 5 mid

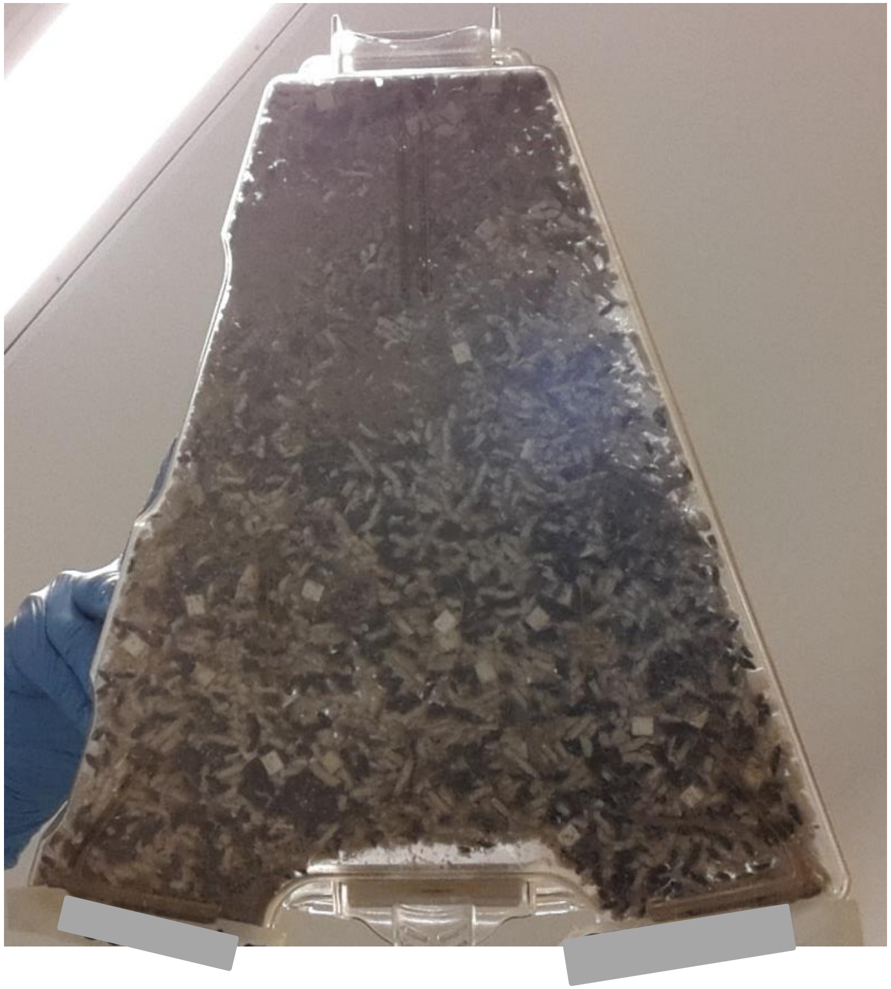

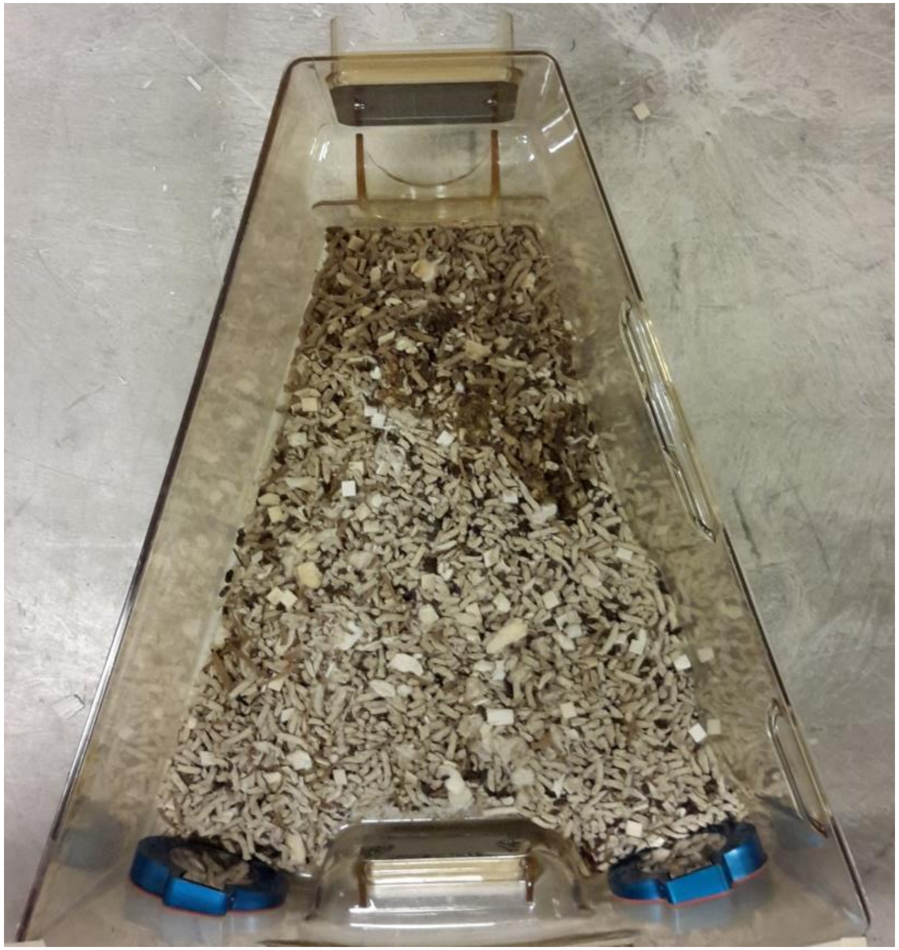

July 16 COMP 5 left

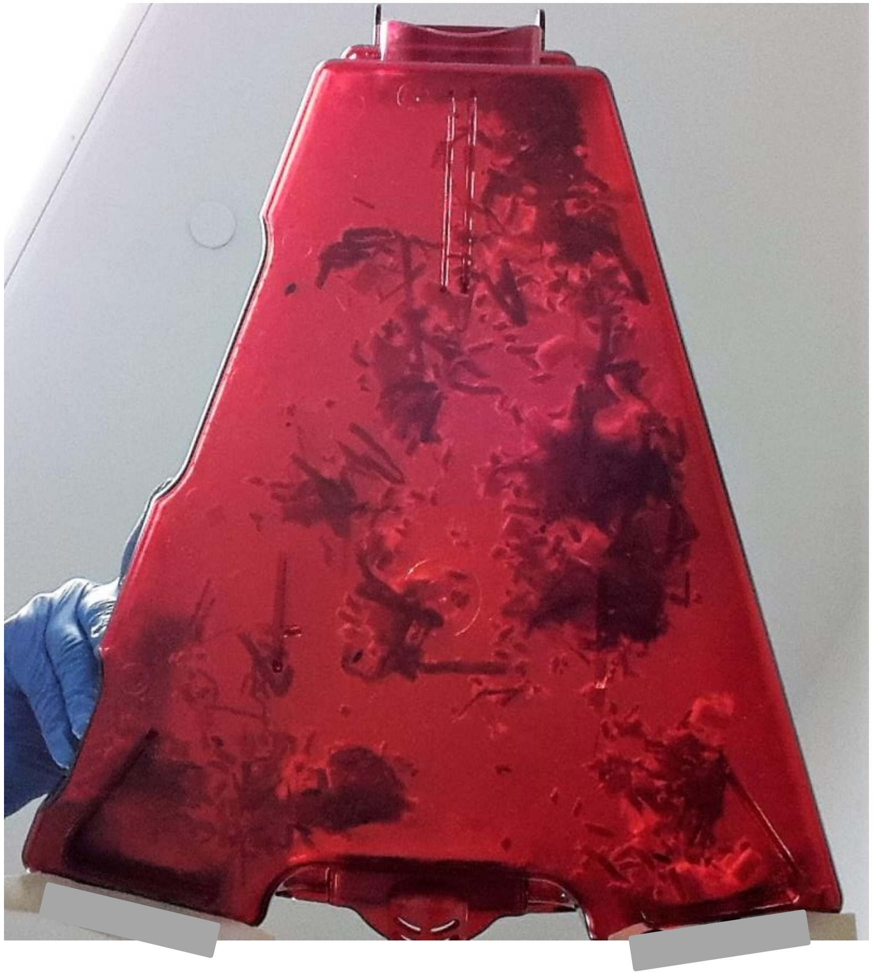

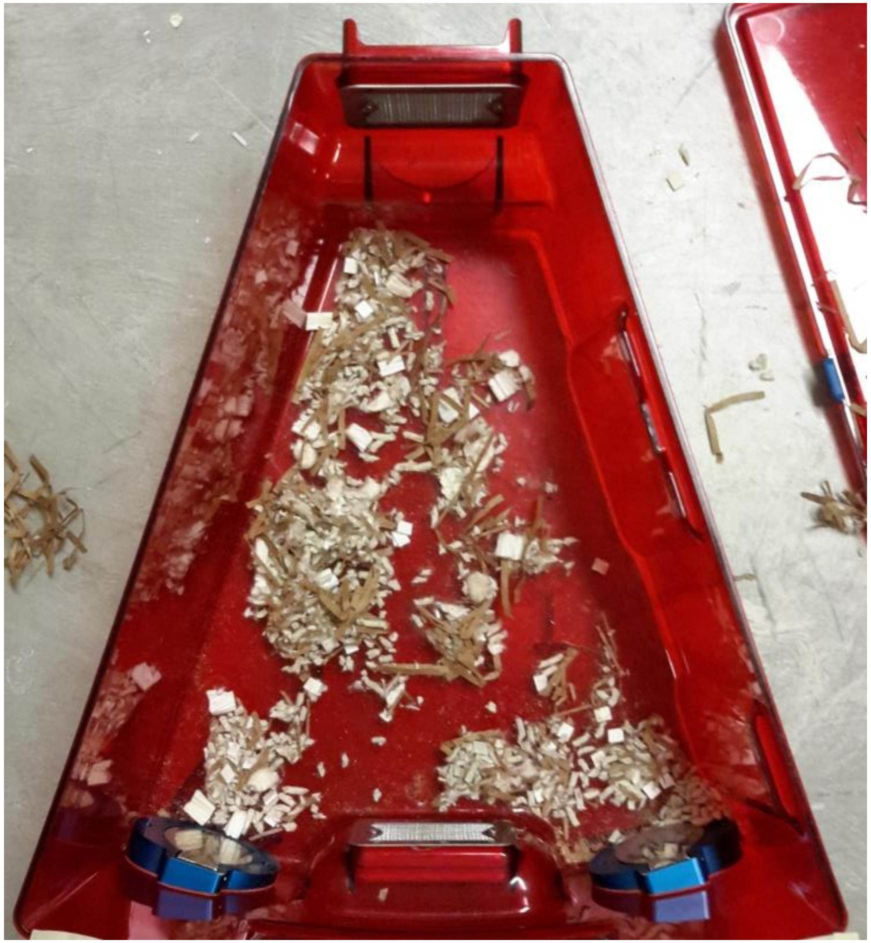

July 16 STD 5

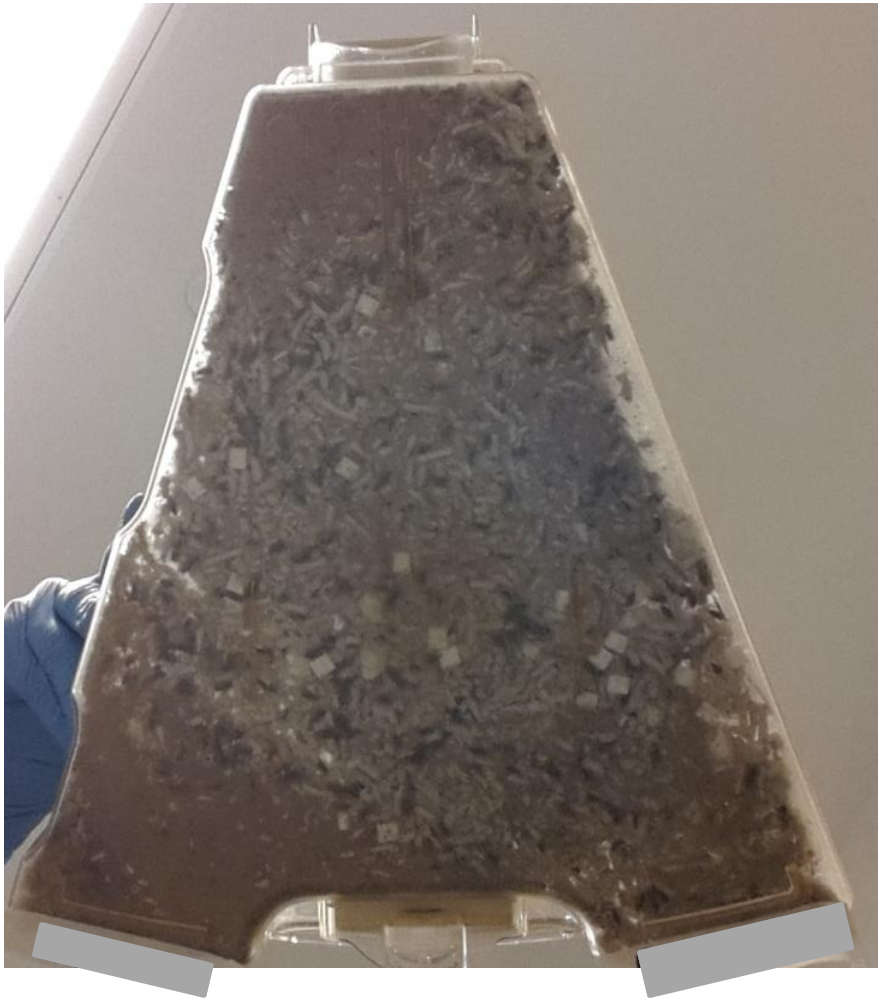

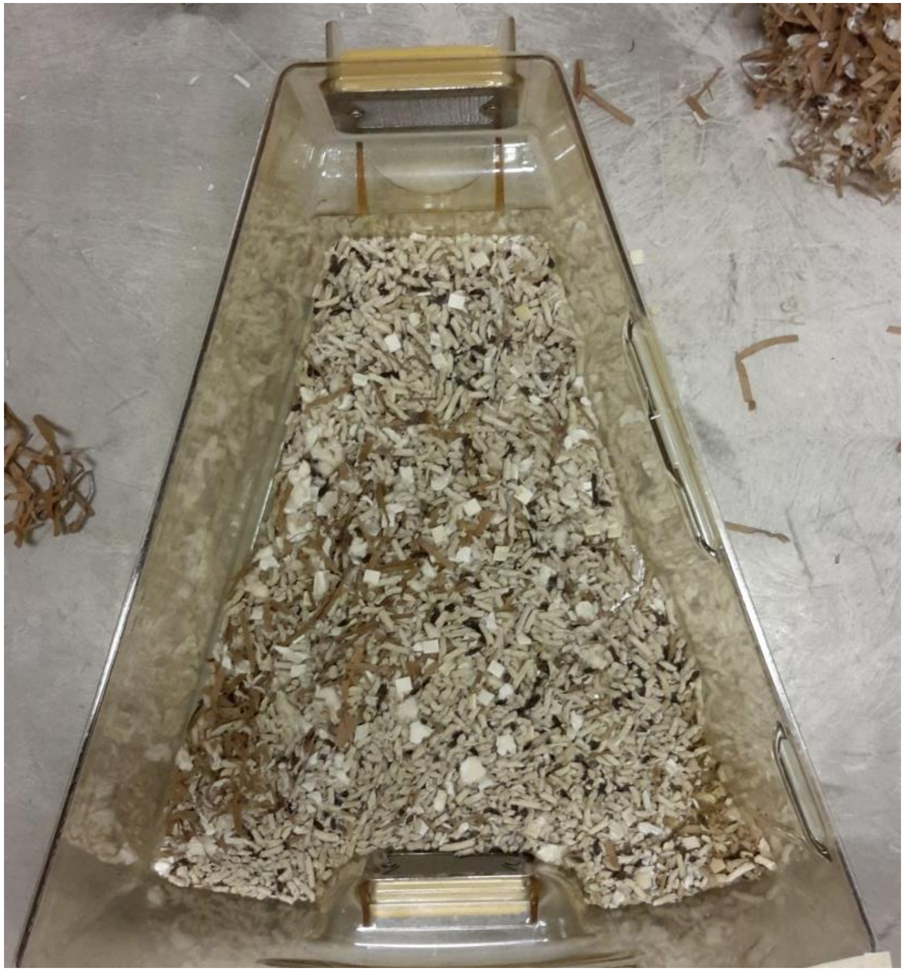

July 21 COMP 1 right

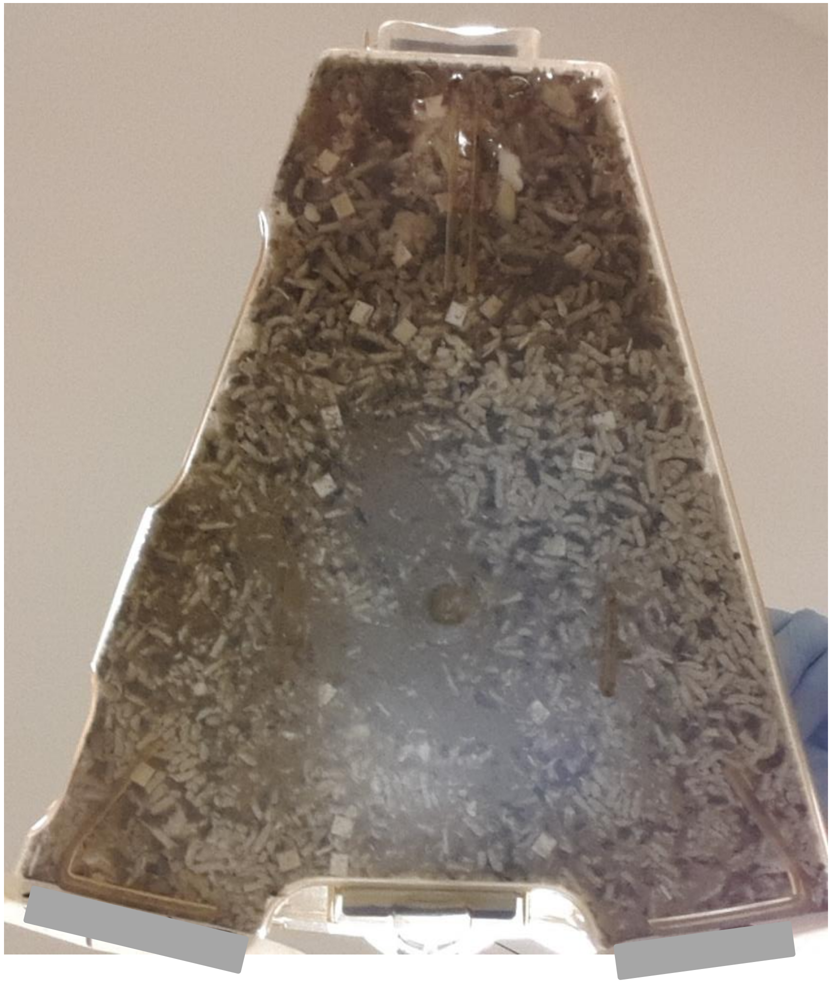

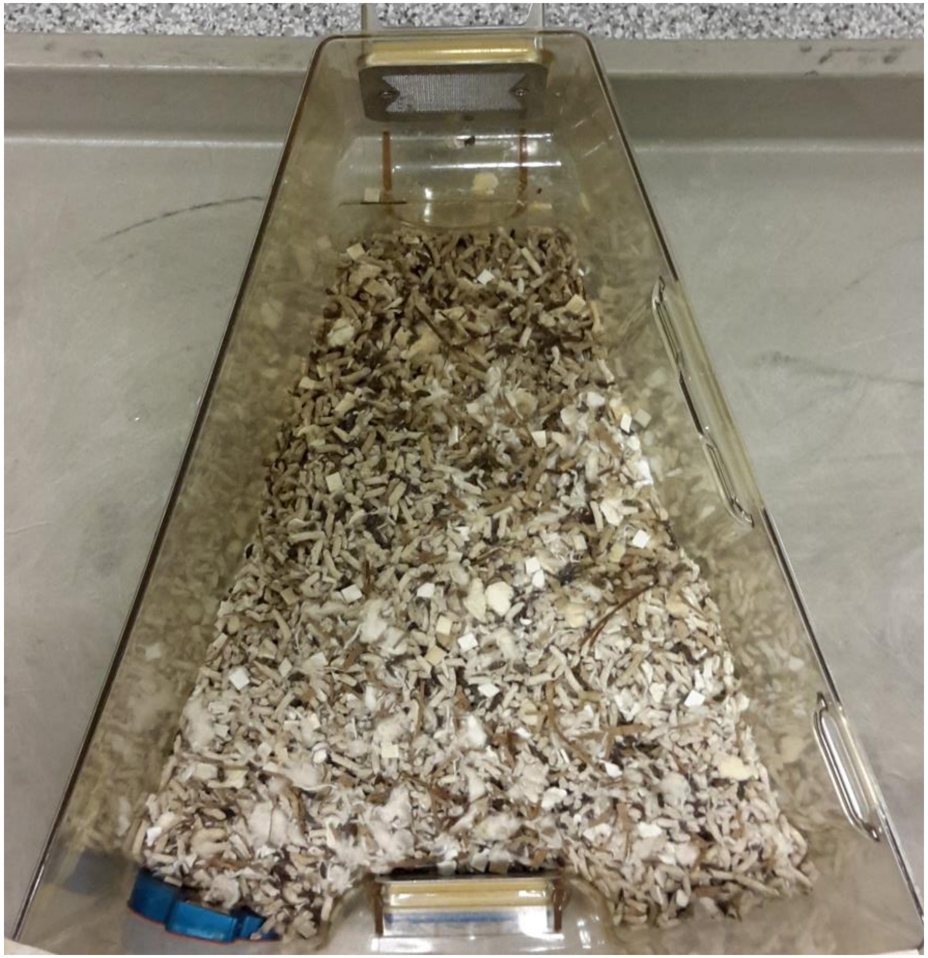

July 21 STD 1

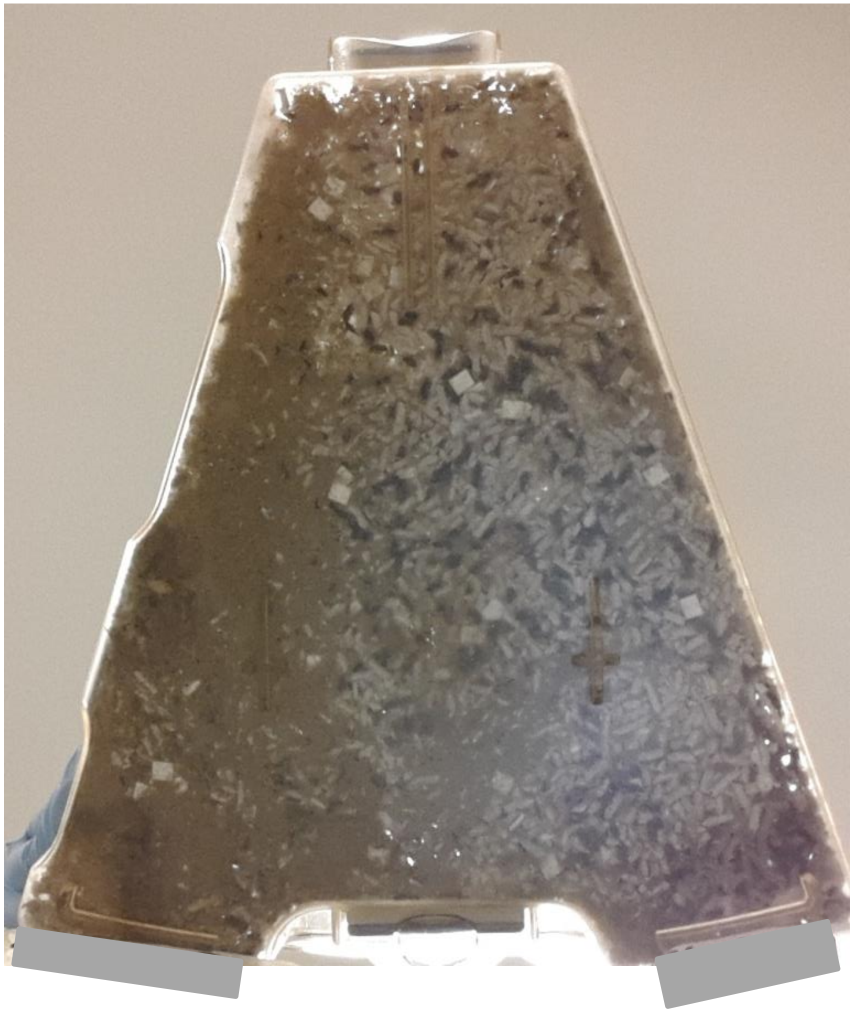

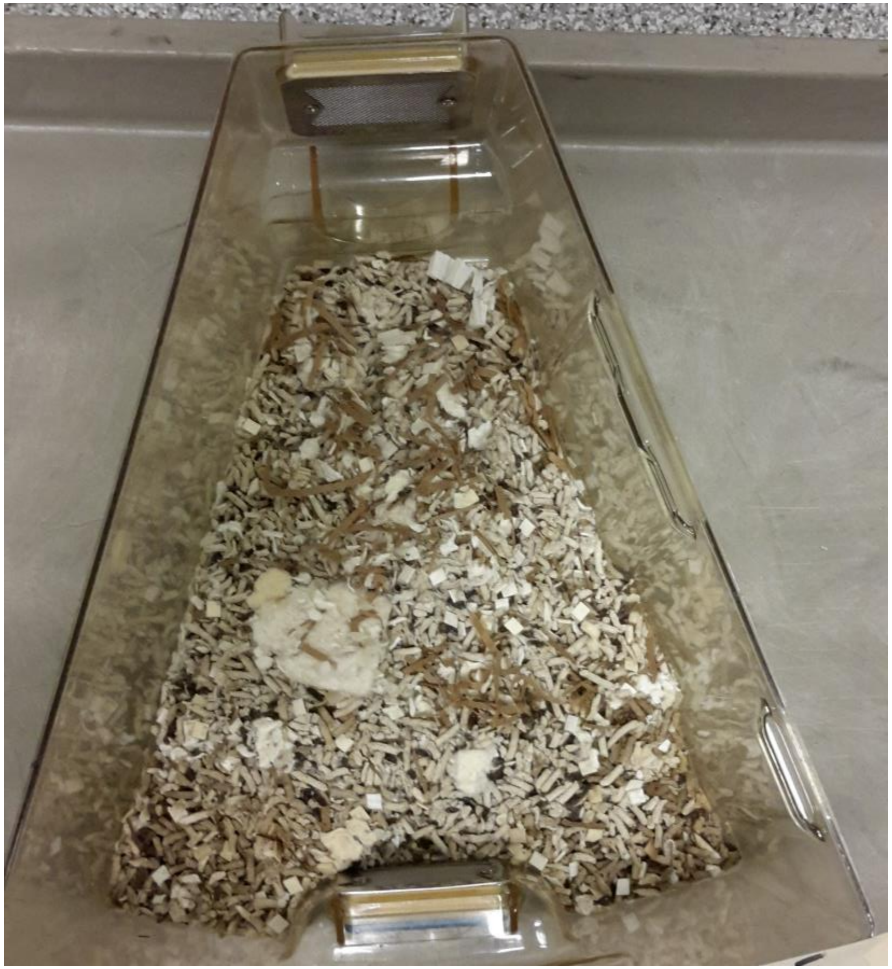

July 21 COMP 2 mid

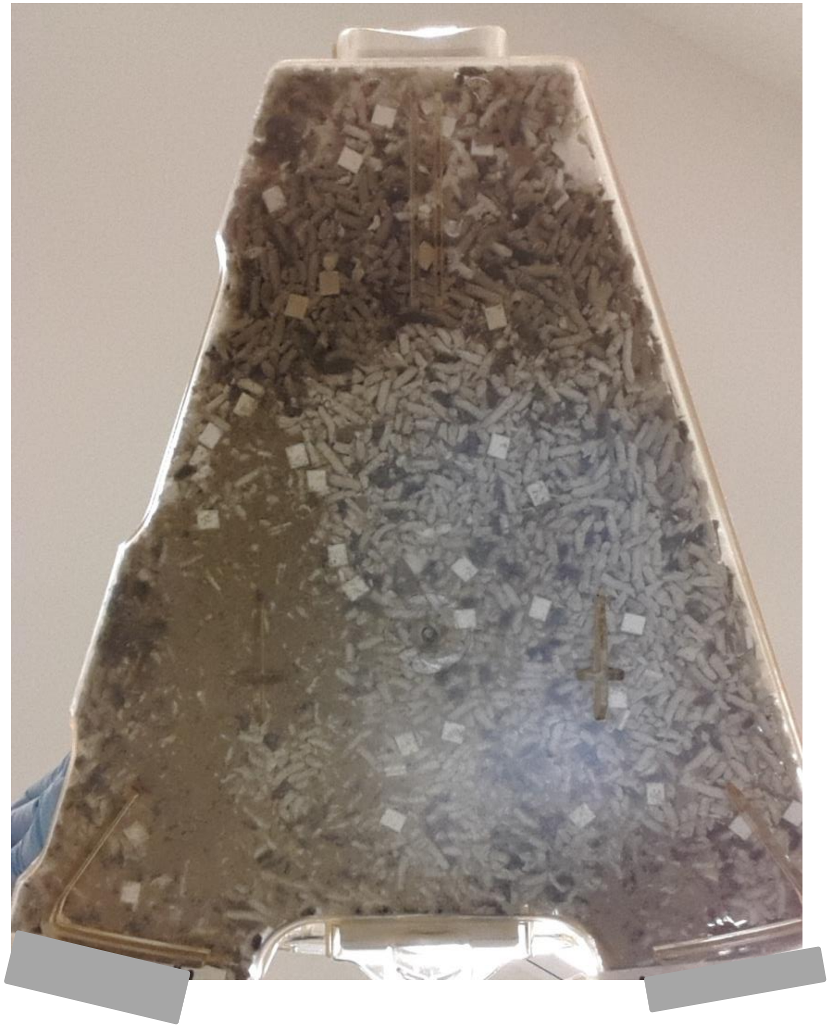

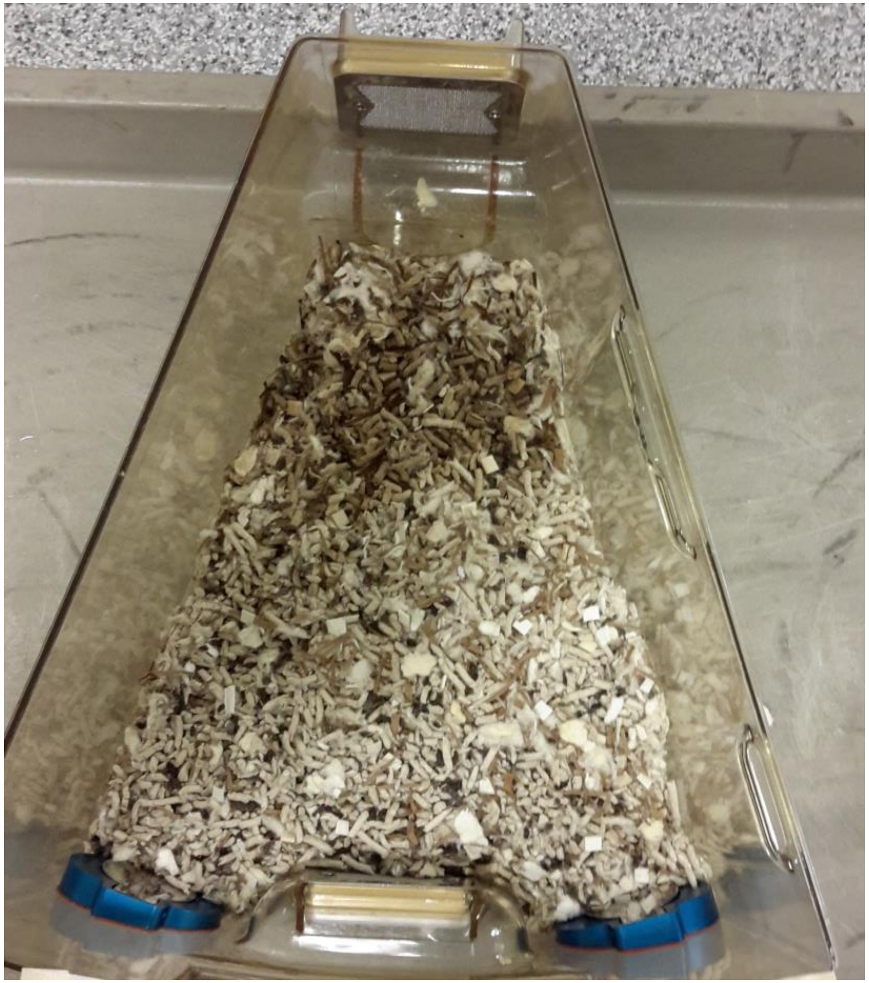

July 21 STD 2

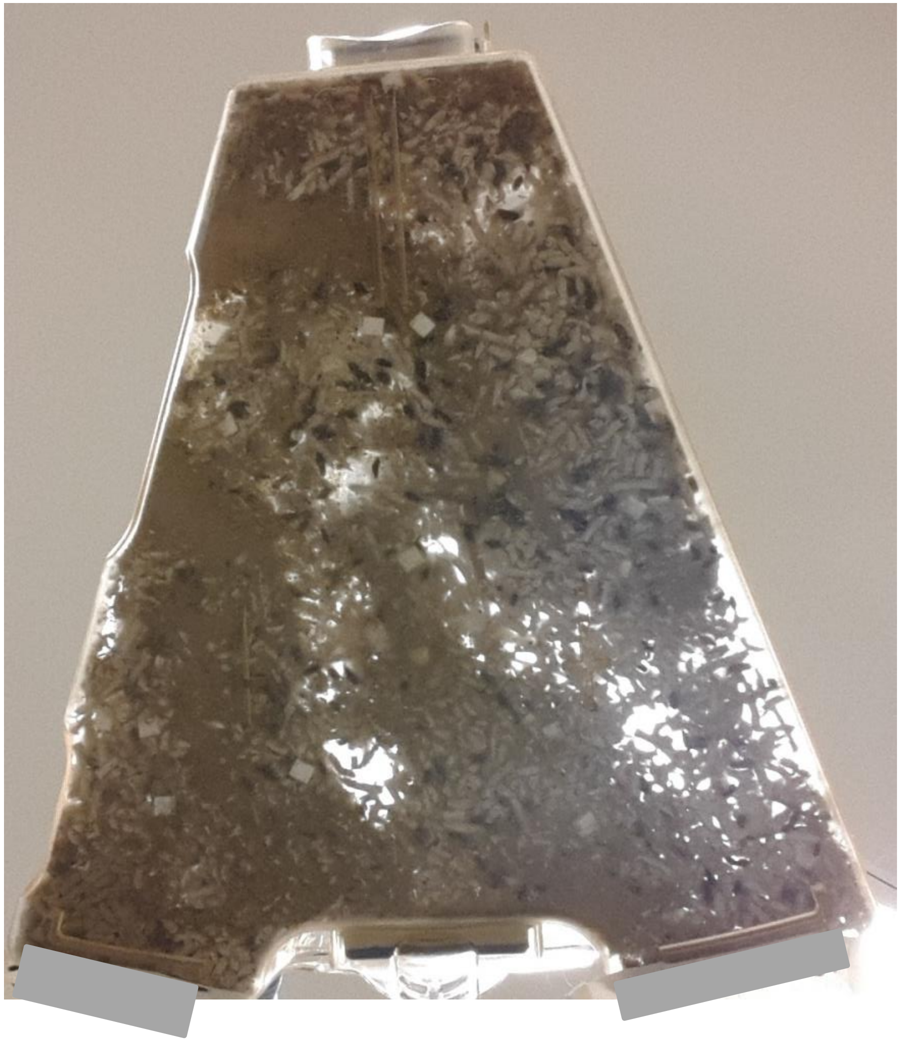

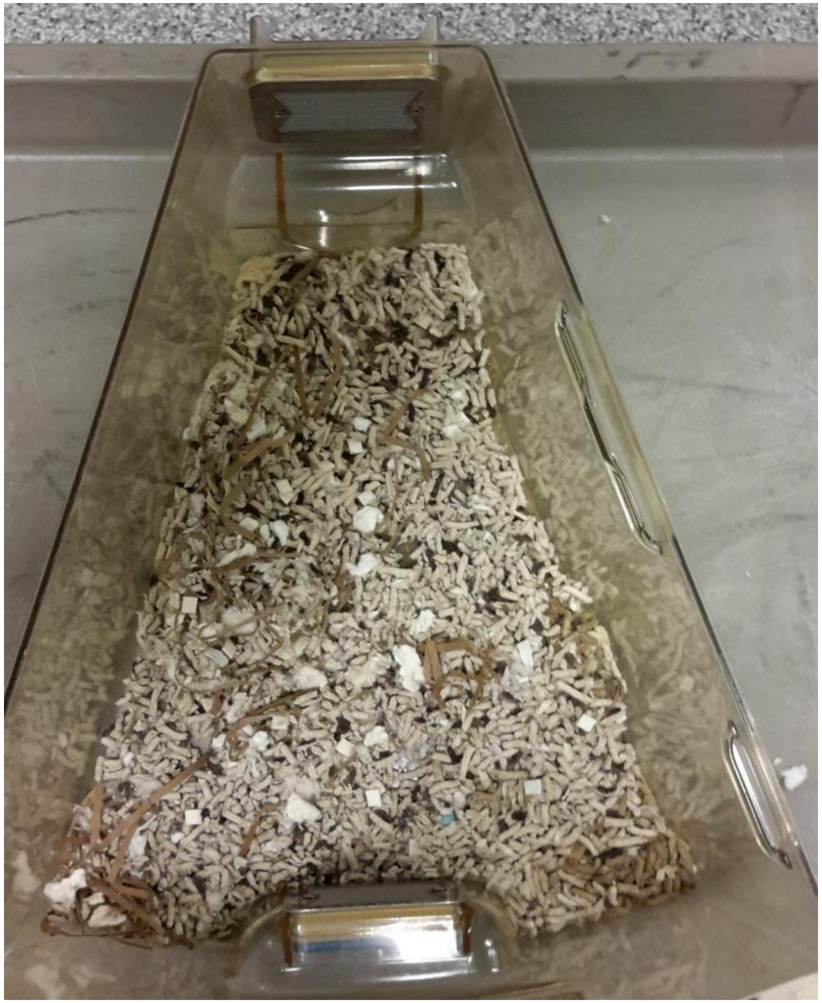

July 22 COMP 3 mid

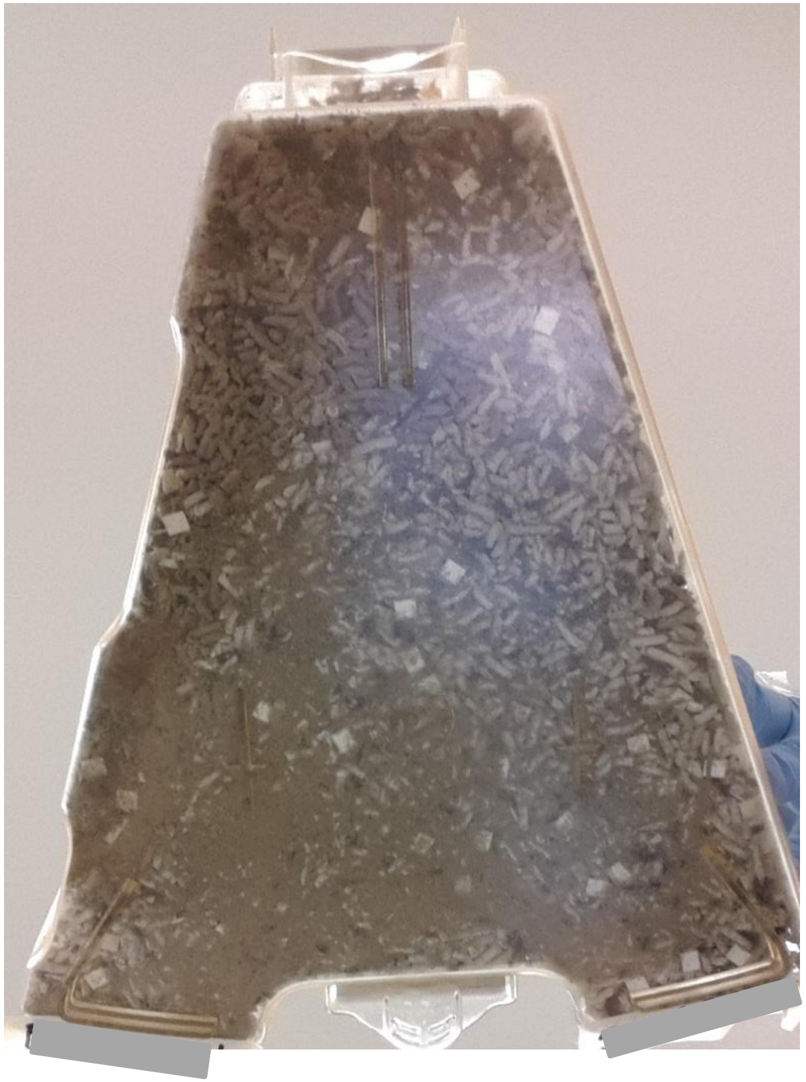

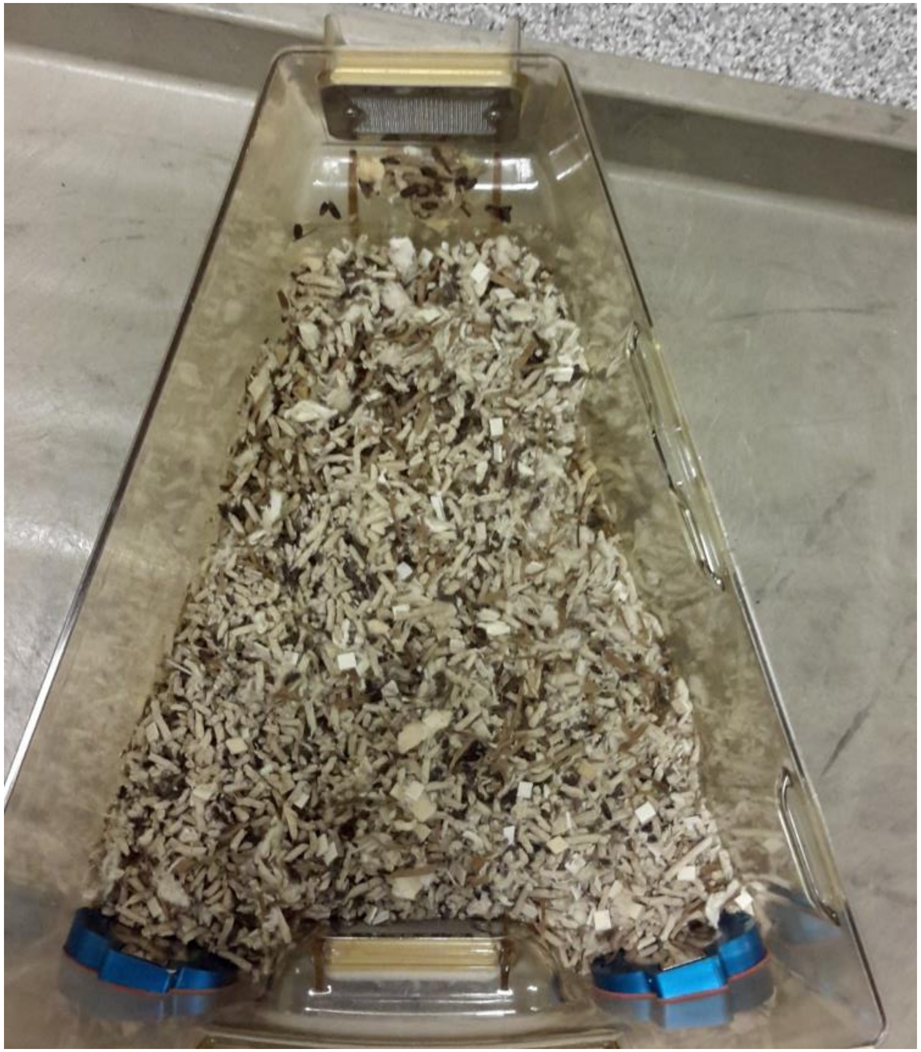

July 22 STD 3

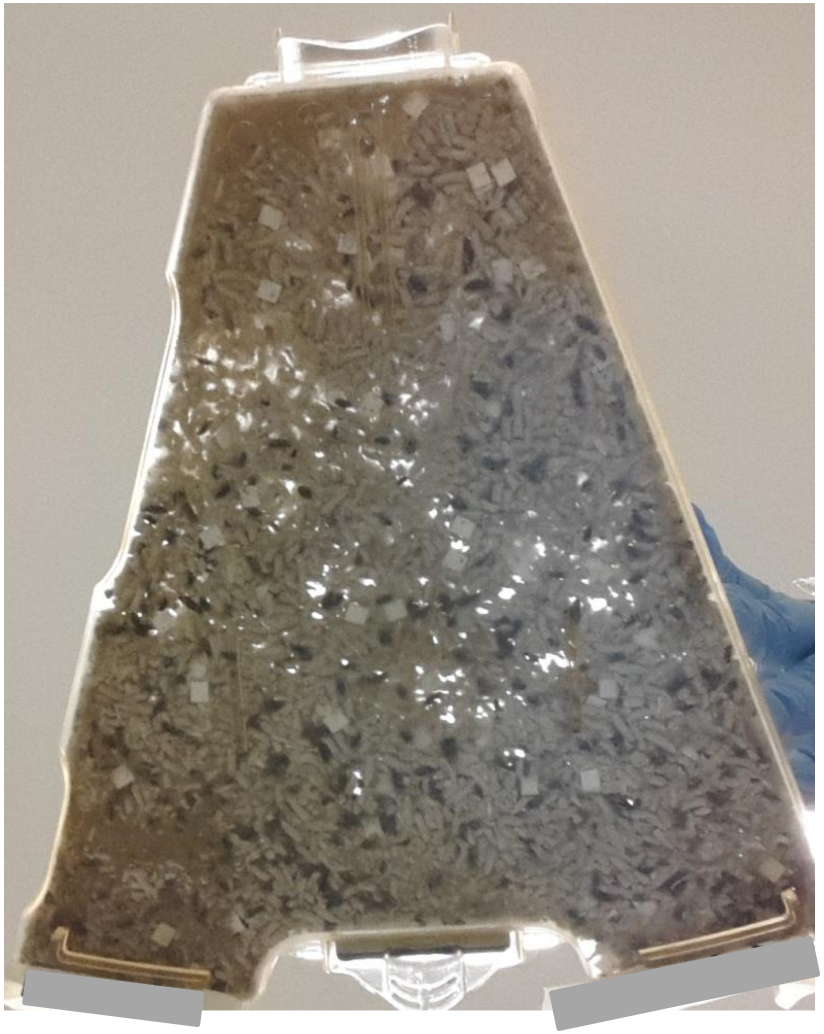

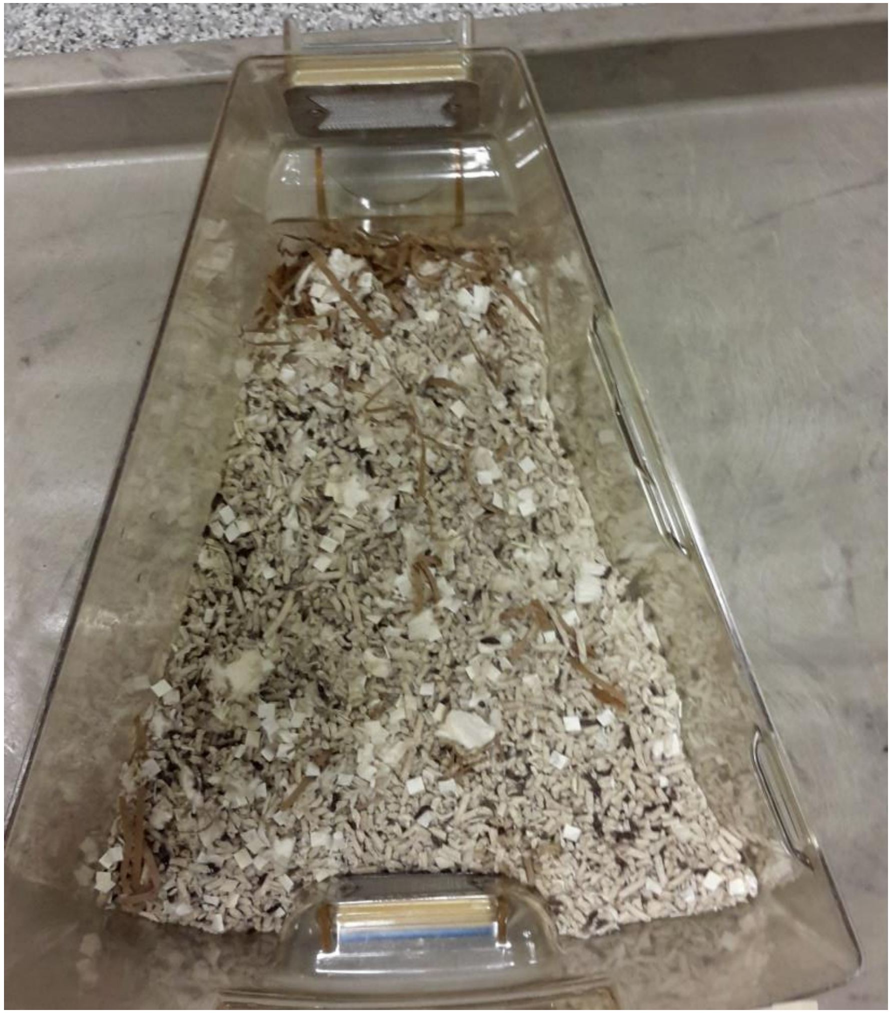

July 22 COMP 4 left

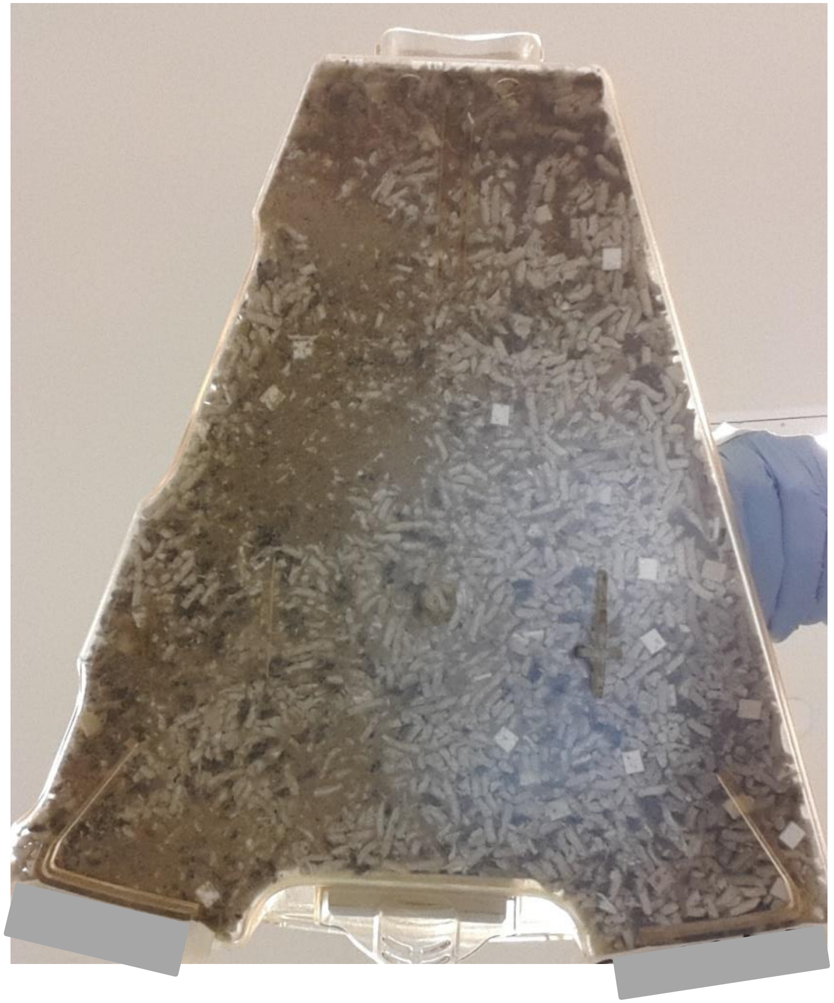

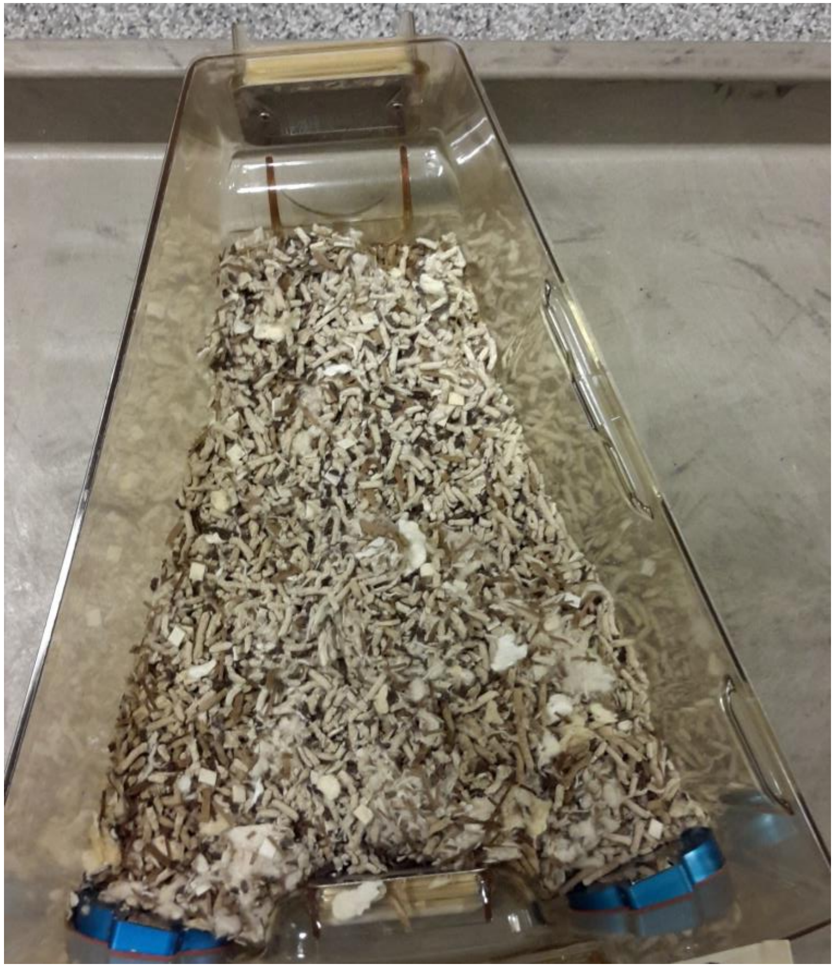

July 22 STD 4

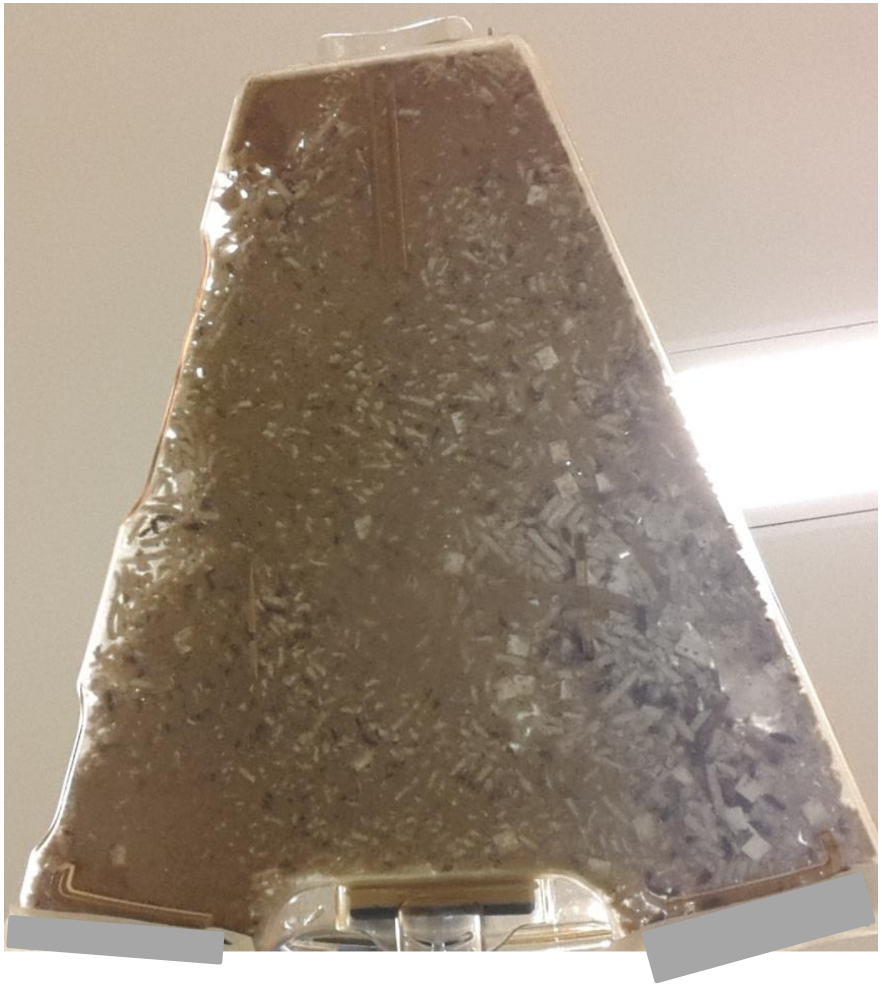

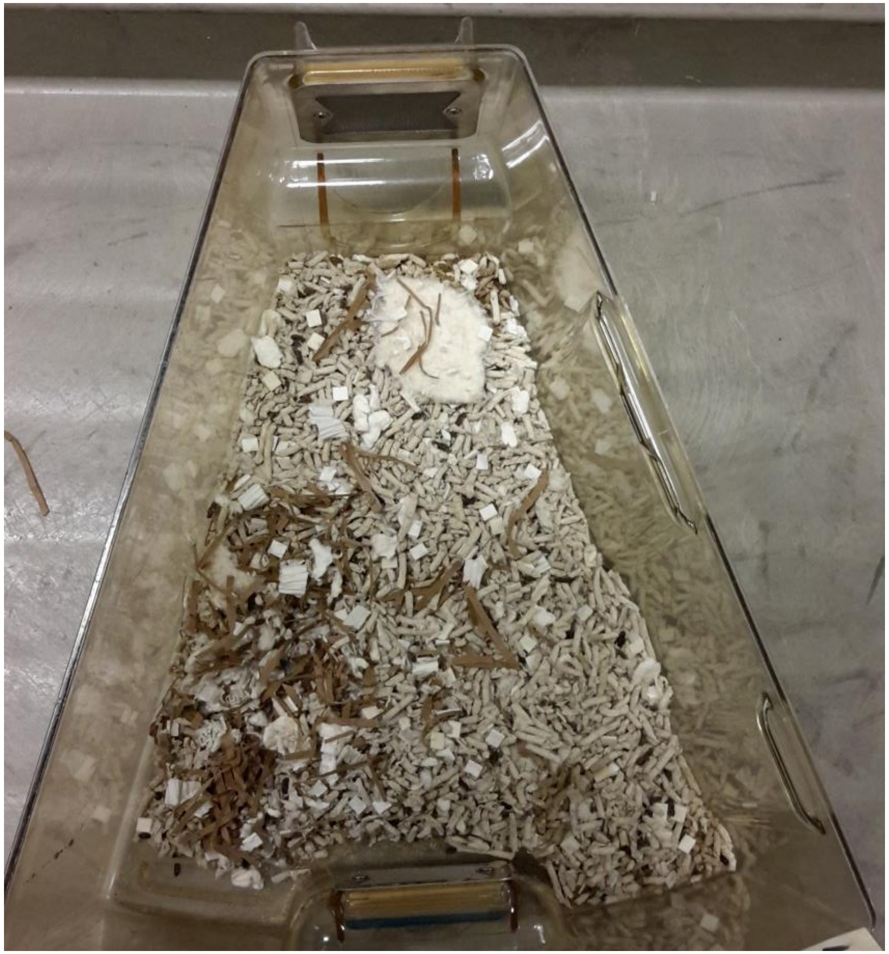

July 23 COMP 5 right

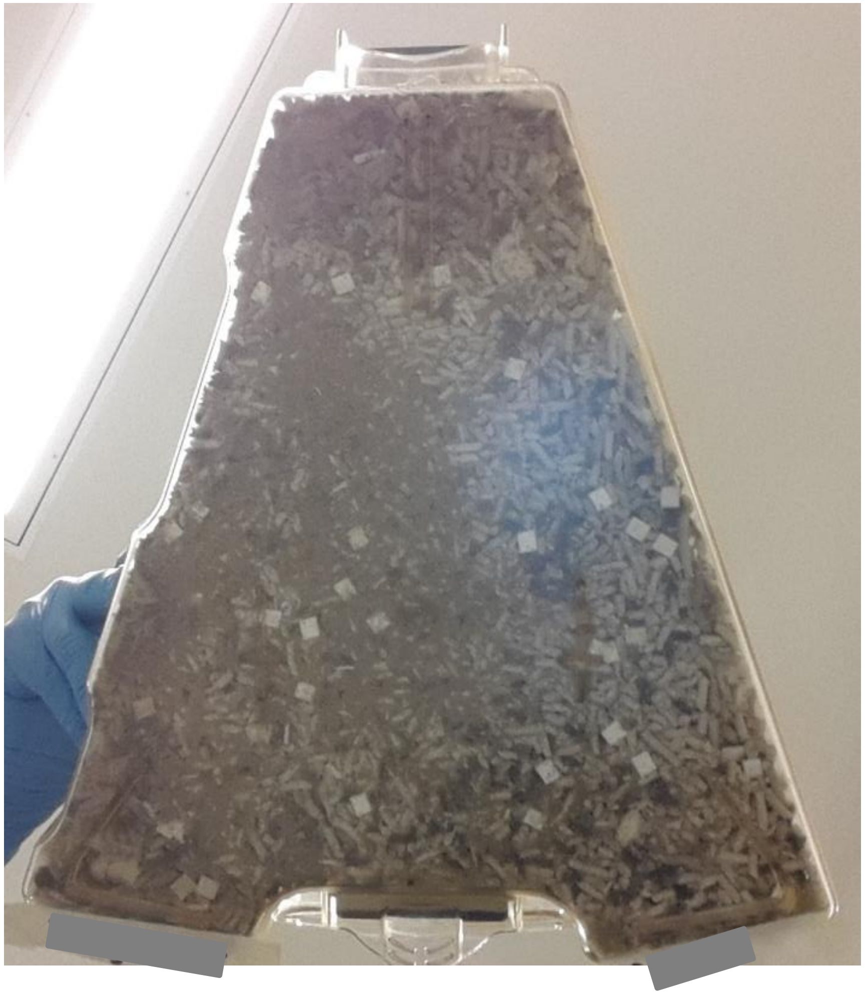

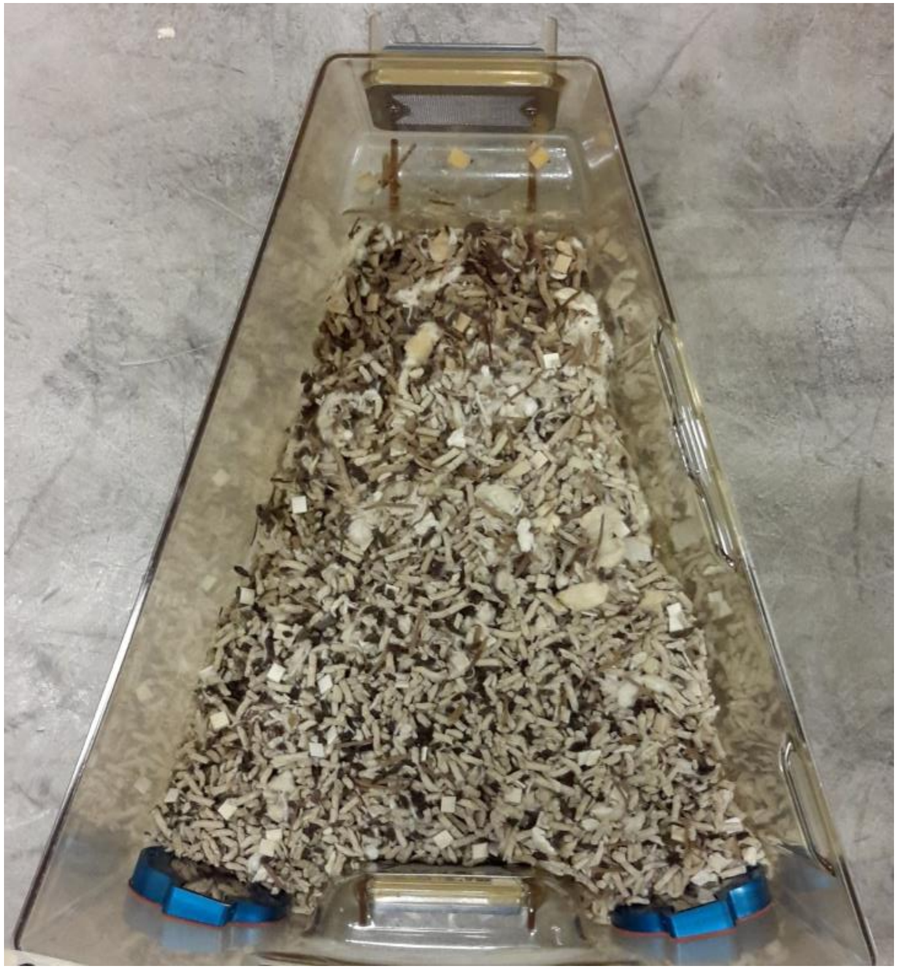

July 23 COMP 5 mid

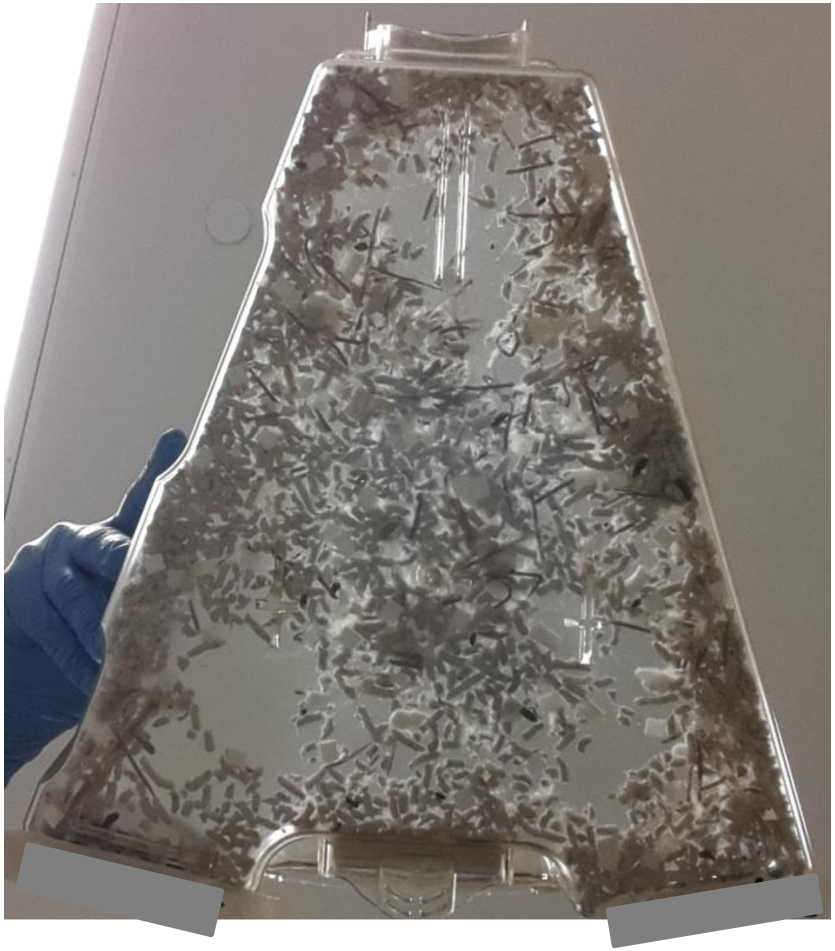

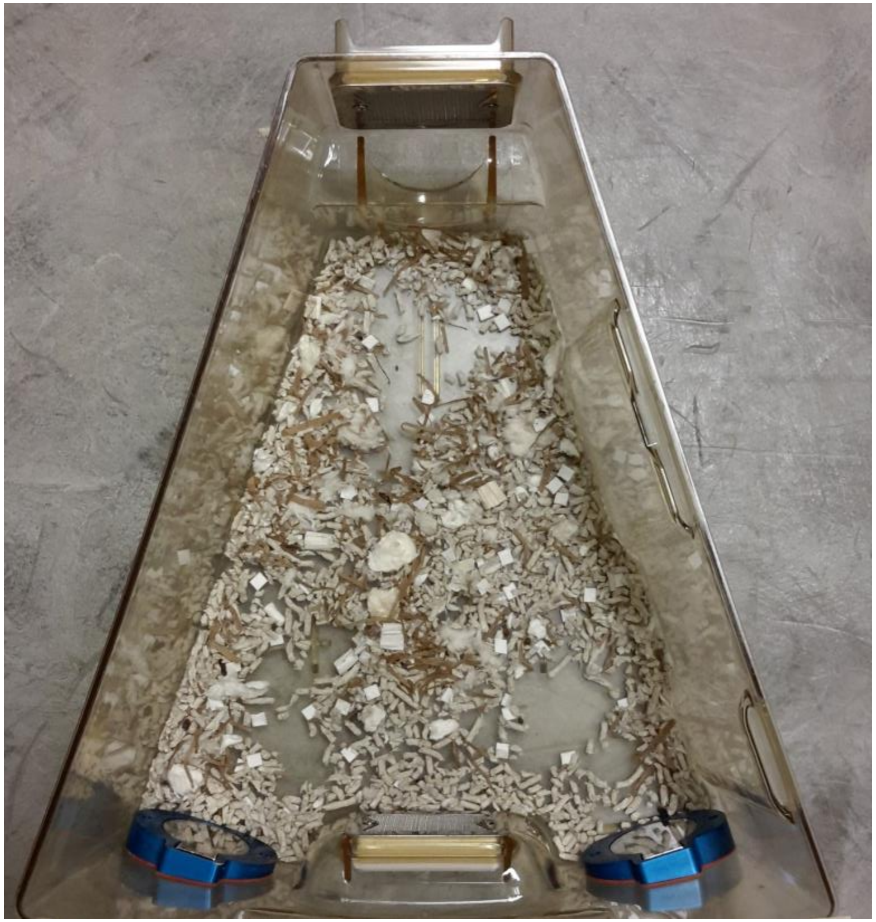

July 23 COMP 5 left

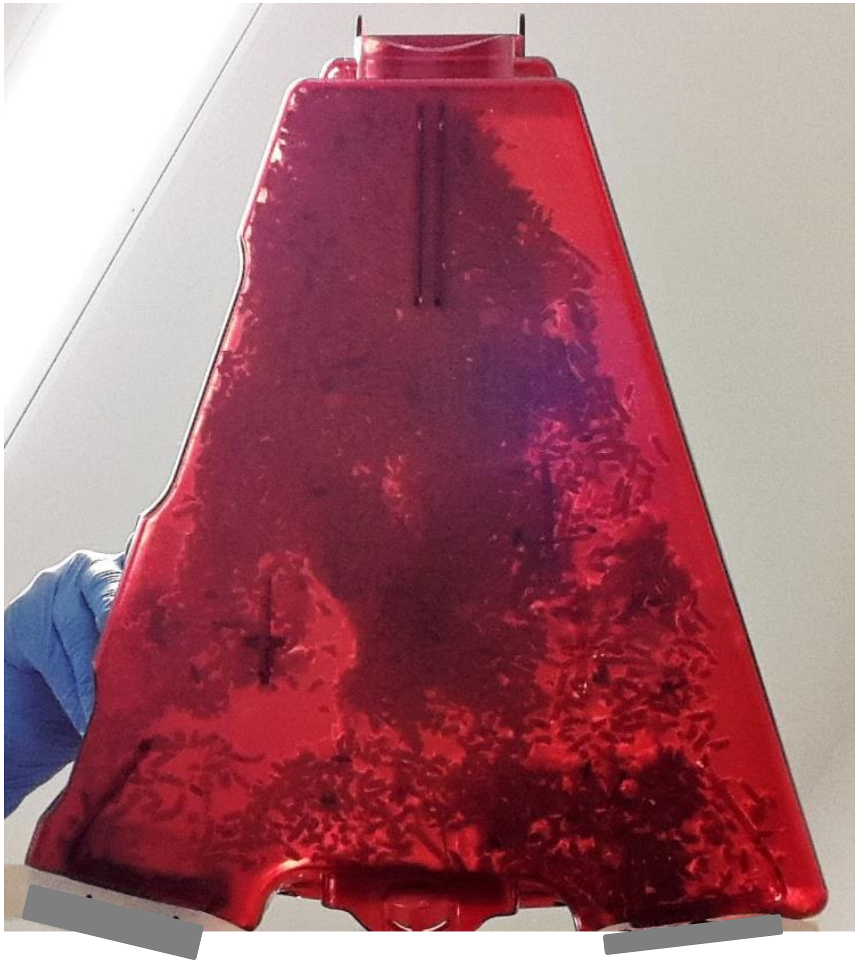

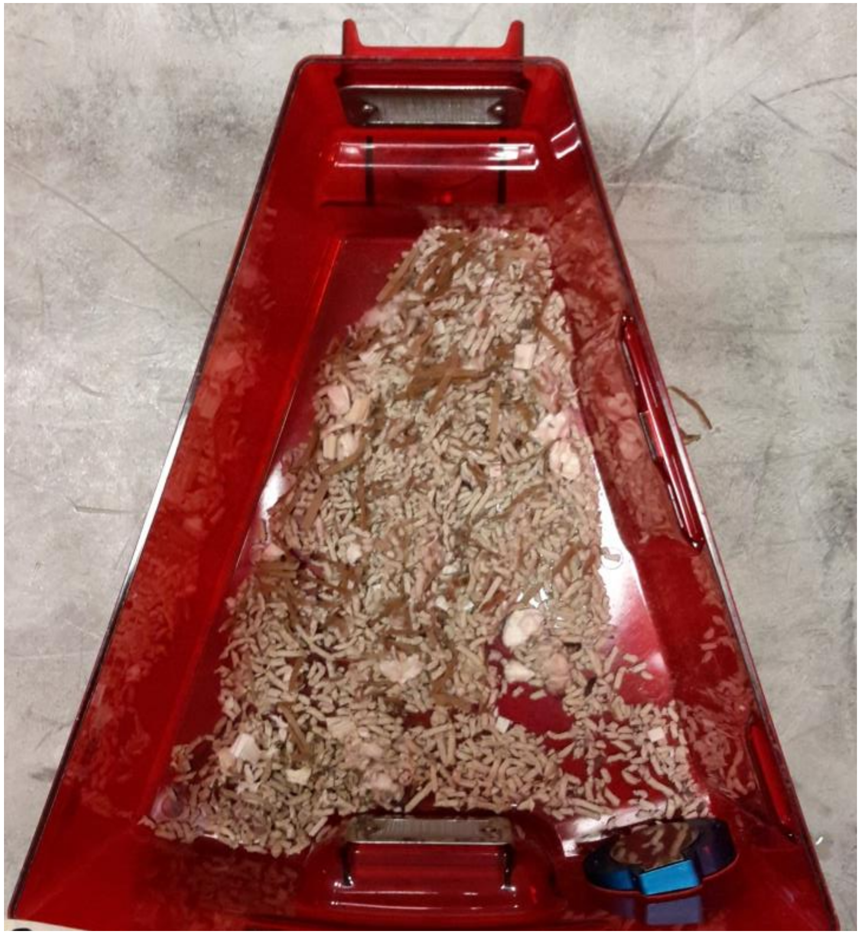

July 23 STD 5

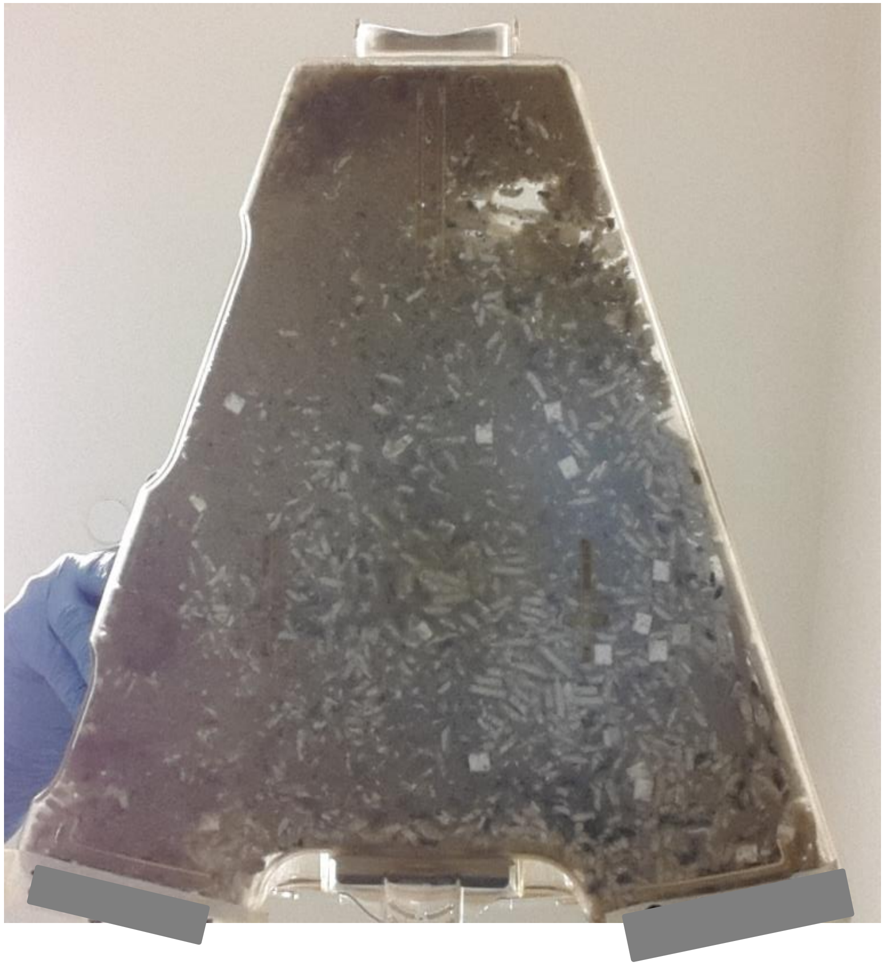

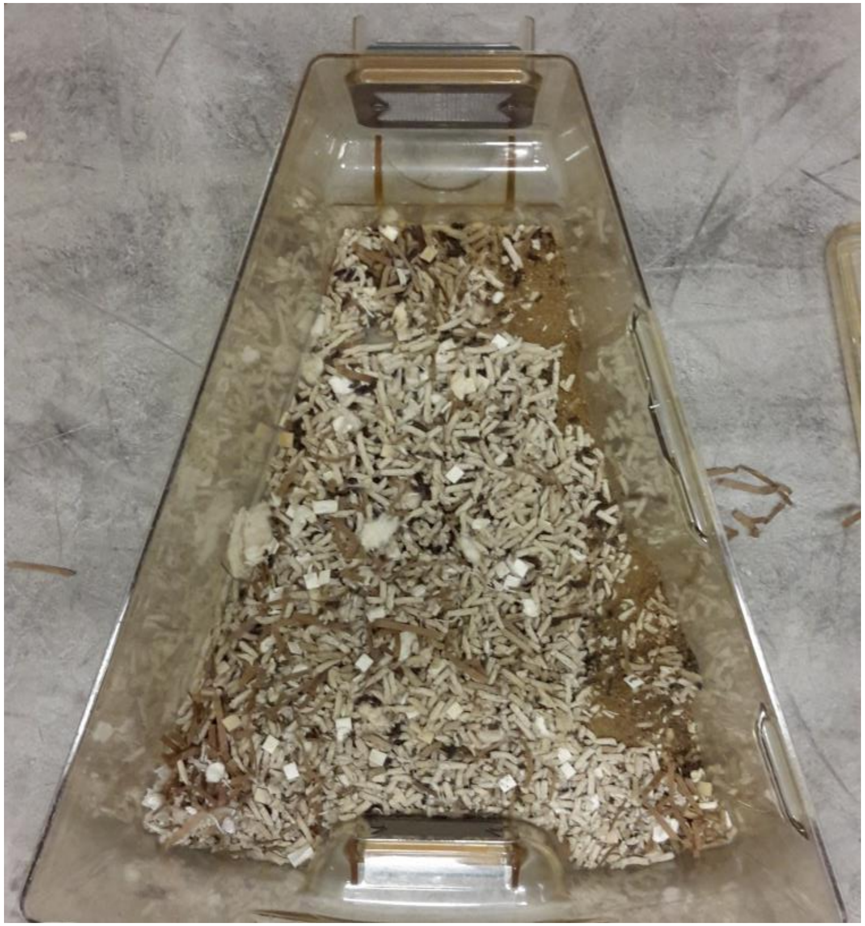

July 28 COMP 1 right

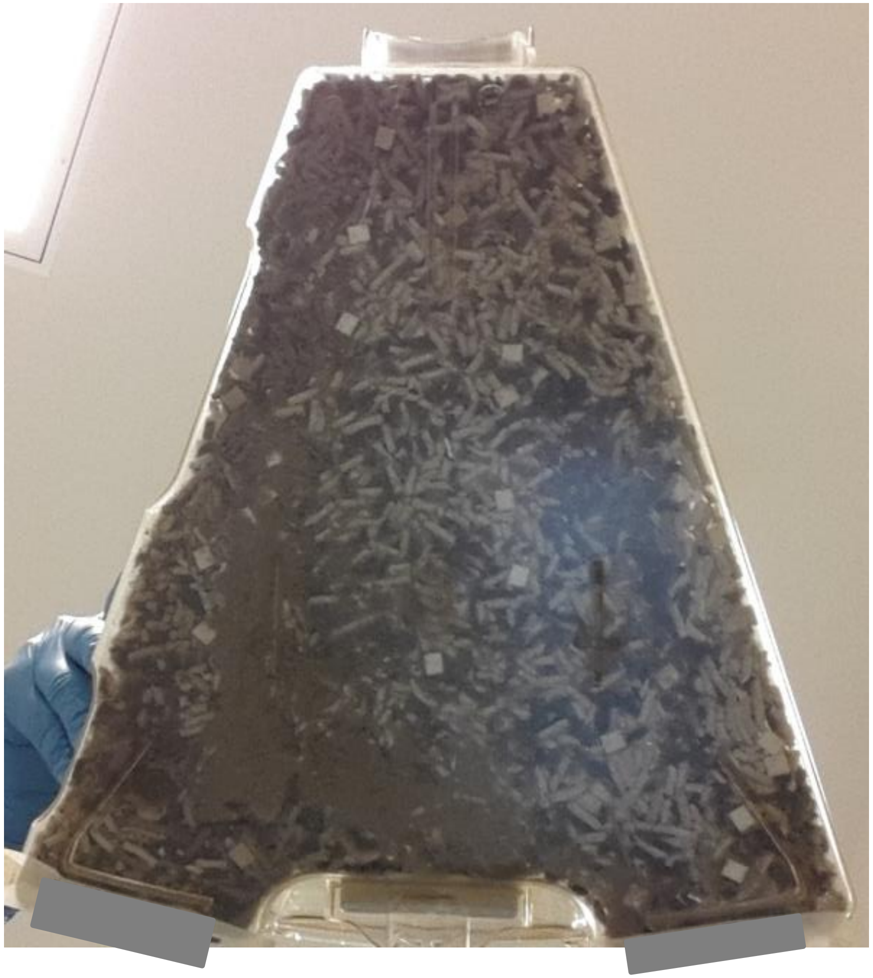
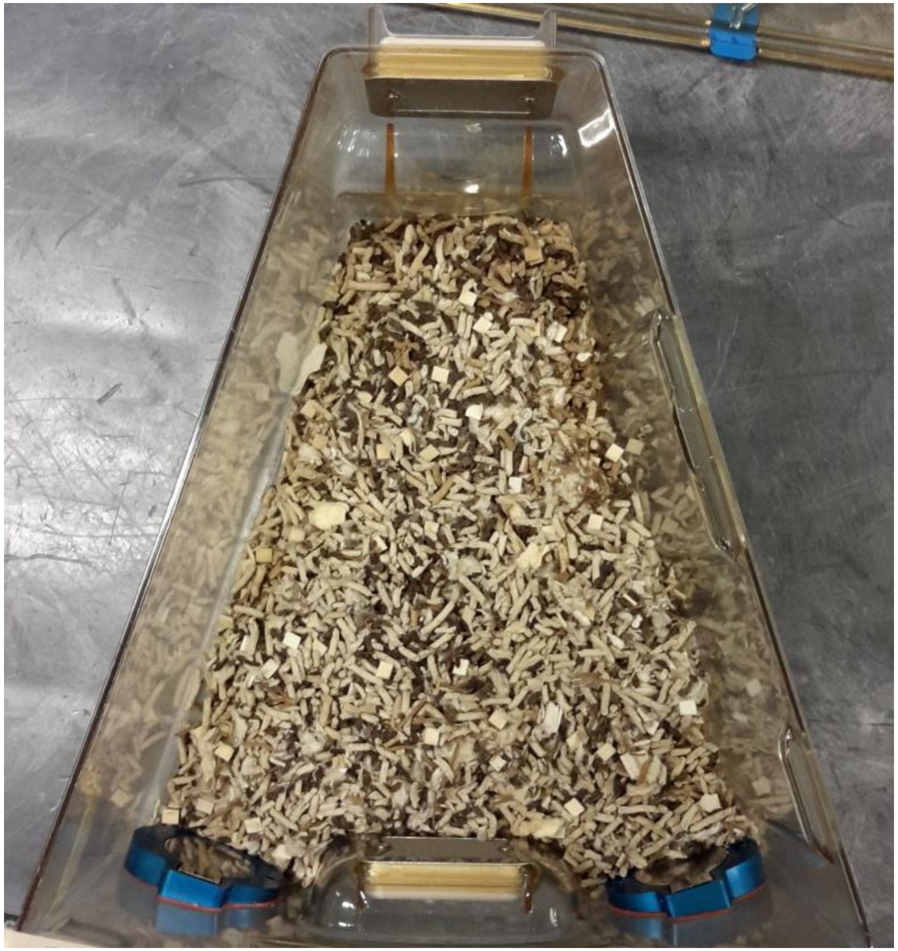

July 28 COMP 1 mid

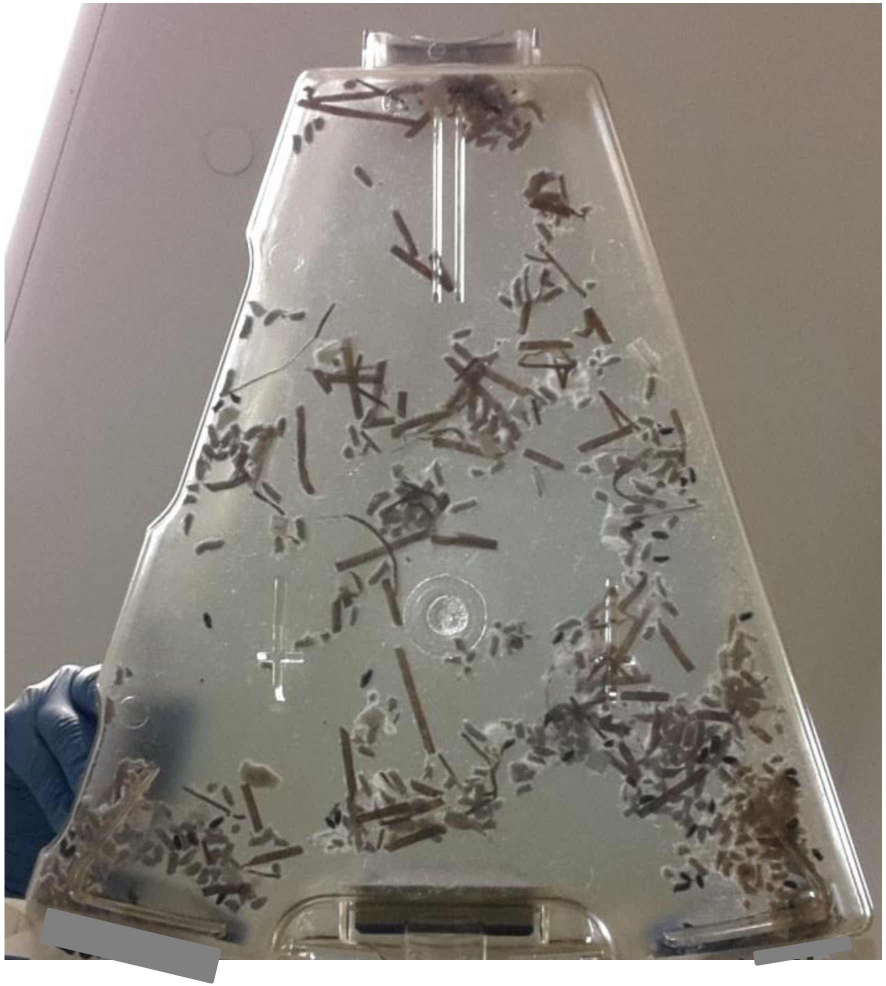

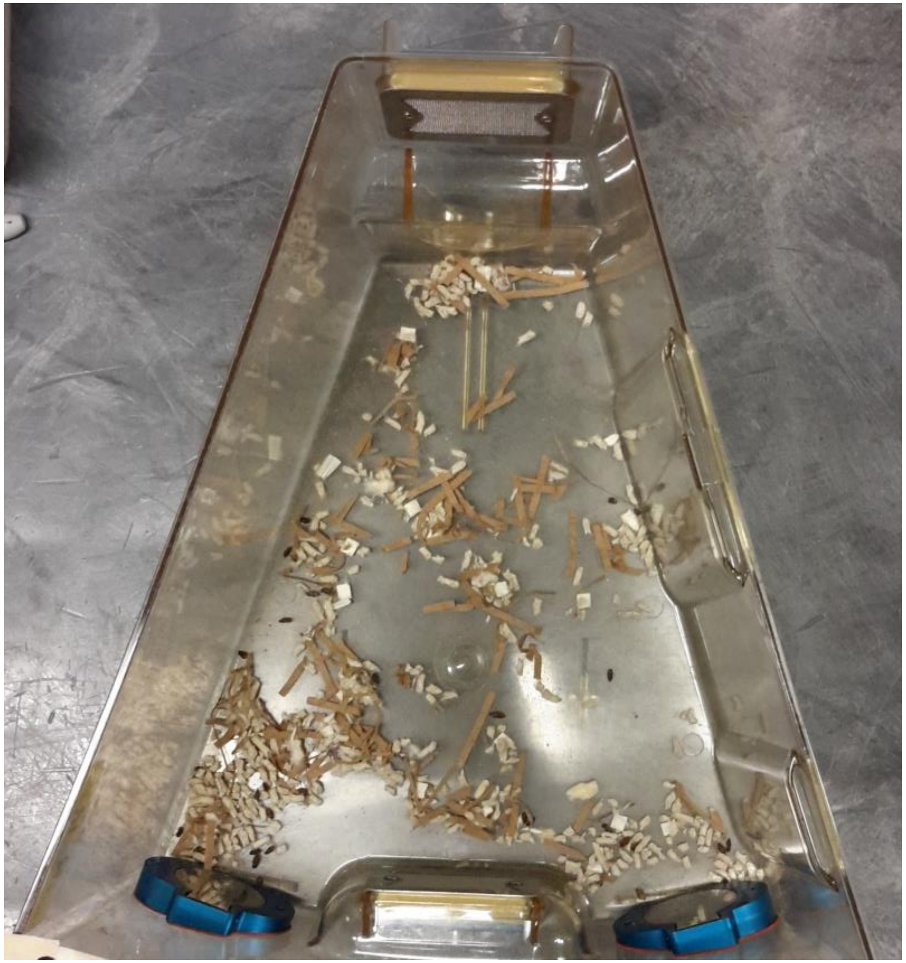

July 28 COMP 1 left

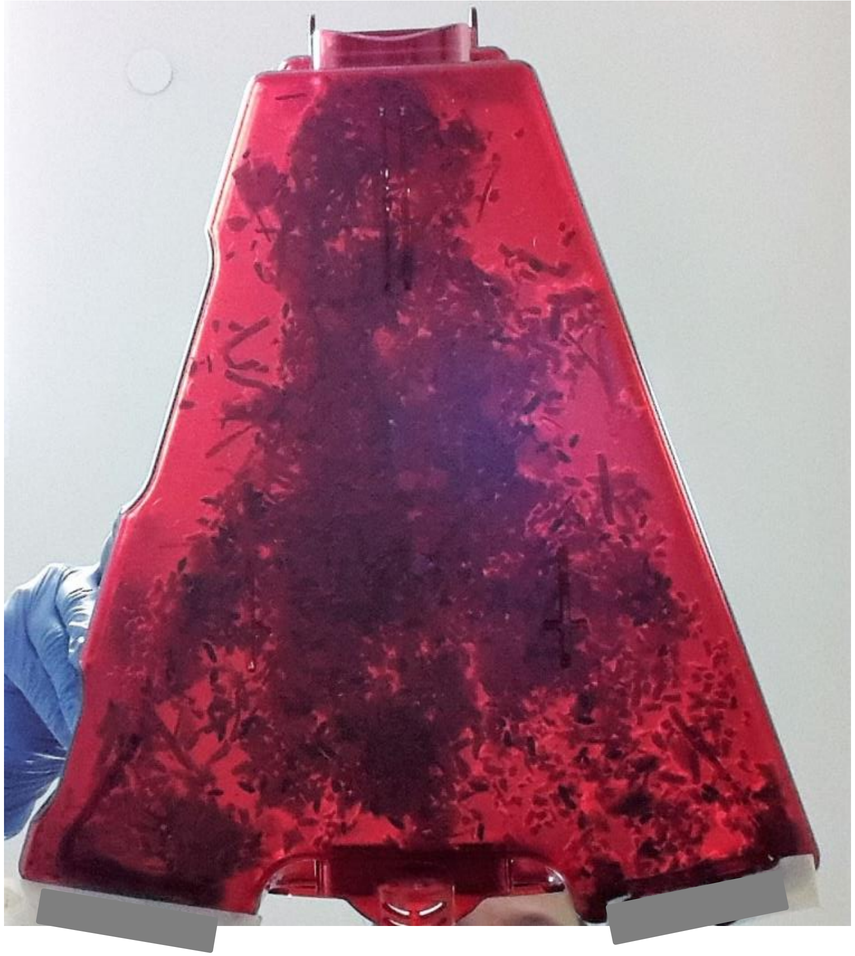

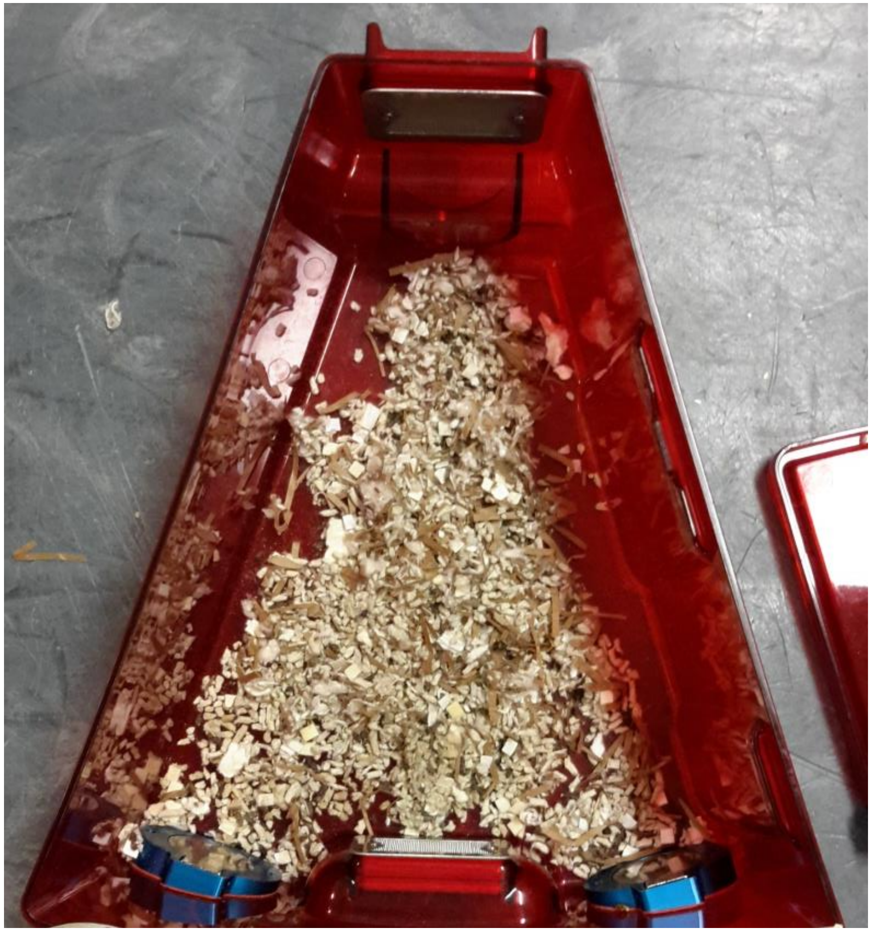

July 28 STD 1

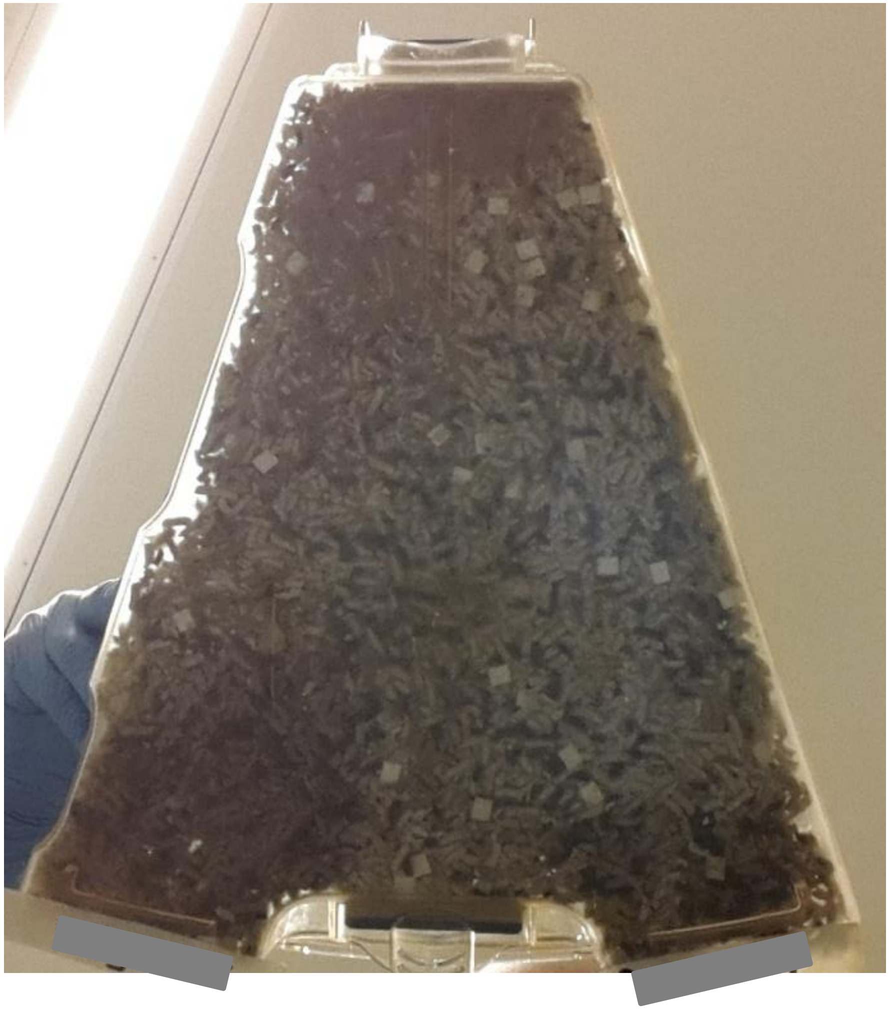

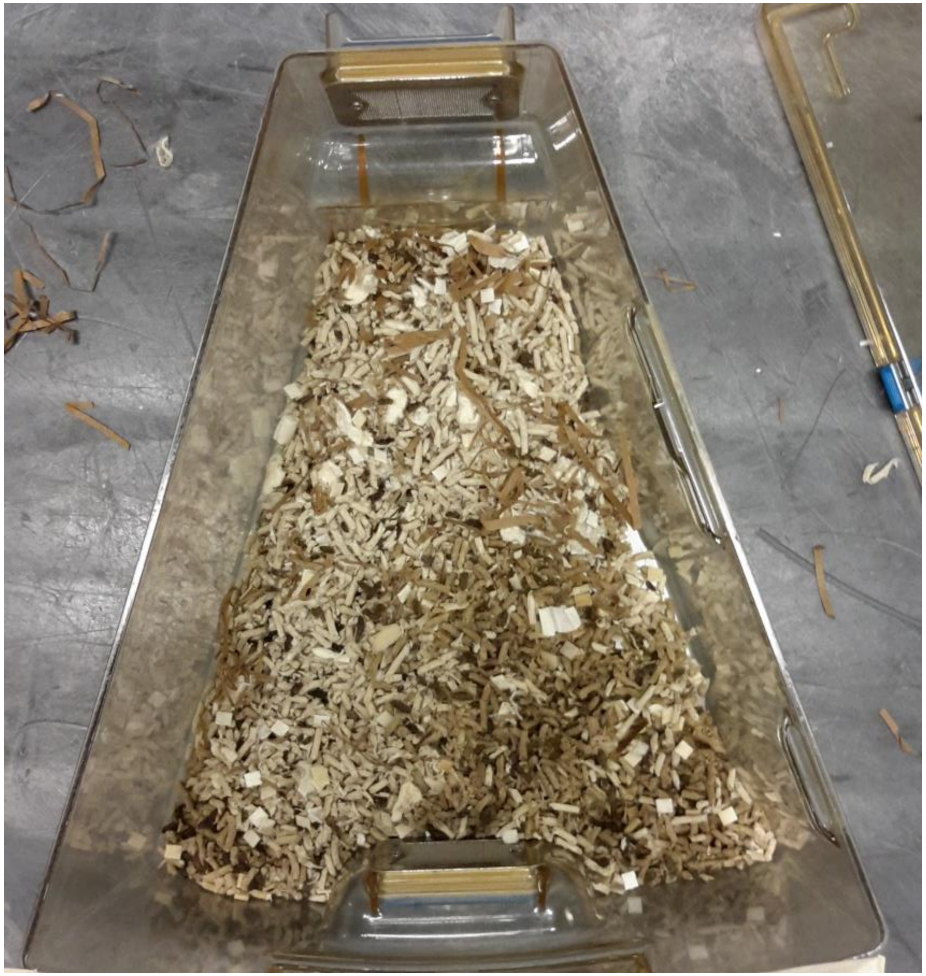

July 28 COMP 2 mid

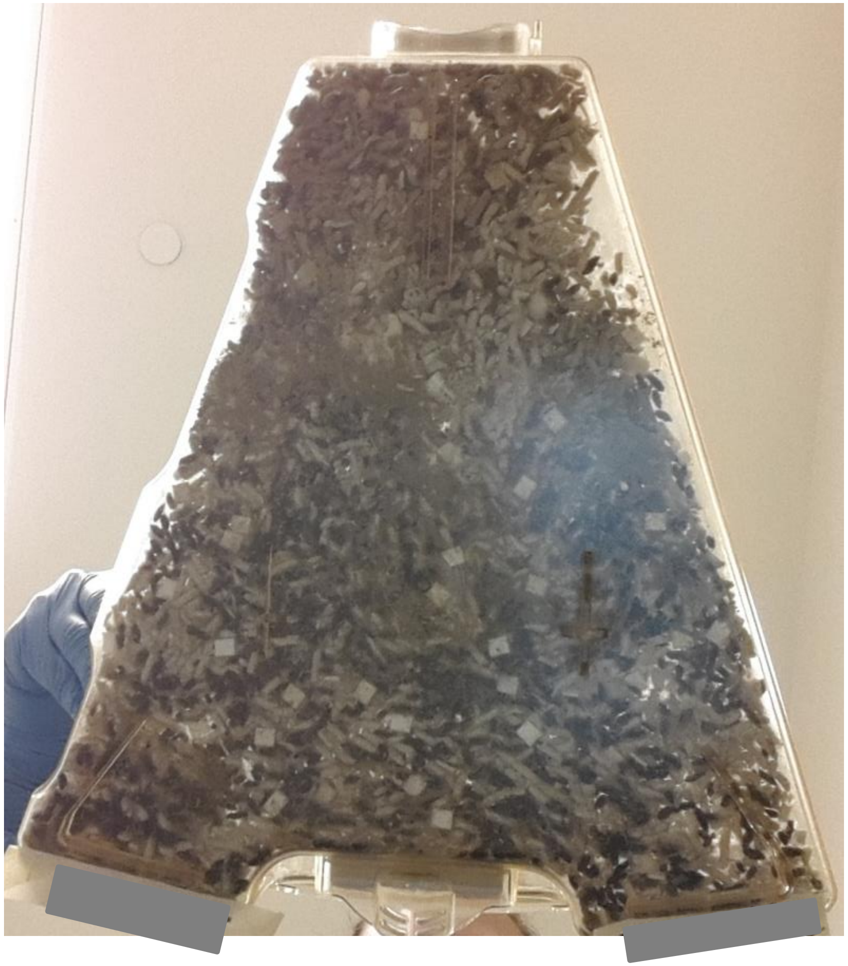

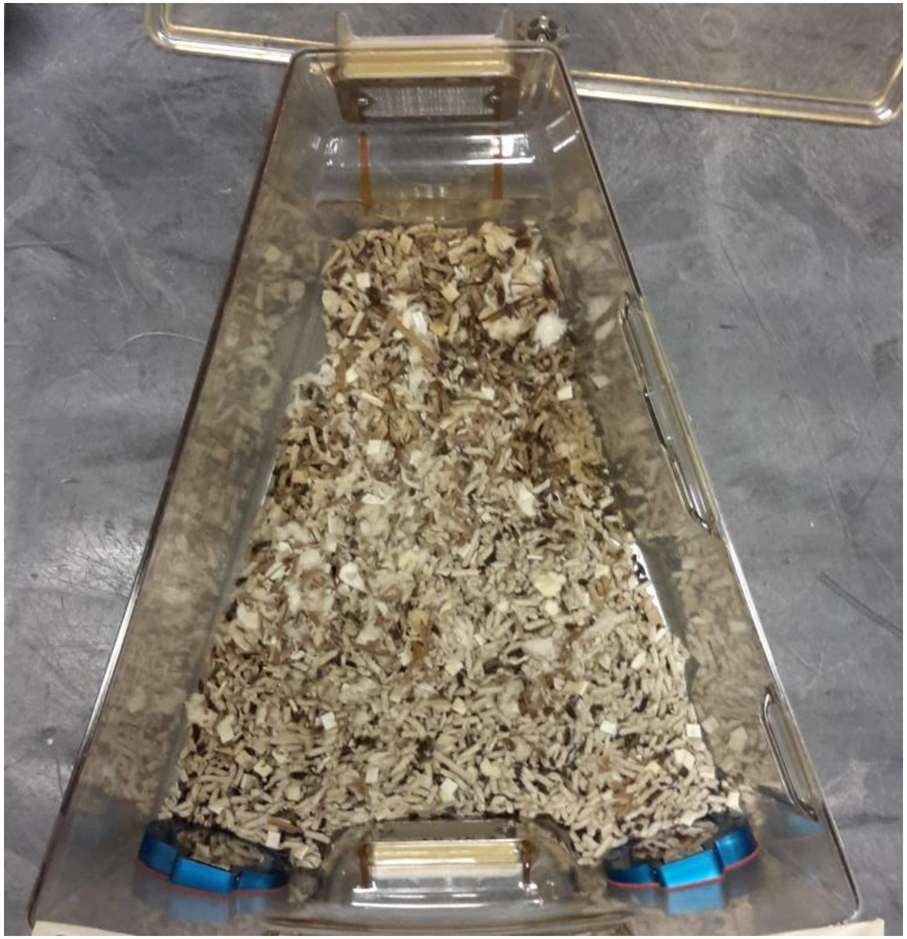

July 28 COMP 2 left

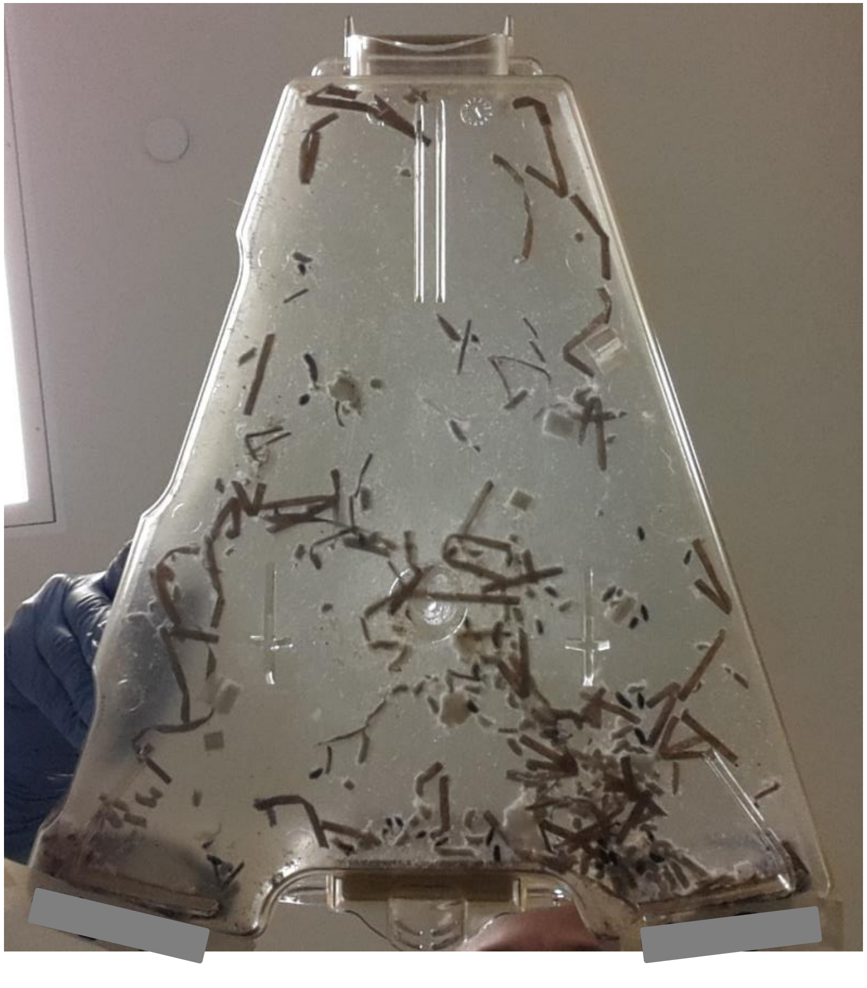

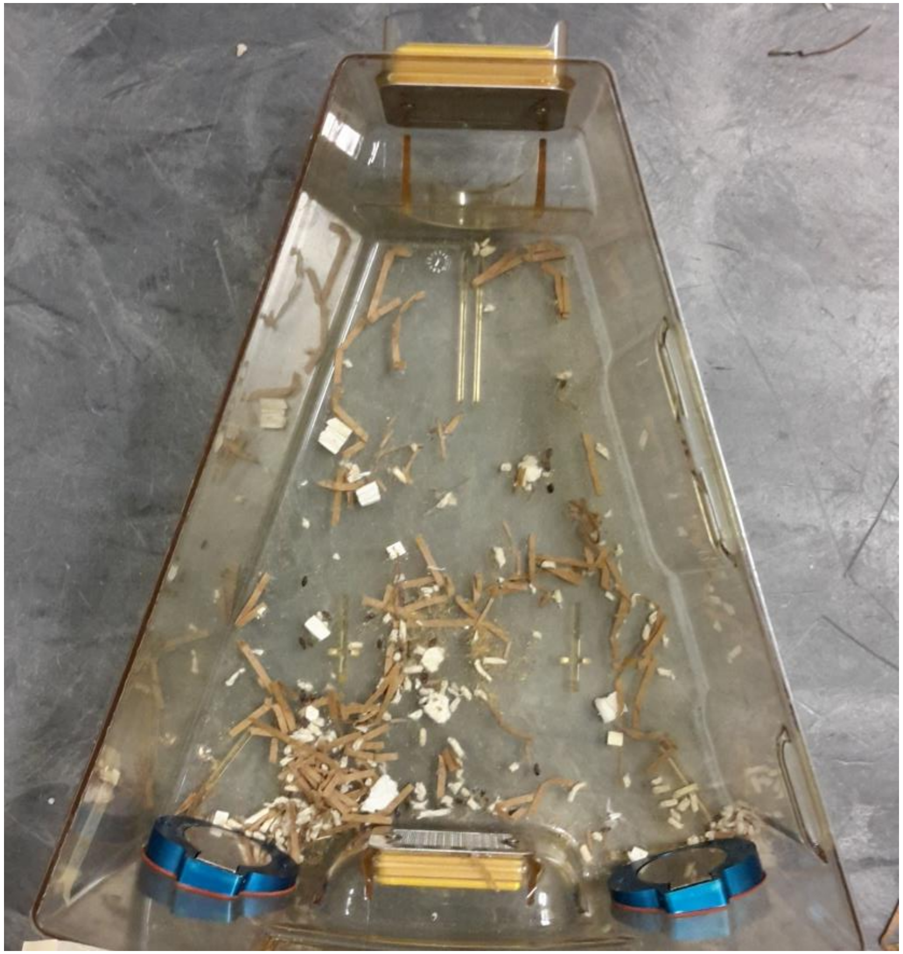

July 28 COMP 2 right

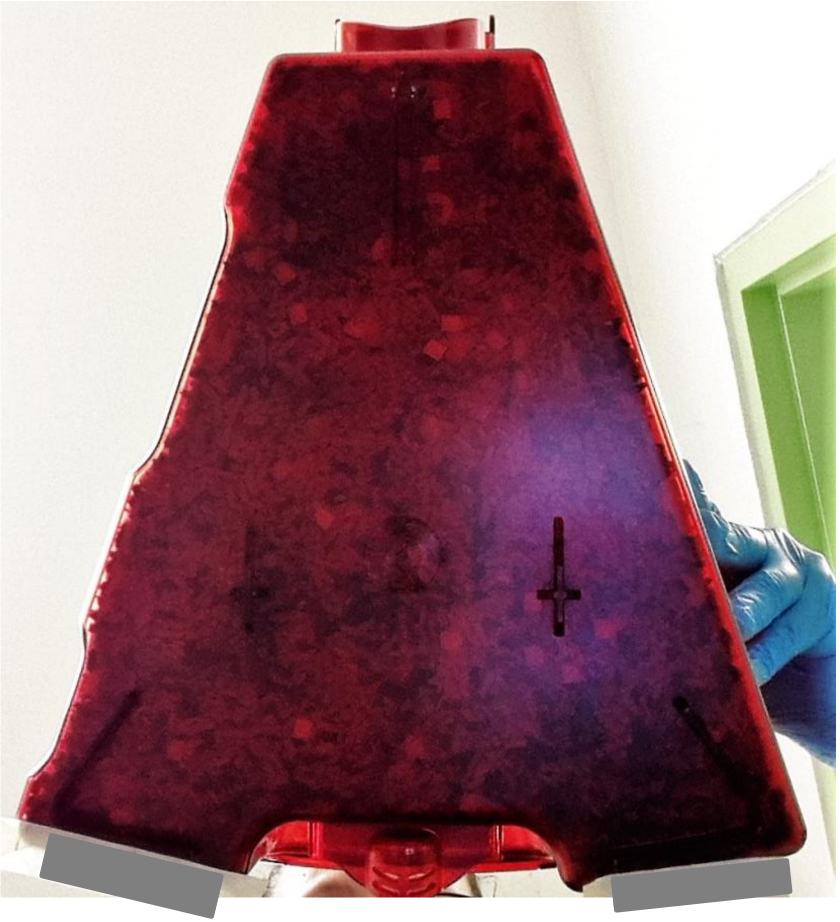

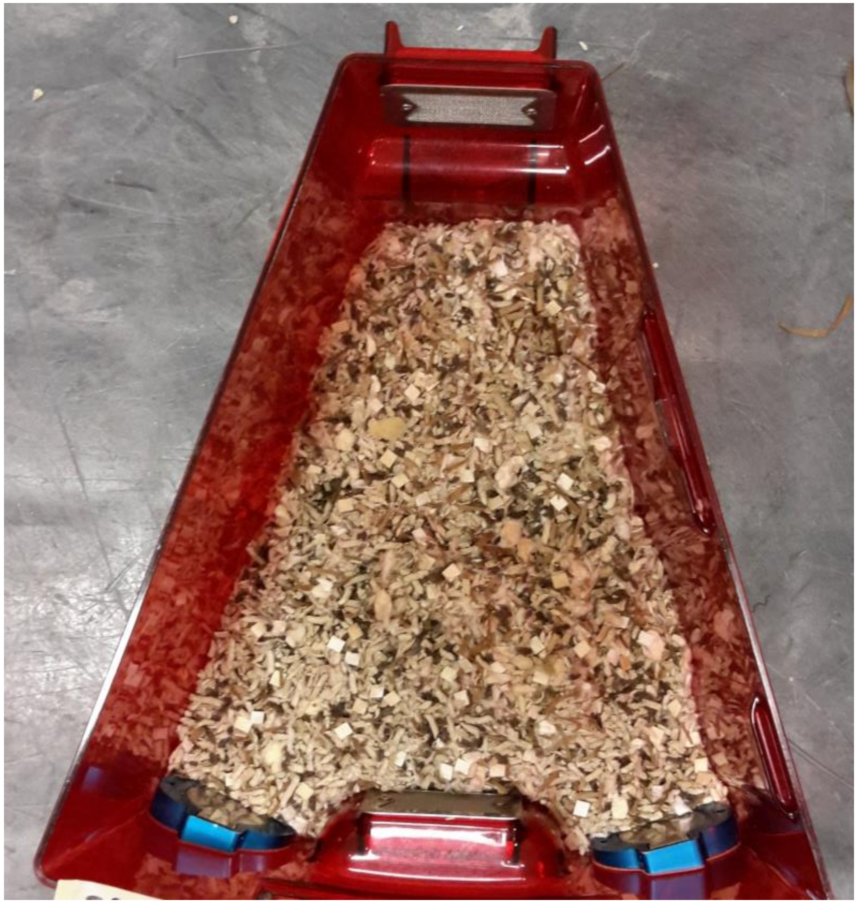

July 28 STD 2

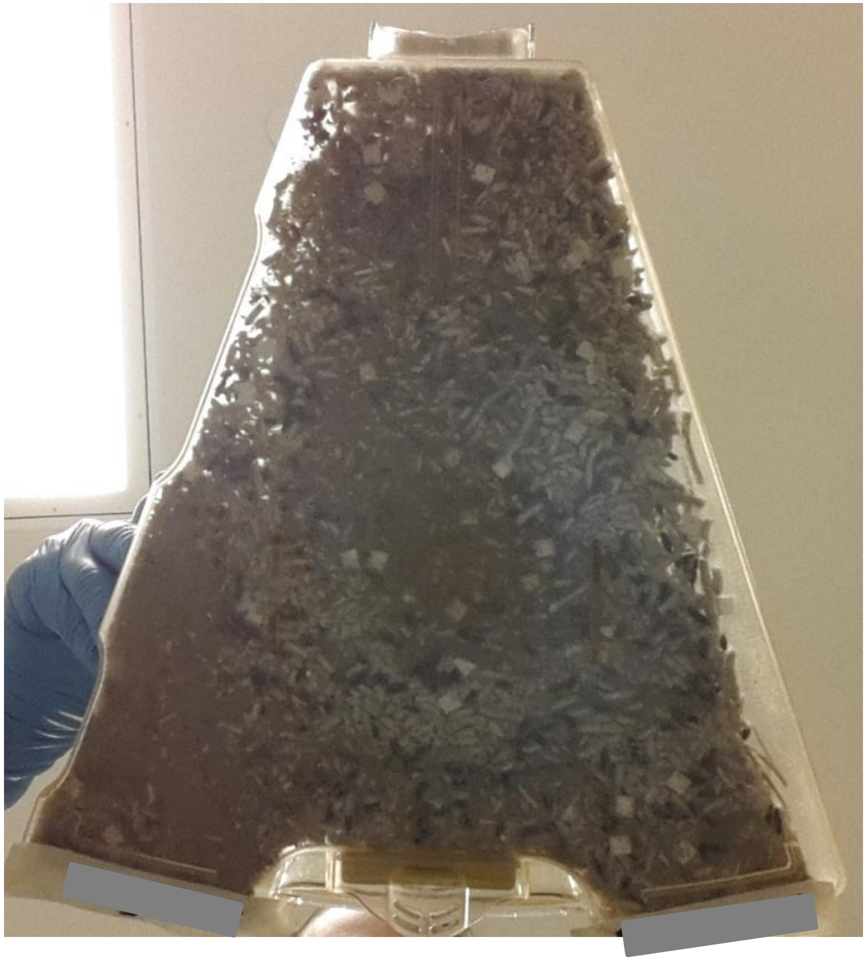

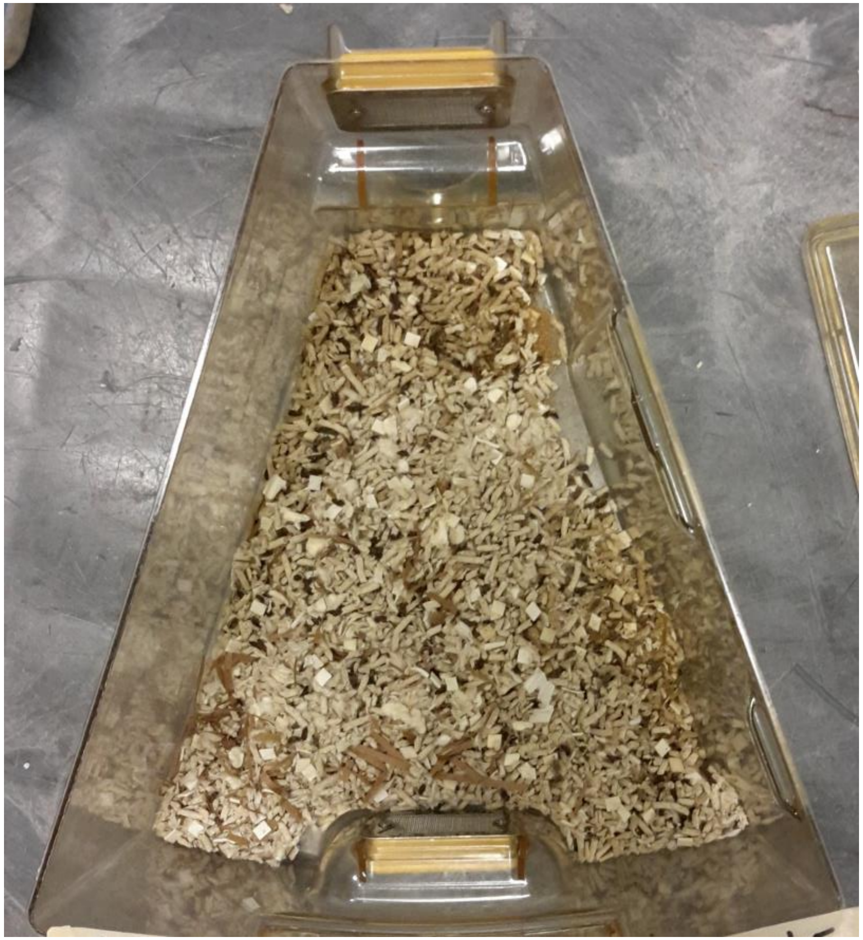

July 29 COMP 3 left

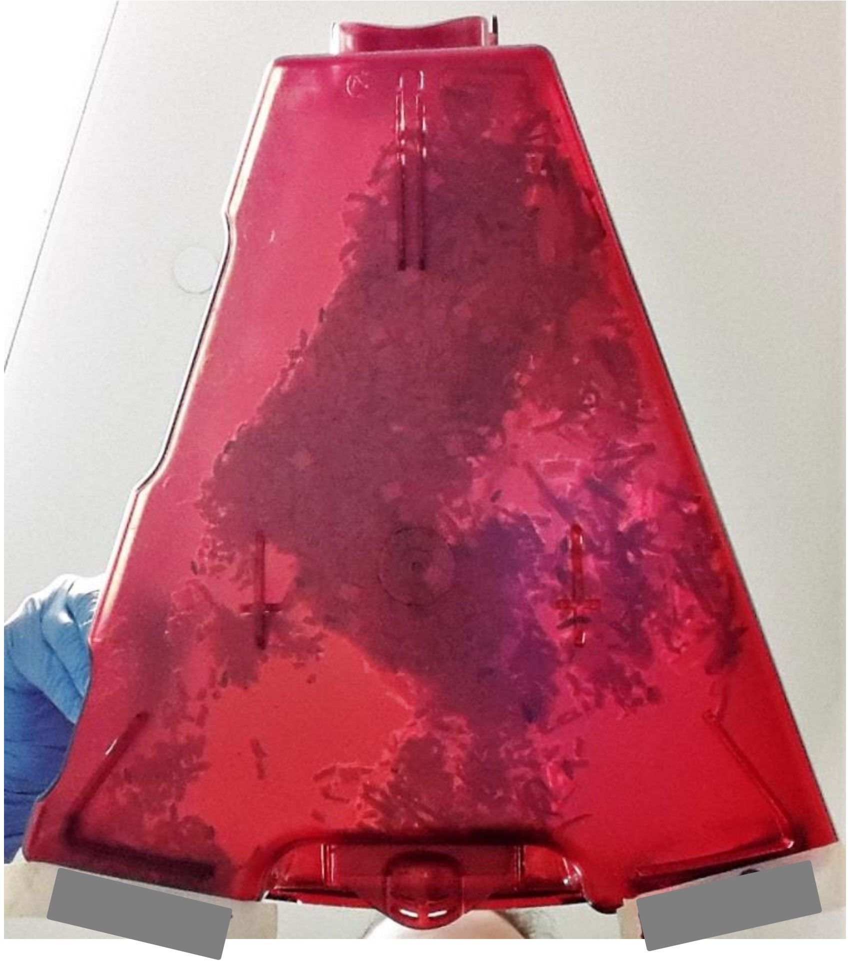

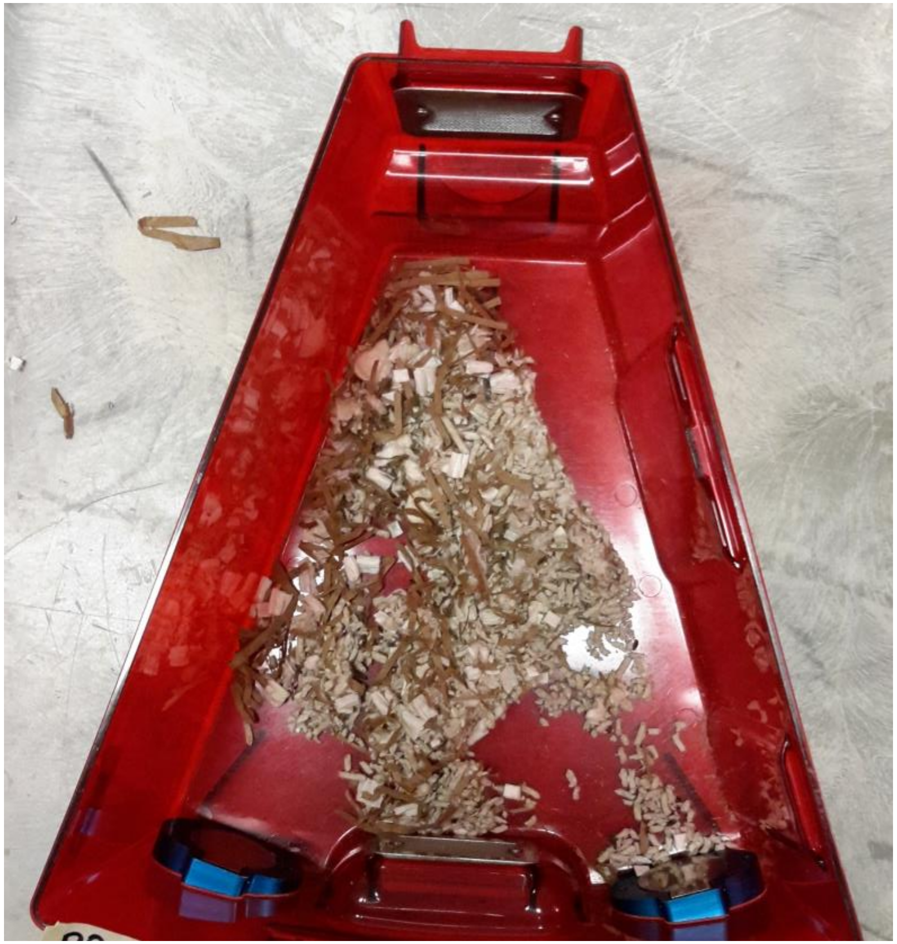

July 29 COMP 3 mid

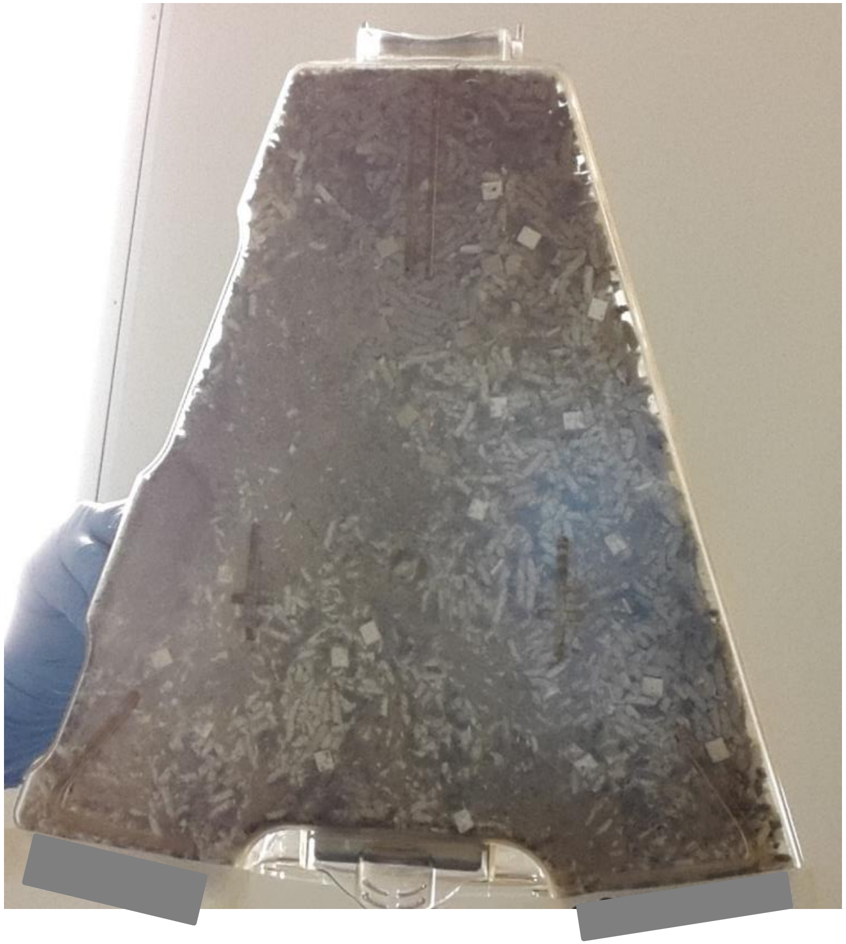

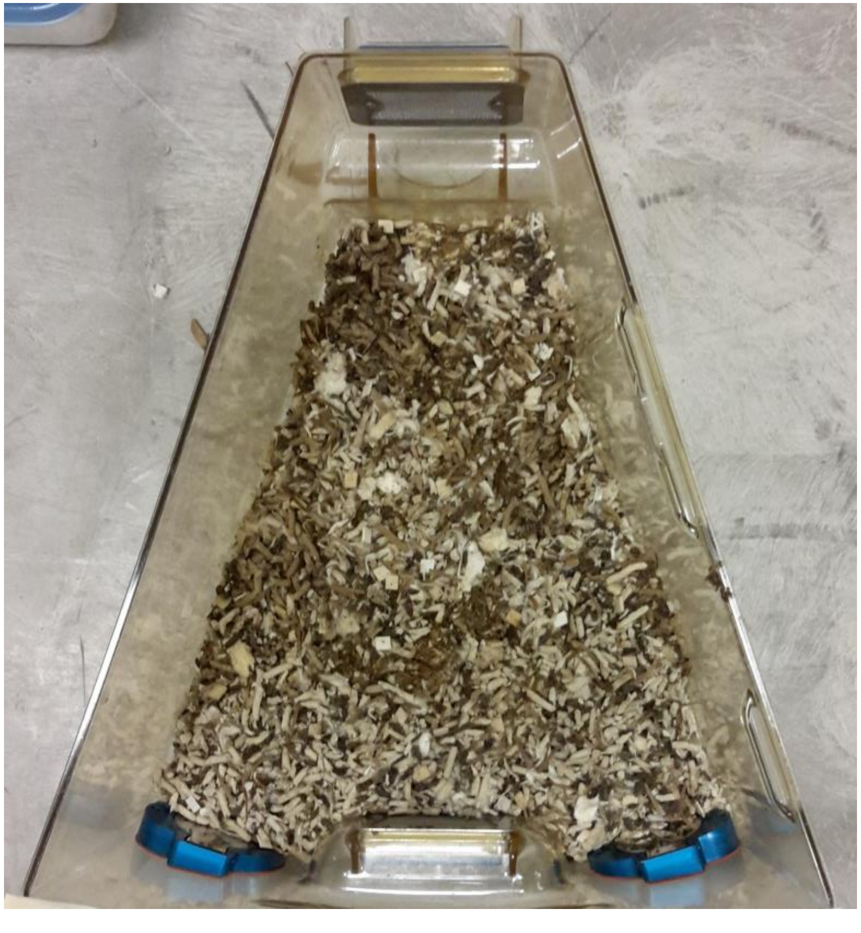

July 29 COMP 3 right

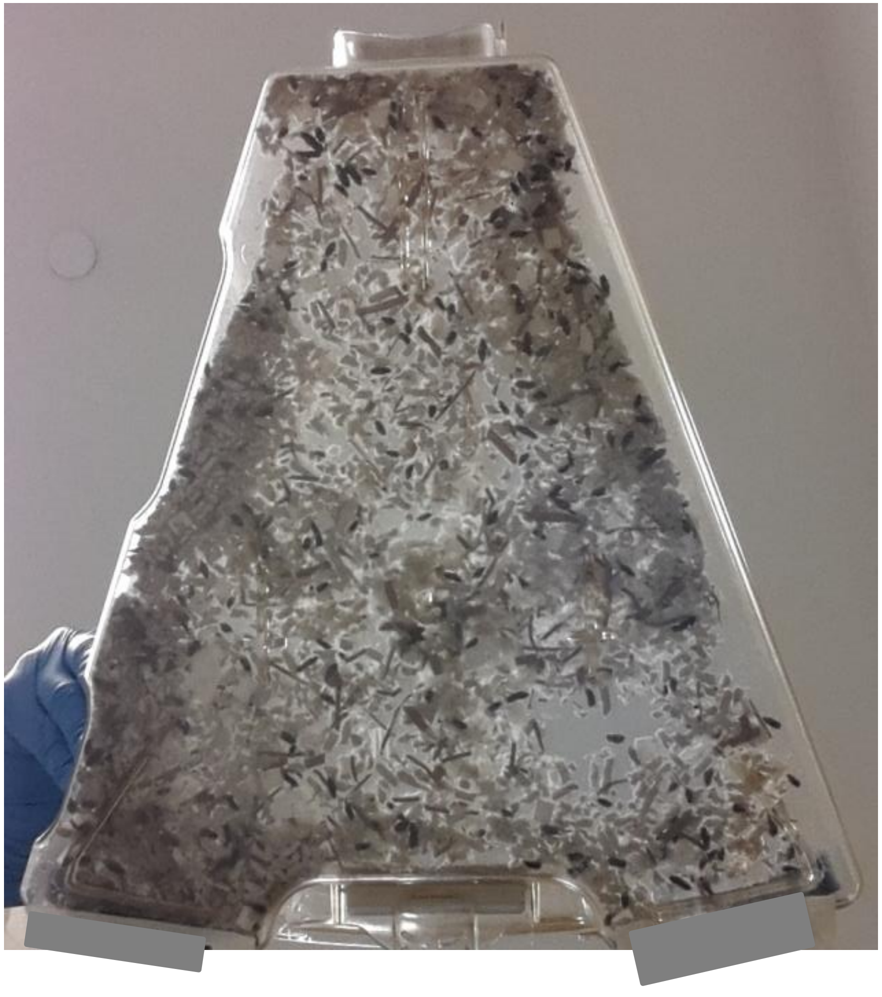

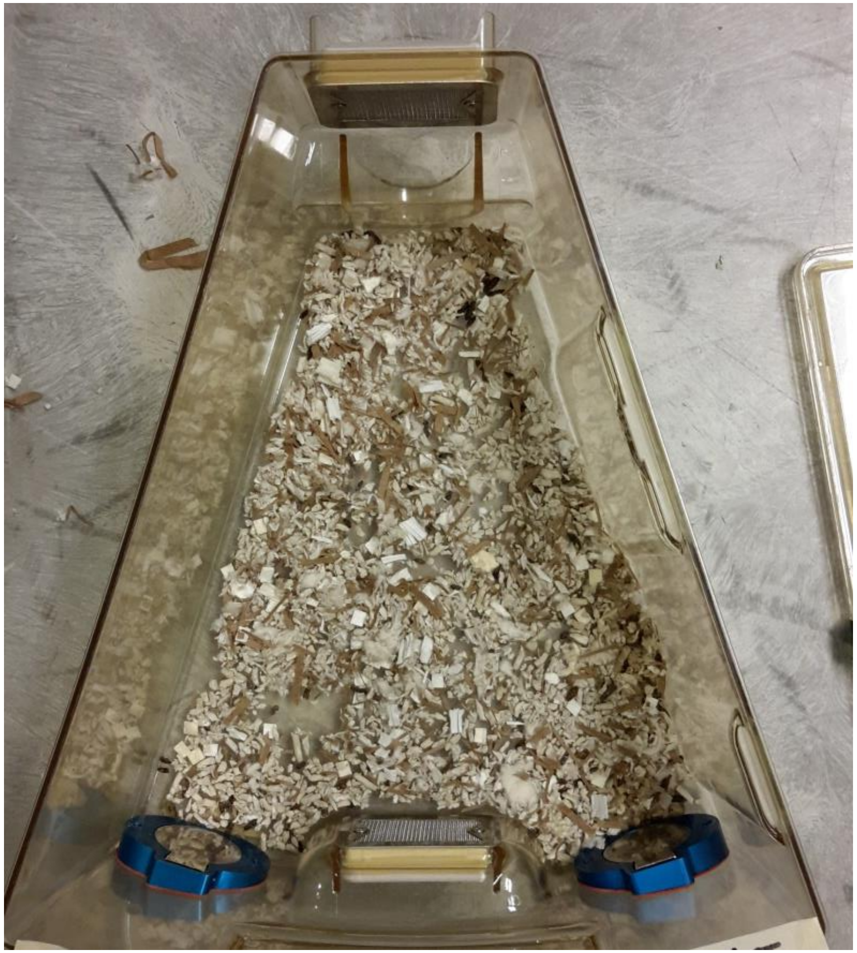

July 29 STD 3

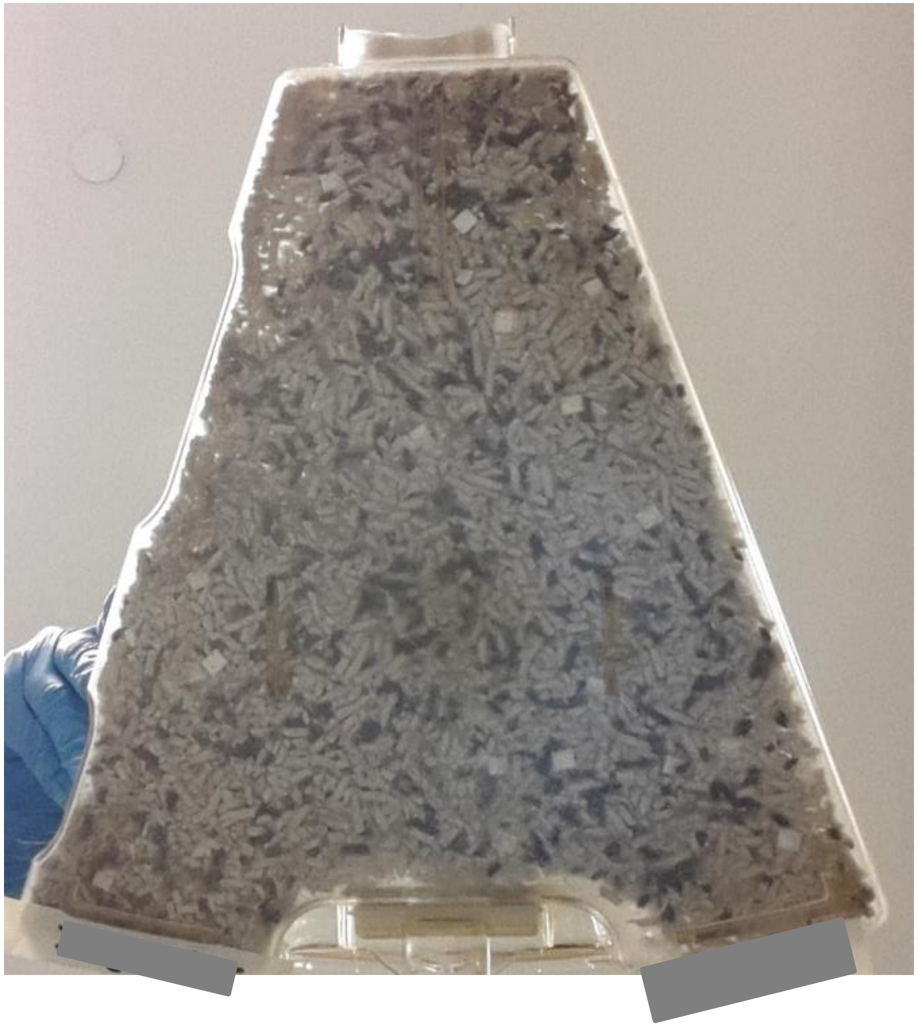

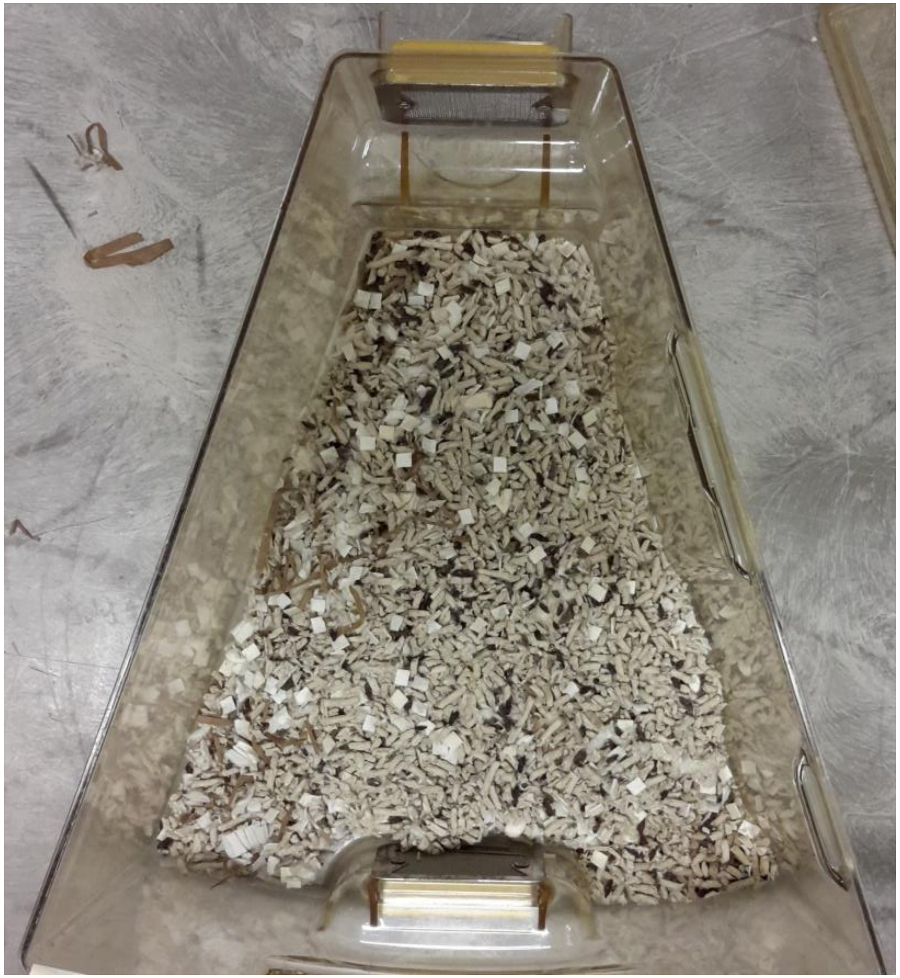

July 29 COMP 4 left

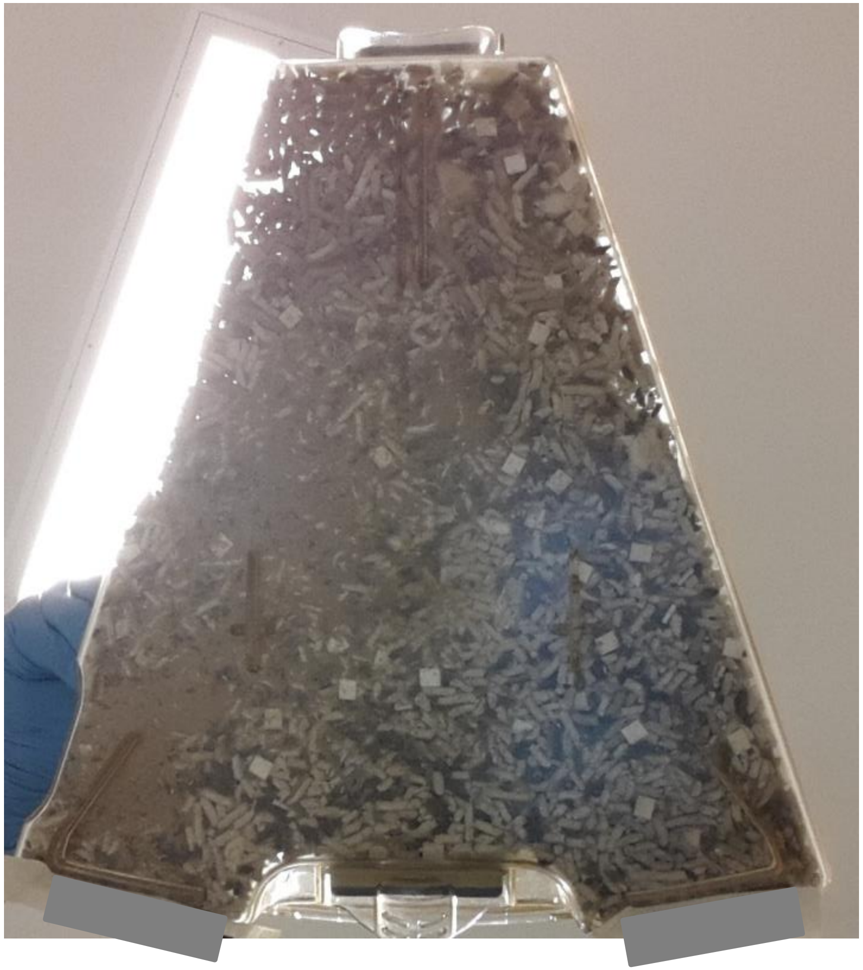

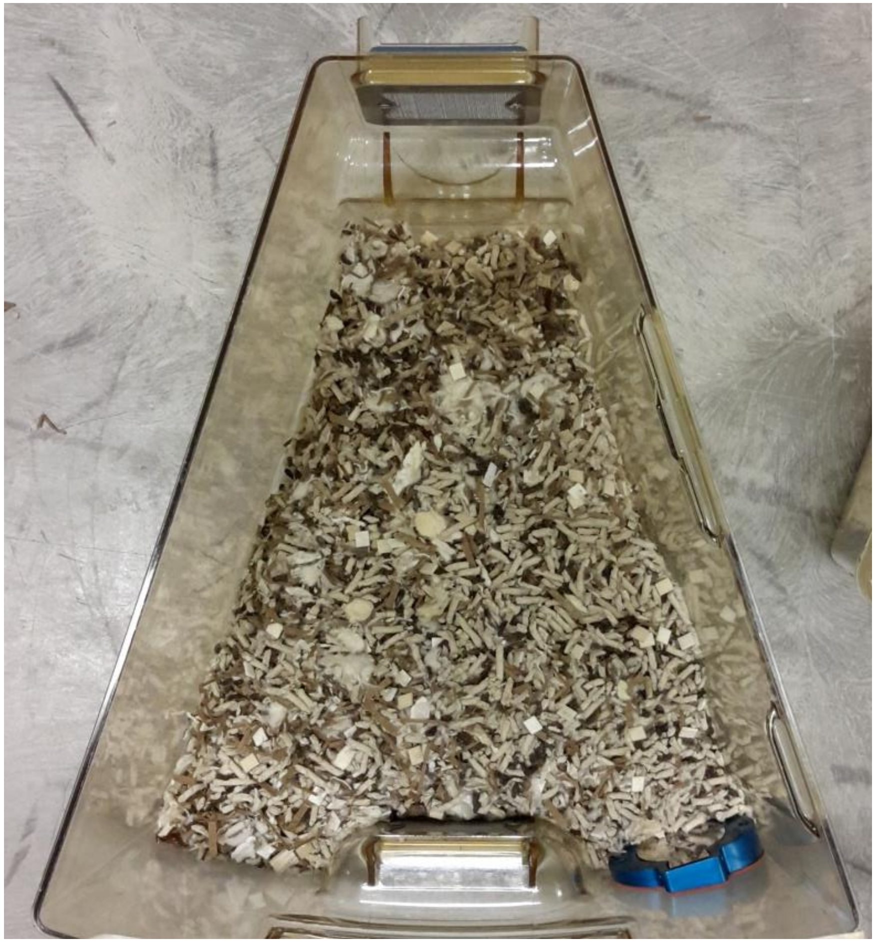

July 29 COMP 4 mid

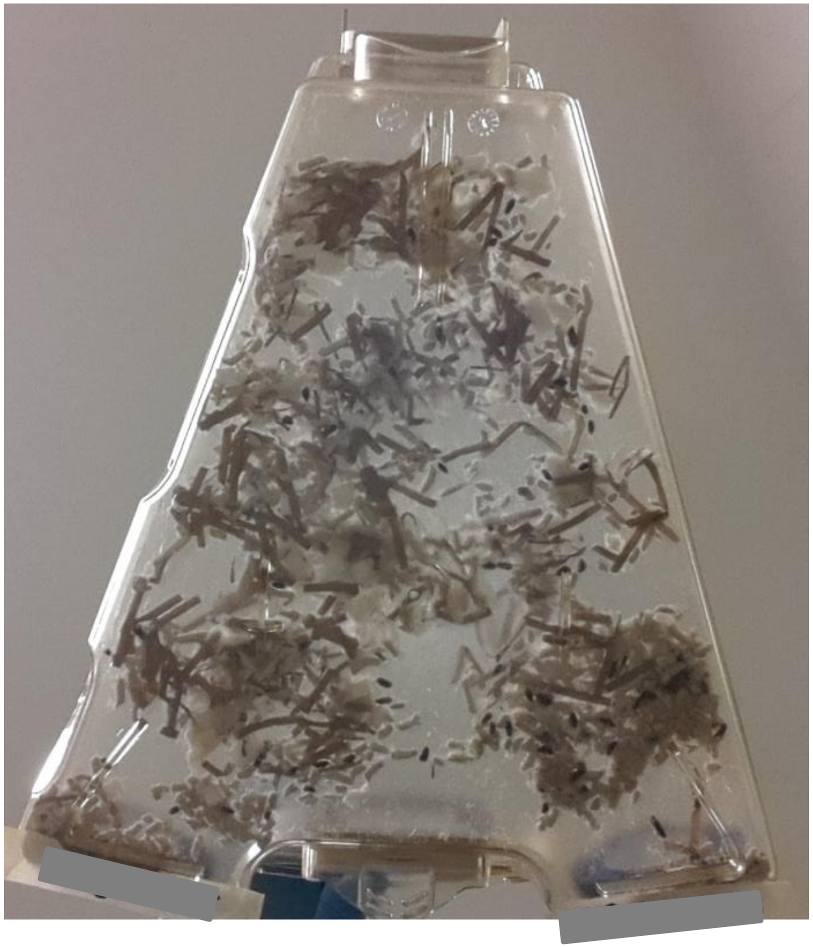

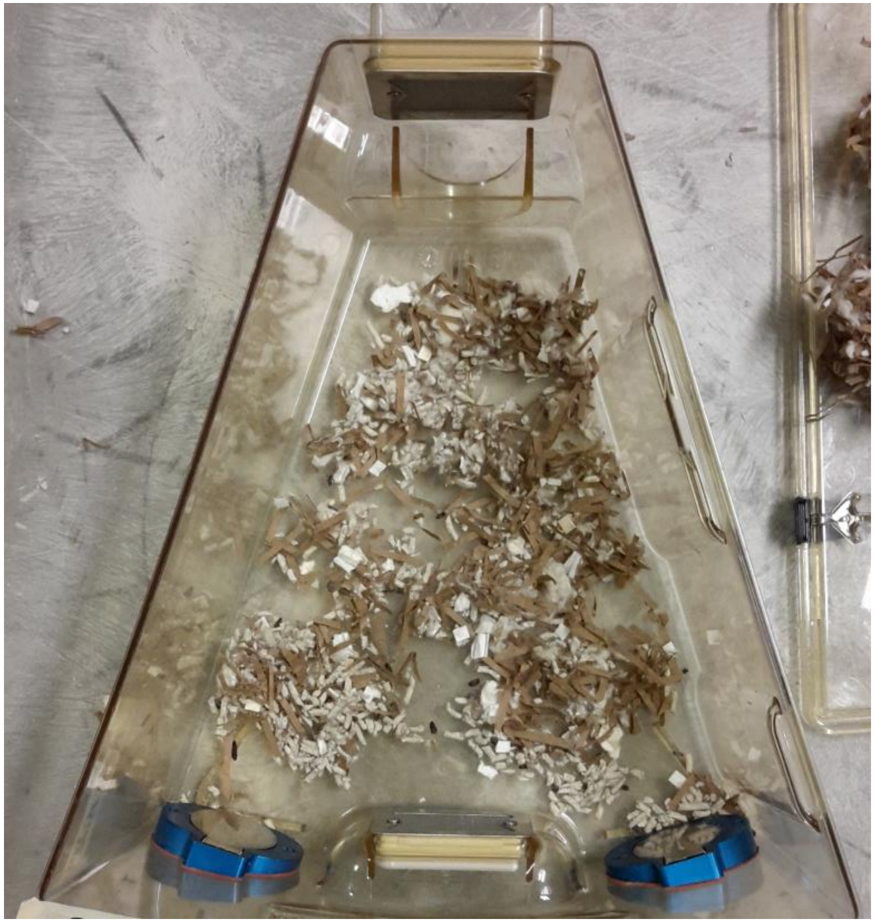

July 29 COMP 4 right

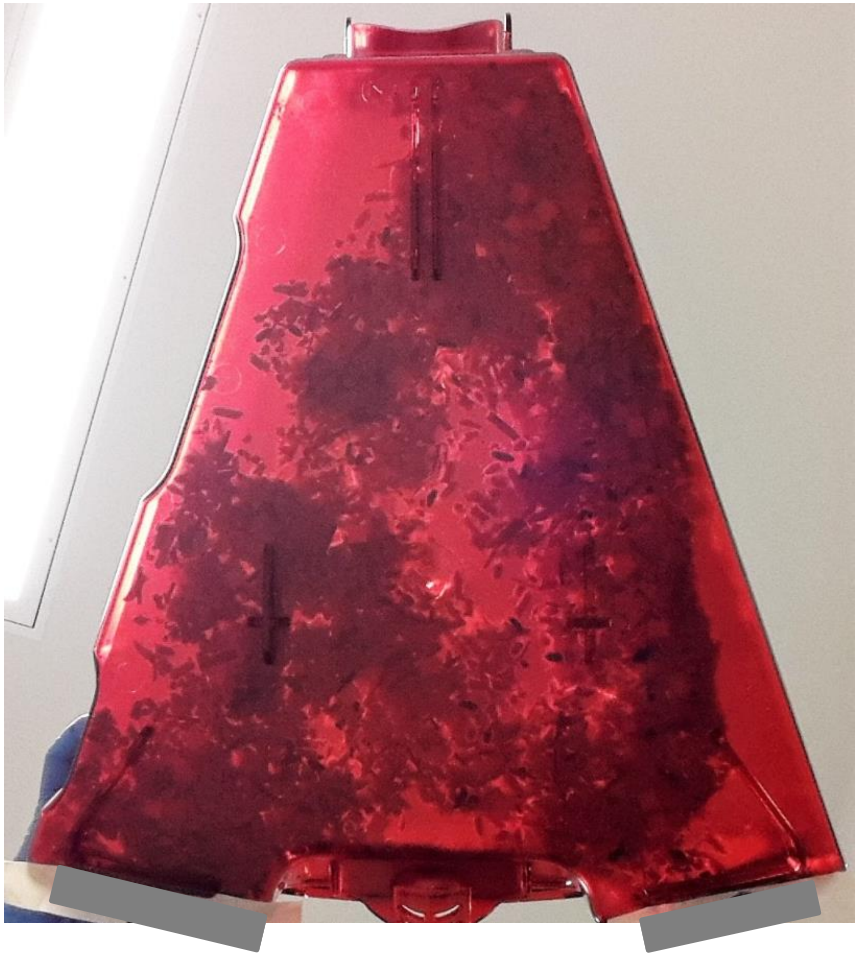

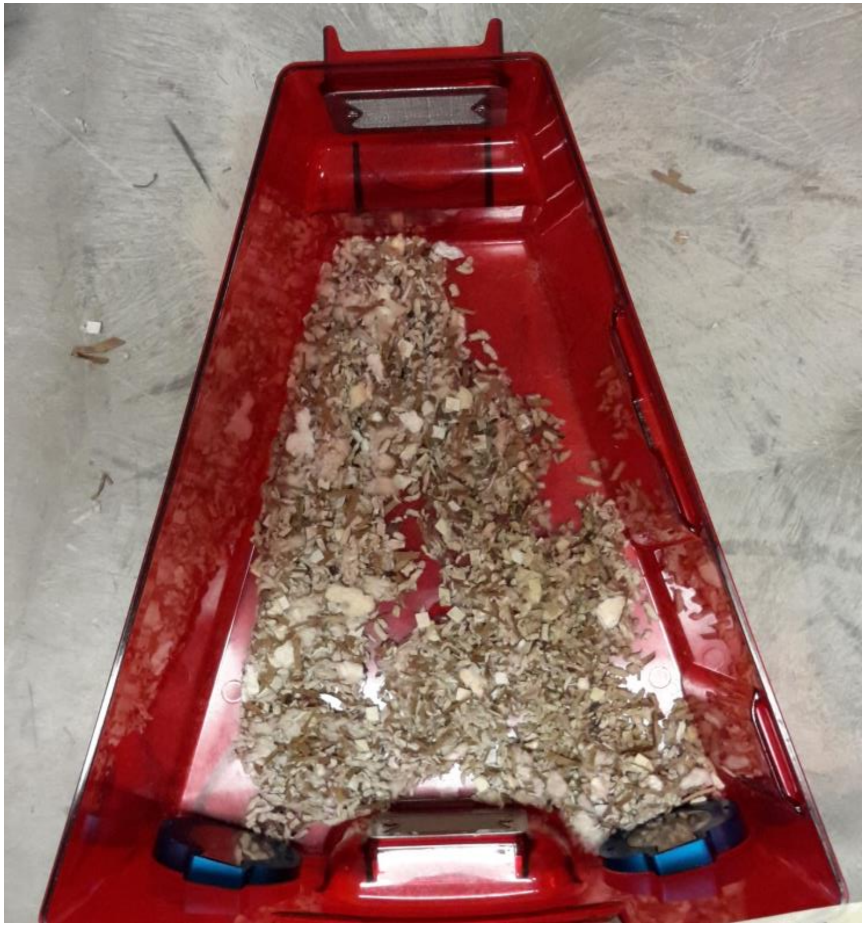

July 29 STD 4

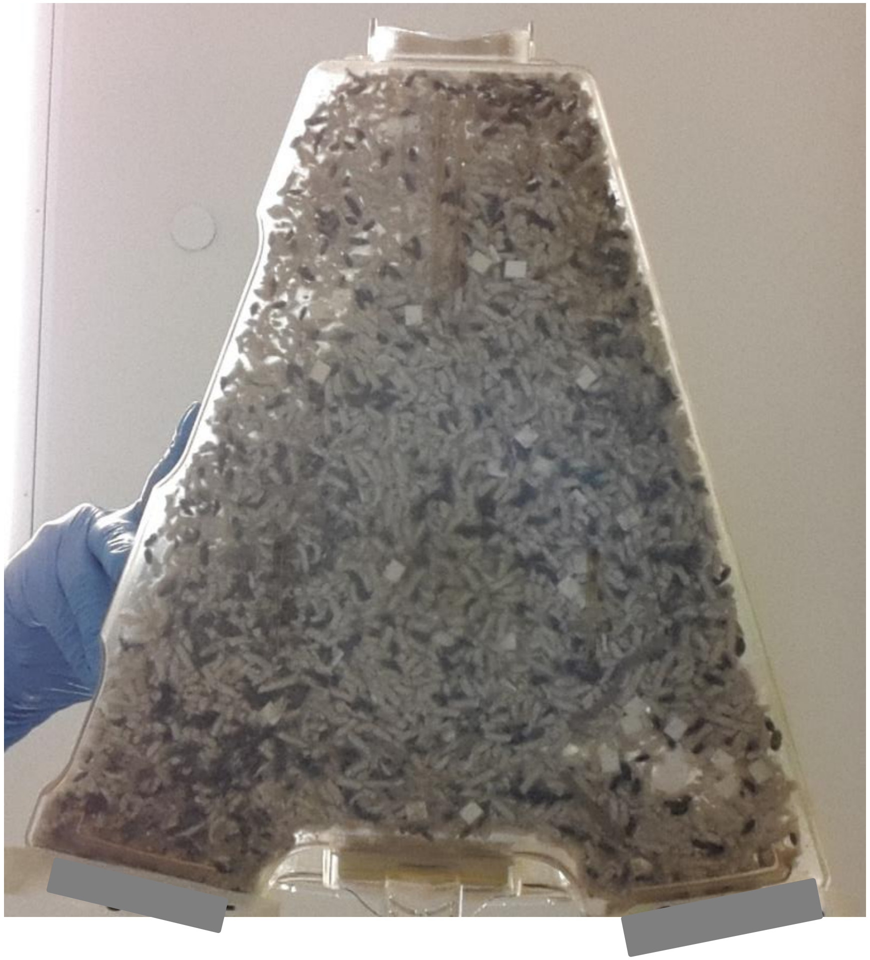

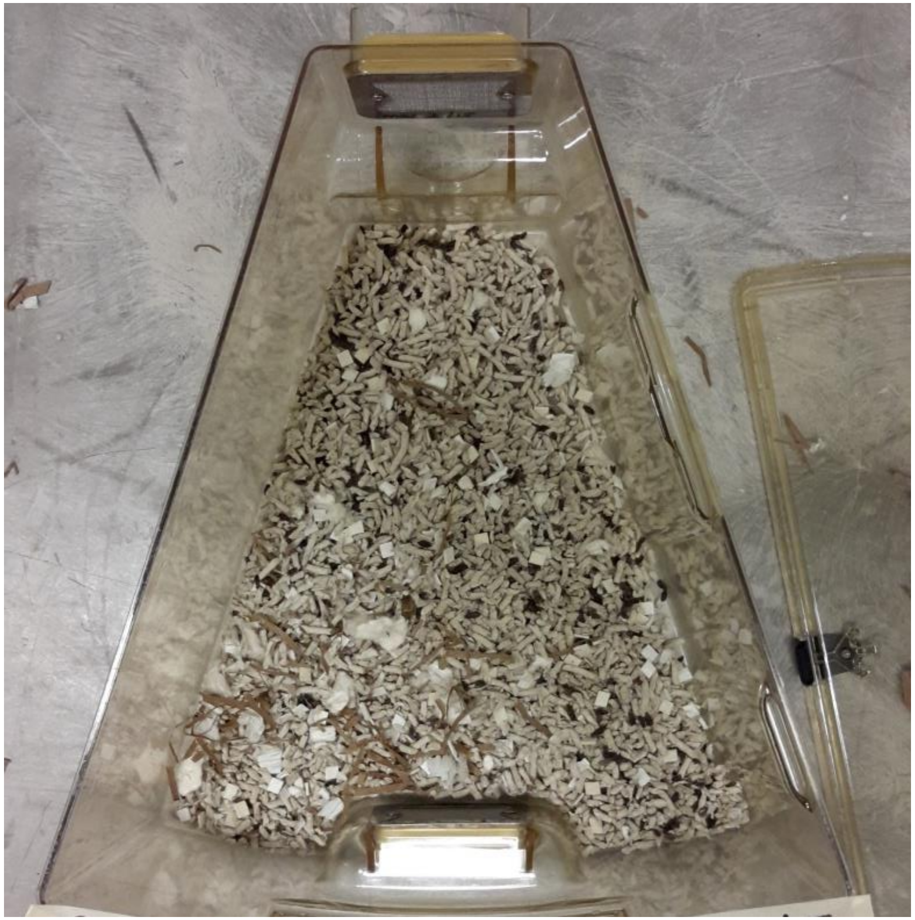

July 30 COMP 5 right

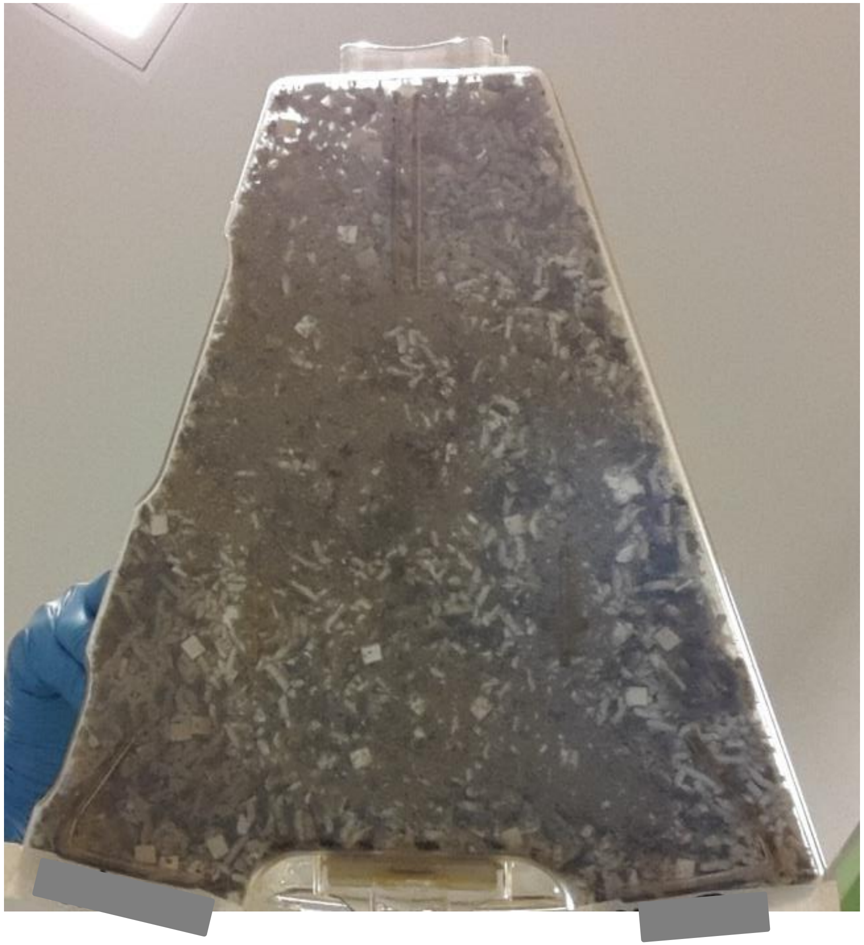

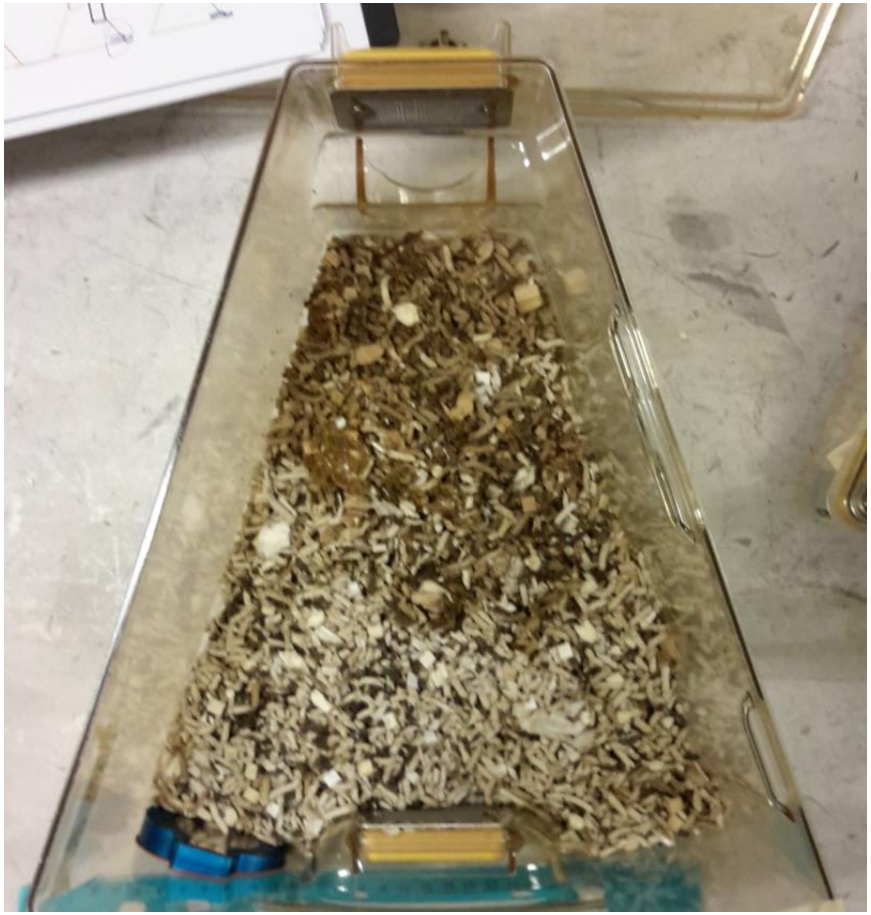

July 30 COMP 5 left

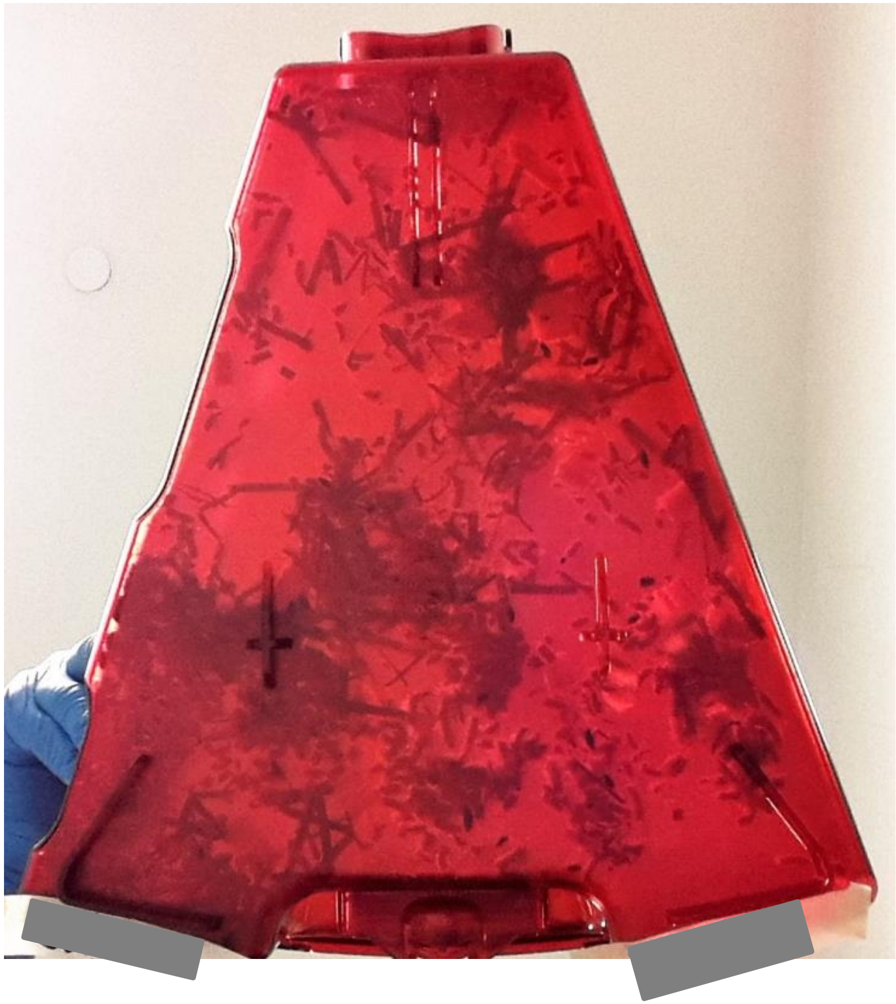

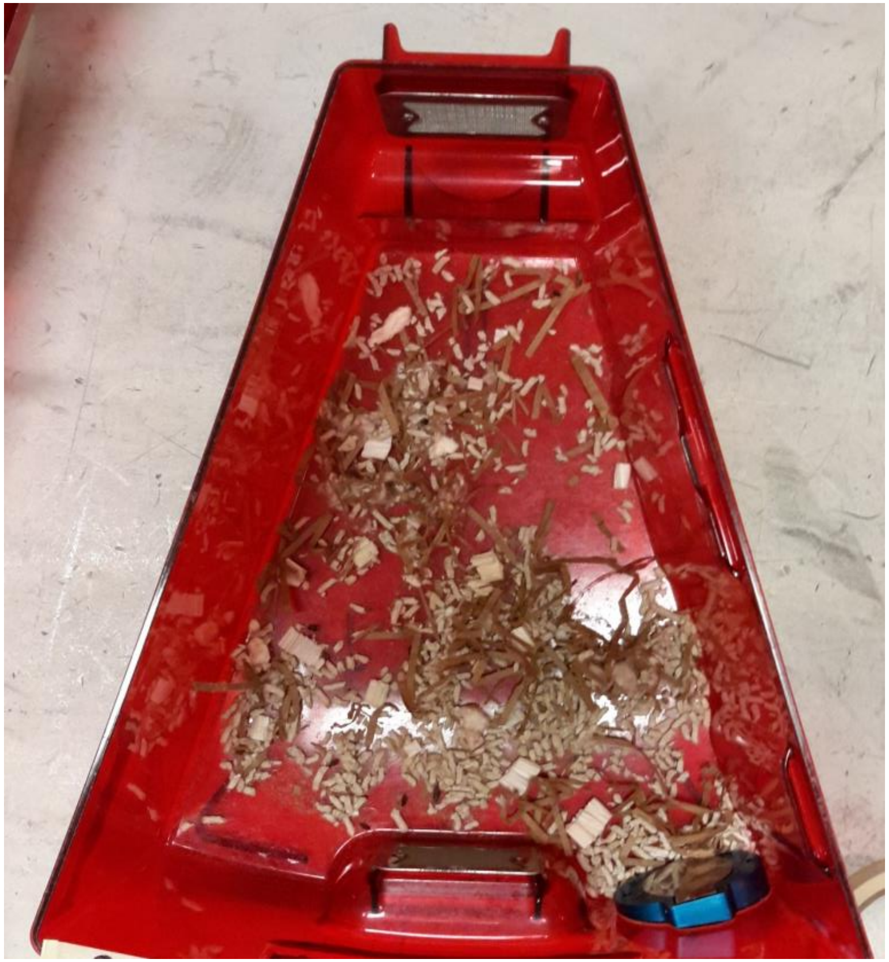

July 30 COMP 5 mid

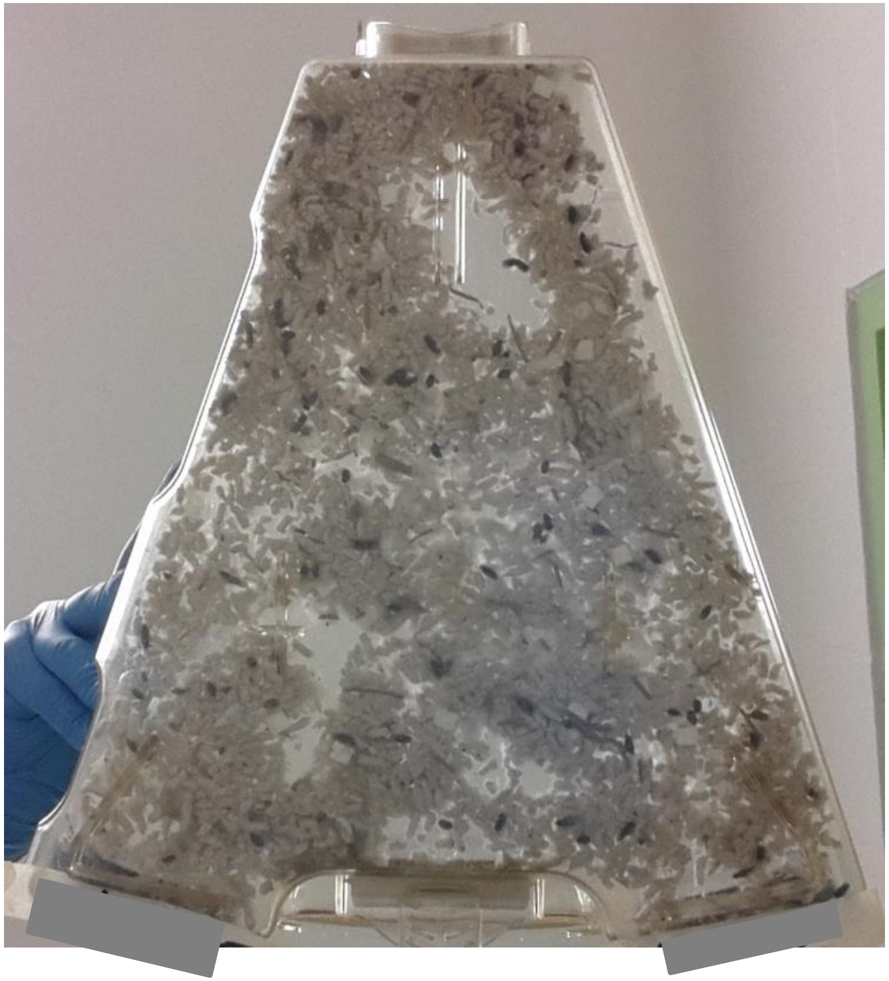

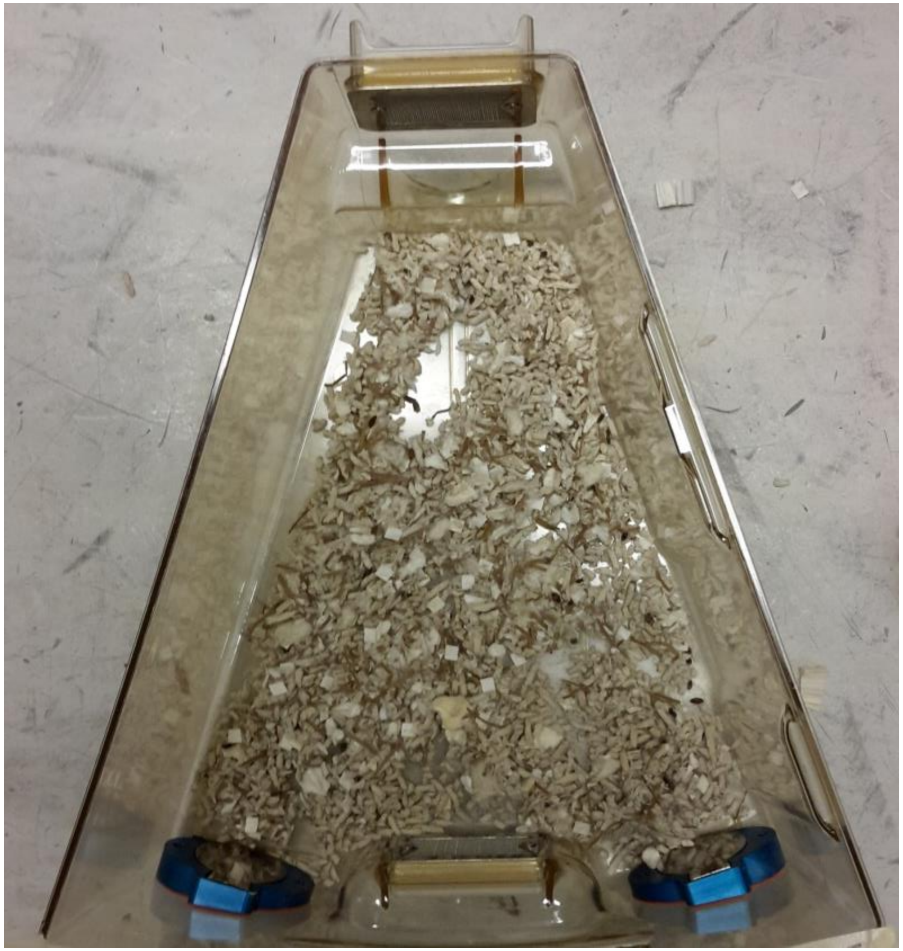

July 30 STD 5

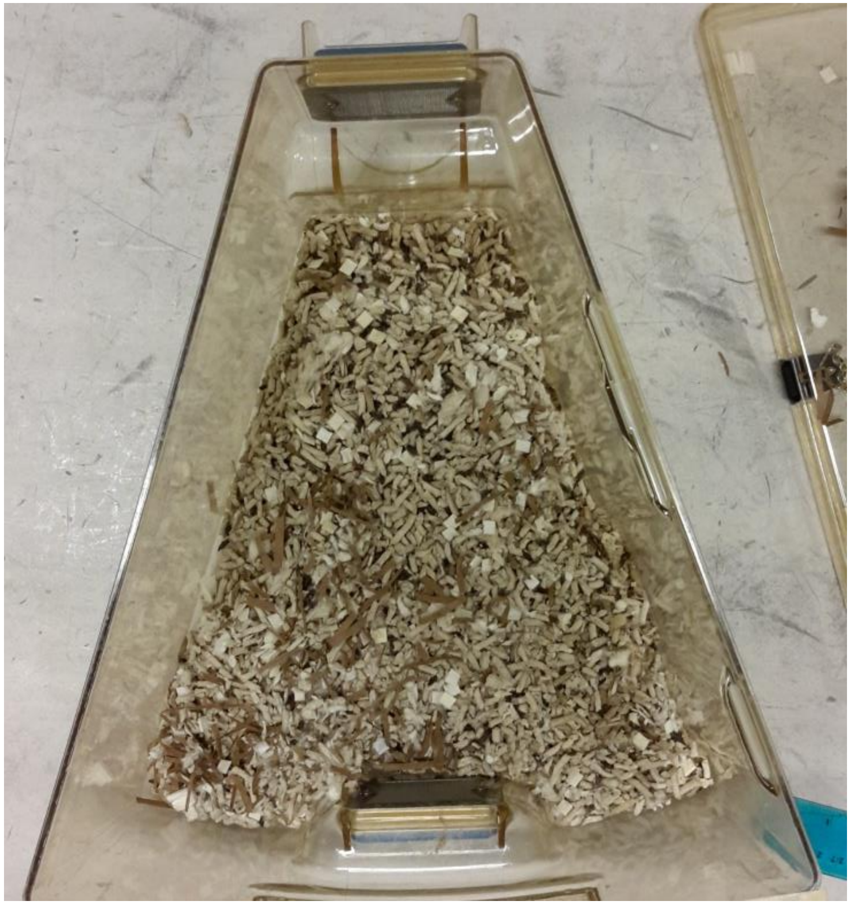

Aug 11 COMP 1 mid

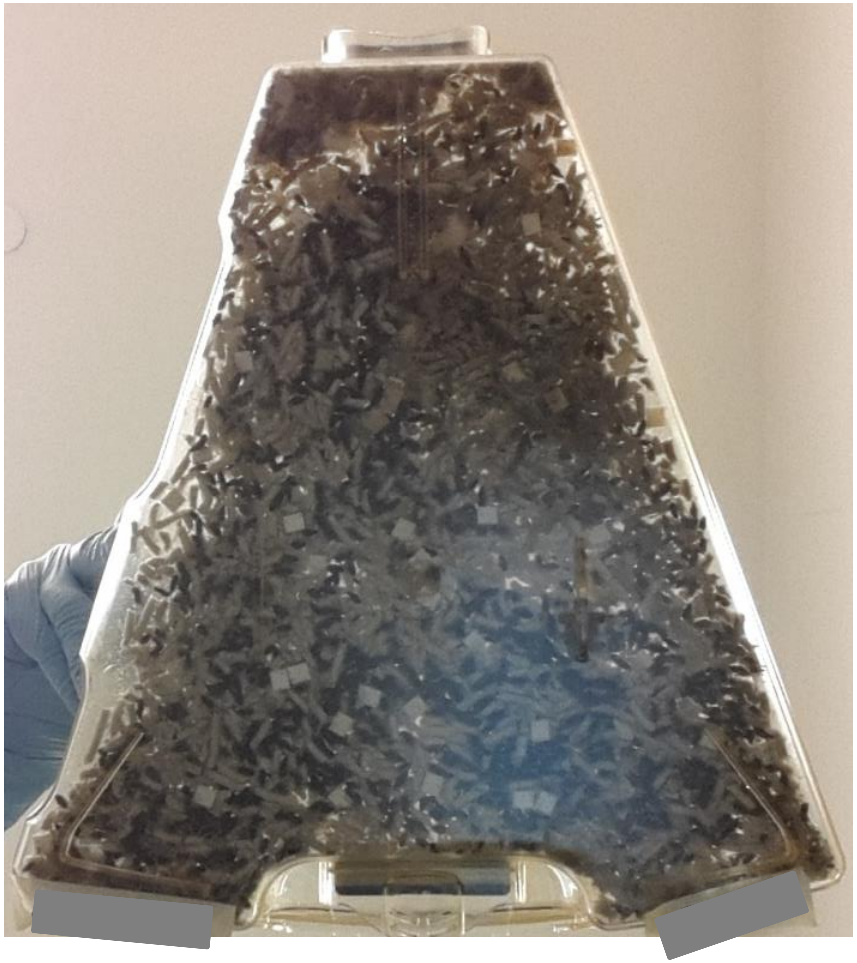

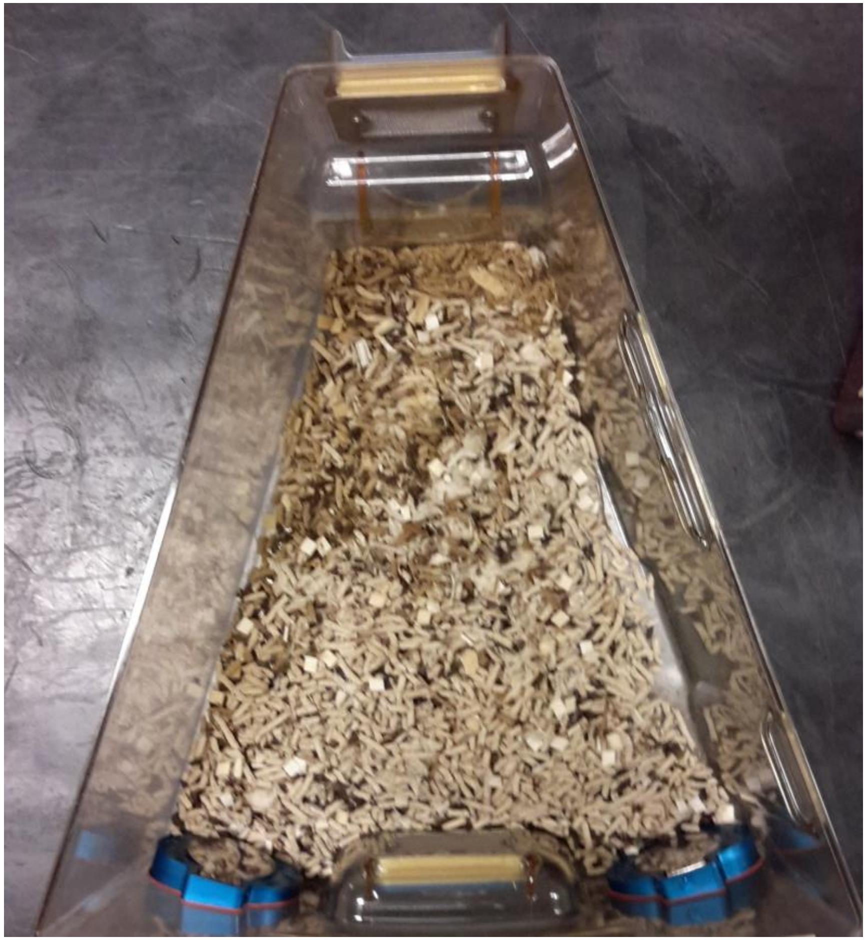

Aug 11 COMP 1 right

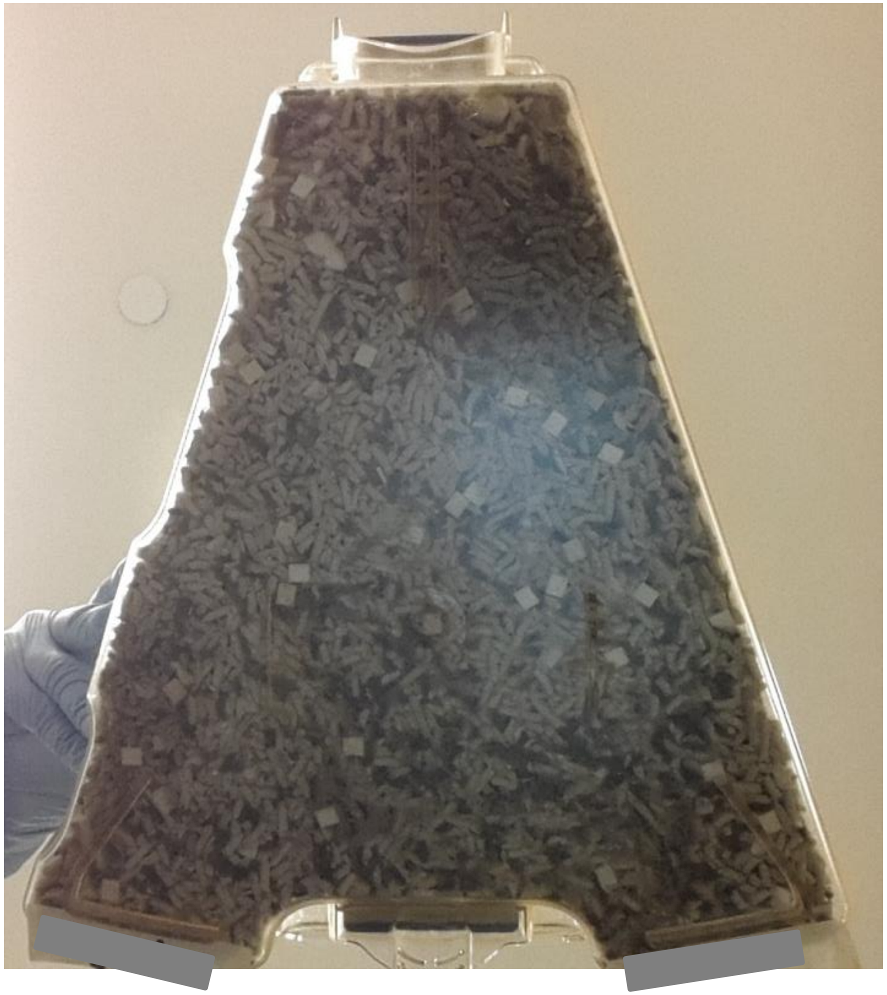

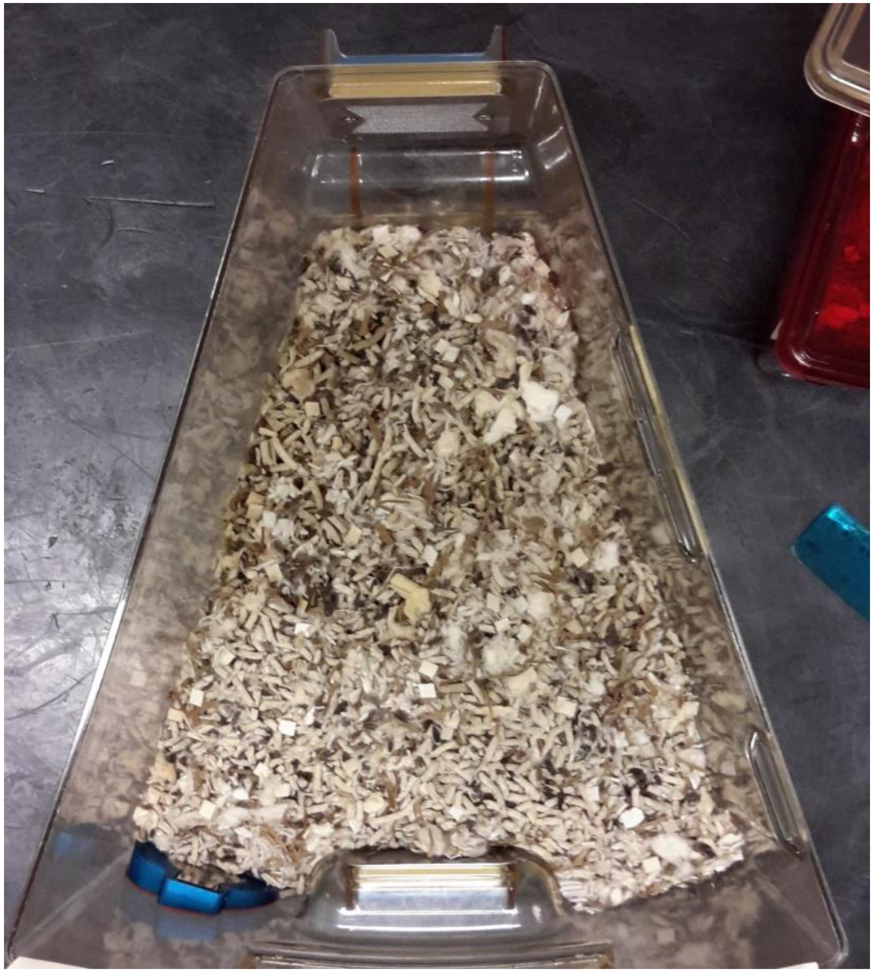

Aug 11 COMP 1 left

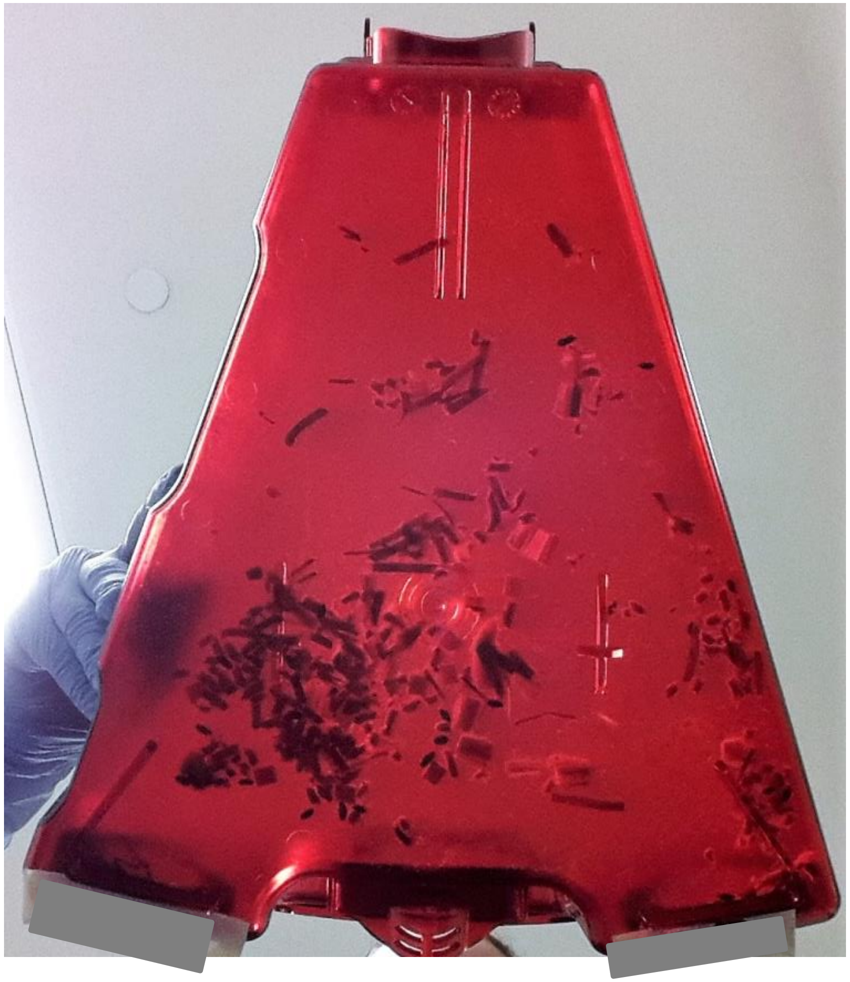

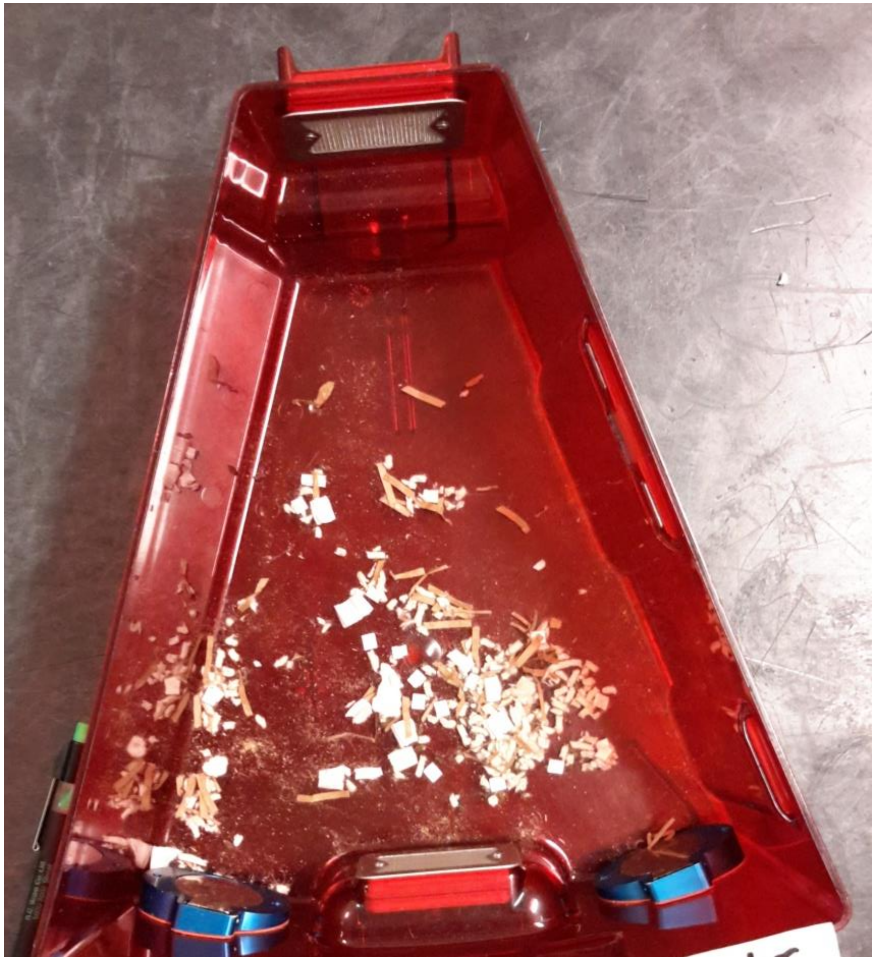

Aug 11 STD 1

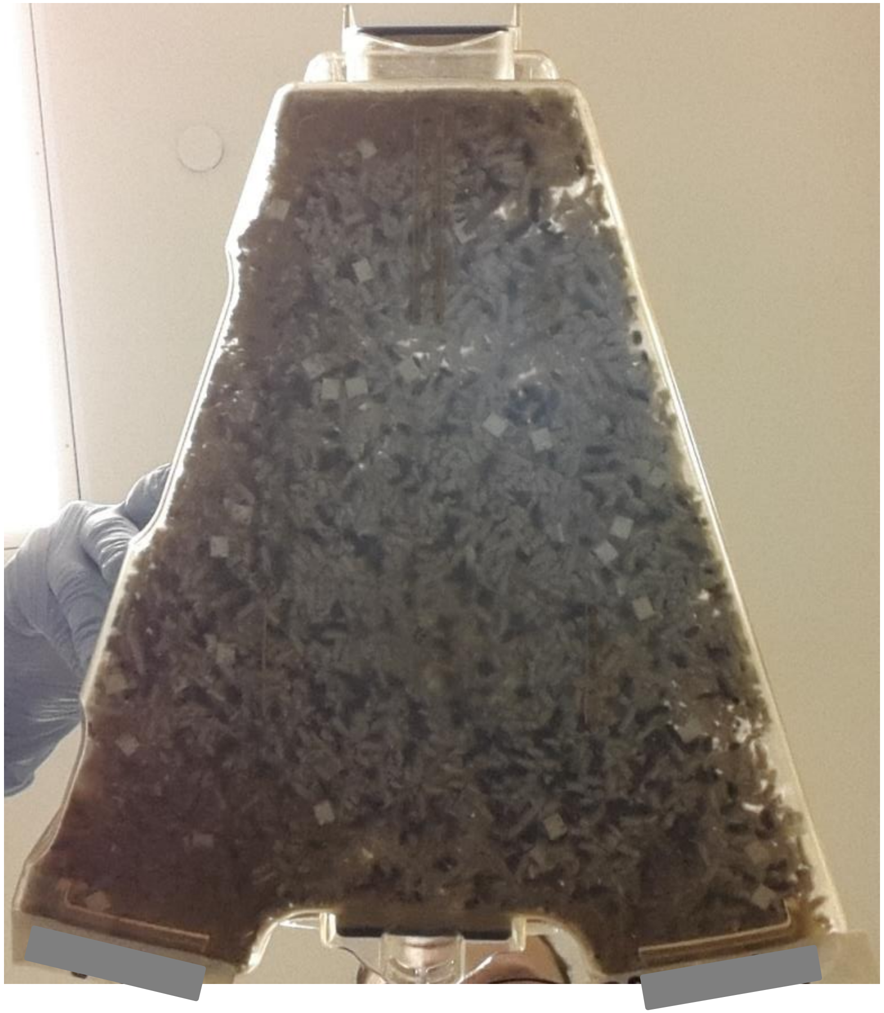

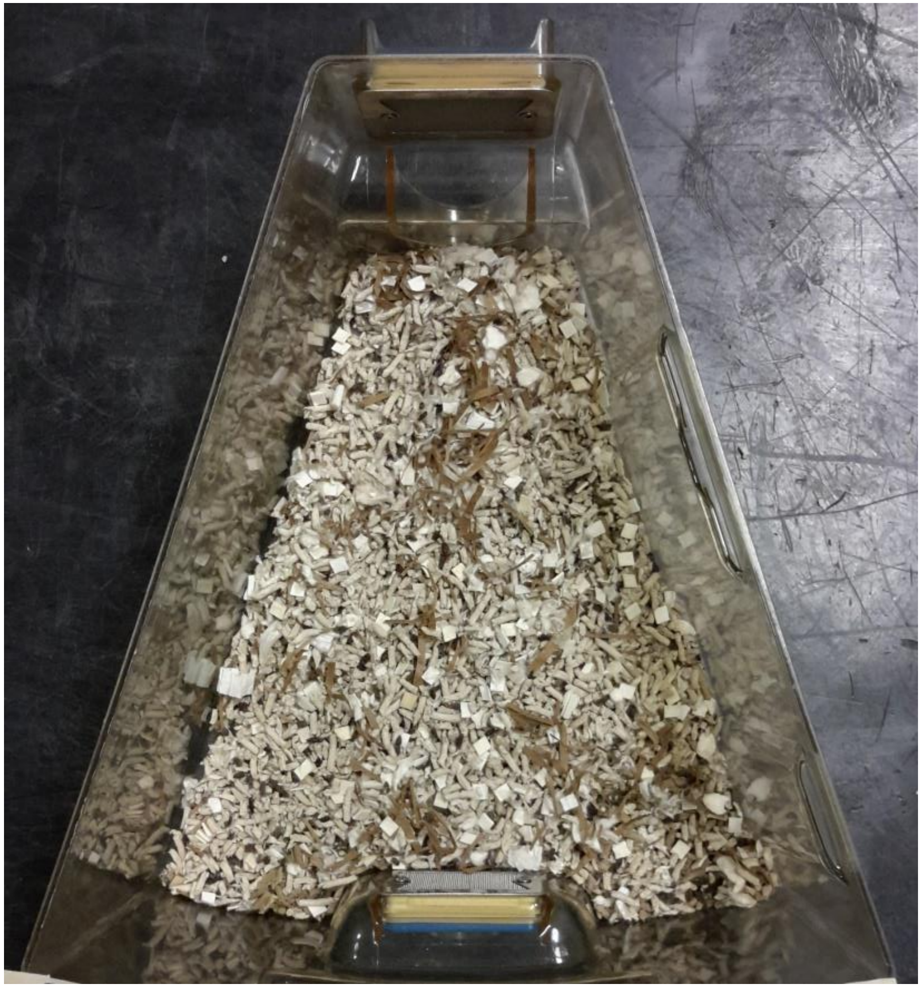

Aug 11 COMP 2 right

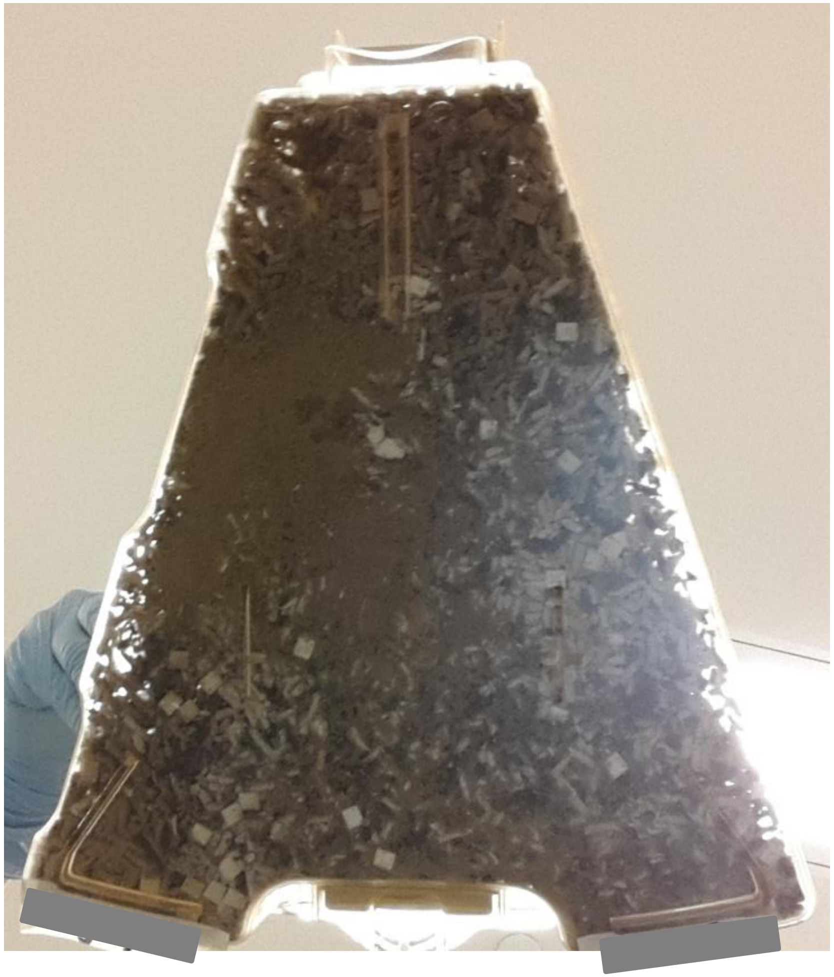

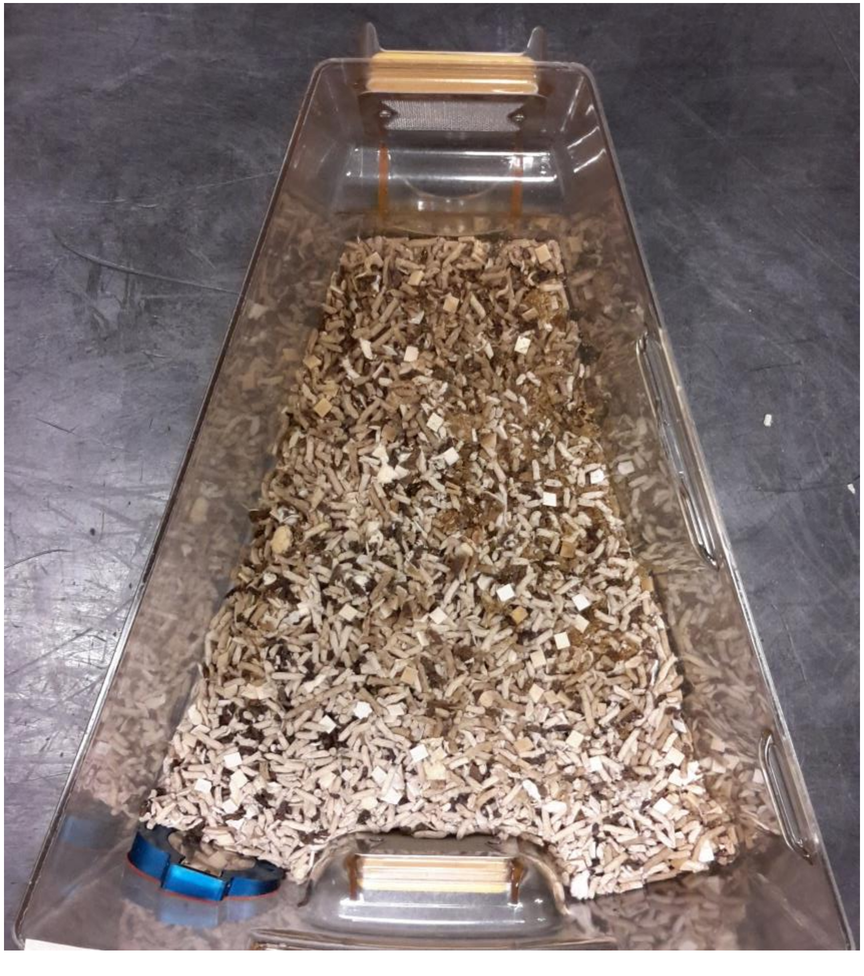

Aug 11 COMP 2 mid

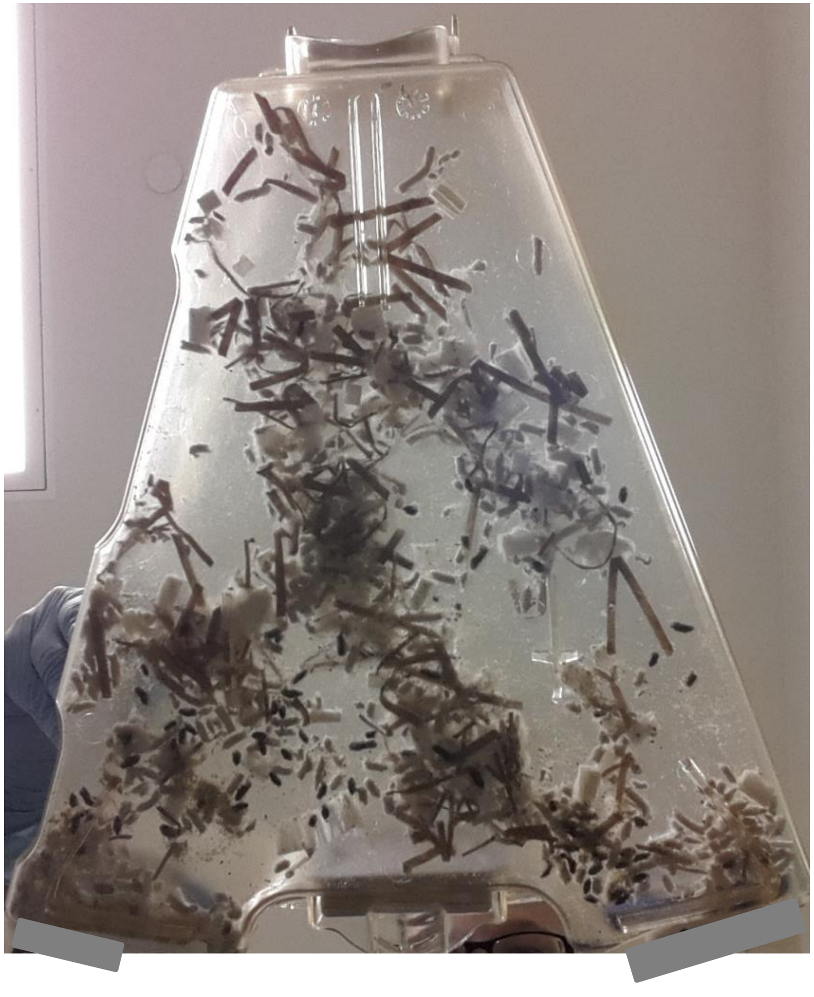

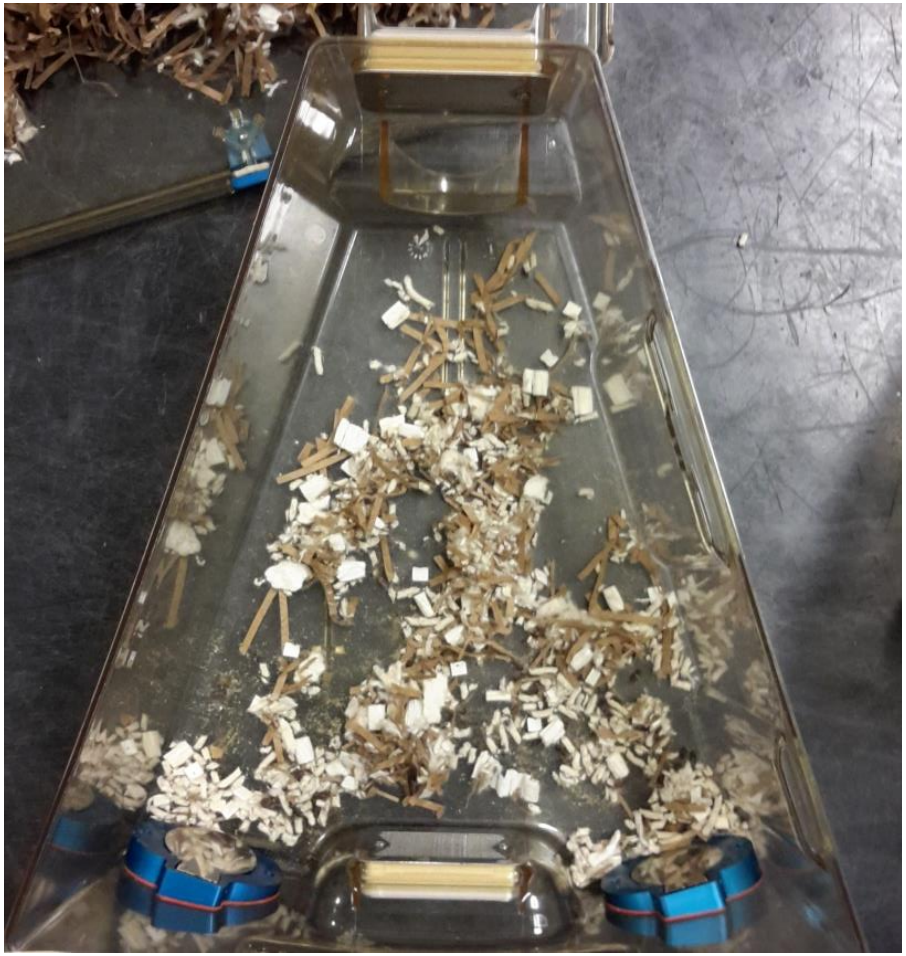

Aug 11 COMP 2 left

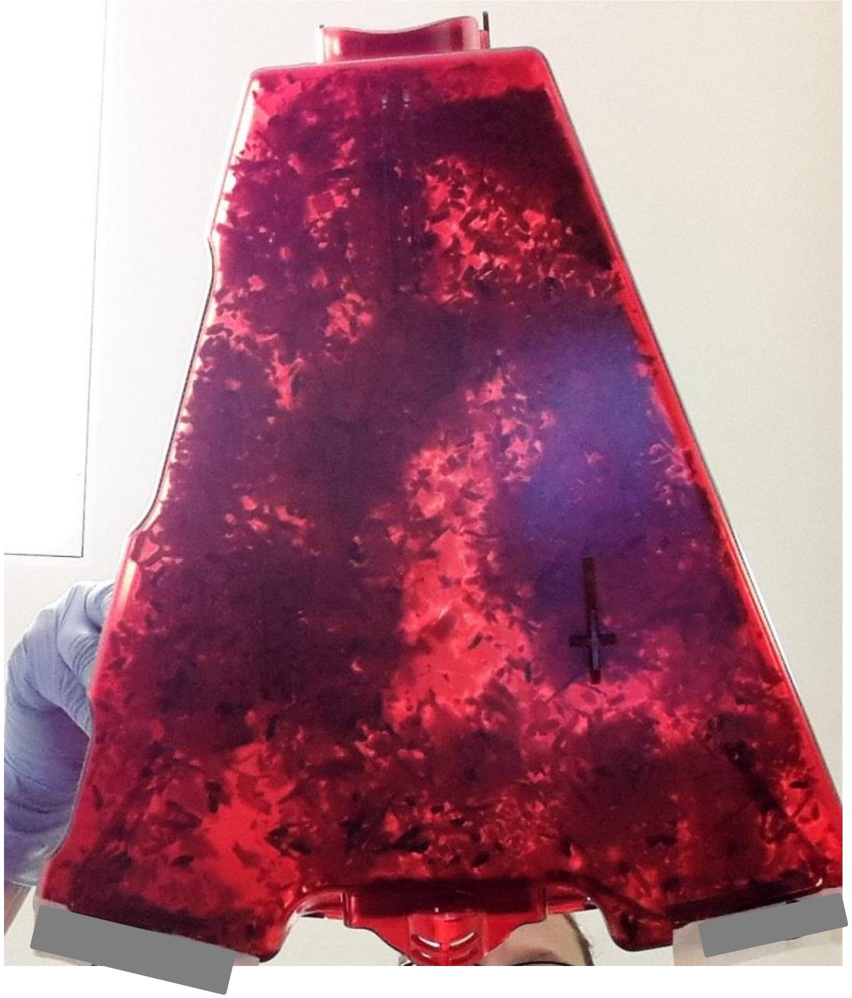

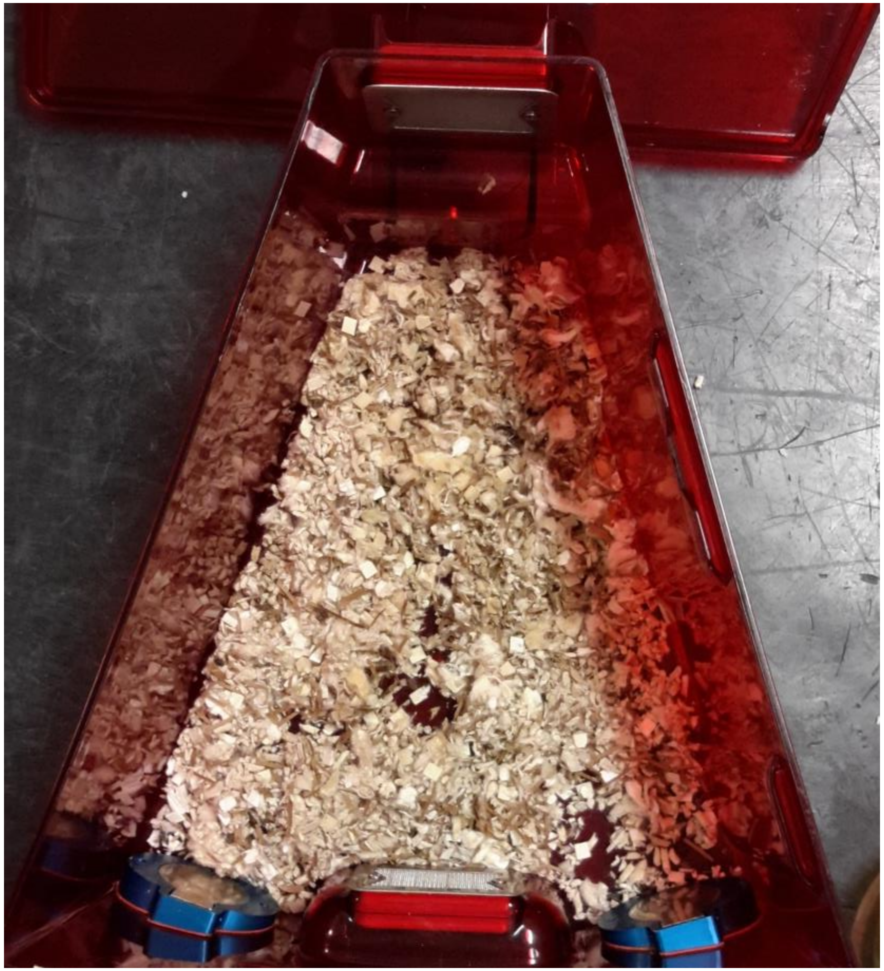

Aug 11 STD 2

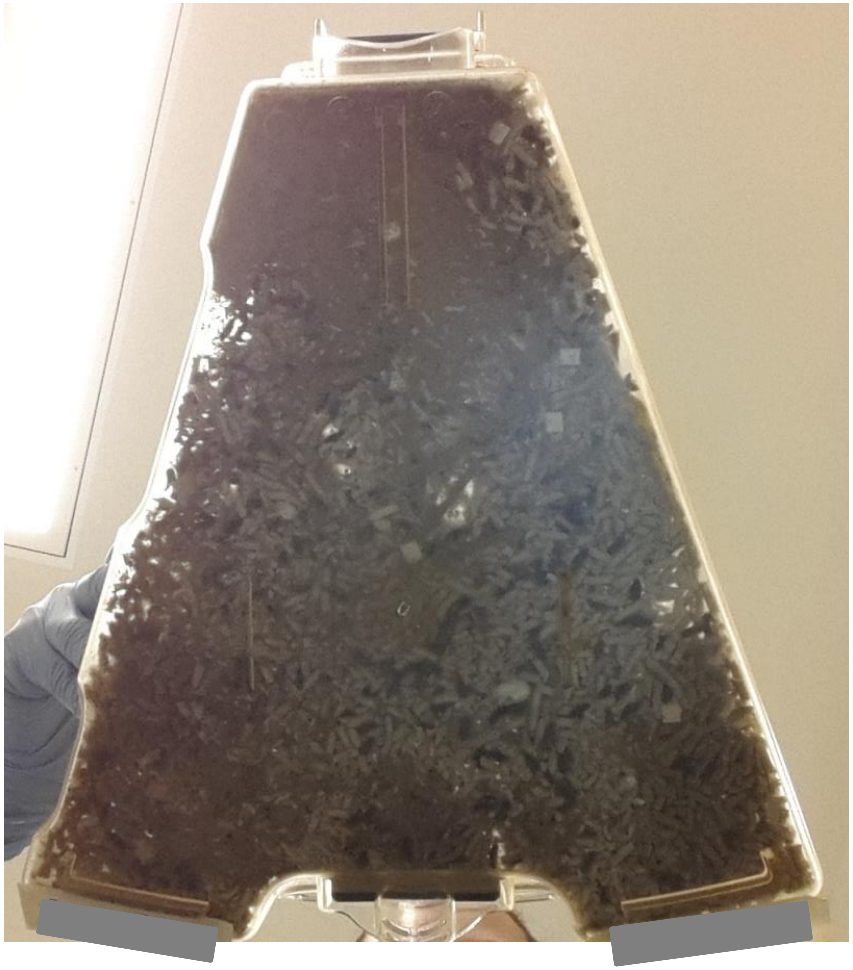

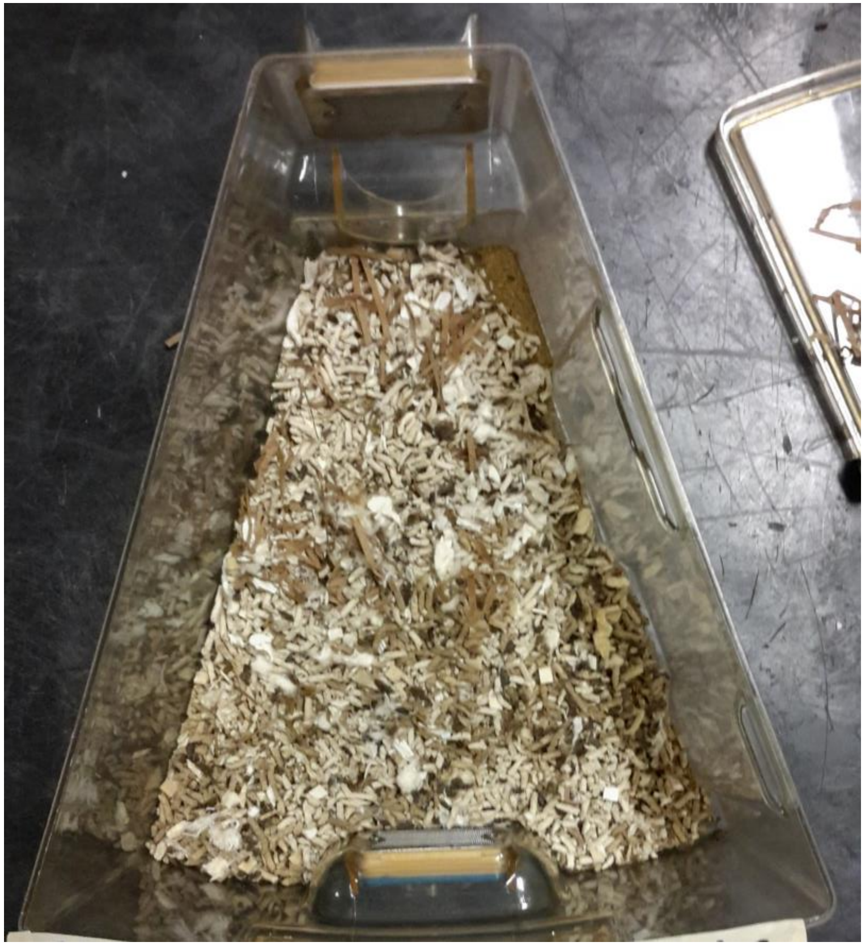

Aug 12 COMP 3 right

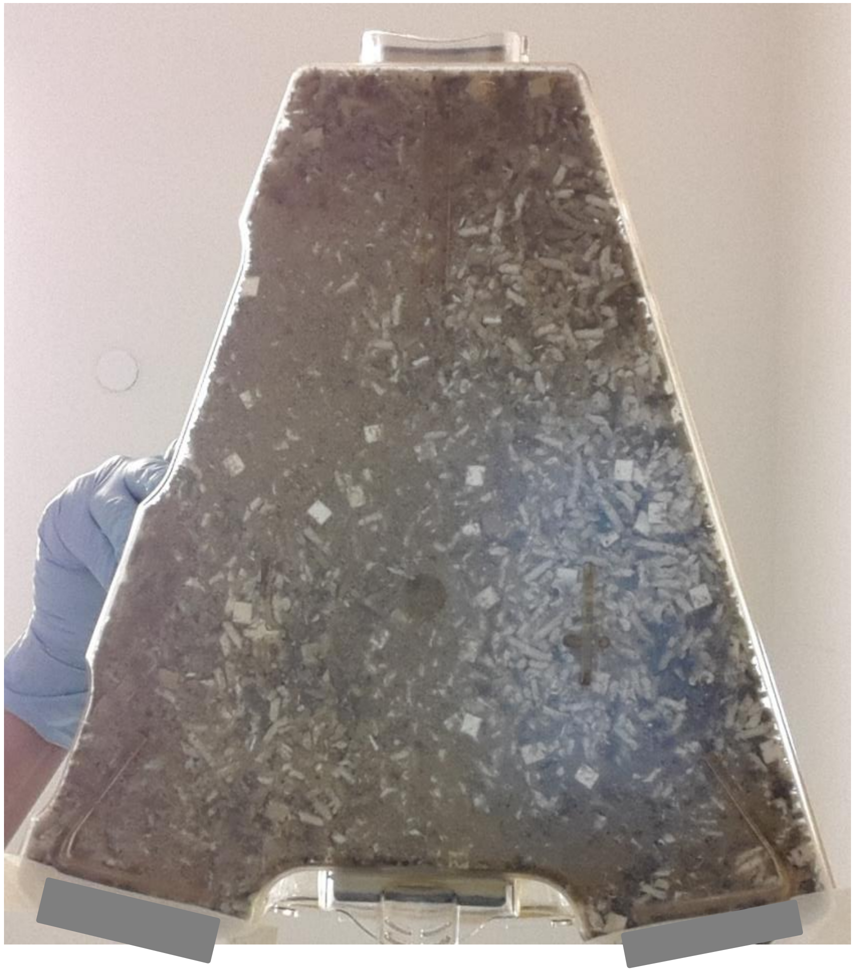

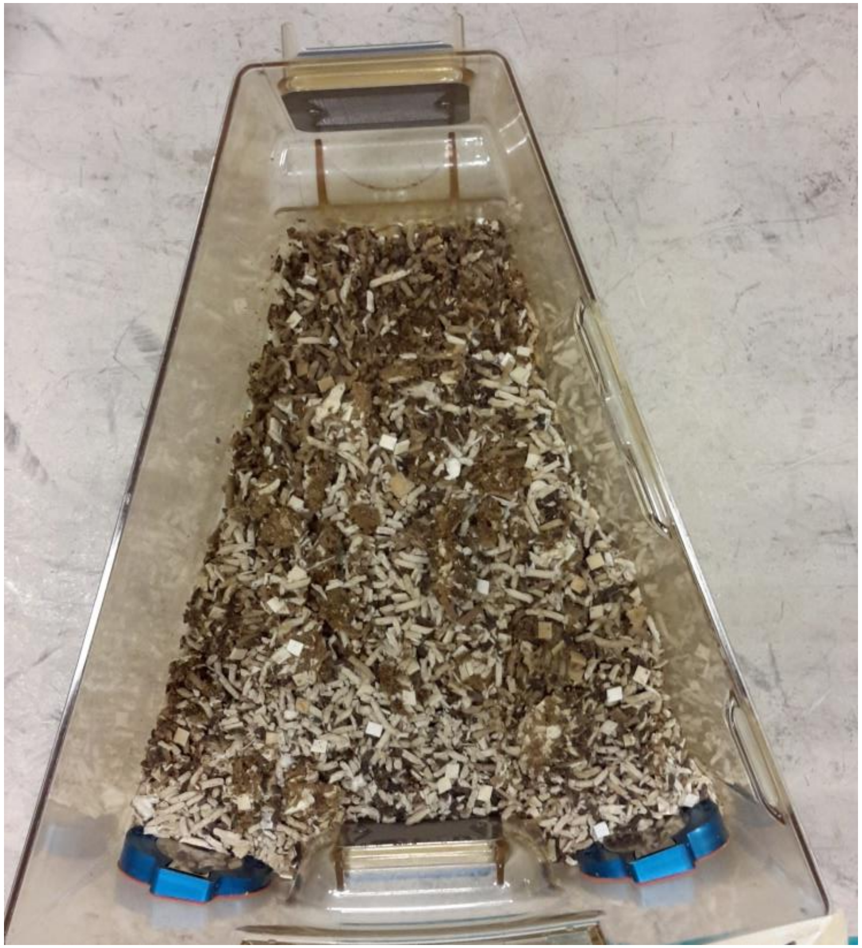

Aug 12 COMP 3 mid

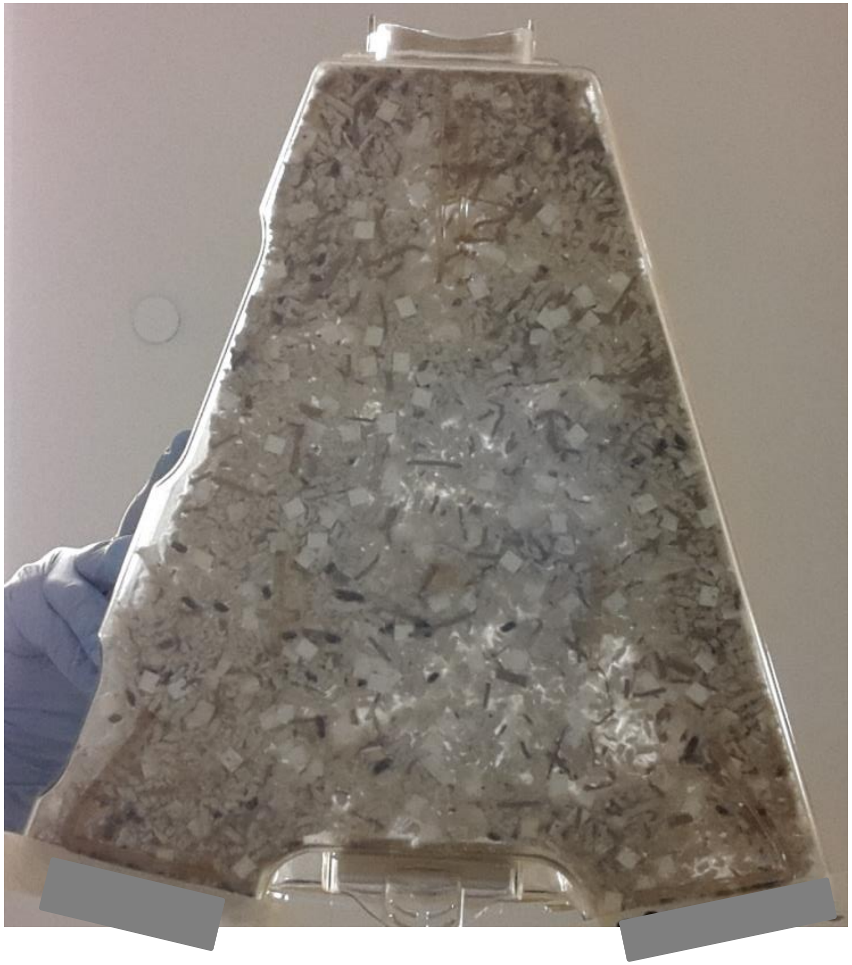

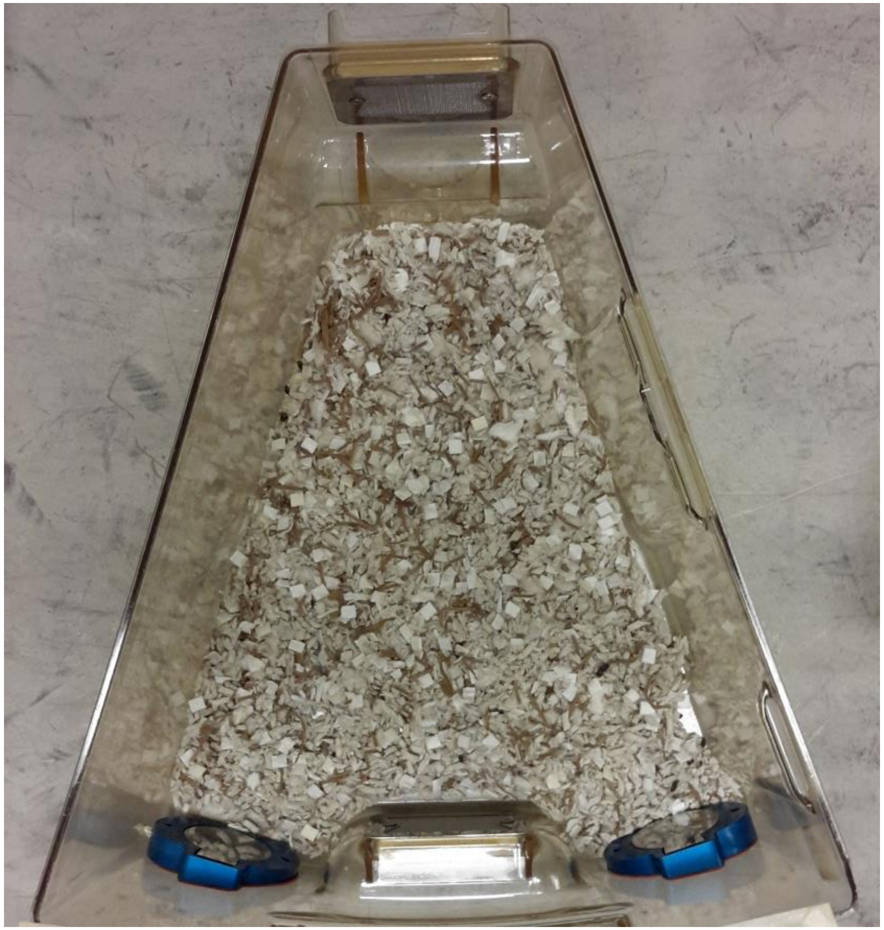

Aug 12 COMP 3 left

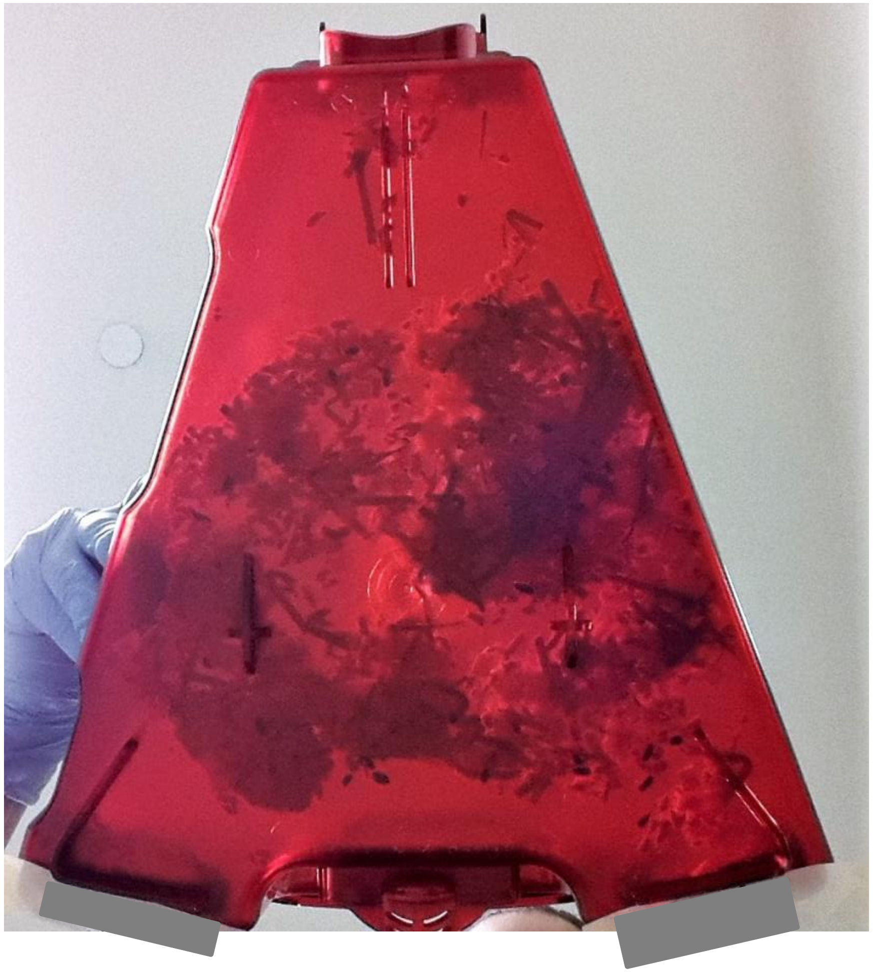

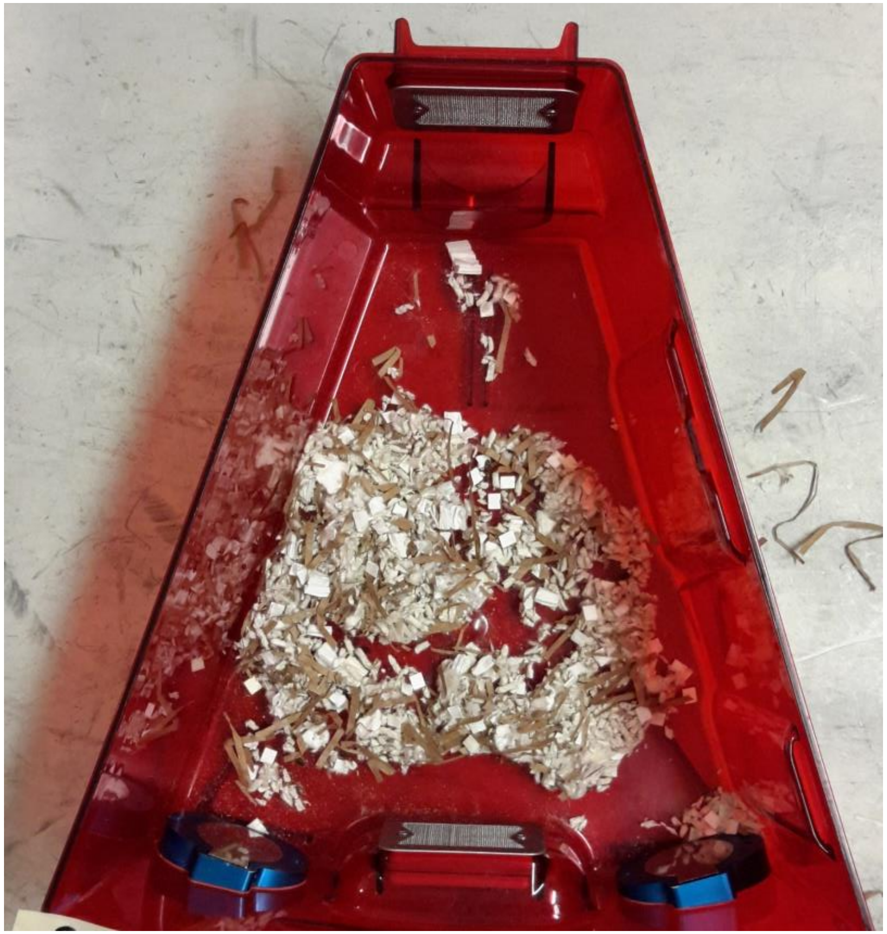

Aug 12 STD 3

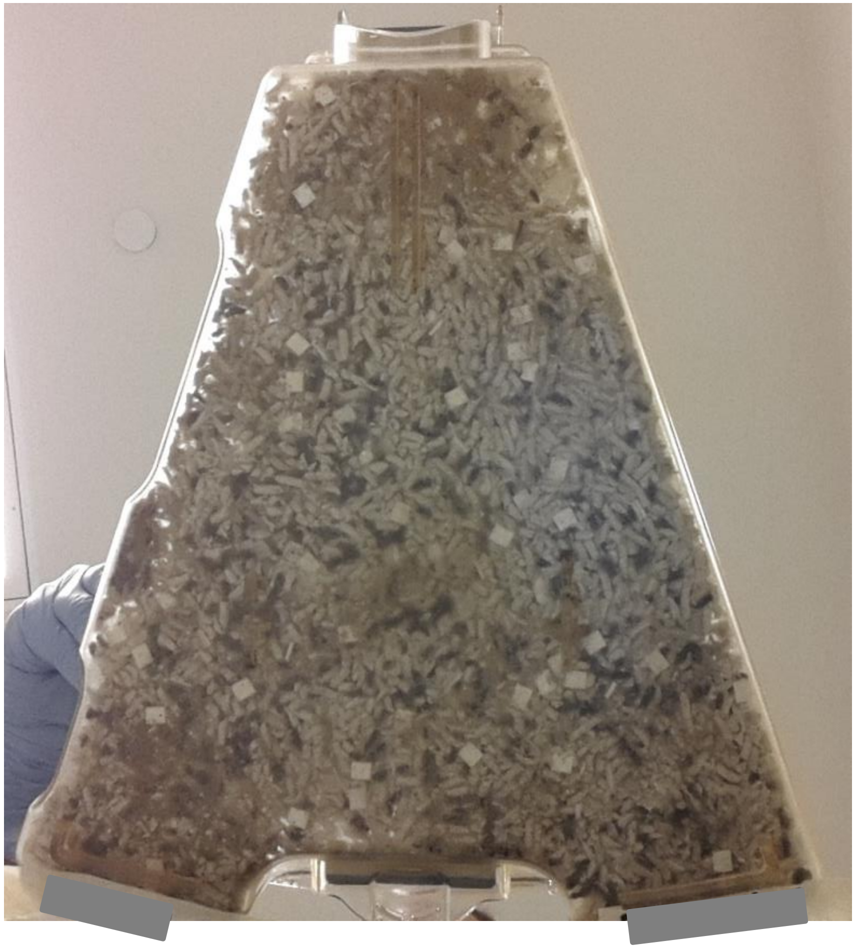

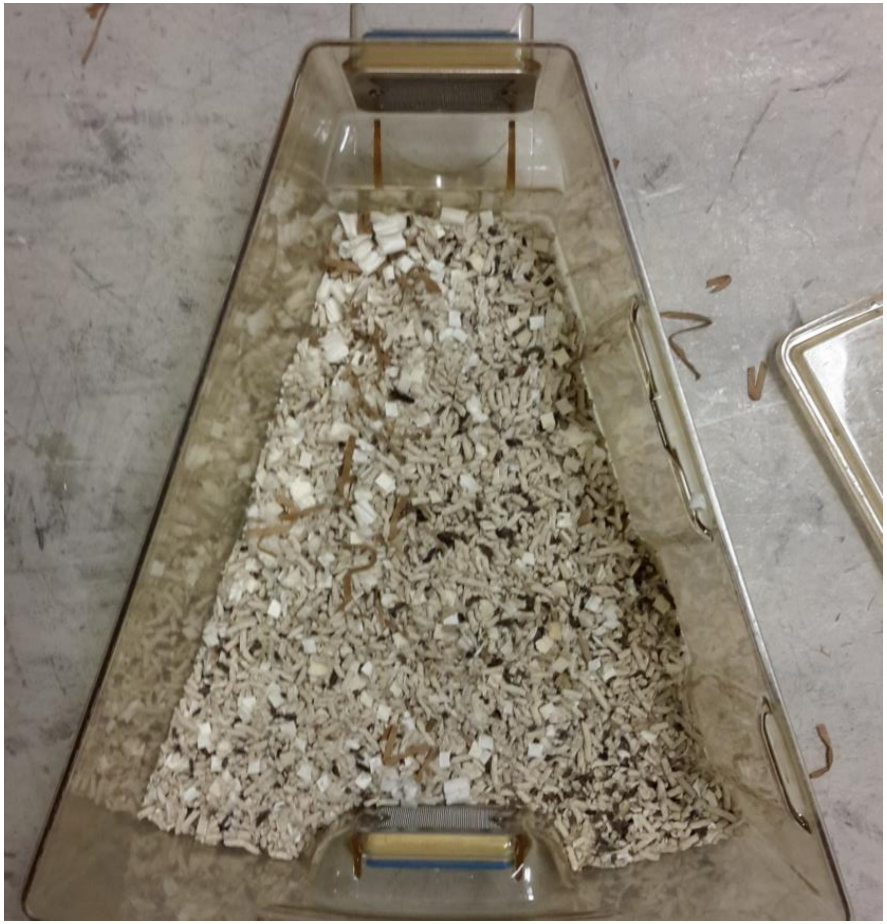

Aug 12 COMP 4 right

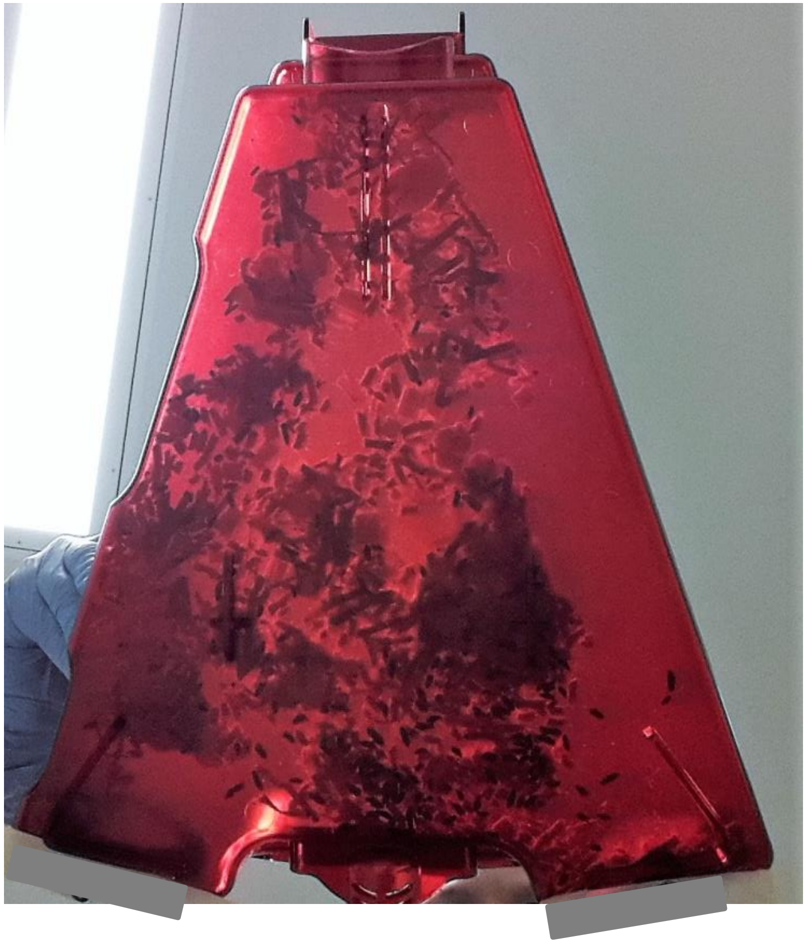

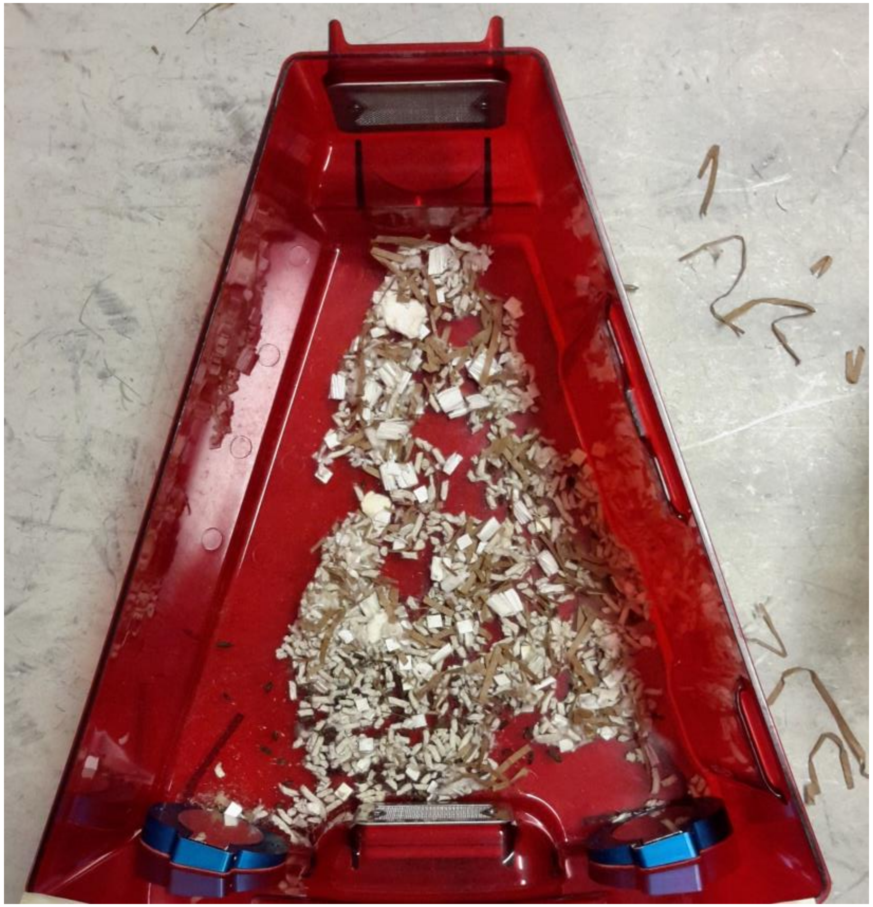

Aug 12 COMP 4 left

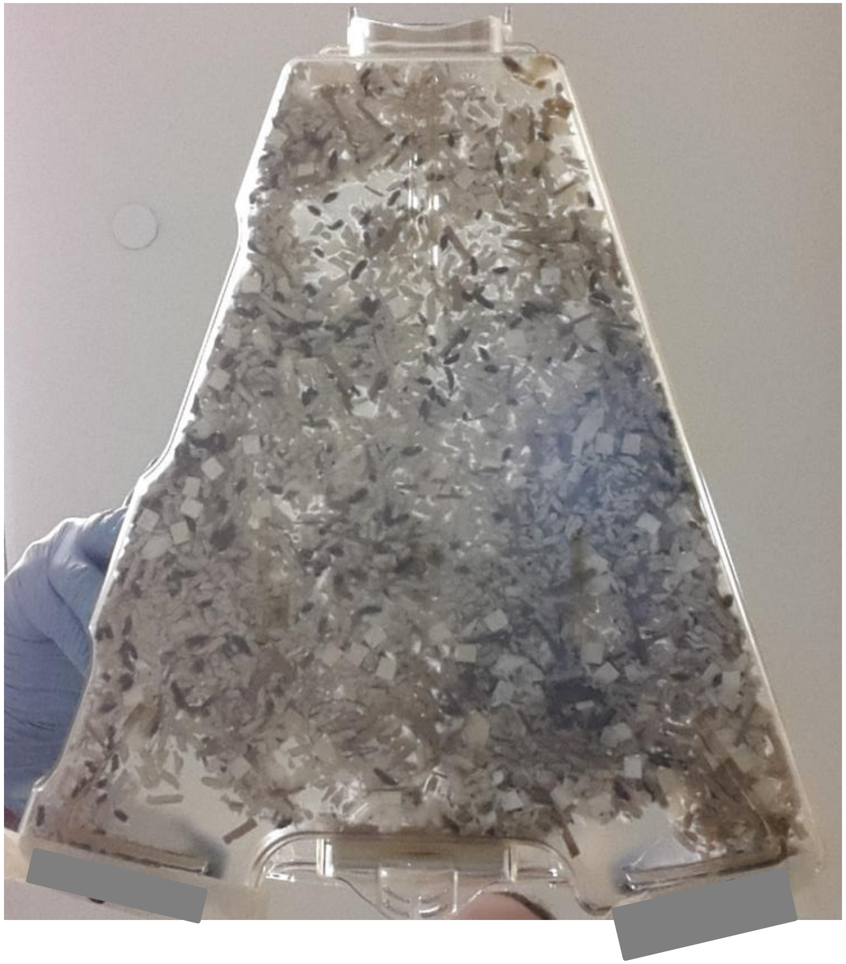

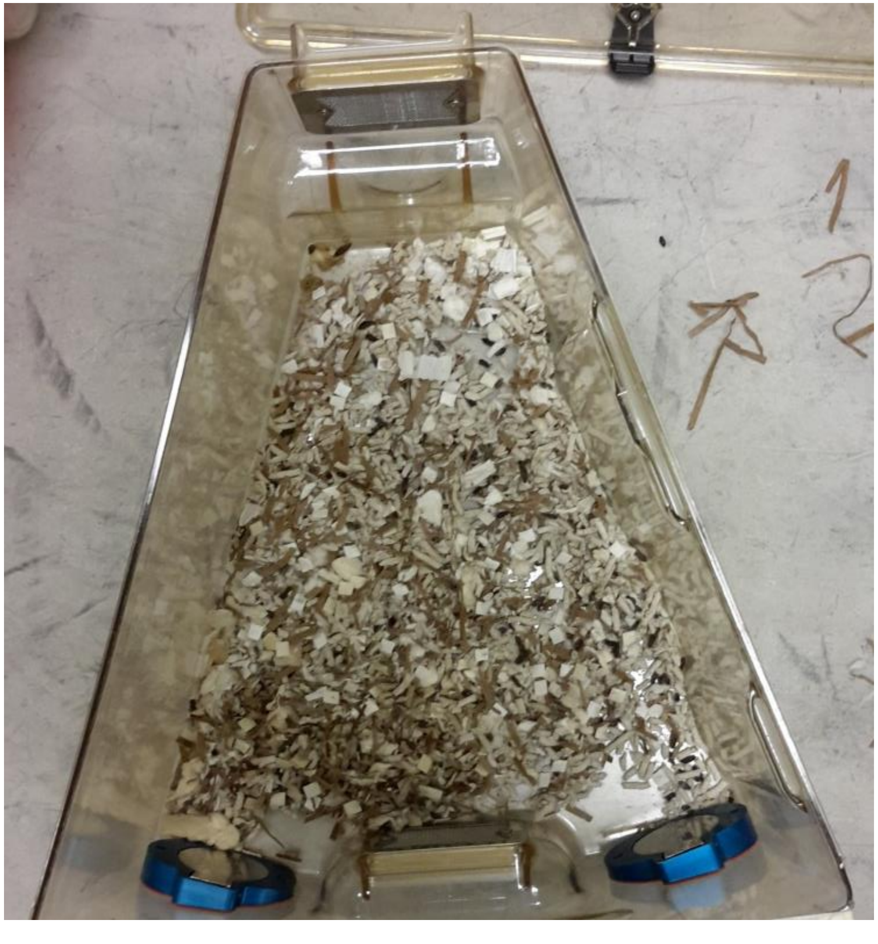

Aug 12 COMP 4 mid

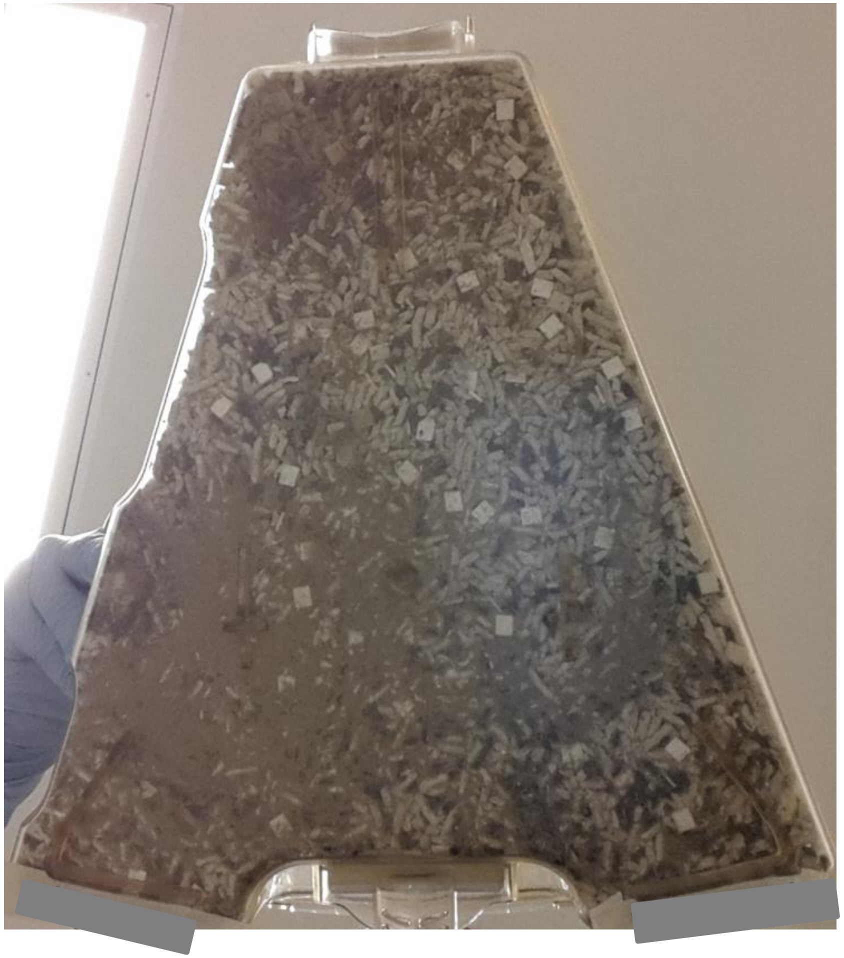

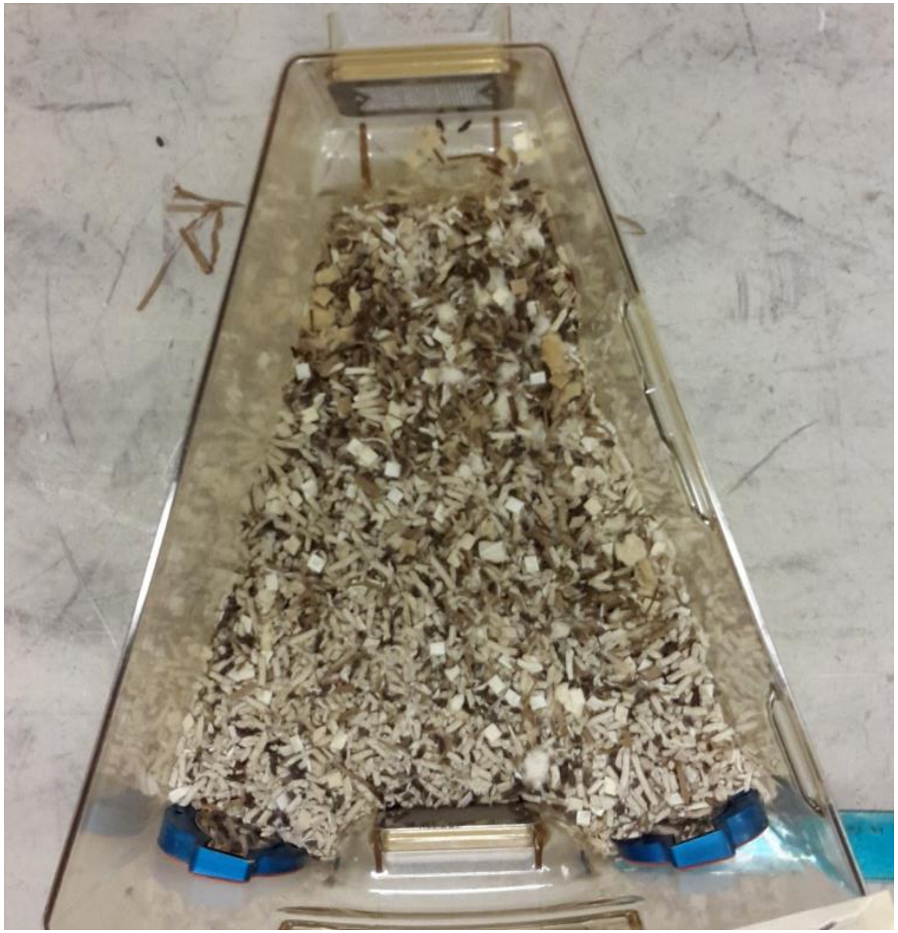

Aug 12 STD 4

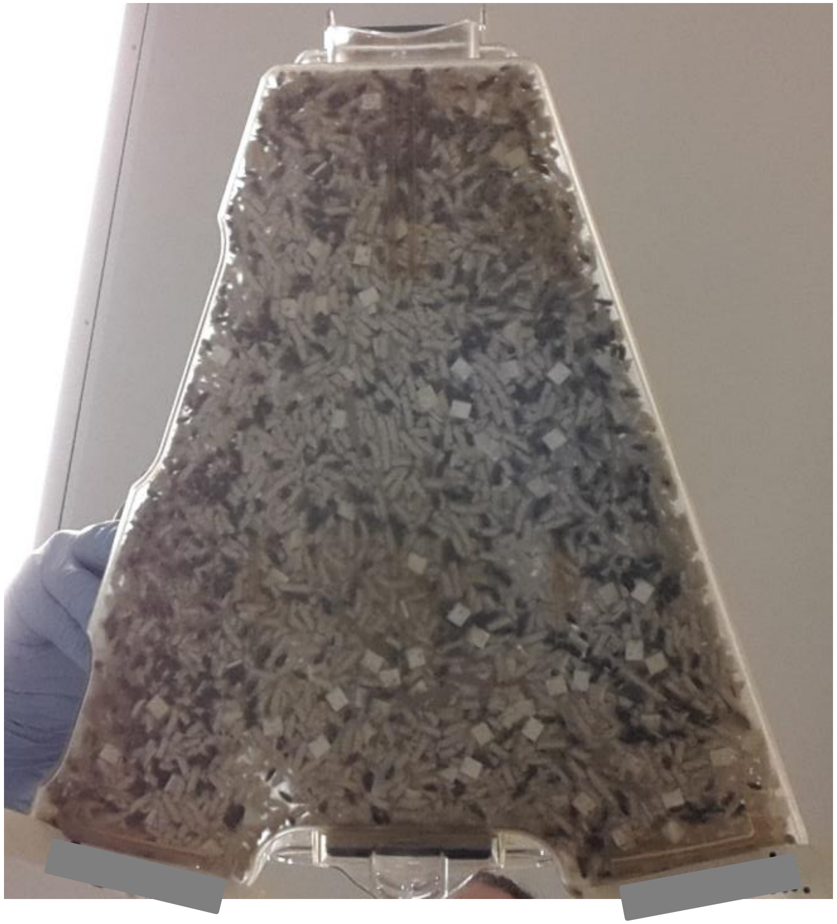

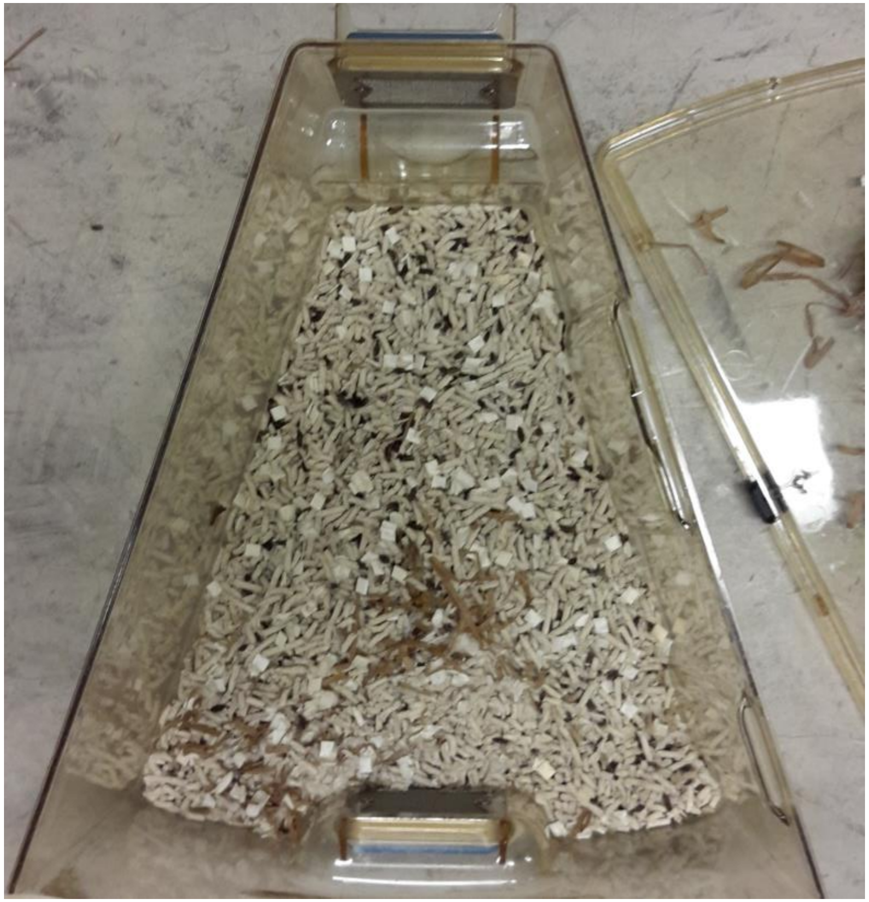

Aug 13 COMP 5 left

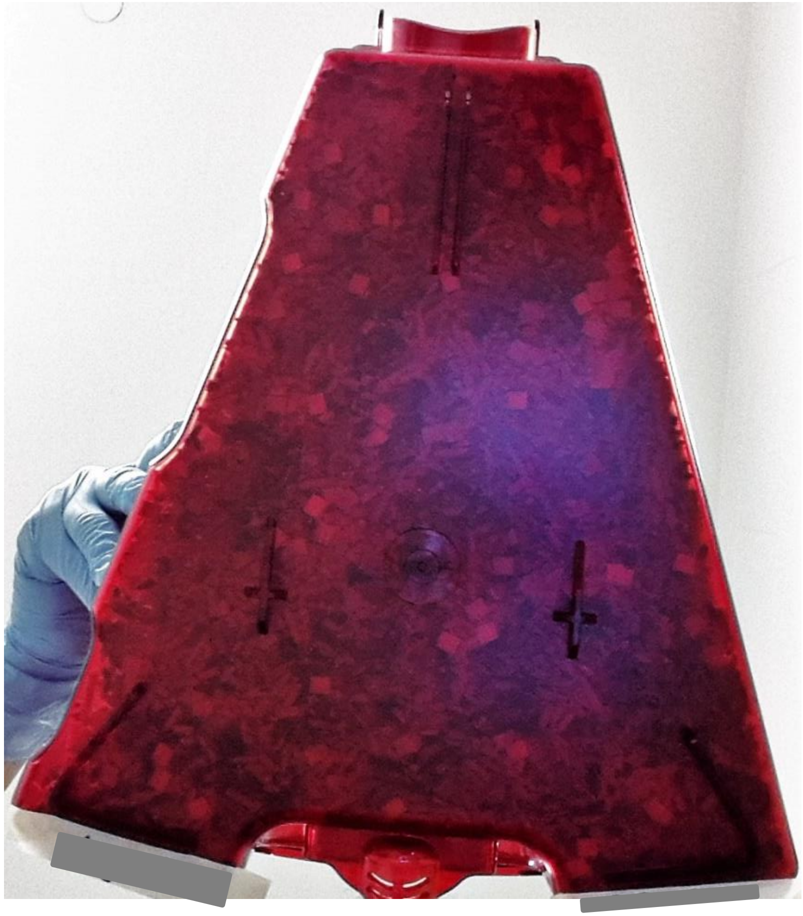

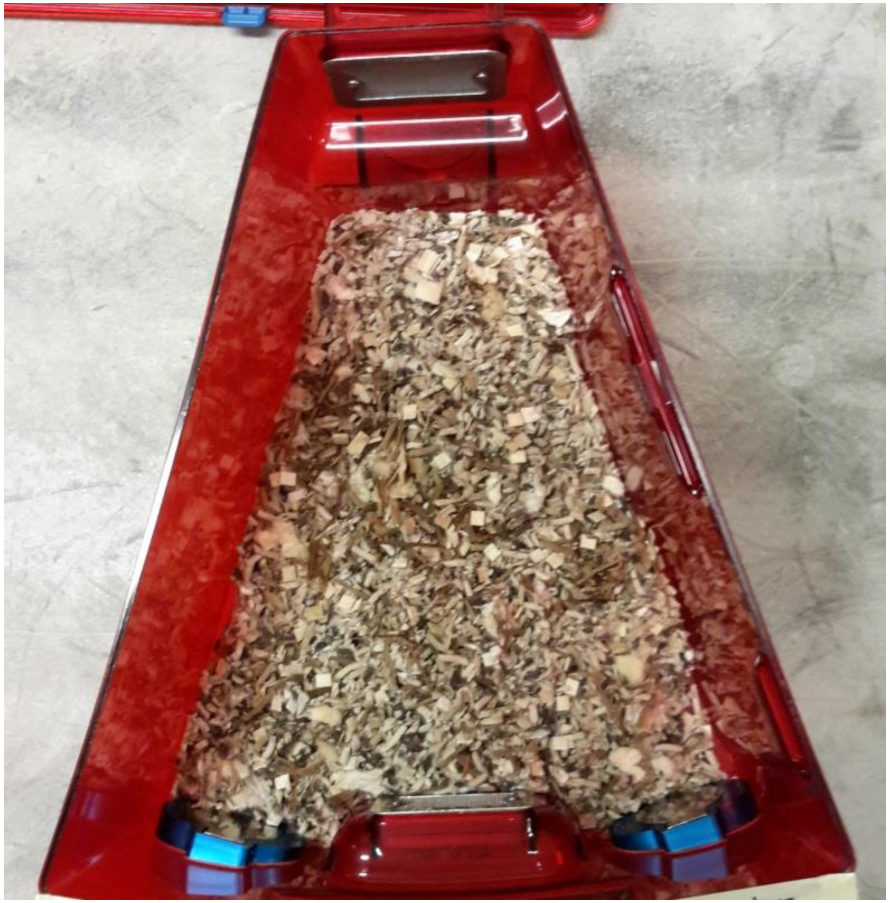

Aug 13 COMP 5 mid

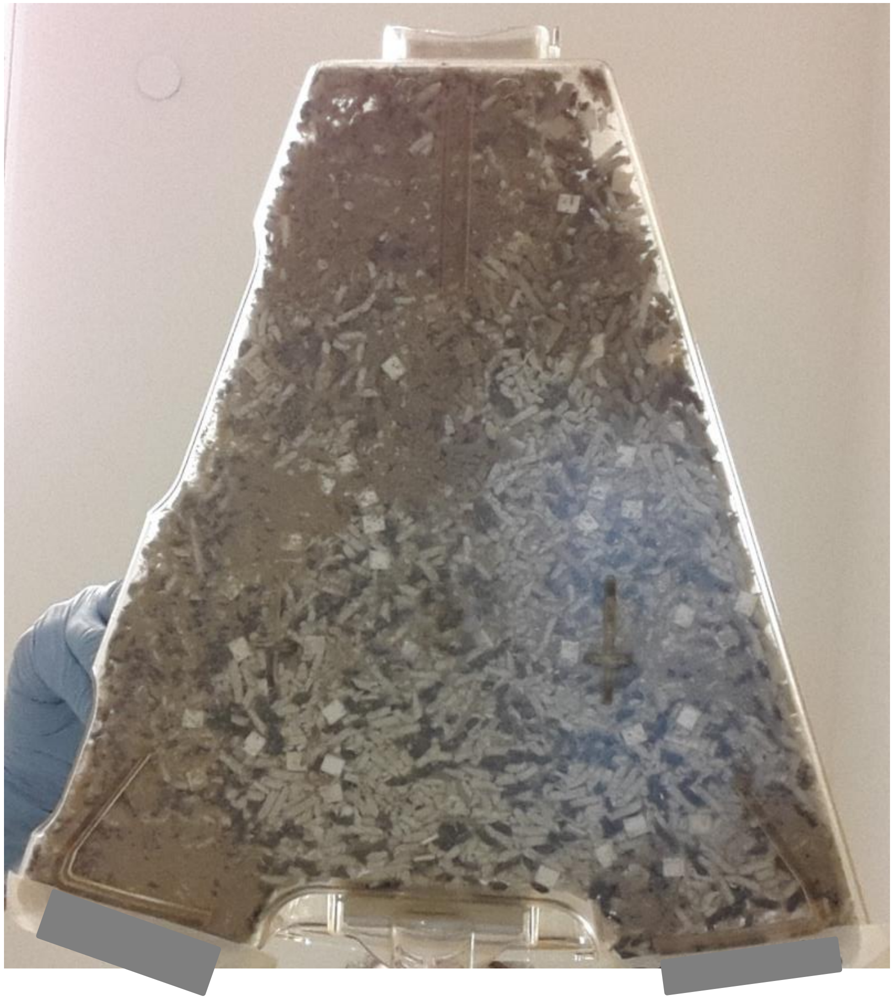

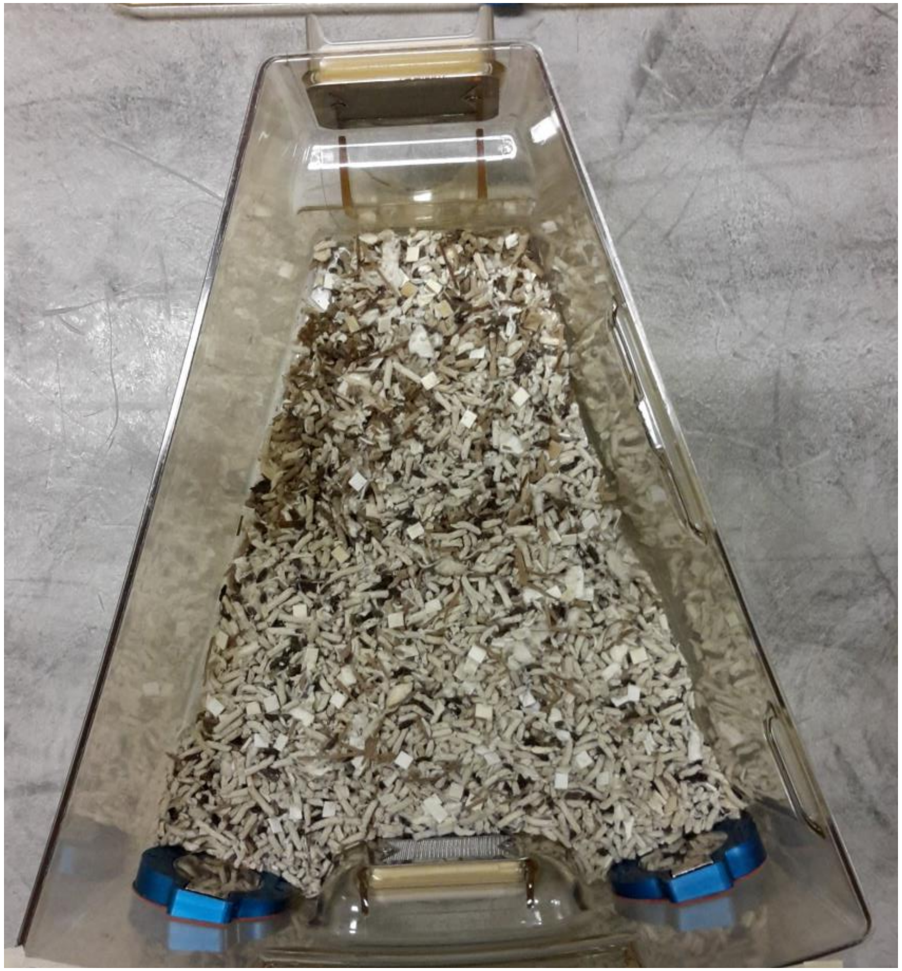

Aug 13 COMP 5 right

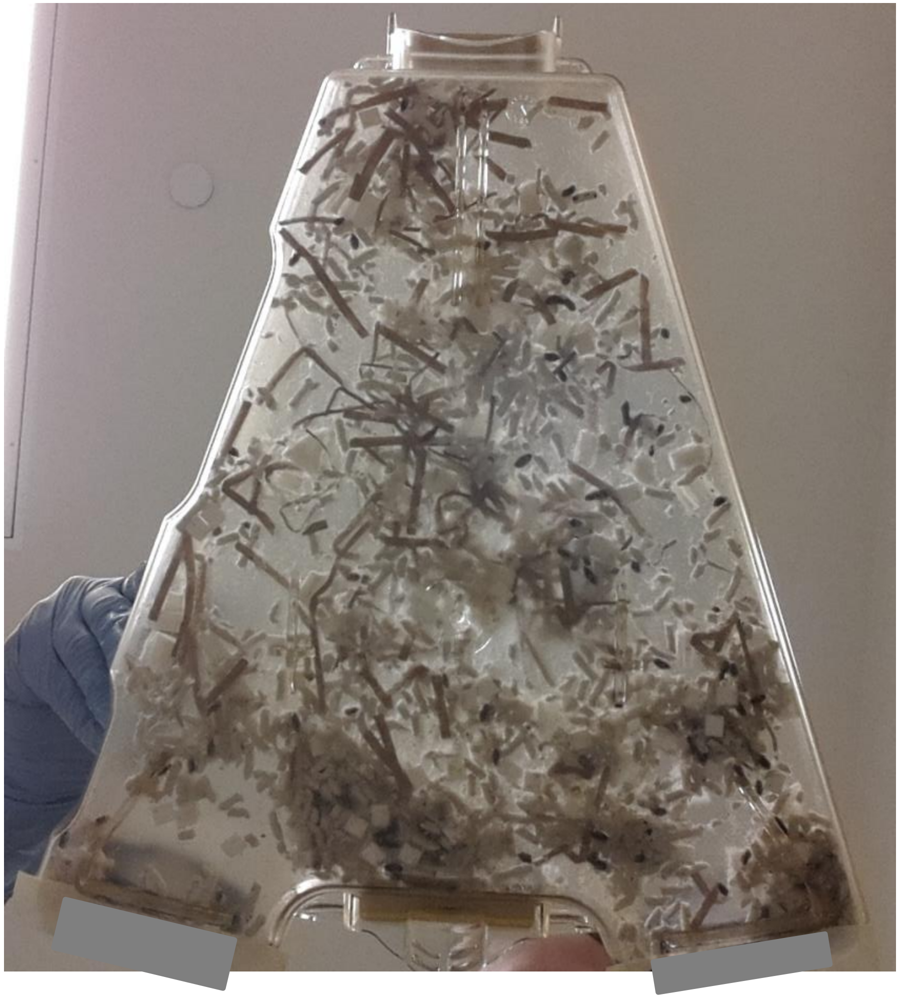

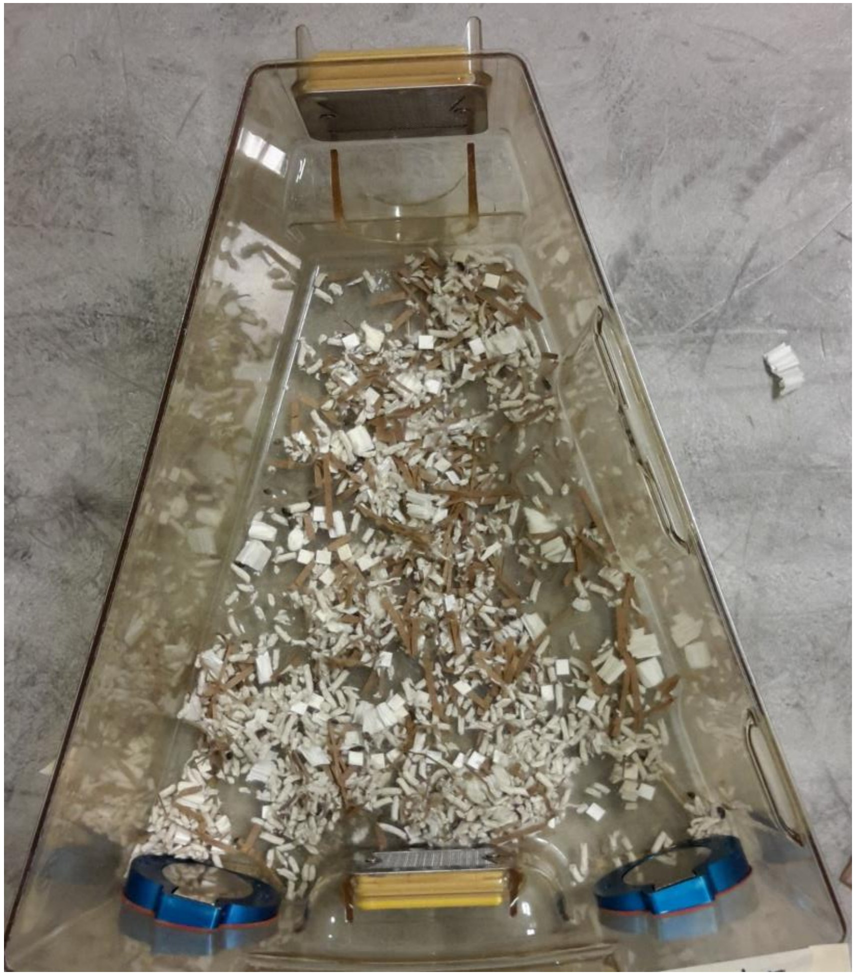

Aug 13 STD 5

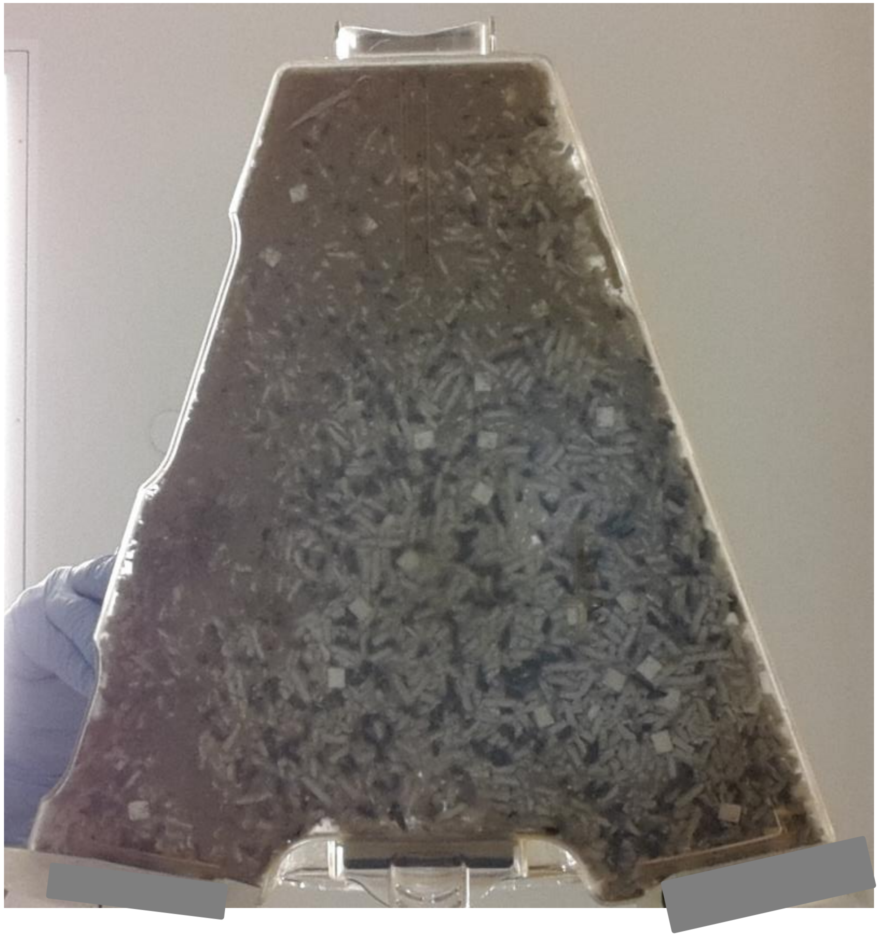

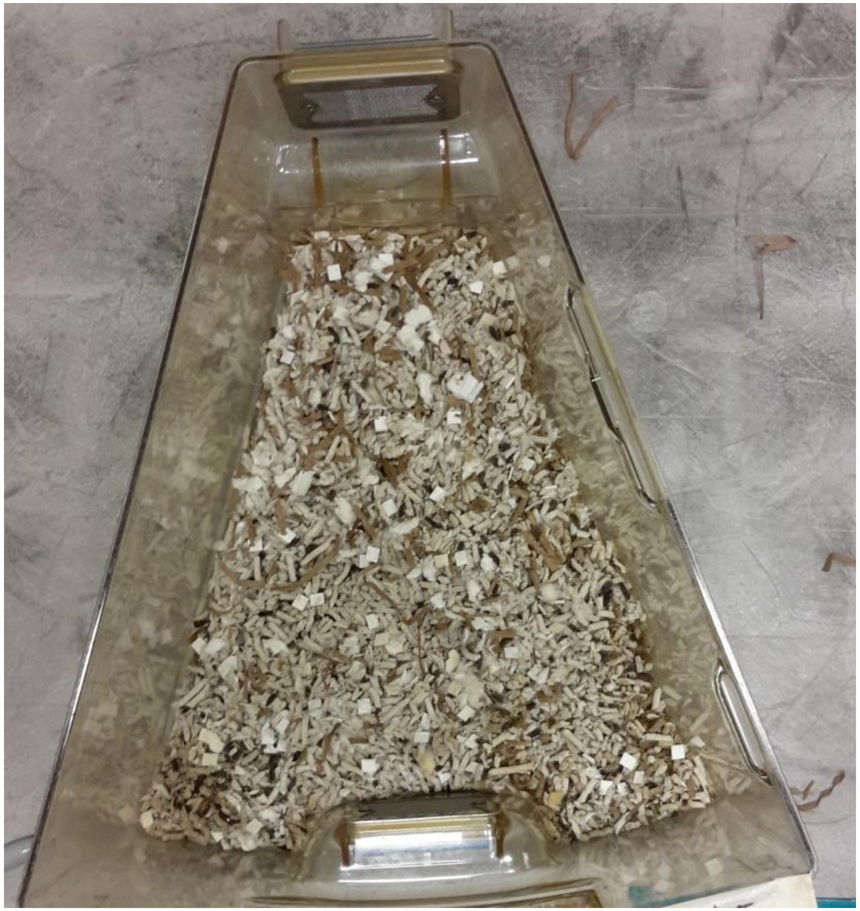

Aug 18 COMP 1 right

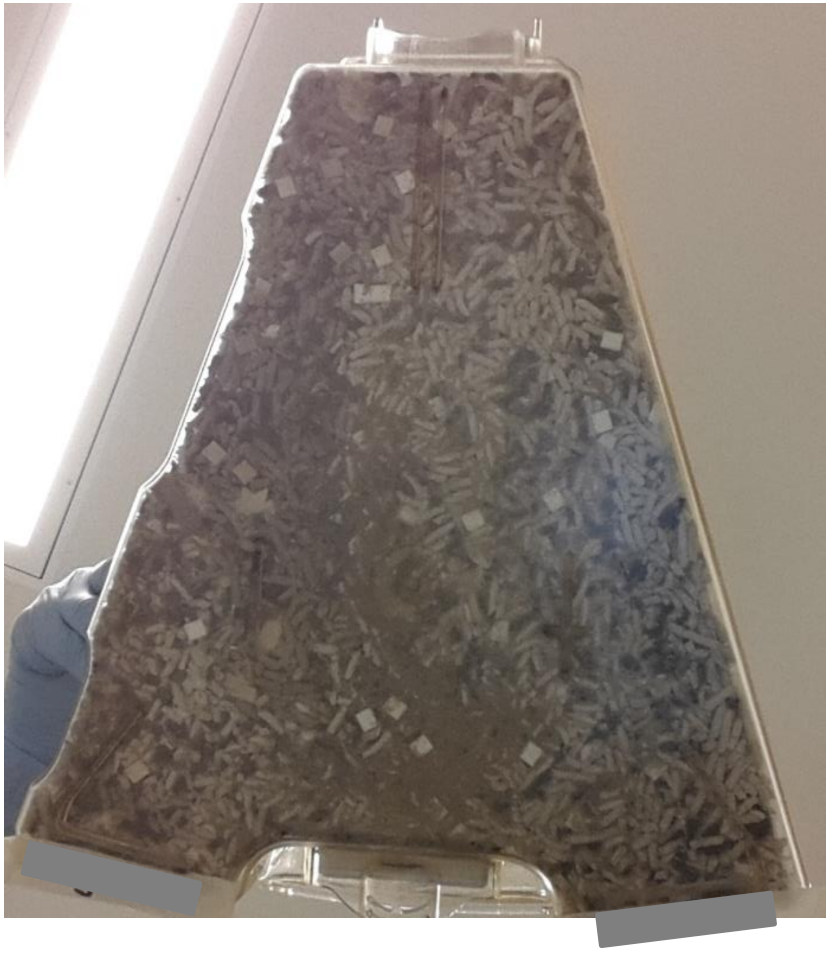

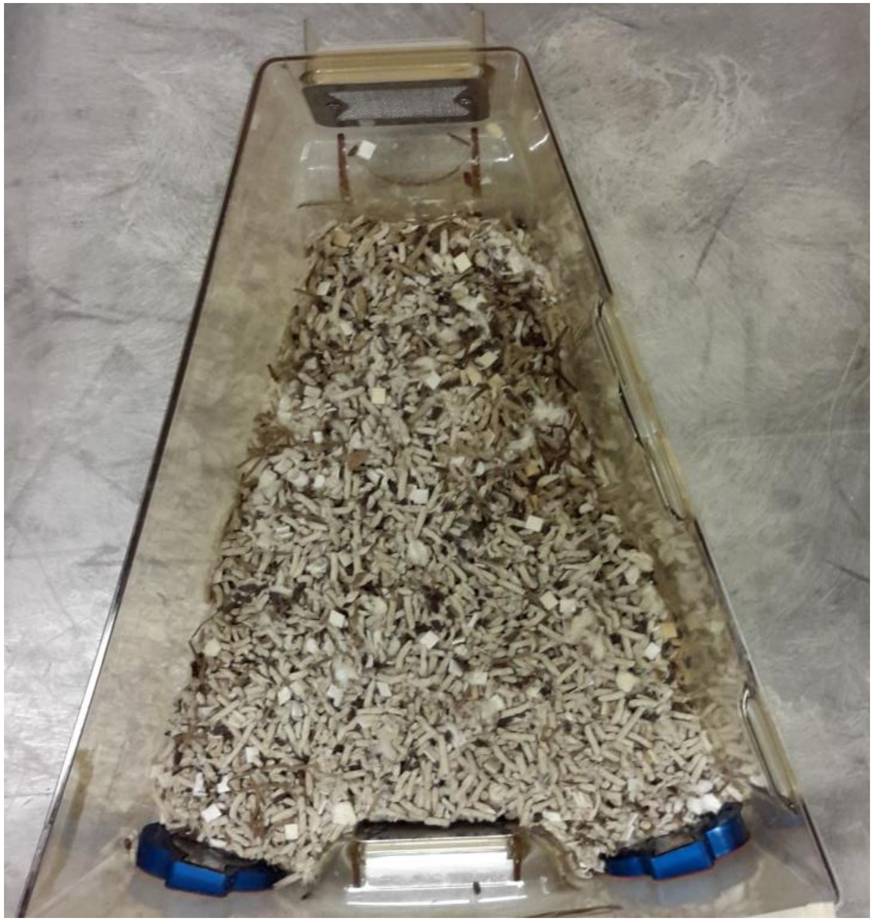

Aug 18 COMP 1 mid

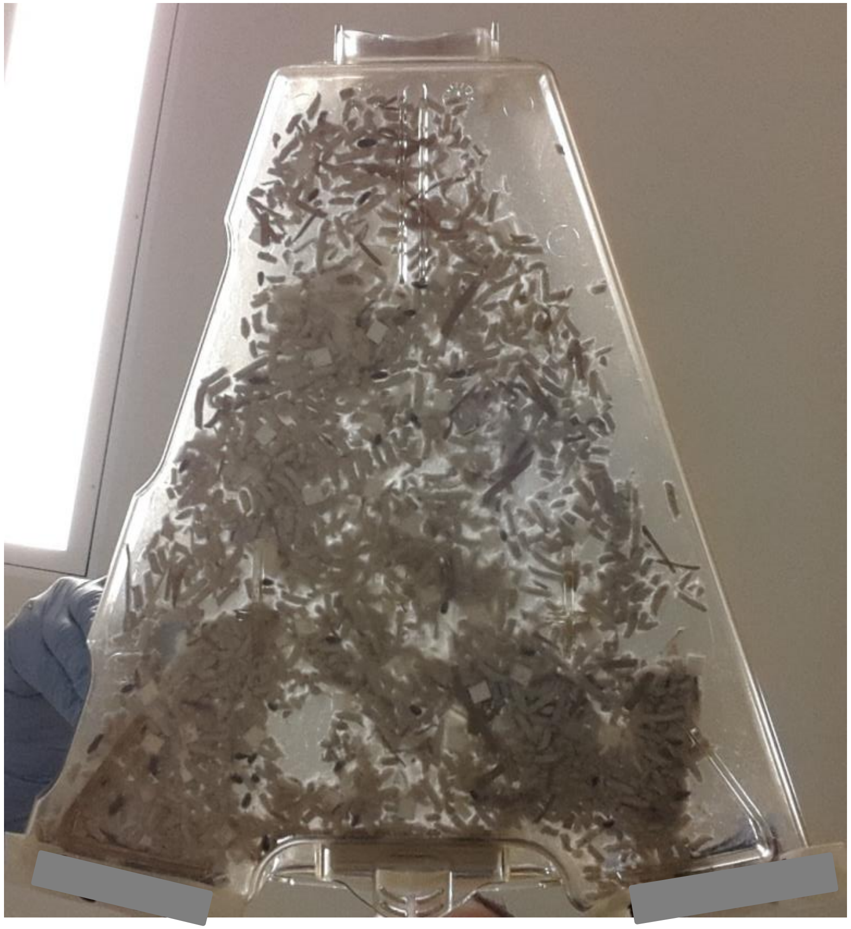

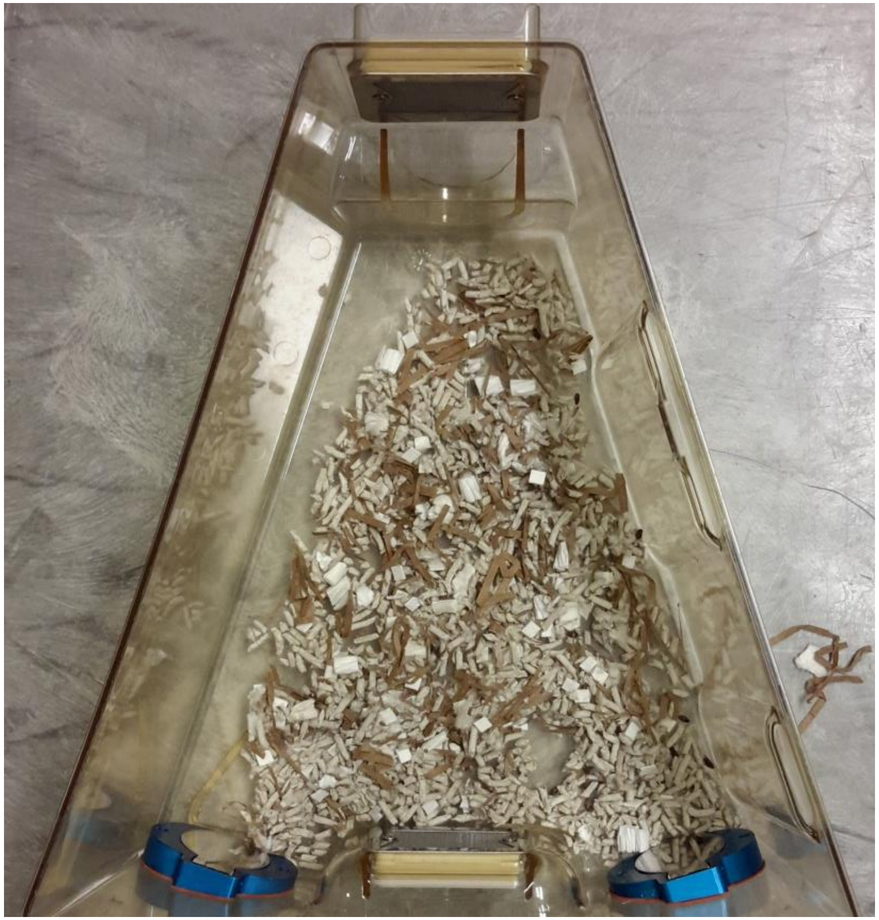

Aug 18 COMP 1 left

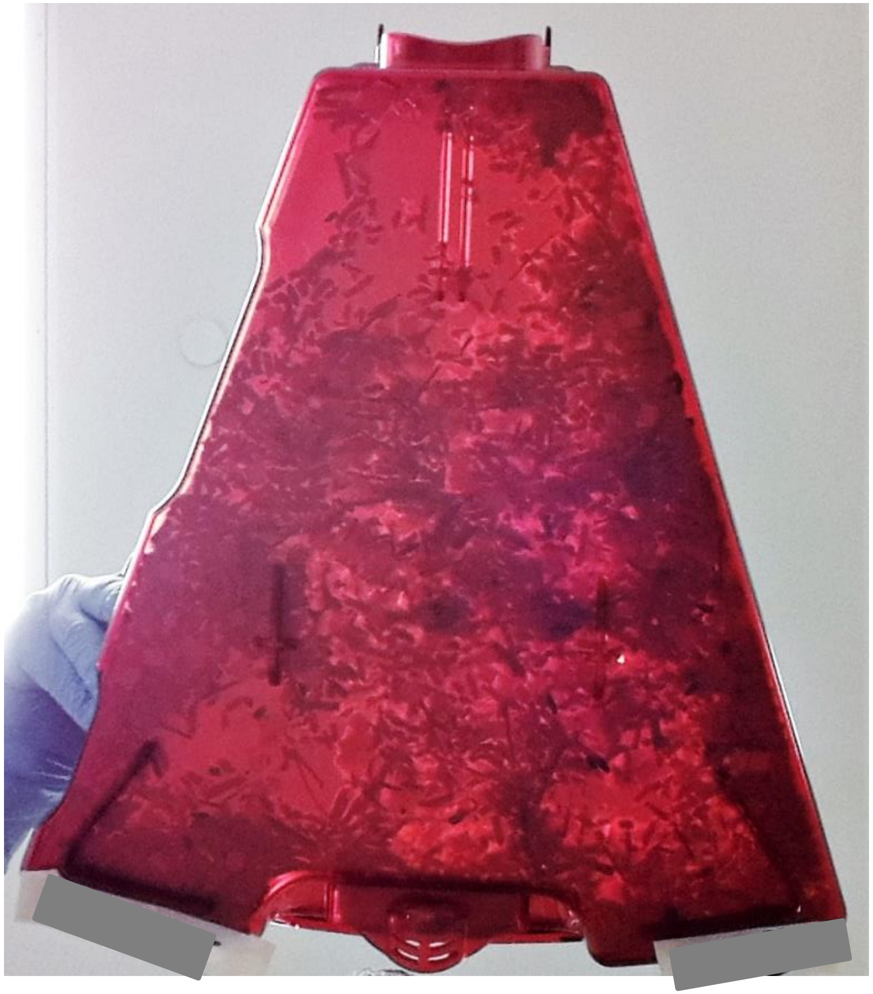

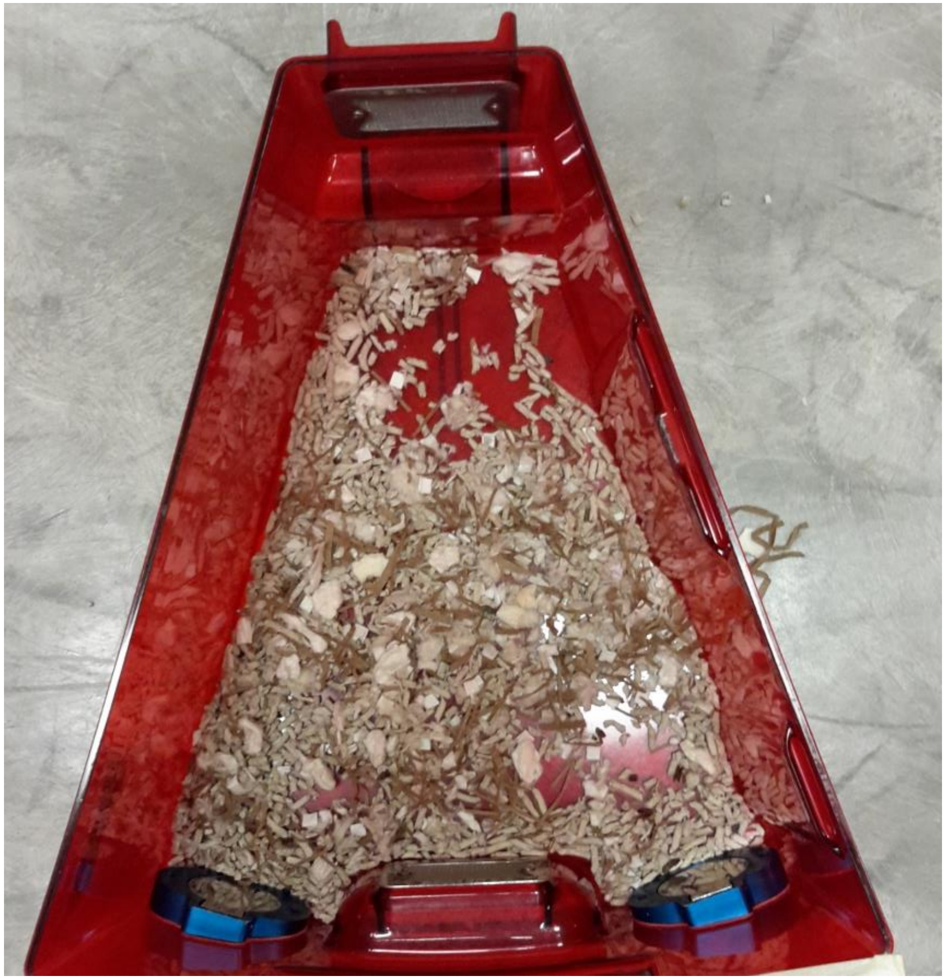

Aug 18 STD 1

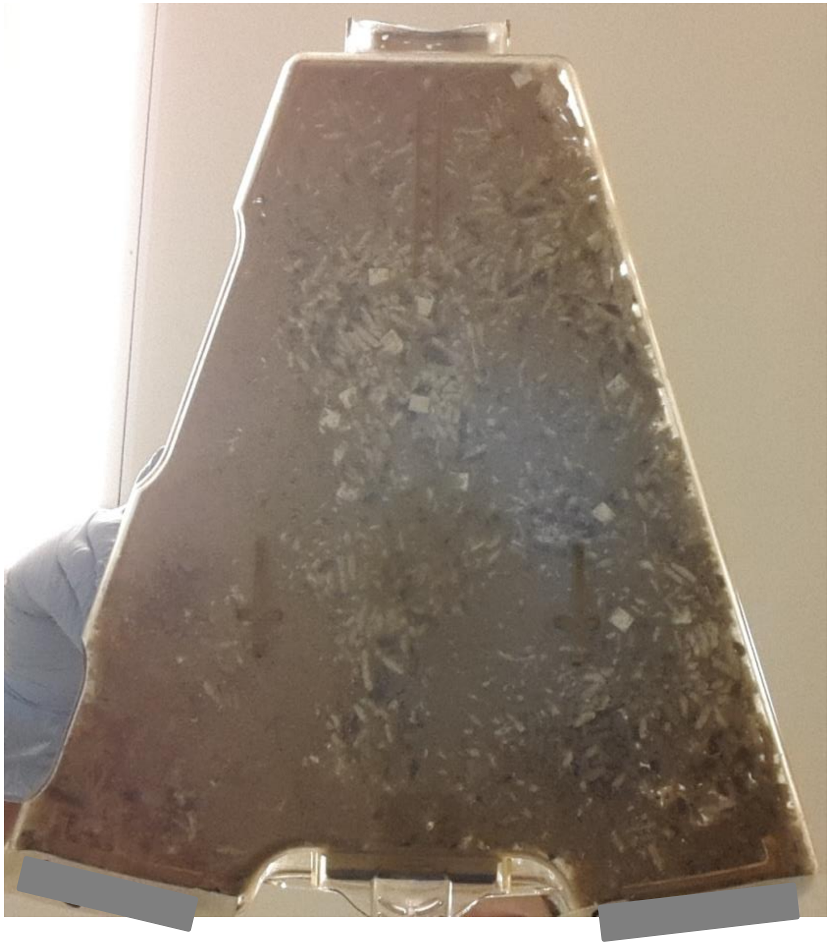

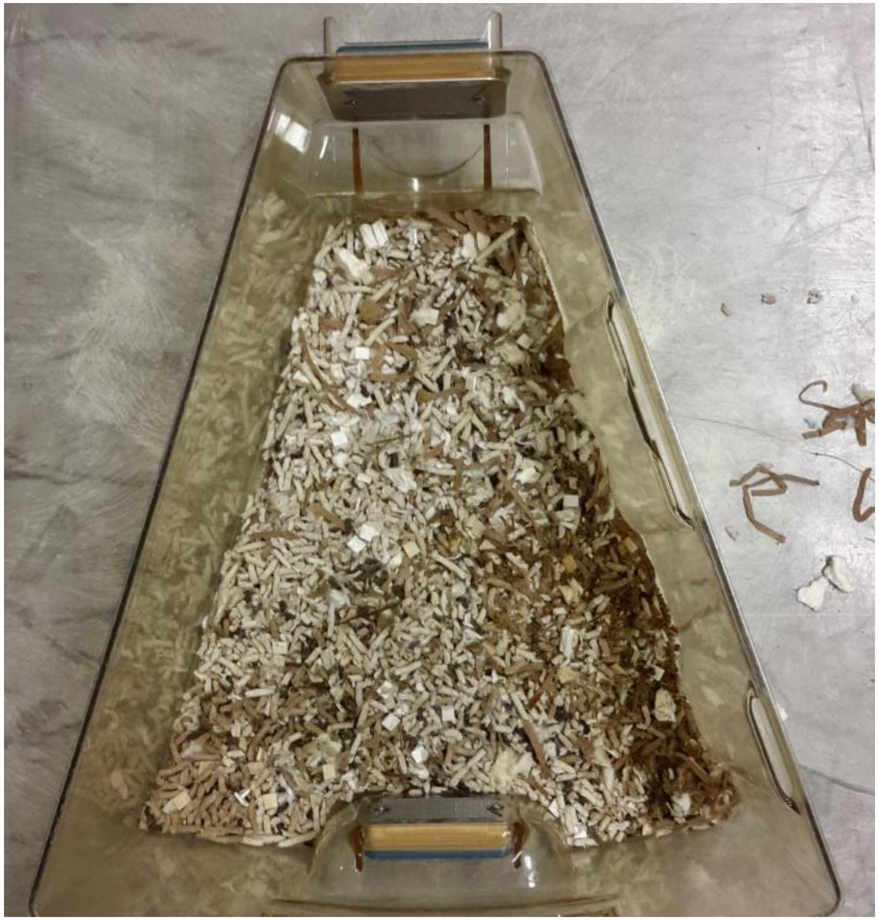

Aug 18 COMP 2 right

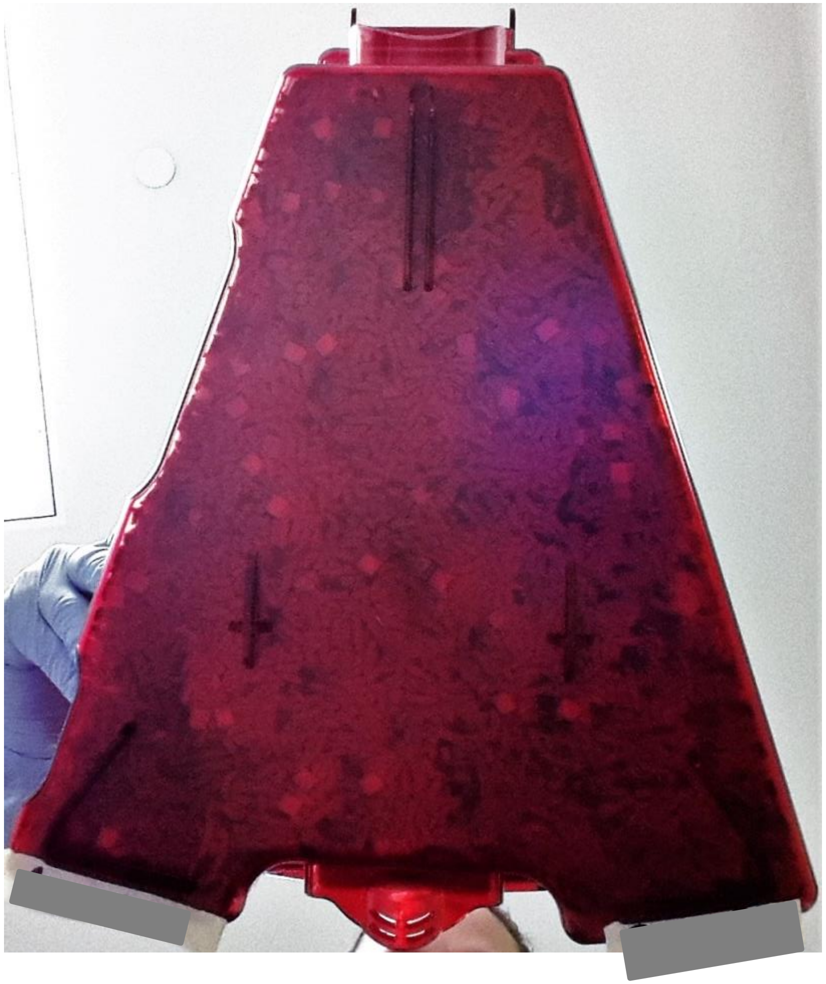

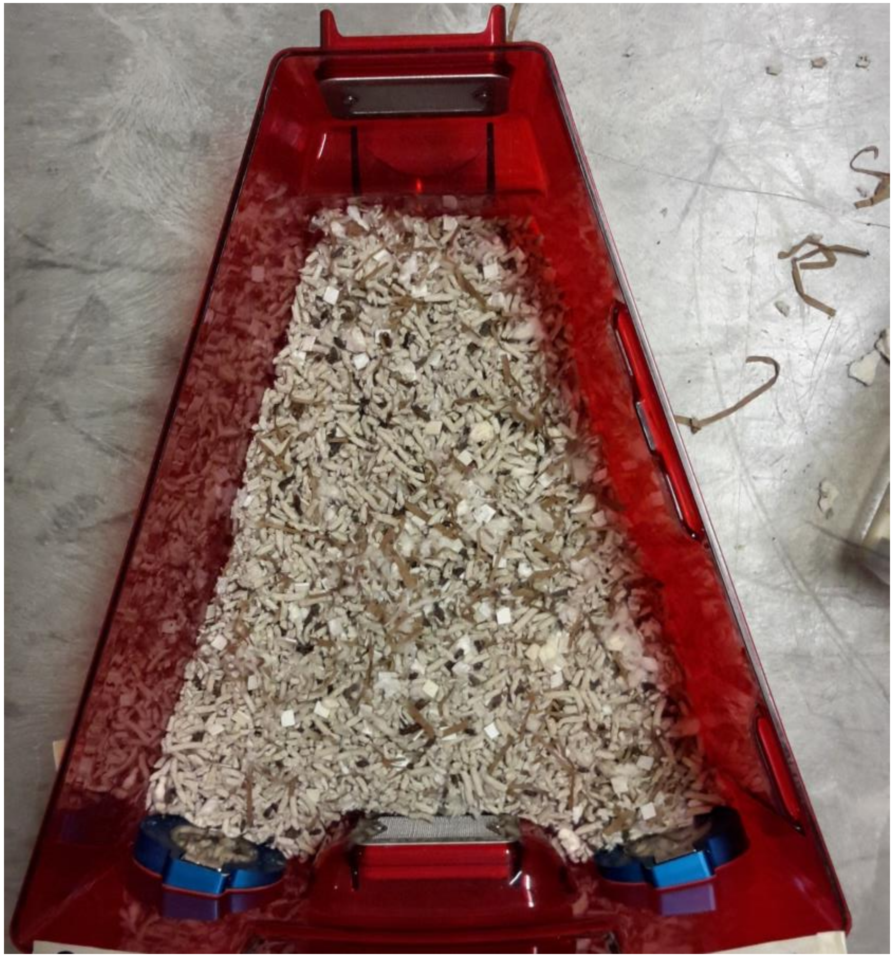

Aug 18 COMP 2 mid

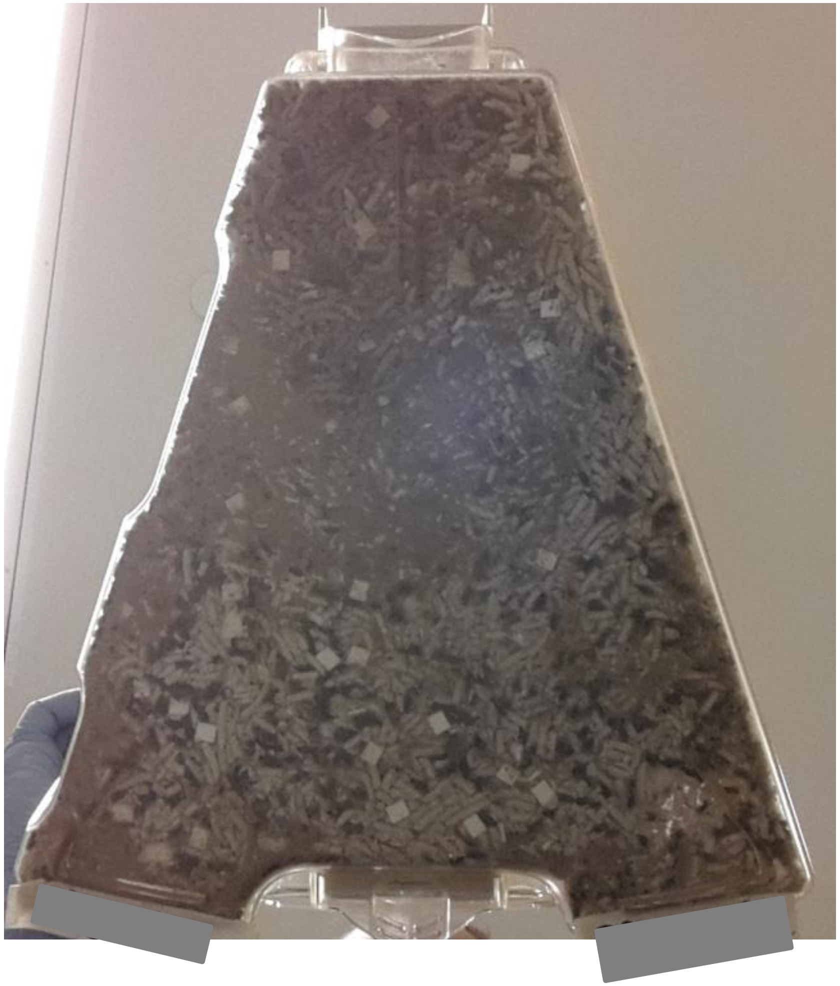

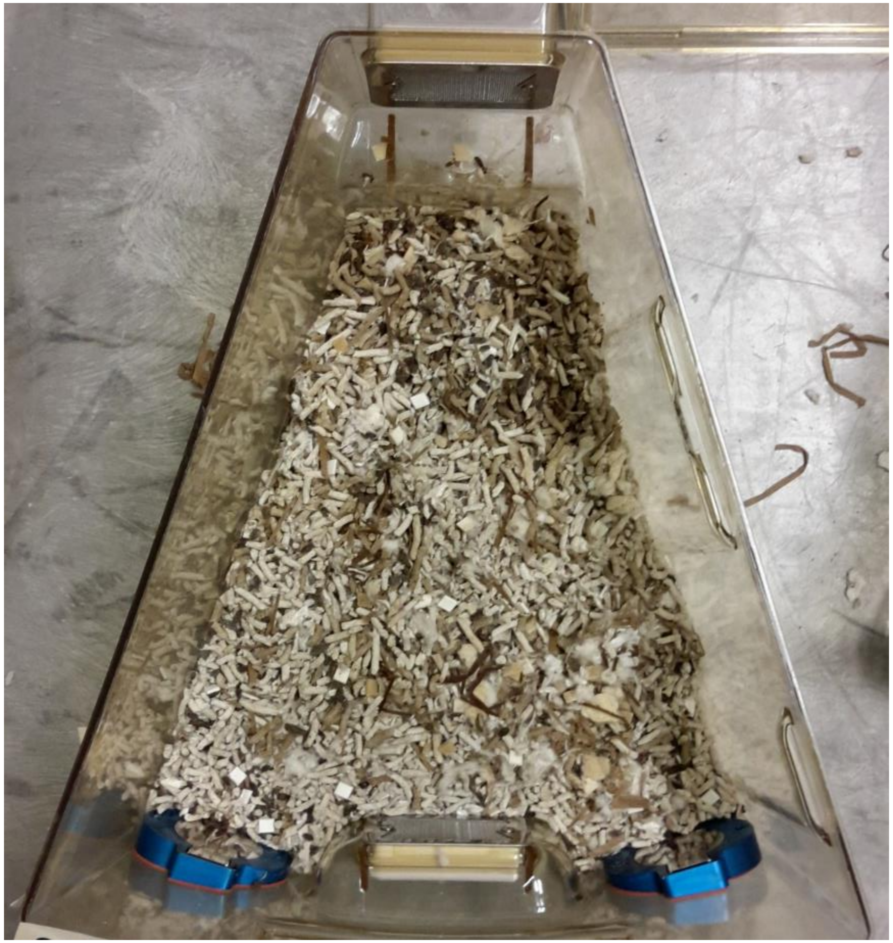

Aug 18 COMP 2 left

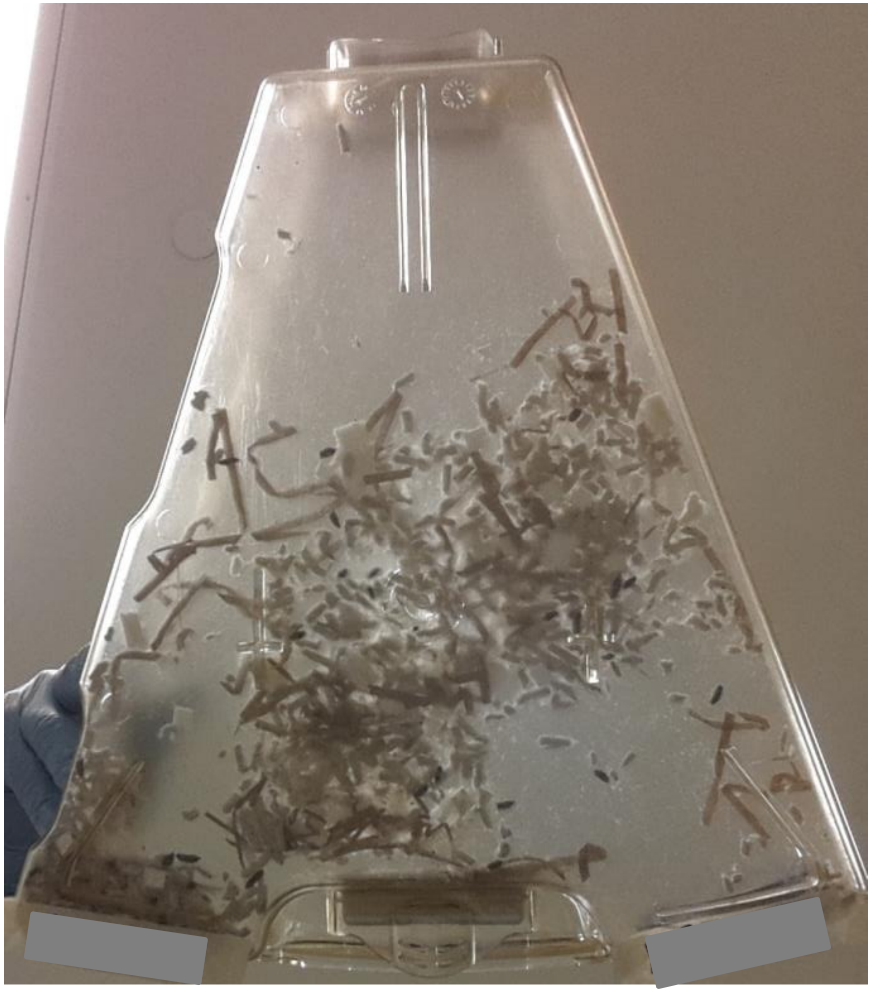

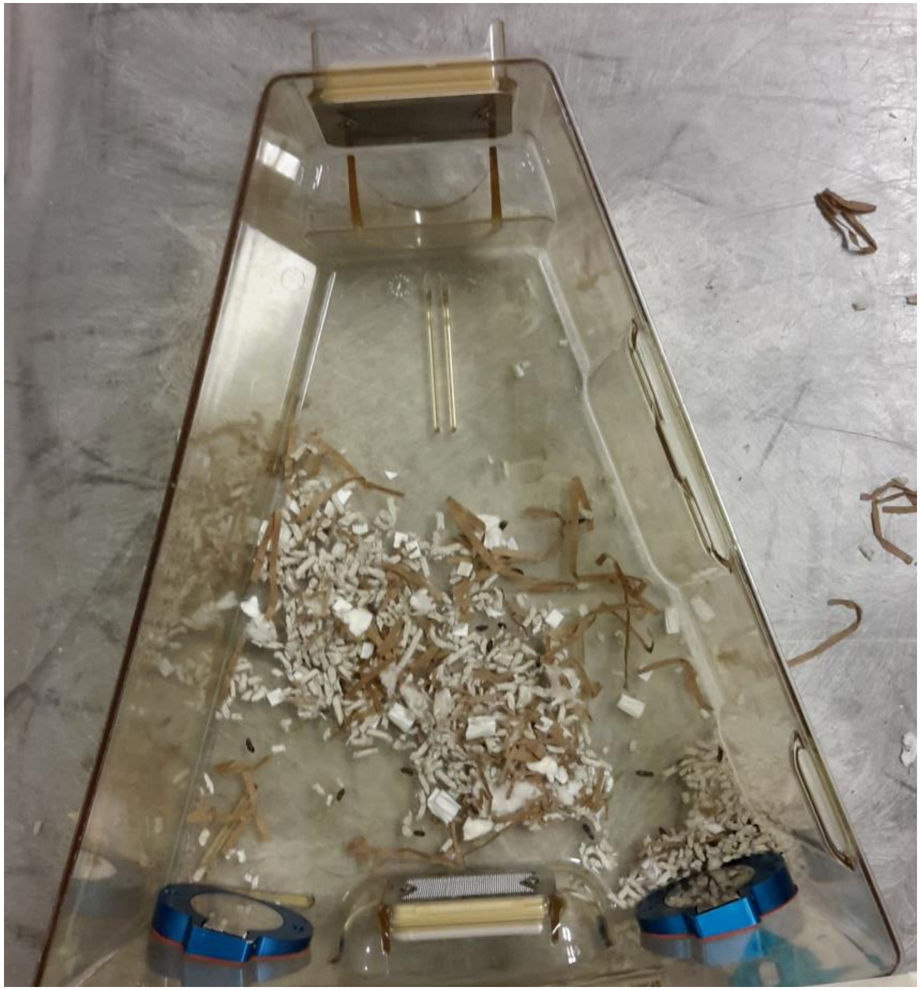

Aug 18 STD 2

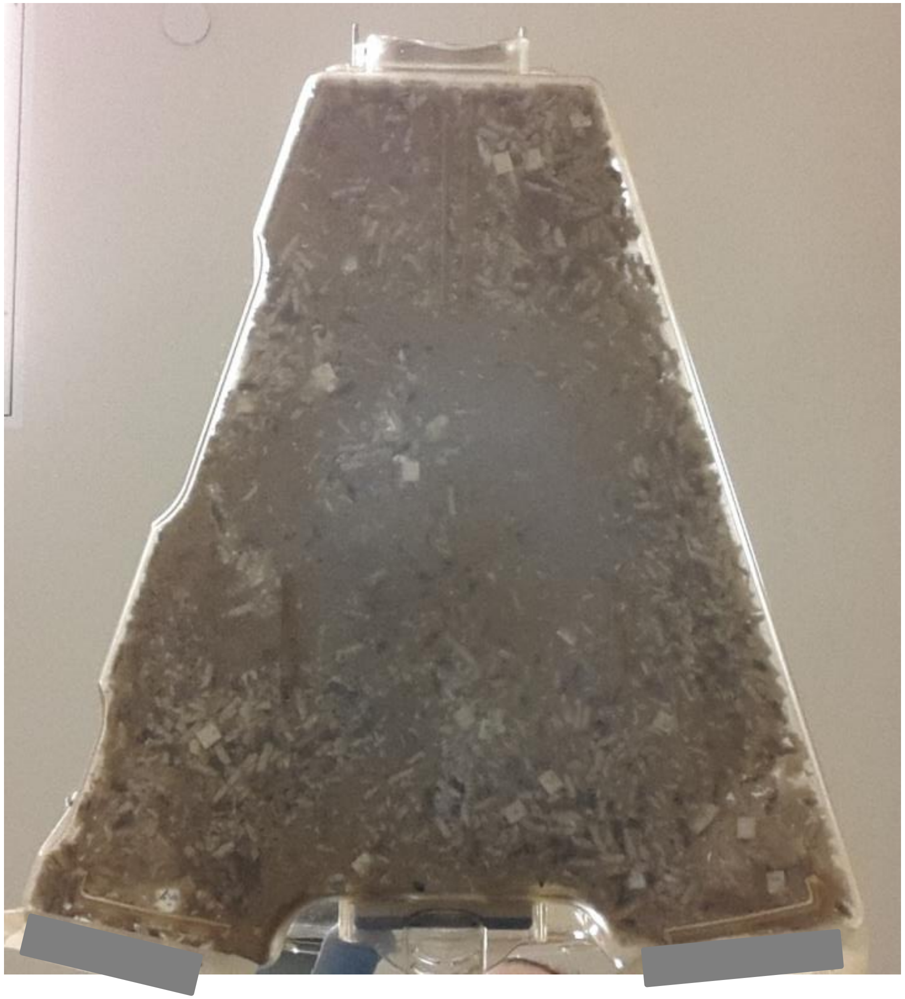

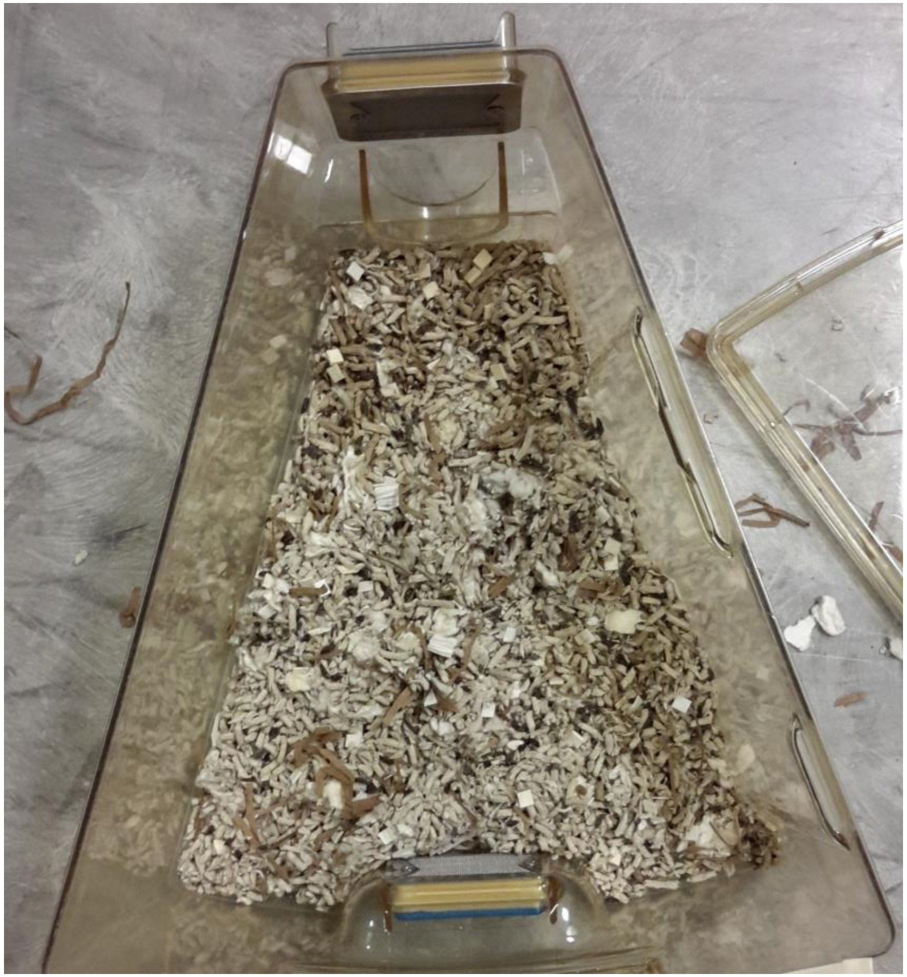

Aug 19 COMP 3 right

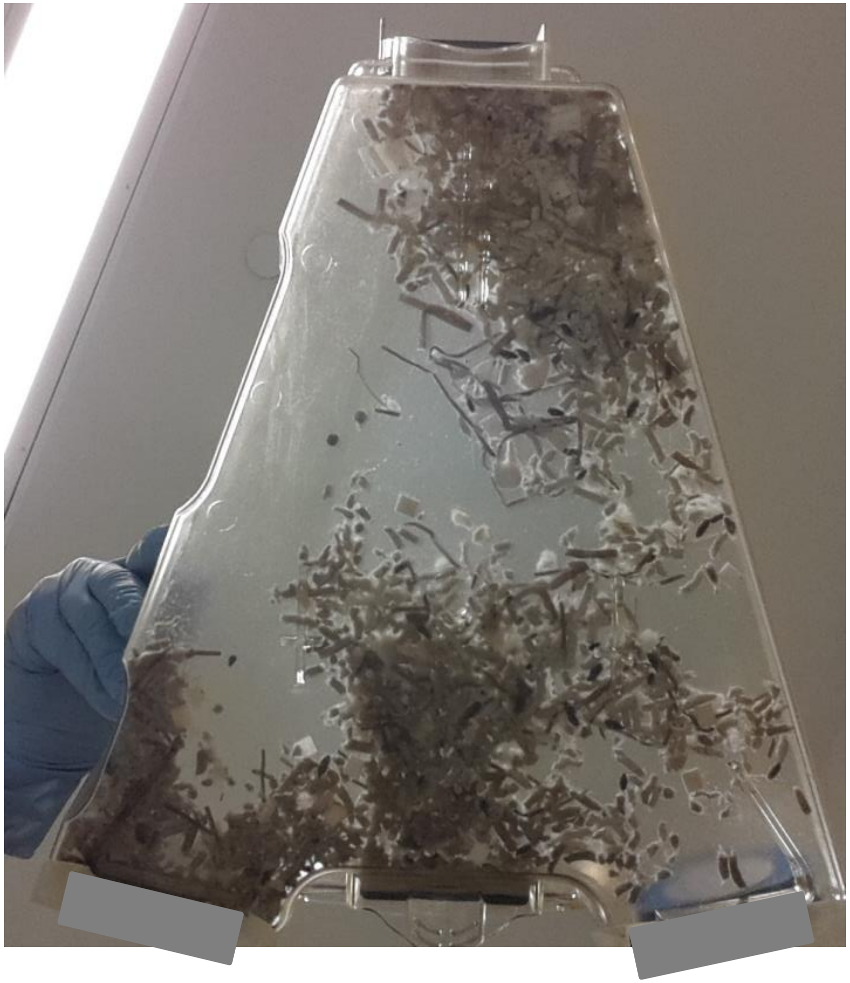

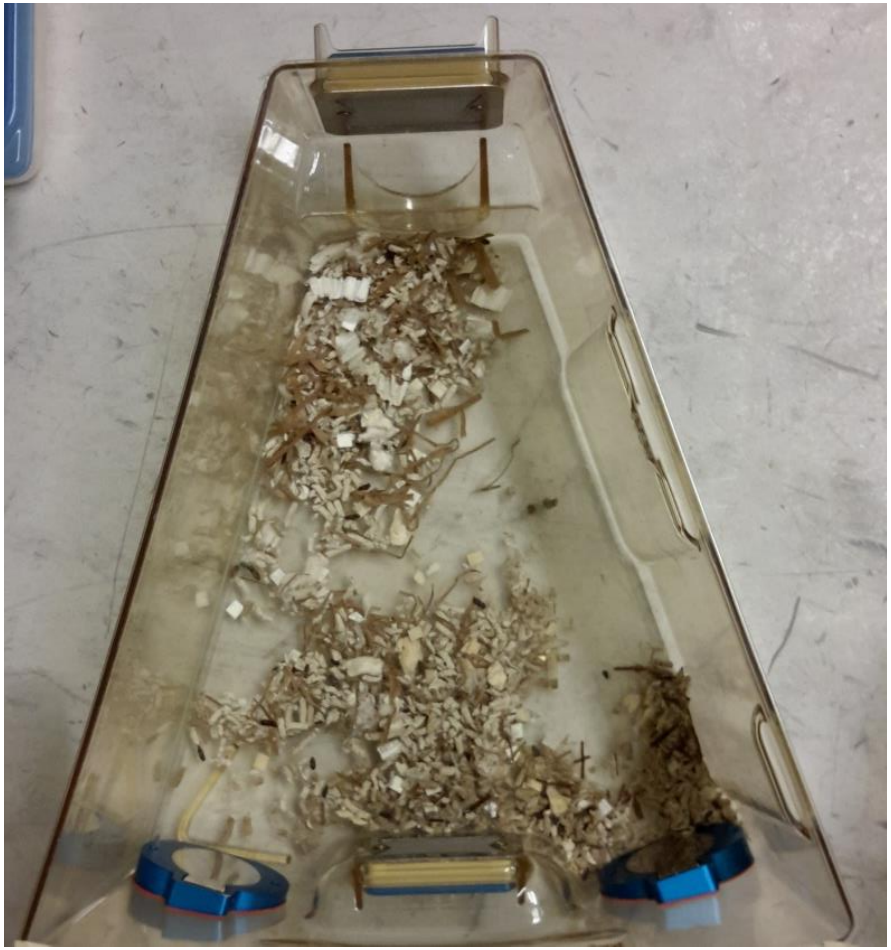

Aug 19 COMP 3 mid

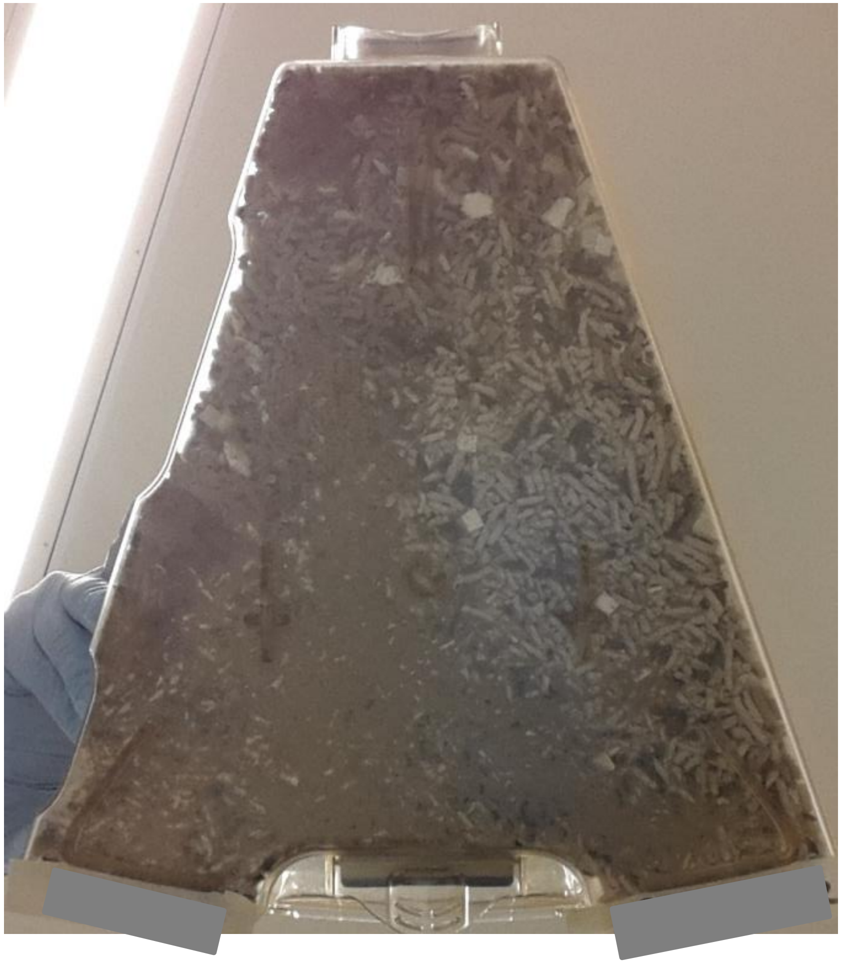

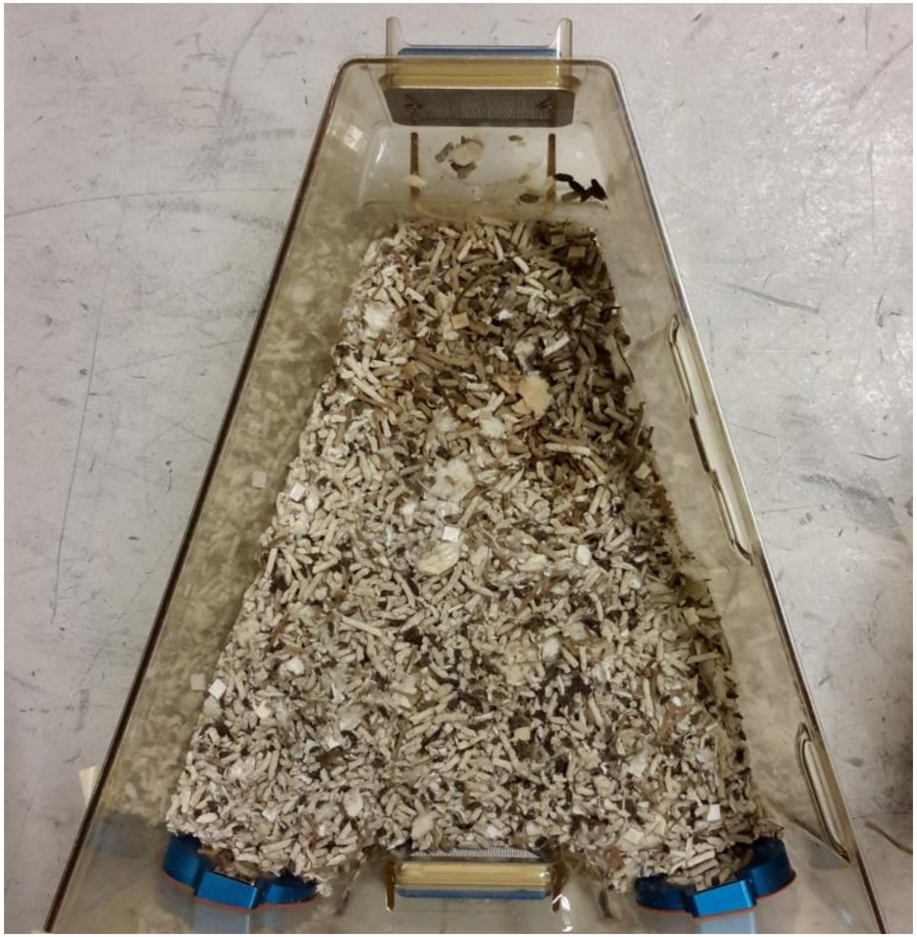

Aug 19 COMP 3 left

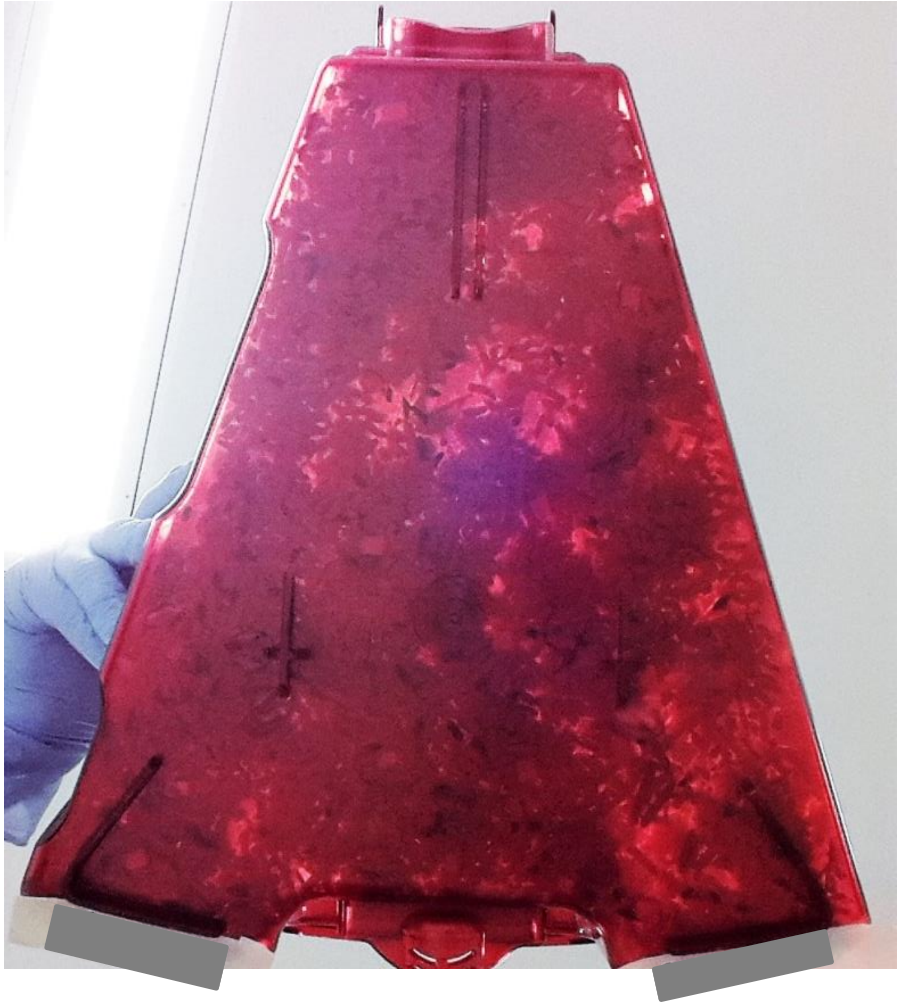

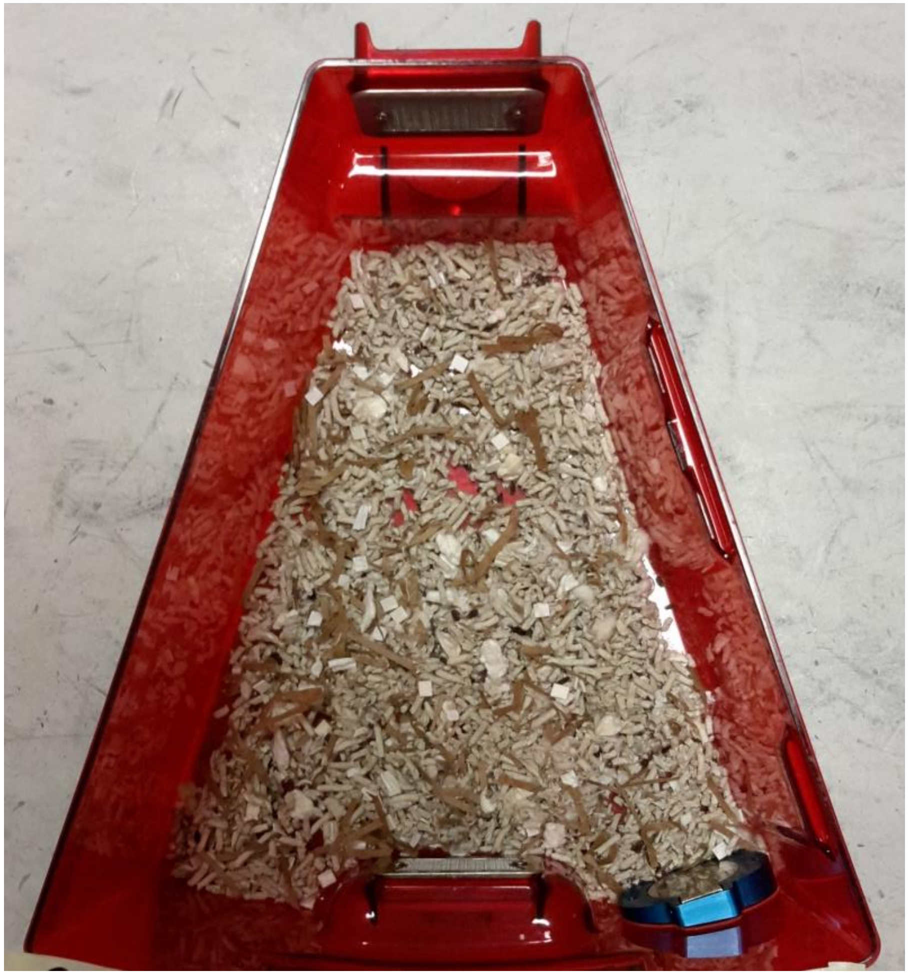

Aug 19 STD 3

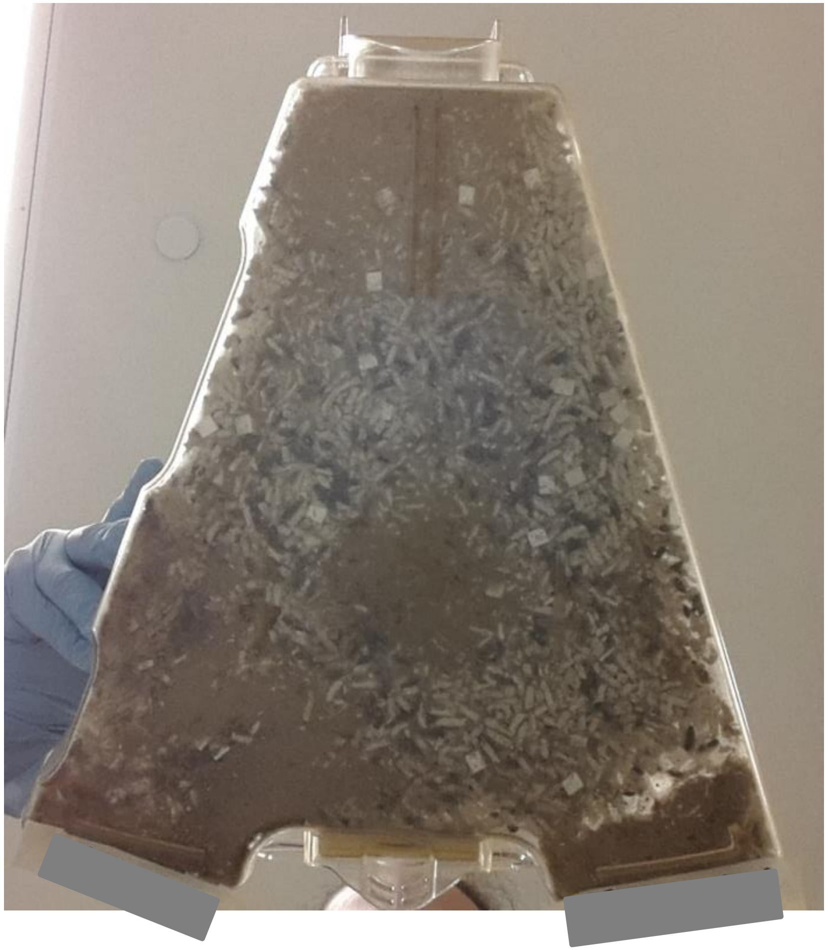

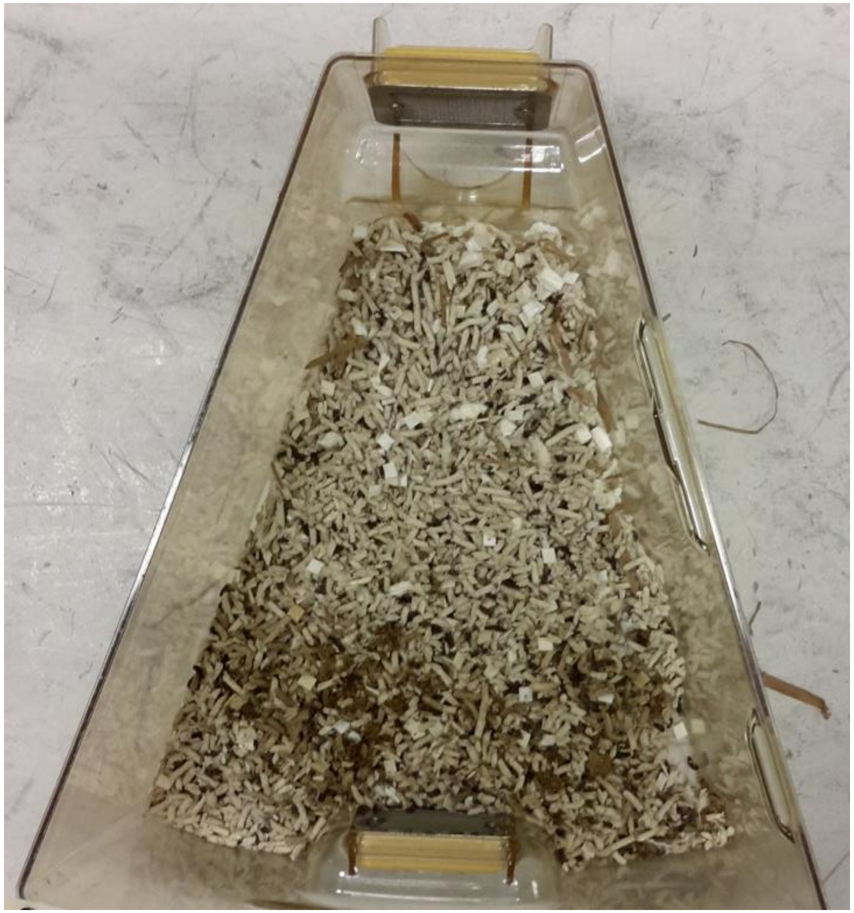

Aug 19 COMP 4 right

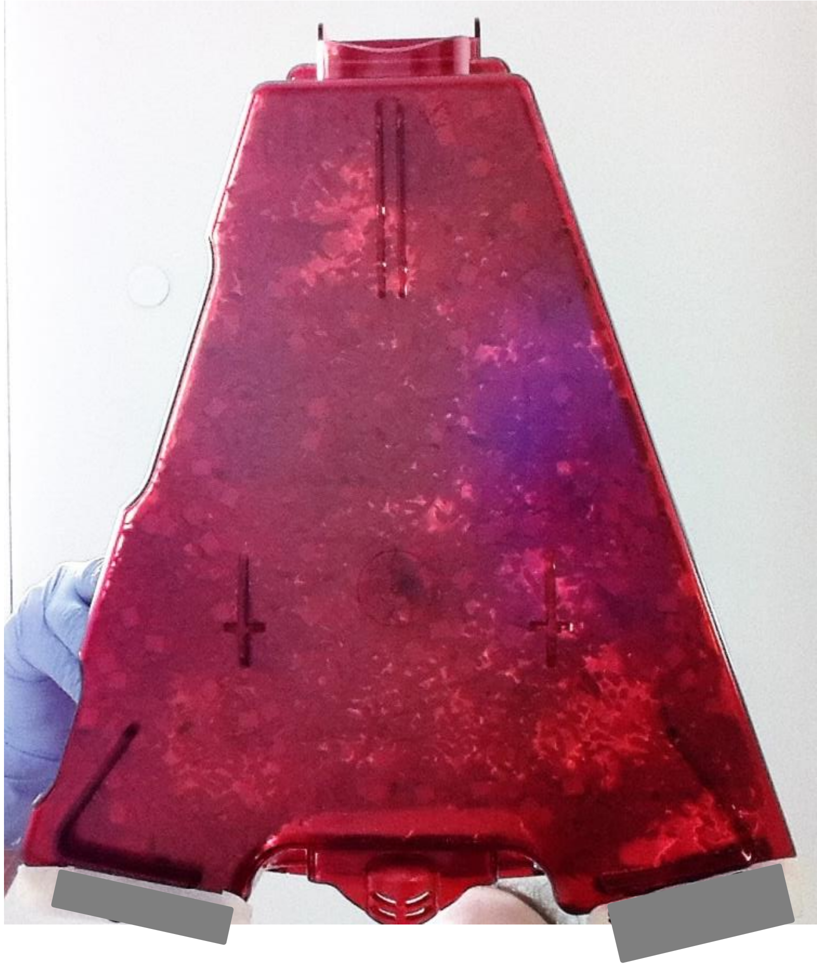

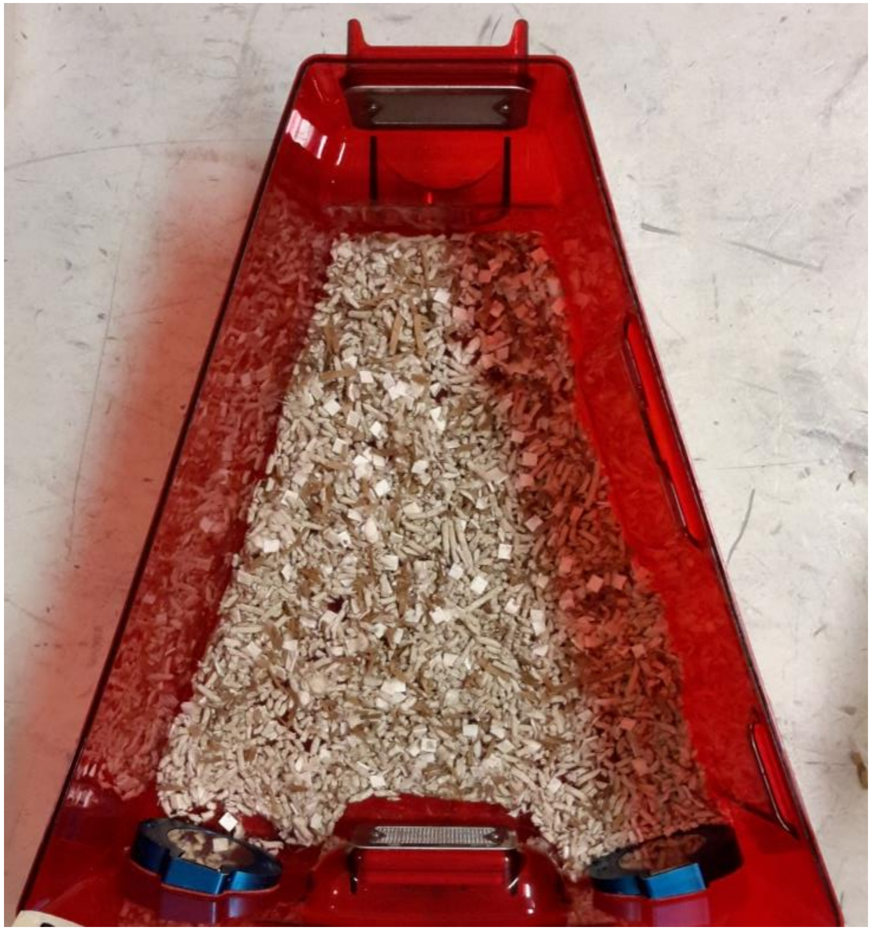

Aug 19 COMP 4 mid

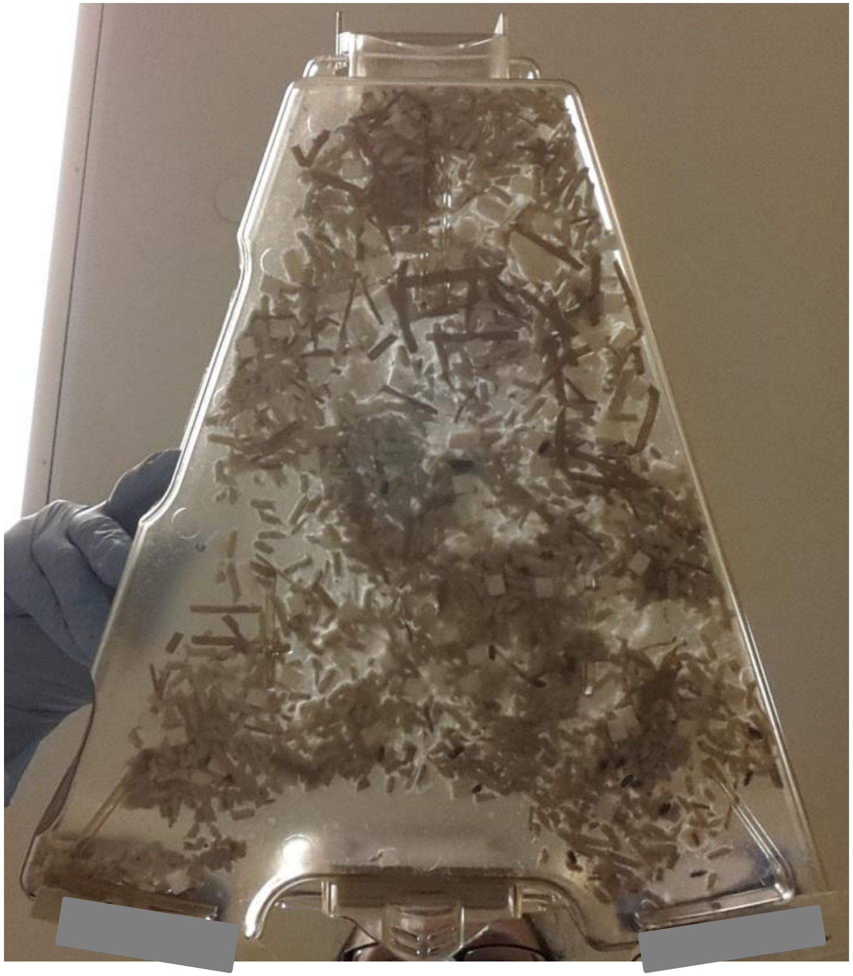

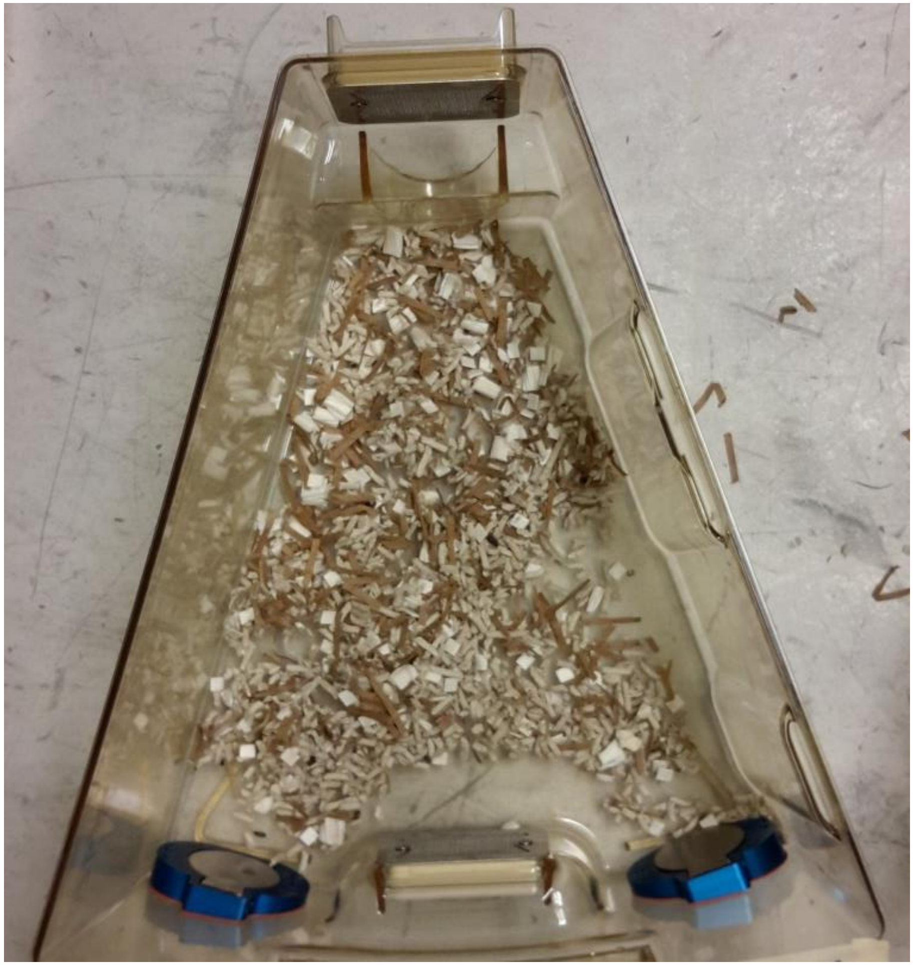

Aug 19 COMP 4 left

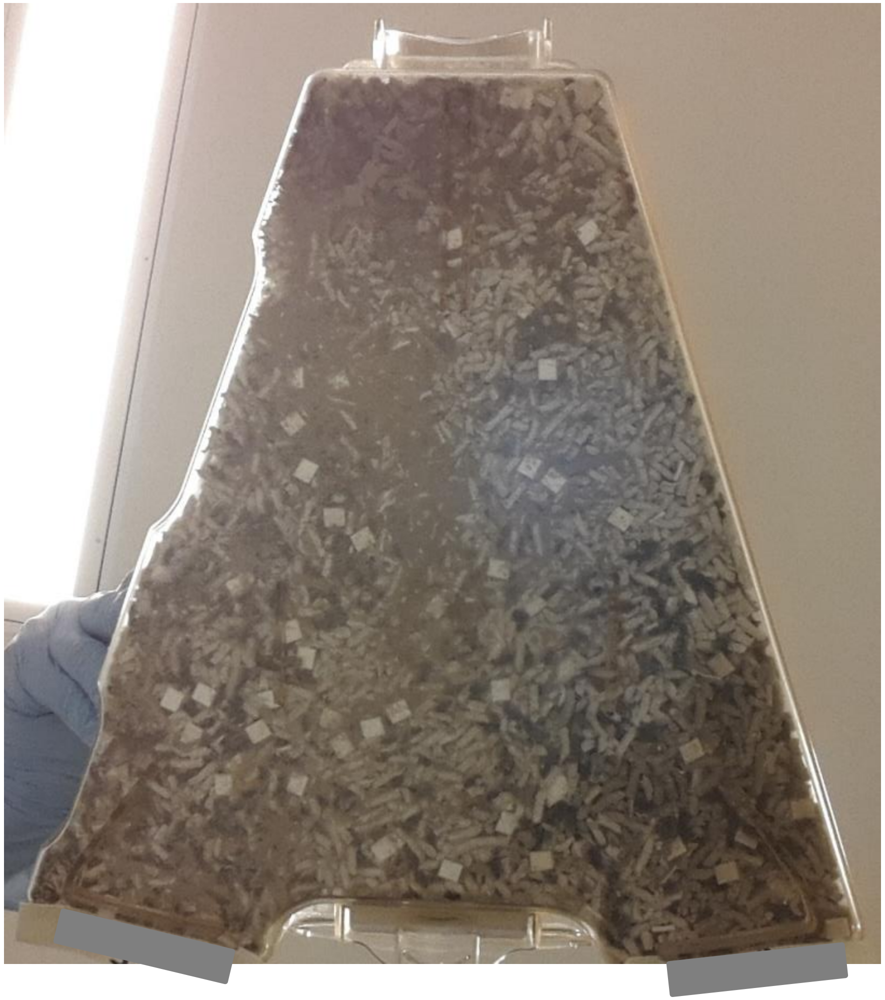

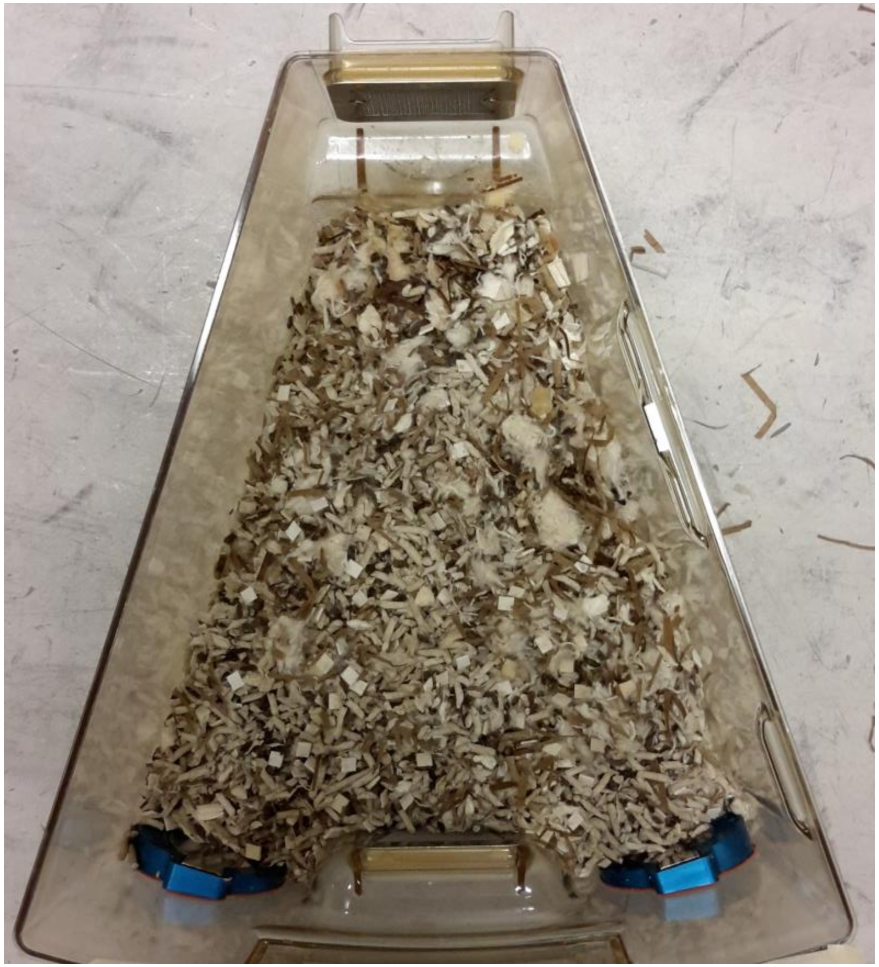

Aug 19 STD 4

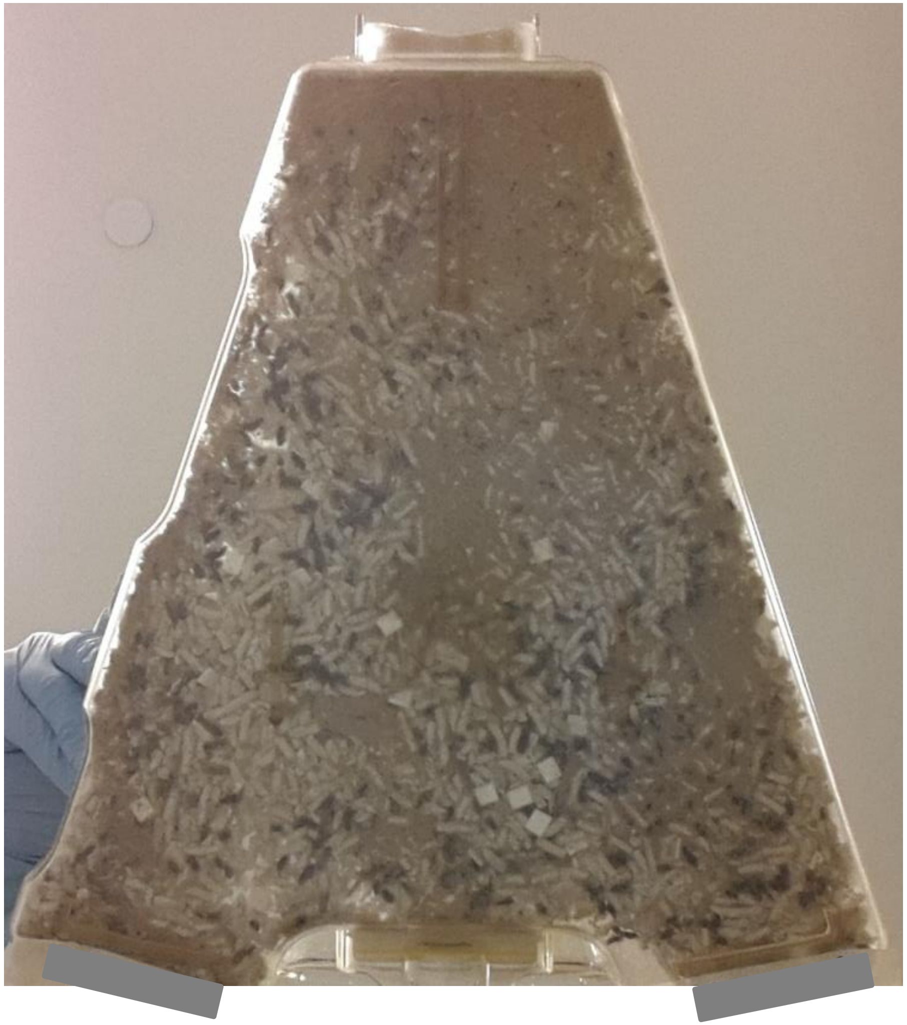

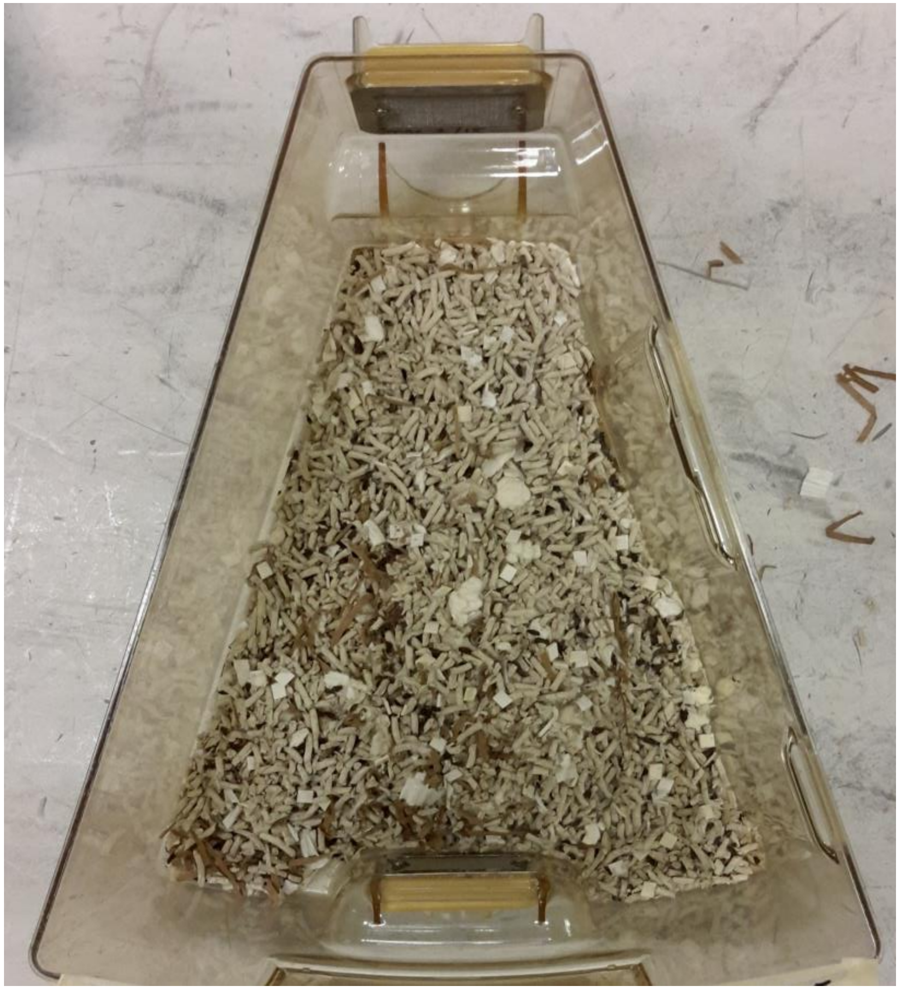

Aug 20 COMP 5 left

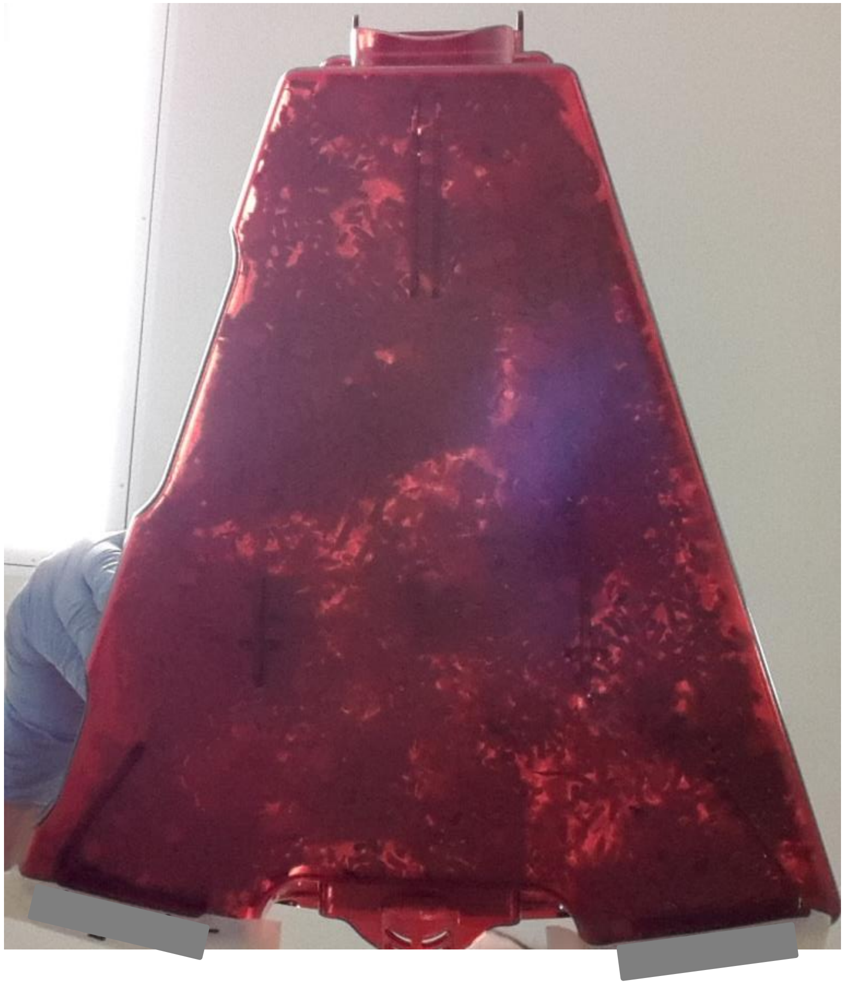

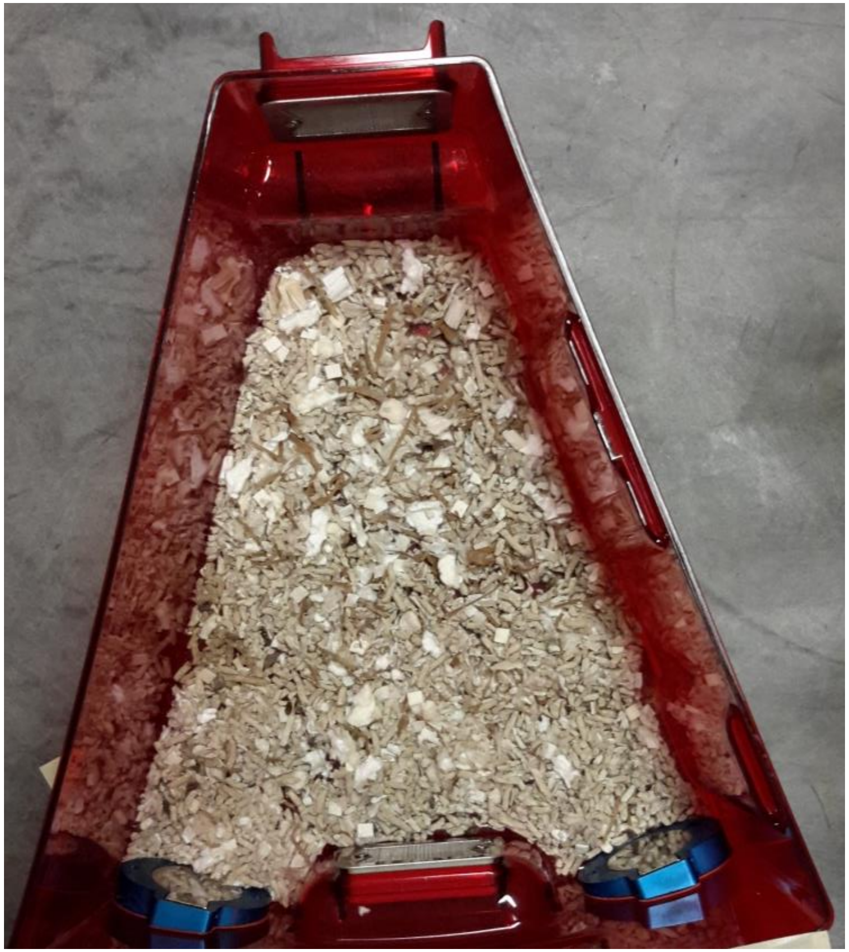

Aug 20 COMP 5 right

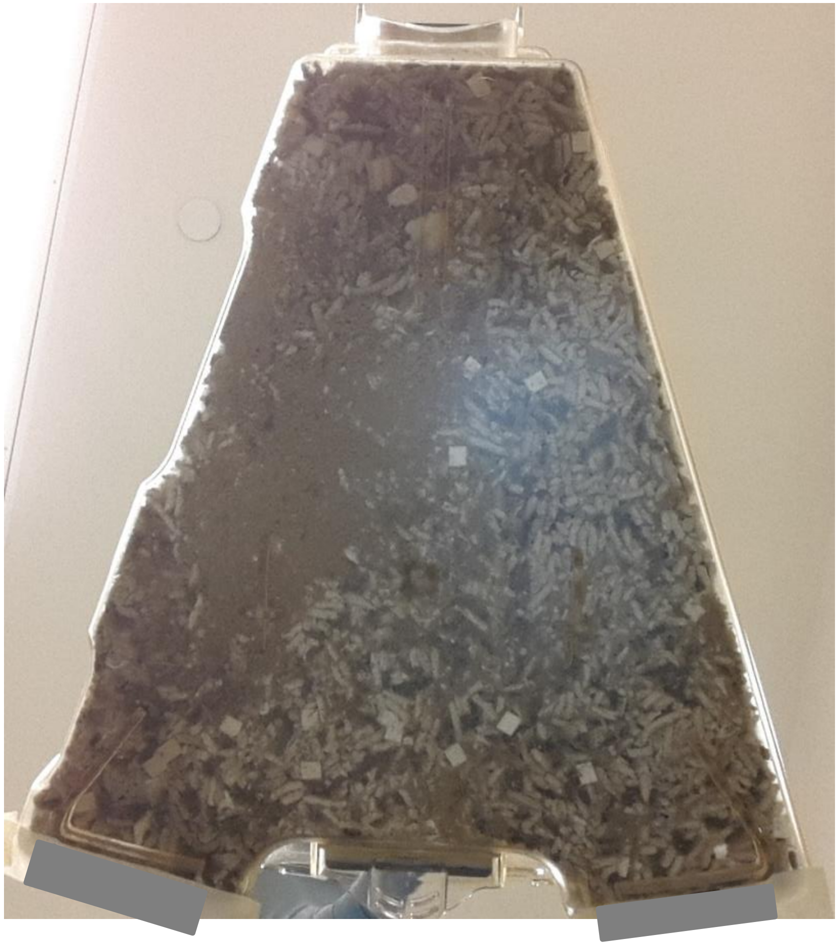

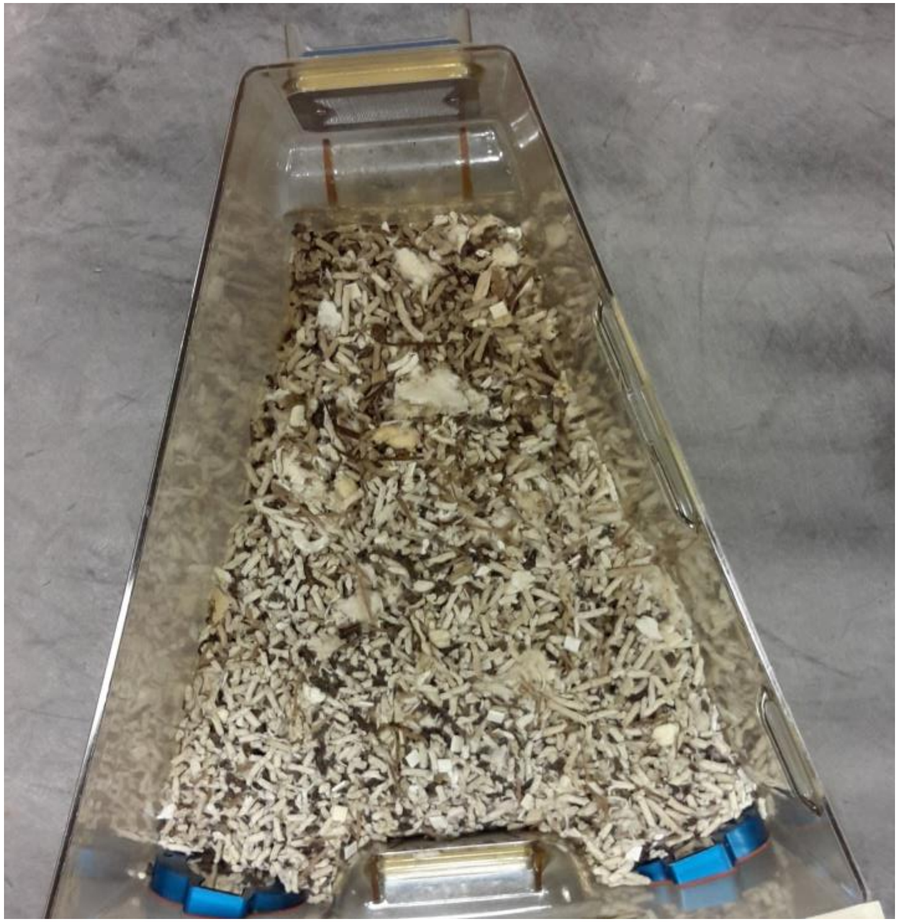

Aug 20 COMP 5 mid

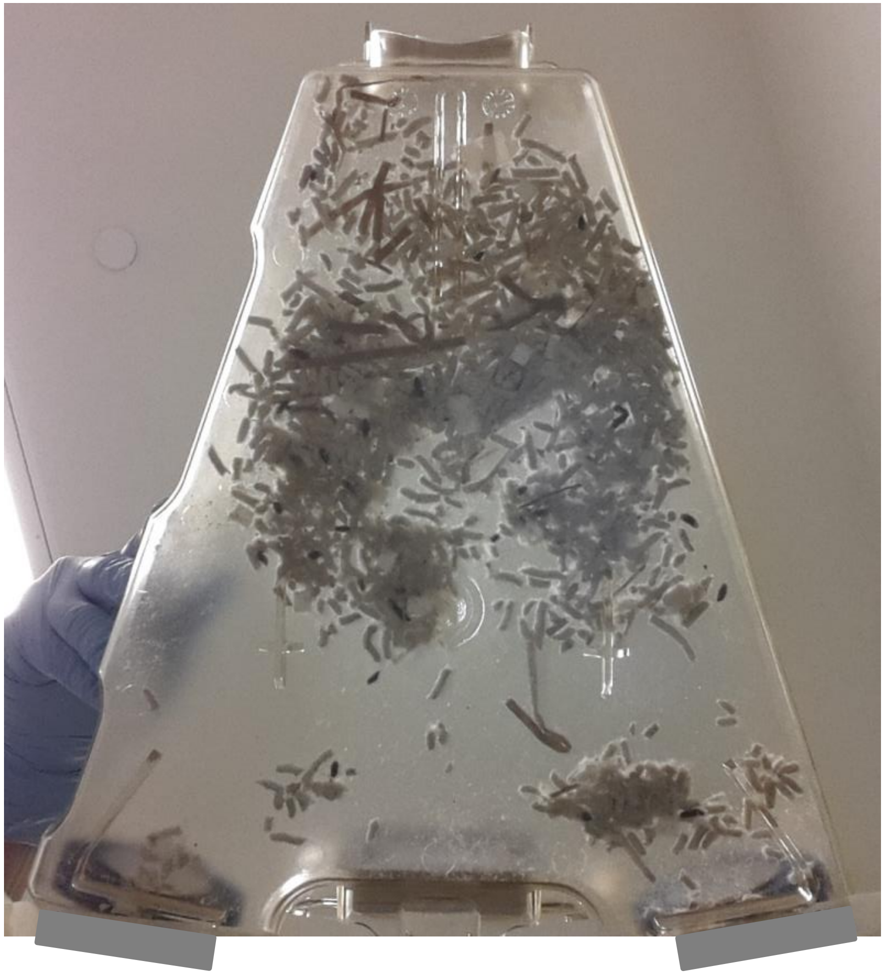

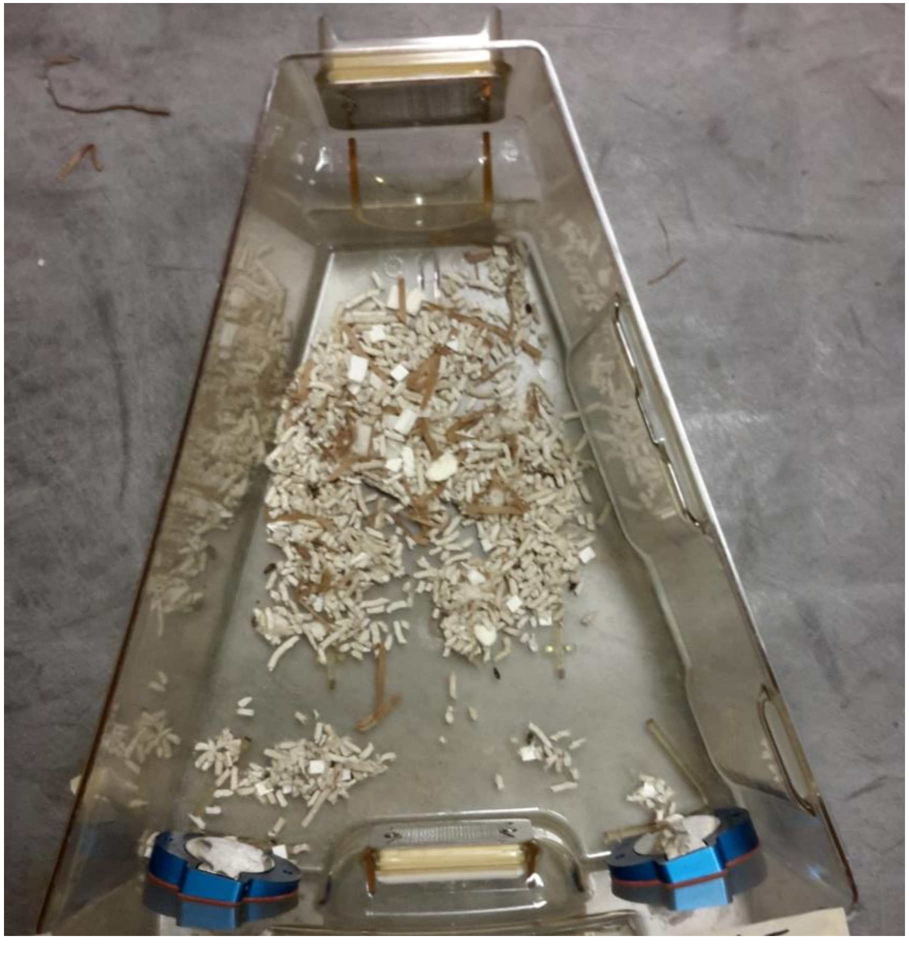

Aug 20 STD 5

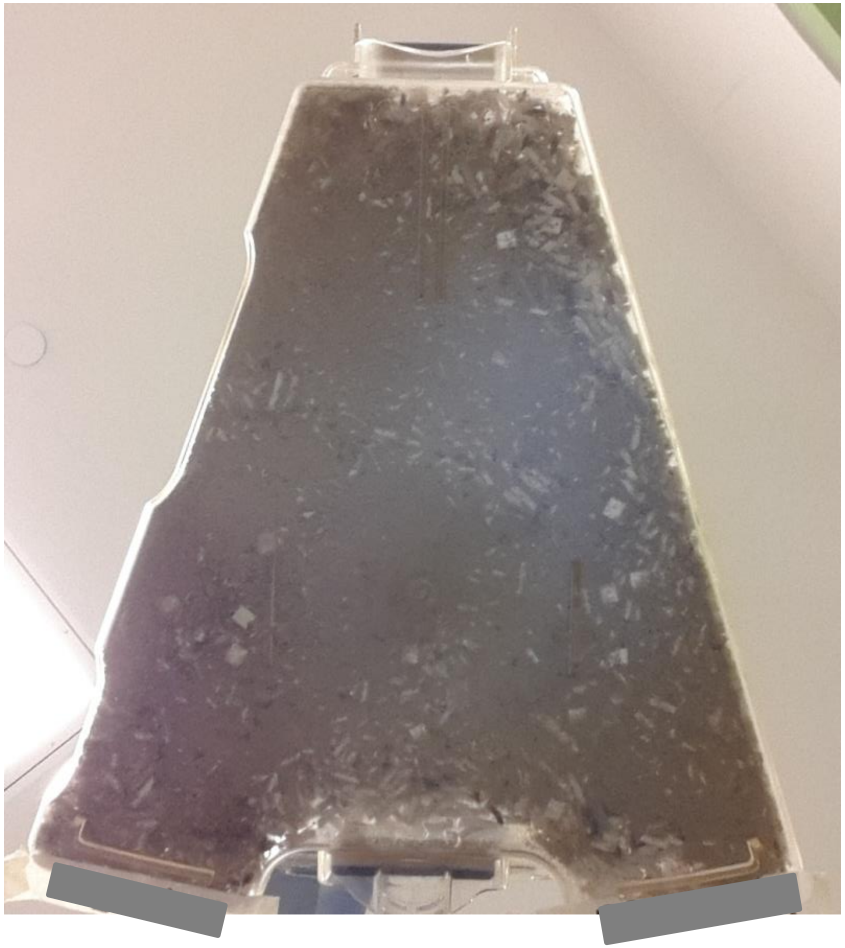

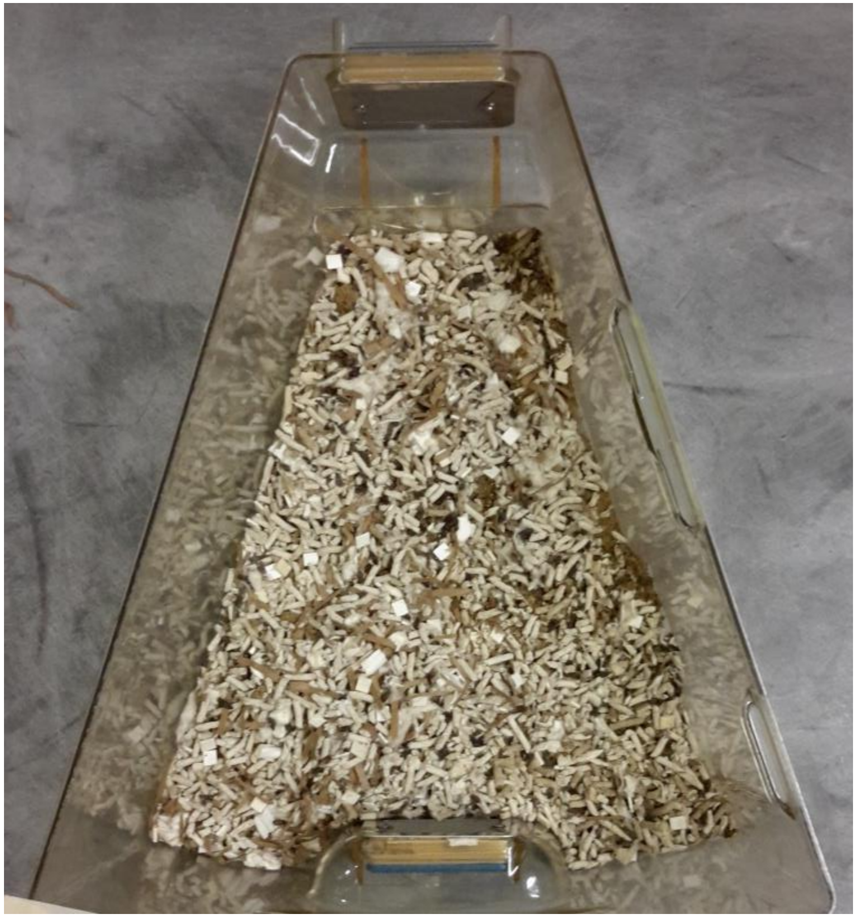

Aug 25 COMP 1 left

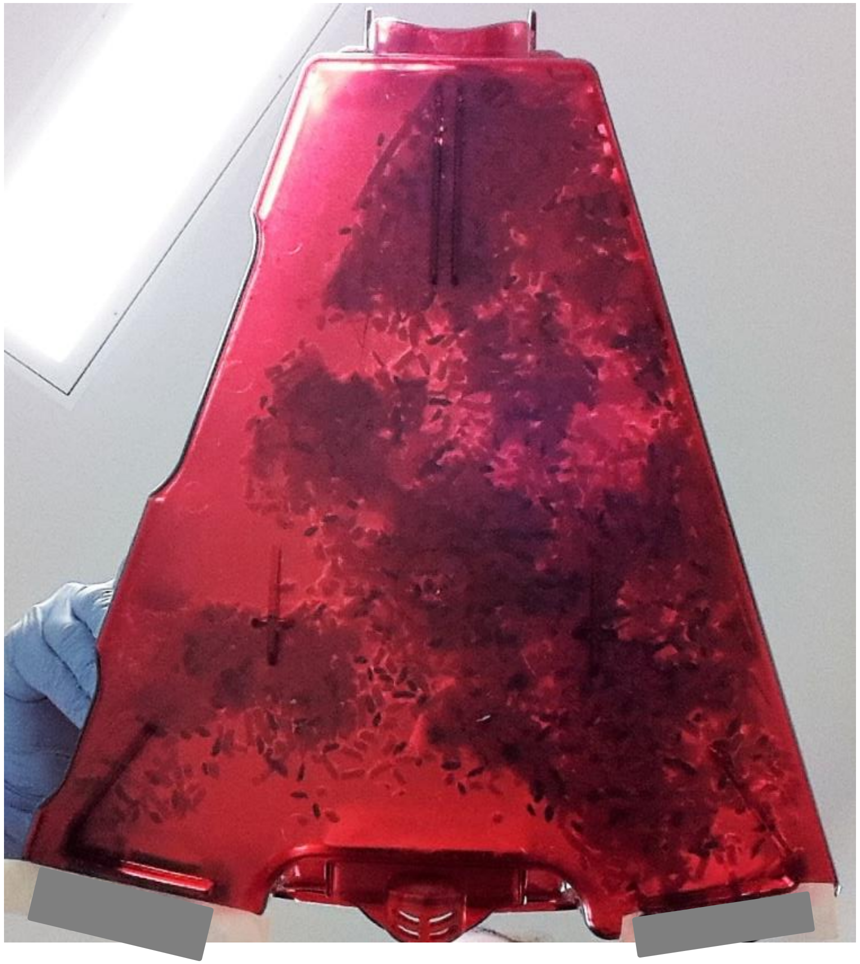

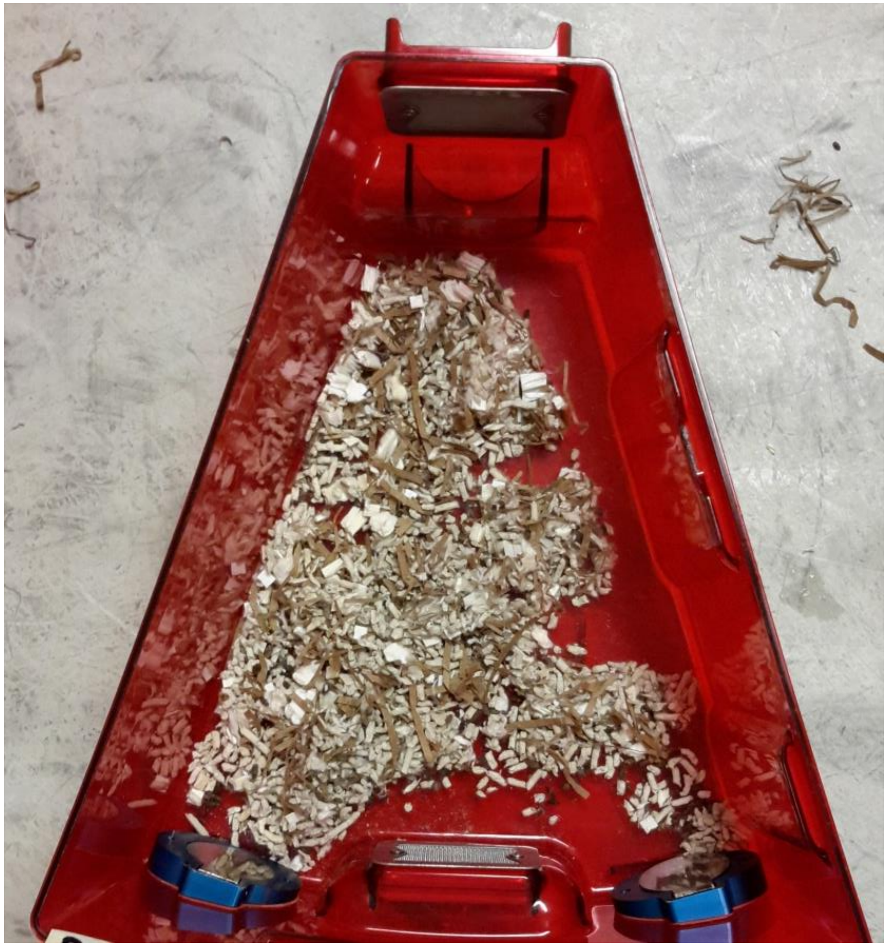

Aug 25 COMP 1 mid

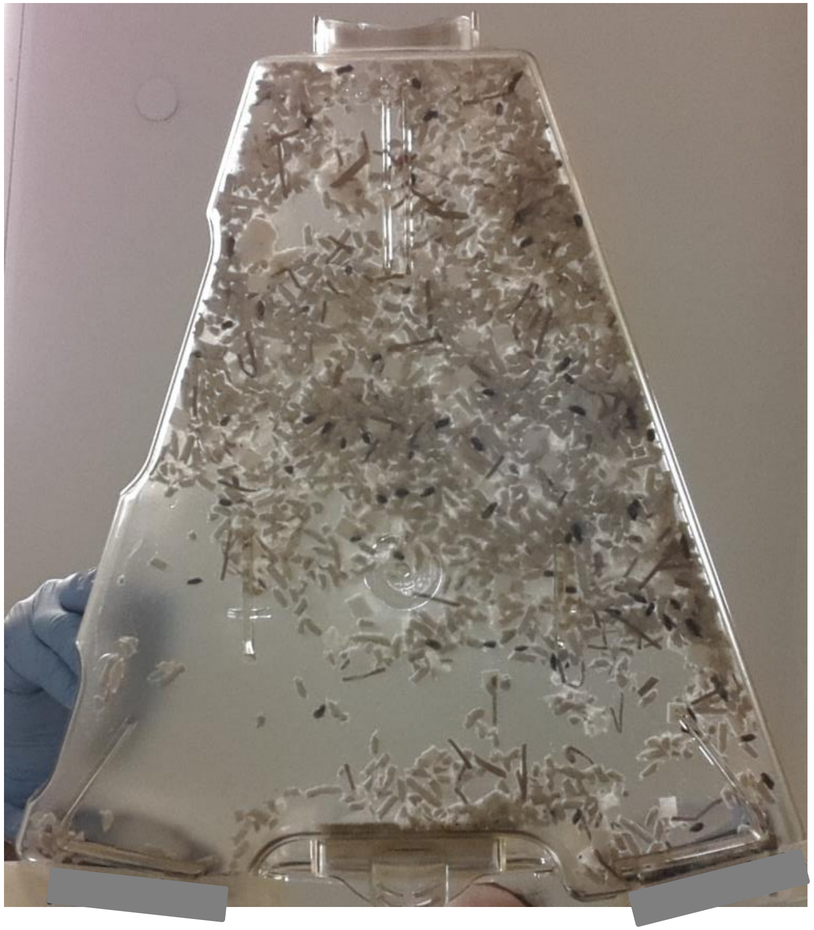

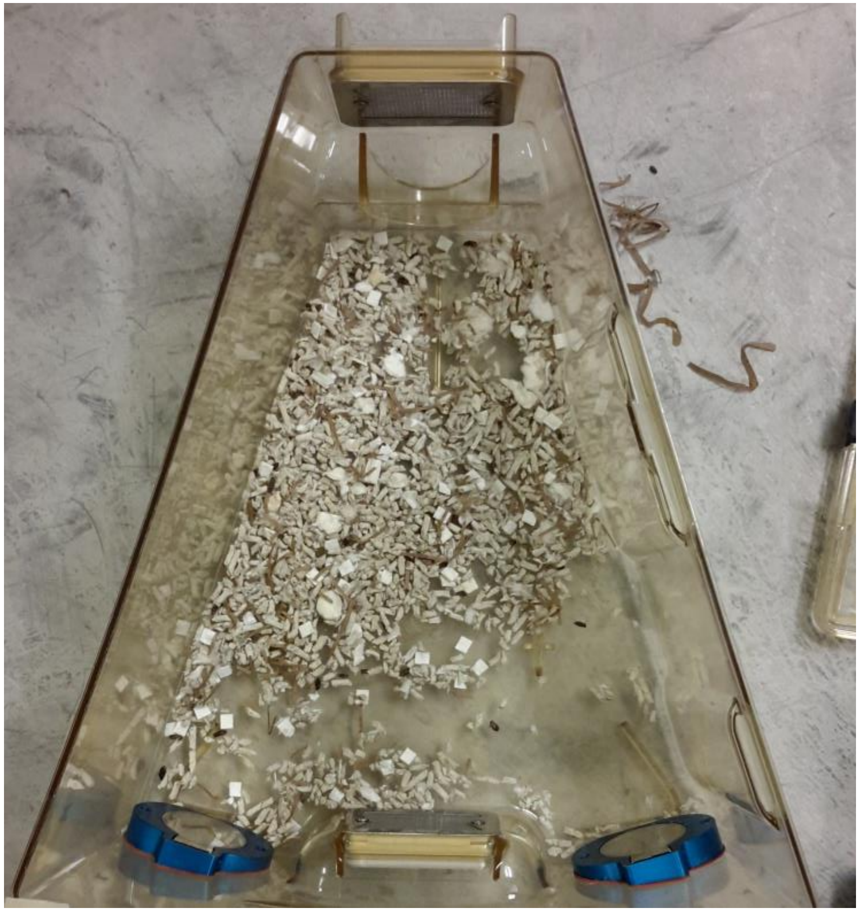

Aug 25 COMP 1 right

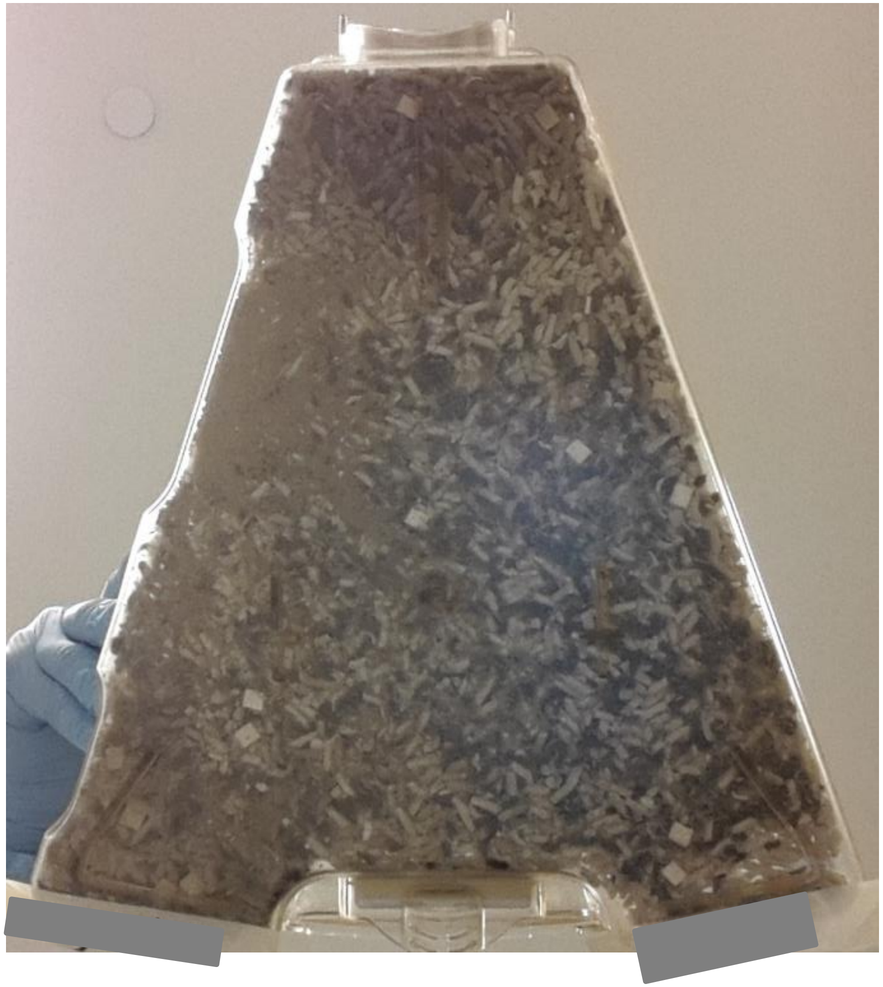

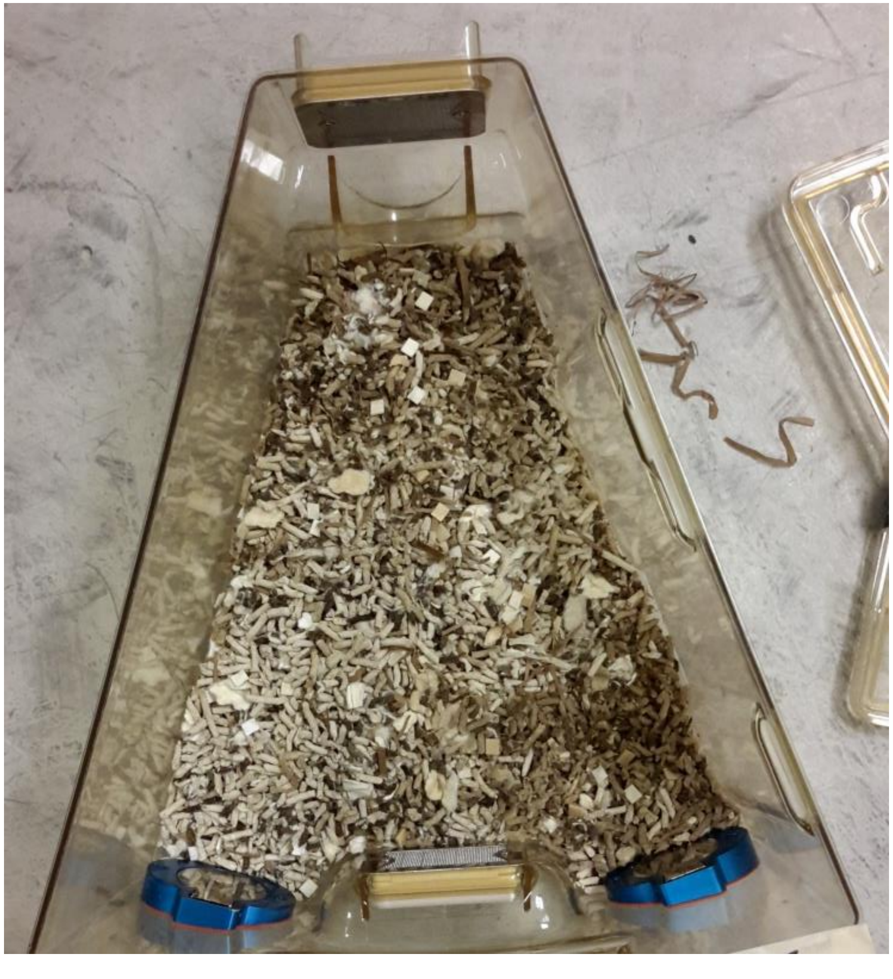

Aug 25 STD 1

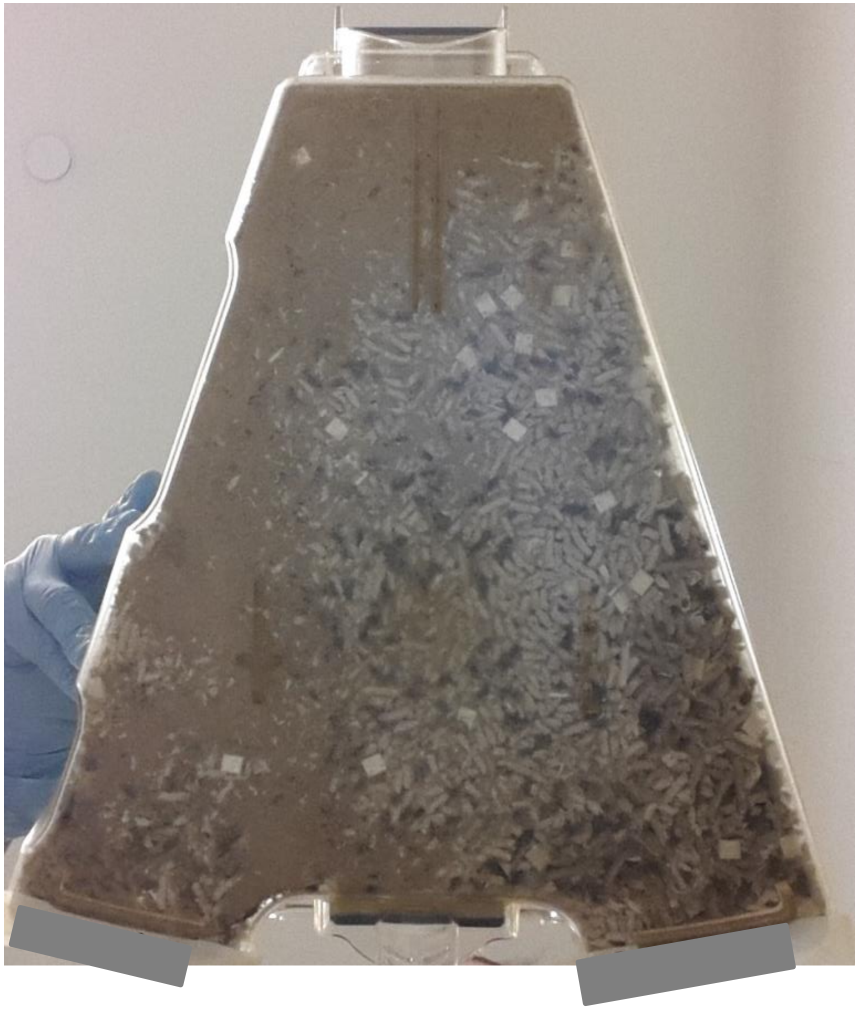

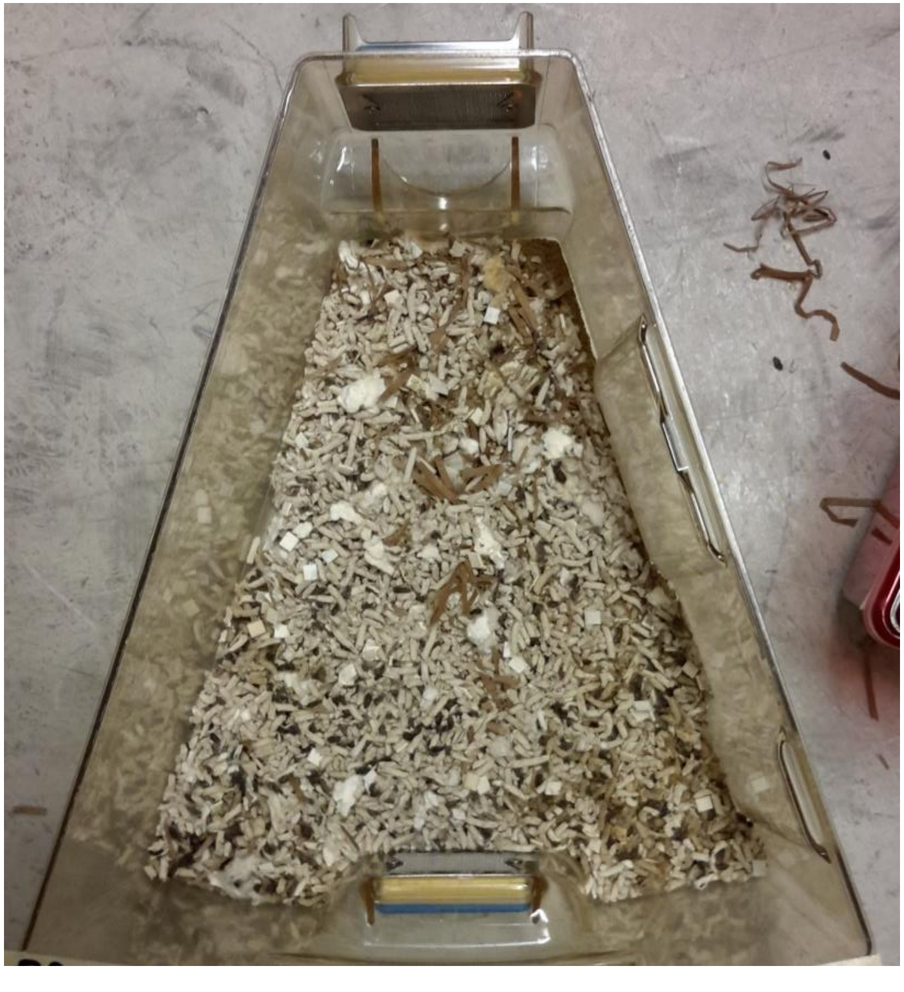

Aug 25 COMP 2 left

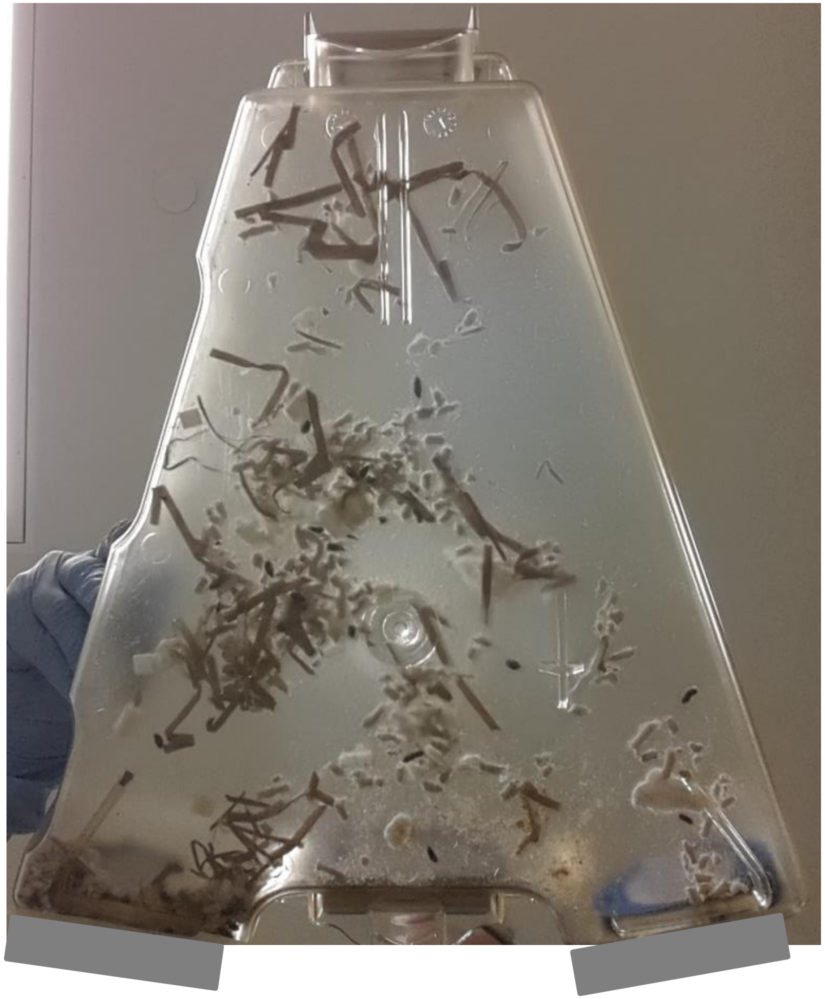

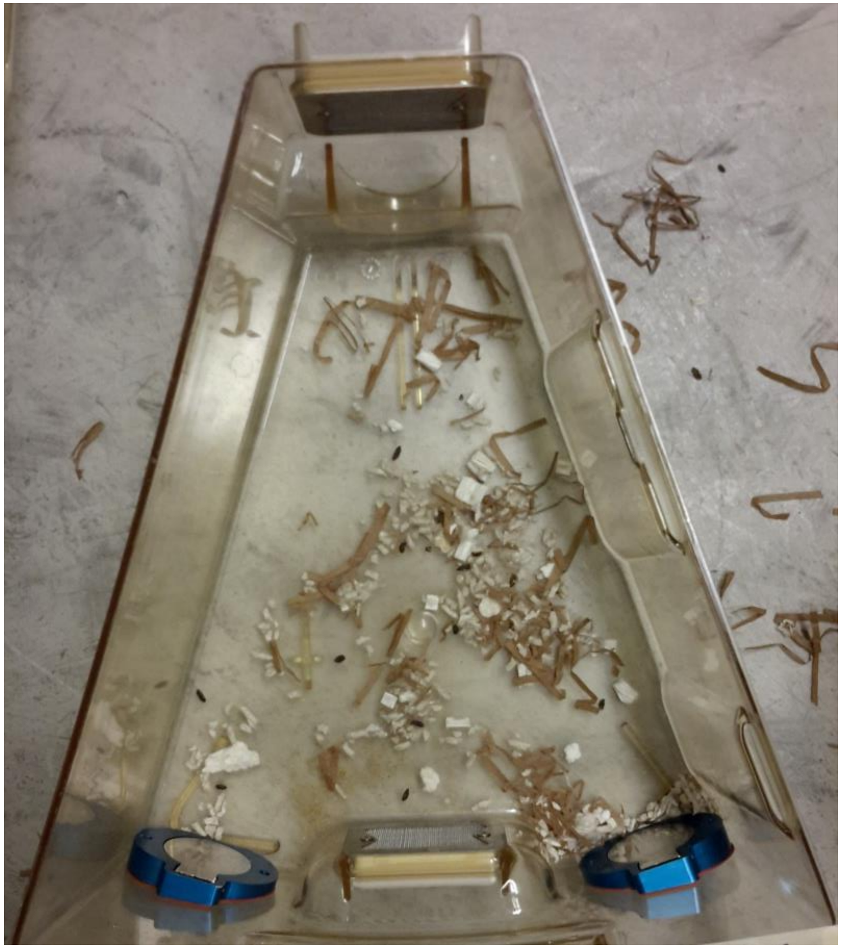

Aug 25 COMP 2 mid

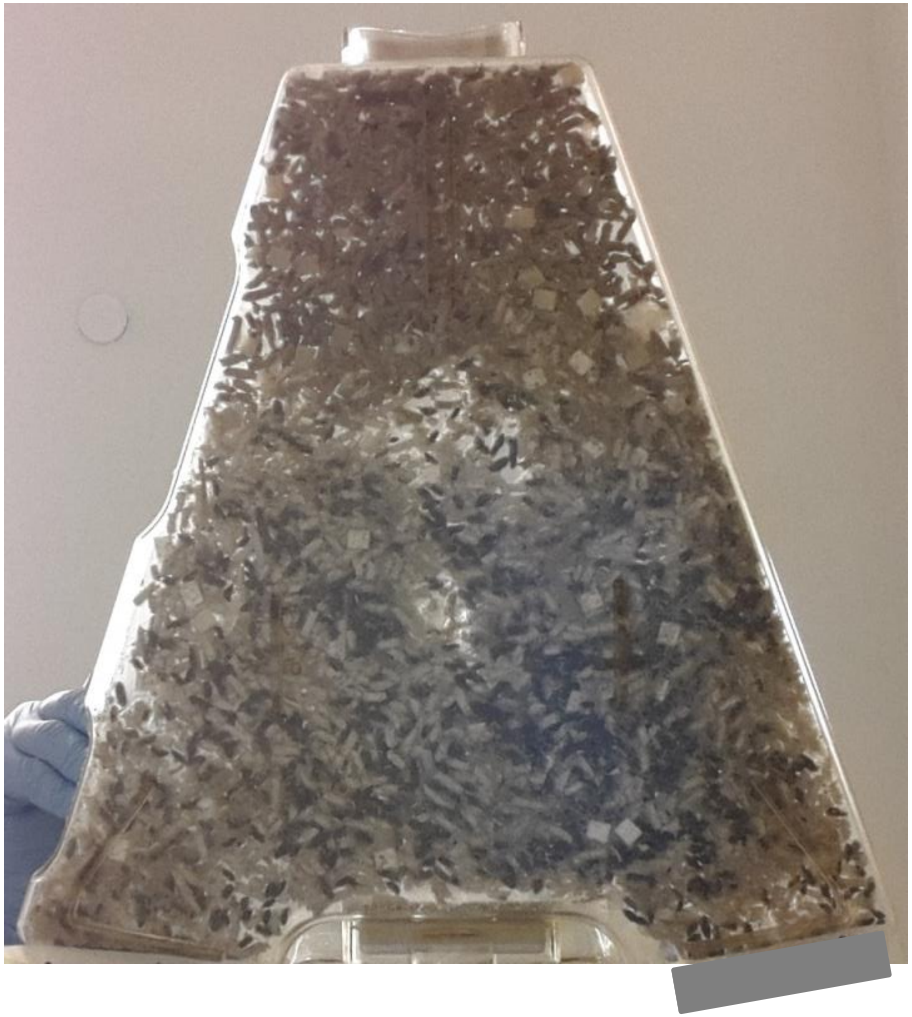

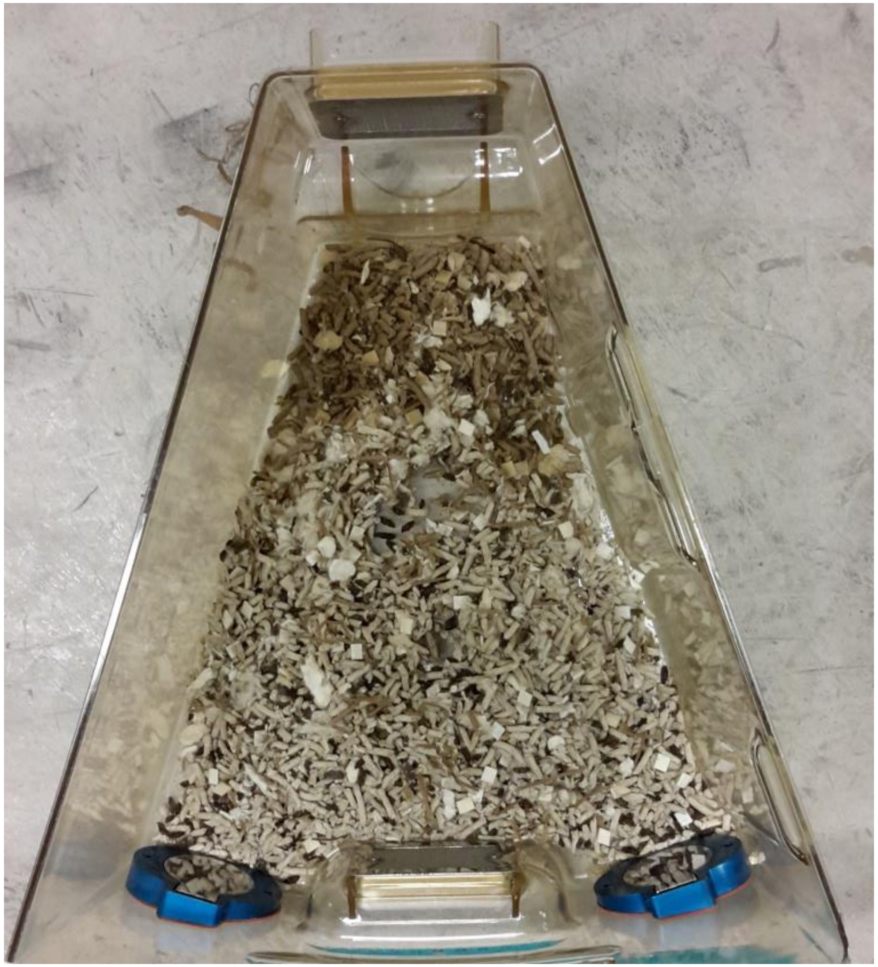

Aug 25 STD 2

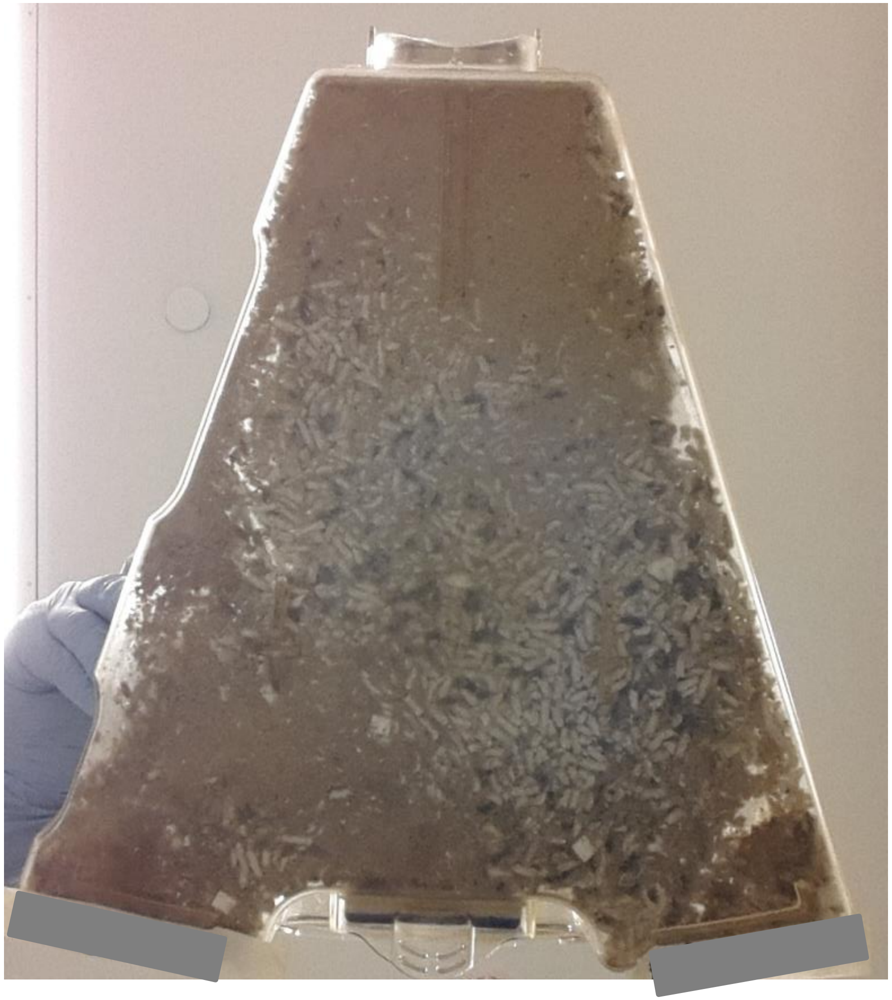

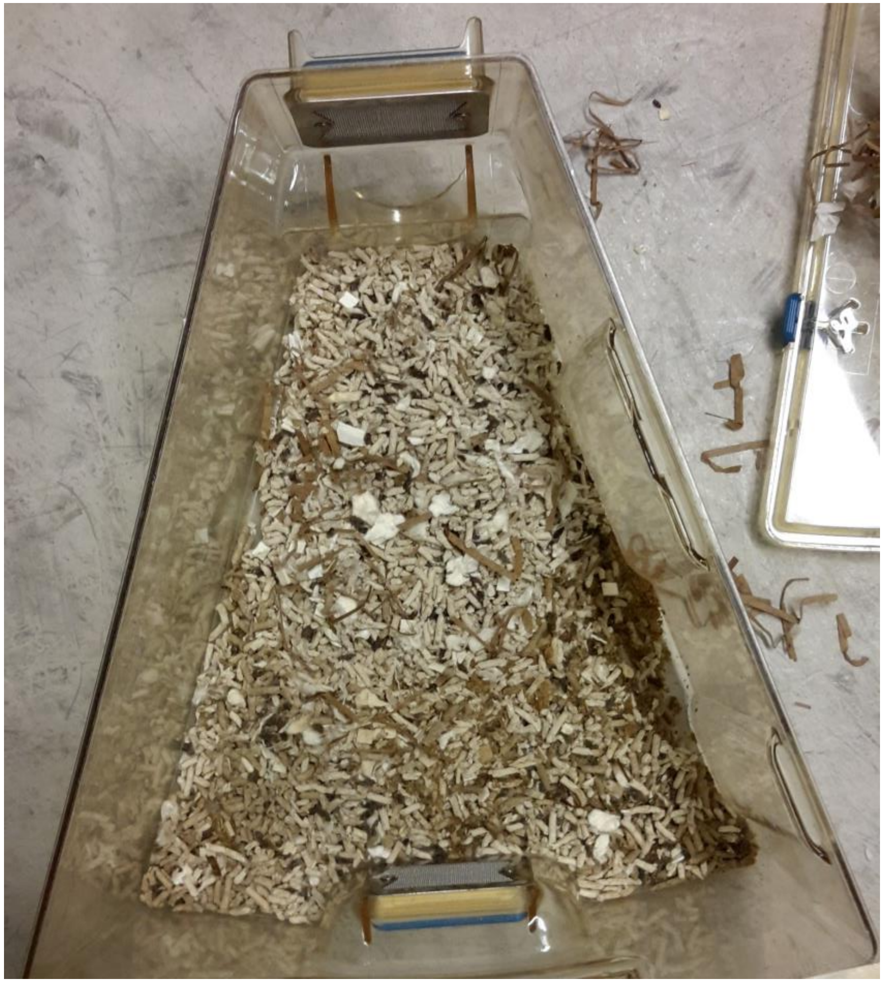

Aug 26 COMP 3 left

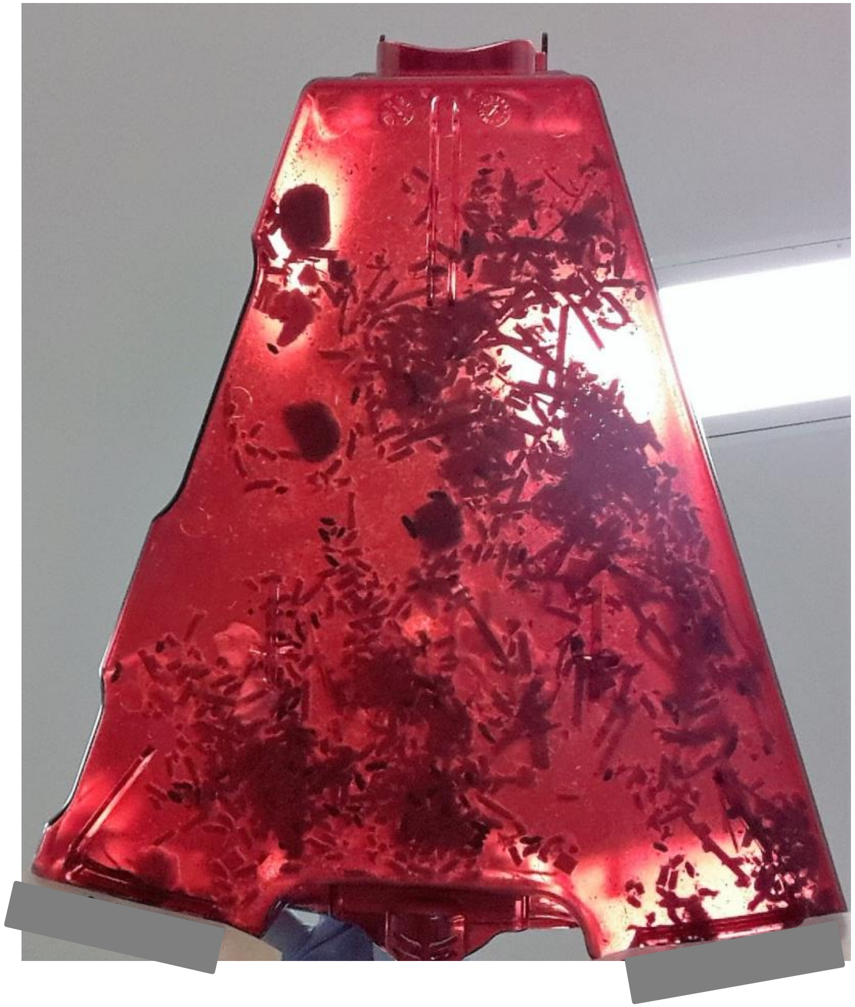

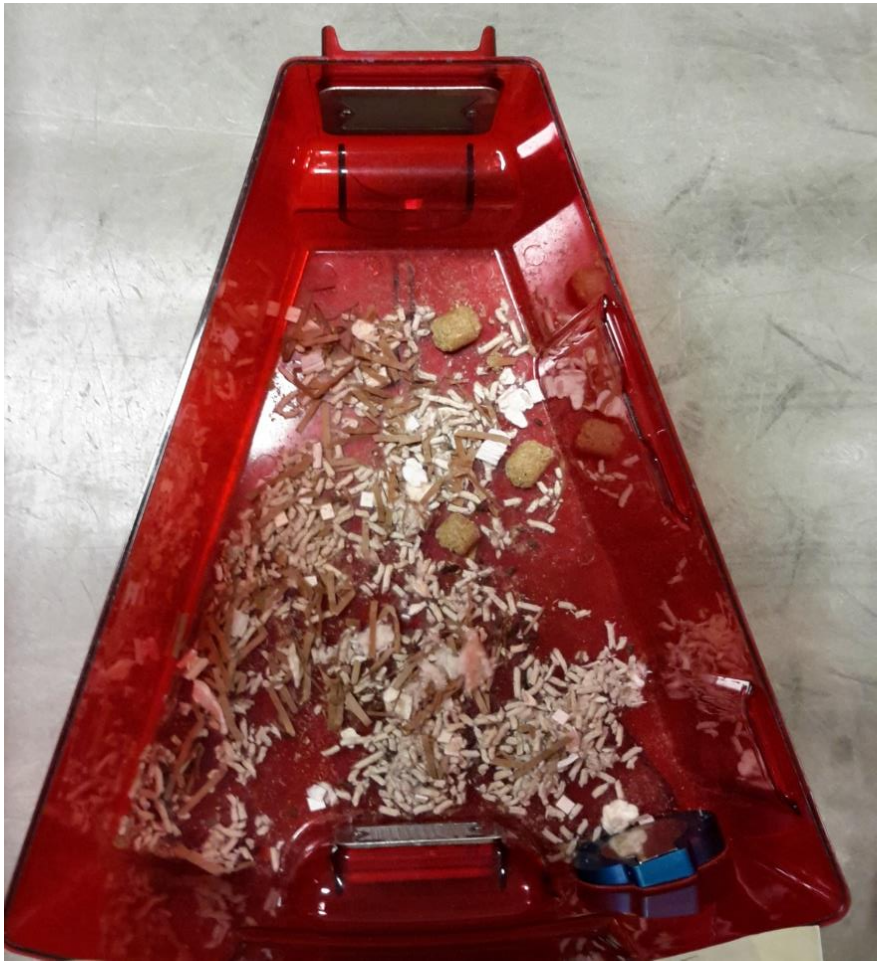

Aug 26 COMP 3 mid

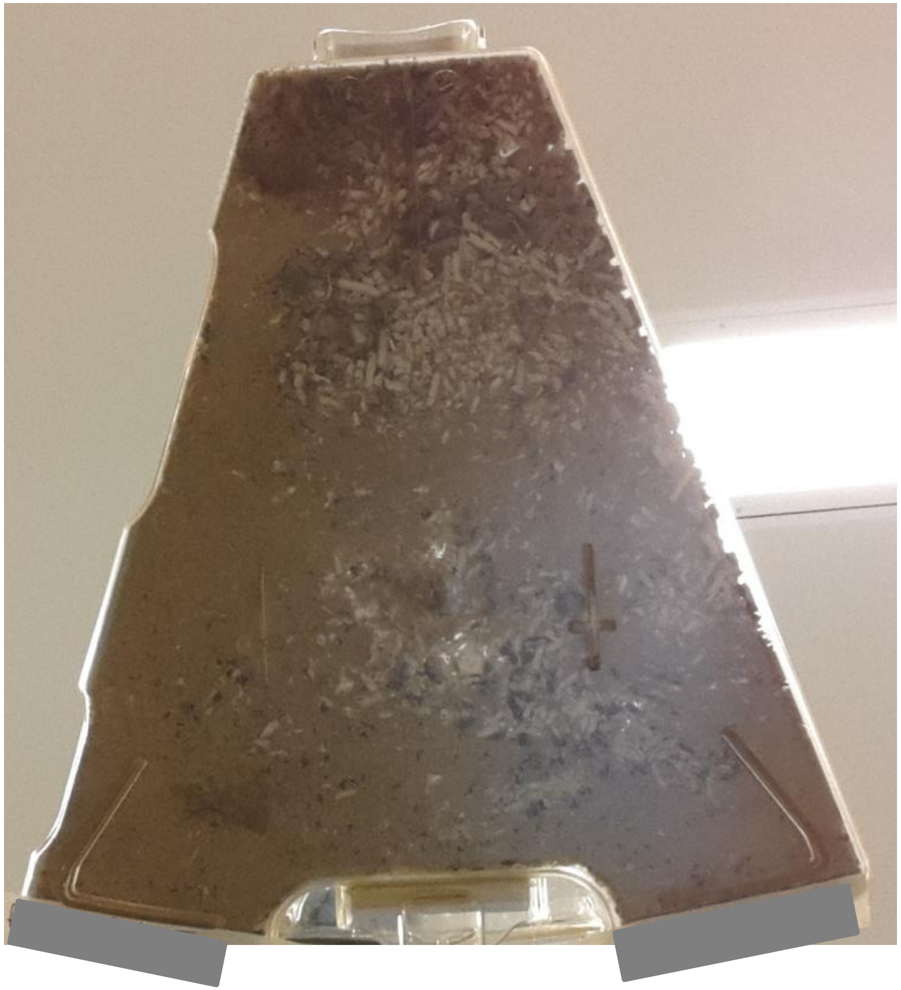

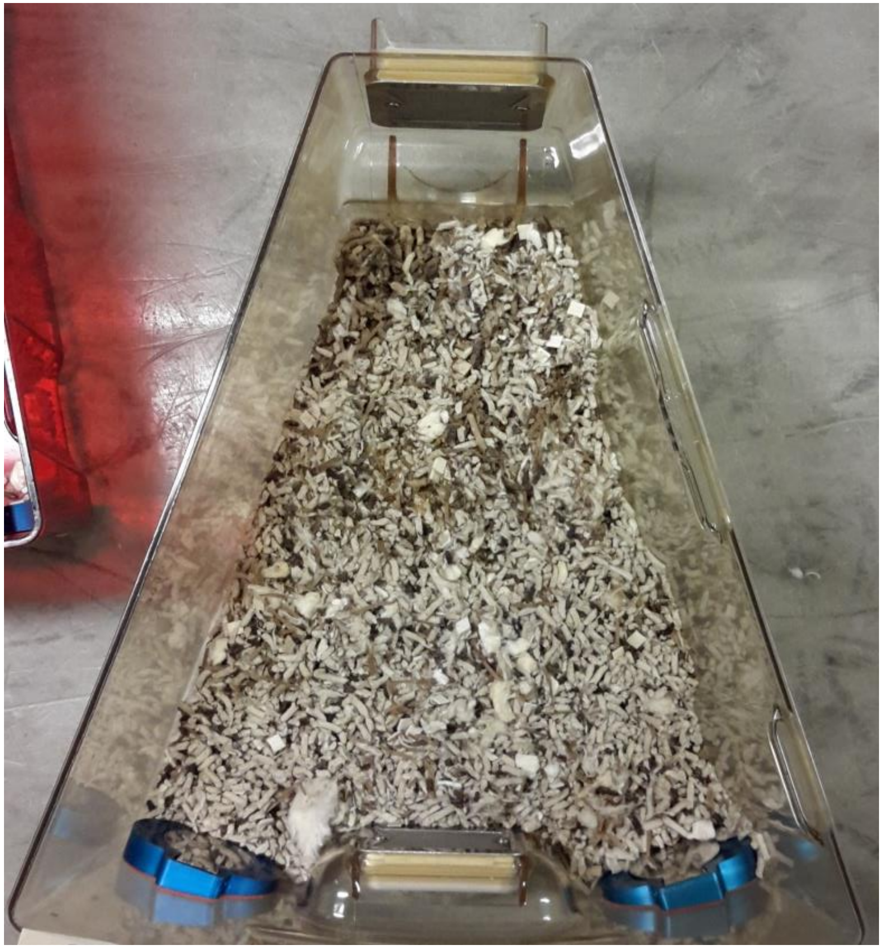

Aug 26 COMP 3 right

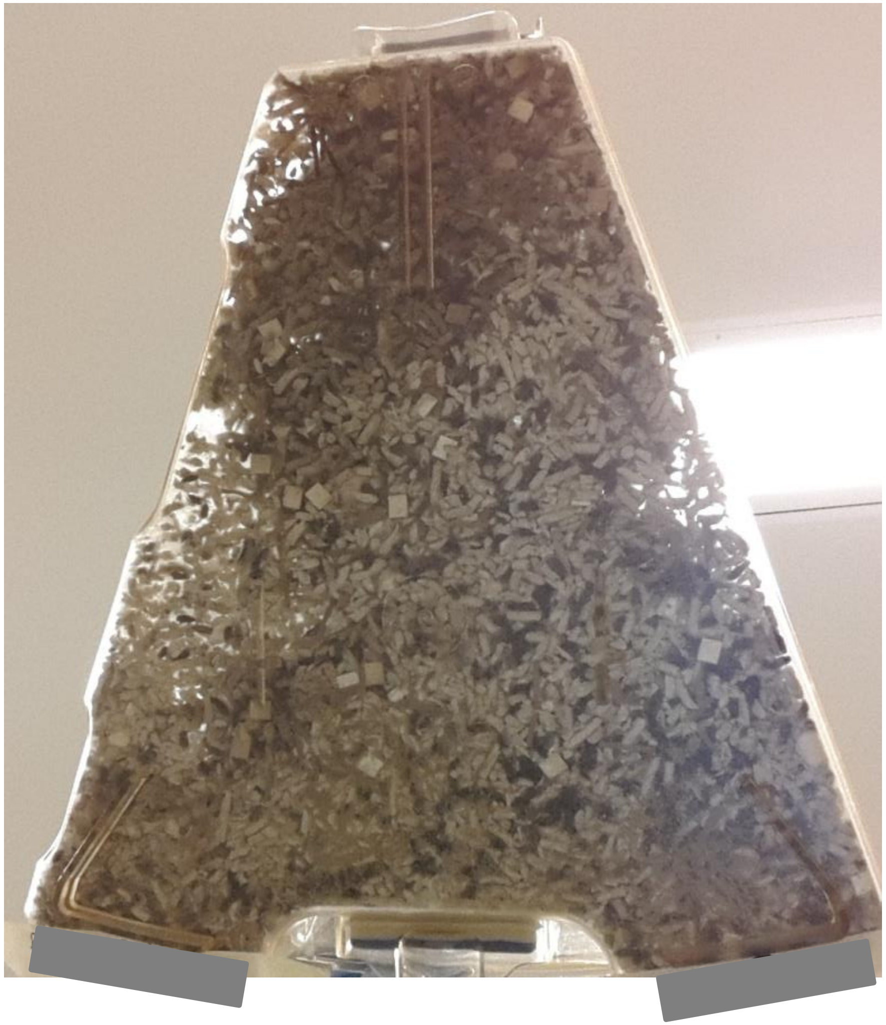

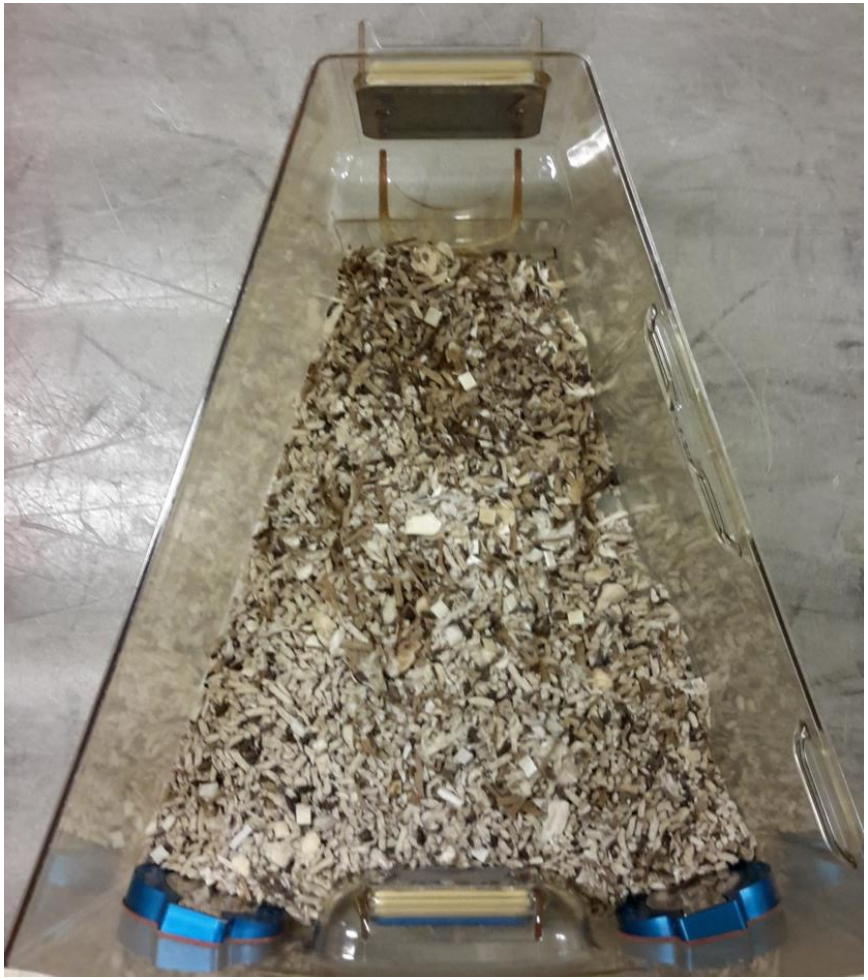

Aug 26 STD 3

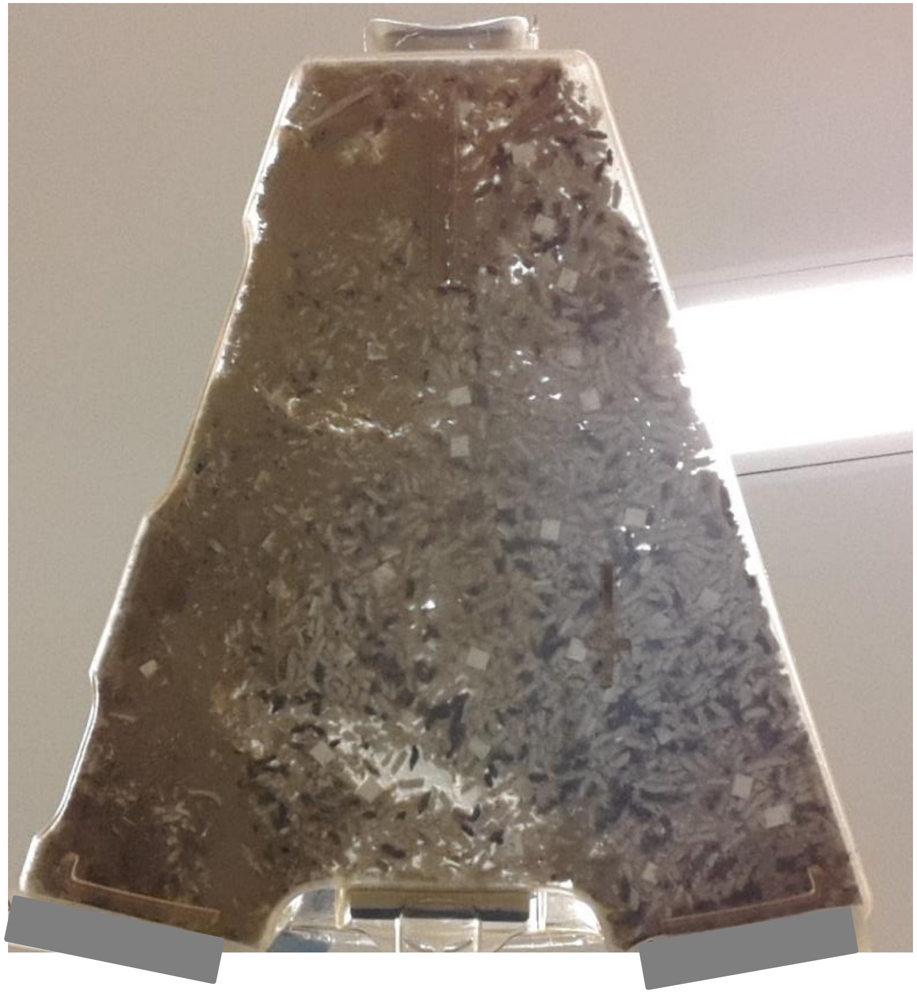

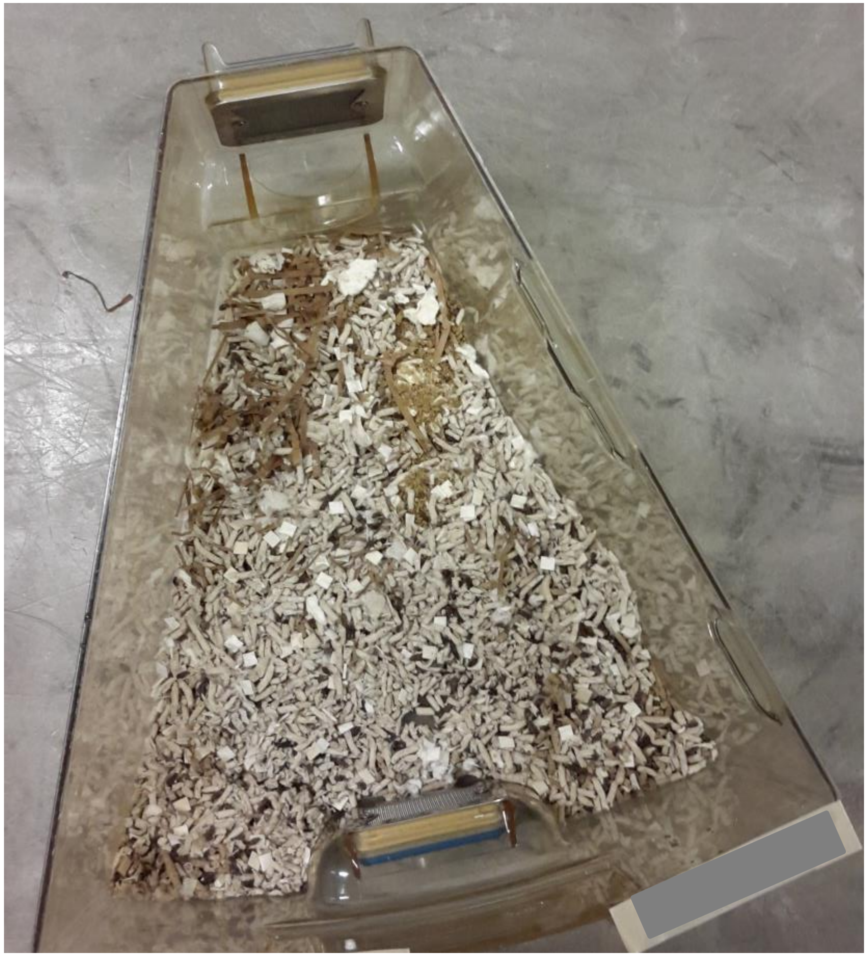

Aug 26 COMP 4 right

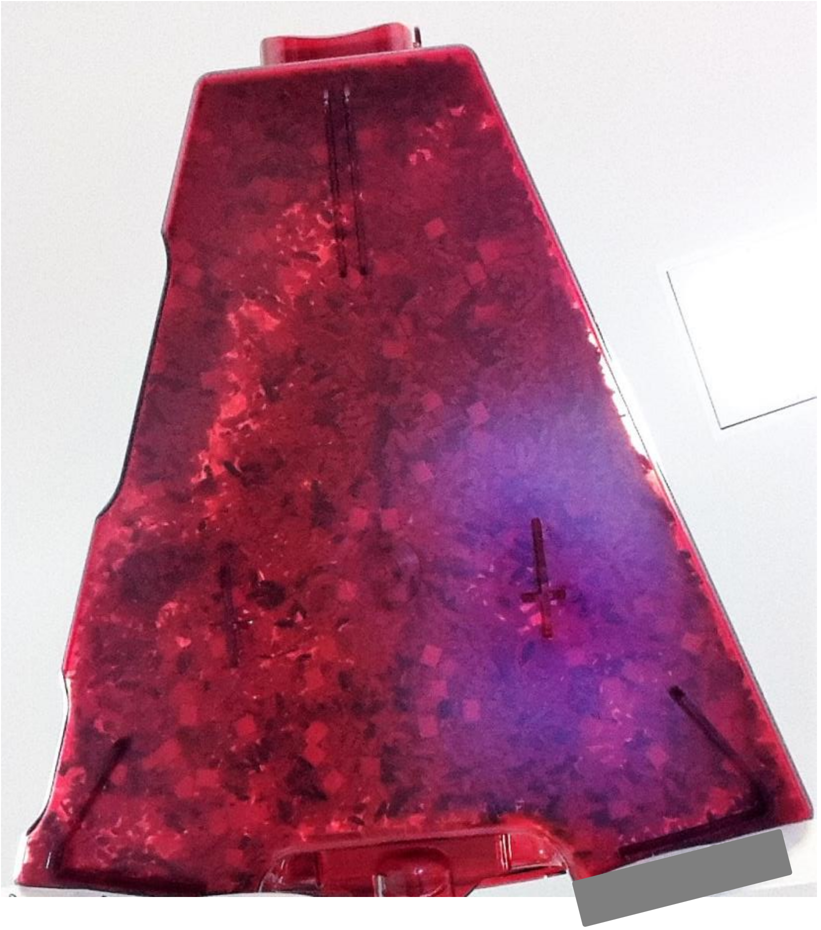

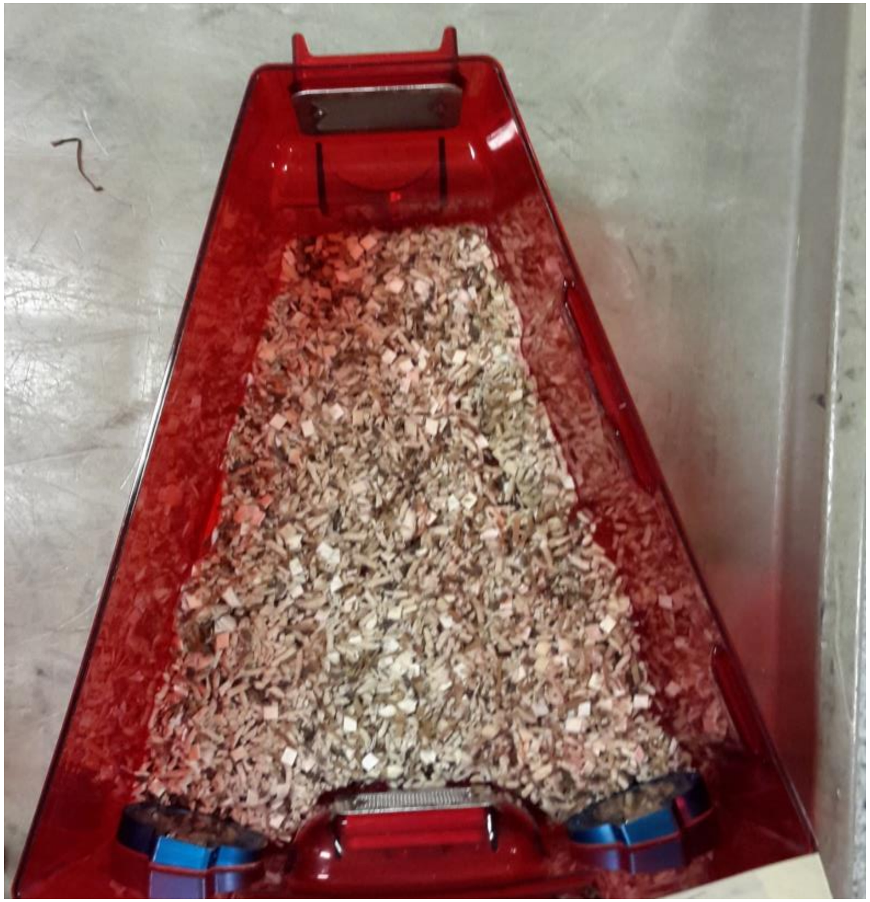

Aug 26 COMP 4 mid

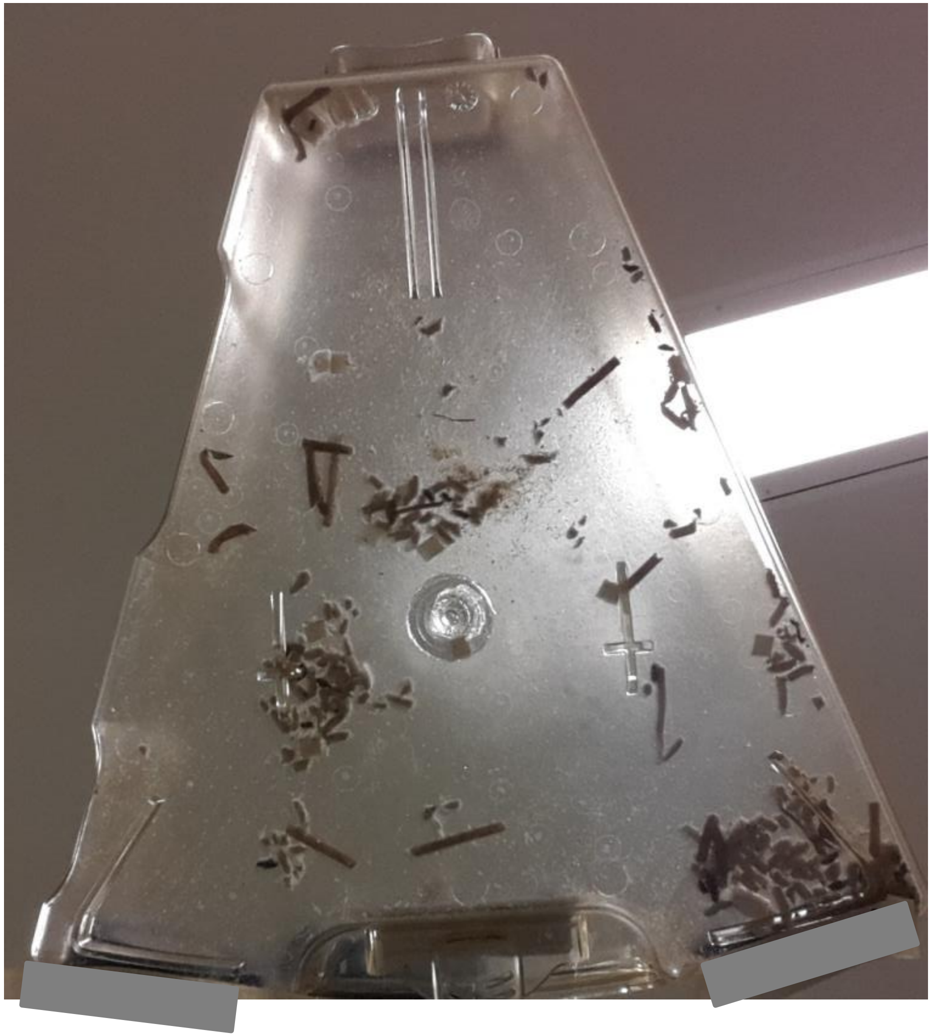

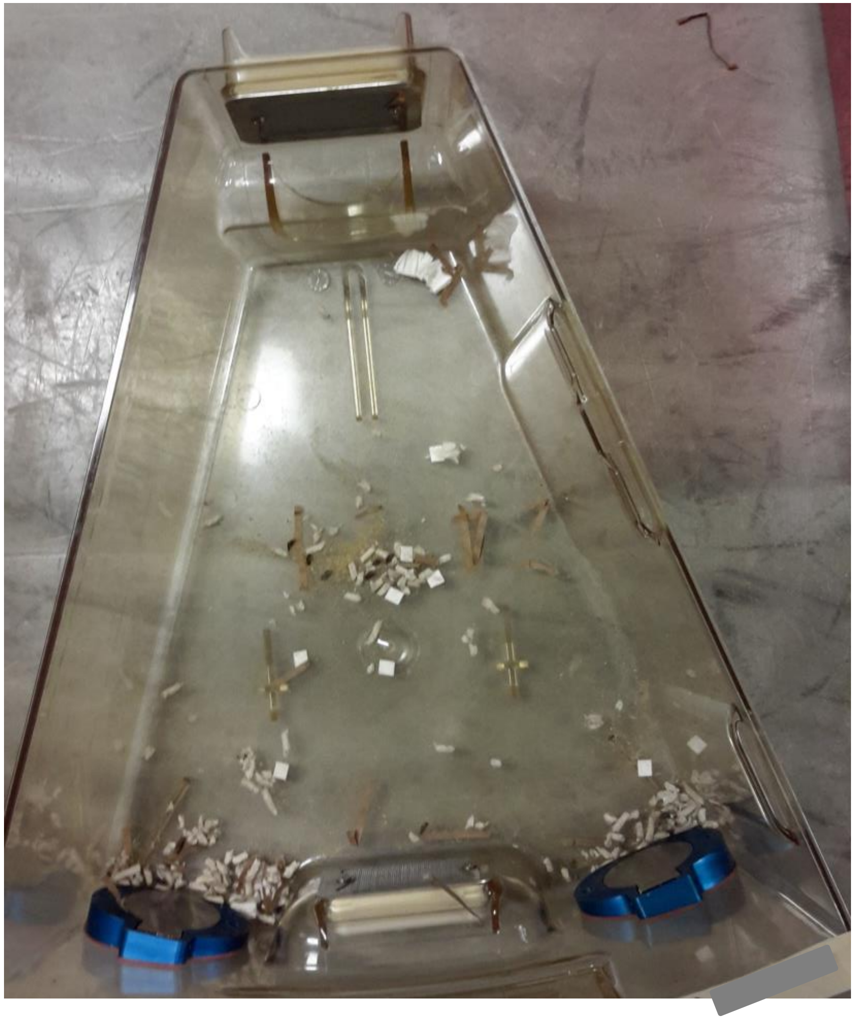

Aug 26 COMP 4 left

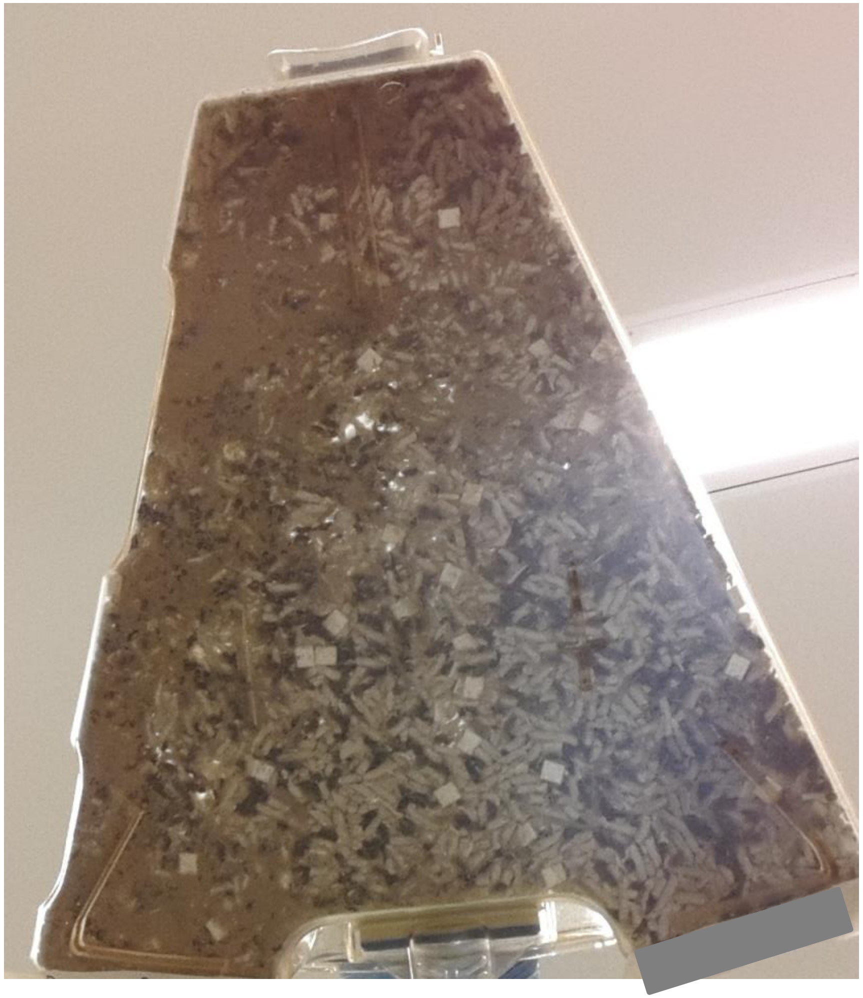

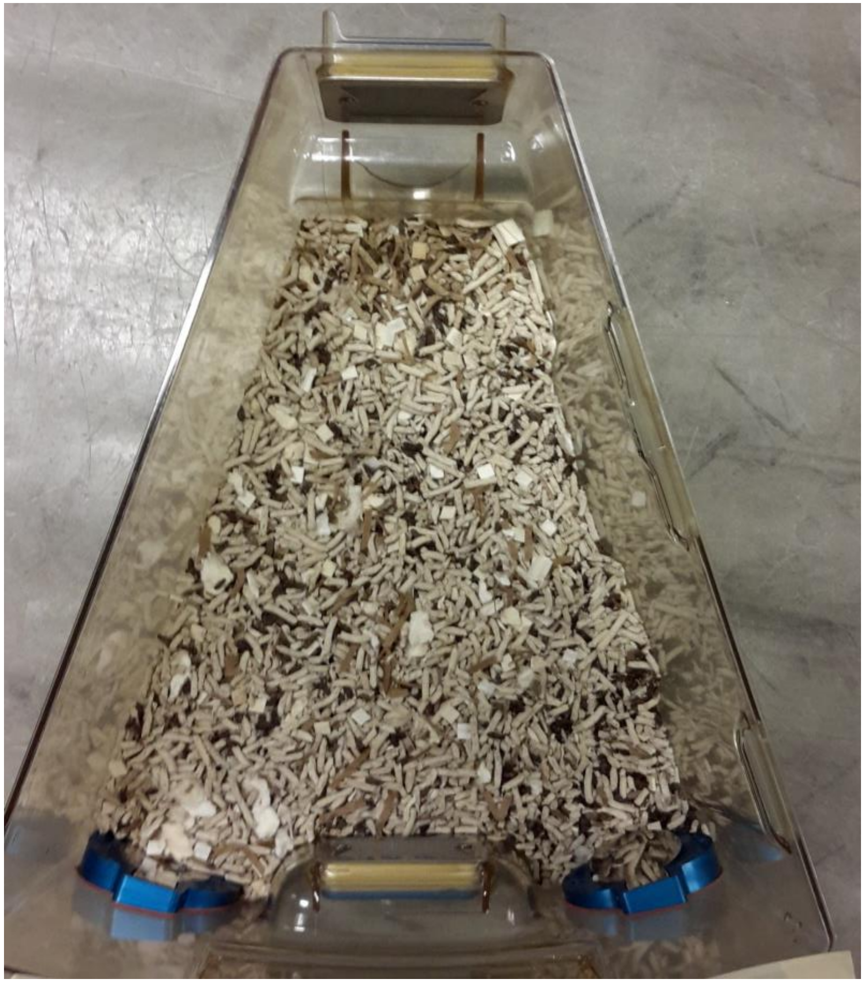

Aug 26 STD 4

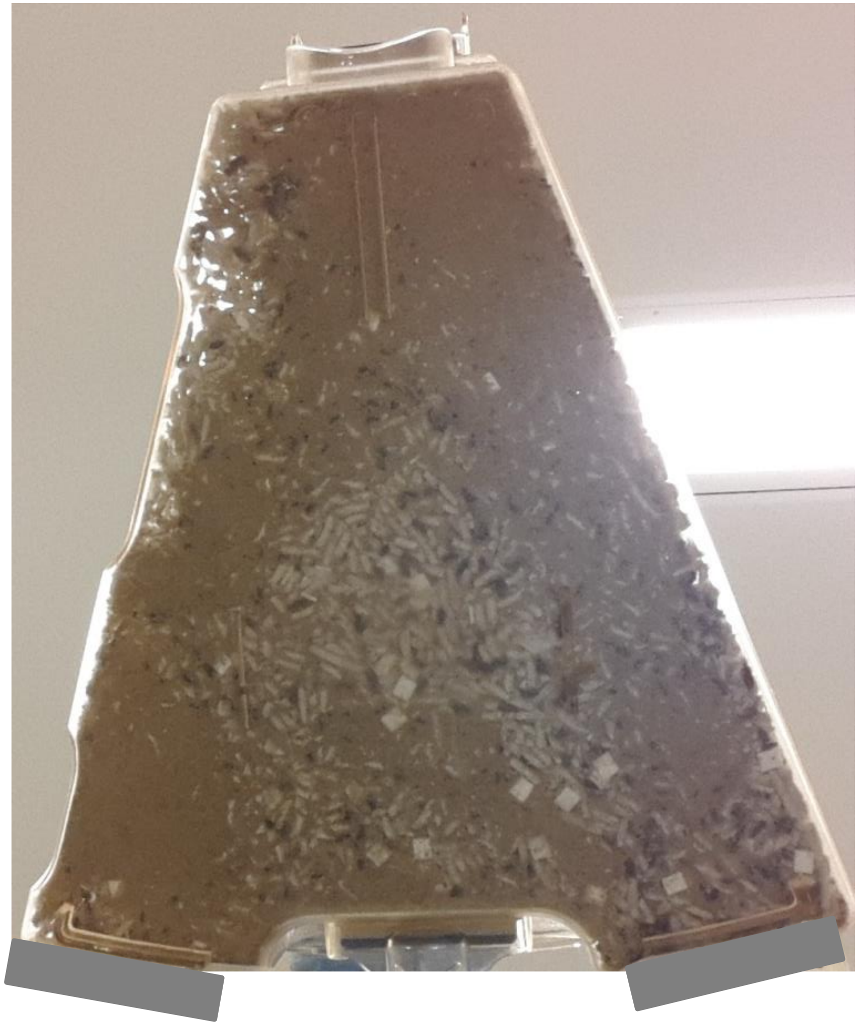 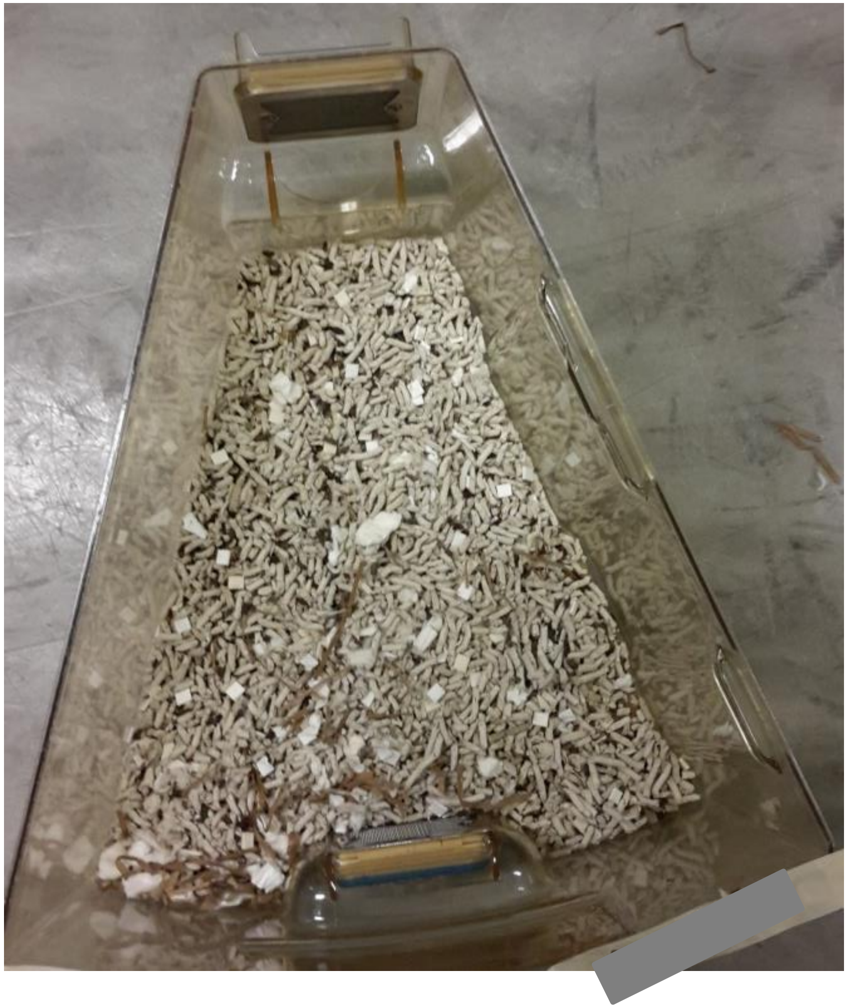

Aug 27 COMP 5 right

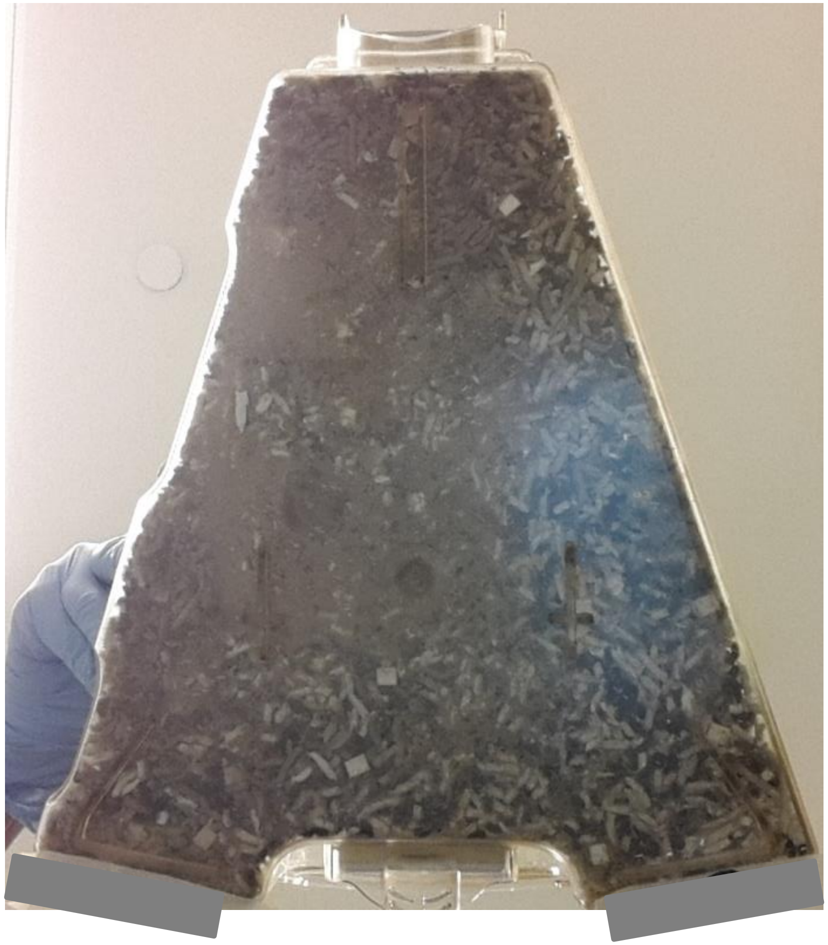

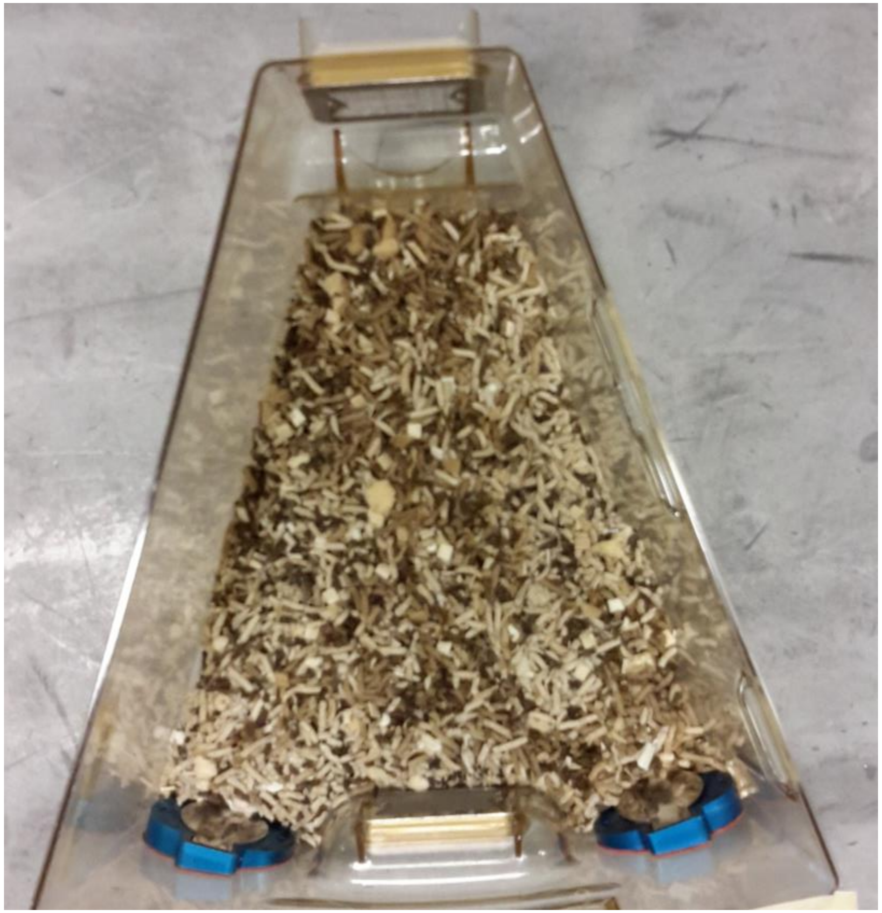

Aug 27 COMP 5 mid

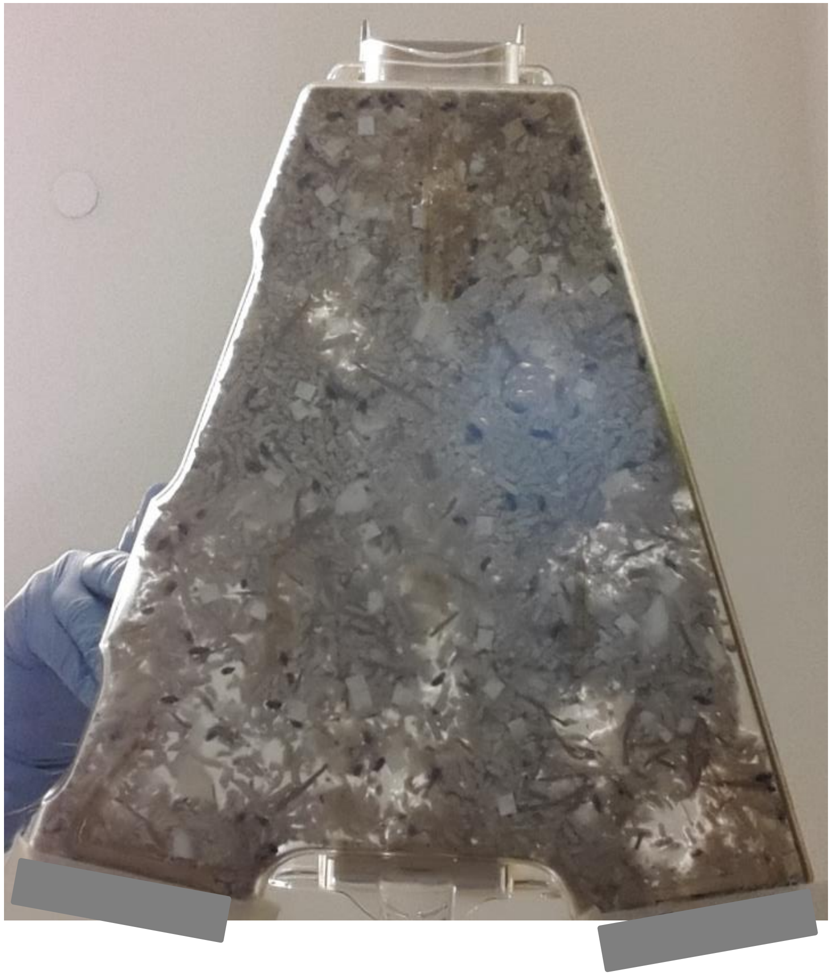

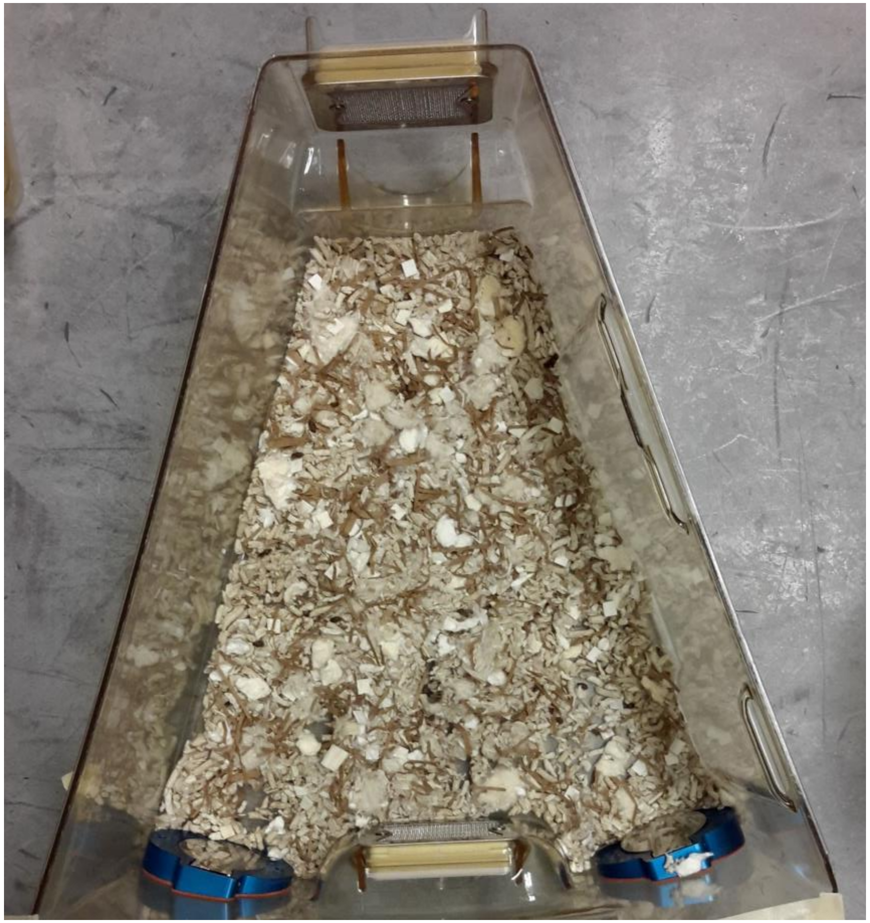

Aug 27 COMP 5 left

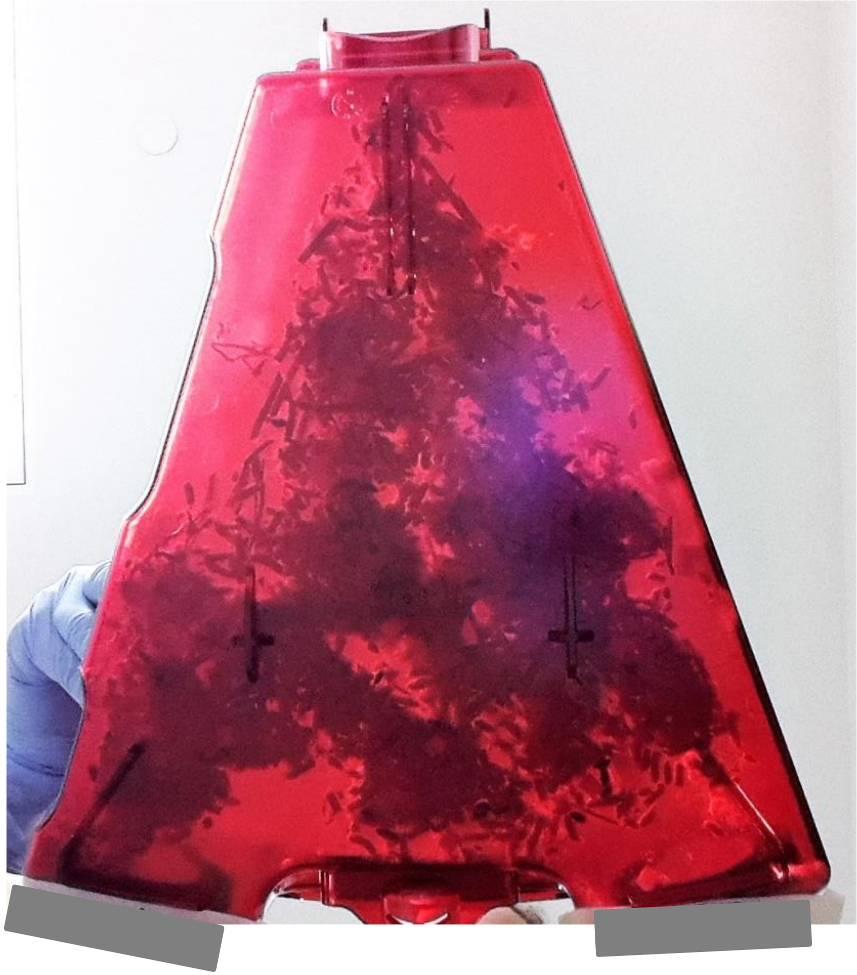

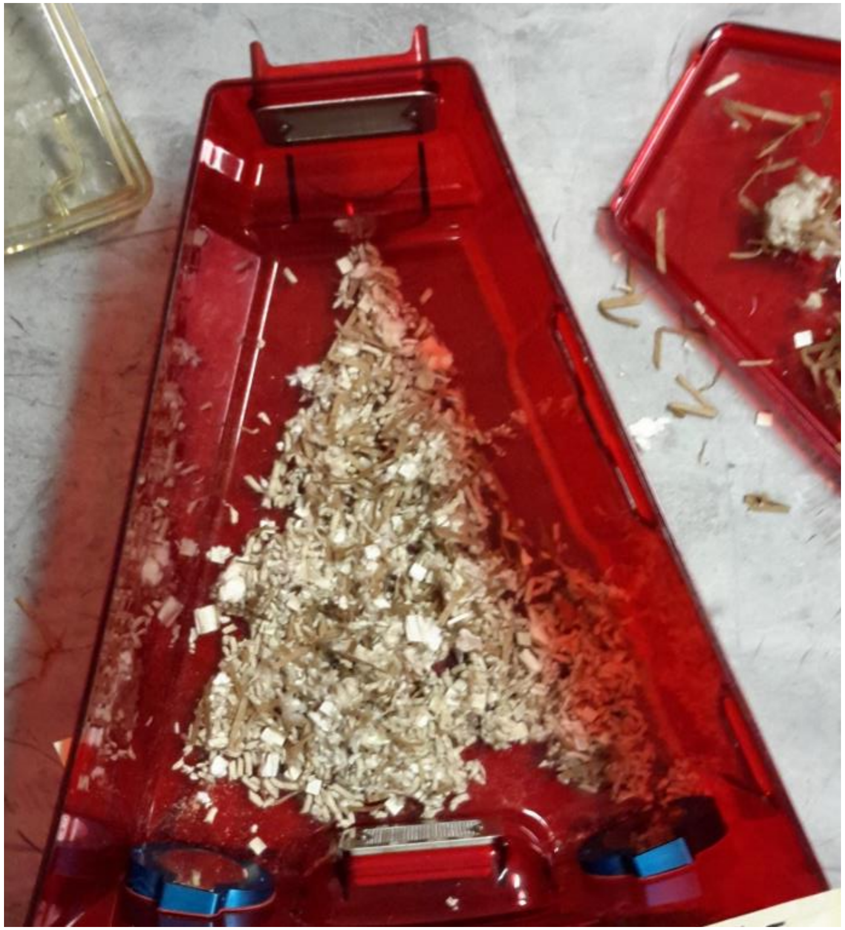

Aug 27 STD 5

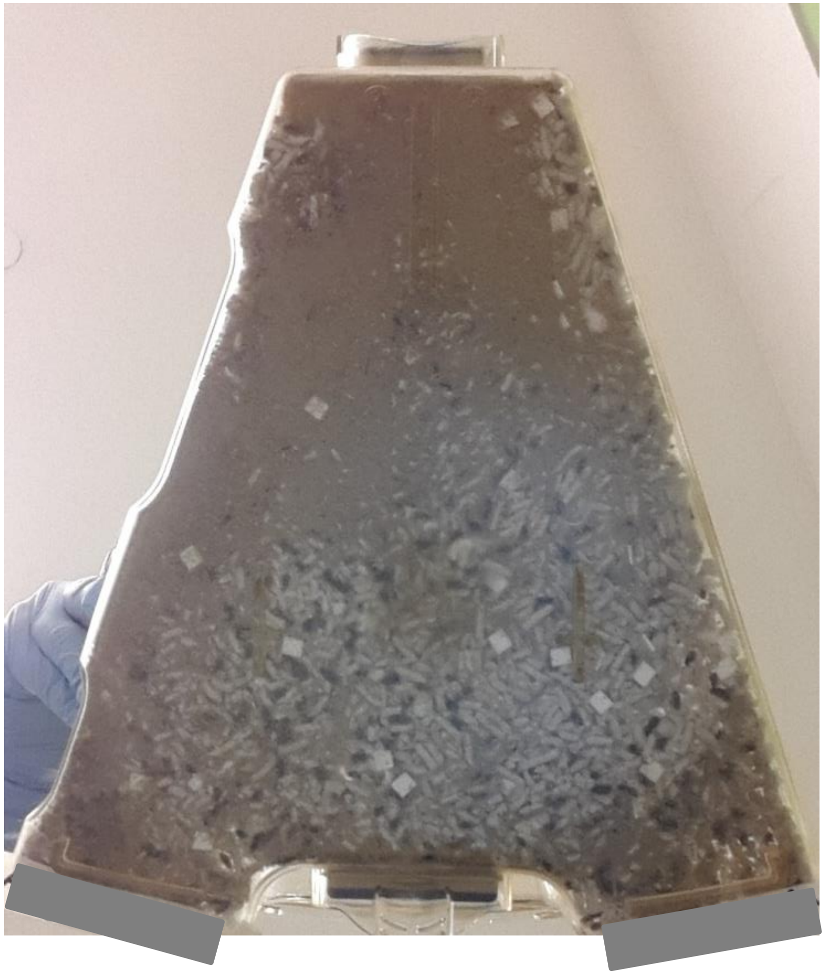

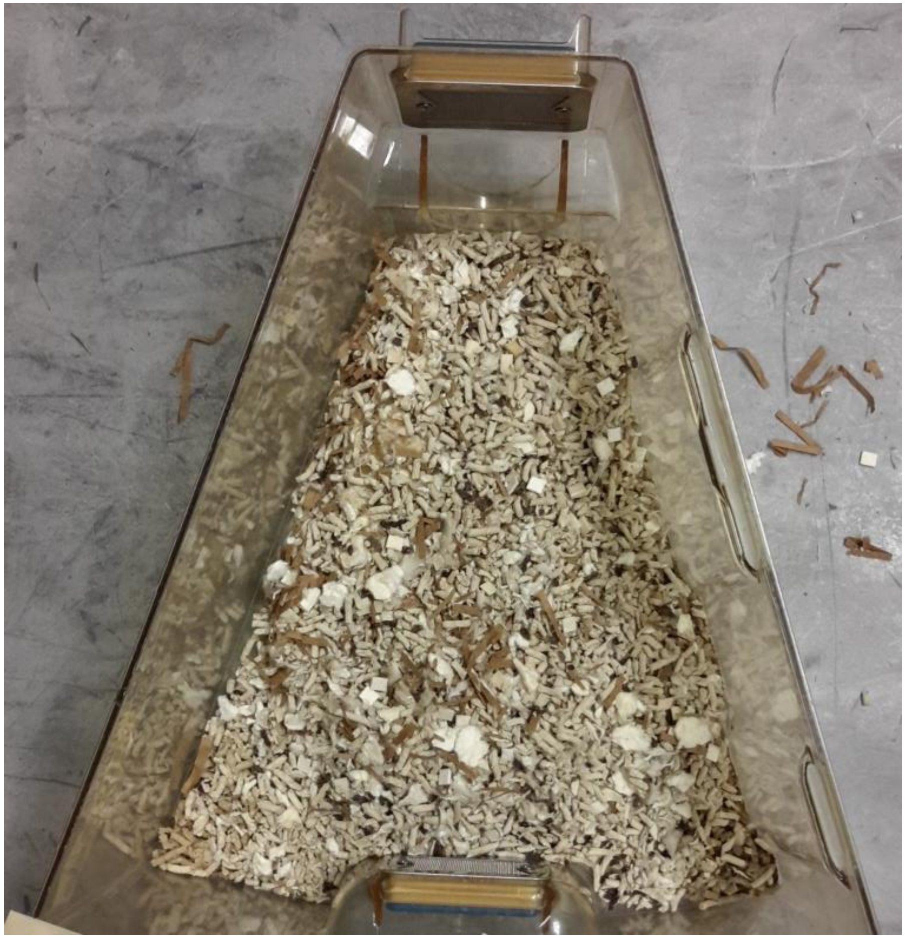

Sept 1 COMP 1 right

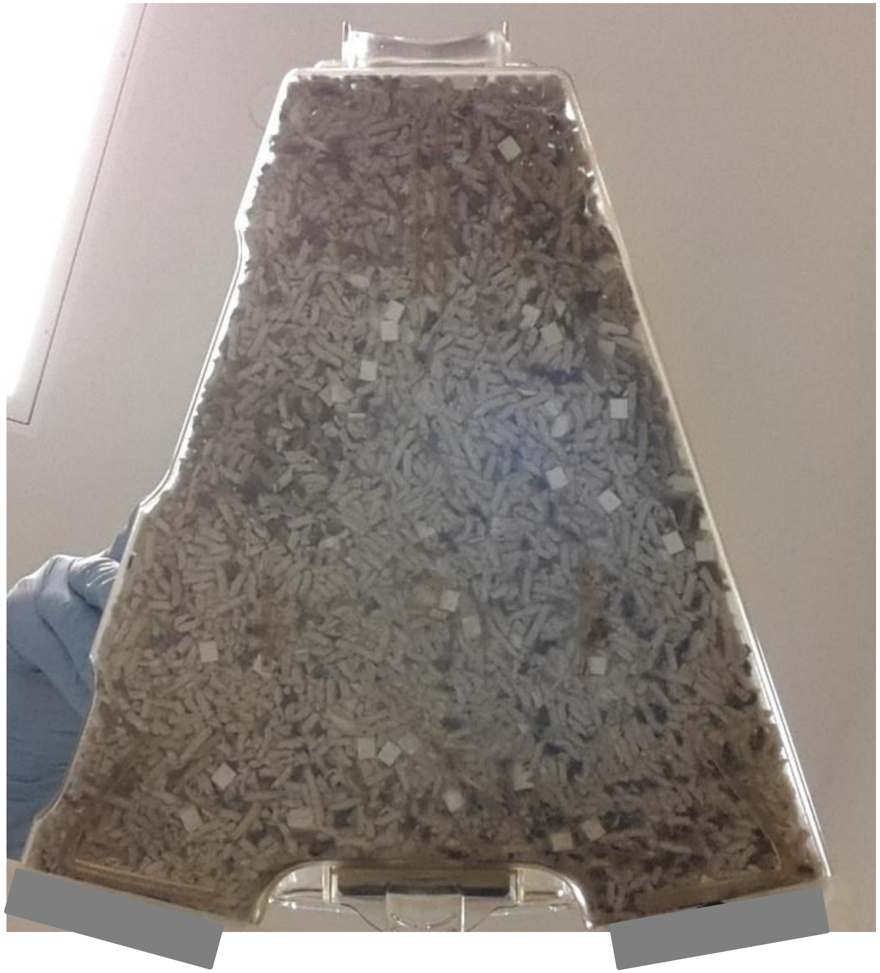

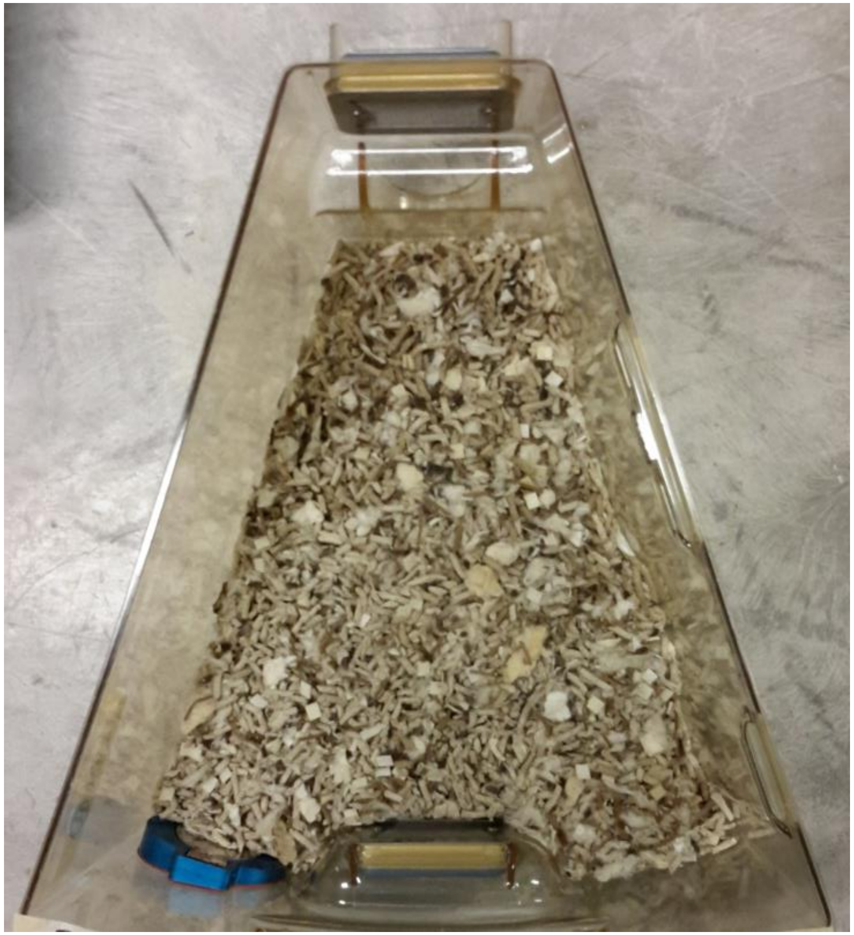

Sept 1 COMP 1 mid

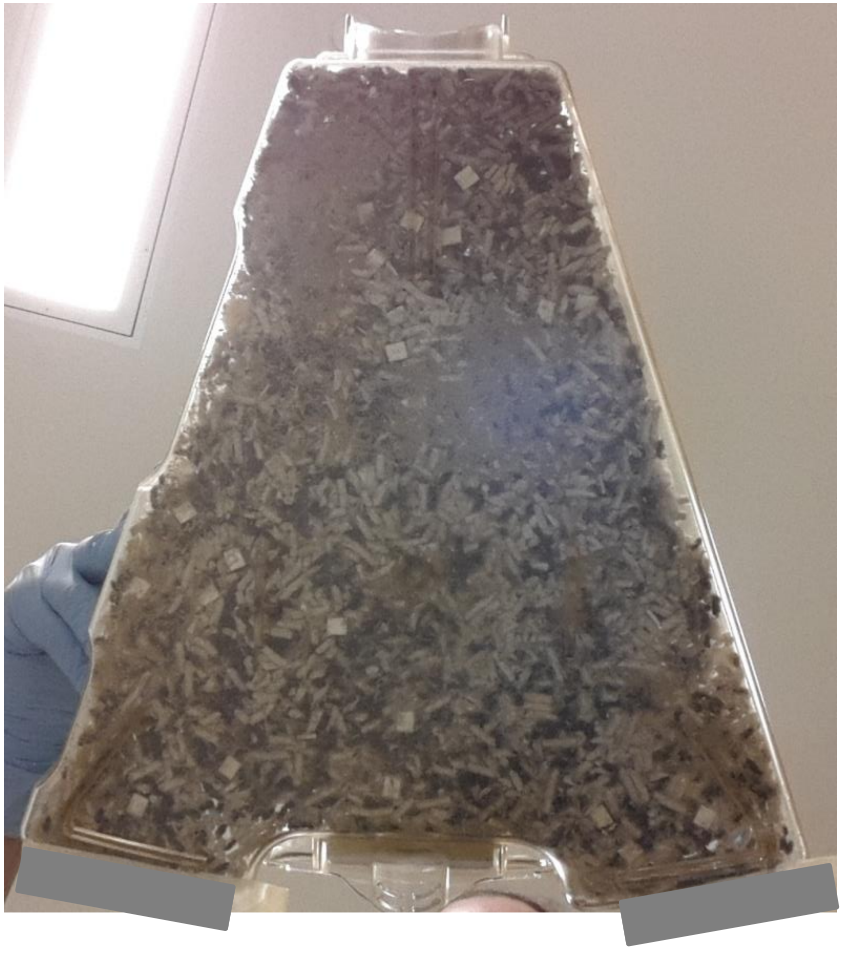

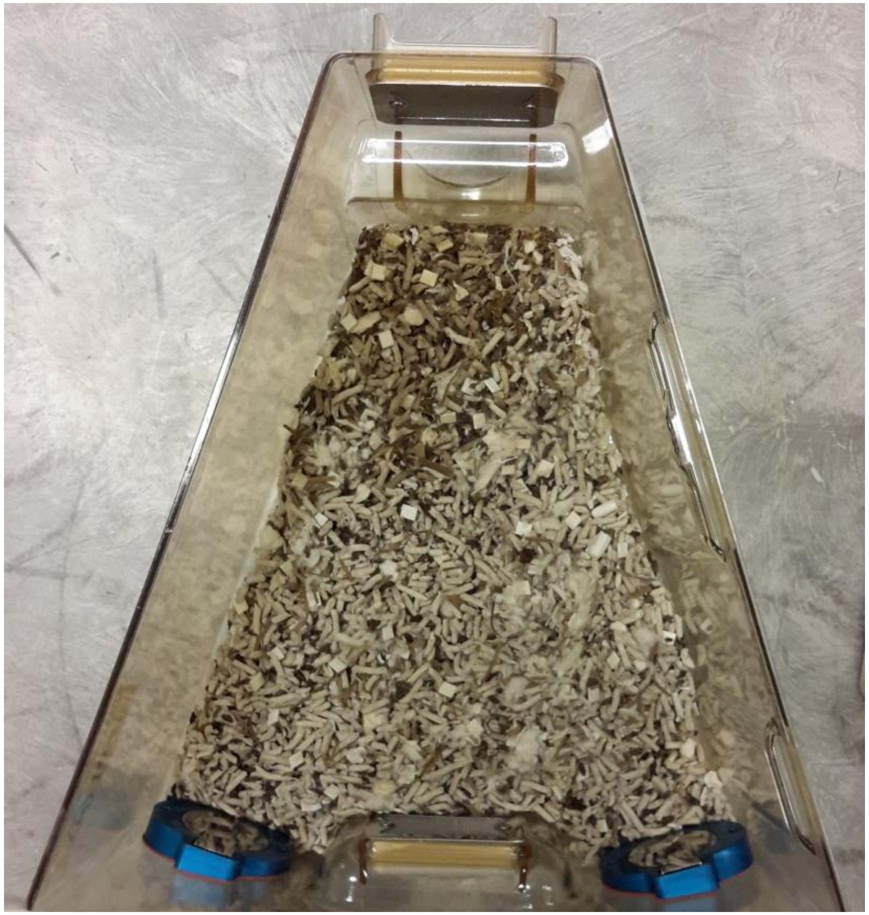

Sept 1 COMP 1 left

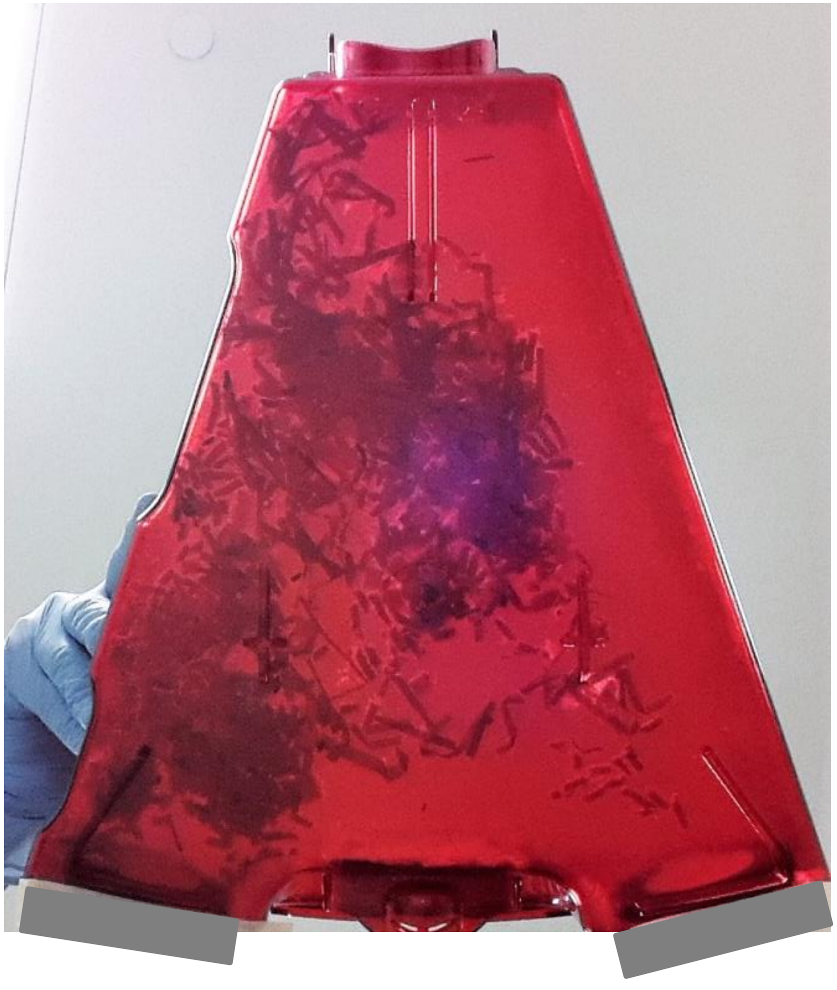

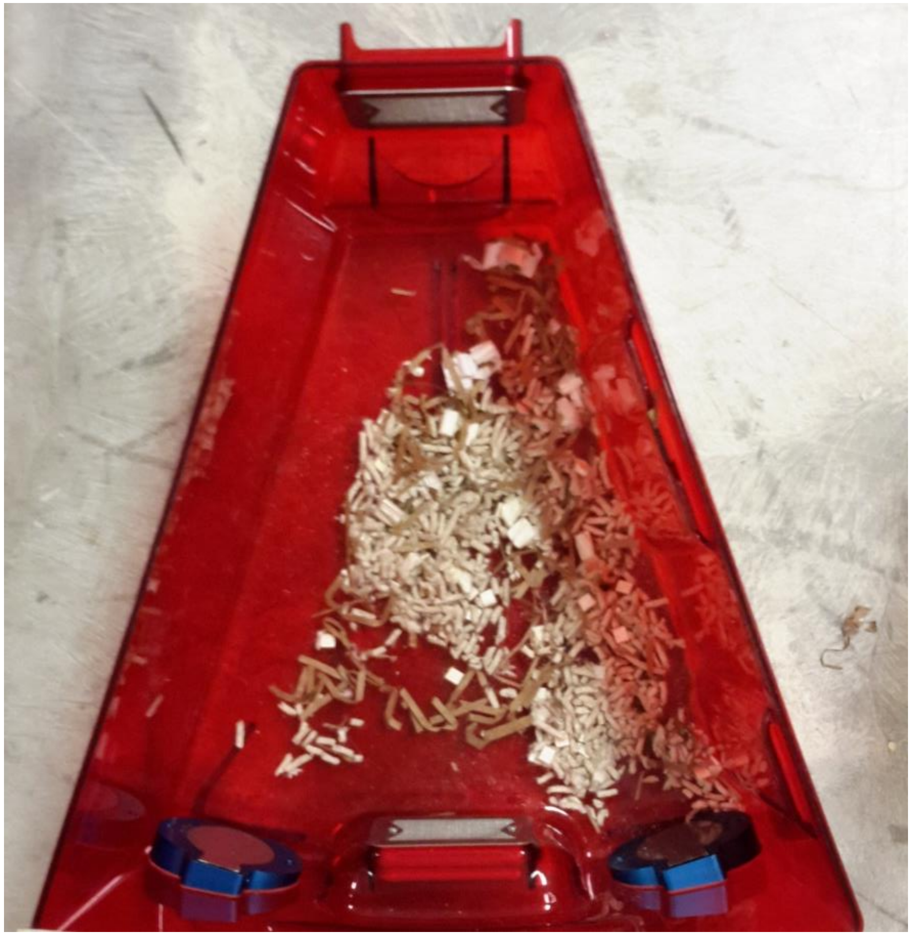

Sept 1 STD 1

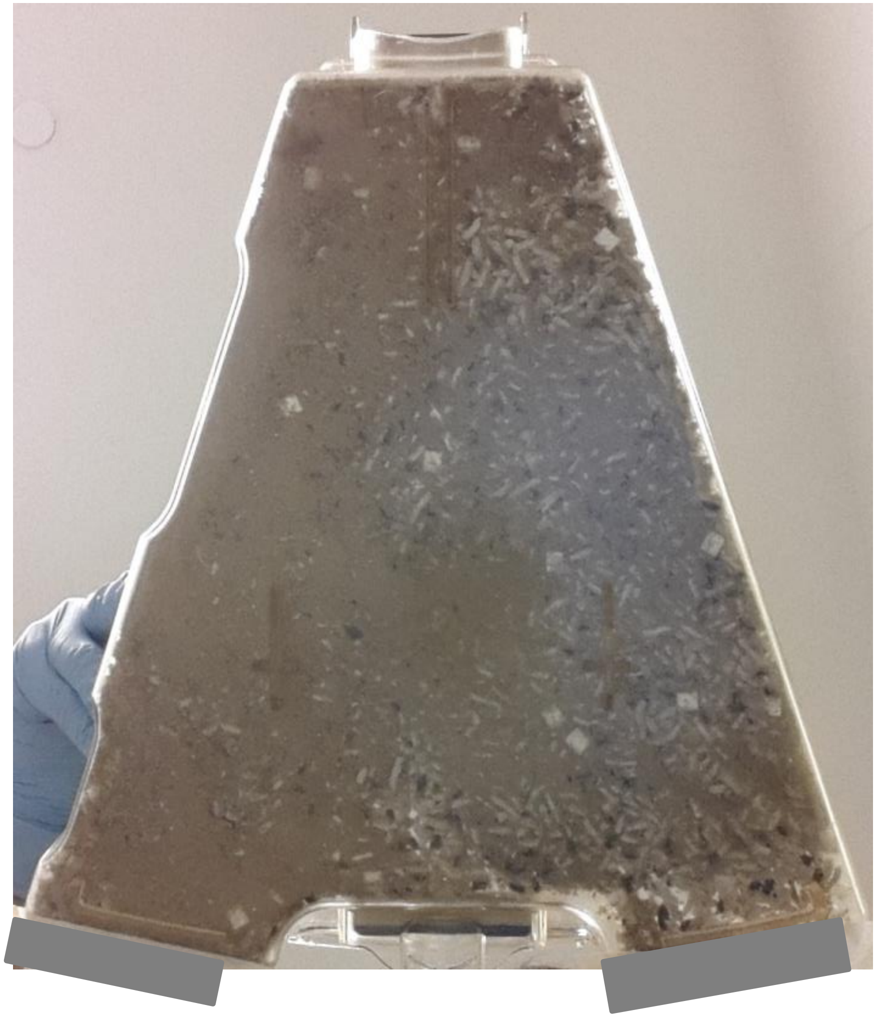

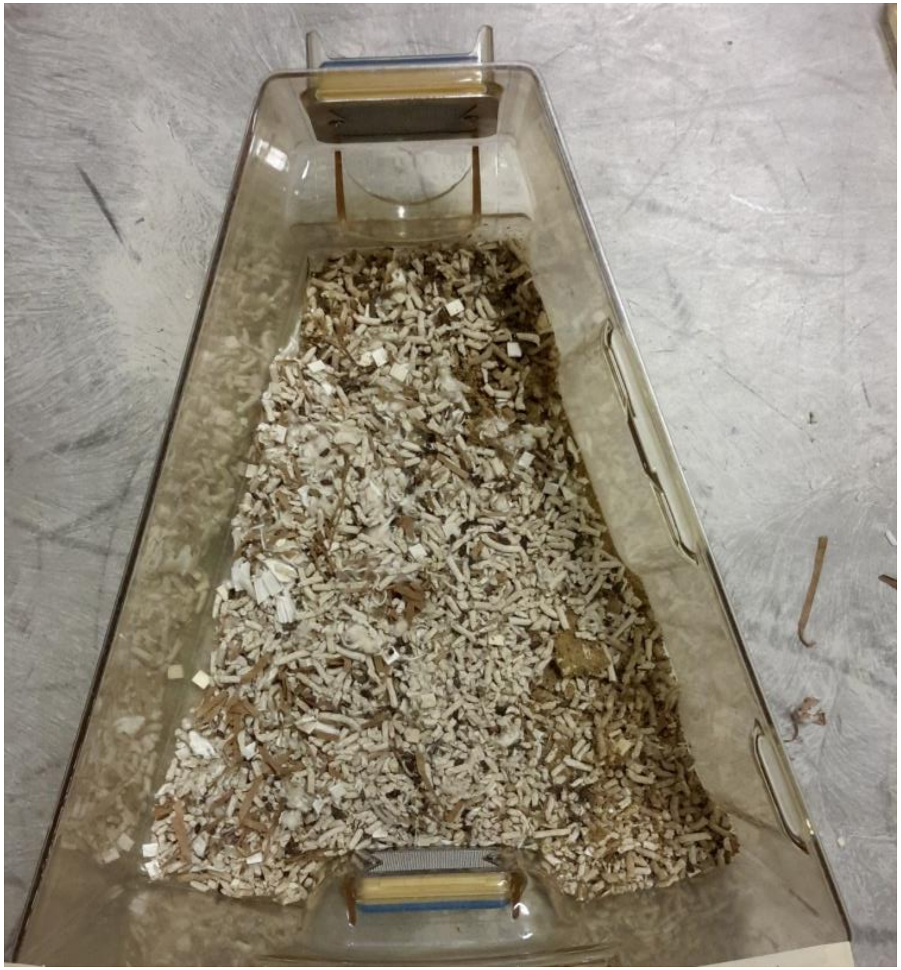

Sept 1 COMP 2 right

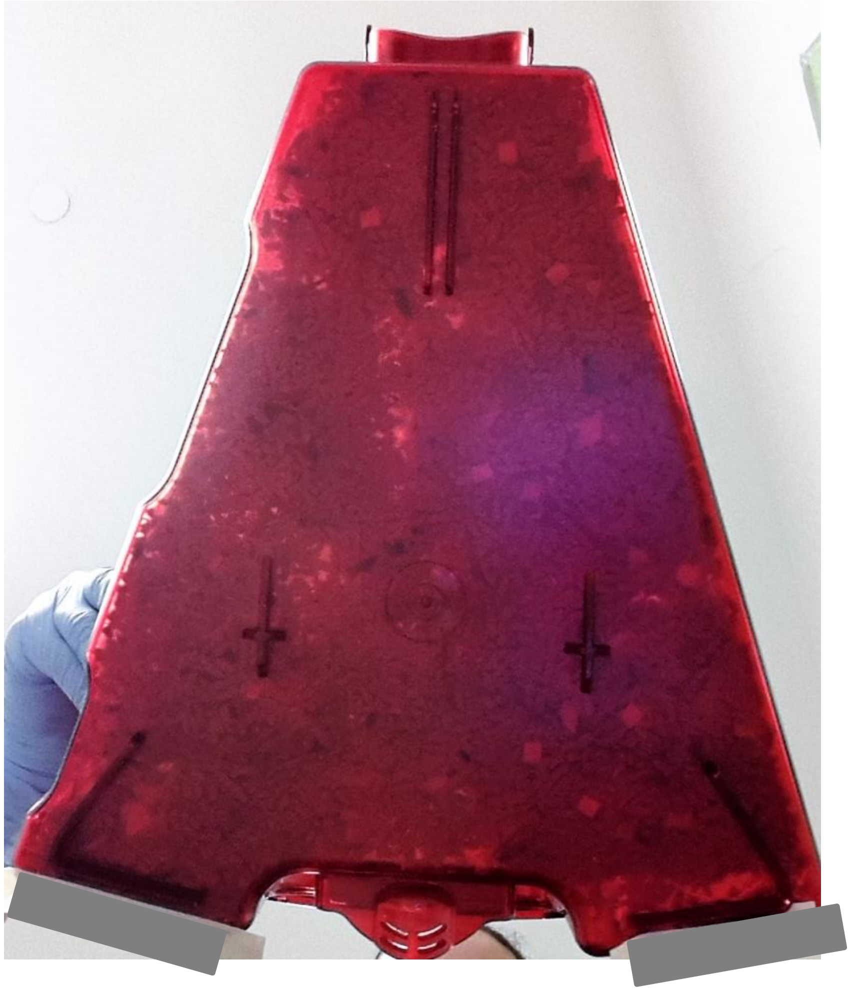

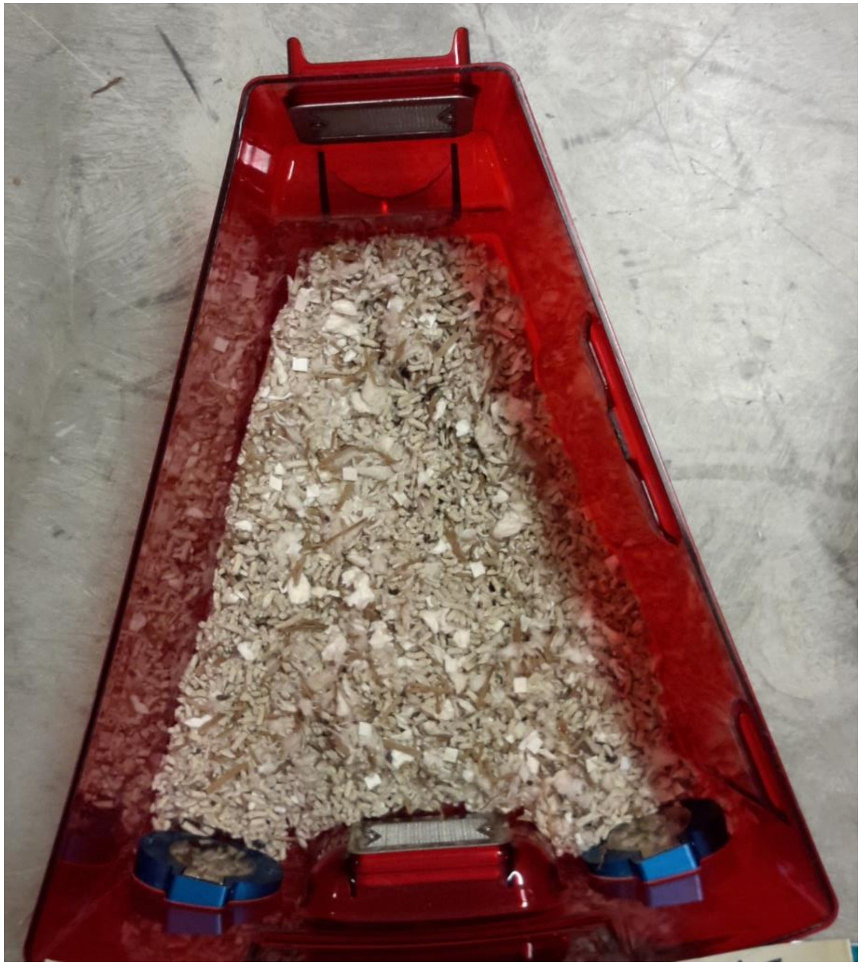

Sept 1 COMP 2 mid

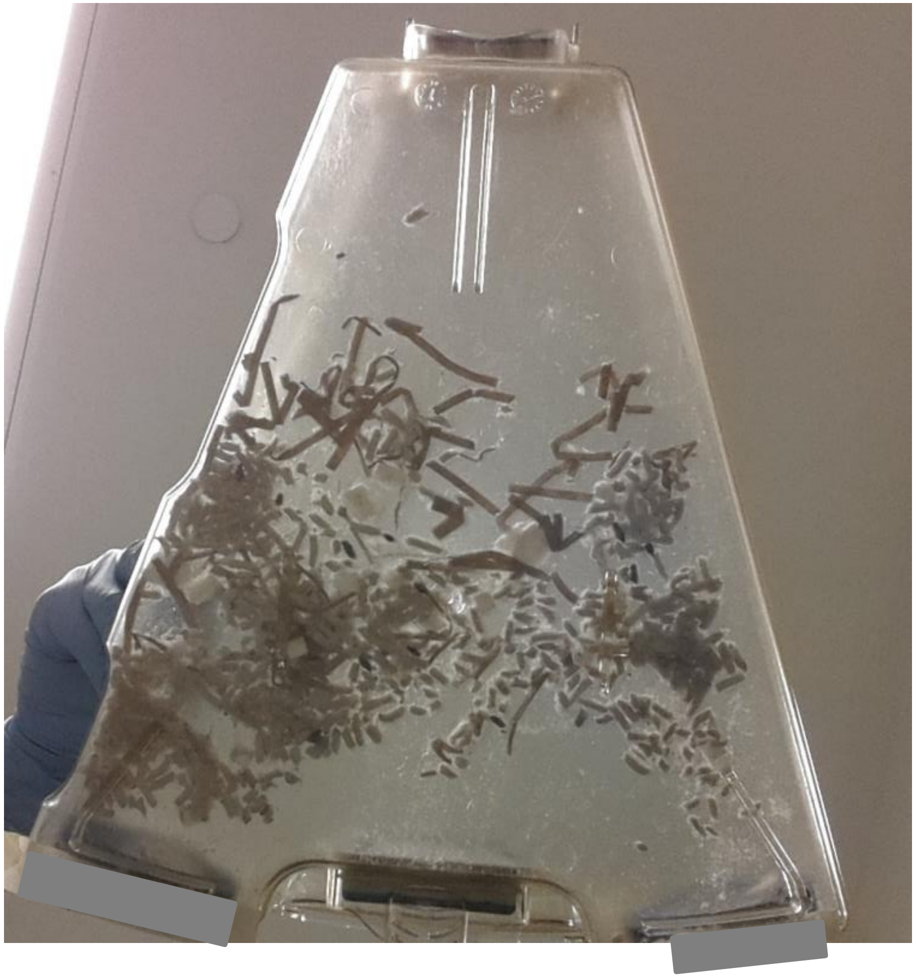

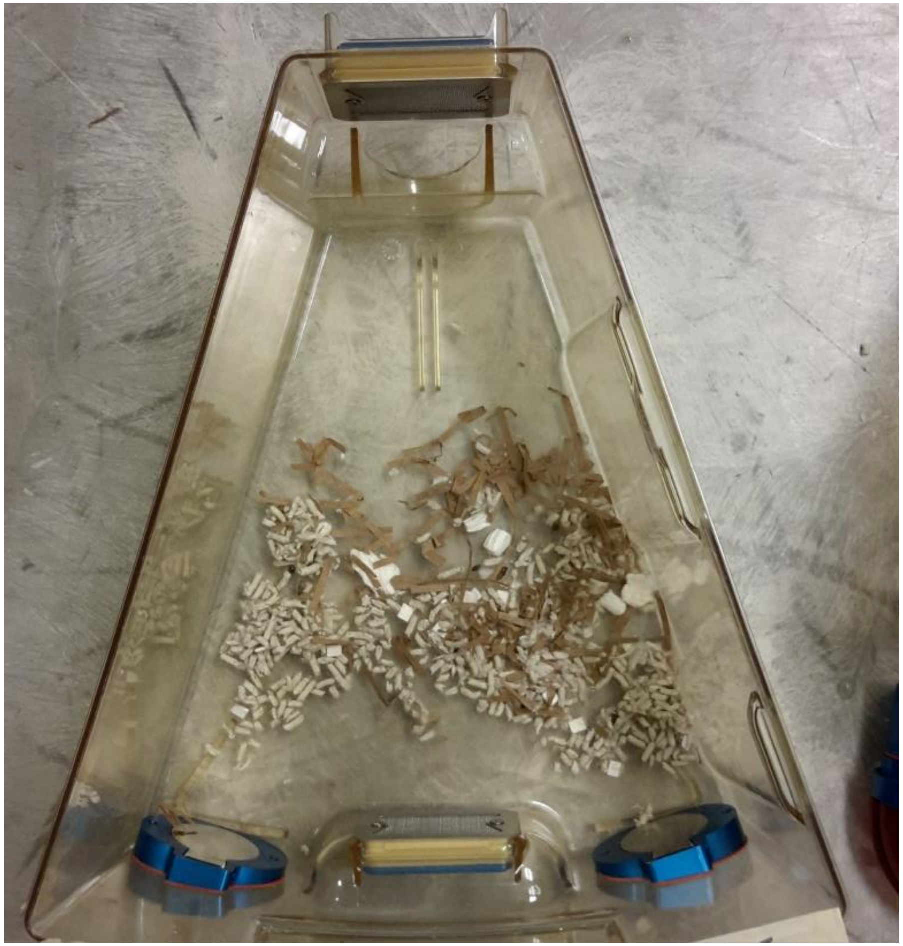

Sept 1 COMP 2 left

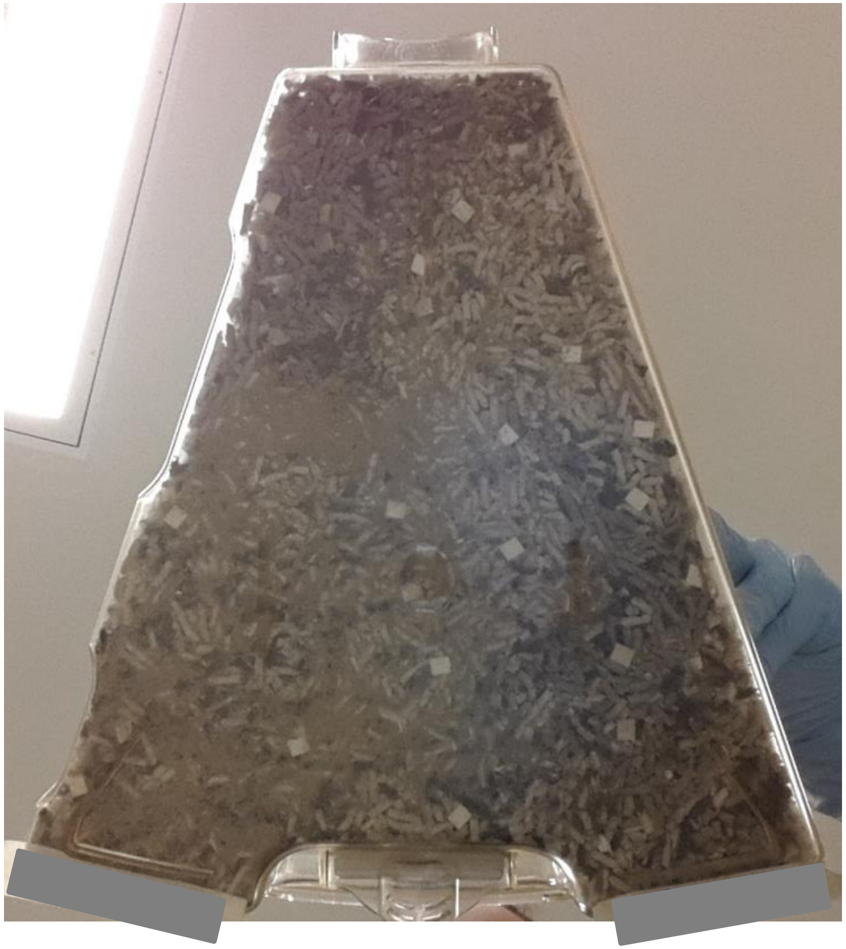

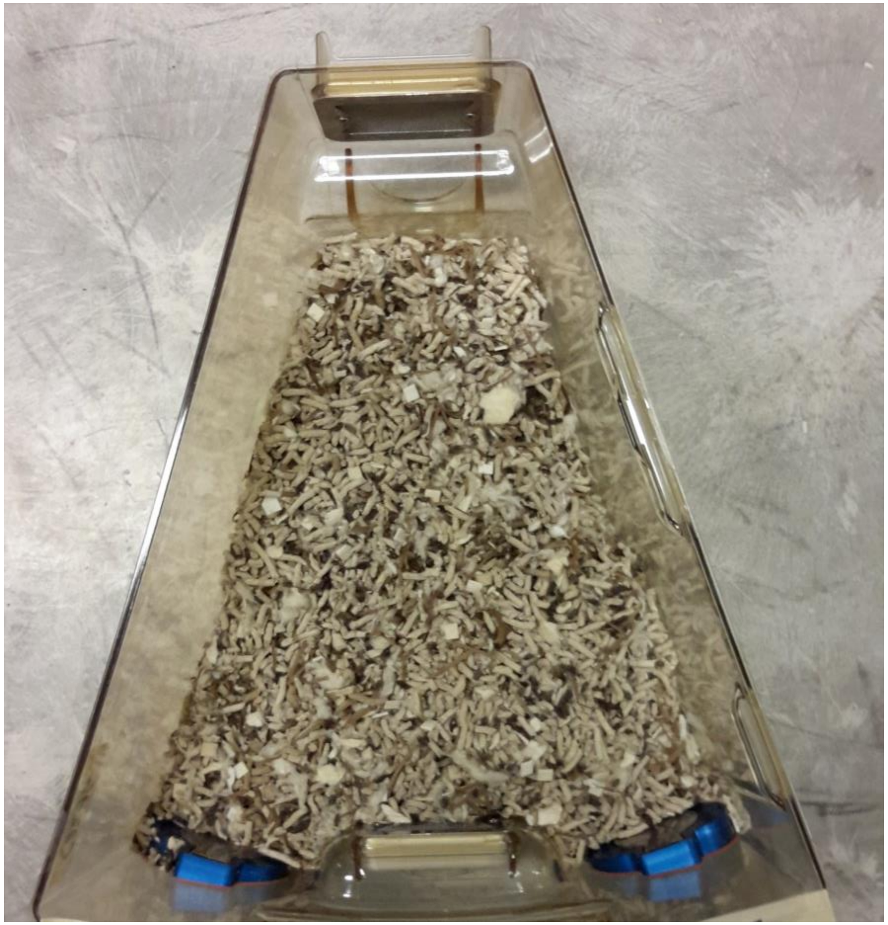

Sept 1 STD 2

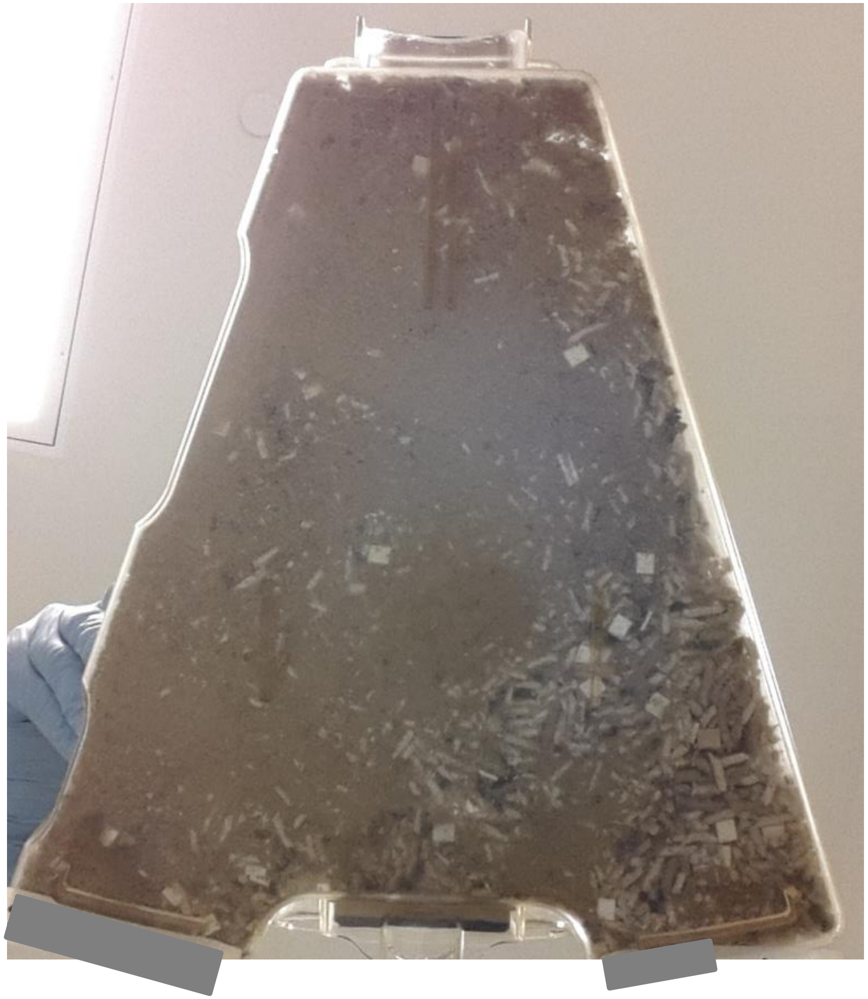

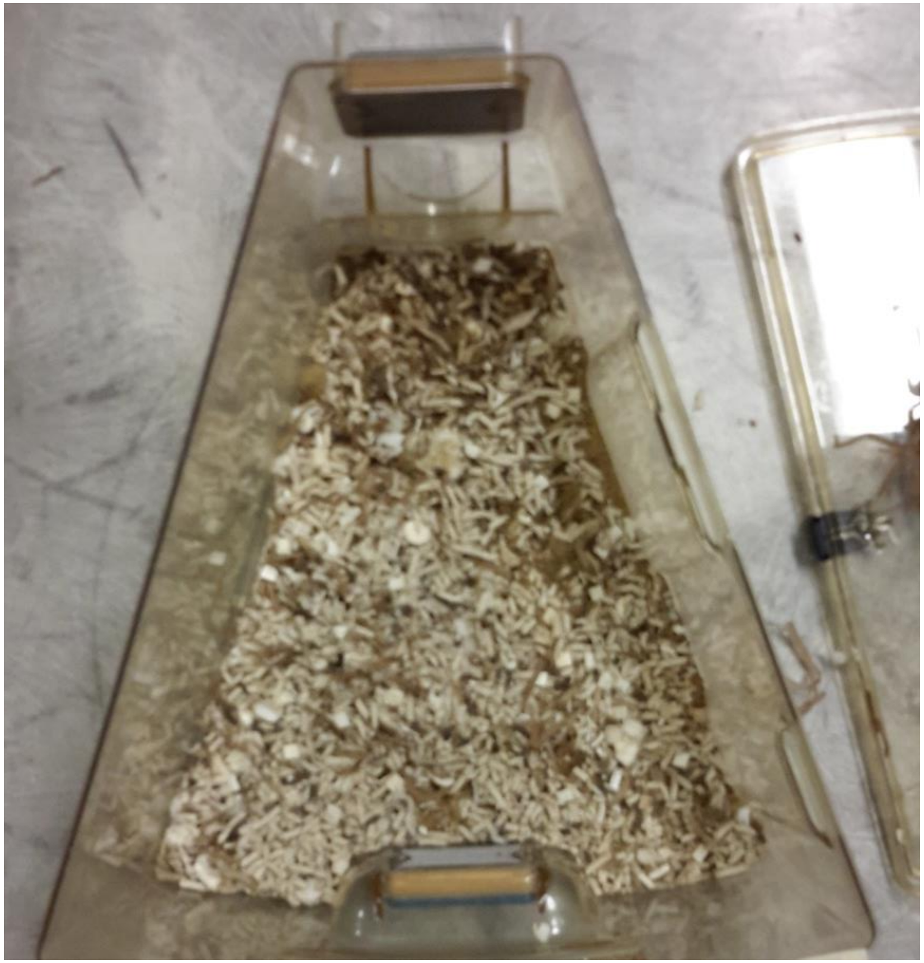

Sept 2 COMP 3 right

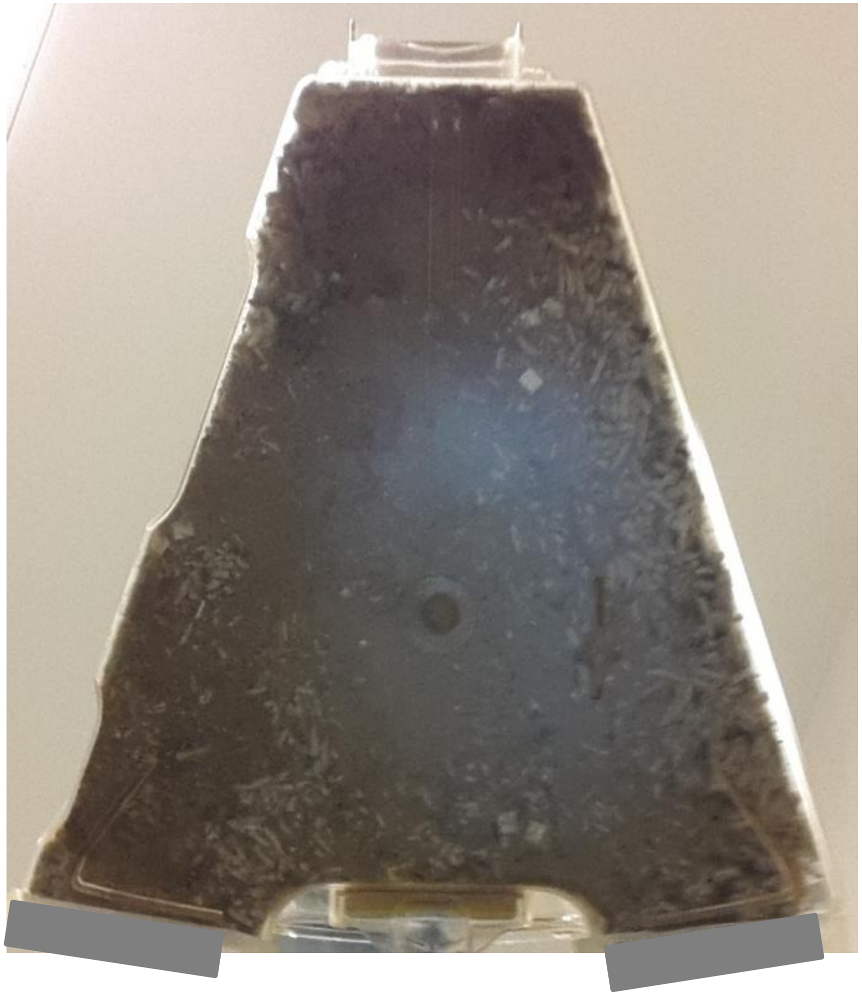

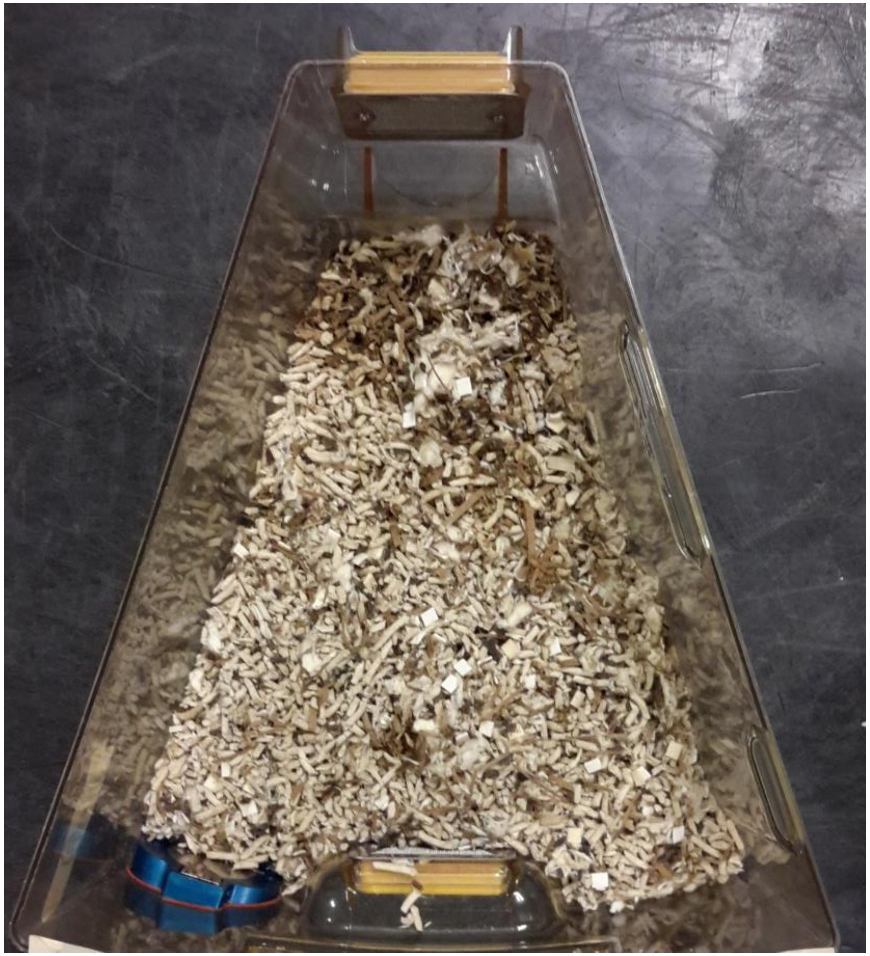

Sept 2 COMP 3 mid

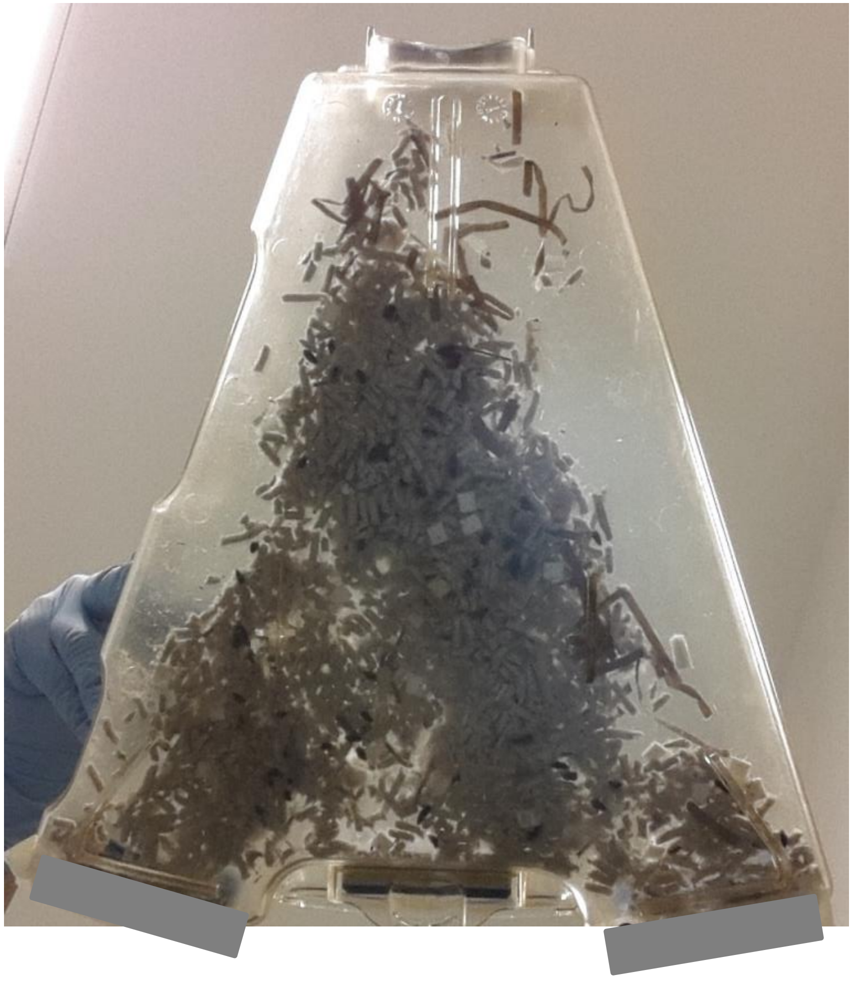

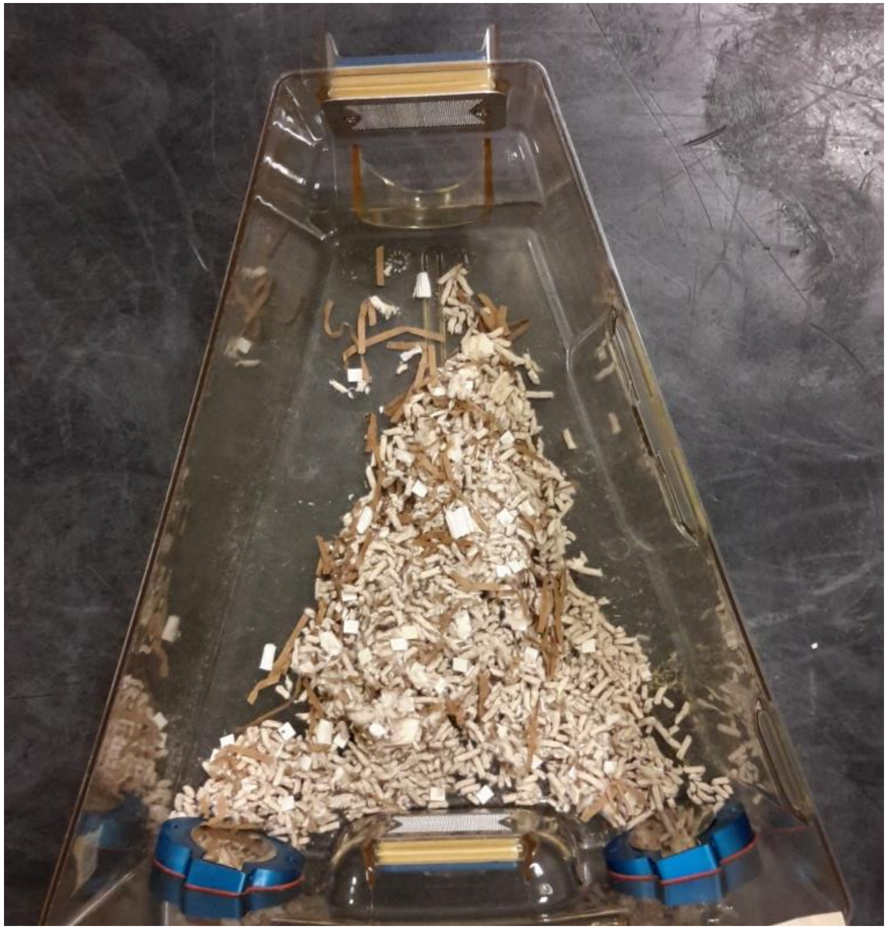

Sept 2 COMP 3 left

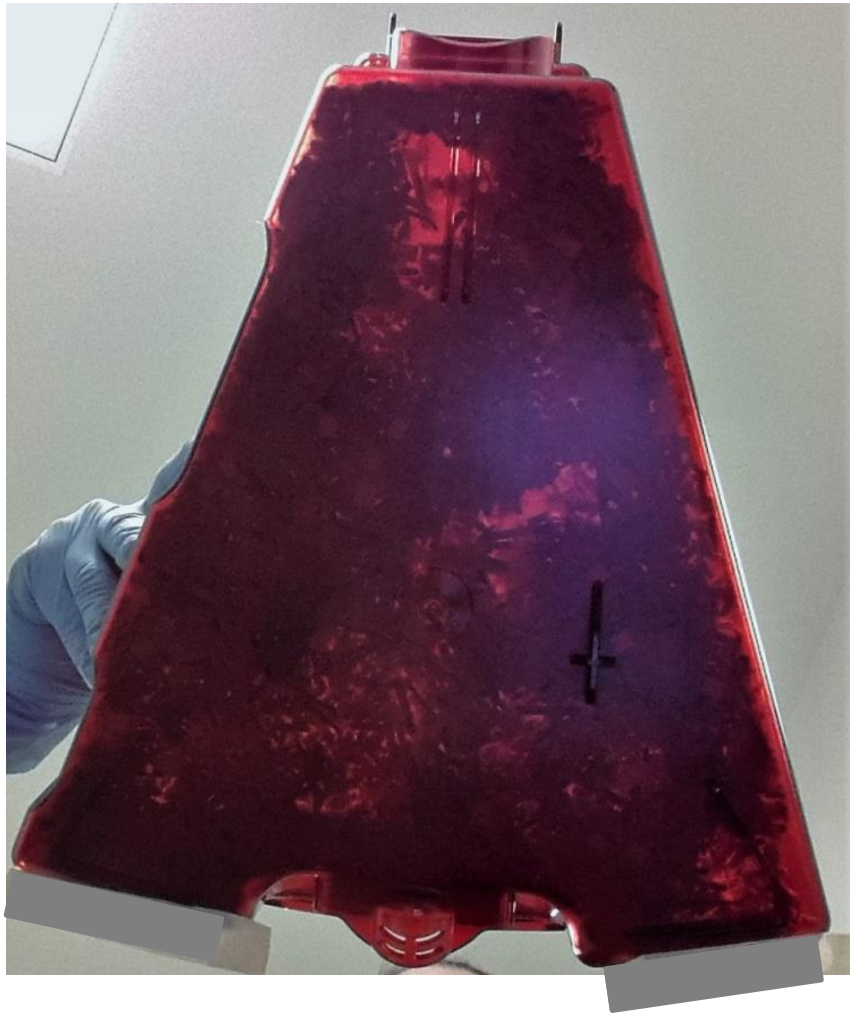

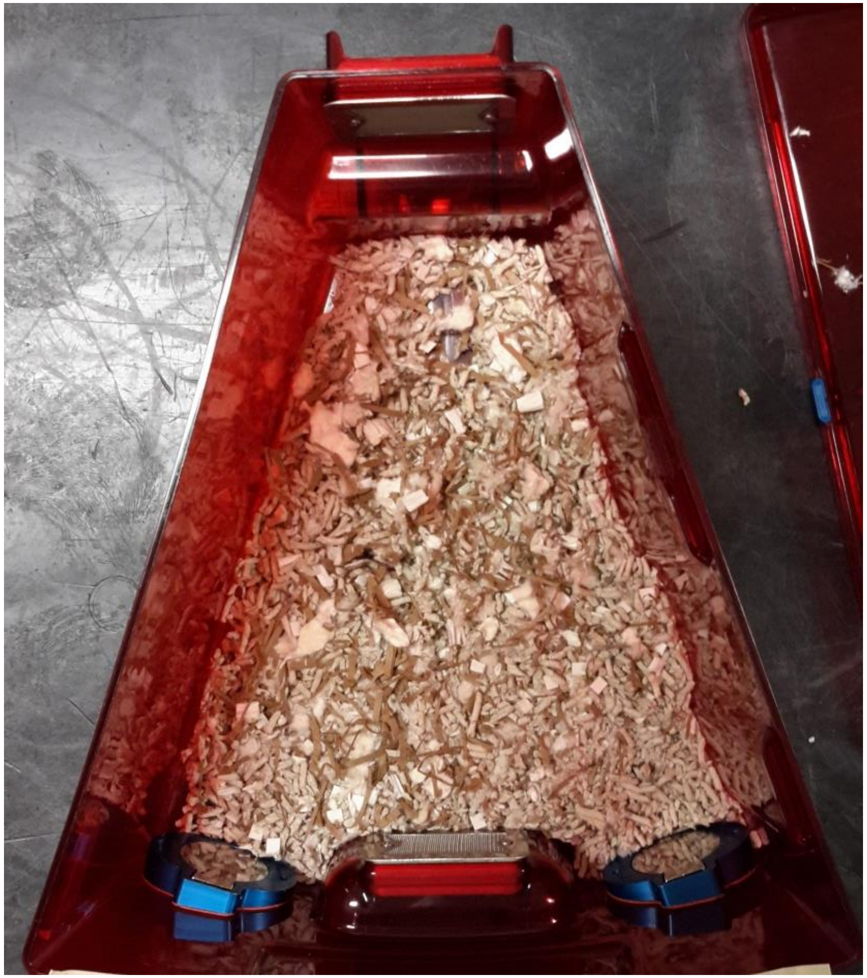

Sept 2 STD 3

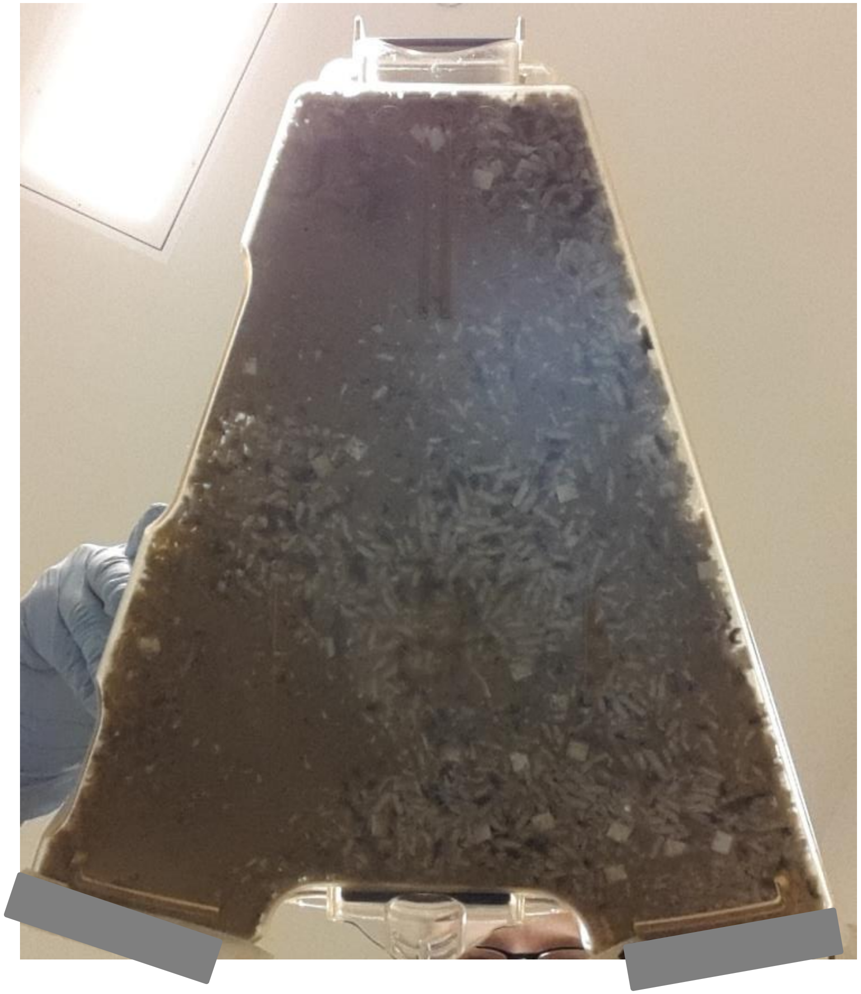

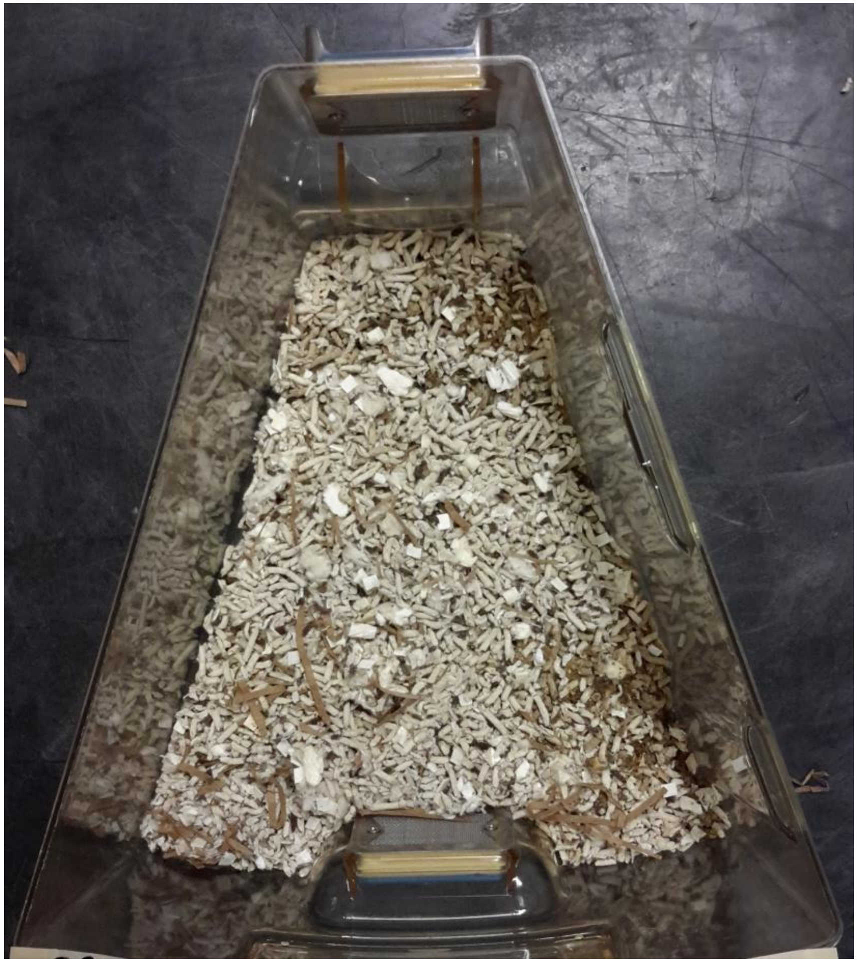

Sept 2 COMP 4 mid

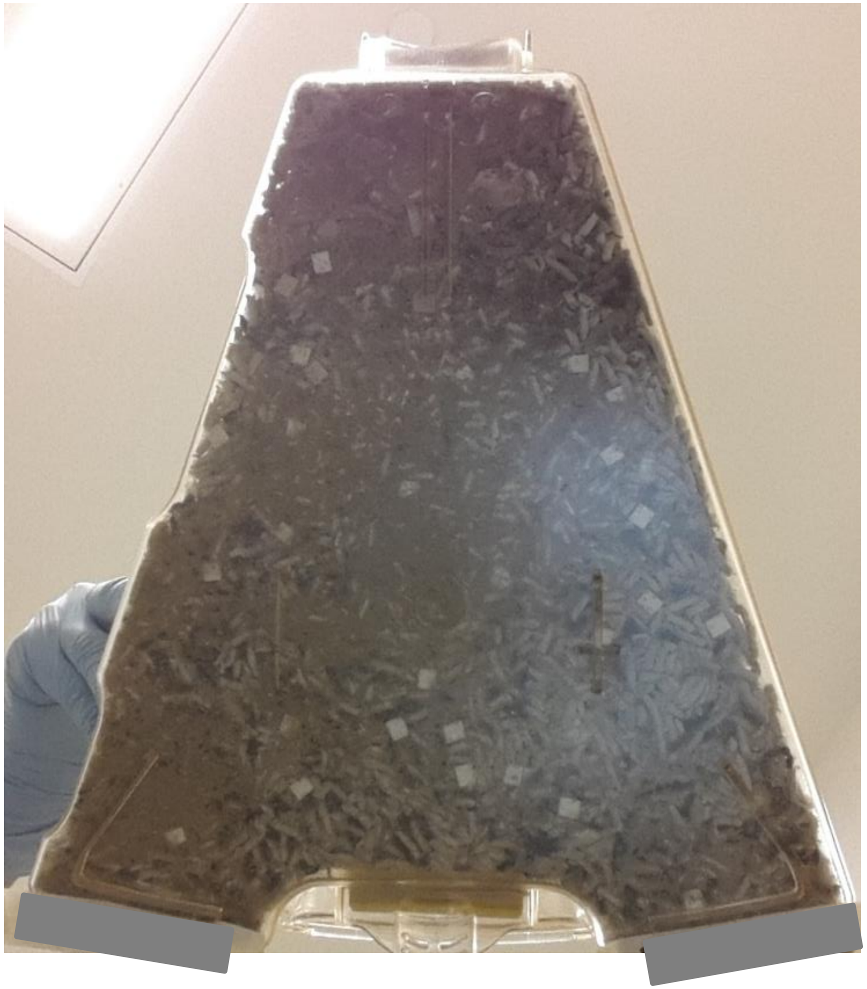

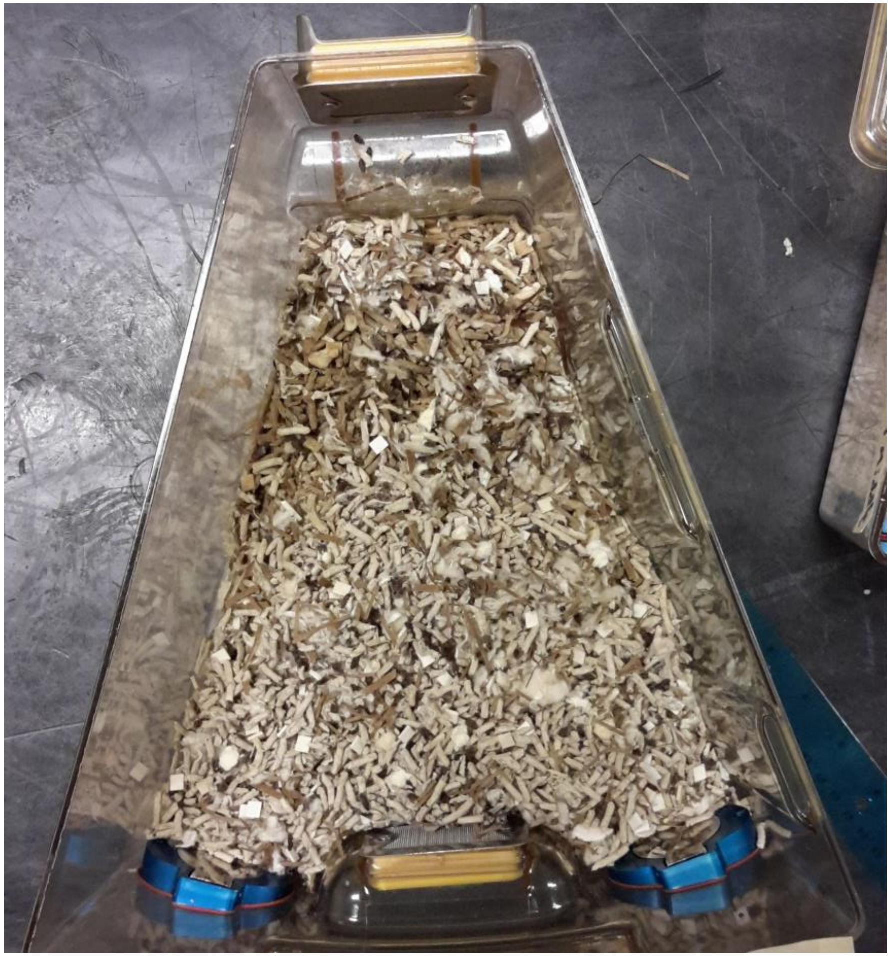

Sept 2 COMP 4 left

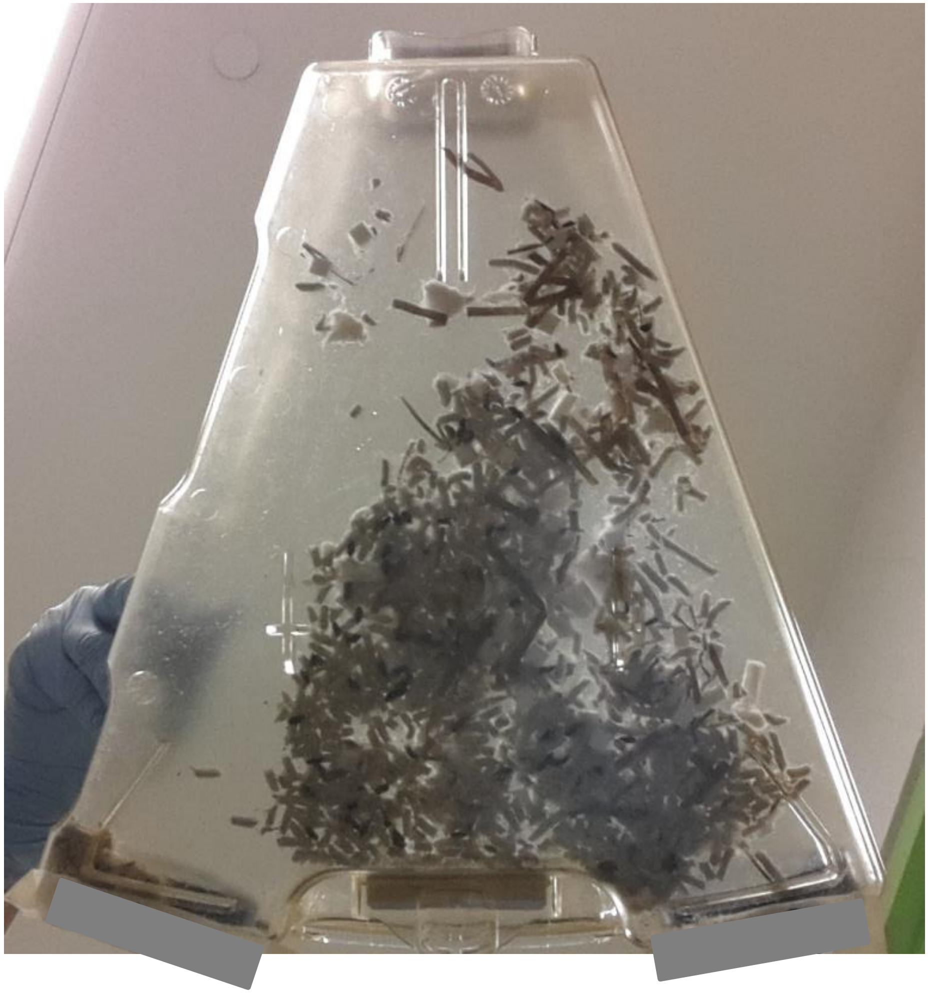

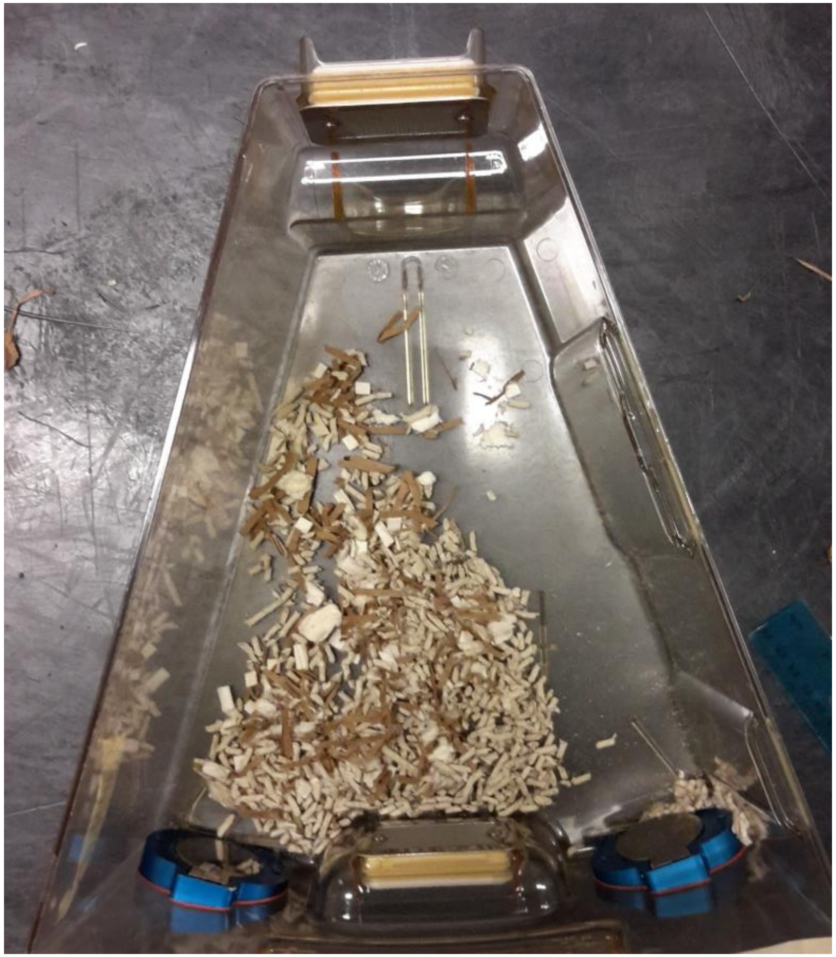

Sept 2 COMP 4 right

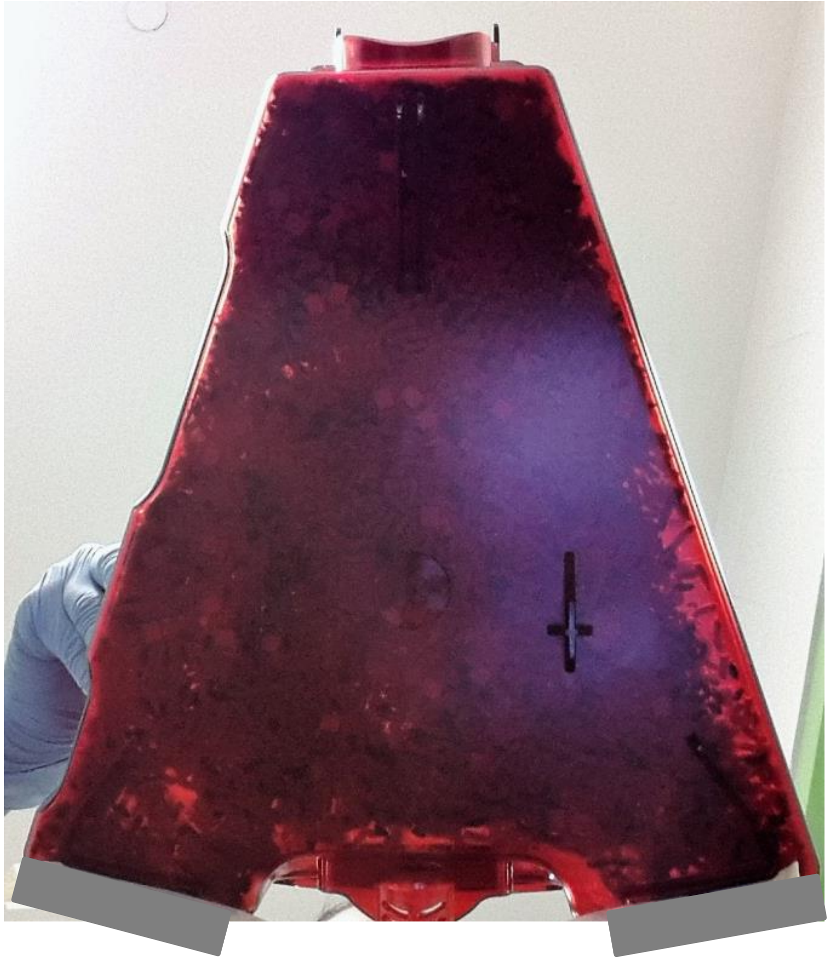

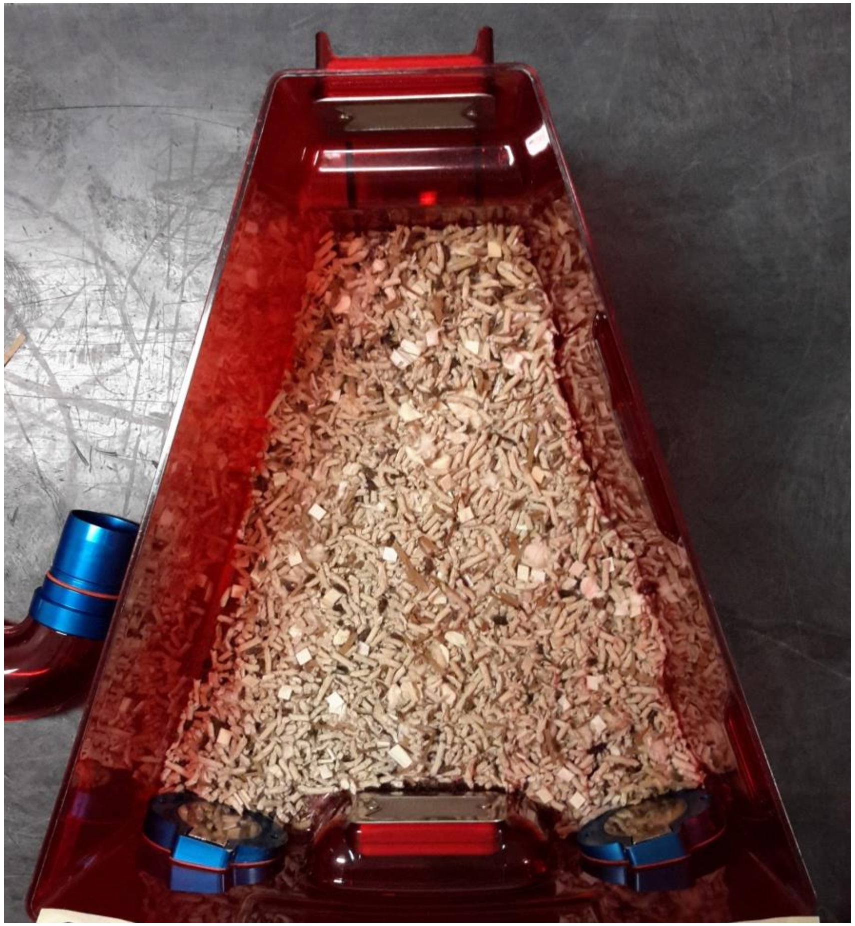

Sept 2 STD 4

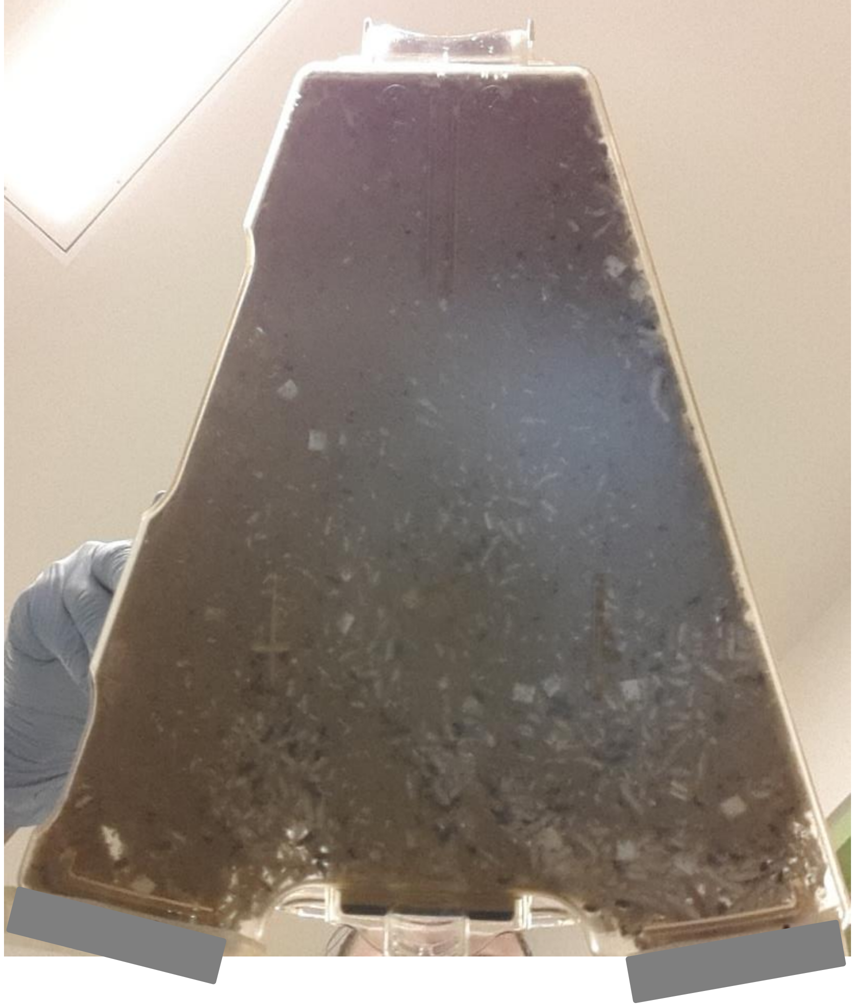

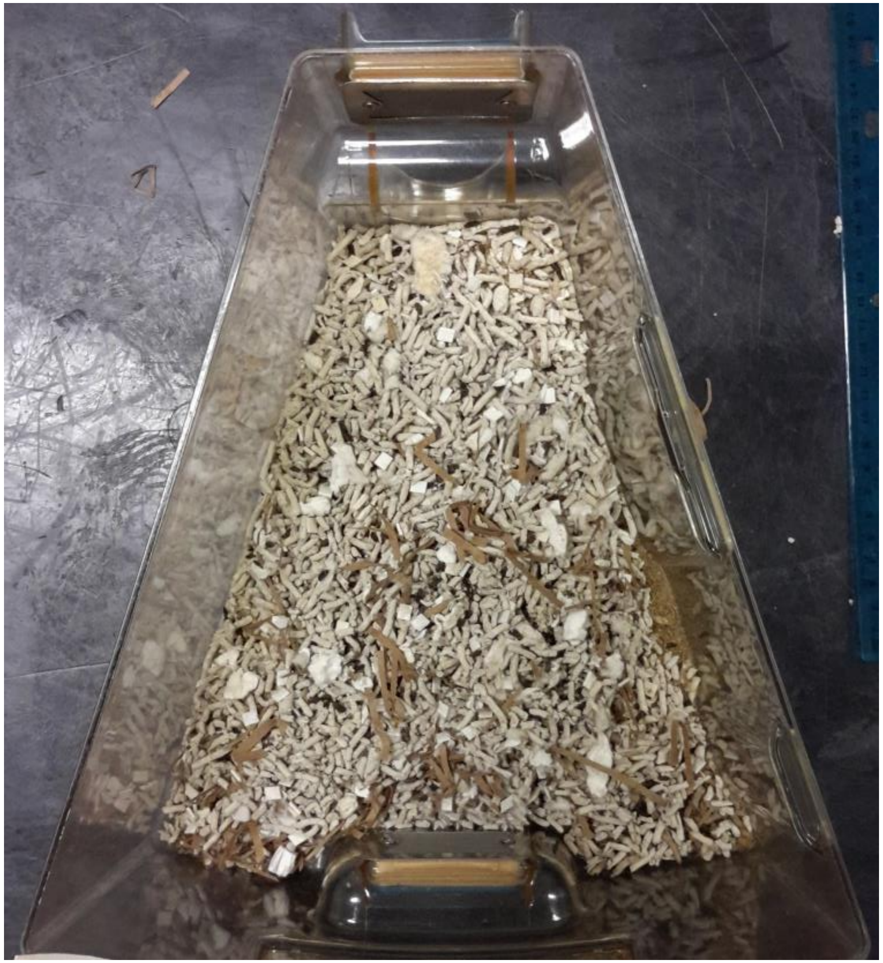

Sept 3 COMP 5 left

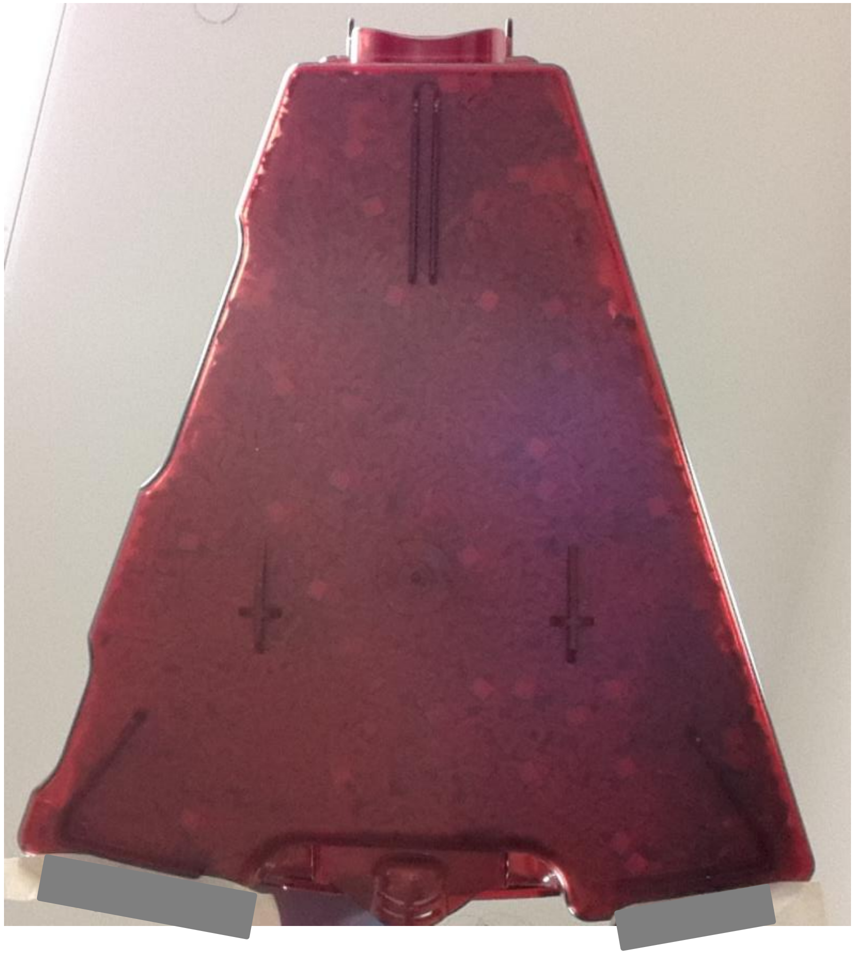

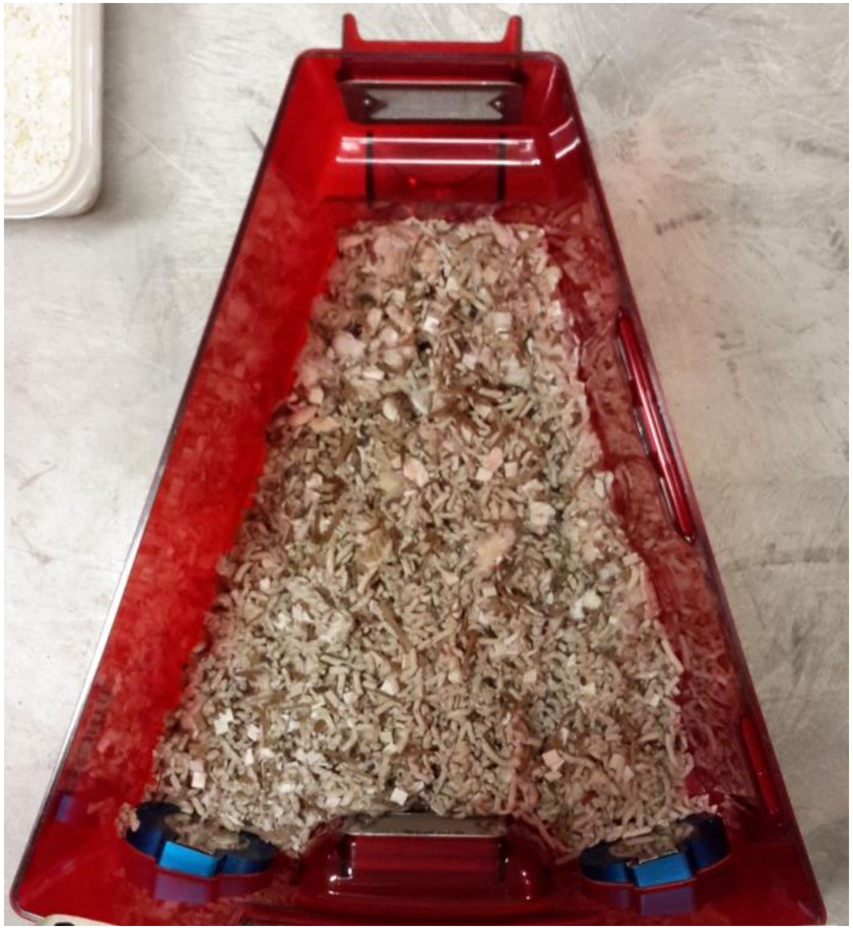

Sept 3 COMP 5 mid

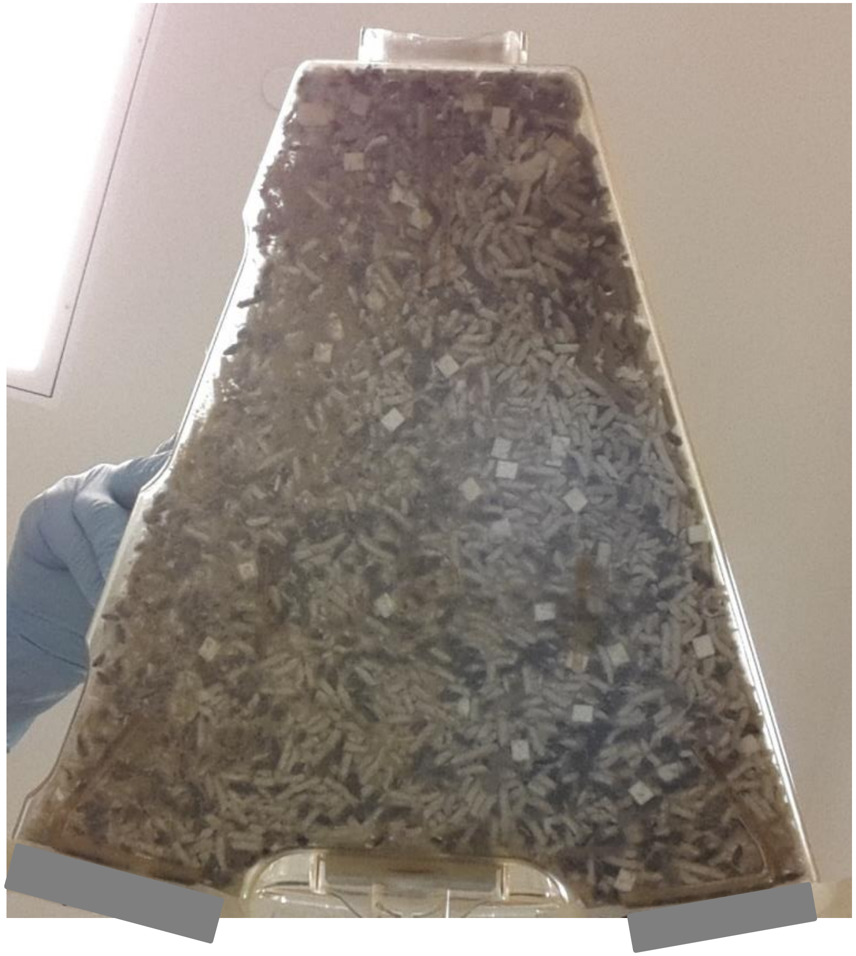

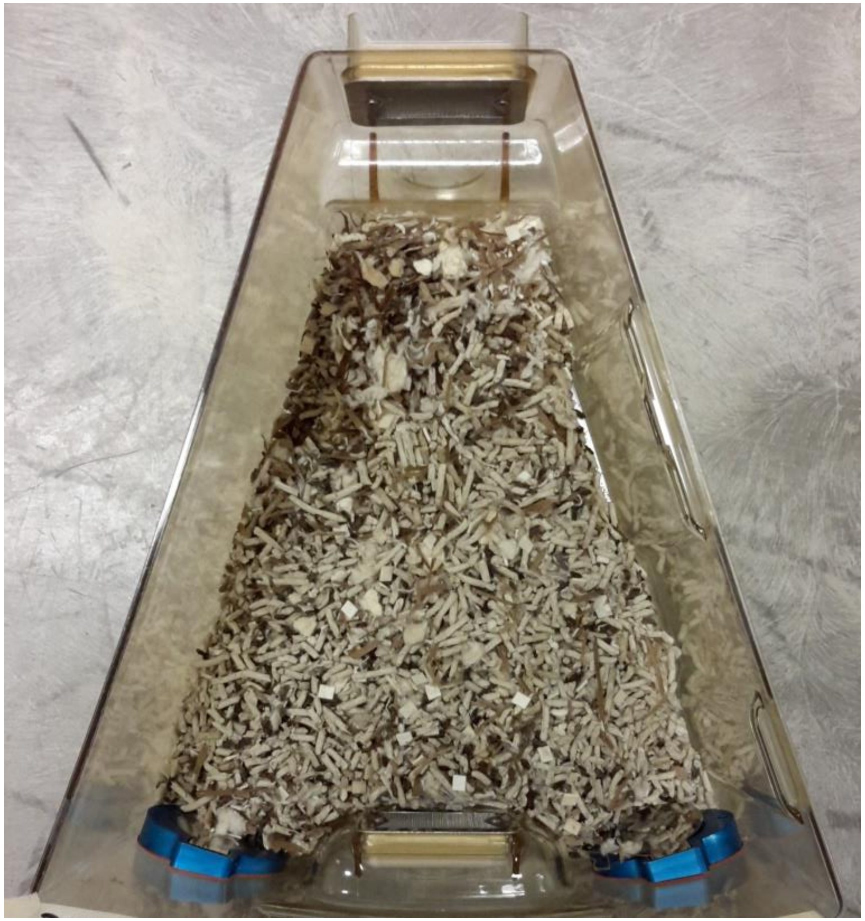

Sept 3 COMP 5 right

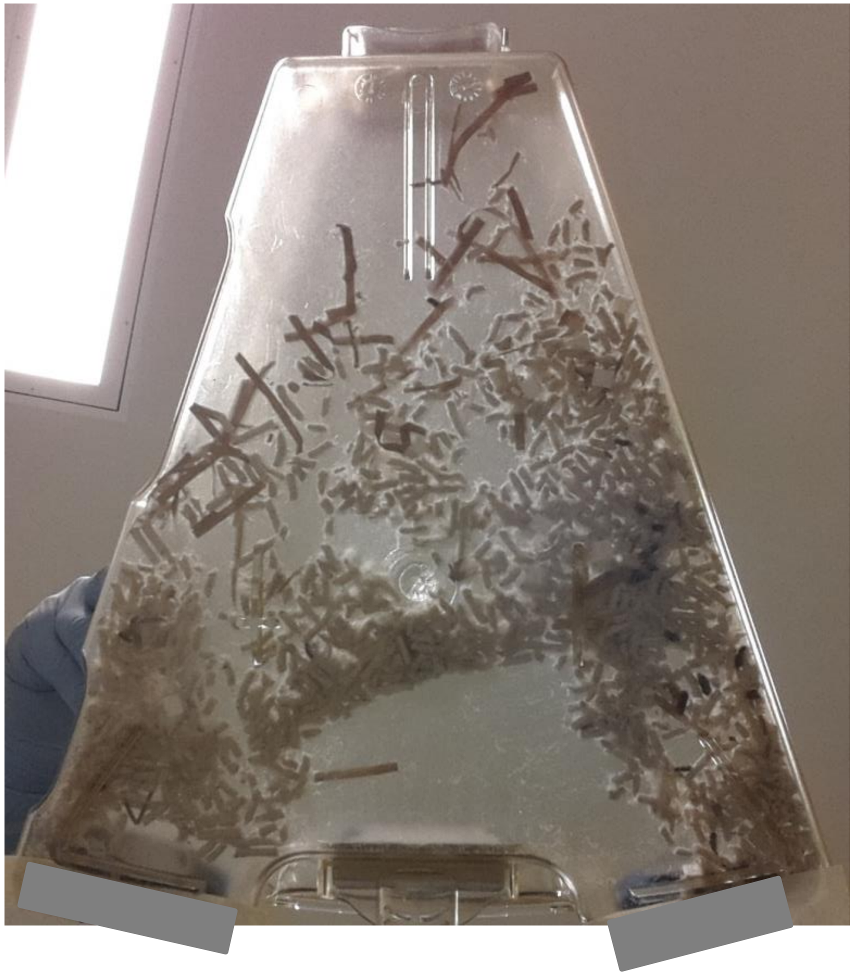

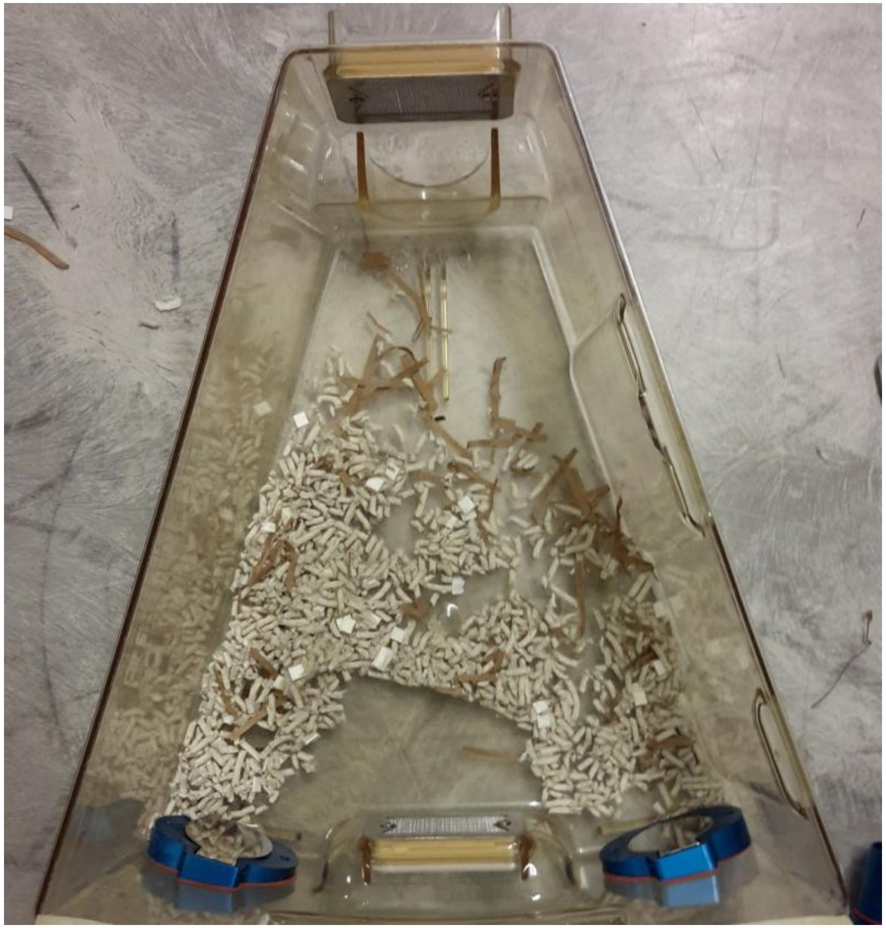

Sept 3 STD 5

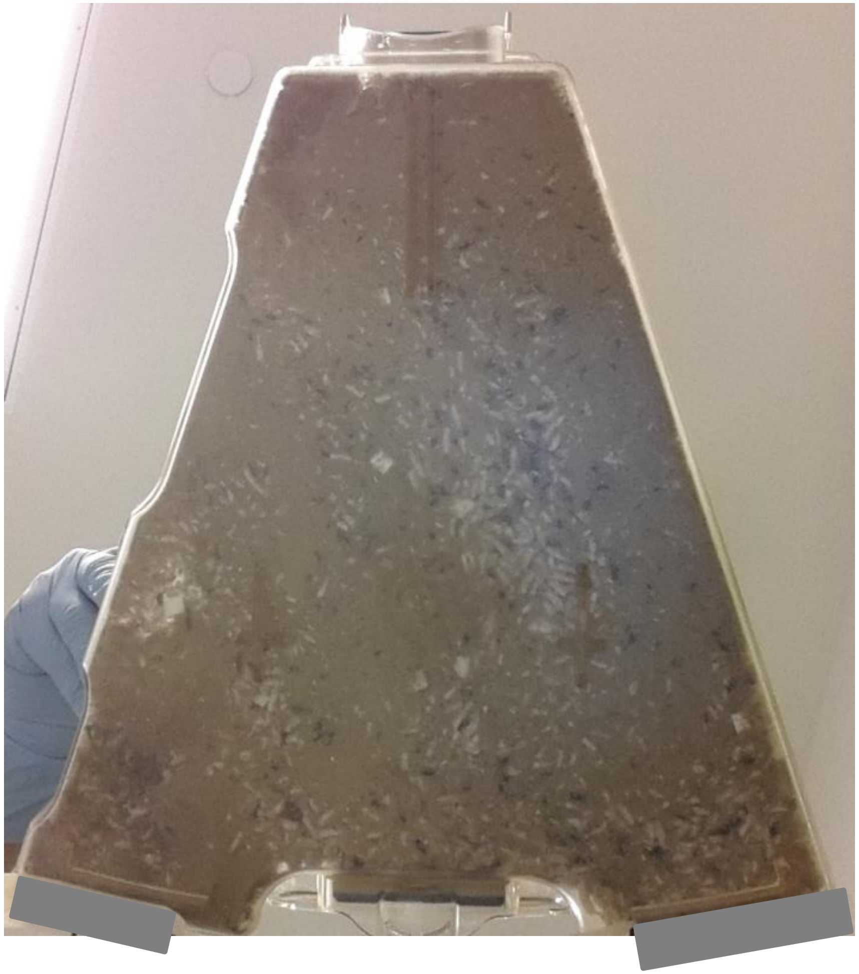

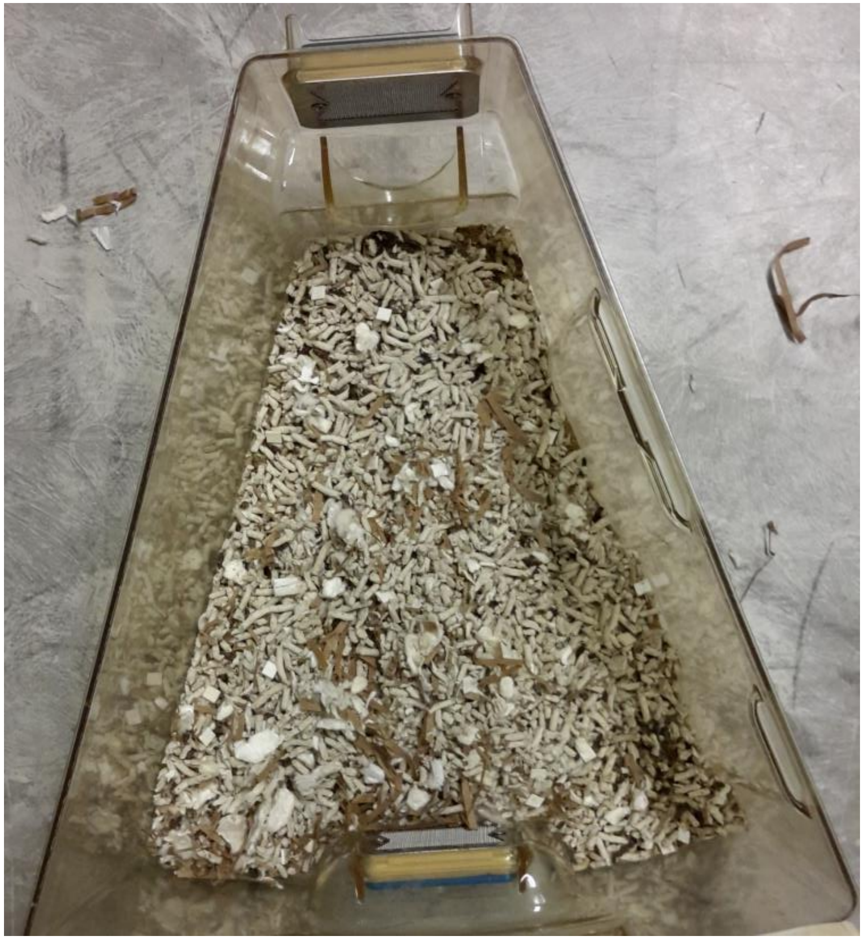

Sept 9 COMP 1 mid

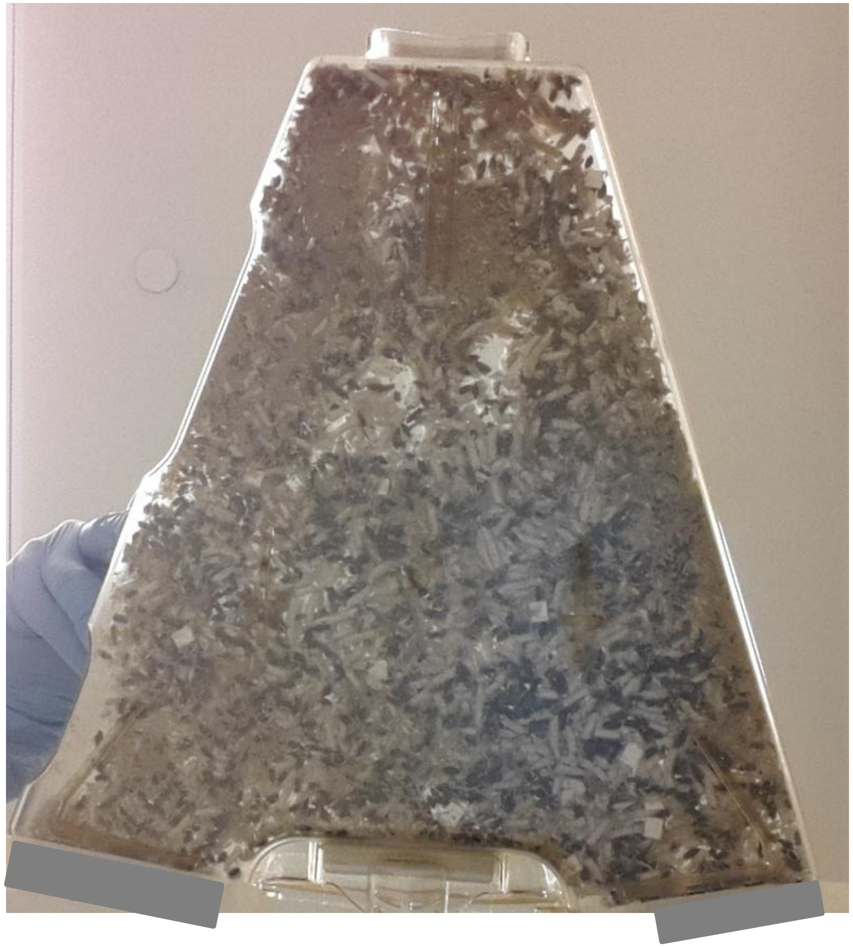

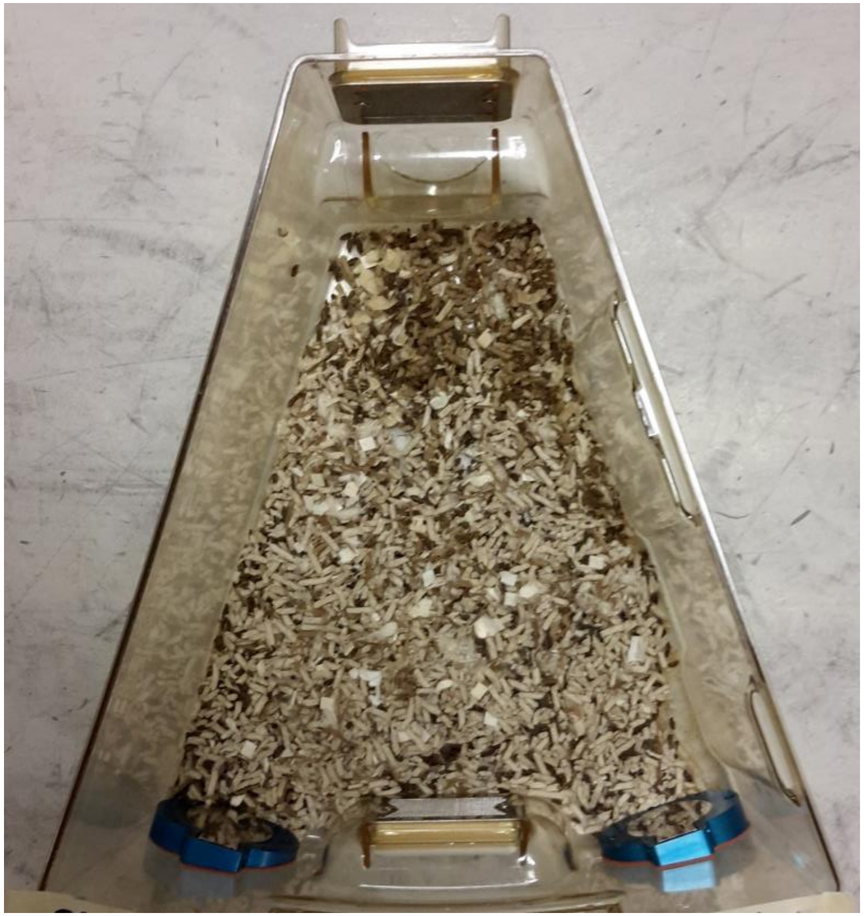

Sept 9 COMP 1 left

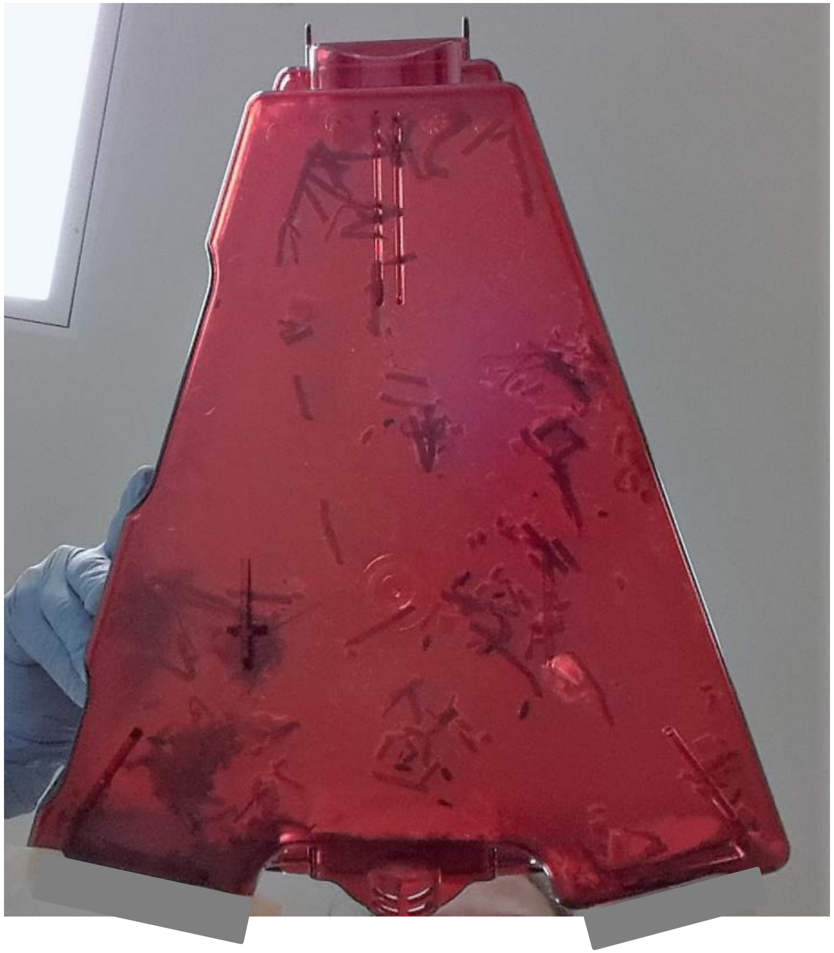

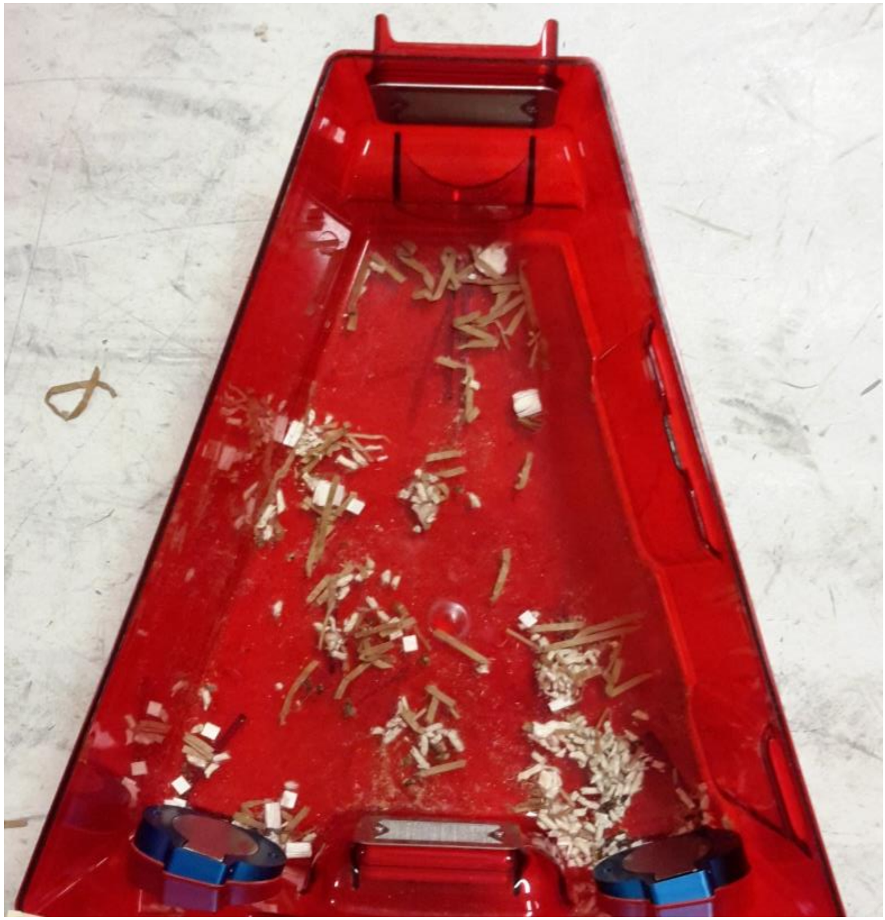

Sept 9 COMP 1 right

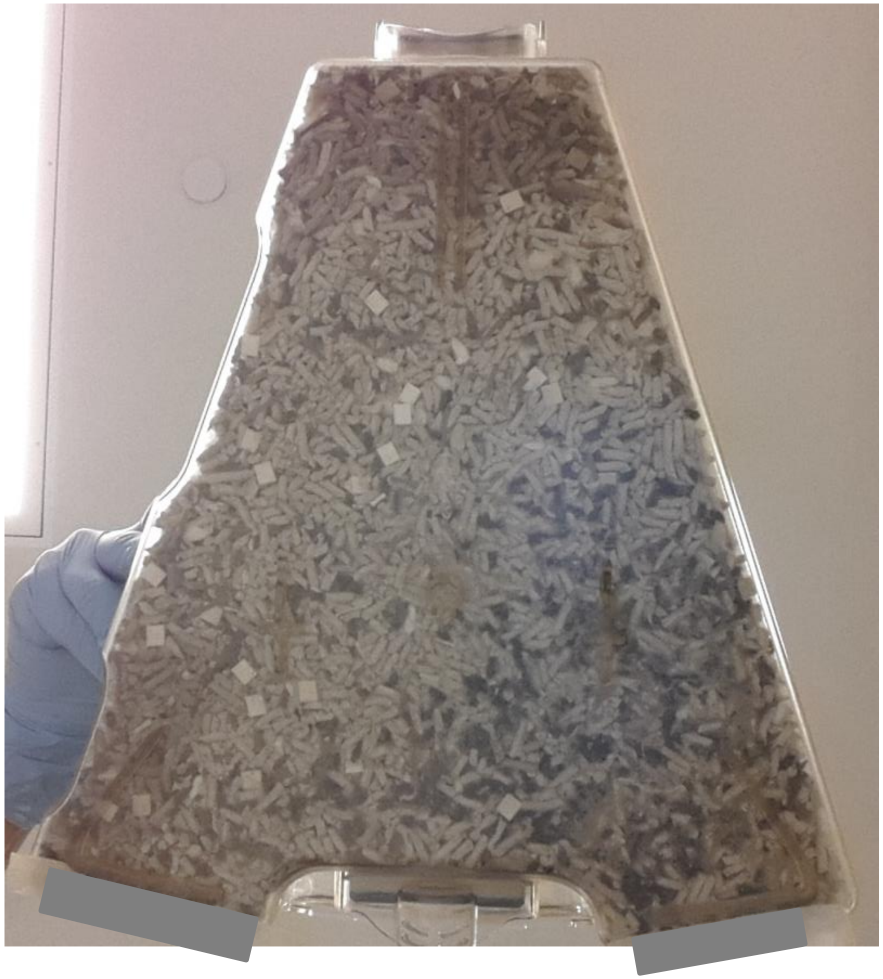

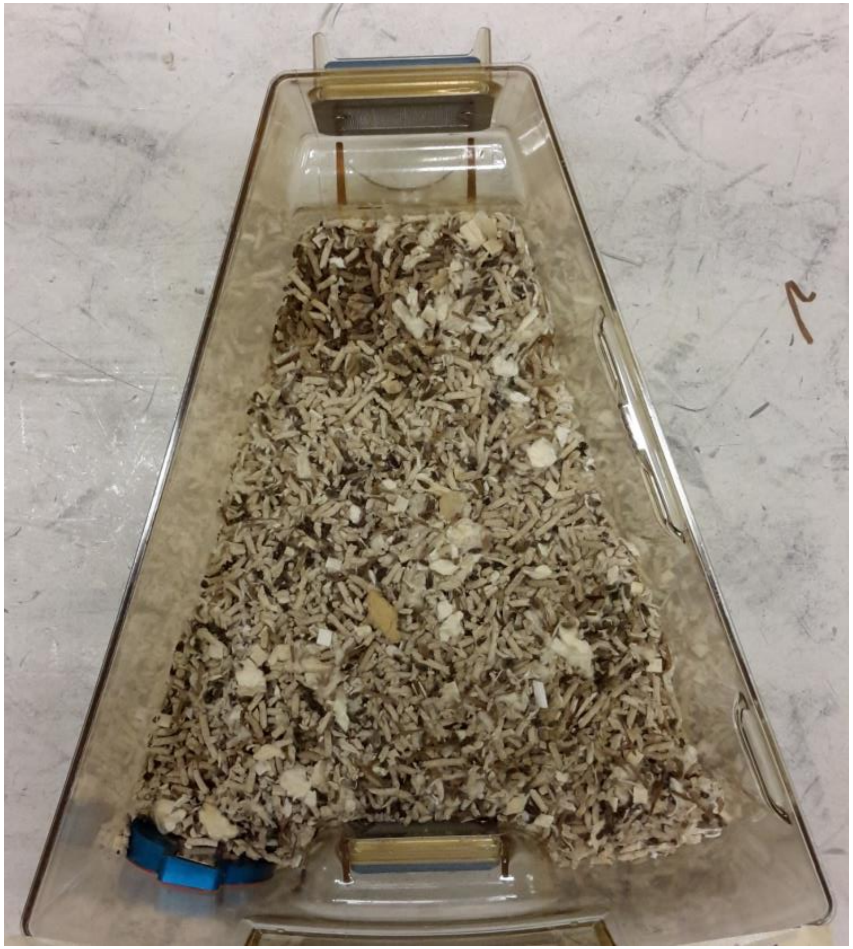

Sept 9 STD 1

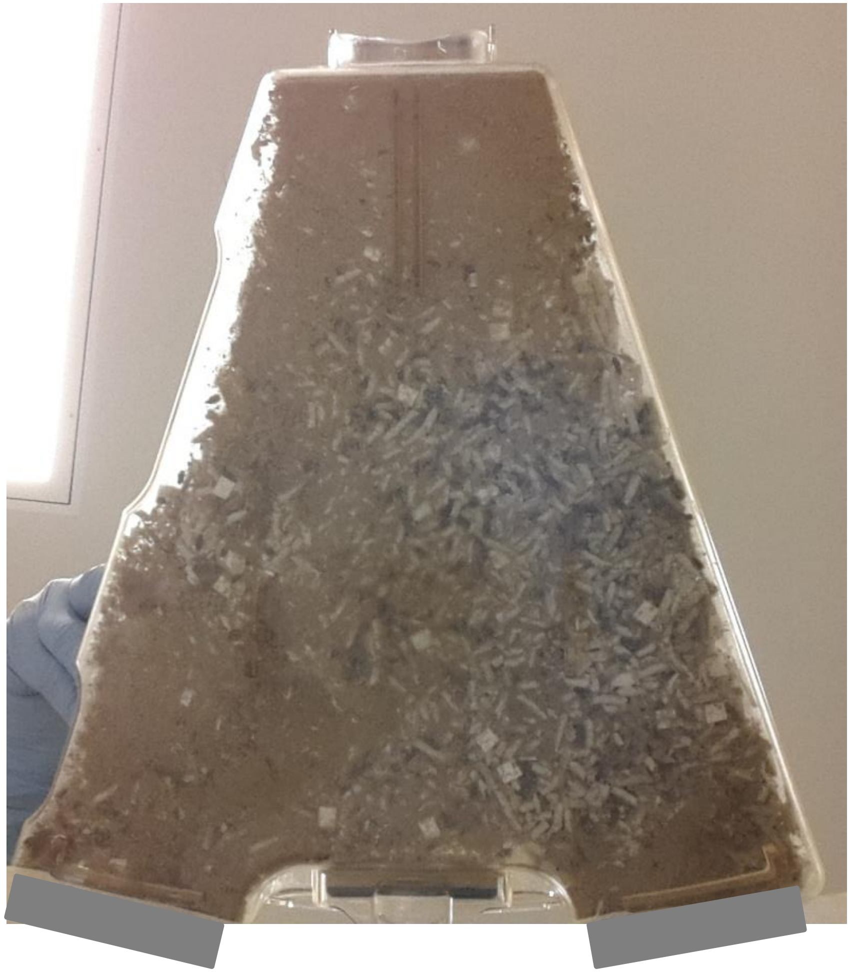

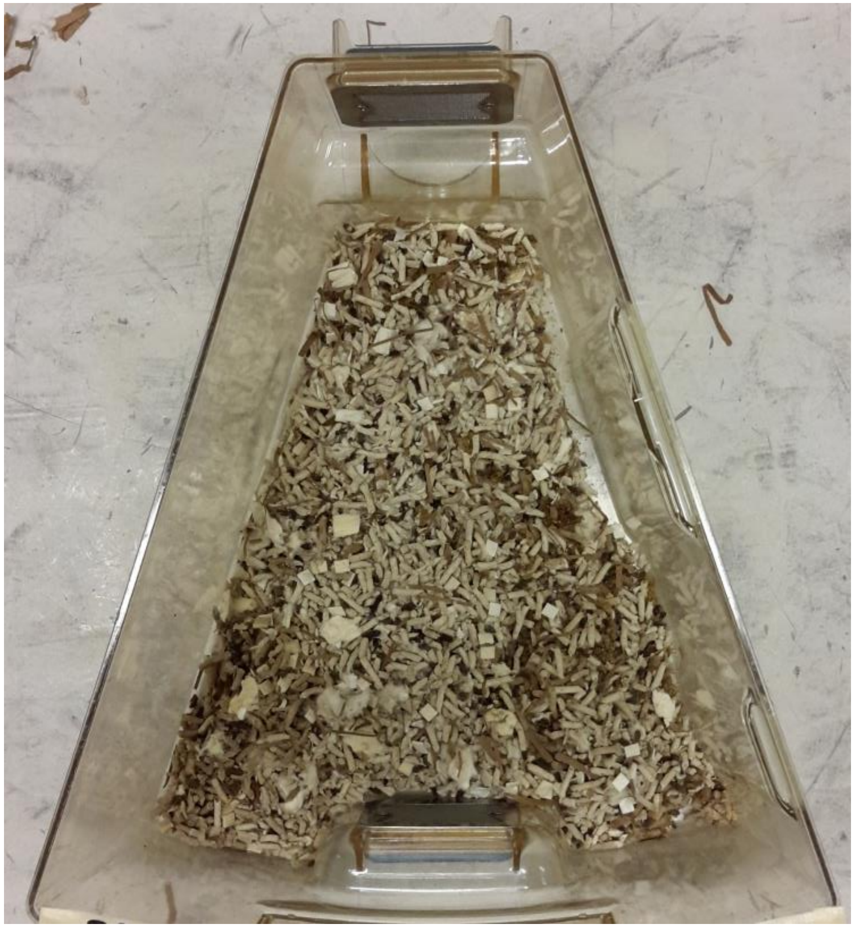

Sept 9 COMP 2 left

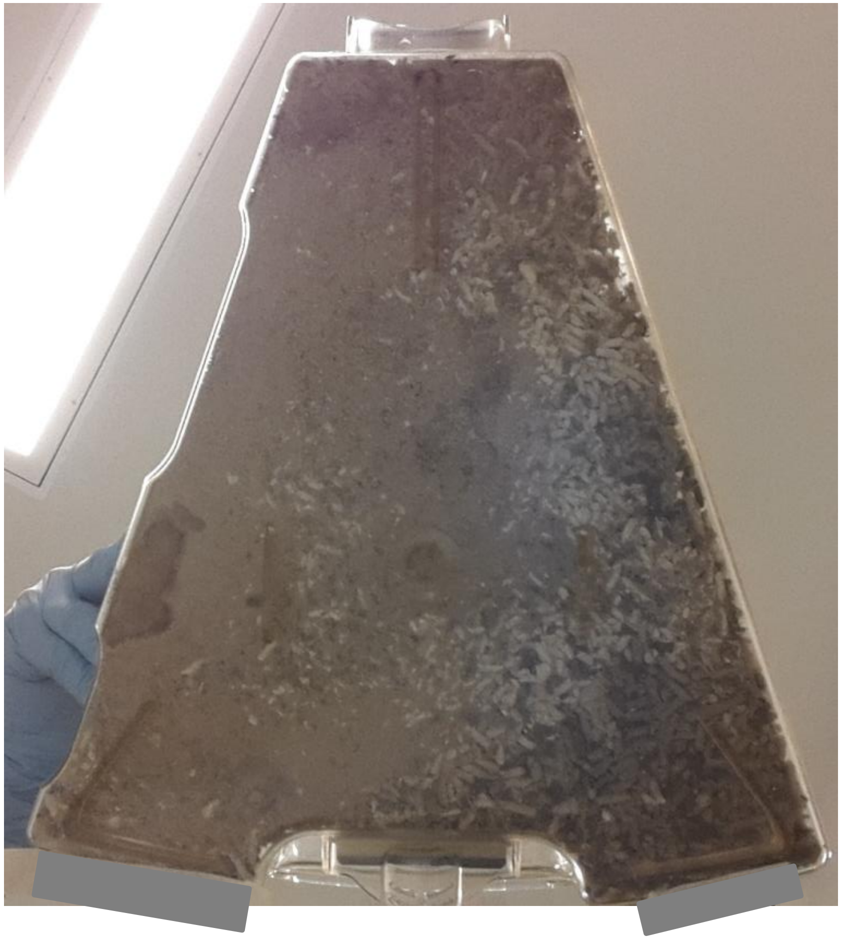

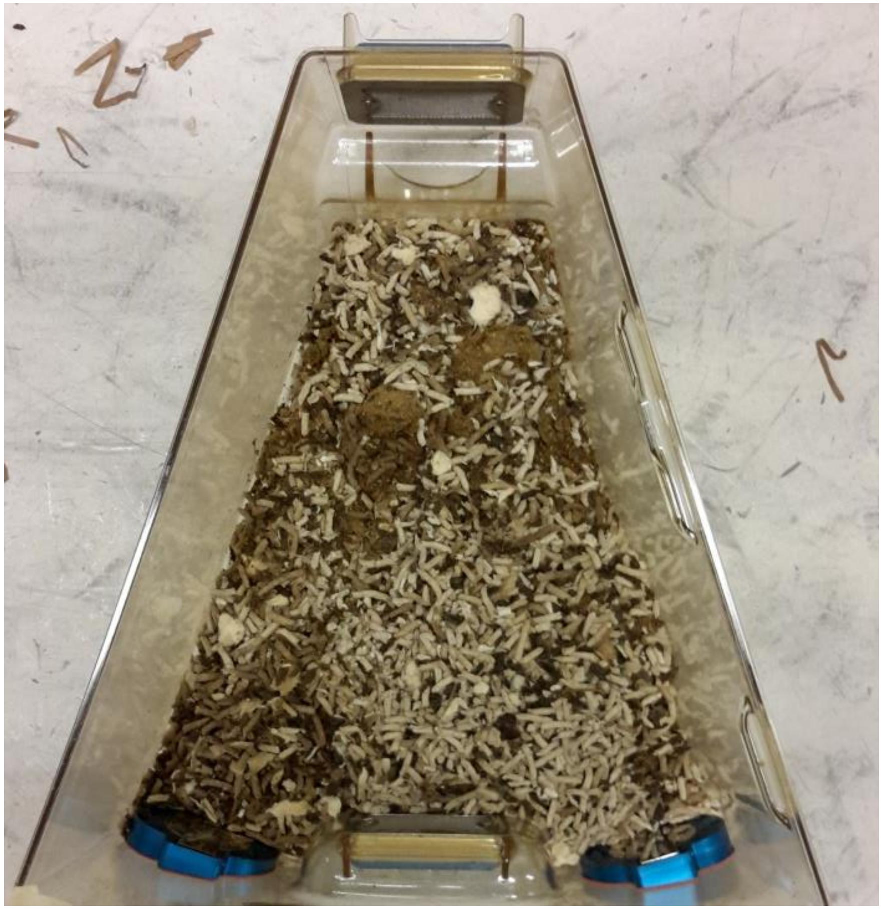

Sept 9 COMP 2 mid

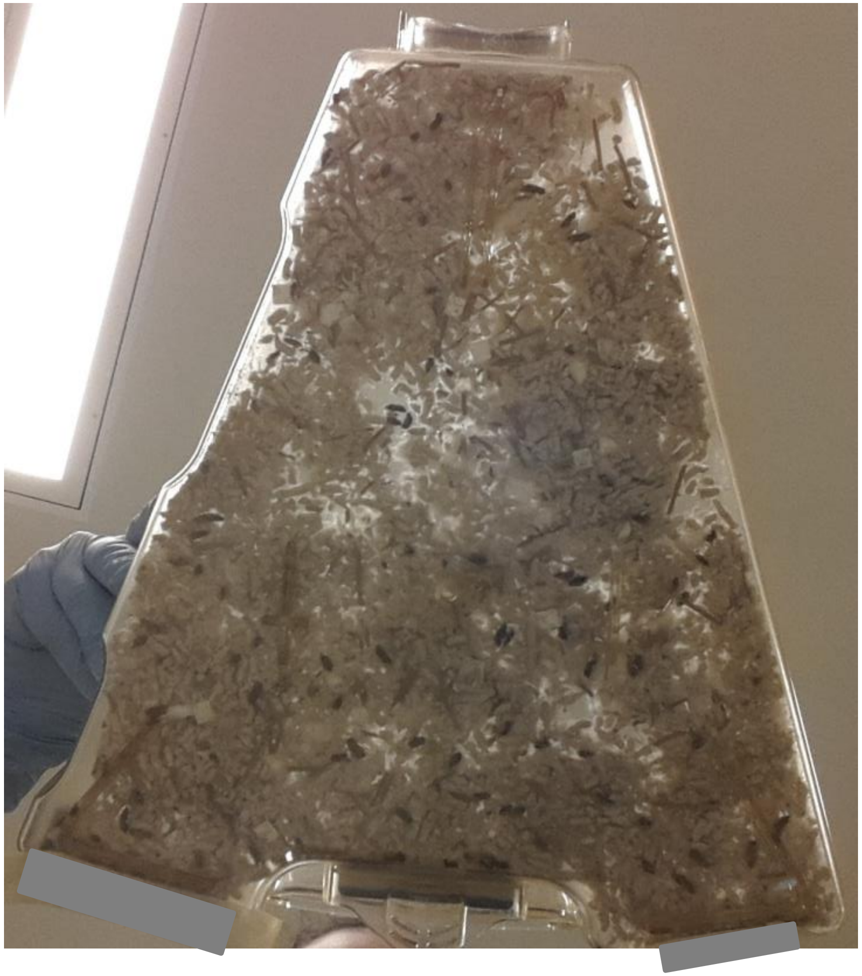

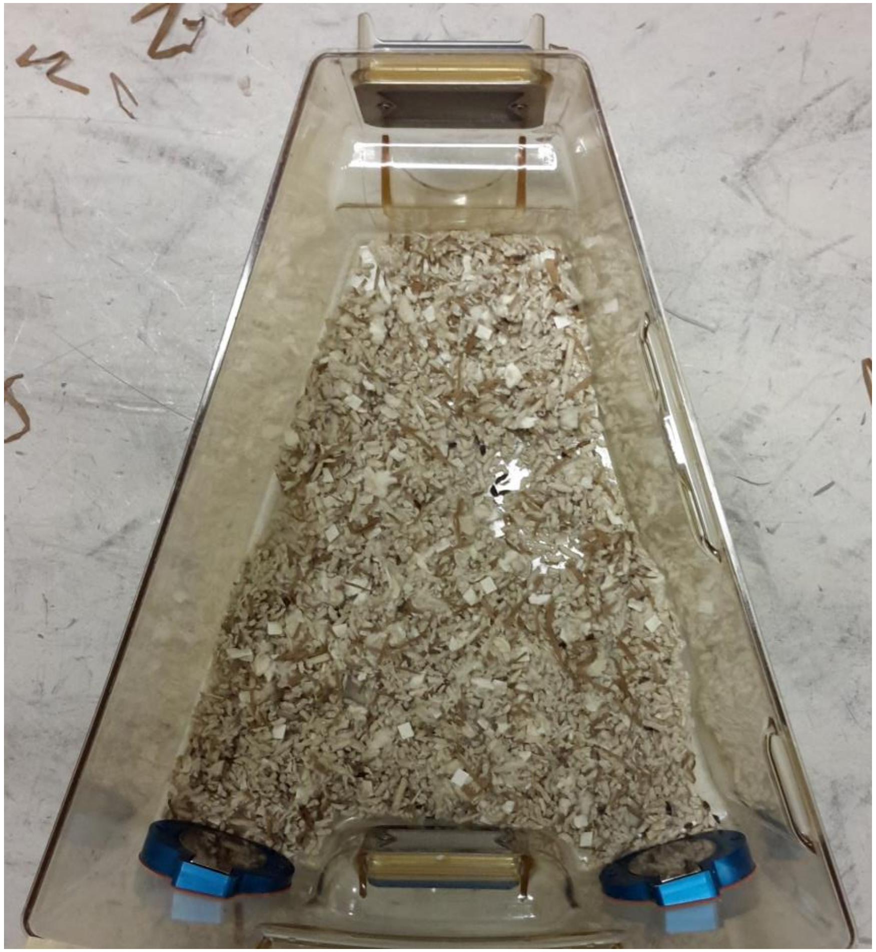

Sept 9 COMP 2 right

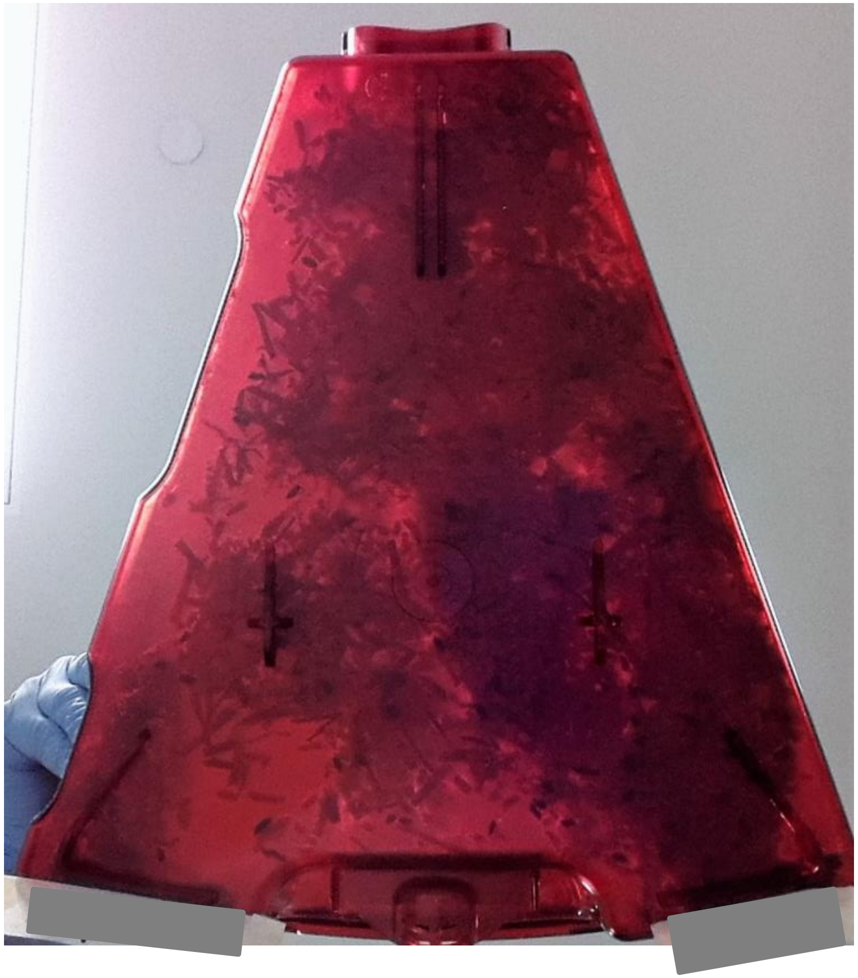

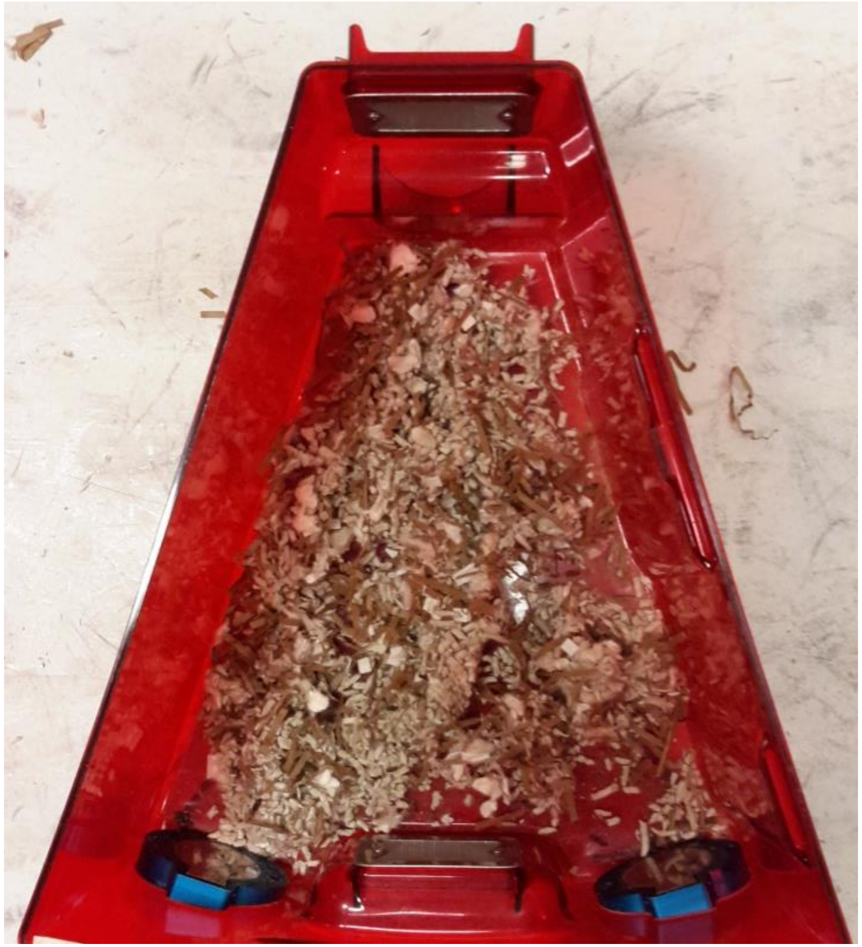

Sept 9 STD 2

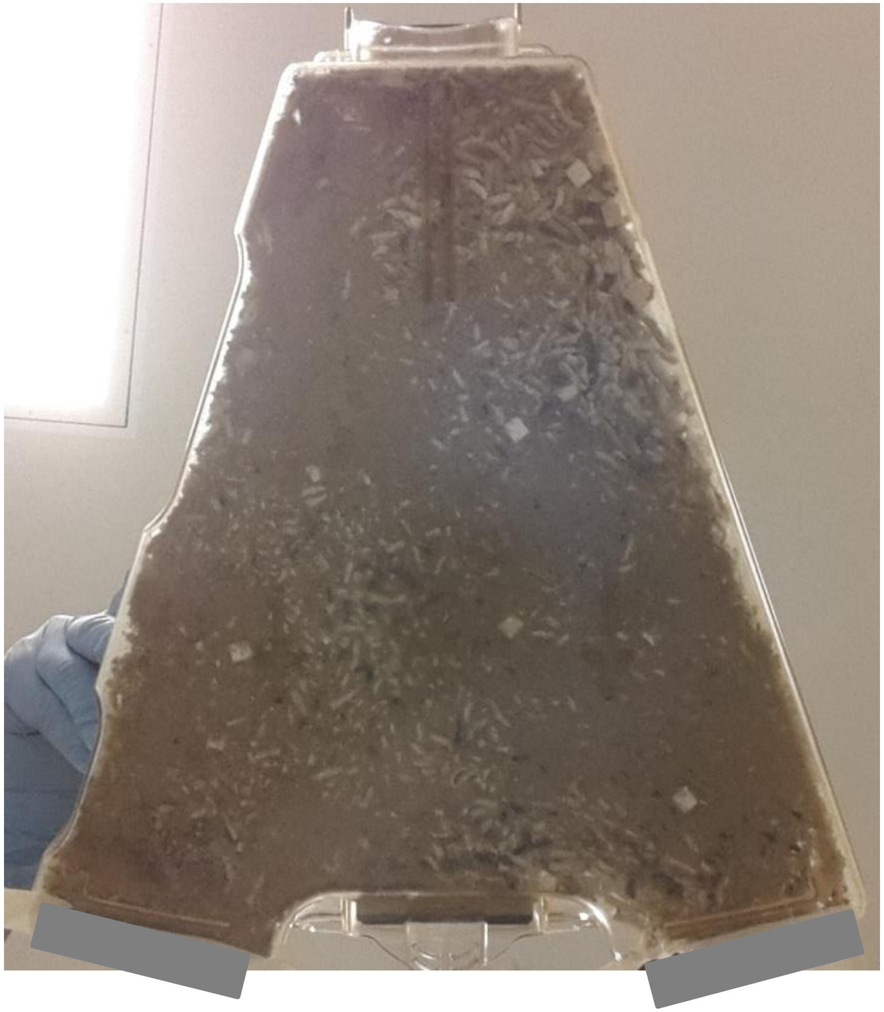

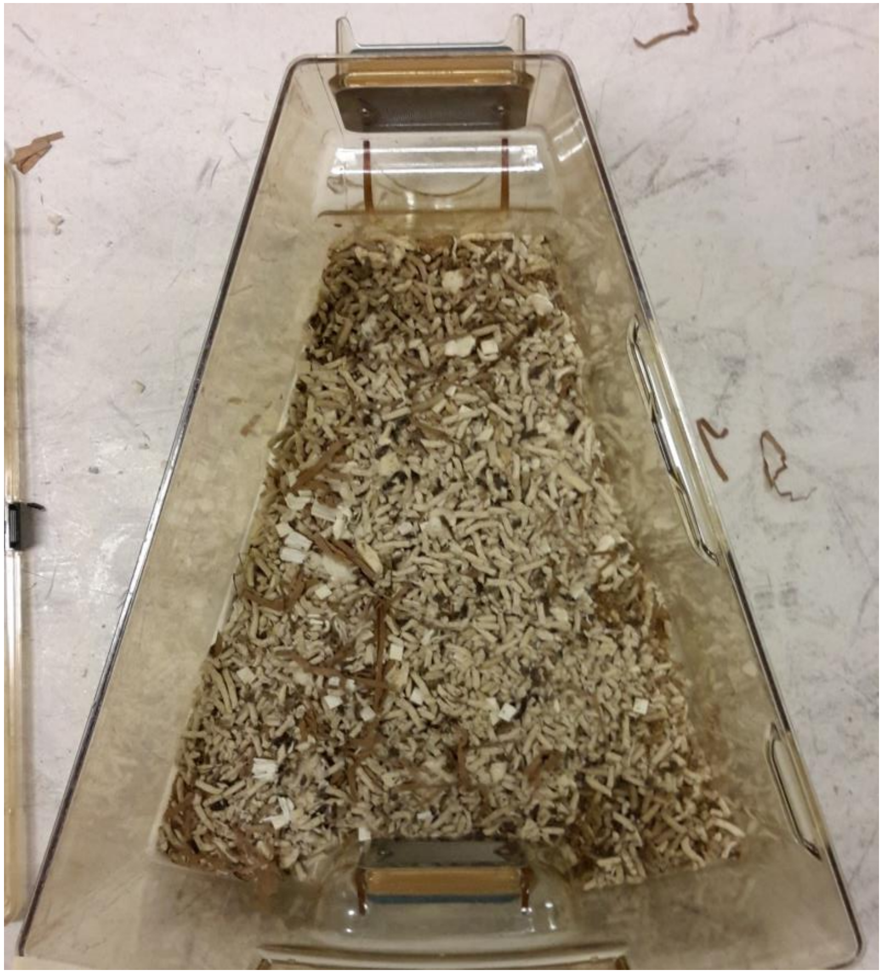

Sept 10 COMP 3 right

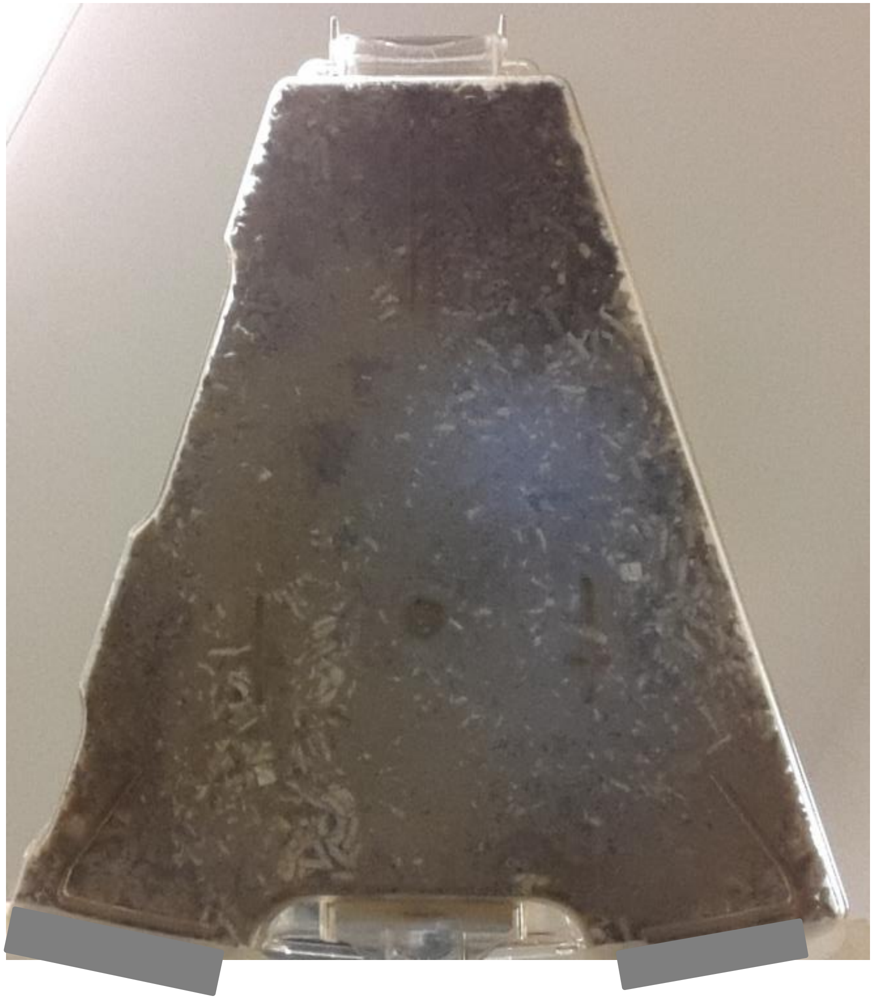

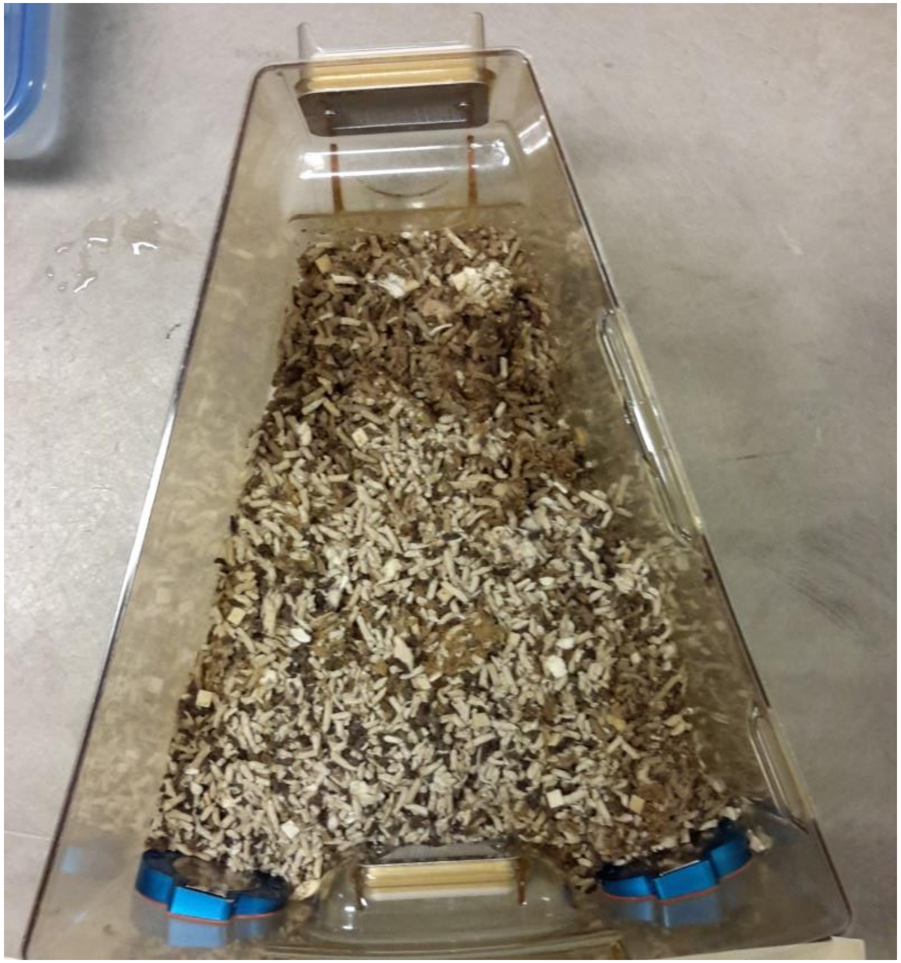

Sept 10 COMP 3 left

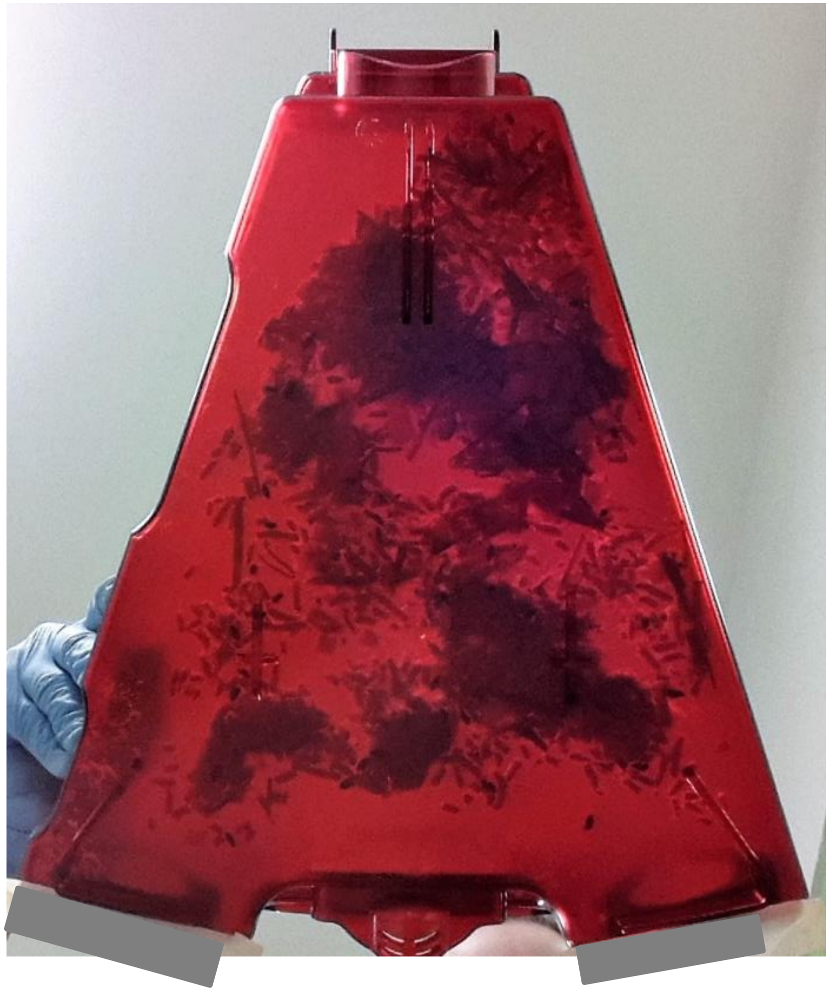

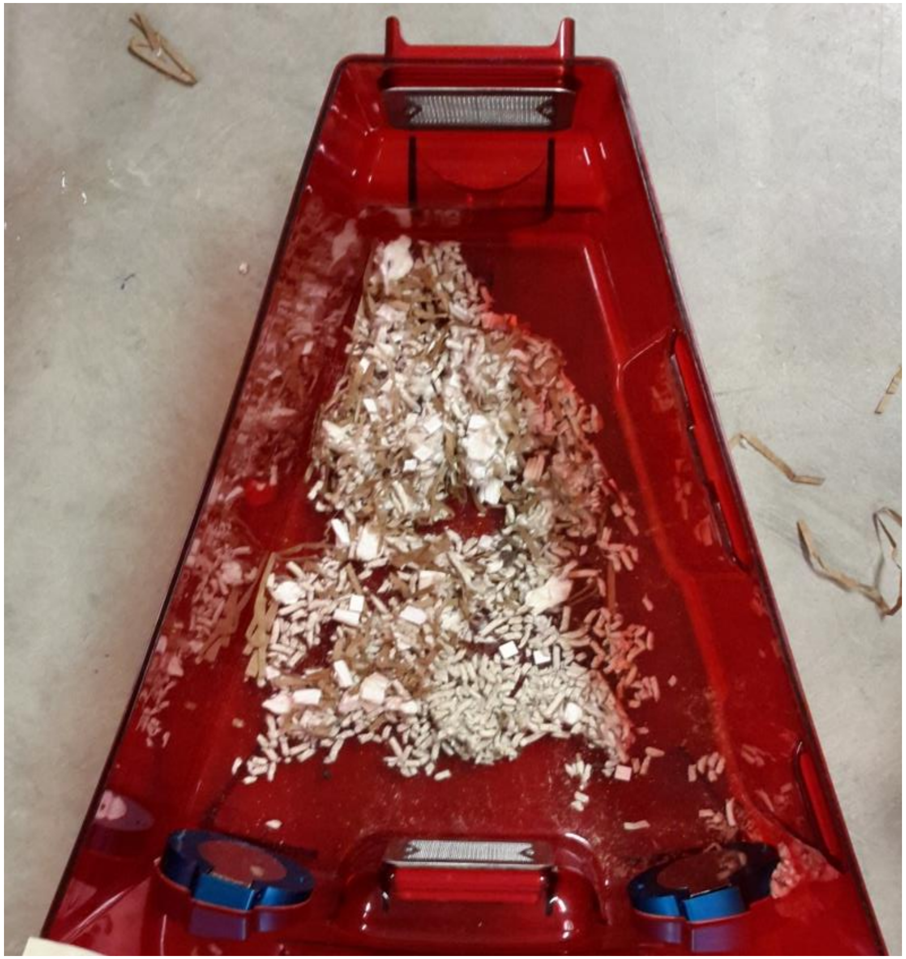

Sept 10 COMP 3 mid

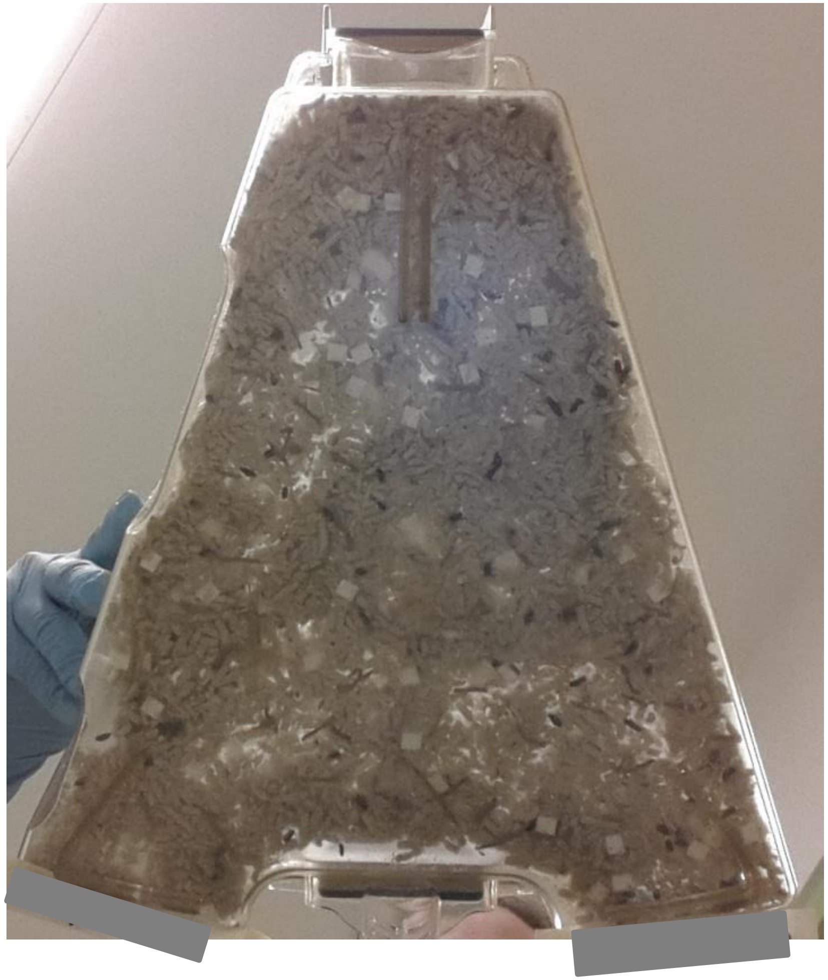

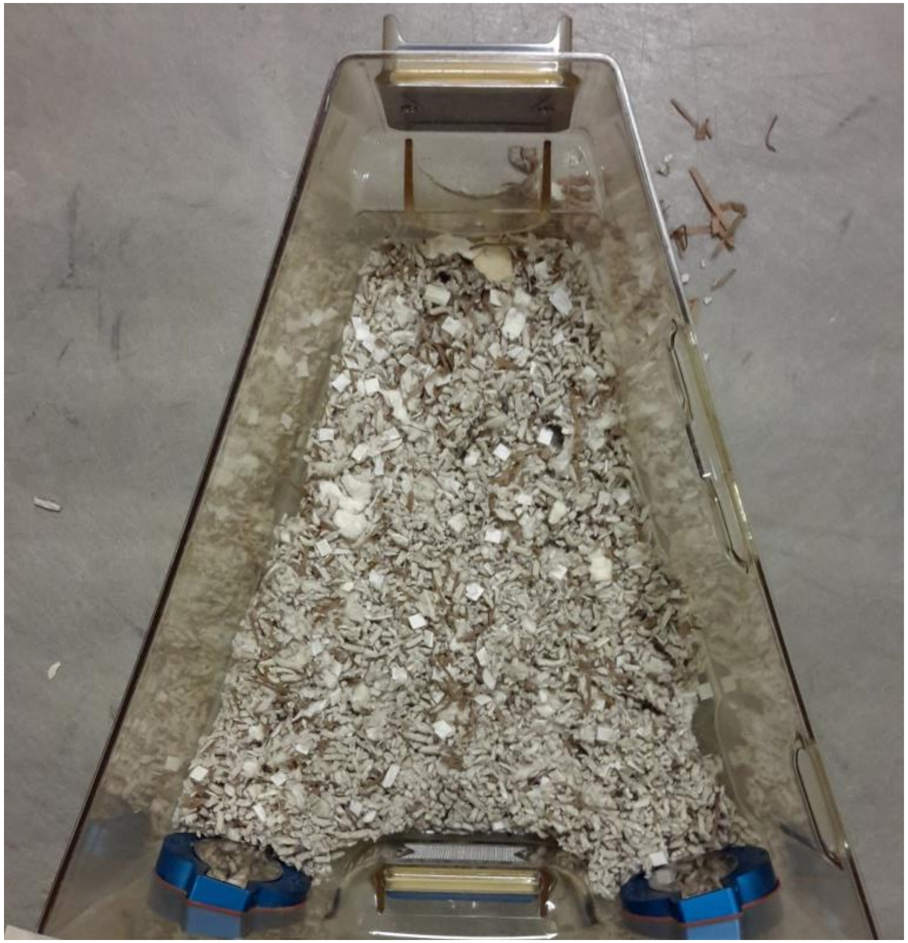

Sept 10 STD 3

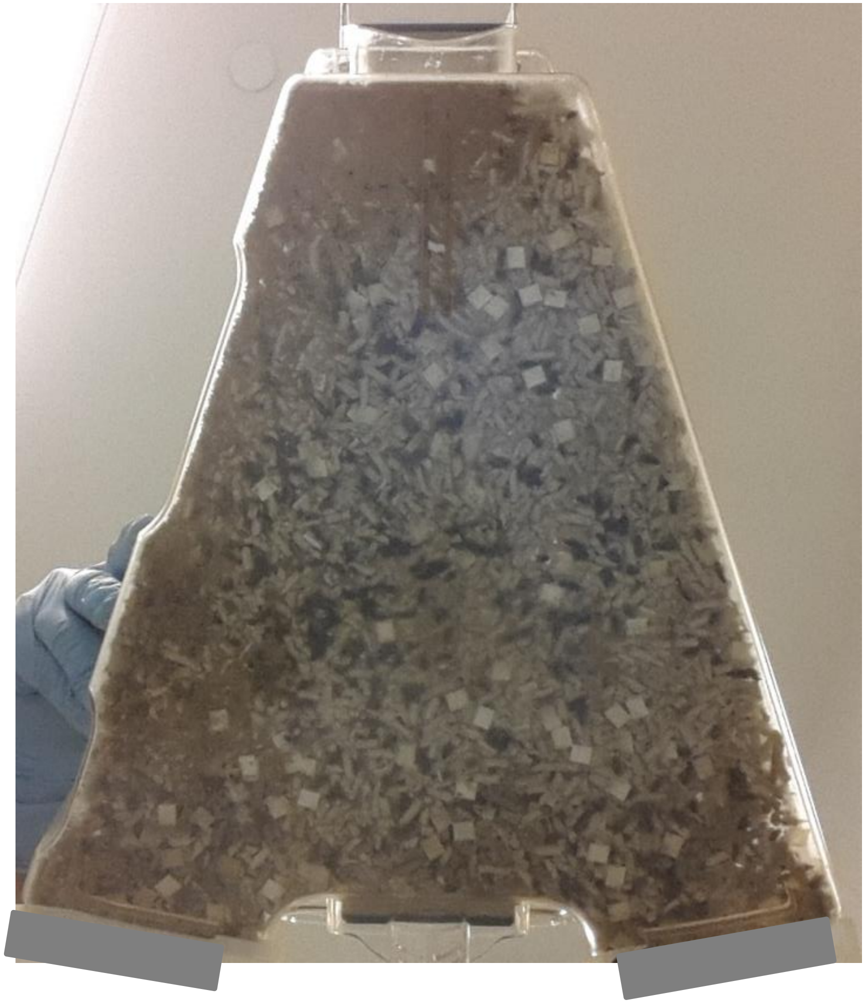

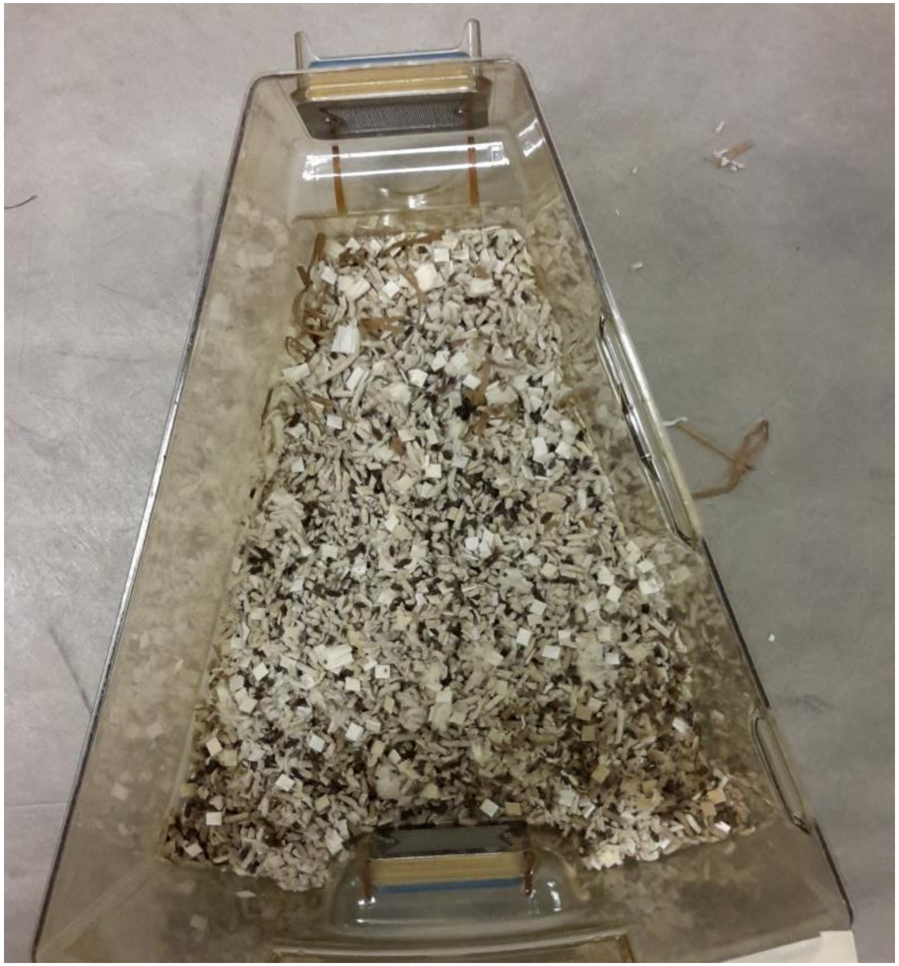

Sept 10 COMP 4 mid

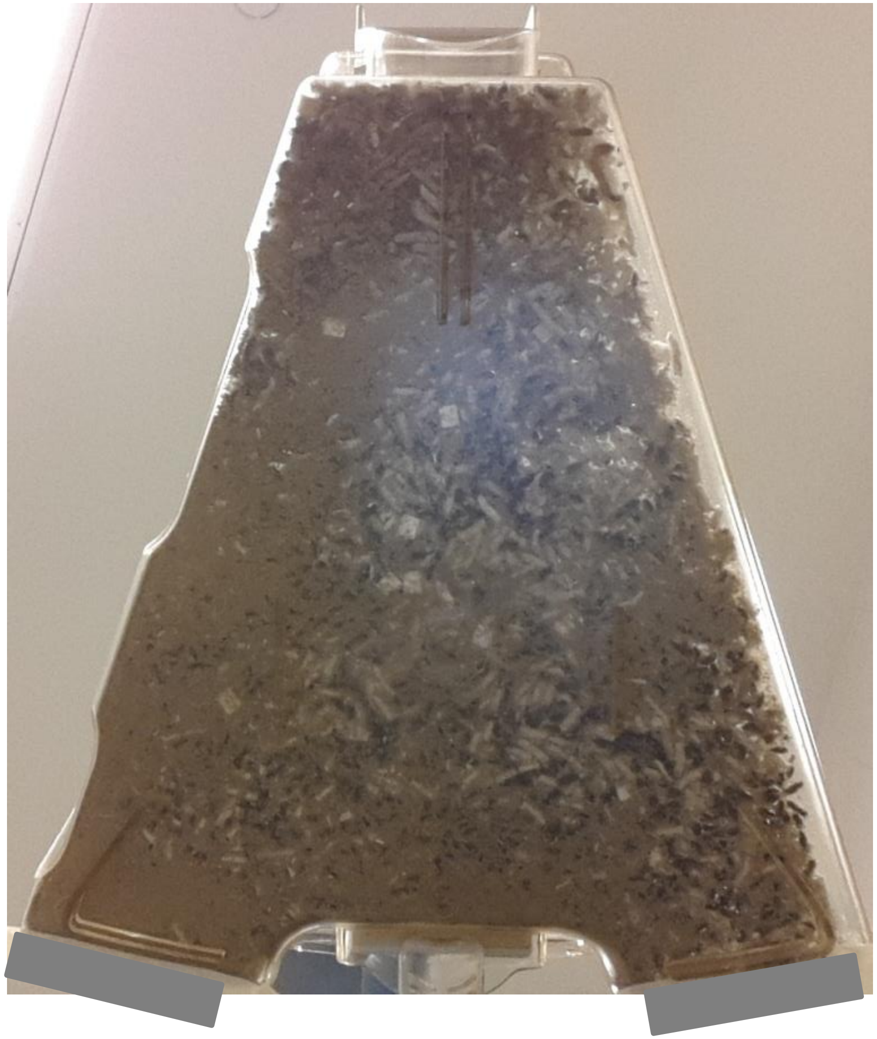

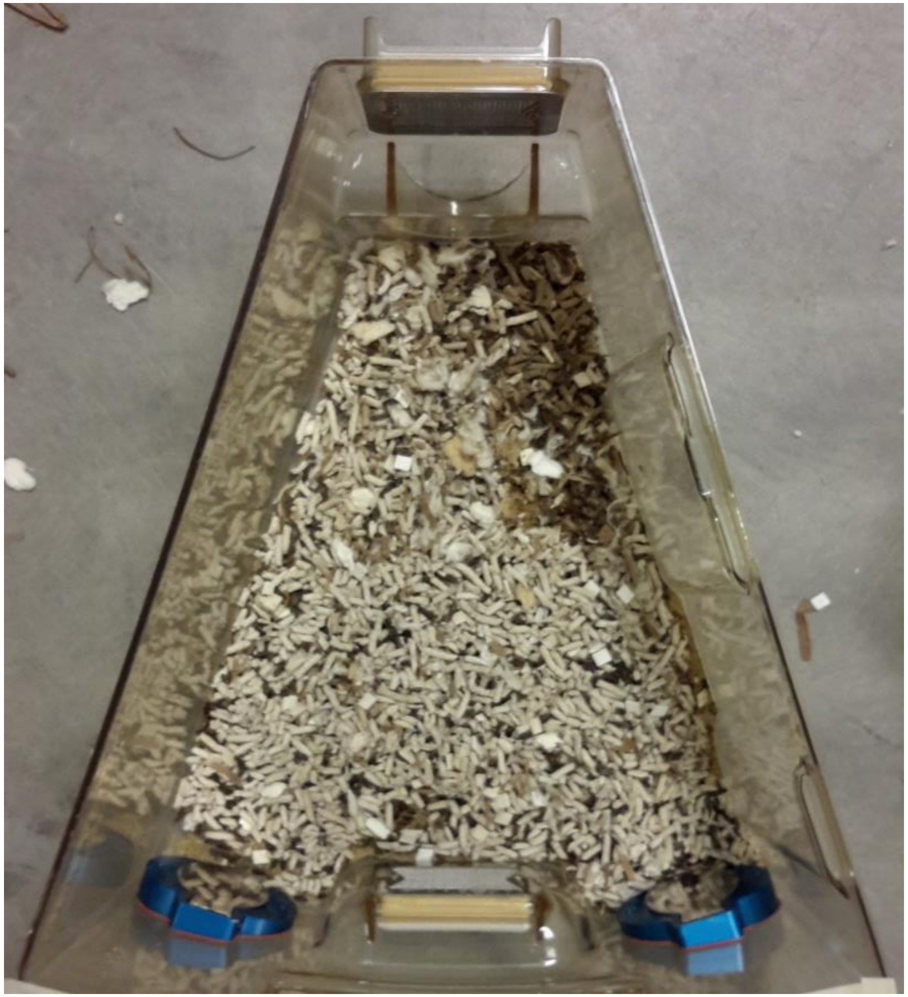

Sept 10 COMP 4 right

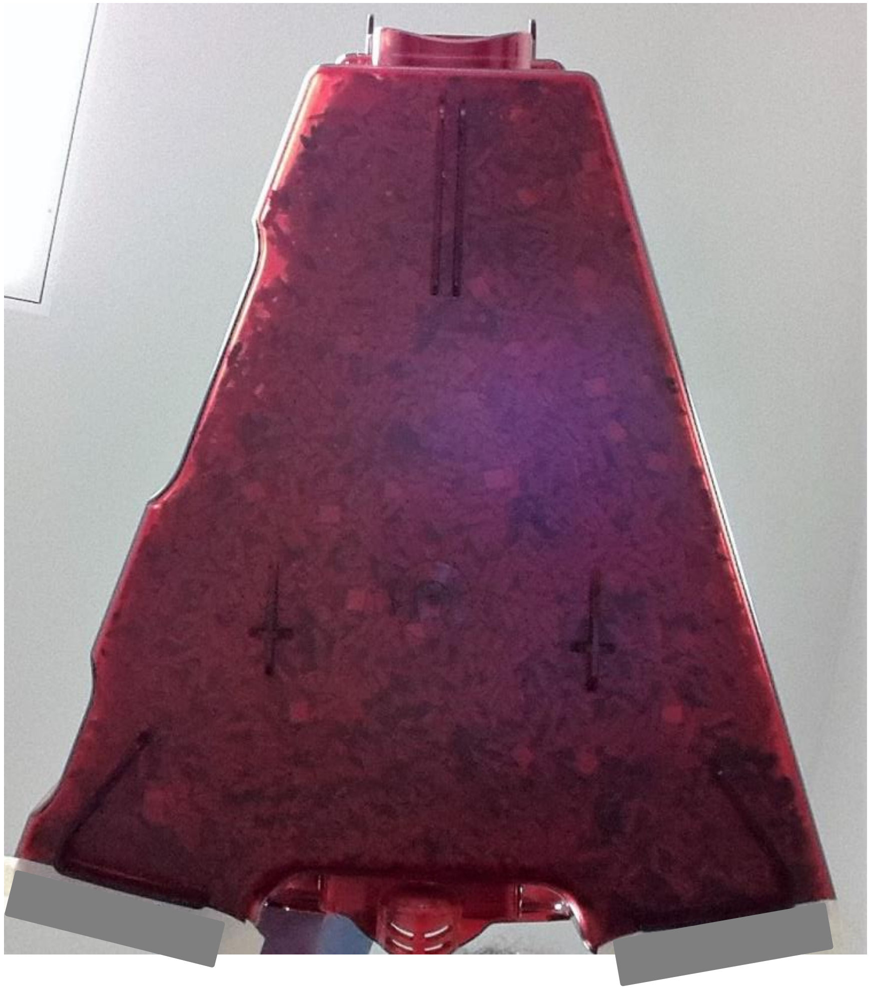

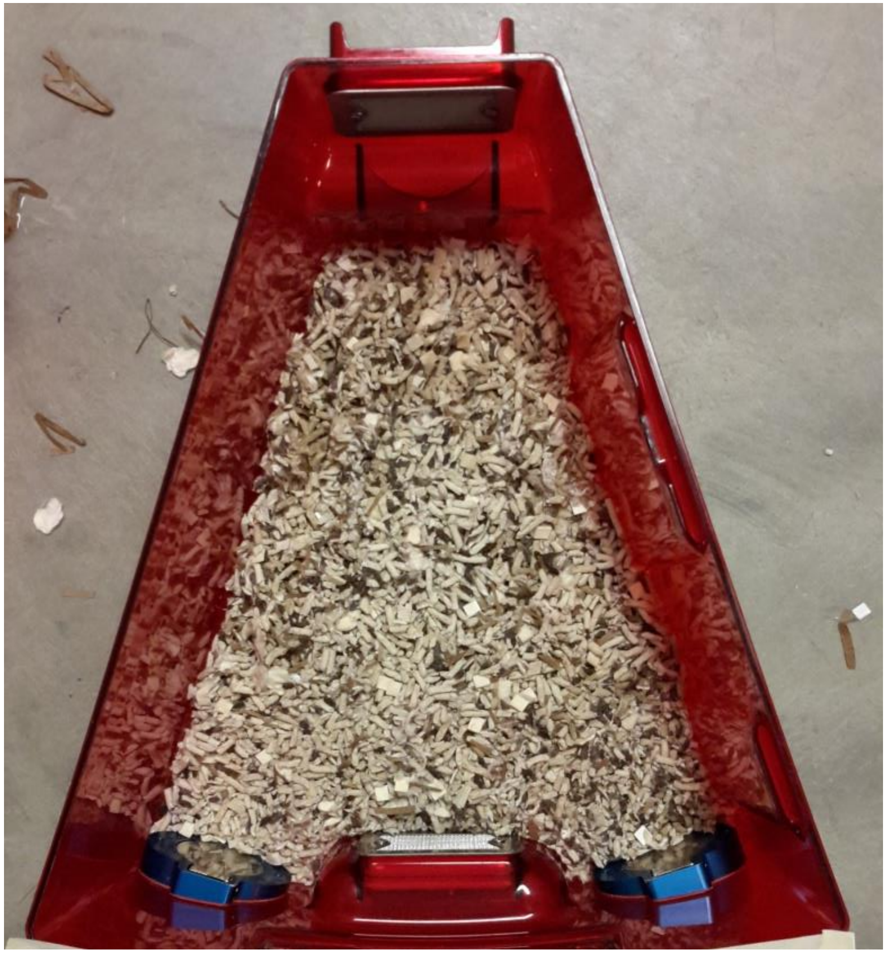

Sept 10 COMP 4 left

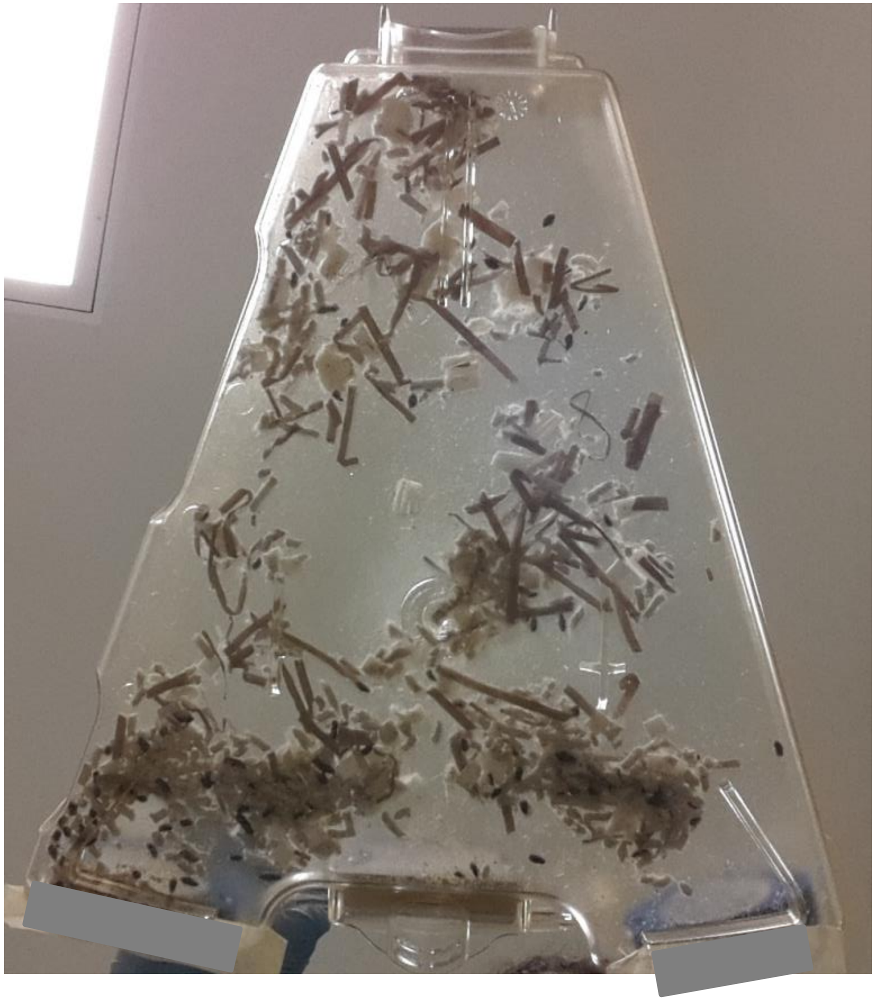

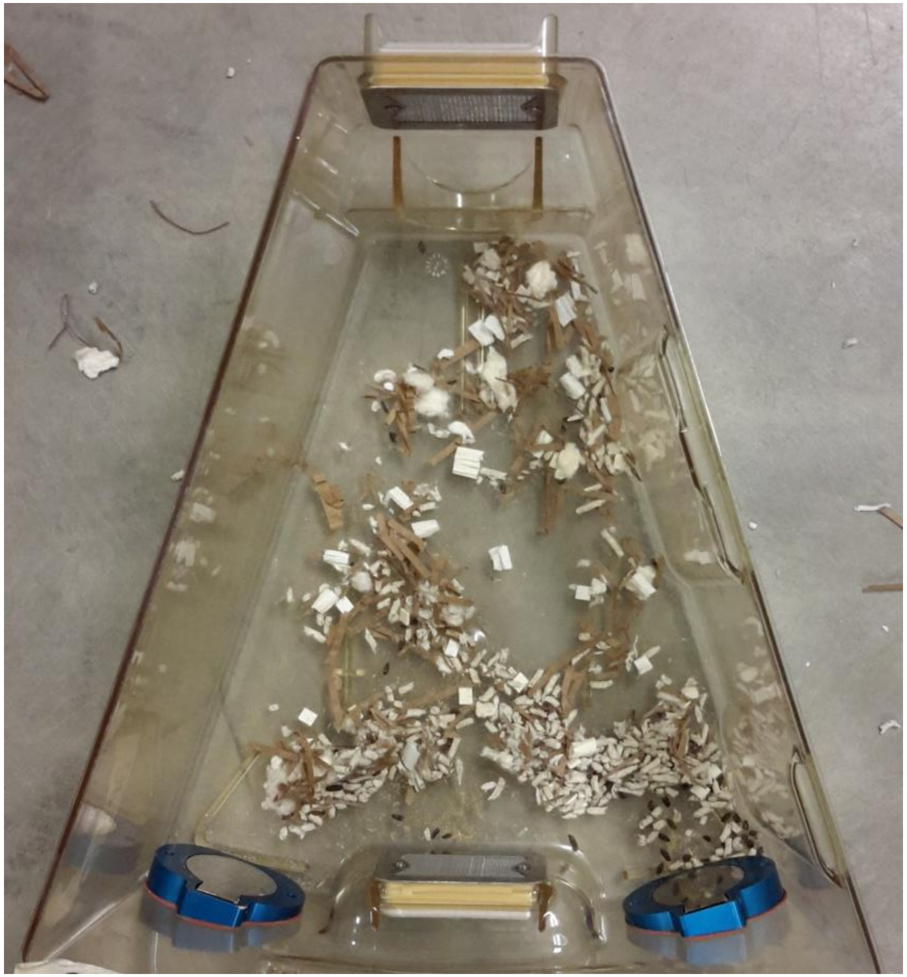

Sept 10 STD 4

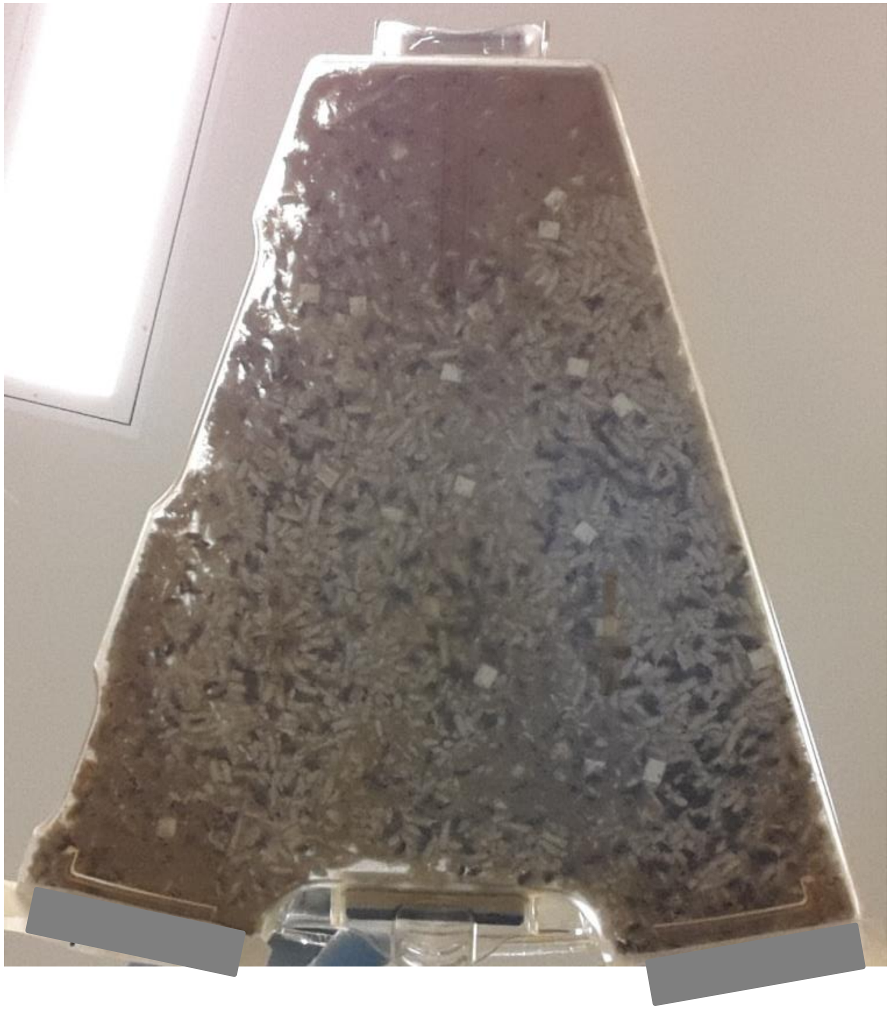

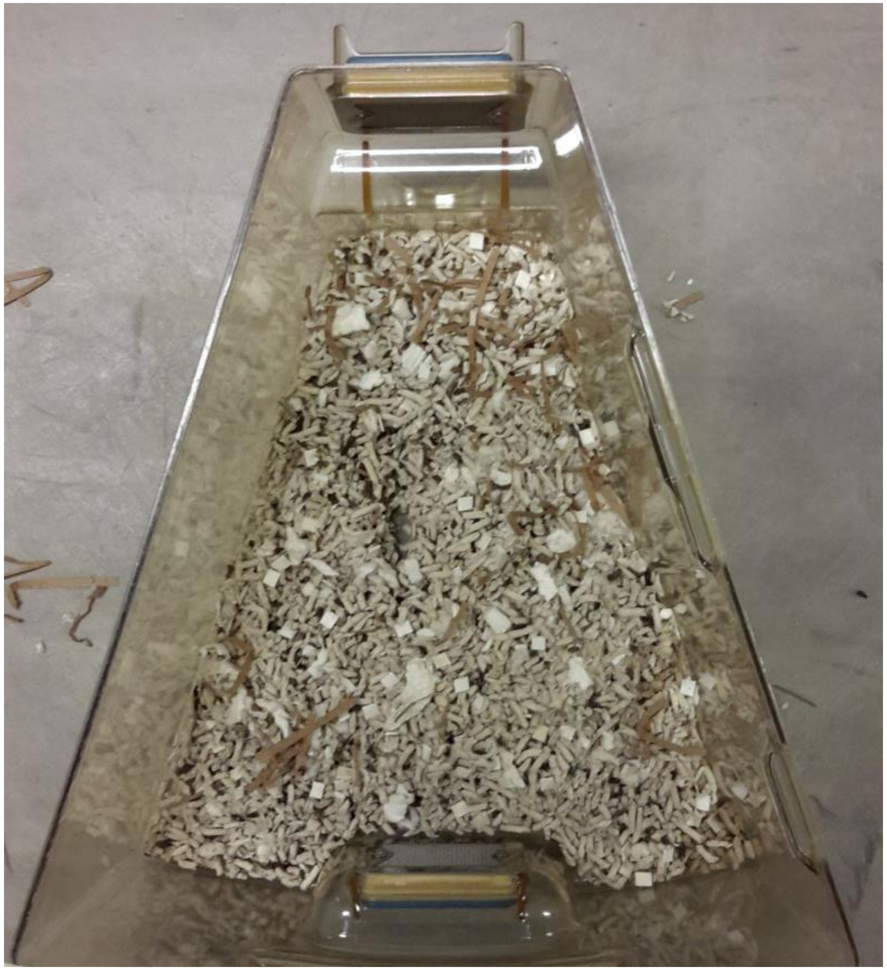

Sept 11 COMP 5 right

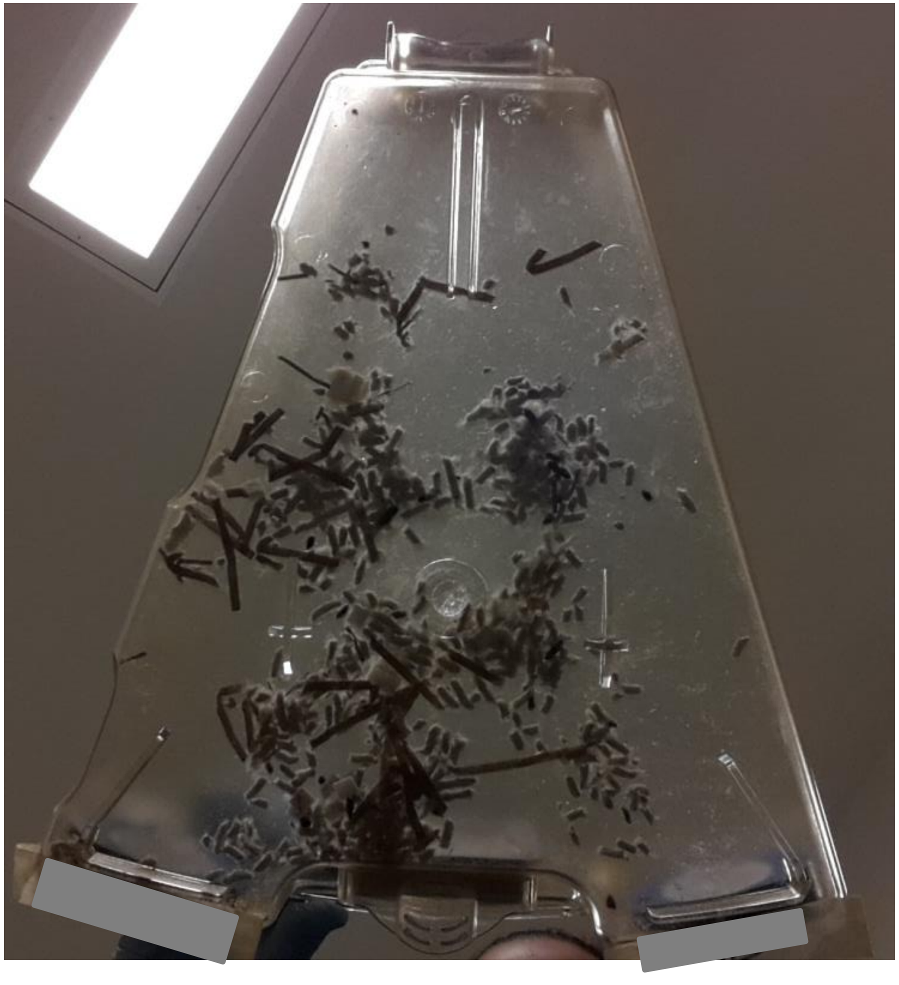

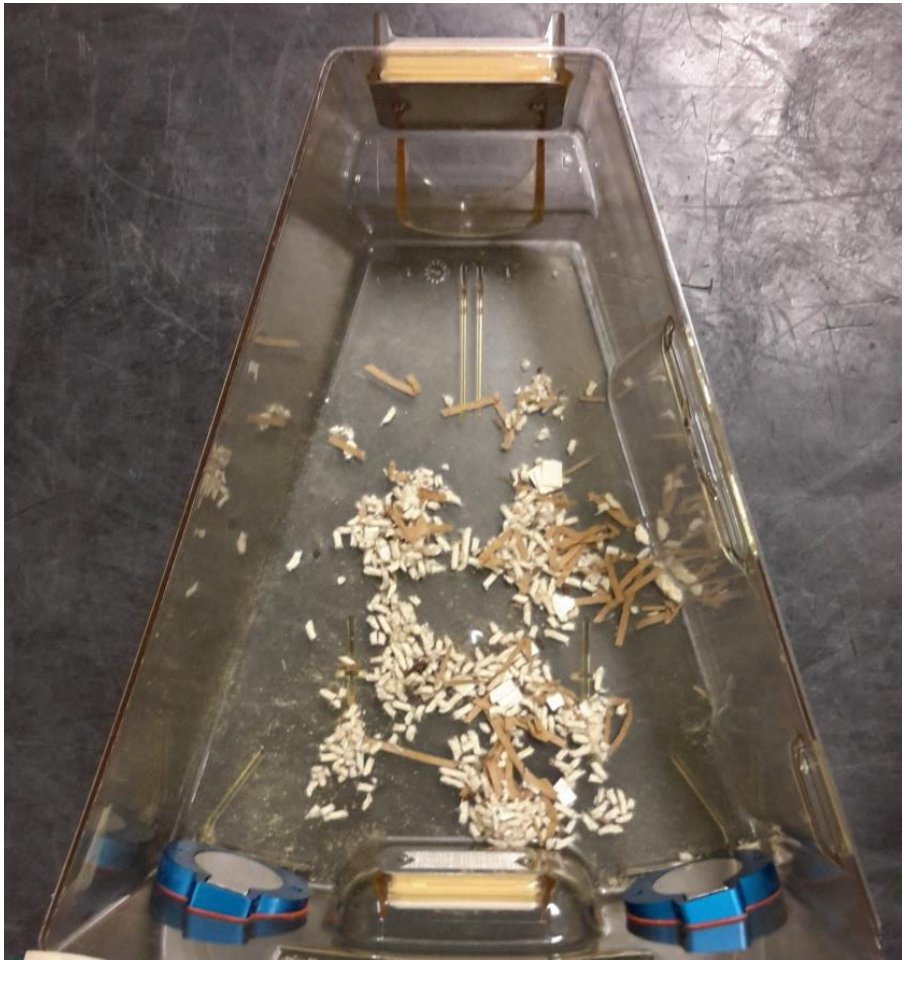

Sept 11 COMP 5 mid

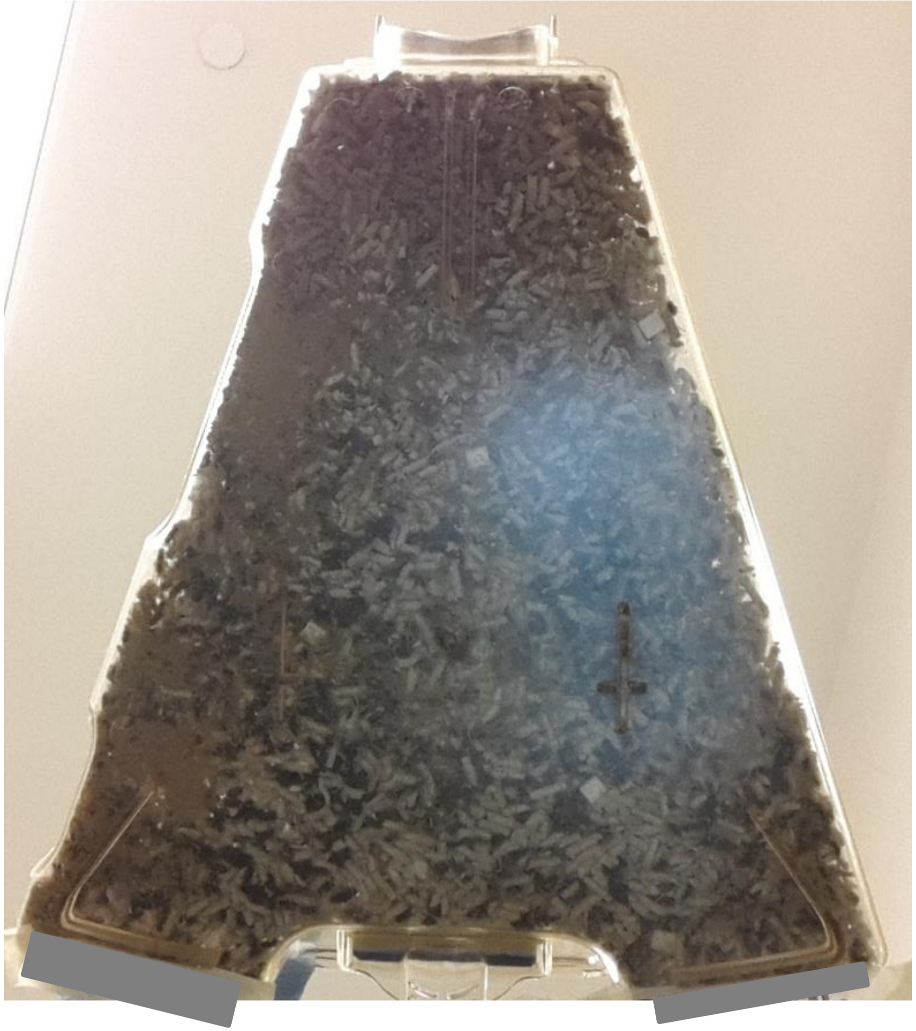

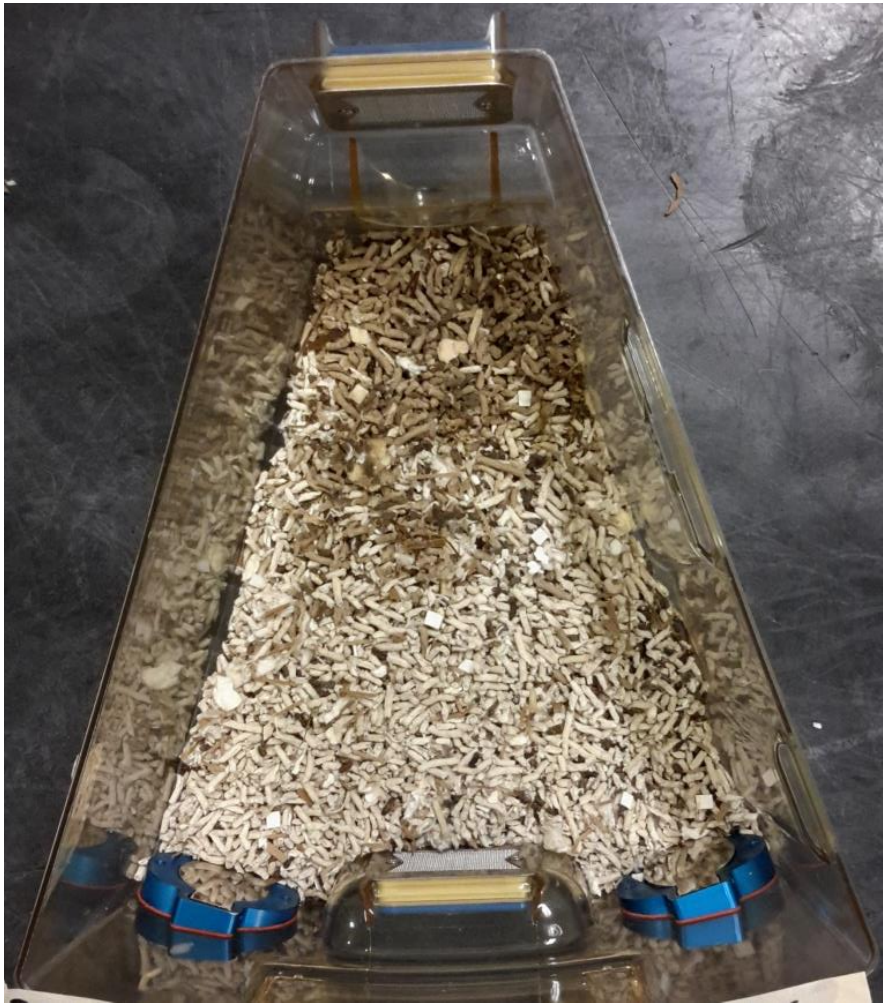

Sept 11 COMP 5 left

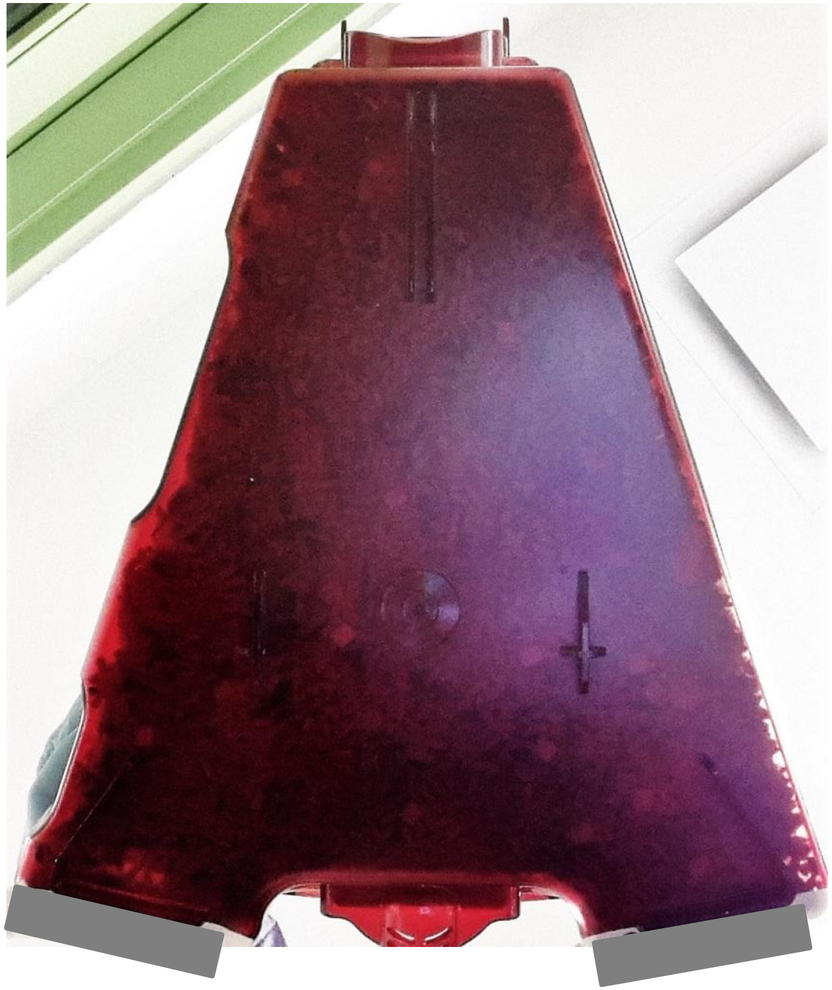

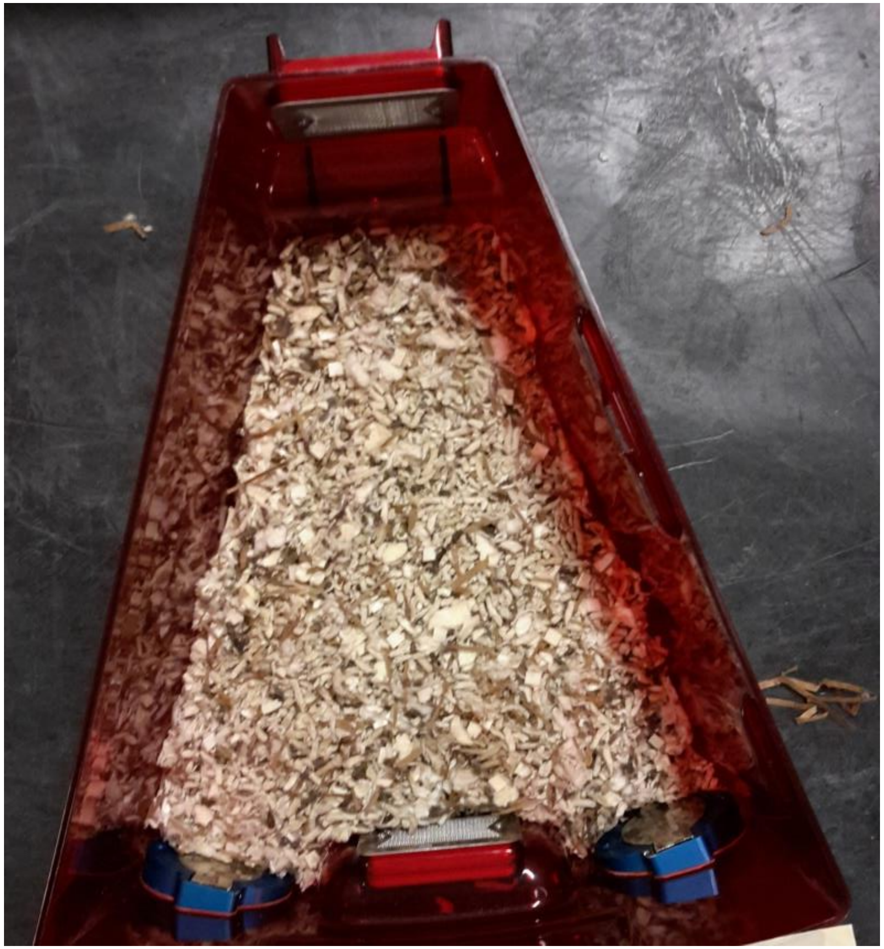

Sept 11 STD 5

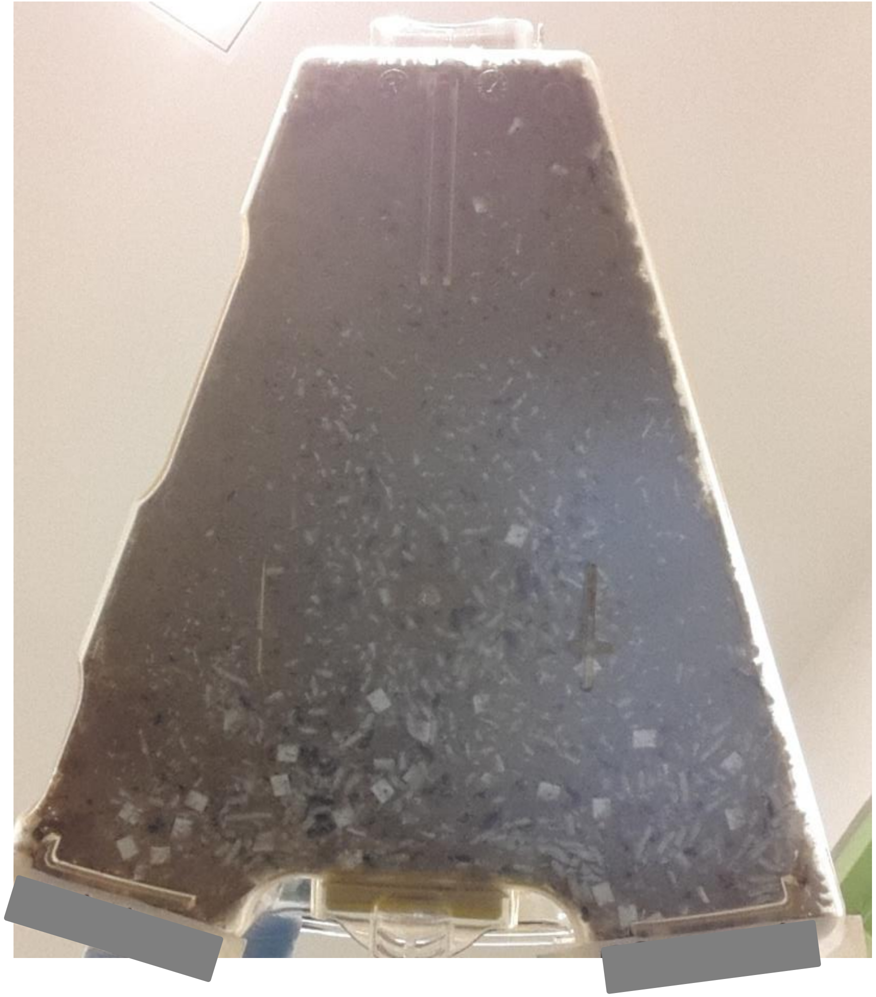

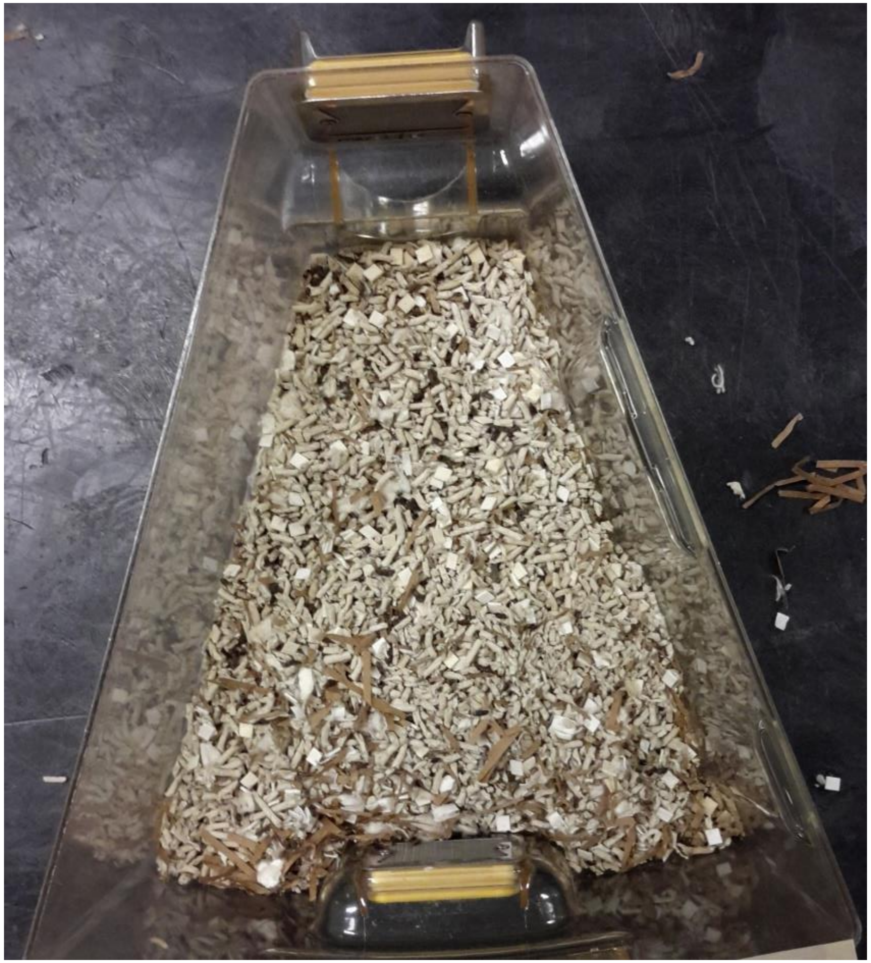

Supplement: Supplementary file 2 — Supplementary Dataset 2 [file 41598_2019_42512_MOESM2_ESM.pdf]
